# Identification and Functional Analysis of Healing Regulators in *Drosophila*

**DOI:** 10.1371/journal.pgen.1004965

**Published:** 2015-02-03

**Authors:** Carmen Álvarez-Fernández, Srividya Tamirisa, Federico Prada, Ariel Chernomoretz, Osvaldo Podhajcer, Enrique Blanco, Enrique Martín-Blanco

**Affiliations:** 1 Instituto de Biología Molecular de Barcelona, Consejo Superior de Investigaciones Científicas. Parc Cientific de Barcelona, Barcelona, Spain; 2 Terapia Molecular y Celular, Fundación Instituto Leloir, Buenos Aires, Argentina; 3 Departamento de Física, Universidad de Buenos Aires, Buenos Aires, Argentina; 4 Departament de Genètica and Institut de Biomedicina (IBUB), Universitat de Barcelona, Barcelona, Spain; Univ of Texas MD Anderson Cancer Center, UNITED STATES

## Abstract

Wound healing is an essential homeostatic mechanism that maintains the epithelial barrier integrity after tissue damage. Although we know the overall steps in wound healing, many of the underlying molecular mechanisms remain unclear. Genetically amenable systems, such as wound healing in *Drosophila* imaginal discs, do not model all aspects of the repair process. However, they do allow the less understood aspects of the healing response to be explored, e.g., which signal(s) are responsible for initiating tissue remodeling? How is sealing of the epithelia achieved? Or, what inhibitory cues cancel the healing machinery upon completion? Answering these and other questions first requires the identification and functional analysis of wound specific genes. A variety of different microarray analyses of murine and humans have identified characteristic profiles of gene expression at the wound site, however, very few functional studies in healing regulation have been carried out. We developed an experimentally controlled method that is healing-permissive and that allows live imaging and biochemical analysis of cultured imaginal discs. We performed comparative genome-wide profiling between *Drosophila* imaginal cells actively involved in healing versus their non-engaged siblings. Sets of potential wound-specific genes were subsequently identified. Importantly, besides identifying and categorizing new genes, we functionally tested many of their gene products by genetic interference and overexpression in healing assays. This non-saturated analysis defines a relevant set of genes whose changes in expression level are functionally significant for proper tissue repair. Amongst these we identified the TCP1 chaperonin complex as a key regulator of the actin cytoskeleton essential for the wound healing response. There is promise that our newly identified wound-healing genes will guide future work in the more complex mammalian wound healing response.

## Author Summary

Two major challenges in our understanding of epithelial repair and regeneration is the identification of the signals triggered after injury and the characterization of mechanisms initiated during tissue repair. From a clinical perspective, a key question that remains unanswered is “Why do some wounds fail to heal?” Considering the low genetic redundancy of *Drosophila* and its high degree of conservation of fundamental functions, the analysis of wound closure in imaginal discs, whose features are comparable to other post-injury events, seems to be a good model. To proceed to genomic studies, we developed a healing-permissive *in vitro* culture system for discs. Employing this method and microarray analysis, we aimed to identify relevant genes that are involved in healing. We compared cells that were actively involved in healing to those not involved, and identified a set of upregulated or downregulated genes. They were annotated, clustered by expression profiles, chromosomal locations, and presumptive functions. Most importantly, we functionally tested them in a healing assay. This led to the selection of a group of genes whose changes in expression level and functionality are significant for proper tissue repair. Data obtained from these analyses must facilitate the targeting of these genes in gene therapy or pharmacological studies in mammals.

## Introduction

Damage to an organism initiates a cascade of events that includes inflammation and the formation and remodeling of new tissue. Multiple studies have revealed significant similarities between how tissues are rebuilt during repair episodes and how they are built during development [1]. Thus, when considering epithelial repair, clear parallels exist at the structural level, as well as in signaling and the control of gene expression with the embryonic dorsal closure or the fusion of imaginal discs in *Drosophila*, ventral enclosure in *C. elegans* or eyelid closure in vertebrates [1–5]. Remarkably, co-assembly of actin cables and filopodial protrusions are instrumental in all these processes, with the majority being dependent on signaling by the JNK cascade [3, 6–9].

In invertebrates, the immediate wound healing response involves the formation of a temporary plug that encapsulate invading microbes, along with the activation of melanization and cross-linking enzymes [10]. In *Drosophila* larvae the outer part of the plug forms a scab within the first few hours of being wounded. The surrounding epidermal cells then orient themselves towards the wound and spread to reestablish a continuous epithelium. In larval epithelia wounds, the inactivation of the JNK pathway inhibits epidermal spreading and reepithelialization [11]. Remarkably, healing of incisional wounds in *Drosophila* adults also proceeds through a JNK dependent, lamellipodial-directed, epidermal cell spreading and scab formation [12]. In contrast, and similar to vertebrate early embryos, *Drosophila* embryos wounded by laser beams accumulate an actomyosin cable at the leading edge and display dynamic filopodial protrusions. The same is observed when imaginal discs are subjected to mechanical or genetic injuries [3, 13, 14]. The actin cable and the filopodial protrusions appear to participate in efficient sealing. The cable pulls the wound margins like a purse-string and assembles in response to different signaling events, including the activity of the JNK pathway [3, 13].

While we begin to understand some of the mechanisms of wound healing, many aspects remain unanswered. Genetically amenable systems, such as the healing of *Drosophila* imaginal discs, do not model all aspects of the repair process, such as inflammation or connective tissue contraction and fibrosis. However, they do allow the less understood aspects of the healing response to be explored; e.g. which cues regulate the migratory and proliferative machinery of reepithelialization? Which signal(s) and transduction pathways are responsible for initiating actin remodeling at the leading edge? How is sealing of the epithelia achieved? What inhibitory cues cancel the healing machinery upon completion? Answering these and other questions first requires the systematic identification and functional analysis of wound specific genes.

Examination of gene expression patterns using whole-genome techniques has been instrumental in the identification of many genes participating in specific developmental processes with potential relevance to disease pathogenesis [15]. To define the transcriptome program and identify pathways and genes critical to wound healing we have carried out genome-wide expression profiling and functional screens for potential healing regulators in *Drosophila* imaginal discs.

Imaginal discs are epithelial sacs composed by several thousand cells that, upon metamorphosis, evert and differentiate to form the external structures of the adult [16]. Imaginal discs are composed of two opposing layers of epithelial cells; a highly columnar epithelium (CE) that develops into adult structures during metamorphosis, and a layer of flat, squamous cells (peripodial epithelium—PE), which has a leading role in disc eversion and fusion [17, 18]. Shortly after puparium formation, imaginal discs evaginate and assume their positions at the surface of the prepupal animal. About 6 hours after pupariation, the proximal disc regions detach from their connection with the larval epidermis and expand, gradually replacing it. Contralateral and ipsilateral discs adjoin each other and finally meet and fuse. Spreading is driven by filopodia and actin bridges, which mediate the stretching of the imaginal epithelia [7]. This process, like embryonic dorsal closure and the healing of wounds either in embryos, larval and adult epidermis, or imaginal discs, depends on JNK activity and involves the specification of leading edge cells, which express *puckered* [7]. Wing imaginal discs mutant for the JNK pathway do not spread, or fail to reach the midline and seal the imaginal thorax. Similar abnormalities are observed after overexpressing Puc or dominant-negative JNK transgenes [19].

Injured imaginal discs heal and regenerate after implantation in adult abdomens, or in culture in growth permissive conditions [20, 21]. After cutting, CE and PE cells at the wound edges initiate healing through formation of a supracellular actin cable and emission of filopodial protrusions. Both epithelia expand and the wound edges zip and match to fulfill healing. Subsequently, discs engage in regenerative processes. The activation of the JNK pathway is observed in several rows of cells at the edge of discs wounds and is instrumental in the process promoting actin cable and filopodia formation [13]. These different steps are fully recapitulated in the repair of imaginal tissues after genetic ablation [14].

We have performed a comparative genome-wide profiling of imaginal cells actively involved in healing (JNK-activity positive) vs their non-engaged siblings. In these analyses, we identified a set of potential wound specific genes and defined specific co-expressed gene families participating in the healing process by Gene Ontology (GO), comparisons with published transcriptomic data, and clustering analysis. Importantly, besides identifying potentially relevant genes, we tested the function of many of their gene products by genetic interference and overexpression in two different processes; the fusion of imaginal discs during metamorphosis and wound healing in cultured discs. The fusion of imaginal discs was chosen as a functionally related proxi to discs healing. These non-saturated analyses identified a set of genes whose change in expression level and functionality was significant for proper tissue repair. Amongst those genes identified in the analysis, we explored in detail the role of individual subunits of the TCP1 chaperonin complex. Chaperones recognize proteins in non-native conformations and mediate the acquisition of the native structure [22]. One such chaperone complex is the TCP1 complex, which is known to mediate the folding of actin and tubulins [23]. We found that interference with TCP1 function affects actin cytoskeleton dynamics leading to early healing failure in imaginal discs. This indicates that the controlled expression of TCP1 subunits constitutes a new regulatory element essential to facilitate protein (actin) folding and wound healing.

Studies of the processes leading to wound closure should allow us to elucidate some of the general mechanisms that regulate natural cell movements during embryogenesis and late development. The factors identified in this study may thus be instrumental in the control of morphogenetic epithelial plasticity.

## Results

To investigate *Drosophila* genes that are specifically involved in healing, wounded imaginal discs were cultured *in vitro*, and one-channel microarrays used to compare the gene expression profiles of healing-engaged cells (those showing activation of the JNK signaling cascade) with cells not participating in healing (silent JNK activity).

### Healing of incised wing discs in culture

**Fig 1:**
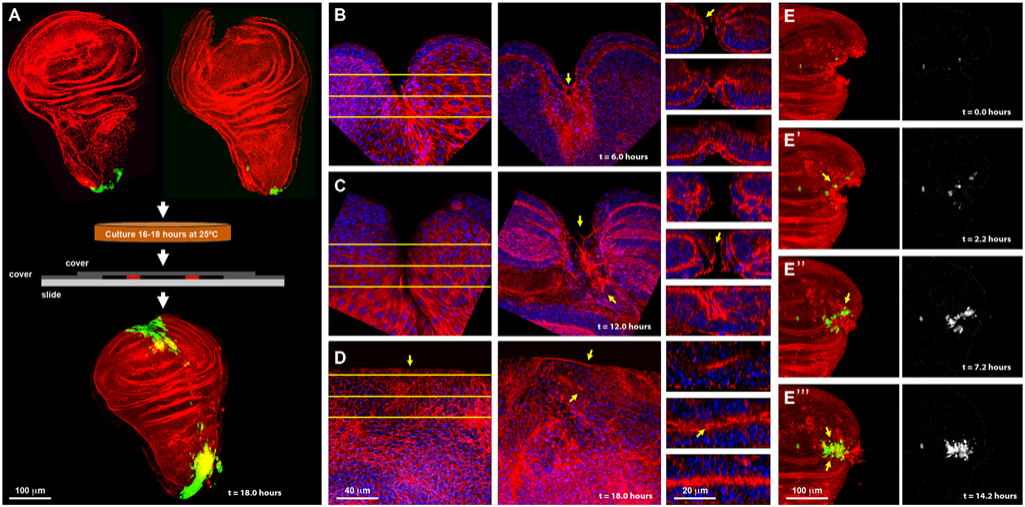
Imaginal discs wound healing *in vitro*. **A)** On the top row are shown dissected wing imaginal discs before injury (left) and just after injury (right) displaying *puc* expression at the stalk and some PE cells. Imaginal discs were cultured in a modified MM3 medium up to 24 hours on chambered slides as shown (center), which prevents discs folding and allows their imaging *in vivo*. At the bottom, a healed imaginal disc after 18 hours of culture displays strong ectopic *puc* expression at the wound edge and surrounding areas. Double staining for *puc* (green; *puc^E69^*-A—Gal4; UAS-GFP) and Actin (Phalloidin—red). **B)** Injured disc after 6 hours of *in vitro* culture. PE view (left) displaying a wide wound gap (yellow lines indicate the positions chosen for Z reconstruction). CE view (middle) of the same disc, showing elongated cells at the leading edge, filopodia and the initial zippering (arrow) of the epithelia. Orthogonal sections at three different levels (right) with the CE facing upwards and the PE downwards. The CE becomes partially disorganized establishing strong heterotypic contacts with the PE (arrow). **C)** Injured disc after 12 hours of *in vitro* culture. PE, CE and orthogonal views are shown as in B. There is a remarkable reduction in wound size and a notable actin accumulation (arrows). **D)** Injured imaginal disc after 18 hours of *in vitro* culture. Complete wound closure is observed for both, the PE (left), and CE (middle). Orthogonal sections show the basolateral zippering of the columnar epithelia (right). For **B** to **D**, phalloidin (actin) is shown in red; DAPI (nuclei) in blue. **E, E', E''** and **E''')** Time-lapse snapshots from S1 Movie of the healing process of a wounded imaginal disc cultured in modified MM3 medium. As culture progresses, *puc* expression (arrows) builds up at the edges on those cells actively engaged in healing. Cell membranes are shown in red (FM4–64) and *puc* expression in green (*puc^E69^*-A—Gal4; UAS-GFP) (left). The green channel is shown on the right. Scale bars are indicated for each panel.

Firstly, we developed an assay to culture and image wing imaginal discs isolated from synchronized late third instar larvae *in vitro*. This assay employed a modified medium, which we found to be healing-permissive (see Materials and Methods). We uncovered that the healing of incised wing discs fully resembled that of wounded discs cultured in adult fly abdomens *in vivo* (Fig. 1A).

Specifically, healing initiation could be morphologically observed after 6 hours in culture. Both disc epithelia (CE and PE) curled towards each other, reducing the wound surface, and establishing heterotypic contacts. This contractile curling seemed to be carried out by microfilaments underlying the CE [24]. Next, the CE initiated wound ‘zippering’ by emitting filopodial extensions from both, the basal and apical surfaces. At this time, actin accumulation was observed at the edges of the wound, initially in the PE. This actin ‘cable’ persisted in healing discs as long as 18 hours. Lastly, the CE cells facing the wound vertex changed their shape elongating towards the wound edge (Fig. 1B). After 12 hours of culture the elongated PE cells initiated wound closure, leading to a remarkable reduction in the wound surface. By this time, homotypic contact had formed within the CE and PE, and the CE completed basolateral zippering (Fig. 1C). Wound closure was completed when the CE and PE met and fused after 18 hours. Afterwards, both, the CE and PE underwent tissue relaxation, leaving no scar behind (Fig. 1D and S1 Movie). All these steps precisely resemble the healing of imaginal discs *in vivo* [13, 24], although with a slightly slower kinetics (18 instead of 12 hours for completion). A mass of cell debris was frequently seen attached to the wound vertex. However, this was extruded upon edges apposition.

*In vitro* cultures allow the epithelial sealing process to occur without any influence or assistance from circulating blood cells (haemocytes) and potential inflammatory responses. It also permits the isolation of sibling healing-engaged and healing-silent cells.

Using transgenic flies expressing GFP under the indirect control (Gal4-UAS system) of a specific JNK-responsive *puc* enhancer (see Materials and Methods), we found that *puc* is expressed in the disc stalk and at low levels in PE cells in non-wounded discs cultured *in vitro*. We also found that the JNK signaling becomes activated in cells participating in healing. Four hours after wounding, GFP expression was initiated both, in CE and PE cells along the epithelial wound edges. By 16 hours, high levels of GFP had expanded laterally from the leading edge and could be observed in all cells actively engaged in healing at the time of sealing completion (Fig. 1E). This observed dynamics of *puc* expression (S1 Movie) supports the previously described correlation between healing and JNK activity.

### Expression profiles of wild type and wounded wing imaginal discs

In order to quantitatively isolate *puc* expressing and non-expressing cells, we cultured wounded and non-wounded discs isolated from synchronized late third instar larvae for a length of time that maximized GFP expression and healing response. To isolate distinct viable cell populations expressing GFP, the discs were then subjected to dissociation and cell sorting by FACS (S1 Fig.).

The genes involved in healing were identified from whole genome expression profiles after four different populations were sampled: (1) JNK-active cells from wild type wing discs (**JNK^+^**); (2) JNK-silent cells from wild type wing discs (**JNK^-^**); (3) JNK-active cells from wounded wing discs (**JNK^+^ W**); and (4) JNK-silent cells from wounded wing discs (**JNK^-^ W**). Probes prepared from these samples were hybridized to microarrays and their median expression ratios were compared. Using stringent filter settings (see Materials and Methods) different sets of transcripts were identified.

**Fig 2:**
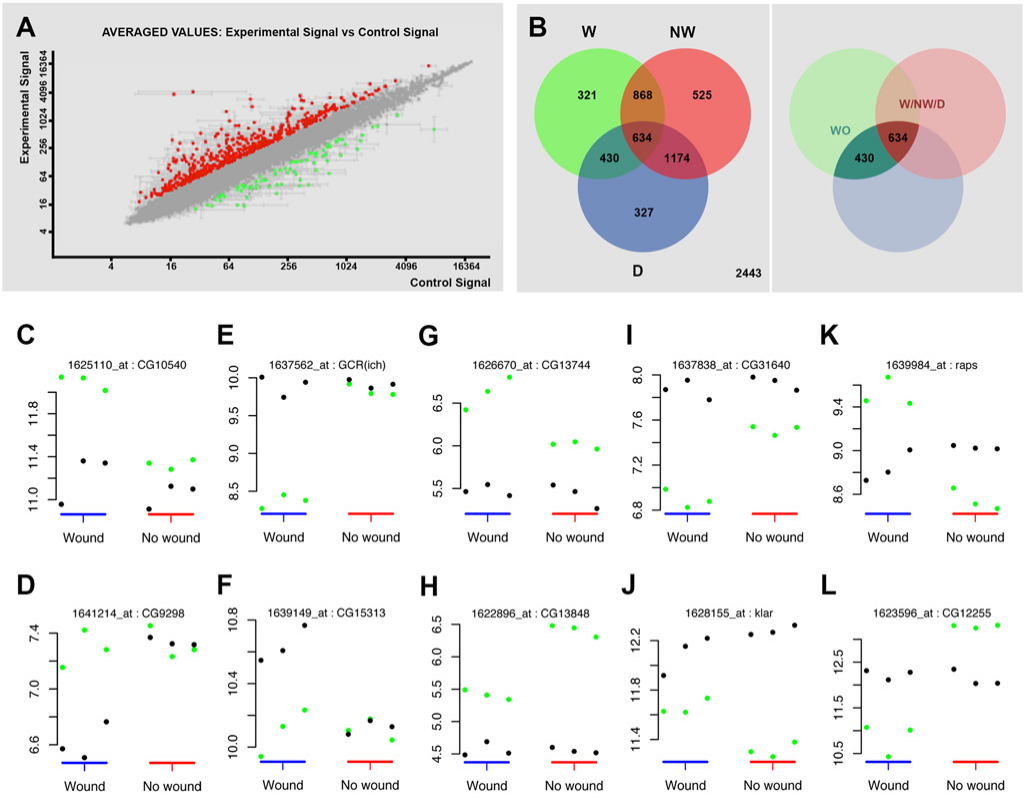
Transcriptomic healing response in imaginal discs. **A)** log-log scatter plot of the averaged microarray data for the global comparison between JNK signaling positive healing-engaged cells and their siblings in wounded imaginal discs (733 upregulated (red) and 748 downregulated (green) genes—FC 1.5, p-value < 0.05). **B)** A Venn diagram was used to represent the interactions of the different identified populations. The **W** subpopulation corresponds to those 2253 genes whose expression is modified in wounded discs. The **NW** subpopulation corresponds to those 3201 genes whose expression is modified in non-wounded discs. The **D** subpopulation corresponds to those 2565 genes in wounded or non-wounded discs that appear differentially modified between JNK-positive and negative cells. The **WO** and **W/NW/D** intersections highlighted on the right covers the 430 genes differentially expressed in wounded discs only and the 634 genes differentially expressed in both, wounded and in non-wounded discs but distinctly in both conditions. **C** to **L**) Representative genes for different subpopulations covering the **WO** and **W/NW/D** sets. Green spots show the level of expression for each replica in JNK-positive cells. Black spots show the levels of expression for each replica in JNK-negative cells. **C)** Genes upregulated in JNK-positive cells in wounded discs only (*CG10540*). **D)** Genes downregulated in JNK-negative cells in wounded discs only (*CG9298*). **E)** Genes downregulated in JNK-positive cells in wounded discs only [*GCR(ich)—CG5812*]. **F)** Genes upregulated in JNK-negative cells in wounded discs only (*CG15313*). **G)** Genes more upregulated in JNK-positive cells in wounded than in non-wounded discs (*CG13744*). **H)** Genes less upregulated in JNK-positive cells in wounded than in non-wounded discs (*CG13848*). **I)** Genes more downregulated in JNK-positive cells in wounded than in non-wounded discs (*CG31640*). **J)** Genes less downregulated in JNK-positive cells in wounded than in non-wounded discs (*klar—CG17046*). **K)** Genes upregulated in JNK-positive cells in wounded and downregulated in non-wounded discs (*raps—CG5692*). **L)** Genes downregulated in JNK-positive cells in wounded and upregulated in non-wounded discs (*CG12255*).

Changes in gene expression between JNK-positive and negative cells were analyzed for both, wounded discs and controls (see Materials and Methods). Firstly, we performed a global comparison in wounded discs (2-fold change, p-value < 0.05) of healing-competent (**JNK^+^ W**) cells vs. their non-engaged siblings (**JNK^-^ W**). This rendered 294 upregulated and 180 downregulated genes. When more relaxed conditions were applied (1.5-fold change, p-value < 0.05), additional sets, comprising 439 upregulated and 568 downregulated genes, were identified (Fig. 2A and S1 Table).

Secondly, we analyzed statistically significant variations between the changes observed in both experimental conditions (wounded vs. controls). Genes whose changes were found to be significant (1.3-fold change, FDR adjusted p-value < 0.05) in any comparison were kept for analysis. Using this method, three subpopulations were identified: 1) **W**, genes whose expression is modified in wounded discs (**JNK^+^ W** vs. **JNK^-^ W**); 2) **NW**, genes whose expression is modified in non-wounded discs (**JNK^+^** vs. **JNK^-^**) and 3) **D**, genes that appear differentially modified between JNK-positive and negative cells in wounded vs. unwounded discs. Combining these subpopulations, seven gene sets could be defined according to the significance pattern displayed by the analyzed comparisons (Fig. 2B). In this way, it was possible to identify the genes differentially expressed in wounded discs only (**WO**—genes present in the **W** and **D** but not in the **NW** subpopulations) or the genes differentially expressed in both, wounded and non-wounded discs, but distinctively in each condition (**W/NW/D**—genes present in the **W**, **NW** and **D** subpopulations).

The **WO** subpopulation comprised 430 genes (293 upregulated and 137 downregulated) that were differentially expressed between **JNK^+^** and **JNK^-^** cells in wounded discs only (S2 Table). On the other hand, the **W/NW/D** subpopulation was composed of 610 genes that were differentially expressed between JNK-positive and negative cells both, in wounded (392 upregulated and 218 downregulated) and in non-wounded (425 upregulated and 185 downregulated) discs. In this case the respective changes were significantly different between the two conditions (e.g. *CG1225*, upregulated in **JNK^+^ W** cells in wounded discs, but downregulated in **JNK^+^** cells in non-wounded controls) (S3 Table).

**Fig 3:**
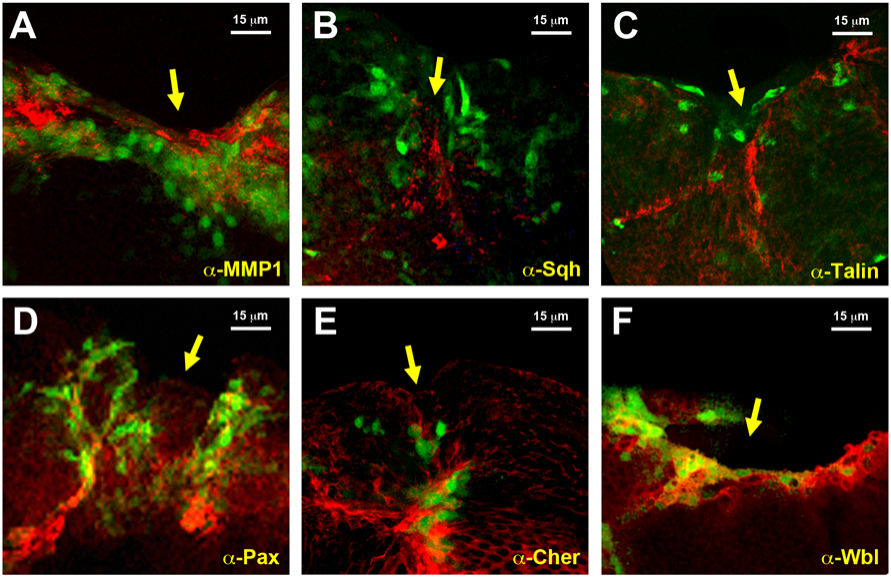
Validation of the genomic expression analysis. **A)** Imaginal disc, 12 hours after incisive wounding, showing enhanced expression of MMP1, a secreted matrix metalloproteinase upregulated in the healing transcriptome, in those cells engaged in healing (red). Similar results were observed for **B)** Spaghetti squash, the homologue of the regulatory light chain of non-muscle myosin II; **C)** Talin, a FERM domain protein linking the cytoplasmic tail of integrins to the actin cytoskeleton; **D)** Paxillin, a cytoskeleton scaffolding protein present at focal adhesions; **E)** Cheerio coding for filamin, an actin binding protein; and **F)** Windbeutel, a resident protein of the endoplasmic reticulum. All these genes were found to be upregulated in the healing transcriptome. GFP expression highlighting *puc* upregulation in all cases is shown in green. Scale bars are indicated for each panel.

**Fig 4:**
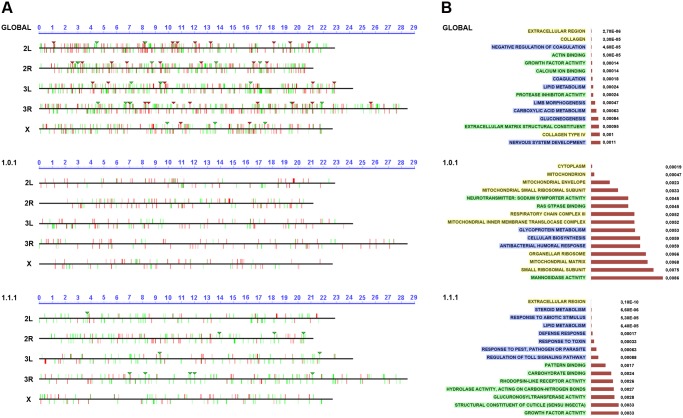
Chromosomal clustering and GO enrichment analysis. **A)** Graphical representation of the chromosomal clustering of the global comparison (JNK-positive vs negative cells in wounded discs) (upper panel), **WO** (middle panel) and **W/NW/D** (lower panel) contrasts. Upregulated (red bars) and downregulated (green bars) genes are positioned in the different *Drosophila* chromosome arms (X, 2L, 2R, 3L and 3R). Identified clusters are represented by inverted triangles. **B)** GO term enrichment analysis. The highest enriched GO terms (15) with lowest p-values (horizontal histograms) are displayed. These terms are distributed between the different terms categories: Cellular Component (**CCs**—yellow), Biological Process (**BPs**—blue) and Molecular Function (**MFs**—green) and represented for the global (upper panel), **WO** (middle panel) and **W/NW/D** (lower panel) contrasts.

**Fig 5:**
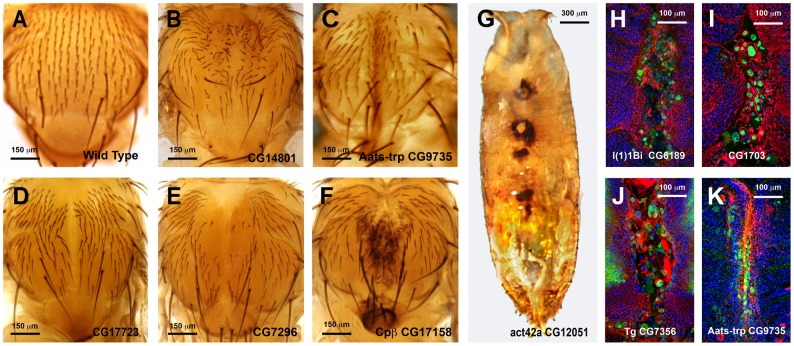
Thorax fusion phenotypes. **A** to **G)** Thorax adult phenotypes grouped in different categories resulting from RNAi interference in the expression of selected genes. UAS-RNAi transgenes were expressed in the presumptive central area of the thorax with a Pnr-Gal4 driver. **A)** Wild Type adult. It shows a canonical midline fusion of the thoracic hemisegments and perfect patterning of sensory bristles **B)** Interference with *CG14801* expression (Class **1TC** phenotype) results in mirrored bristle polarity defects in the adult thorax. **C)** Interference with *Aats-trp* (*CG9735*) expression (Class **2TC** phenotype) results in an excess of fusion with the hemithoraces collapsing at the midline. **D)** Interference with *CG17723* expression (Class **3TC** phenotype) results in a weak thorax cleft. **E)** Interference with *CG7296* expression (Class **4TC** phenotype) results in an intermediate thorax cleft. Some bristle defects are also observed. **F)** Interference with *cpβ* expression (Class **5TC** phenotype) results in a big thorax cleft and scutellum abnormalities. **G)** Interference with *Act42a* (*CG12051*) expression (Class **6TC** phenotype) results in pupal lethality and failure of discs eversion and/or fusion. Necrotic areas are found along the dorsal midline of an uneverted pupae. **H** to **K**) Expansion and fusion of imaginal discs are driven by leading edge cells, which extend long filopodia and accumulate an actin cable at the front edge. The JNK signaling pathway is activated in the leading edge cells during the fusion process. 7–7.5 h APF thoraces stained with Phalloidin (actin—red), DAPI (nuclei—blue) and Anti-LacZ (*puc* expression—Green). are shown. Anterior is up. **H)** Dorsal view of the thoracic area of a pupa which has failed to properly close upon downregulation of *l(1)1Bi* (*CG6189*) expression (Class **5TC**). Phalloidin staining shows loss of actin accumulation or filopodia extensions in the leading edge of the discs, while *puc*-expressing cells are present along the full edge of both hemithoraces. **I)** Dorsal view of the thoracic area of a pupa showing irregular closure at the midline upon downregulation of *CG1703* expression (Class **5TC**). Phalloidin staining shows fusion defects, enlarged leading edge cells that seem to detach, which sustain strong *puc* expression. Picnotic nuclei are present in the scutellum area. **J)** Dorsal view of the thoracic area of a pupa which has failed to fully close upon downregulation of *tg* (*CG7356*) (Class **4TC**). Phalloidin staining points to a clumsy accumulation of actin, most evident anteriorly. Several cells at the leading edge detach and round up and *puc* expression is sustained at the thorax midline. **K)** *Aats-trp* downregulation during thorax closure results in an excess of fusion (Class **2TC**) that does not seem to affect actin accumulation or *puc* expression (dorsal thoracic area). Scale bars are indicated for each panel.

In our analysis, we compared the absolute expression values for each gene for all experimental conditions evaluated. Thus, it was possible to determine if the overall variation in expression was due to changes in wounded discs relative to controls for individual genes; e.g. an apparent differential increase in the expression of a particular gene in **JNK^+^ W** cells in wounded discs could be a consequence of either the upregulation of the gene in this population or its specific downregulation in **JNK^-^ W** siblings (relative to its level of expression in non-wounded controls) (S2 and S3 Figs.). These comparisons allowed us to categorize the genes of the **WO** set into 4 different subsets: (1) genes that show upregulation in **JNK^+^ W** cells; e.g. *CG10540* (Fig. 2C); (2) genes that show downregulation in **JNK^-^ W** cells; e.g. *CG9298* (Fig. 2D); (3) genes that show downregulation in **JNK^+^ W** cells; e.g. *GCR (ich)* (Fig. 2E); and (4) genes that show upregulation in **JNK^-^ W** cells; e.g. *CG15313* (Fig. 2F) (S4 Table, see also S2 Fig.). The genes in the **W/NW/D** set were also categorized into different subsets. Six subsets depict the autonomous upregulation or downregulation of gene expression in **JNK^+^ W** cells in the absence of any change in **JNK^-^ W** cells: (1) genes that show higher upregulation in wounded discs than in non-wounded; e.g. *CG13744* (Fig. 2G); (2) genes that show higher upregulation in non-wounded discs than in wounded; e.g. *CG13848* (Fig. 2H); (3) genes that shows higher downregulation in wounded discs than in non-wounded; e.g. *CG31640* (Fig. 2I); (4) genes that show higher downregulation in non-wounded discs than in wounded discs; e.g. *Klar* (Fig. 2J); (5) genes upregulated in wounded discs but downregulated in non-wounded discs; e.g. *raps* (Fig. 2K); and (6) genes downregulated in wounded discs but upregulated in non-wounded discs; e.g. *CG12255* (Fig. 2L). Six equivalent subsets illustrate non-autonomous changes in gene expression in **JNK^-^ W** cells in the absence of any expression change in **JNK^+^ W** cells. Last, alternative combinations portraying different simultaneous autonomous and non-autonomous effects (up to 27 subsets in total) were also noted (S5 Table, see also S3 Fig.).

In order to group genes displaying the same pattern of expression changes throughout the four experimental conditions (**JNK^+^ W**, **JNK^-^ W**, **JNK^+^** and **JNK^-^**), which could be overlooked in the previous classifications, a further cluster analysis was performed. We used a template matching approach [25] to categorize absolute expression values [high (3), medium (2) and low (1)] for all genes expressed in each condition. These were used to generate different profiles [up to 81 potential combinations—from **JNK^+^ W 1, JNK^-^ W 1, JNK^+^ 1 JNK^-^ 1** (**1.1.1.1**) to **JNK^+^ W 3, JNK^-^ W 3, JNK^+^ 3 JNK^-^ 3** (**3.3.3.3**)]. In total, it was possible to allocate 288 genes (313 probes) to 34 different clusters (see Materials and Methods, S4 Fig. and S6 Table).

### Validation of genomic expression analysis

To validate the datasets obtained from the microarray analyses we compared the level of expression of selected genes. Protein expression was analyzed for selected genes by direct immunostaining of wounded discs during healing. MMP1, a secreted matrix metalloproteinase previously identified as essential for embryonic healing and regulated in response to JNK activity [26] (upregulated 7.43 times in GFP-positive cells) showed ubiquitous staining in the wing discs that was enhanced in those cells surrounding the healing edge (Fig. 3A). An overall but not full correlation between the domain of MMP1 expression enhancement and *puc*-GFP expressing cells was observed. These differences may result from variations in GFP expression levels (driven by the Puc-Gal4 driver) amongst healing-engaged cells. Similar results were observed for Spaghetti squash (Sqh), a homologue of the regulatory light chain of non-muscle myosin II, whose participation in wound repair has been previously documented [27] (upregulated 1.73 times in GFP-positive cells) (Fig. 3B); Talin [28], a FERM domain protein linking the cytoplasmic tail of integrins to the actin cytoskeleton (upregulated 1.65 times in GFP-positive cells) (Fig. 3C); Paxillin [29], a cytoskeleton scaffolding protein present at focal adhesions (upregulated 2.30 times in GFP-positive cells) (Fig. 3D); Cheerio [30] coding for filamin, an actin binding protein (upregulated 3.12 times in GFP-positive cells) (Fig. 3E) and Windbeutel [31], a resident protein of the endoplasmic reticulum (upregulated 2.98 times in GFP-positive cells) (Fig. 3F). Altogether, these data supported the accuracy of the transcriptome analysis (see Discussion).

### Cluster analysis

One potential cause of the co-expression of genes in different cell populations could be their common chromosomal location, which may lead to the hijacking of shared enhancers. To investigate the existence of latent healing-specific enhancers shared by co-expressed genes, we surveyed for the presence of gene clusters at specific chromosomal locations. We defined clusters as group of genes located on the same chromosome and in a close proximity to each other, but not necessarily adjacent to each other that showed the same expression behavior (upregulation or downregulation) during healing (see Materials and Methods and Fig. 4A). For the global comparison we identified 33 upregulated and 19 downregulated clusters (S7 and S8 Tables), while for the **W/NW/D** set we found no upregulated and 10 downregulated clusters (S9 Table). No clusters were identified for the **WO** set.

### Ontology analysis of healing genes

The differential expression of a gene in healing-engaged cells does not guarantee their significance as a regulator or structural element participating in healing. Their relevance will only be shown after characterizing their functionality. To prioritize “healing” genes for functional assays we performed a GO term enrichment analysis [32]. For the global comparison between **JNK^+^ W** and **JNK^-^ W** cells we identified 5 Cellular Component (CCs), 33 Biological Process (BPs) and 20 Molecular Function (MFs) enriched terms with a p-value < 0.01. In the **WO** subpopulation, we detected 17 CCs, 26 BPs and 22 MFs enriched terms with a p-value < 0.05; while 10 CCs, 78 BPs and 34 MFs were distinguished for **W/NW/D** (Fig. 4B and S10 to S12 Tables). These GO term enrichment analyses gave us a complex heterogeneous outline of the potentially relevant gene categories differentially expressed during healing.

### Functional analysis of “healing” genes

GO term enrichment was extremely heterogeneous and to select genes for functional analysis we did not take it in consideration. Instead, we utilized three specific criteria: (1) Robust changes of gene expression, (2) availability of functional tools, and (3) relevance of gene annotation (when known) in relation to healing in *Drosophila* or other models and/or to JNK signaling.

To investigate the potential role of selected genes, we interfered with the expression of upregulated genes, or restore, by overexpression, those that were downregulated. A search for suitable tools led to collect 309 UAS-RNAi lines potentially able to interfere with the expression of 202 upregulated genes (S13 Table). Furthermore, we collected 21 UAS lines designed to direct the expression of 15 downregulated genes (S14 Table). These two collections are obviously non-saturating and future expansion of this analysis should provide additional relevant information on the functionality of untested genes.

In an initial round, we analyzed the functionality of RNAi and overexpression lines in imaginal discs fusion (an amenable model resembling the healing process—see Introduction). We employed a Pnr-Gal4 driver and scored notum fusion phenotypes. Pnr-Gal4 is expressed in a broad domain in the presumptive notum region of the wing disc, reflecting the expression pattern of the *pannier* (*pnr*) gene [19]. In summary, 151 RNAi lines (48.86% of those tested) corresponding to 104 genes (51.48% of those tested) displayed distinguishable phenotypes ranging from mild notum suture imperfections to early lethality. It is important to note that these figures probably underscore the real number of potential positive regulators as many of the tested RNAi lines had not been analyzed before and they could eventually fail to abolish the expression of their presumed targets. 16 overexpression constructs (76% of those tested) corresponding to 11 genes (73% of those tested) also led to thorax fusion defects. The phenotypes obtained from this screen were grouped according to their defects [bristle polarity defects (**1TC**), excess of fusion (**2TC**), weak notum clefts (**3TC**), medium notum cleft (**4TC**), strong notum clefts (**5TC**), pupal lethal (**6TC**) and embryonic lethal (**7TC**)] (S15 Table; See Fig. 5A to 5G for representative phenotypes of RNAi and overexpression lines).

Pupal lethality or strong thorax phenotypes were, in some cases, further analyzed by monitoring the shape of leading cells, actin accumulation or *puc* (JNK activity) expression. For this purpose we crossed specific UAS-RNAi lines with Pnr-Gal4, *puc^E69^*-LacZ recombinant flies. Thoraces from pupae of 5 to 7 h after puparium formation (APF) were dissected and monitored for actin (Phalloidin) and *puc* expression (LacZ) (See Fig. 5H to 5K). Interestingly, neither a failure to complete thorax closure, nor an unrestrained fusion, necessarily results in the suppression of *puc* expression. In those cases presented, leading edge cells sustain high levels of JNK activity despite alterations to cell shape, actin accumulation or ectopic cell death. Whether these genes act as effectors of the JNK signaling pathway during healing, or participate in the healing process in a JNK-independent manner, remains to be determined.

**Fig 6:**
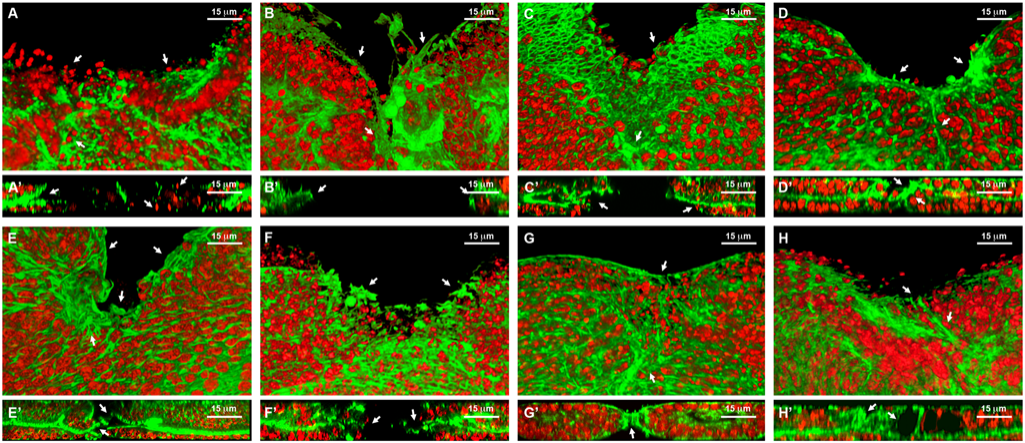
Healing phenotypes categories. 3D reconstruction of the peripodial and columnar epithelia at 18 hours after wounding. Phalloidin (actin) is shown in green; DAPI (nuclei) in red. **A)** Interference with *Act42a* expression (Class 1 phenotype) results in a large open wound and extensive cell death (arrows). **A’)** Orthogonal section. No heterotypic contacts, absence of actin cytoskeleton and occasional picnotic nuclei are depicted with arrows. **B)** Interference with *Aats-Val* expression (Class 2 phenotype) results in an extensive open wound with actin and active filopodia present at the leading edge (arrows). **B’)** Orthogonal section. Early approximation of the leading edges but failure on fusion. **C)** Interference with *pvr* expression (Class 3 phenotype) results in a large open wound. The CE has initiated healing but the PE fails to heal (arrows). **C’)** Orthogonal section. Arrows point to the delay on healing of the peripodial epithelia. **D)** Interference with *cpβ* expression (Class 4 phenotype) shows a large open wound and abnormal accumulation of actin (arrows). **D’)** Orthogonal section. Actin accumulation impedes the normal fusion of the epithelia (arrows). **E)** Interference with *scab* expression (Class 5 phenotype) shows a large open wound and a gap between the peripodial and the columnar epithelia (arrows). **E’)** Orthogonal section. The gaps at the edge between layers are more evident. The columnar epithelium initiates sealing apically (arrows). **F)** Overexpression of *mirror* (Class 5 phenotype) shows a partial closure and an abnormal distribution of actin (arrows). **F’)** Orthogonal section. Apical sealing precedes basal attachments that seem disorganized (arrows). **G)** Interference with *fimbrin* expression (Class 6 phenotype) displays advanced closure but disorganized actin cytoskeleton in the peripodial epithelia (arrows). **G’)** Orthogonal section. Actin accumulation at the junction is highlighted. The peripodial epithelia remains disorganized at the fusion point (arrows). **H)** Interference with *arc1* expression (Class 7 phenotype) displays a full closure but shape abnormalities at the junction and adjacent territories (arrows). **H’)** Orthogonal section. Apical gaps are observed at the fusion area and surrounding. Tissue layers are shifted (arrows). Scale bars are indicated for each panel.

**Fig 7:**
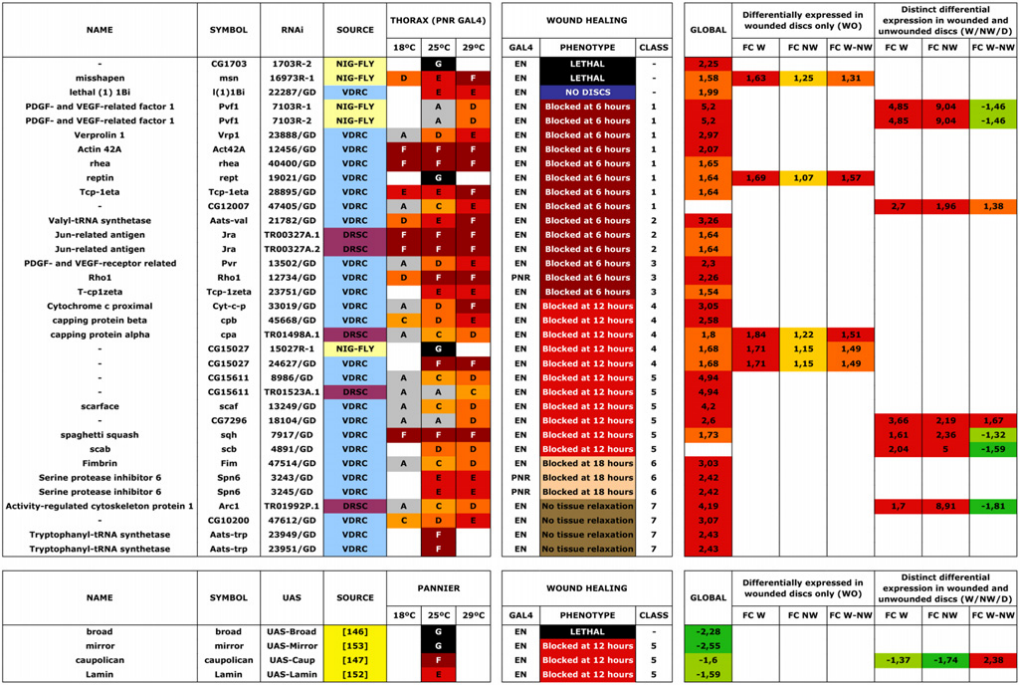
Wound healing phenotypes. 53 RNAi and 4 UAS lines were employed for interference or overexpression in the Pnr- or En-Gal4 domains. 35 and 4 lines respectively yielded distinguishable phenotypes. The thorax phenotypes (**PANNIER GAL4**) at different temperatures (**18°C, 25°C** and **29°C**) are indicated (**A** -Wild Type; **B**—Bristle Defects; **C**—Weak Thorax Cleft; **D**—Thorax Cleft; **E**—Strong Thorax Cleft; **F**—Pupal Lethal and **G**—Embryonic lethal). Healing was assayed at 25°C with different **GAL4** (**EN** or **PNR**). Healing phenotype is indicated and phenotypic classes are coded as in the text (**1** -Early (6 hours) defects—No Actin; **2**—Early (6 hours) defects—Unstructured Actin; **3**—Early (6 hours) defects—Actin present; **4**—Intermediate (12 hours) defects—CE zippering fails; **5**—Intermediate (12 hours) defects—Gaps between the epithelia; **6**—Late (18 hours) defects—Incomplete closure and **7**—No tissue Relaxation—Tissue folds) (see Functional analysis of “healing” genes section). Transcriptomic results are presented as described in S1, S2 and S3 **Tables** in progressive color series by fold change, p-value < 0.05. **GLOBAL**—global comparison of healing-competent JNK-positive cells vs their non-engaged siblings in wounded discs; **WO**—Genes differentially expressed in wounded discs only [**FC W**—comparison of JNK-positive and negative cells in wounded discs (**W**)—**FC NW**—comparison of JNK-positive and negative cells in non-wounded discs (**NW**)—**FC W-NW**—ratio of expression differences for JNK-positive vs negative cells between wounded and non-wounded discs (**D**)]; **W/NW/D**—Distinct differential expression in wounded and unwounded discs [**FC W**—comparison of JNK-positive and negative cells in wounded discs (**W**)—**FC NW**—comparison of JNK-positive and negative cells in non-wounded discs (**NW**)—**FC W-NW**—ratio of expression differences for JNK-positive vs negative cells between wounded and non-wounded discs (**D**)].

A second screen was performed for a collection of genes rendering strong defects in thorax closure. The criteria used to select these genes were their significant upregulation in the microarrays, the magnitude of their defects in thorax fusion, and considerations from GO data about their potential as healing regulators. 53 UAS RNAi lines corresponding to 44 genes and 4 UAS-overexpressing lines for 4 genes were tested (S15 Table). Each UAS line was screened using two different Gal4 lines: Pnr-Gal4 or En-Gal4. For Pnr-Gal4, the expression of several UAS transgenes led to lethality, which prevented us from performing the healing assays. En-Gal4 is a posterior compartment specific Gal4 driver that reflects the expression pattern of the *engrailed* (*en*) gene [33]. The phenotypes induced by transgenes expressed under this line are confined to the posterior region of the disc. The anterior compartment can therefore be used as internal control. All crosses were set at 25°C and third instar larval imaginal discs were dissected, wounded in a specific region depending on which Gal4 line was used (in most cases employing the En-Gal4 driver) and cultured in modified medium for 20–24 hours. At least 5 discs were screened for healing defects for each genotype after performing incisive cuts ≈ 30 μm long. The data obtained were extremely robust and almost no variation was observed between individual samples. RNA interference for 35 UAS RNAi lines corresponding to 29 genes (66,0 and 65,9% respectively of those tested) showed healing defects, while all 4 genes tested by overexpression (100%) produced a unique phenotype. These phenotypes (both, for interfered and overexpressed genes) were grouped according to the timing of healing arrest and the qualitative evaluation of defects in cell contacts and actin cytoskeleton organization (see Figs. 6 and 7 and S5 Fig.).

**Class 1.** Interference with the expression of genes belonging to this group (*act42a-CG12051* (Fig. 6A), *verprolin (vrp)-CG13503*, *pvf1-CG7103*, *reptin-CG9750*, *CG12007*, *rhea/*Talin-*CG6831*, *TCP1η-CG8351* and *TCP1ζ-CG8231*) completely prevented healing initiation. The wounded tissue showed no signs of filopodia formation at the leading edge. Extensive apoptotic nuclei could also be observed (See S2 Movie for *TCP1ζ*).

**Class 2.** Interference with the expression of *jra-Jun related antigen-CG2275* or *aats-val-CG4062* (Fig. 6B) caused the healing process to pause as early as 6 hours after wounding. The leading edge of the wounded tissue showed unstructured actin accumulation and no filopodia formation. The cells of the CE align along the edge rounding at the vertex and showed distinct shape changes. Heterotypic contacts were not achieved.

**Class 3.** Imaginal discs in which the expression of *rho1-CG8416* and *PDGF- and VEGF-receptor related-CG8222* (Fig. 6C) is abolished, completed most of the initial steps in healing (within 6 hours), including actin accumulation, heterotypic contacts between two membranes and initiation of zippering of the CE. However, they showed no vertex cells rounding, peripodial sealing or obvious filopodia at the leading edge (they are essentially defective in homotypic contact formation).

**Class 4.** Healing assays on discs defective in *capping protein α-CG10540*, *capping protein β-CG17158* (Fig. 6D), *CG15027* and *cytC-p-CG17903* yielded incomplete closure and no vertex cells rounding, although heterotypic contacts were present and partial PE sealing was achieved. In the CE, filopodia do not form properly, a great accumulation of actin can be observed and zippering was affected.

**Class 5.** Imaginal discs in which the expression of *CG7296*, *scab-CG8095* (Fig. 6E), *scarface-CG11066*, *CG15611* or *sqh-CG3595* is abolished, or the expression of *mirror-CG1943* (Fig. 6F), *caup-CG10605* and *lamin-CG6944* is increased by overexpression, yielded incomplete healing. Here, the healing process halts after 12 hours of culture, causing both, CE and PE epithelia to proceed up until the closure step, before discontinuing. Gaps are found between the epithelia. Both, the PE and CE established homotypic contacts but they failed to complete closure.

**Class 6.** Downregulation of *fimbrin-CG8649* (Fig. 6G) by UAS-RNAi in healing assays yielded incomplete closure after 18 hours. The PE, and to a greater extent the CE, fail to tightly join. The tissue remains loose and disorganized with frequent apical gaps.

**Class 7.** Healing assays for *arc1-CG12505* (Fig. 6H), *serpin55B-CG10913* (*spn 6*) or *CG10200* deficient imaginal discs yielded complete healing of both, CE and PE. However, the disc tissues failed to relax back to an untensed condition, leading to numerous constricted folds. This was most evident at the CE.

### The expression of TCP1 chaperonin complex subunits is a regulator of actin folding and wound healing in imaginal discs

**Fig 8:**
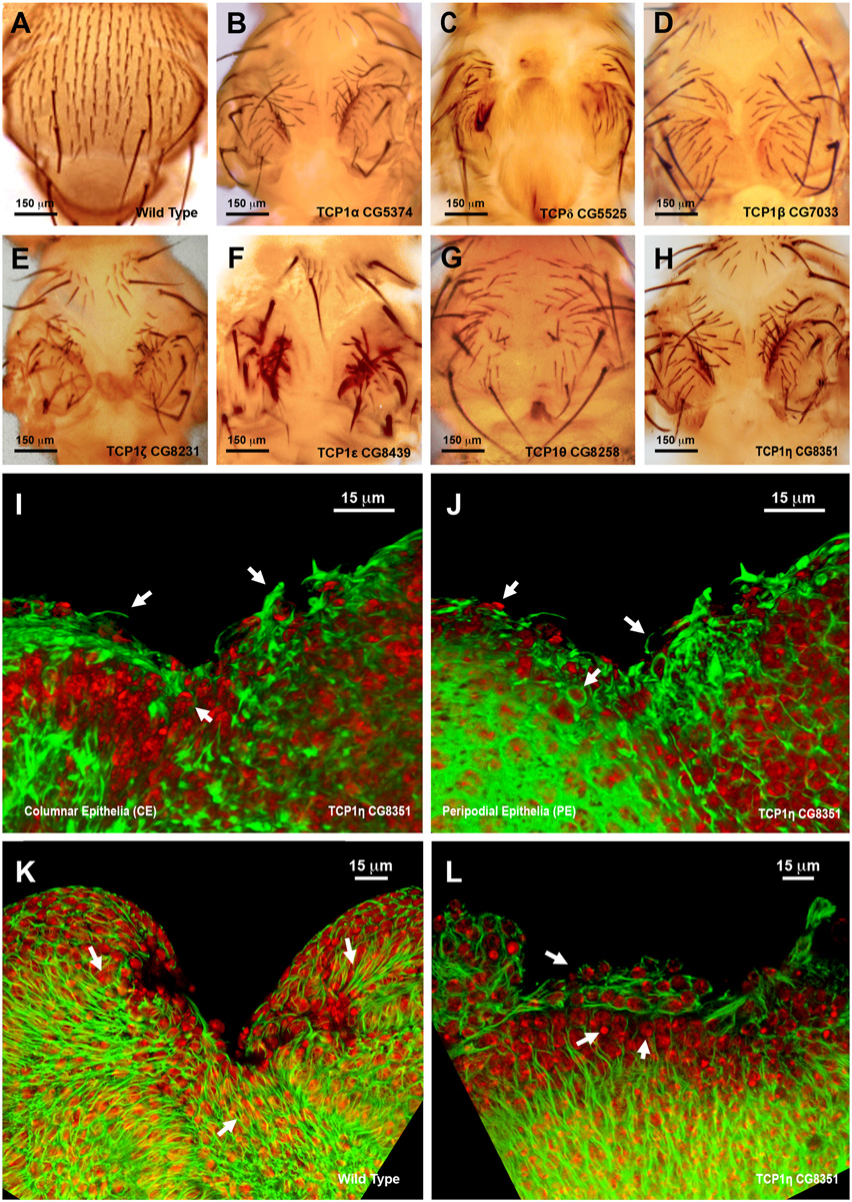
Thorax fusion and impaired healing phenotypes of TCP1 subunits. **A)** Wild type notum of an adult *Drosophila*. **B** to **H**) Thorax malformations observed after interference (RNAi) with the expression of different TCP1 subunits as labeled (tested with a Pnr-GAL4 driver). **I** and **J**) 3D reconstruction of the peripodial and columnar epithelia at 18 hours after wounding. (**I**) PE and (**J**) CE views. *TCP1η* RNAi knockdown results in impaired healing after 20–24 hours of culture *in vitro* (tested with a En-GAL4 driver). Arrows point to abnormal actin-rich structures and arrowheads to dead cells. Phalloidin (actin) is shown in green; DAPI (nuclei) in red. **K**) Injured wild type disc after 12 hours of *in vitro* culture. CE view showing the perpendicular alignment of microtubules to the sealing front (arrows) and elongated cells at the leading edge. A remarkable reduction in wound size is observed. **L**) Injured *TCP1η* RNAi knockdown disc after 12 hours of culture *in vitro*. CE view showing conspicuous picnotic nuclei (arrowheads) and an abnormal transverse alignment of microtubules (arrows) at the leading healing front. Wound closure fails to proceed. Tubulin is shown in green; DAPI (nuclei) in red. Scale bars are indicated for each panel.

The strong phenotypes observed following interference with the TCP1 subunits *TCP1η* and *ζ* prompted us to further explore its role in tissue healing. TCP1 as a cytosolic chaperonin that promotes the folding of non-native actins and tubulins in eukaryotic cells *in vivo* [34]. *TCP1η* and *ζ* showed significant upregulation, 1.64- and 2.54-fold respectively, in direct comparisons in healing-engaged cells. Their downregulation by UAS-RNAi during thorax closure resulted in strong defects in imaginal disc fusion (see above). Importantly, strong thoracic clefts were also observed after knocking down every subunit of the complex, with the exception of TCP1*γ*, which resulted in embryonic lethality (Fig. 8A to 8H). This suggests that the fusion of the imaginal discs is dependent on a systemic function implemented by the TCP1 complex as a whole, i.e., the loss of anyone subunit will reduce the functionality of the complex in this process.

Healing assays with En-Gal4 and RNAi lines specific for the different TCP1 subunits (Fig. 8I and J and S6 Fig.) yielded an open wound phenotype after 20–24 hours of culture. Healing was blocked as early as 6 hours after wounding. This early phenotype was observed for all subunits except TCP1*γ*, whose inhibition led to embryonic lethality. Both, the CE and PE epithelia failed to close wounds. The wounded tissue showed disorganized actin accumulation while filopodia formation at the leading edge was abolished (Fig. 8I and 8J). On the other hand, microtubules, which in the wild-type condition align along the direction of healing progression (Fig. 8K) reposition themselves to transverse orientations on the epithelial leading cells of interfered discs (Fig. 8L). As for imaginal fusion, all subunits are apparently required for wound closure.

To analyze the potential role of *Drosophila* TCP1 on the regulation of actin dynamics, we performed RNAi mediated knockdowns of TCP1 subunits in *Drosophila* S2R+ cells. S2R+ cells were transiently transfected with subunit specific TCP1 dsRNAs and an actin reporter construct, pMT-Act-GFP, carrying an actin-GFP fusion under the control of a metal activated promoter (see Materials and Methods). Four days after transfection, cells were subjected to live imaging, monitoring actin dynamics. TCP1 dsRNA (*η* subunit) treated S2R+ cells changed their morphology, became rounded, and displayed no filopodial protrusions (S7 Fig.). Further, TCP1 appears to be necessary for the production and polymerization of actin. We analyzed TCP1 ability to influence actin filaments polymerization by employing Latrunculin A (LatA) [35] (see Materials and Methods). Both, control and TCP1 dsRNA (*η* subunit) transfected cells became fully rounded immediately after LatA treatment. Remarkably, after a recovery period of 48 hours in the absence of the drug, control cells restore their normal morphology and F-actin levels, while dsRNA treated cells remained rounded with no signs of actin polymerization recovery (S7 Fig.). Since imaginal fusion and wound healing are actin-dependent, and the actin cytoskeleton is deregulated following the knockdown of TCP1 subunits, it is reasonable to assume that the regulation of actin synthesis and folding by TCP1 is essential in *Drosophila* for proper epithelial tissue healing.

## Discussion

### Wing discs healing transcriptome

We have developed a new *in vitro* model of wound healing for wing imaginal discs that recapitulates most previously described discs healing stages. We employed this method as a mean to characterize the transcriptomic make up of healing discs.

The transcriptomic profile of healing tissues has been previously explored in *Drosophila*. Comparison of the RNA profiles of laser wounded vs non-wounded wild type and macrophage-deficient *serpent* (*srp*) embryos [36] led to the identification of wound-activated haemocyte genes important in the immune response to wounding. Other analyses were performed in regenerating wing discs manually fragmented and implanted into adult females [37]. This last approach allowed the identification of multiple genes expressed in the early and late stages of regeneration. Still, a lack of discrimination between healing engaged and passive cells reduced its informative capacity.

Based on this current knowledge, we aimed to identify the “healing transcriptome” of those cells activating JNK signaling upon wounding in imaginal discs. In injured imaginal discs, *puc* expression is detected after just a few hours suggesting that the activation of the JNK pathway is one of the earliest responses to wounding. JNK activity is required for discs healing and early regeneration and cell lineage studies show that *puc*-expressing cells contribute to the regenerated tissue [13, 38–41]. How the JNK pathway is activated during healing is not known. Neither, the primary responses triggered by the activation of JNK signaling following injury. We employed *puc^E69^*-Gal4 I/UAS-GFP transgenic animals, which express GFP under the control of *puc* enhancers to identify and isolate JNK active cells from wounded and control discs. These cells respond both, to JNK signaling agonists and antagonists, as well as to incisive and ablating wounding [13]. To distinguish the endogenous transcriptional response of JNK active cells [18] from that of the cells that activate JNK in response to wounding, we performed quadruplicated transcriptomic assays. Our analyses tackled the early/intermediate events of the healing process, which predominantly involve cell rearrangements and dynamic changes in cell adhesion and cytoskeleton activity. To single out the transcriptomic healing response, we performed a one to one global comparison between cells involved in healing and neighboring cells in wounded discs and dual comparisons aimed to identify genes with differential expression in wounded discs only, **WO** (genes specific to wound healing and not to disc eversion and fusion) and with distinct expression changes in wounded and non-wounded discs, **W/NW/D** (genes important for both, healing and fusion). To identify those cellular and molecular functions augmented amongst the genes whose expression changes during healing we performed GO term enrichment analyses.

### Ontology analysis of healing genes

When comparing our results to previously reported expression profiles of imaginal discs during regeneration [37], several similarities arise amongst enriched GO terms. Amongst them, the upregulation of genes involved in stress and immune response and regulation of transcription, or the downregulation of genes related to RNA processing. In contrast, the upregulation of genes involved in cytoskeleton organization, biogenesis and cell proliferation or the downregulation of helix-loop-helix transcription factors in JNK-positive cells in our analyses differs with the overrepresentation of these categories amongst downregulated and upregulated genes respectively during regeneration [37]. These differences are probably due to the fact that each study tackles a different paradigm. Our approach did not evaluate the regeneration response of whole discs [37] but focused on early/intermediate healing events.

Protein and cellular biosynthesis, mitochondrial activity, ATP synthesis, ribosome activity and chromatin remodeling GO terms are enriched amongst those genes whose expression is enhanced in JNK-positive cells in wounded discs only in the **WO** subpopulation. Cellular localization, secretory pathways, signaling and extracellular attachments are also overrepresented GO terms. In particular, a meaningful swift in the degradative properties of the tissue appears to be necessary to allow healing progression. We found that secreted MMP1 or Timp (Tissue inhibitor of metalloproteases) [26, 42] amongst other extracellular matrix proteins are specifically upregulated in JNK-positive cells upon wounding, while a different set of peptidases is overrepresented amongst downregulated genes.

Otherwise, we found that intracellular communication and regulation of cell motility and adhesion are GO terms enriched amongst the genes upregulated in JNK-positive cells in the **W/NW/D** subpopulation. In vertebrates, it has been extensively demonstrated the importance of secretory factors in the repair of wounded areas [43]. Also, in flies, multiple growth factors and cytokines, including PDGF, VEGF, TGFβ (Transforming Growth Factor β), FGF (Fibroblast Growth Factor) or Egr (Eiger) have been reported to be necessary for thorax fusion and healing [7, 43–45]. In particular, we found that *pvf1* and *pvf2*, which code for PDGF- and VEGF-related factors are strongly expressed in JNK-positive cells and, remarkably, also expressed at high levels in JNK-negative non-engaged cells during healing just in wounded discs. The meaning of this upregulation is unclear but it could point to a systemic coordination between the cells actively involved in repair and the surrounding ones to accommodate the tissue topology to the healing process. This possibility remains to be tested. On the other hand, organ development, cell metabolism regulation and structural constituents of cuticle GO terms are enriched amongst the downregulated genes in JNK-positive cells in both, wounded and non-wounded discs suggesting that the functions of these genes, e.g. cuticle deposition, may need to be cut short both, for imaginal fusion and proper tissue repair.

Last, some GO terms are overrepresented in both subpopulations, **WO** and **W/NW/D**, e.g. cytoskeleton, GTPase activity, JAK/STAT signaling, induction and regulation of cell death and defense and stress responses are enriched amongst upregulated genes in JNK-positive cells. The upregulation of GTPases [2, 46–50], the JAK/STAT cascade [51–53], pro-apoptotic genes [54, 55] and innate immunity genes might be connected to the activation or propagation of JNK activity as a physiological response to stress. GTPases function may be also associated to their known roles as cytoskeleton regulators and linked to the cytoskeletal rearrangements observed in the initial phases of both, healing and discs fusion.

### Functional analysis of “healing” genes

Different functional screens to identify genes involved in wound healing have been performed in *Drosophila* [56–58]. A forward genetic screen by insertional mutagenesis have led to the identification of 30 lethal mutants with defects in embryonic epithelia repair [56]. Further, in a screen of deficiencies and single mutations, several genes have been identified as regulators of the transcription of wound-responsive markers in response to puncture wounds in embryos [58]. Finally, UAS-RNAi transgenes have been overexpressed in larvae to identify genes involved in the control of epithelial migration during postembryonic wounds [57]. These screens are recent, and so their subsequent follow-up has been limited. However, when taken together, they provide solid evidences supporting the conservation of multiple aspects of the mammalian wound response in *Drosophila*.

To investigate the functionality of the genes identified in our transcriptomic analysis during tissue repair, we performed two reverse functional screens. First, we interfered with the natural process of thorax closure identifying at least 115 genes (approximately 53% of those tested) whose upregulation or downregulation resulted in closure defects. Considering, that many RNAi lines do not efficiently knock down gene expression, these results probably underestimate the real number of regulators in our transcriptome data. Several of those lines reproducibly yielding strong phenotypes were further tested in a disc-healing assay. We found that 33 (69%) of the 48 genes tested display wound healing defects.

The most significant set of relevant genes identified in the functional screenings includes factors involved in the activation or transduction of JNK signaling. Interference in the expression of *Jra—DJun* (as previously observed in embryo and larvae [56, 57]) and GTPase activity regulators such as *loco* or JAK/STAT elements (*upd*) [59, 60] display extreme defects in healing (Phenotypic Class 2). Interestingly, several potential regulators or mediators of JNK activity were also identified for the first time. An element of this class is *CG1703*, a member of the ABC-F subfamily of ABC proteins [61]. ABC-Fs are ATPases that regulate translation [62]. The downregulation of *CG1703* resulted in a phenotype (Class 5TC) strikingly similar to that observed upon downregulation of the JNK pathway [18]. It is tempting to speculate that *CG1703* may somehow regulate the activity of the JNK cascade at the level of translation.

Transcriptional activators such as *AP-2*, *mirror*, *ara*, or *caup* and repressors such as *Ssb-c31a* or *rept* constitute a second category of genes highly represented amongst those whose misregulation resulted in strong healing phenotypes. *mirror*, *ara* and *caup*, which code for helix-loop-helix proteins of the Iroquois complex [63, 64] are downregulated during healing. The overexpression in the notum of these genes results in embryonic (Class 7TC) for *ara* or *mirror* or pupal lethality (Class 6TC) for *caup*. Remarkably, the overexpression of *mirror* or *caup* in cultured discs resulted in early homotypic contacts between the PE and CE but a failure to proceed further (Phenotypic Class 5). The implication of this complex in wound healing is unexpected and its role remains to be explored.

Interference in genes involved in intercellular communication, such as cytokines and growth factors, which have been previously reported as mediators of healing in vertebrates [43–45], also resulted in discs healing defects. The inhibition of *pvf1* or *pvr1* resulted in notum malformations (Class 4TC and 5TC respectively) and caused incomplete healing with large wounds (Phenotypic Class 1 and 3 respectively). Pvf and Pvr are necessary in *Drosophila* for the apical assembly of the actin cytoskeleton during embryogenesis [65] and required for other diverse developmental processes [66–68]. Recently, Pvf1 and Pvr have also been shown to participate in larval wound healing [69].

Regulators of actin cytoskeleton dynamics including *Act42a*, *vrp*, *TCP1η* and *ζ* *cpα* and *β* and *fimbrin* are also enriched amongst those genes whose function is essential for healing. Downregulation of *Act42a*, *vrp* and *TCP1η* or *ζ* results in pupal lethality (Class 6TC) and yields an early open wound phenotype (Phenotypic Class 1). Vrp encodes a WH2-containing actin binding protein needed for actin polymerization during polarized growth [70]. Vertebrate Vrps regulate actin dynamics either by binding directly to actin or to proteins of the WASP family [71]. The TCP1 complex is a cytosolic chaperonin involved in the folding of non-native actins and tubulins, amongst other substrates [34]. Milder healing defects are observed upon interference in *cpα* and *β* and *fimbrin* expression. Cpα and Cpβ are regulators *of actin filament growth [72] that cap the barbed ends of filaments preventing the addition or* release of actin subunits [73]. In *Drosophila*, Cpα is required for the proper development of the wing blade primordium and its loss results in actin and myosin accumulation, cells extrusion and apoptosis [74]. On the other hand, loss of Cpβ affects border cell migration, reducing the length of actin protrusions. It also causes abnormal actin aggregation on nurse cell membranes [75]. Downregulation of *cpα* or *β* resulted in notum malformation (Class 4TC and 5TC respectively) and yielded incomplete healing affecting CE zippering (Phenotypic Class 4). Fimbrin is a cytoskeletal protein associated with microfilaments in microvilli, microspikes, membrane ruffles, and cell-substratum attachment sites [76]. It directs the formation of tightly bundled F-actin assemblies [77] and in *Drosophila* has been shown to participate in chromosome segregation during meiosis in females [78]. Downregulation of *fimbrin* results in notum malformation (Class 4TC) and incomplete healing after 18 hours (Phenotypic Class 6). Altogether, these data indicate that proper levels, folding and polymerization of actin appear to be essential for the earliest steps of healing, while the correct regulation of actin filaments growth or bundling seems to be essential for late healing completion.

### The TCP1 complex

TCP1 is a cytosolic chaperone complex of about 900 kDa ubiquitously expressed [79]. Each of its eight subunits is coded by individual highly divergent genes [80]. Although many of its substrates have yet to be identified, it is well established that it participates in the folding of actin and tubulin monomers. Clinically, TCP1 has been linked to colorectal cancer progression and to prevention of polyglutamate-containing proteins aggregation in neurodegenerative diseases like Huntington’s [81, 82].

We found that two subunits of the complex, *TCP1η* and *ζ*, are upregulated in healing-engaged cells. We also found that interfering in their expression led to strong defects in thorax fusion, and to an early healing arrest. Importantly, although the remainder of TCP1 subunits was not upregulated upon wounding, when their expression was impaired, defects in thorax closure and wound healing were also observed. Fusion and healing arrest in TCP1 compromised discs is accompanied by a failure to organize actin microfilaments and to generate filopodia at the edges. It is also associated with defects in the organization of the microtubular network. This phenotype is very similar to that observed after interfering with *Act42a* or *vrp* suggesting that one of the roles of the TCP1 complex during healing might be to fold actin monomers and thus promote the active and rapid rebuilding of the cytoskeleton. Providing a sufficient level of folding capacity by increasing the transcriptional expression of its subunits would synergize with the upregulation of actin and Vrp upon wounding. This mechanism constitutes a new way of improving actin dynamics, working in parallel to those implemented by conventional actin regulators during healing.

It has been shown that specific TCP1 subunits are involved in the recruitment of distinct substrates to the complex [83]. Our findings suggest, however, that, at least during healing, the structural integrity and systemic activity of the complex is essential for its function: i.e. all subunits must be expressed at sufficient levels to modulate the TCP1 folding activity and cannot be compensated by others. Tissue repair is not related to any docking specificity associated to the individual components of the complex. Actin and tubulin have been identified as bona fide general substrates of the TCP1 complex in yeast and vertebrates. Indirectly, this suggests that actin (and maybe tubulin) might be the main substrates targeted by TCP1 during thorax closure and wound healing. Most probably, the upregulation of the *TCP1η* and *ζ* subunits responds to the need to reach sufficient expression levels to cope with the exceptional requests imposed by the healing process. How healing-engaged cells sense the need to increase TCP1 subunits mRNA levels and how they do implement it are open questions.

TCP1 in *Drosophila* appears to share its major substrates, actin and tubulin, with other organisms [79]. We have substantiated this assertion by noticing that interfering with TCP1 expression in *Drosophila* S2R+ cells results in strong alterations of their morphology and the organization and dynamics of their actin cytoskeleton. Moreover, cells deficient for TCP1 are unable to recover their natural levels of polymerized actin upon a pulse of Latrunculin A. Interestingly, a persistent upregulation of *TCP1ζ* during fibrotic healing in adult cutaneous and mucosal wounds in rabbits has been reported [84] suggesting that the critical role we uncovered for TCP1 may be conserved between invertebrates and vertebrates.

In summary, in this study, we have uncovered a new set of genes functionally relevant for the control and implementation of healing in *Drosophila* imaginal discs. We identified 33 genes whose inhibition or overexpression results in defects on healing or its complete absence. Another set of 60 genes show defects in thorax closure and they could also assist in the healing response. In relation to the signals triggering tissue remodeling, we recovered some known (*msn*, *jra*, *pvf1*, *pvr*, *rho1* and *scarface*) and unknown elements (*CG12007* and *CG17003*) potentially linked to the JNK and Pvf/Pvr cascades. Their study will be instrumental for understanding the initiation of the healing response. With respects to the process of tissue expansion, we, as expected, found multiple regulators of the actin cytoskeleton (*act42a*, *vrp*, *sqh* and *cpα* and *β*) and matrix attachments components (*scab* and *rhea/*Talin) (some never associated to healing before). We also discovered a novel role for the TCP1 chaperonin (*TCP1η* and *ζ*) in the context of healing. We concluded that TCP1 provides a systemic function transcriptionally regulated during healing as a modulator of cytoskeleton dynamics. Further, we uncovered a role for the Iroquois complex of transcriptional regulators (*mirror*, *ara* and *capu*) as a negative factor preventing healing progression. In addition, a series of genes with established (*l(1)1Bi*, *AP-2*, *Ssb-c31a*, *rept*, *aats-val*, *cytC-p*, *broad* and *lamin*) or unidentified (*CG7296*, *CG15027* and *CG15611*) functions were documented as active elements on healing progression for the first time. Their functions in this context remain to be explored. Last, regarding the sealing of the epithelia, we identified five genes *fimbrin*, *aats-trp*, *arc1*, *serpin55B* (*spn 6*) and *CG10200* whose absence, without affecting healing itself, leads to a failure of final sealing or impedes the tissue relaxation. How do they work? We don’t know, but they constitute genetic entry points that could help us to understand how epithelial closure is achieved or regulated.

Considering the low genetic redundancy of *Drosophila*, and the high degree of conservation of fundamental cellular functions and signaling pathways when compared to vertebrates, data obtained from our analyses must facilitate investigating and targeting functionally relevant genes in vertebrate healing studies. A variety of different microarray analyses of murine and humans have already described gene expression profiles of cells located at wound sites [85, 86], however, very few studies have addressed the functional role of genes potentially involved in healing regulation. There is promise that our newly identified wound-healing genes will guide further work in the more complex process of mammalian tissue repair.

## Materials and Methods

### Culture *in vitro*, wounding, live imaging and immunostaining of imaginal discs

Third instar larvae wing imaginal discs were dissected and incubated in a MM3 medium (Shields and Sang´s) supplied with fly extract. The fly extract was prepared following the protocol suggested by the DGRC, Indiana University [87]. This modified media allows wing imaginal discs to be cultured for more than 24 hours.

Third instar larvae imaginal disc dissections were performed in cultured medium and incisions of precise sizes were carefully executed with a pair of tungsten needles. This was followed by static culture in supplemented MM3 media for 16–18 hours. We found that imaginal discs in culture lose their morphology after 5 to 6 hours, becoming spherical in shape. To flatten the samples we mounted them within a silicon square “sandwich”. This enabled us to perform time-lapse confocal imaging. At least 10 imaginal discs were placed into the silicone square area. When all discs were cut the corresponding slide field was refilled with media mixed with 1 ml of FM4–64 (9 μM) to label the imaginal disc membranes. The imaginal discs were covered with a coverslip and slightly compressed to minimize the free space to avoid imaginal discs spherical deformation (eversion). The ready-made chamber also prevents desiccation. Time-lapse recording was initiated after 5 hours of incubation using inverted Leica SP5 and SP2 or Zeiss LSM700 confocal microscopes and 63 X objectives. Images (Z-stacks of 1 μm thickness) were captured every 10 minutes and in different positions to film at least 3 imaginal discs per slide. Laser intensity was kept at a minimum to avoid photobleaching and to minimize phototoxicity. Image analysis was performed with Leica and Zeiss Confocal Software and ImageJ (NIH Image) was used for mounting time-lapse movies in AVI format.

After 16–18 hours the imaginal discs were recovered using a 200 μl pipette humidified in MM3 media to prevent imaginal discs adhering to the walls of the tips. This was followed by a fast wash in PBS 1X to eliminate MM3 media remnants and fixation in 4% paraformaldehyde for 20 minutes. Antibody incubations were performed following standard procedures. All steps were carried out at room temperature on a shaker. After several washes, imaginal discs were mounted in Vectashield (Vector).

### Statistical analysis

The Microarrays datasets were employed for two different types of assessments: a global comparison of healing-competent (JNK-positive) cells to their non-engaged siblings and dual comparisons between JNK-positive and negative cells of both, wounded and non-wounded discs.

For the global comparison, microarray raw intensities were converted to gene expression estimates using a robust multichip average procedure (RMA). Before the statistical analysis, a pre-filtering step was performed to eliminate the genes presenting low signal (genes should have intensity values larger than 40 units, in at least 25% of samples) and/or minimal variability across samples (inter-quartile range should be larger than the 10% percentile). 6722 probe sets, out of 18952 passed this non-specific prefiltering process. The LIMMA package from Bioconductor was used to fit linear models to log-transform expression data. A moderated t-statistic and log-odds of differential expression were computed to assess for statistically significant changes. The corresponding p-values were corrected for multiple hypotheses testing using a False Discovery Rate criterion. Once the statistical analysis was accomplished we selected genes displaying statistically supported evidence of differential change by applying a p-value and a Fold Change cut off. The results obtained with the global analysis just display expression level changes of JNK-positive vs negative cells in wounded discs.

We also performed dual comparisons employing the microarrays generated from wounded and unwounded discs. The numerical raw data for these comparisons were processed through a between-array quantile normalization step, and a final robust summarization of probe-set signals. The statistical significance of differential gene expression signals was assessed using a moderated t-statistic with the LIMMA Bioconductor package. A relatively mild fold change cut-off (1.3) was applied to facilitate the computation of the interaction terms amongst datasets. A Venn diagram (Fig. 2B) was used to represent the interactions of the different identified subpopulations (**W**, **NW** and **D**). Dual comparisons between wounded and unwounded discs not only gave more information about the behavior of the cells involved in healing respect to their neighboring cells, but allowed us to create graphical representations of the expression of JNK-positive and negative cells for all chips in both conditions, wounded and unwounded.

### Template matching analysis

In order to perform this analysis, and disregard noisy genes, we picked 5106 genes (out of the 6722 from the pre-filtered set) that displayed an FDR-adjusted significance p-value < 0.05, for at least one of the three following contrasts: changes in expression between JNK-positive and negative cell samples in wounded (**W**), in non-wounded (**NW**) conditions, and when significant differences could be established between the both aforementioned expression changes (**D**).

Gene expression values were then discretized into a three level scale [high (**3**), medium (**2**) and low (**1**)] for all expressed genes in each condition. Different template profiles were generated (**1.1.1.1** to **3.3.3.3**) and clusters were defined from gene profiles that could be associated (Pearson correlation R^2^ > 0.95) to respective template profiles.

### Gene mapping in the genome

All genes identified through our two analyses (global and dual comparisons) with a fold change higher or lower than 2 and -2 respectively were mapped to the fly genome using the RefSeq track of the UCSC genome browser. Only one transcript per gene was considered (the first one present in the annotations). The file refGene.txt was used to retrieve the coordinates of each gene in RefSeq. The statistics of gene distribution on each chromosome were computed using the 19.670 unique RefSeq genes contained in the file refGene.txt. To measure the statistical significance of the gene distribution, 1.000 random sets of genes with the same size were generated and evaluated by p-value using a hyper-geometric distribution. The sets of up- and downregulated genes in the microarray were then mapped in the genomes using appropriate catalogues.

### Localized cluster identification and characterization

A cluster was defined as a group of neighboring genes located in a limited region of the genome, but not necessarily consecutive, that shows the same expression pattern (upregulation or downregulation) in the microarray experiment. Clusters were determined by the physical position in the genome, the number of co-expressed genes and the total number of genes in the genomic fragment. To evaluate the significance of the number of clusters identified in our microarray, 100 random sets with the same size and chromosomal gene distribution were generated from the sets of genes involved in wound healing. The clustering analysis was then performed in such data sets to infer the number of clusters that could be obtained by chance (expected clusters).

Different values were computed (using refGene.txt): the average gene length, the gene length distribution, the number of bidirectional/opposed genes on each cluster according to their strand, and the average intergenic distance. Such values were compared to the whole population of genes annotated in the fruit fly genome, to evaluate their significance.

### Functional annotations

A hypergeometric test (GOStats) was performed to assess GO term overrepresentation relative to aleatory assignment in our selected categories (**WO** and **W/NW/D**). GO terms and relationships for *Drosophila* were downloaded (gene_ontology_edit.obo, release 1.2 and gene_association.fb, release 1.94) from the GO web site. Annotation of the different gene sets was done at level 3 of the 'molecular function' ontology tree. We climbed up in the ontology to obtain the corresponding parent term at level 3 for those genes that were annotated at deeper levels in the hierarchical tree. A statistical analysis was performed to test the probability of observing a given GO term significantly enriched in genes belonging to such clusters using a hypergeometric distribution.

### Thorax fusion functional screen

The thorax fusion screen was carried out using a collection of transgenic fly lines carrying UAS-RNAi and UAS overexpressing constructs (see above). To drive their expression to the notum, males from each line were crossed with Pnr-Gal4 virgin females and tested at 3 different temperatures 18°C, 25°C and 29°C. Each phenotype was scored in a semi-quantitative manner based on the affected area within the Pnr-Gal4 expression domain. These phenotypes have been further classified based on the severity of phenotypes as described in the text.

### Wound healing functional screen

Males carrying UAS-RNAi or overexpressing constructs were crossed with either En-Gal4 or Pnr-Gal4 females and maintained at 25°C. Third instar larval imaginal discs were wounded, cultured at 25°C and evaluated for healing defects after 18 hours of culture.

Further Materials and Methods are described in S1 Text.

## Supporting Information

S1 Text Supplemental materials and methods and references. The supplemental Material and Methods include information on *Drosophila* Culture and Fly Stocks, Immunohistochemistry Reagents, including Primary and Secondary Antibodies, and protocols for Cells dissociation and FACS, RNA extraction and Quantification, Linear Amplification and hybridization to Affymetrix chips, Cell culture, DsRNA treatment of S2R+ cells, Live imaging and immunostaining of S2R+ cells and Inhibitor treatment. It also includes new references associated to the described materials. References are labeled in sequential order to those included in the main text.
(DOC)

S1 Fig *In vitro* imaginal disc culture and cells isolation. **A)** Imaginal discs were cut into three pieces and cultured in modified MM3 medium during 16–18 hours. Actin is shown in red (Phalloidin), *puc* expression is shown in green (*puc^E69^*-Gal4 A; UAS-GFP) and nuclei are shown in blue (DAPI). Scale bars are indicated for each panel. Cultured discs fractions were dissociated by trypsinization. **B)** Dissociated cells were subjected to Flow Cytometry and cells expressing *puc* (green) and siblings (white) sorted out. **C)** Cell profiles of control discs (non-GFP expressing discs) (top) and experimentally wounded labeled discs (bottom). GFP intensity is shown in the vertical axis. Particles sizes are shown in the horizontal axis. Control discs cells autofluorescence do not overpass a 10^2^ threshold. Homogeneous populations in terms of GFP intensity (GFP-positive cells in red and GFP-negative cells in blue) were sorted out in a very conservative way to avoid cross-contamination. **D)** To verify GFP expression and integrity, imaginal cells sorted by FACS were cultured and stain with an anti-GFP antibody (*puc*-GFP expression) and DAPI (nuclei). Sorted populations were homogeneous in size and label and viable.
(TIF)

S2 Fig Relative expression changes in the WO subpopulation. Overall changes in expression in wounded discs (for JNK-positive, negative or both cell types) relative to controls for individual genes in the **WO** subpopulation (genes differentially expressed in wounded discs only). Green spots show the level of expression for each replica in JNK-positive cells. Black spots show the levels of expression for each replica in JNK-negative cells.
(PDF)

S3 Fig Relative expression changes in the W/NW/D subpopulation. Overall changes in expression in wounded discs (for JNK-positive, negative or both cell types) relative to controls for individual genes in the **W/NW/D** subpopulation (genes differentially expressed in wounded and in non-wounded discs but distinctly in both conditions). Green spots show the level of expression for each replica in JNK-positive cells. Black spots show the levels of expression for each replica in JNK-negative cells.
(PDF)

S4 Fig Clustering by absolute expression values. Representation of gene clusters by absolute expression scores. Each absolute value of expression for each gene in each replica (3) for the four conditions studied (**JNK^+^ W**, **JNK^-^ W**, **JNK^+^** and **JNK^-^**) was scored. Relative relationships in expression [classified in 3 levels (1/2/3) from lower to higher] between the different conditions were employed to cluster the different genes. For each cluster, all genes absolute expression values were described and represented as cumulative continuous lines in different colors across replicas and conditions (**JNK^+^ W**—1/2/3; **JNK^-^ W**—1/2/3; **JNK^+^**—1/2/3; and **JNK^-^**—1/2/3). 34 distinct clusters of different sizes comprising 313 probes are represented.
(PDF)

S5 Fig Wound healing phenotypes classes. Healing was assayed at 25°C with different Gal4 lines (En or Pnr). Healing phenotypic classes are coded as in the text (**1** -Early (6 hours) defects—Apical Actin; **2**—Early (6 hours) defects—Unstructured Actin and vertex cells rounding; **3**—Early (6 hours) defects—Actin and basal filopodia present but not vertex cells rounding; **4**—Intermediate (12 hours) defects—Vertex cells rounding and CE zippering fails; **5**—Intermediate (12 hours) defects—Gaps between the epithelia and no PE closure; **6**—Late (18 hours) defects—Incomplete closure and **7**—No tissue Relaxation—Tissue folds) (see Functional analysis of “healing” genes section). In green are shown the cellular events achieved in each interference assay. In red are shown those that failed.
(TIF)

S6 Fig TCP1 subunits knockdown leads to impaired healing. **A** to **F**) TCP1 subunits RNAi knockdowns result in impaired healing after 20–24 hours of culture of wounded imaginal wing discs *in vitro*. An early phenotype and an absence of filopodia formation and aberrant actin-rich structures were observed after interference (RNAi) with the expression of different TCP1 subunits as labeled (tested with an En-Gal4 driver). Phalloidin (actin) is shown in red; DAPI (nuclei) in blue. Scale bars are indicated for each panel.
(TIF)

S7 Fig TCP1 knockdown affects the actin cytoskeleton of S2R+ cells. **A** and **B)** *Drosophila* S2R+ cells were transfected with pMT-Act-GFP +/- *TCP1ζ* dsRNA. Four days after transfection control cells (**A**) display a flat morphology and conspicuous filopodia and lamellipodia, while *TCP1ζ* depleted cells (**B**) show a defective actin cytoskeleton and lack filopodia. **C** and **D)** S2R+ cells were transfected with pMT-Act-GFP +/- *TCP1ζ* dsRNA and treated with four days after transfection LatA. In both cases, the actin cytoskeleton completely collapses in 1 hour and the cells become fully rounded. **E** and **F)** pMT-Act-GFP +/- *TCP1ζ* dsRNA transfected cells treated with LatA four days after transfection for 1 hour were let to recover for two days. The control wild type cells (**E**) fully restore the regular arrangement of their cytoskeleton and show a flat morphology. *TCP1ζ* depleted cells **(**green in **F**), on the contrary, failed to reassemble their actin cytoskeleton and display low level of filamentous actin (arrows), while their untransfected siblings fully recover. pMT-Act-GFP expression is shown in green and Phalloidin (F-actin) is shown in red. Scale bars are indicated.
(TIF)

S1 Table Global comparison between JNK signaling positive, healing-engaged cells and their siblings in wounded imaginal discs. 733 upregulated (progressive red series) and 748 downregulated (progressive green series) genes—Fold Change ≥ ± 1.5, p-value < 0.05 (1533 probes) are displayed.
(XLSX)

S2 Table Genes differentially expressed in wounded discs only (WO). The **WO** subpopulation comprises 293 upregulated (progressive red series) and 137 downregulated (progressive green series) genes. Fold Changes ≥ ± 1.5 (and their corresponding p-values < 0.05) for the **W** subpopulation (**JNK^+^ W** vs **JNK^-^ W**), the **NW** subpopulation (**JNK^+^** vs **JNK^-^**) and the **D** subpopulation are displayed.
(XLSX)

S3 Table Genes differentially expressed in wounded and in non-wounded discs but distinctly in both conditions (W/NW/D). The **W/NW/D** subpopulation comprises 610 genes differentially expressed between JNK-positive and negative cells both, in wounded [392 upregulated (red series) and 218 downregulated (green series)] and in non-wounded discs [425 upregulated (red series and 185 downregulated (green series)] but whose respective changes were significativelly different between the two conditions. Fold Changes ≥ ± 1.3 (and their corresponding p-values < 0.05) for the **W** subpopulation (**JNK^+^ W** vs **JNK^-^ W**), the **NW** subpopulation (**JNK^+^** vs **JNK^-^**) and the **D** subpopulation are displayed.
(XLSX)

S4 Table Expression changes subsets in the WO subpopulation. Expression changes subsets were defined for the upregulation **(+)**, the downregulation **(-)** or the absence of changes in their level of expression **(0)** in three conditions: between JNK-positive cells when comparing wounded vs non-wounded discs [**JNK^+^ (W** vs **NW)**]; between JNK-negative cells when comparing wounded vs non-wounded discs [**JNK^-^ (W** vs **NW)**] and between JNK-positive and negative cells in wounded discs [**JNK^+^** vs **JNK^-^ (W)]**. In this way it was possible to define 4 simple subsets (Fig. 2C to 2F): 1A) Upregulated genes in wounded discs that only show upregulation in JNK-positive cells (70 genes). 1B) Upregulated genes in wounded discs that only show downregulation in JNK-negative cells (54 genes). 2A) Downregulated genes in wounded discs that only show downregulation in JNK-positive cells (24 genes). 2B) Downregulated genes in wounded discs that only show upregulation in JNK-negative cells (30 genes). Other complex subsets (from 1C to 9C) showing dual or reverse upregulation or downregulation in different cells (JNK-positive and negative) are represented below. Genes and their expression values are represented as in S2 Table.
(XLSX)

S5 Table Expression changes subsets in the W/NW/D subpopulation. Expression changes subsets were defined for the upregulation **(+)**, the downregulation **(-)** or the absence of changes in their level of expression **(0)** in three conditions: between JNK-positive cells when comparing wounded vs non-wounded discs [**JNK^+^ (W** vs **NW)]**; between JNK-negative cells when comparing wounded vs non-wounded discs [**JNK^-^ (W** vs **NW)**]; between JNK-positive and negative cells in wounded discs **[JNK^+^** vs **JNK^-^ (W)**]; and between JNK-positive and negative cells in non-wounded discs [**JNK^+^** vs **JNK^-^ (W)**]. In this way it was possible to define 6 simple subsets depicting the autonomous up or downregulation of gene expression JNK-positive cells in the absence of any change in JNK-negative cells (Fig. 2G to 2L): 1) Genes that show higher upregulation in wounded discs than in non-wounded (13 genes). 2) Genes that show higher upregulation in non-wounded discs than in wounded (48 genes). 3) Genes that shows higher downregulation in wounded than in non-wounded (4 genes). 4) Genes that show higher downregulation in non-wounded than in wounded (18 genes). 5) Genes upregulated in wounded but downregulated in non-wounded discs (3 genes). 6) Genes downregulated in wounded but upregulated in non-wounded discs (9 genes). Further, six equivalent subsets (1B to 6B) (4, 80, 6, 34, 7 and 13 genes respectively) illustrate non-autonomous changes of gene expression in JNK-negative cells in the absence of any change in JNK-positive cells. Other complex subsets (from 1C to 15C) showing dual or reverse upregulation or downregulation in different cells (JNK-positive and negative) and in wound and non-wound discs are represented below. Genes and their expression values are represented as in S3 Table.
(XLSX)

S6 Table Gene clusters by absolute expression scores. 34 distinct gene clusters by level of expression for the four conditions studied (**JNK^+^ W**, **JNK^-^ W**, **JNK^+^** and **JNK^-^**) were scored (from 81 potential combinatorial possibilities). The numbers of genes in each cluster varied from 1 to 63. They are represented color coded in a concentric Pie Chart. For each cluster, all genes absolute expression values are displayed by their level of expression for each condition (**JNK^+^ W**—1/2/3; **JNK^-^ W**—1/2/3; **JNK^+^**—1/2/3; and **JNK^-^**—1/2/3).
(PDF)

S7 Table Dataset for the chromosomal clustering for the global comparison upregulated genes. Chromosomal clusters of upregulated genes for the global comparison (33) are described by their chromosomal location, number of genes, number of co regulated genes, identity of each gene and each gene’s GO Terms. Genes highlighted in orange are those transcriptionally co regulated during healing.
(PDF)

S8 Table Dataset for the chromosomal clustering for the global comparison downregulated genes. Chromosomal clusters of downregulated genes for the global comparison (19) are described by their chromosomal location, number of genes, number of co regulated genes, identity of each gene and each gene’s GO Terms. Genes highlighted in orange are those transcriptionally co regulated during healing.
(PDF)

S9 Table Dataset for the chromosomal clustering for the W/NW/D downregulated genes. Chromosomal clusters of downregulated genes for the **W/NW/D** comparison (10) are described by their chromosomal location, number of genes, number of co regulated genes, identity of each gene and each gene’s GO Terms. Genes highlighted in orange are those transcriptionally co regulated during healing.
(PDF)

S10 Table Gene ontology terms enrichment analysis of the global comparison: JNK^+^ W vs JNK^-^ W (2 Fold Change, p-value < 0.01). **CC** (Cellular Component), **BP** (Biological Process) and **MF** (Molecular Function) GO enrichment analysis were performed. GO IDs, GO terms, node size, expected number, experimental number, p-value for enrichment and gene symbols are displayed.
(PDF)

S11 Table Gene ontology terms enrichment analysis of the WO subpopulation: (1.3 Fold Change, p-value < 0.05). **CC** (Cellular Component), **BP** (Biological Process) and **MF** (Molecular Function) GO enrichment analysis were performed. GO IDs, GO terms, node size, expected number, experimental number, p-value for enrichment and gene symbols are displayed.
(PDF)

S12 Table Gene ontology terms enrichment analysis of the W/NW/D subpopulation: (1.3 Fold Change, p-value < 0.05). **CC** (Cellular Component), **BP** (Biological Process) and **MF** (Molecular Function) GO enrichment analysis were performed. GO IDs, GO terms, node size, expected number, experimental number, p-value for enrichment and gene symbols are displayed.
(PDF)

S13 Table UAS-RNAi lines dataset. 309 UAS-RNAi lines were gathered from three different sources, the NIG-Fly, VDRC and DRSC to be employed in expression interference experiments in thorax closure or wound healing. Probes’ codes, gene IDs, gene names, gene symbols, RNAi line codes, sources and level of expression of each probe/gene in the different analyzed comparisons (as in S1, S2 and S3 Tables) are displayed.
(XLSX)

S14 Table UAS-Overexpression lines dataset. 21 UAS-Overexpression lines were gathered from different sources (see references), to be employed for the overexpression of different genes in experiments performed in thorax closure or wound healing. Probes’ codes, gene IDs, gene names, gene symbols, UAS codes, sources and level of expression of each probe/gene in the different analyzed comparisons (as in S1, S2 and S3 Tables) are displayed.
(XLSX)

S15 Table Thorax closure phenotypes. From 309 RNAi and 21 UAS lines employed for interference or overexpression in the Pnr-Gal4 domain, 151 and 16 respectively yielded distinguishable phenotypes. Assays were performed at different temperatures (**PNR18**, **PNR25** and **PNR29**) as indicated and phenotypic categories are coded (**A** -Wild Type; **B**—Bristle Defects; **C**—Weak Thorax Cleft; **D**—Thorax Cleft; **E**—Strong Thorax Cleft; **F**—Pupal Lethal and **G**—Embryonic lethal). Phenotypic classes as described in the main text (**1TC** to **7TC**) are indicated. Transcriptomic results as described in S1, S2 and S3 Tables are also displayed.
(XLSX)

S1 Movie Live imaging of imaginal disc healing *in vitro*. Imaginal discs were cultured in modified medium during 16–18 hours. Membranes are shown in red (FM4–64) and *puc* expression is shown in green (*puc^E69^*-Gal4 A; UAS-GFP). Few hours (6) after wounding a clear shortening of the wound edge while cells at the wound margin showed actin-rich protrusions more abundant in the close proximity of the vertex of the healing wound is observed. In addition to these filopodia, we detected a rapid actin cytoskeleton reorganization that occurred in both, the columnar and the peripodial epithelium with the formation of a purse-string. 12 hours after cutting the discs, the size of the gap is considerably reduced and by 16–18 hours the incised discs have zipped and sealed the gap. Hence, healing does undergo a slightly slower kinetics than that showed for implanted discs (12 hours for completion). Also, in contrast to *in vivo* cultured discs, a mass of cell debris was frequently observed, attached to the wound vertex that was actively extruded upon edges apposition. *puc* expression initiates after 2 hours in culture in scattered cells at the wound edges. At 7 hours post wounding most of the cells at the wound edges express *puc*. This expression develops further and reaches a maximum at 15 hours of cultured when several rows of cells decorated the almost fully healed region.
(MOV)

S2 Movie Healing failure upon interference in TCP 1ζ expression. Downregulation of TCP1ζ-CG8231 with a specific RNAi construct in the posterior compartment with an En-Gal4 driver results in healing failure. CE and PE fail to heal the injury, actin does not accumulate and numerous apoptotic figures are observed. GFP expression corresponds to posterior compartment cells expressing a UAS-GFP reporter.
(MOV)
