# Supplementary material for: Identification and Functional Analysis of Healing Regulators in Drosophila
Source: PLoS Genet. 2015 Feb 3;11(2):e1004965. doi: 10.1371/journal.pgen.1004965 (PMC4315591; doi:10.1371/journal.pgen.1004965)
Supplement: S1 Text — The supplemental Material and Methods include information on Drosophila Culture and Fly Stocks, Immunohistochemistry Reagents, including Primary and Secondary Antibodies, and protocols for Cells dissociation and FACS, RNA extraction and Quantification, Linear Amplification and hybridization to Affymetrix chips, Cell culture, DsRNA treatment of S2R+ cells, Live imaging and immunostaining of S2R+ cells and Inhibitor treatment. It also includes new references associated to the described materials. References are labeled in sequential order to those included in the main text. (DOC) [file pgen.1004965.s001.doc]

**Identification and Functional Analysis of Healing Regulators in *Drosophila***

**Álvarez-Fernández et al**

**SUPPLEMENTAL MATERIALS AND METHODS**

***Drosophila* Culture and Fly Stocks**

All the *Drosophila* lines were maintained on Standard culture media and according to standard procedures. Flies carrying dsRNAs under the control of UAS sequences (UAS-RNAi) were ordered from the VDRC, Nig-FLY and DRSC Stock Centers and are enlisted in Table S13. UAS overexpressing constructs are included in Table S14 . Other Stocks used were as follow: Pnr-Gal4 (*yw; if/CyO; pnr-Gal4/TM6B*) ; Pnr-Gal4–Puc*^E69^* (*yw; If/CyO; pnr-Gal4, puc^E69^-LacZ/TM6B*) ; Jupiter-GFP (*yw; JupiterZCL0931/TM3, Ser*) (BDSC 6825); Life actin-GFP (*yw; UAS-Lifeact-GFP*) (BDSC 35544); Puc*^E69^*-Gal4 A; UAS-GFP (*yw; UAS-GFP; puc^E69^-Gal4 A/TM6B*) and Puc*^E69^*-Gal4 I; UAS-GFP (*yw; UAS-GFP; puc^E69^-Gal4 I/TM6B*) ; En-Gal4; UAS-GFP (*yw; en-Gal4; UAS-GFP*) (BDSC 1973).

**Immunohistochemistry Reagents**

Primary antibodies are detailed in the tables below. For nuclei staining, DAPI (Sigma) was used at a dilution of 1:1000 from a stock solution at 1 mg/ml. The actin cytoskeleton was labeled with phalloidin coupled with rhodamine (Molecular Probes) 1:1000 (1 mg/ml).

Primary Antibodies

| **Antibody** | **Source** | **Reference** | **Working dilution** |
| --- | --- | --- | --- |
| Anti-Talin | Nicholas Brown |  | 1:50 |
| Anti-Integrin (β PS) | Hybridoma Bank | CF6G11 | 1:50 |
| Anti-Sqh | Robert Ward |  | 1:50 |
| Anti-MMP1 | Hybridoma Bank | 5H7B11 | 1:20 |
| Anti-Beta-Gal | Cappel | 8559761 | 1:200 |
| Anti-Tubulin-FITC | Invitrogen tech | F2168.2ML | 1:500 |
| Anti Tubulin (beta) | Hybridoma Bank | E7 | 1:500 |

Secondary Antibodies

| **Antibody** | **Reference** | **Working dilutions** |
| --- | --- | --- |
| Mouse Alexa-488 | Invitrogen-A11029 | 1:500 |
| Mouse Alexa-555 | Invitrogen-A21424 | 1:500 |
| Rabbit Alexa-488 | Invitrogen-A11034 | 1:500 |
| Rabbit-Alexa-555 | Invitrogen-A21429 | 1:500 |
| Mouse-Cy5 | Jackson 115-175-205 | 1:500 |

**Cells dissociation and FACS**

Before sorting cells by FACS, imaginal discs (*yw; UAS-GFP; puc^E69^-Gal4 I/TM6B*) were dissociated by trypsinization. Trypsinization was carried out in 35 mm dishes (Nunclon) with 1 ml of Trypsin-EDTA (Sigma –T4174) 9 X and 5 ml of Hoechst 33342 (final dilution 0.1 μg/ml). Around 100 imaginal discs were shaken for 2-3 hours (170 rpm) at room temperature. During the last 30 minutes, 5 ml of propidium iodide (1 mg/ml, Invitrogen) was added to detect dead cells. The degree of dissociation was monitored under a microscope.

The selection and sorting of GFP positive and negative cells were done by Flow Cytometry using a MoFlo machine based on drop formation (DakoCytomation). Simple excitations were done with an ion-argon laser (Coherent Enterprise II). Optic alignments were based on optimal signals for 10 µm fluorescence particles (Flowcheck, Coulter Corporation). We considered different parameters for cell sorting: GFP and PI fluorescence and FSC and SSC refringency obtained using a 488 nm laser line at 220 mW and Hoechst 33342 fluorescence obtained by UV excitation (25 mW). The different areas of cell sorting were defined by the combination of the green fluorescence intensity (GFP), red (Propidium Iodide - PI), blue (Hoechst 33342) and parameters (FSC and SSC). GFP positive cells were those with a green fluorescence signal higher than 10^1^ and GFP negative cells were those with no GFP signal. Dead cells were those with a PI signal in between 10^1^ - 10^2^. We considered alive and intact cells those with Hoechst intensity around 10^3^. This procedure allowed us to differentiate whole cells from degraded cells and aggregates.

**RNA extraction and Quantification**

RNA was extracted using the Qiagen RNeasin® Micro Kit. In order to obtain approximately 10 ng of RNA we employed around 30.000 to 40.000 cells. The quality and quantity of the extracted RNA were analyzed using the RNA Pico Lab Chip with an Agilent Bioanalyzer (Agilent Technologies).

**Linear Amplification and hybridization to Affymetrix chips**

Due to the small size of the sample, the RNA was indirectly labeled with two rounds of linear amplification. The resulting cRNAs were hybridized to GeneChip *Drosophila* Genome 2.0 Arrays (Affymetrix, Santa Clara, CA). Three independent samples (biological replicas) for each population were employed. Raw data and expression values for all genes have been deposited in the NCBI-GEO.

**Cell culture**

*Drosophila* S2R+cells were cultured in Schneider’s medium (GIBCO, Invitrogen) supplemented with 10% heat inactivated fetal bovine serum (GIBCO, Invitrogen), penicillin and streptomycin (100 units/ml and 100 μg/ml) (GIBCO, Invitrogen) in 25 ml cell culture flasks at 25°C. Cells were maintained in cultures by splitting (1:5) every 4 days.

**DsRNA treatment of S2R+ cells**

DsRNAs were used to interfere with gene function in Schneider S2R+ cells. The dsRNAs were obtained from the DRSC stock center (CG8351 - DRSC16410 and CG8231 - DRSC20095). Approximately 200.000 cells/well were plated in 24 well plates a day prior to transfection in a final volume of 500 µl. They were treated with Effectene (Qiagen) following standard procedures. 2 μg/μl of a control cellular reporter DNA (pMT-Act-GFP) to label the actin cytoskeleton and 5μg/μl of dsRNA were transfected simultaneously. The next day, the transfection medium was replaced by fresh medium and the cells were incubated for four additional days at 25ºC and subjected afterwards to either live imaging or immunostaining.

**Live imaging and immunostaining of S2R+ cells**

Transfected *Drosophila* S2R+ cells were cultured in 2 wells chambered cover glass (2 ml capacity, NUNC 155380). Live imaging was performed with Leica SP2 or Zeiss LSM700 microscopes at 40X magnification. Images (Z-stacks with 1μm thickness sections) were acquired at intervals of 5 minutes. Immunostaining of transfected and/or dsRNA-treated cells was performed using standard protocols as described .

**Inhibitor treatment**

The actin polymerization inhibitor Latrunculin A (LatA) was used to depolymerize F-actin in S2R+ cells. *Drosophila* S2R+ cells were split at 200.000 cells/per well in 24 well plate a day prior to transfection in a final volume of 500 μl. The next day, the cells were transfected with control DNA and dsRNA and incubated for 4 days at 25°C. After 4 days the cells were treated with LatA (0.5 mg/ml) for 1 hour and let to recover for 48 hours. Last, they were immunostained with Phalloidin and imaged.

**SUPPLEMENTAL REFERENCES**

88. Kerber B, Monge I, Mueller M, Mitchell PJ, Cohen SM (2001) The AP-2 transcription factor is required for joint formation and cell survival in Drosophila leg development. Development 128: 1231-1238.

89. McNeill H, Yang CH, Brodsky M, Ungos J, Simon MA (1997) mirror encodes a novel PBX-class homeoprotein the functions in the definition of the dorsal-ventral border in the Drosophila eye. Genes & Development 11: 1073-1082.

90. Horiuchi D, Barkus RV, Pilling AD, Gassman A, Saxton WM (2005) APLIP1, a kinesin binding JIP-1/JNK scaffold protein, influences the axonal transport of both vesicles and mitochondria in Drosophila. Current Biology 15: 2137-2141.

91. Schock F, Purnell BA, Wimmer EA, Jackle H (1999) Common and diverged functions of the Drosophila gene pair D-Sp1 and buttonhead. Mechanisms of Development 89: 125-132.

92. Diez del Corral R, Aroca P, Gomez-Skarmeta JL, Cavodeassi F, Modolell J (1999) The Iroquois homeodomain proteins are required to specify body wall identity in Drosophila. Genes & development 13: 1754-1761.

93. Steneberg P, Englund C, Kronhamn J, Weaver TA, Samakovlis C (1998) Translational readthrough in the hdc mRNA generates a novel branching inhibitor in the Drosophila trachea. Genes & Development 12: 956-967.

94. Brown S, Castelli-Gair Hombria J (2000) Drosophila grain encodes a GATA transcription factor required for cell rearrangement during morphogenesis. Development 127: 4867-4876.

95. Grillenzoni N, van Helden J, Dambly-Chaudiere C, Ghysen A (1998) The iroquois complex controls the somatotopy of Drosophila notum mechanosensory projections. Development 125: 3563-3569.

96. Guillemin K, Williams T, Krasnow MA (2001) A nuclear lamin is required for cytoplasmic organization and egg polarity in Drosophila. Nature Cell Biology 3: 848-851.

97. Lin Y-Y, Gubb D (2009) Molecular dissection of Drosophila Prickle isoforms distinguishes their essential and overlapping roles in planar cell polarity. Developmental Biology 325: 386-399.

98. Kopp A, Duncan I, Carroll SB (2000) Genetic control and evolution of sexually dimorphic characters in Drosophila. Nature 408: 553-559.

99. Levashina EA, Langley E, Green C, Gubb D, Ashburner M, et al. (1999) Constitutive activation of toll-mediated antifungal defense in serpin-deficient Drosophila. Science 285: 1917-1919.

100. Budnik V, Koh YH, Guan B, Hartmann B, Hough C, et al. (1996) Regulation of synapse structure and function by the Drosophila tumor suppressor gene dlg. Neuron 17: 627-640.

101. Bachmann A, Timmer M, Sierralta J, Pietrini G, Gundelfinger ED, et al. (2004) Cell type-specific recruitment of Drosophila Lin-7 to distinct MAGUK-based protein complexes defines novel roles for Sdt and Dlg-S97. J Cell Sci 117: 1899-1909.

102. Zhang L, Ward REt (2011) Distinct tissue distributions and subcellular localizations of differently phosphorylated forms of the myosin regulatory light chain in Drosophila. Gene Expr Patterns 11: 93-104.

103. Rogers SL, Wiedemann U, Stuurman N, Vale RD (2003) Molecular requirements for actin-based lamella formation in Drosophila S2 cells. The Journal of cell biology 162: 1079-1088.

104. Pereira AM, Tudor C, Kanger JS, Subramaniam V, Martin-Blanco E (2011) Integrin-dependent activation of the JNK signaling pathway by mechanical stress. PLoS One 6: e26182.
