# Supplementary figures and images for: Identification and Functional Analysis of Healing Regulators in Drosophila
Source: PLoS Genet. 2015 Feb 3;11(2):e1004965. doi: 10.1371/journal.pgen.1004965 (PMC4315591; doi:10.1371/journal.pgen.1004965)

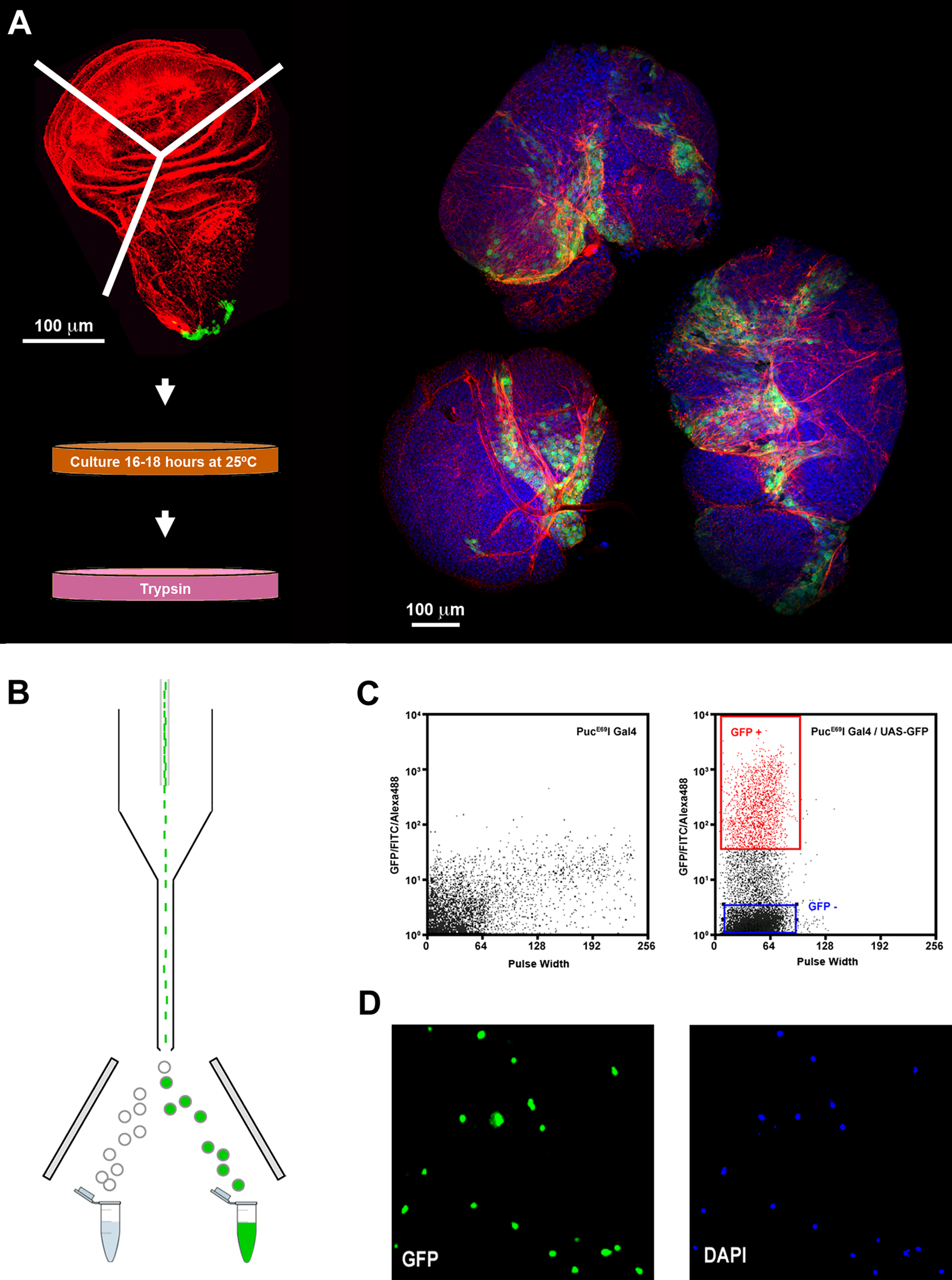

Supplement: S1 Fig — A) Imaginal discs were cut into three pieces and cultured in modified MM3 medium during 16–18 hours. Actin is shown in red (Phalloidin), puc expression is shown in green (pucE69-Gal4 A; UAS-GFP) and nuclei are shown in blue (DAPI). Scale bars are indicated for each panel. Cultured discs fractions were dissociated by trypsinization. B) Dissociated cells were subjected to Flow Cytometry and cells expressing puc (green) and siblings (white) sorted out. C) Cell profiles of control discs (non-GFP expressing discs) (top) and experimentally wounded labeled discs (bottom). GFP intensity is shown in the vertical axis. Particles sizes are shown in the horizontal axis. Control discs cells autofluorescence do not overpass a 102 threshold. Homogeneous populations in terms of GFP intensity (GFP-positive cells in red and GFP-negative cells in blue) were sorted out in a very conservative way to avoid cross-contamination. D) To verify GFP expression and integrity, imaginal cells sorted by FACS were cultured and stain with an anti-GFP antibody (puc-GFP expression) and DAPI (nuclei). Sorted populations were homogeneous in size and label and viable. (TIF) [file pgen.1004965.s002.tif]

## **Supplementary Figure 2**

WO Gene Set

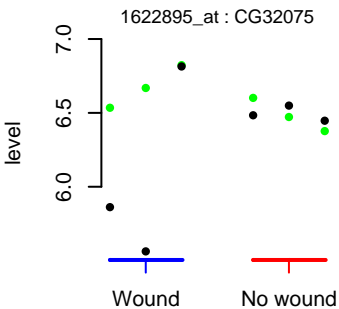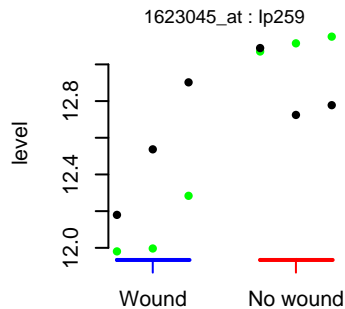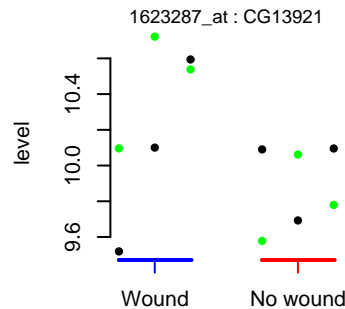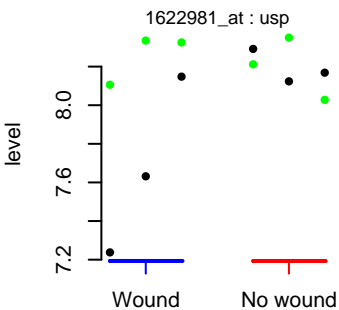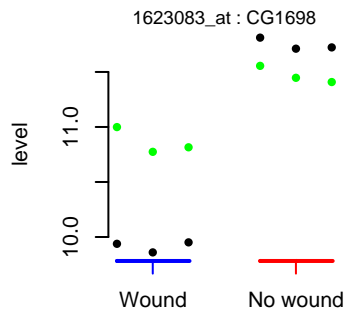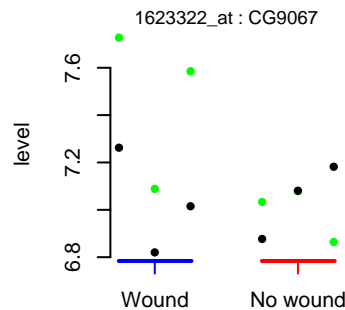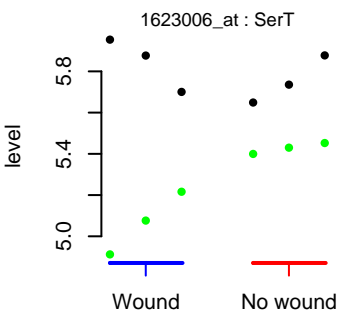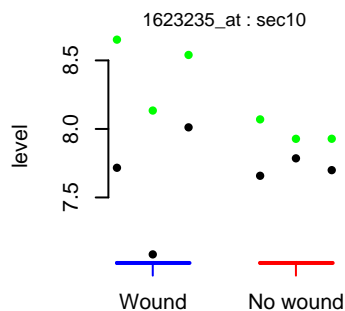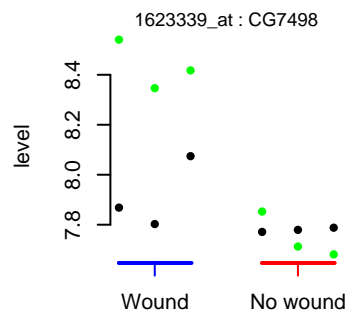

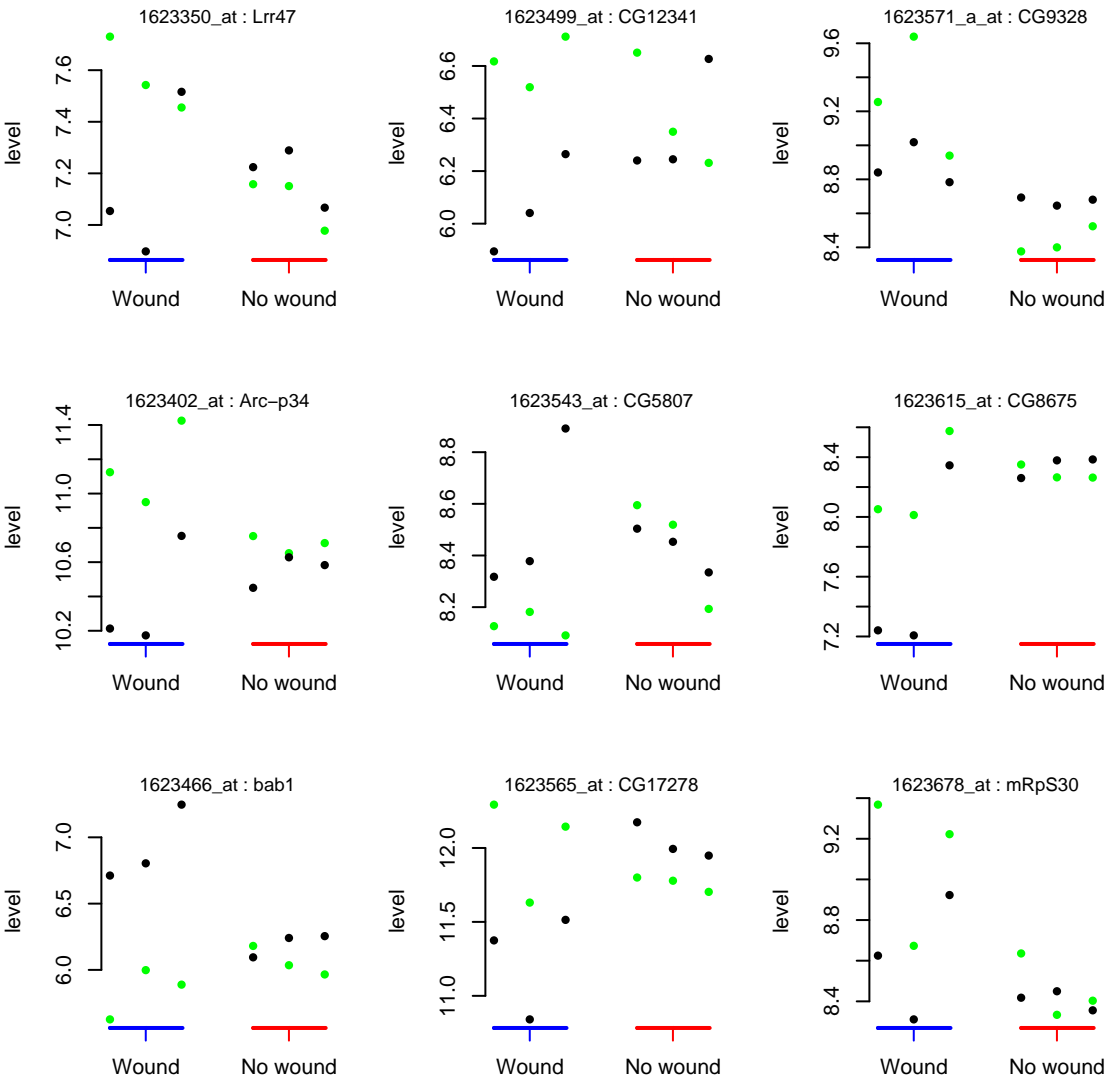

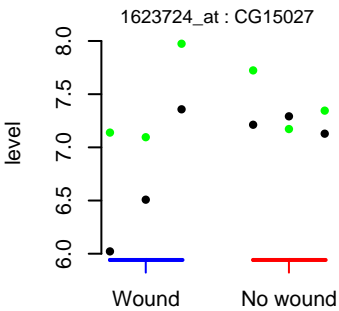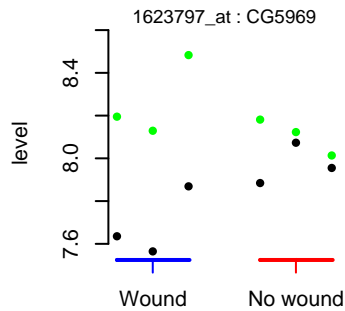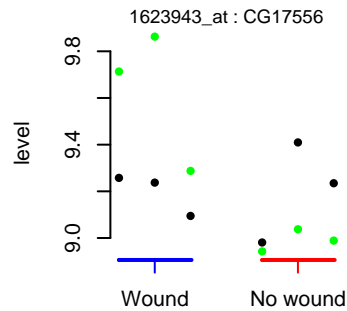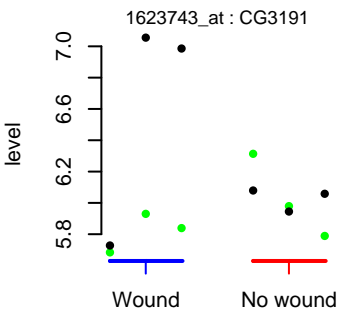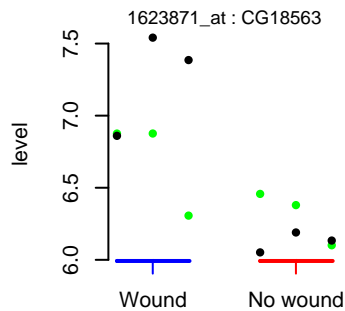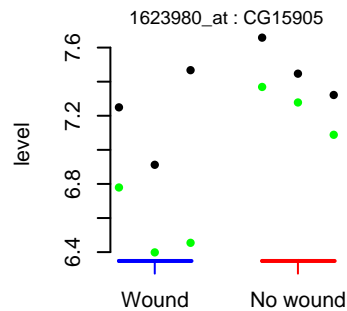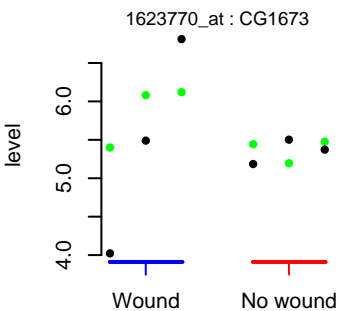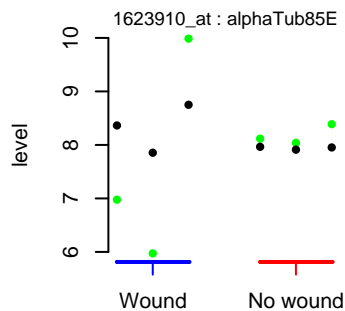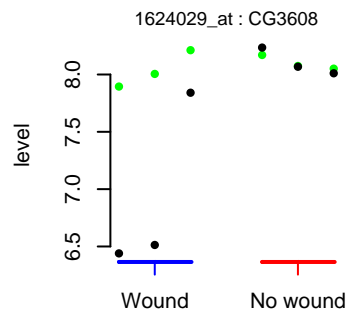

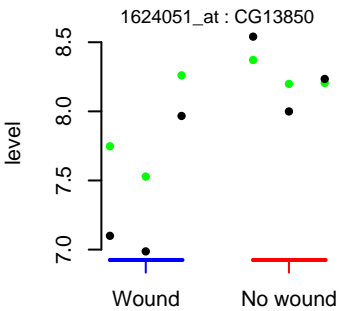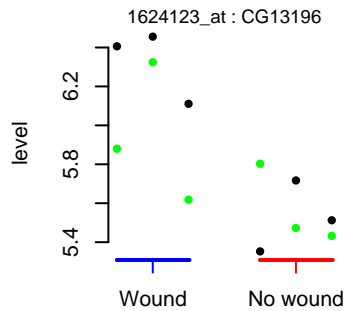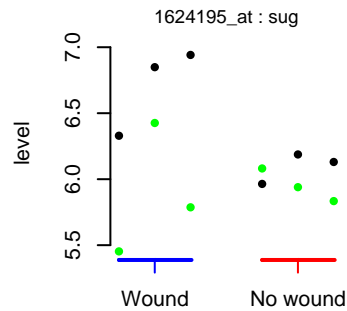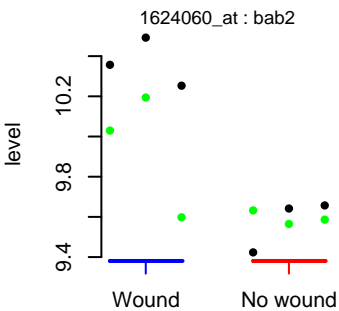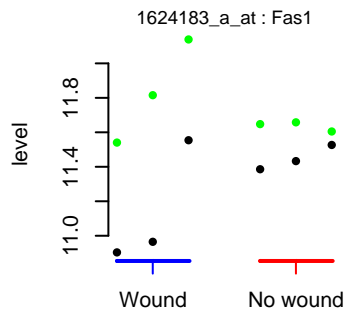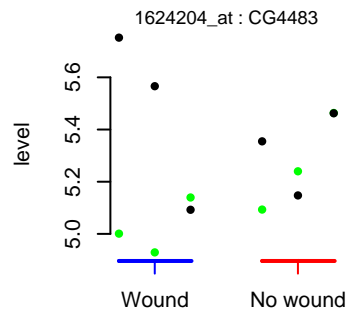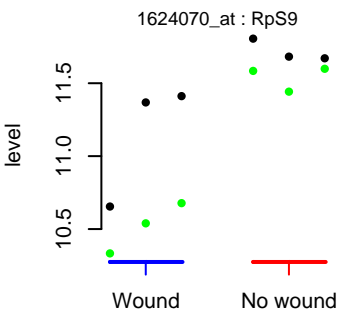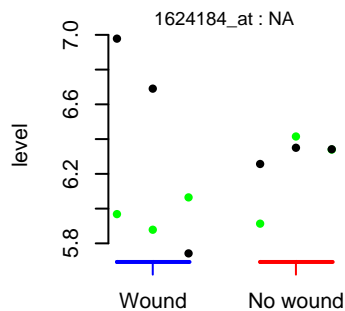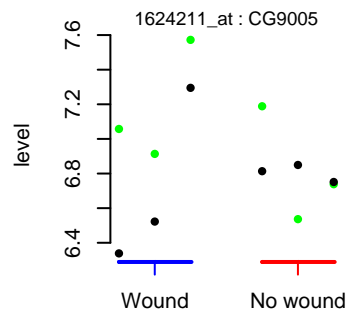

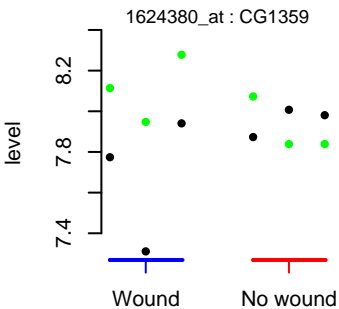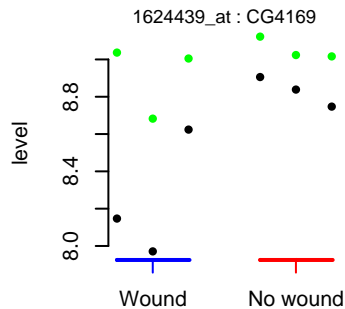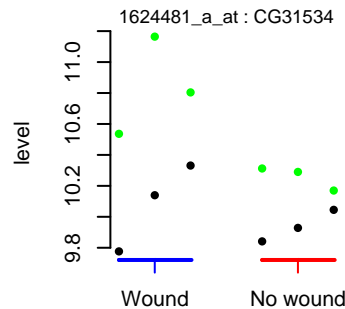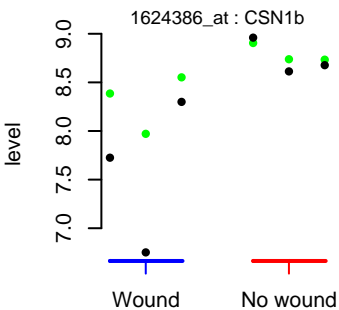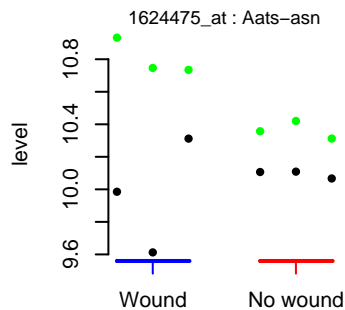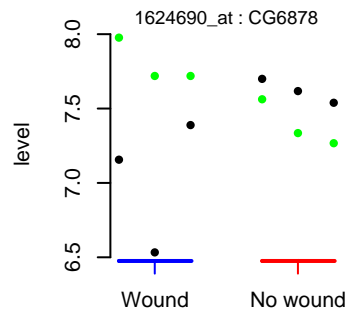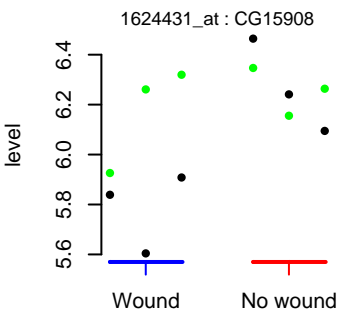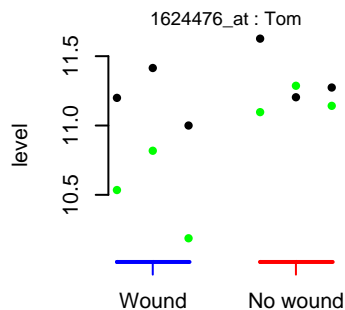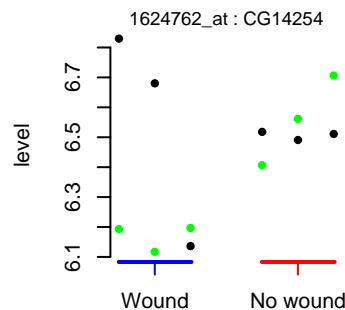

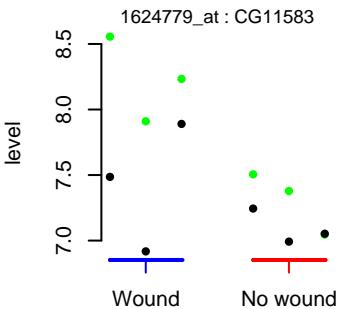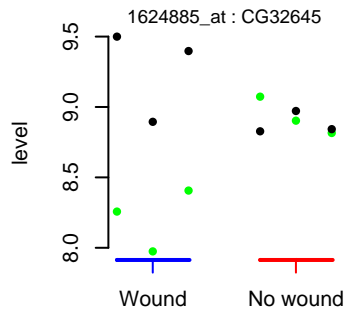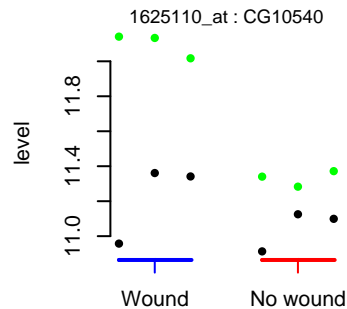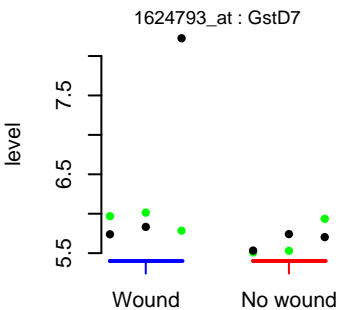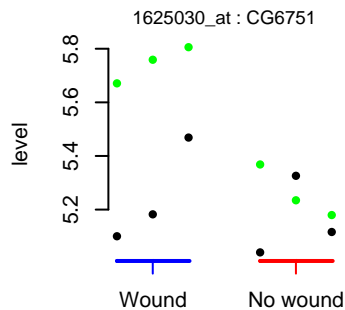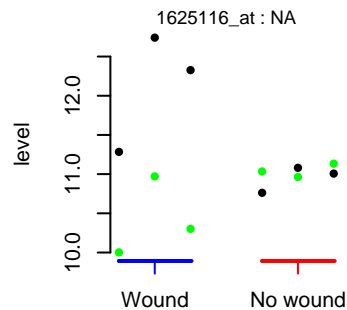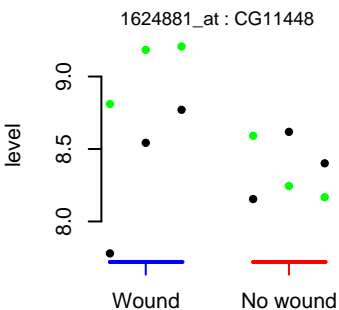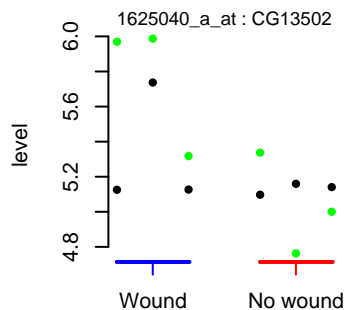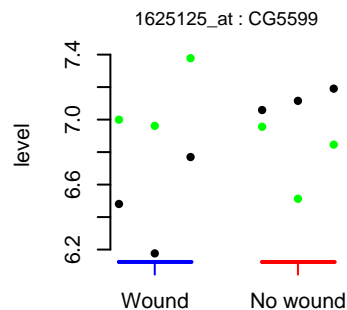

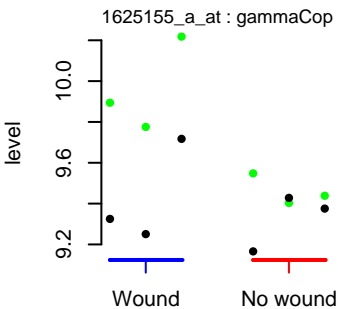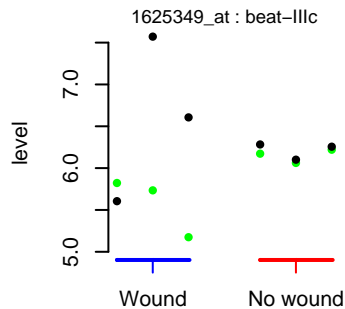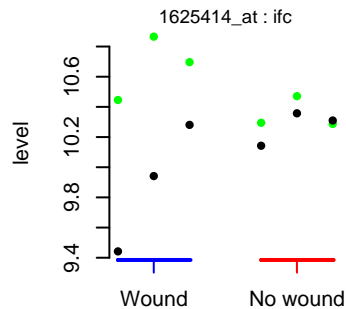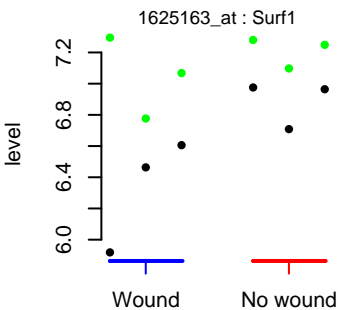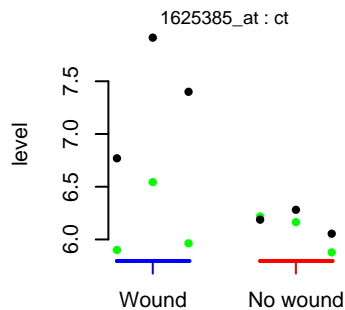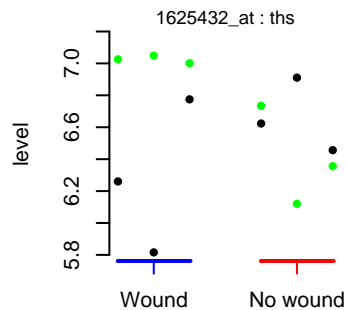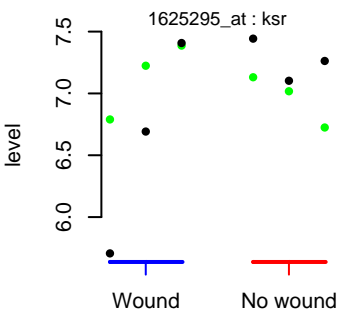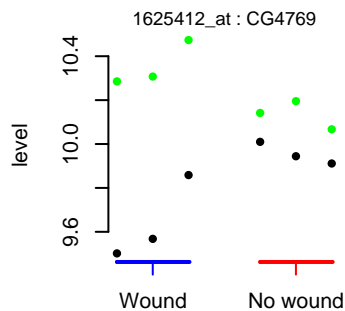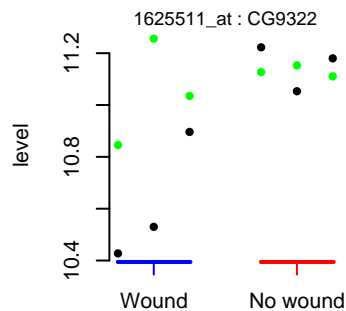



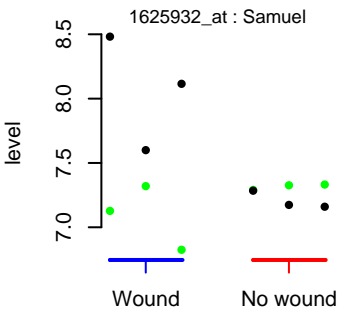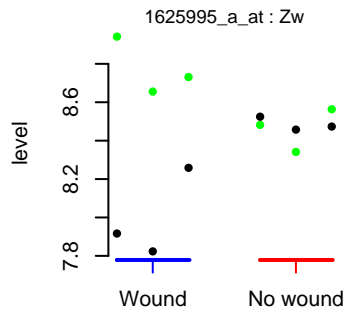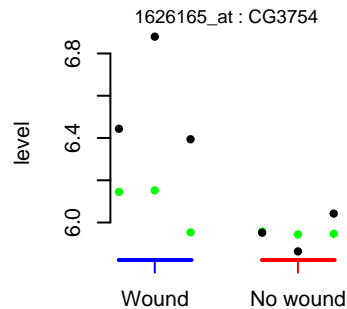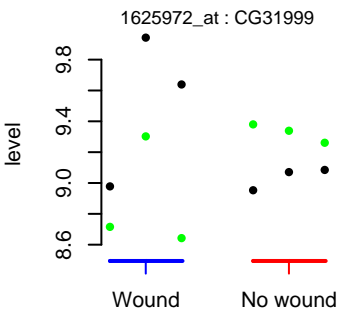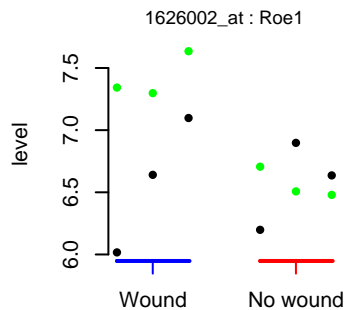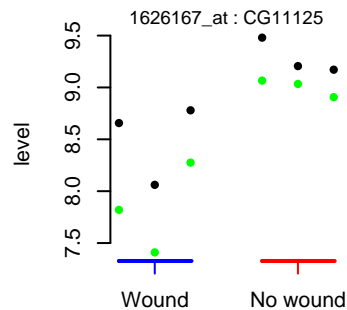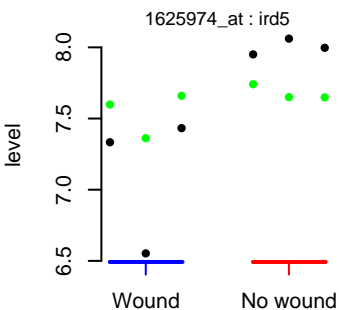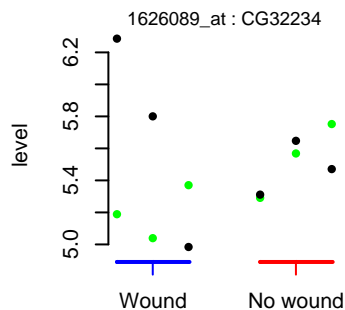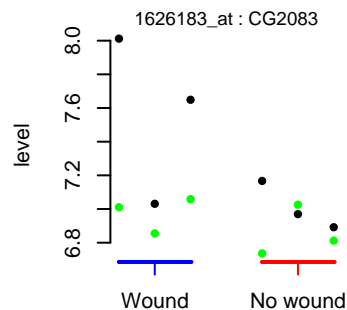

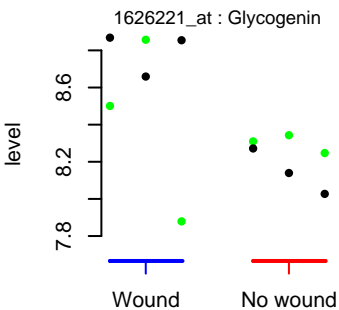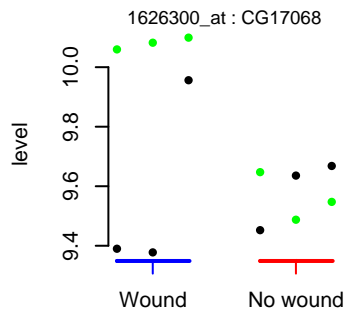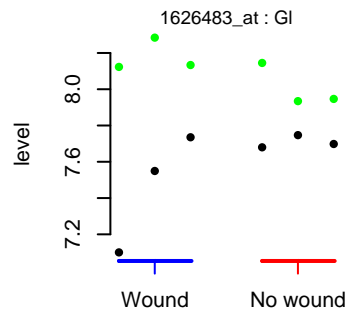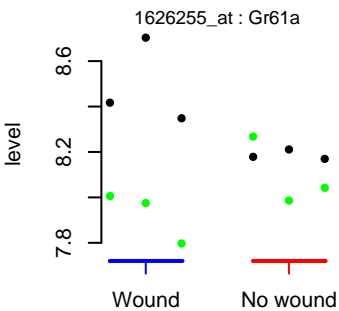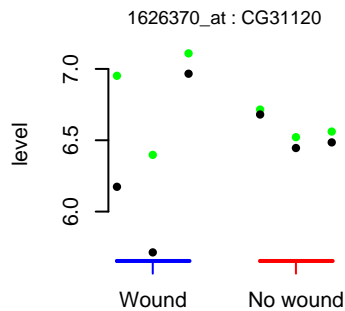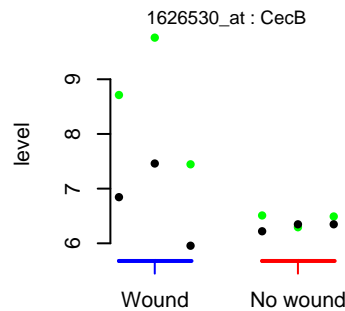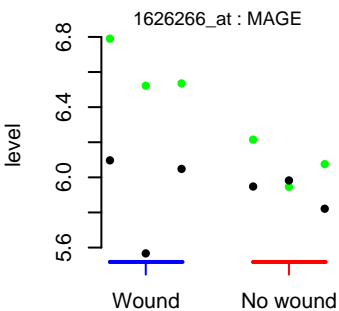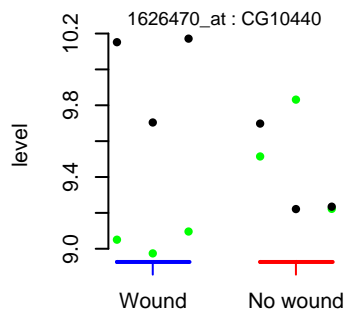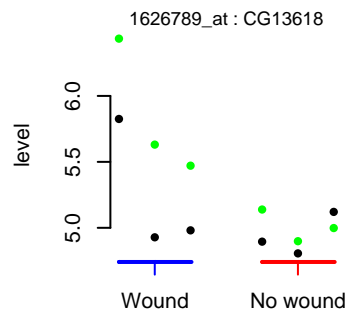

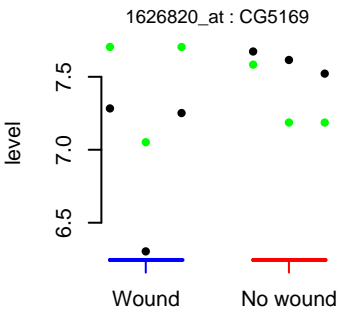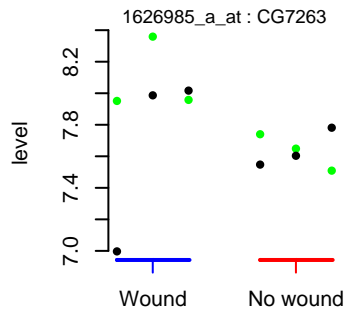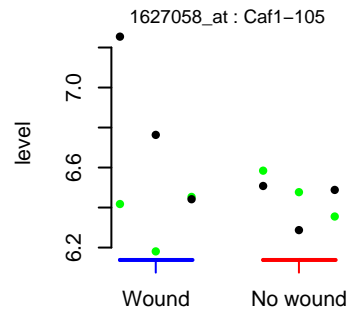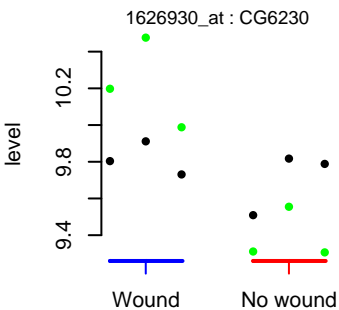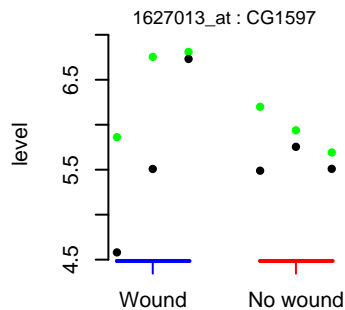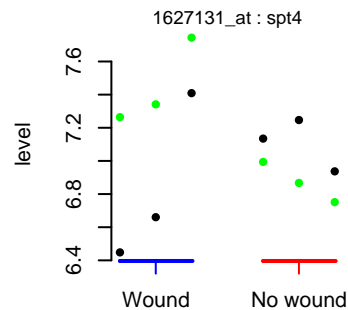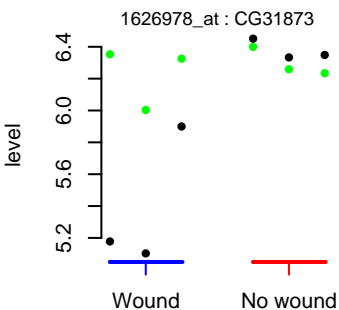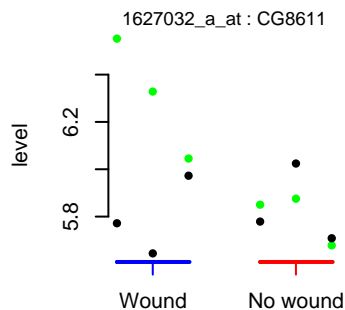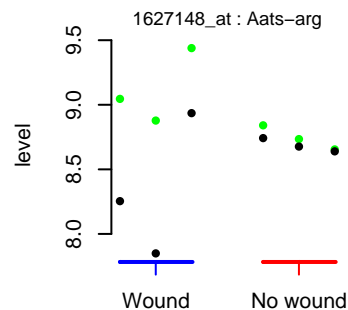

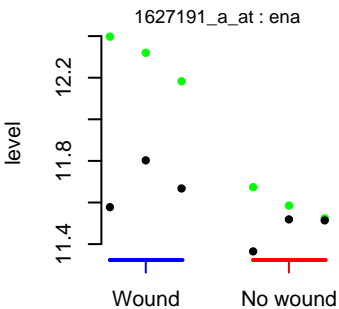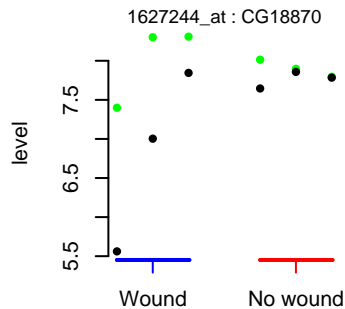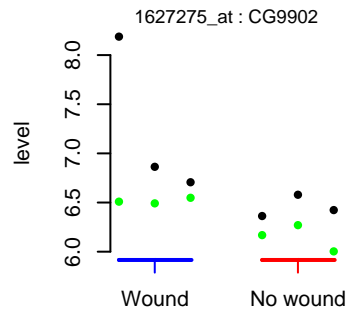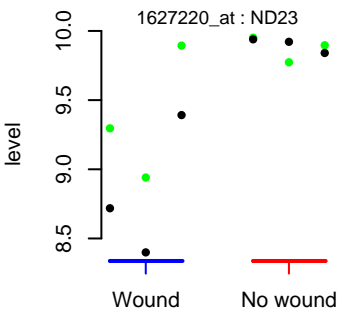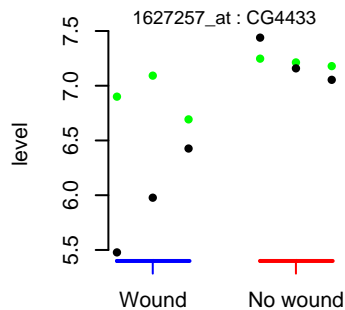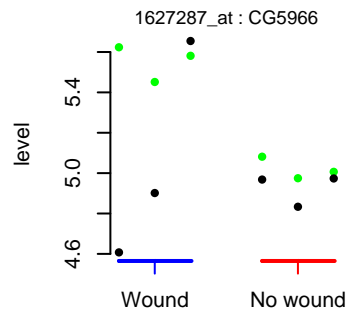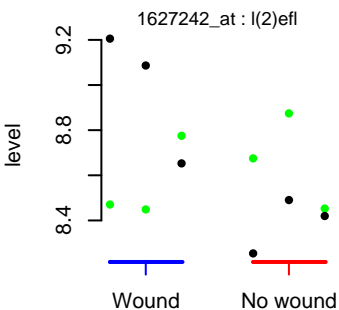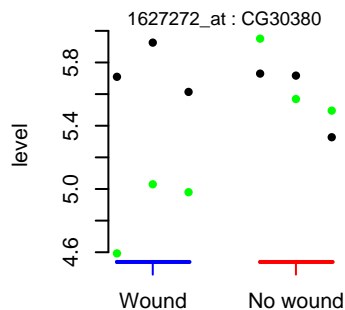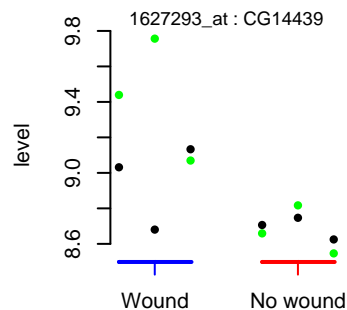

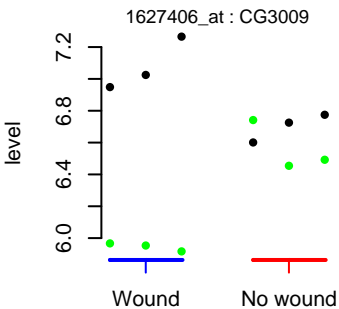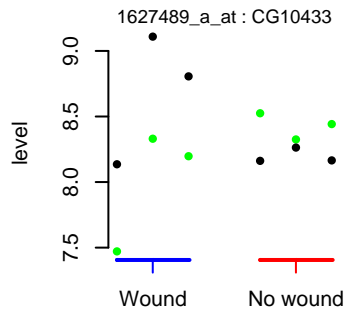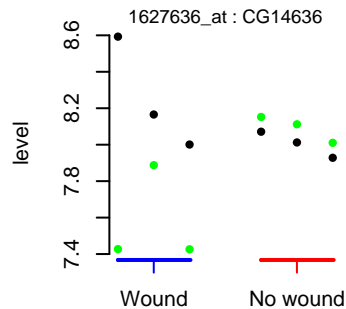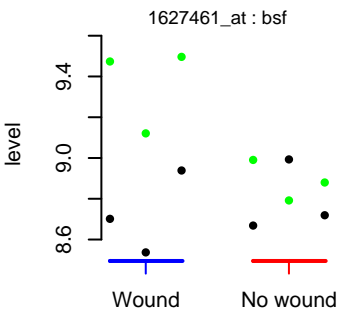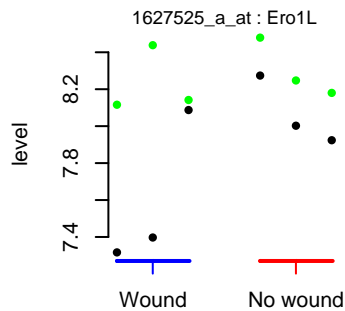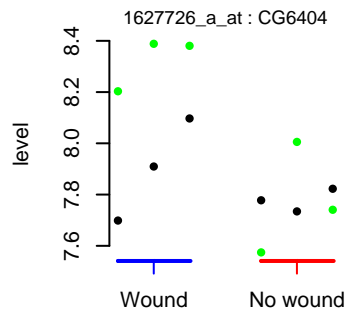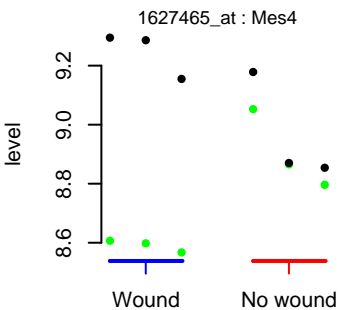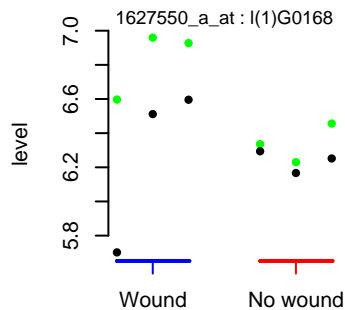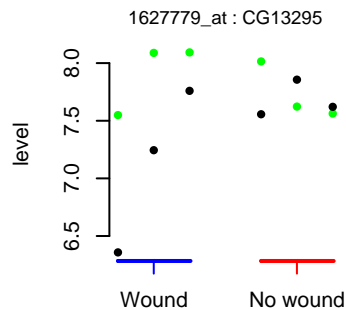

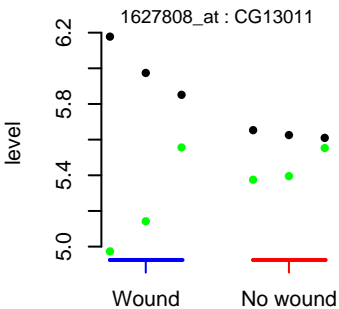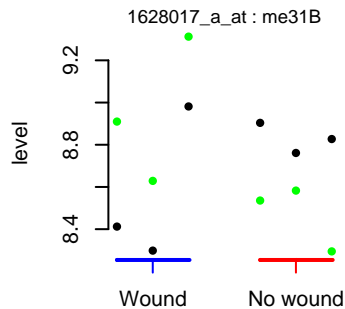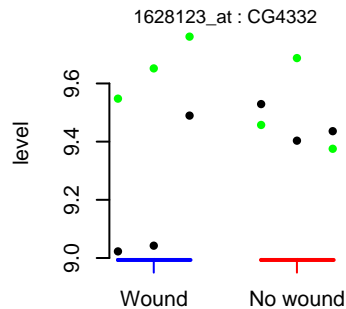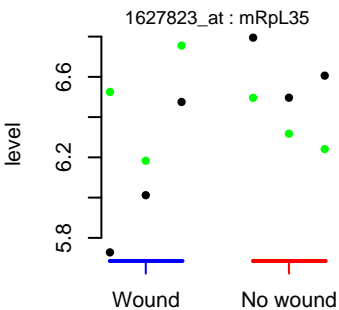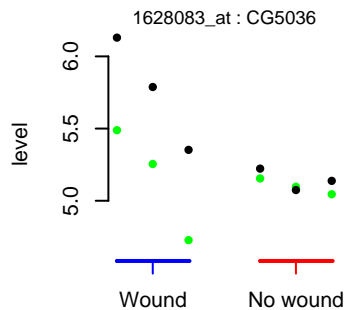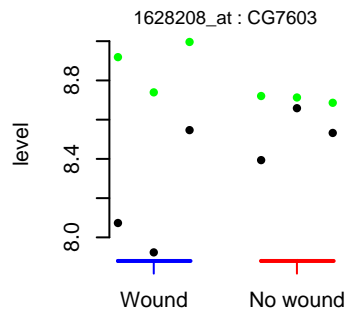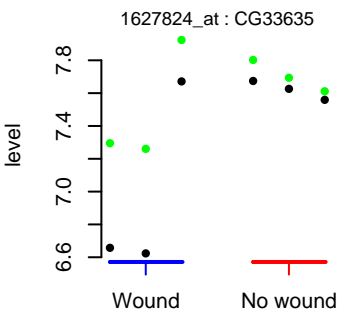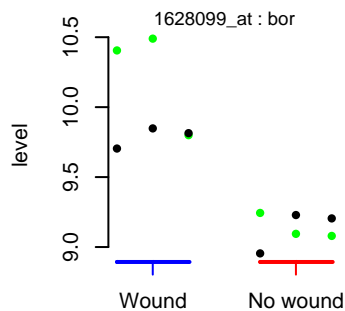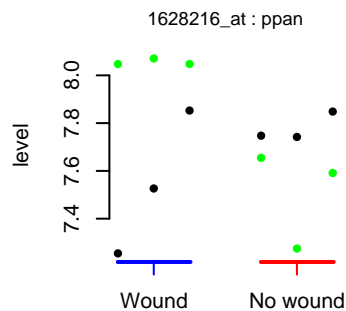

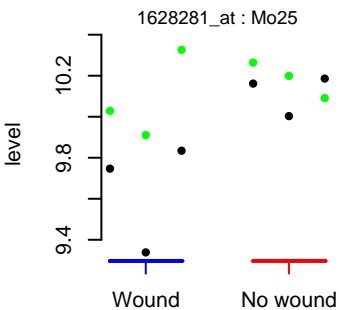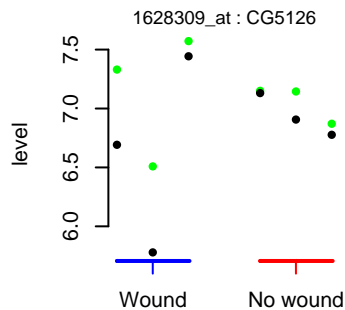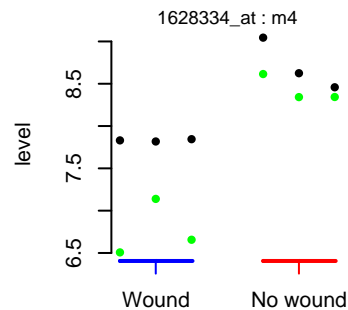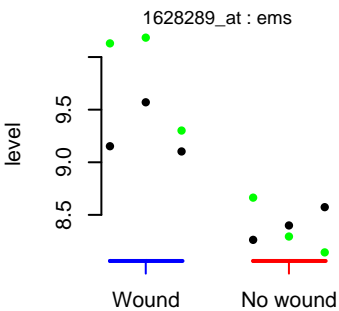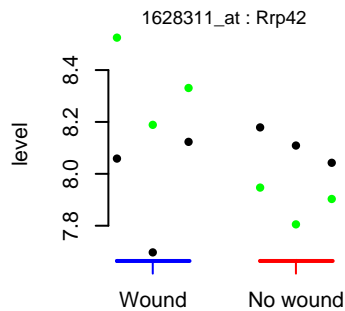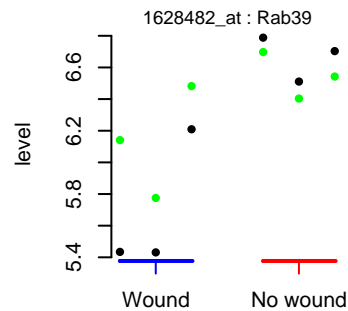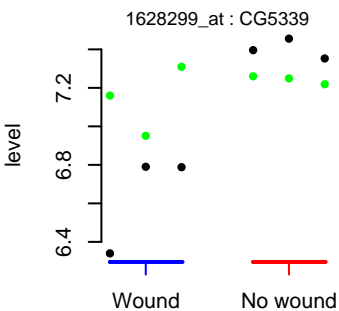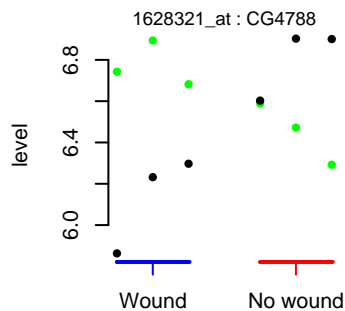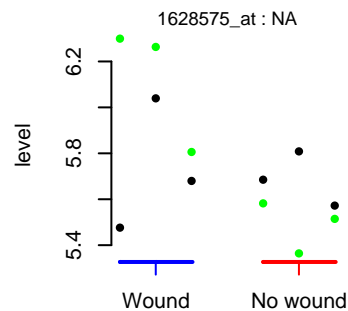

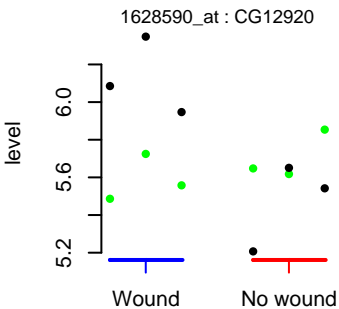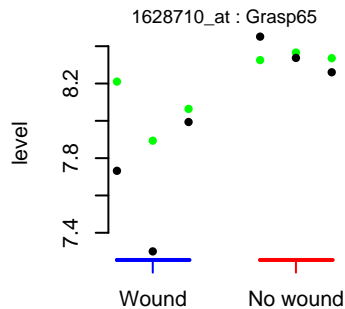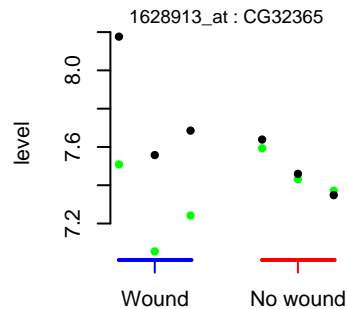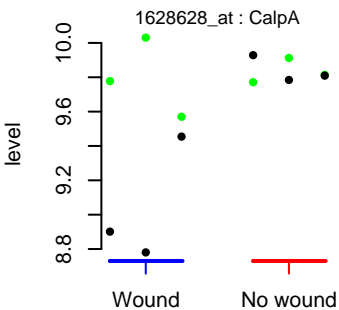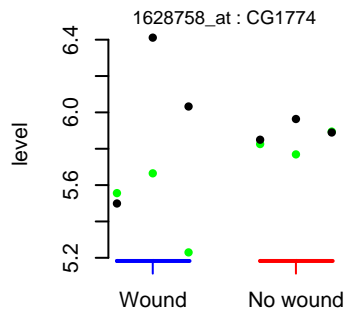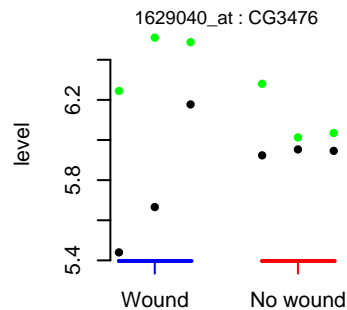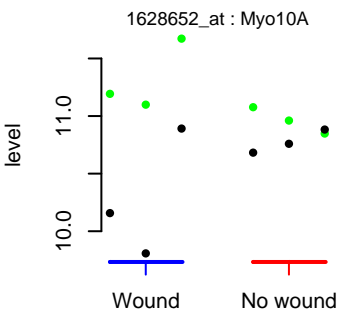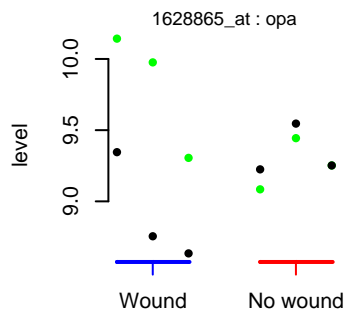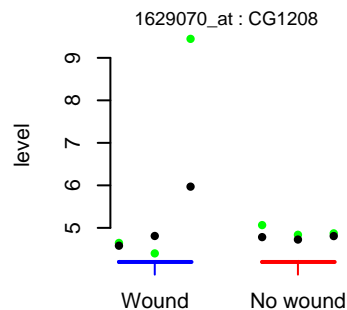

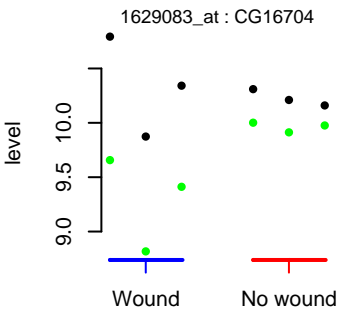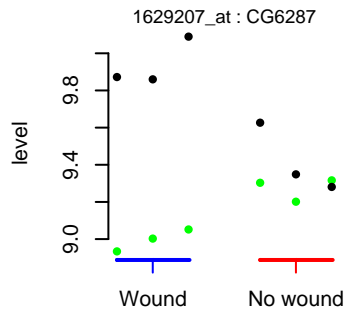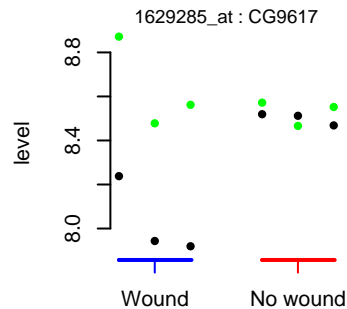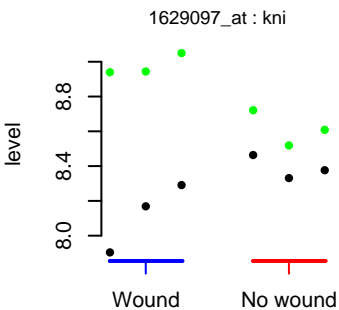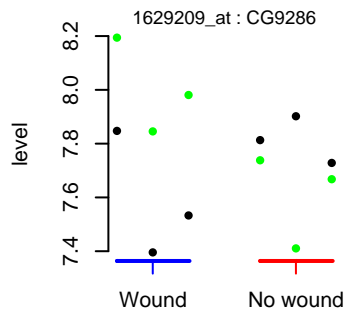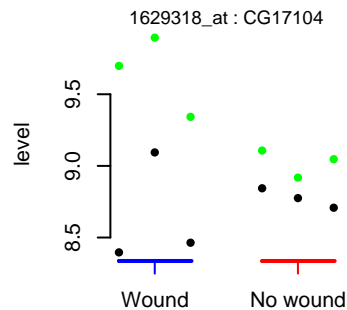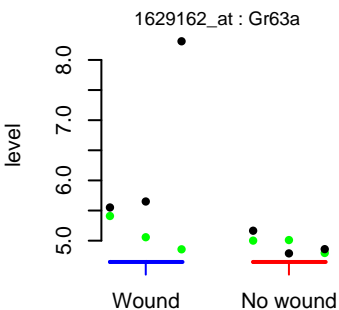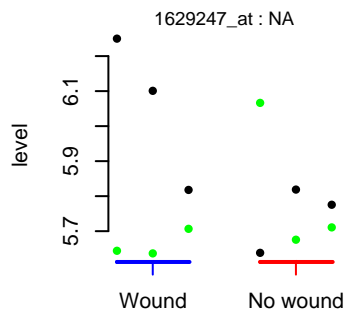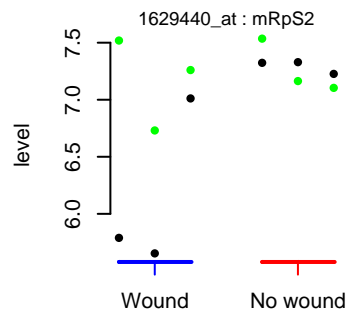

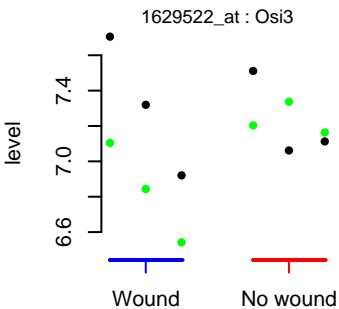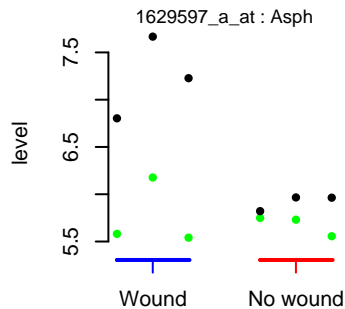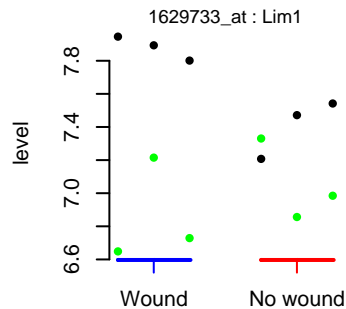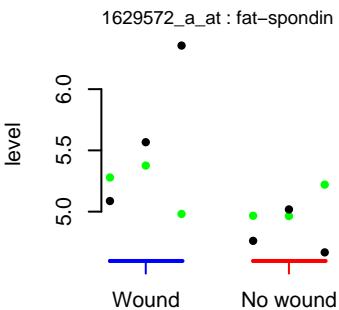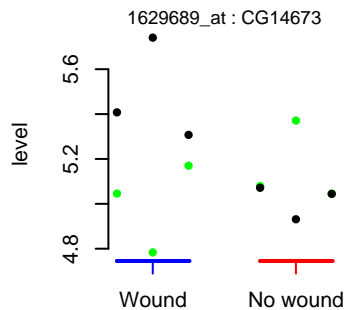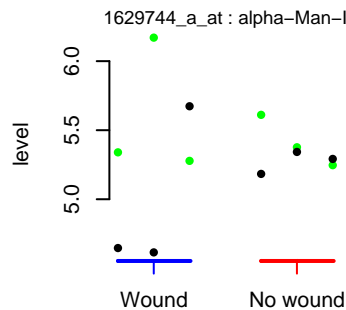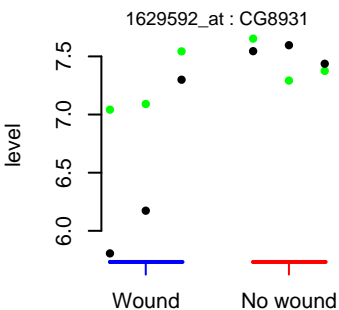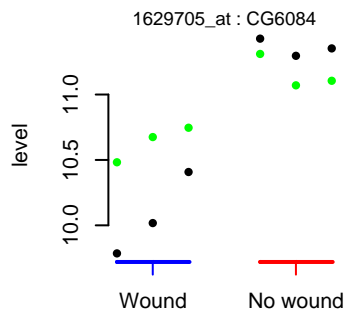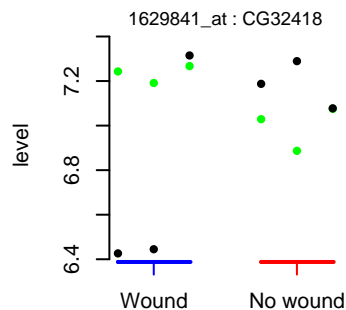

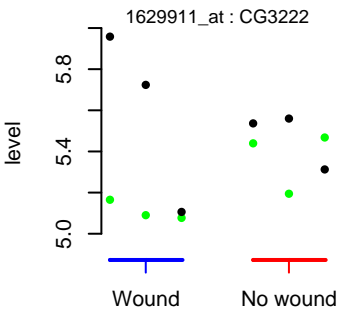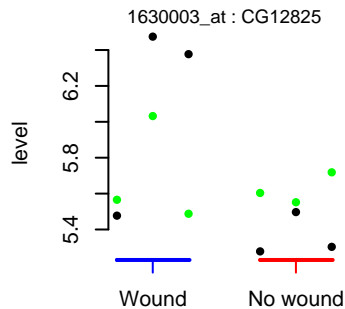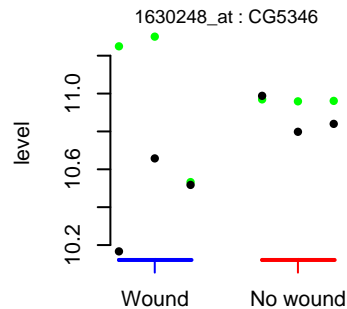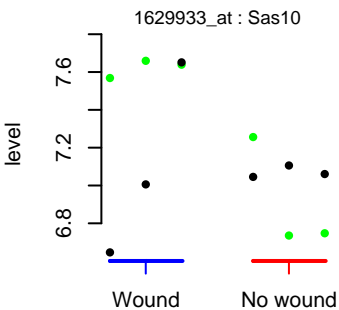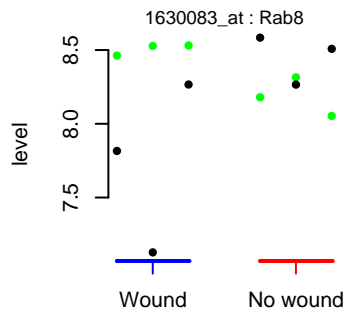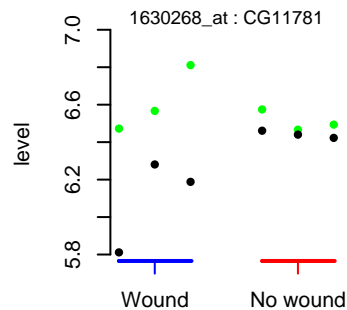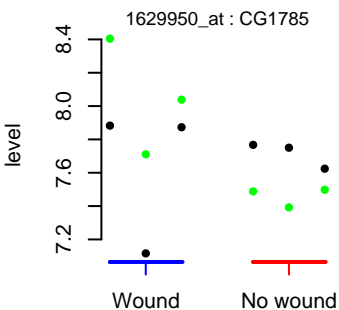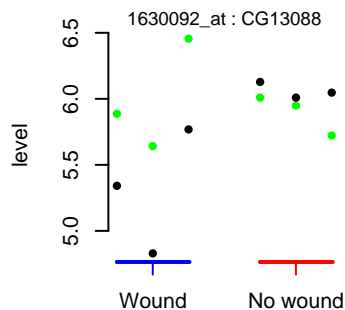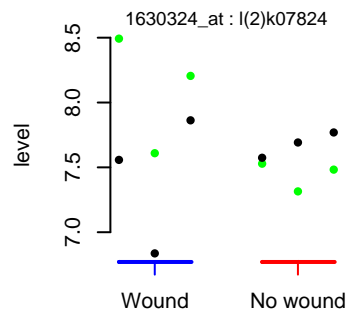

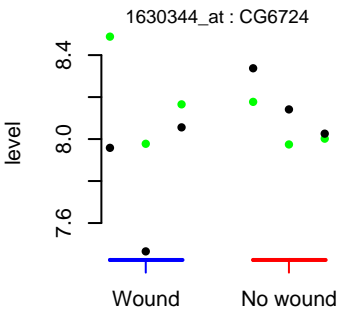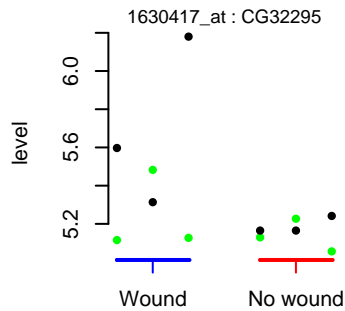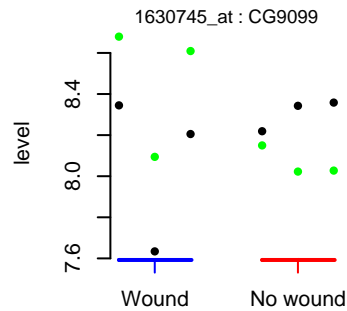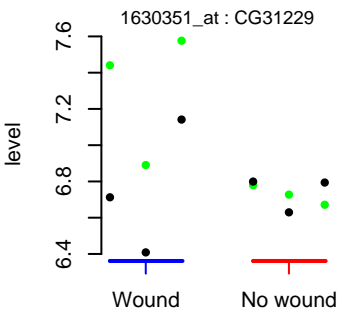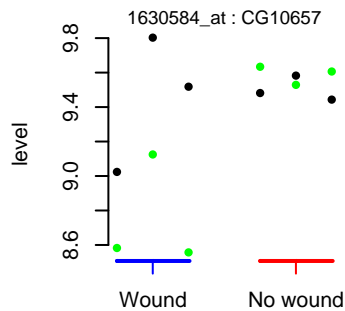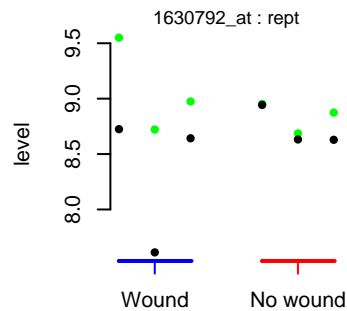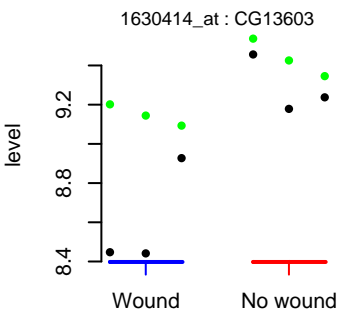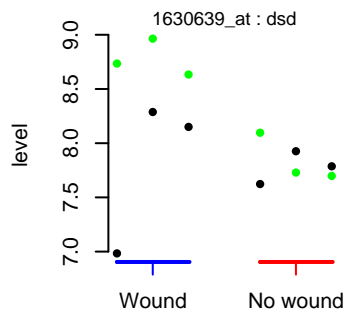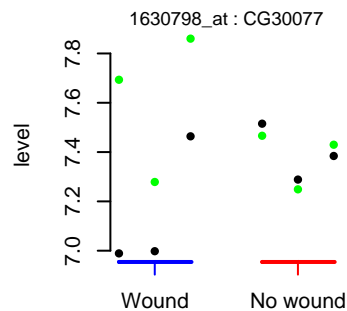

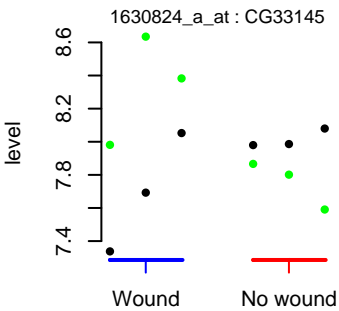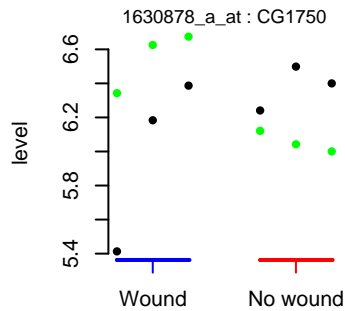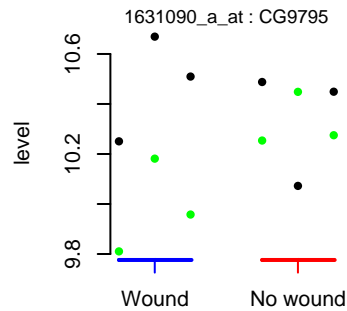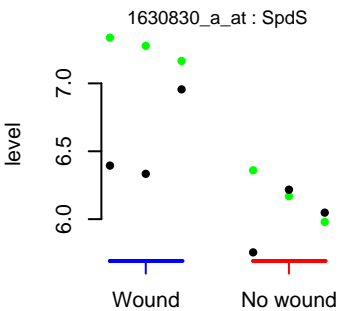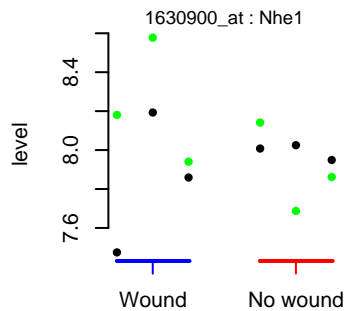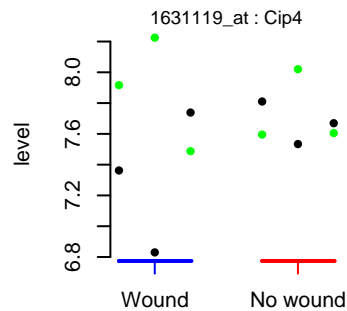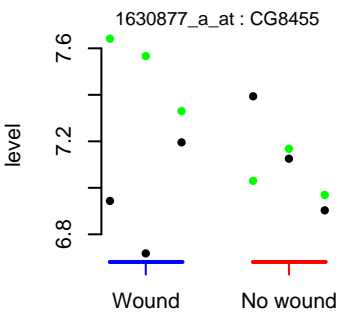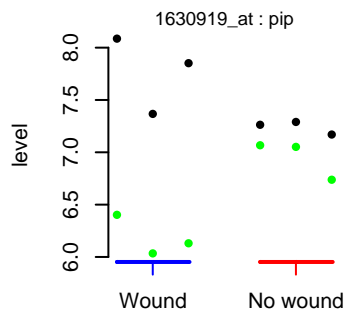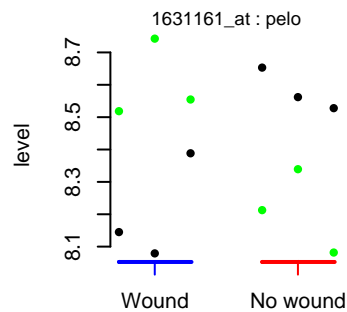

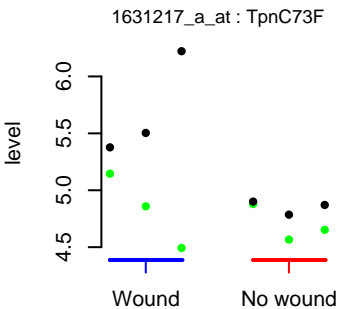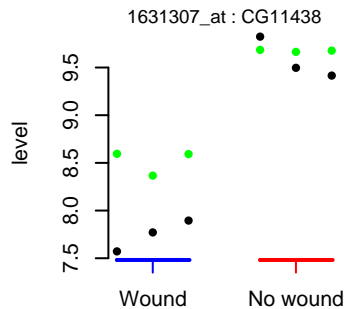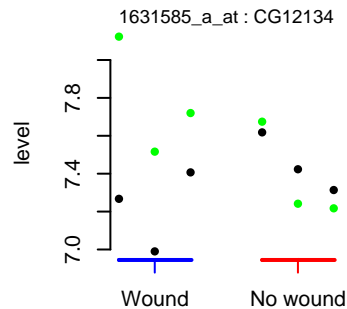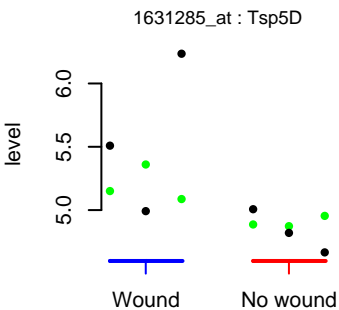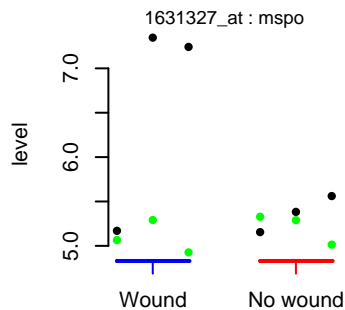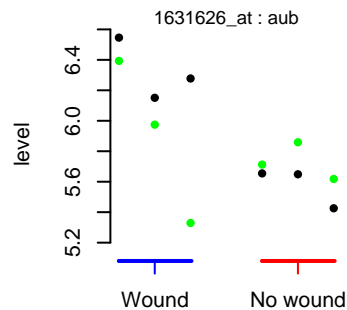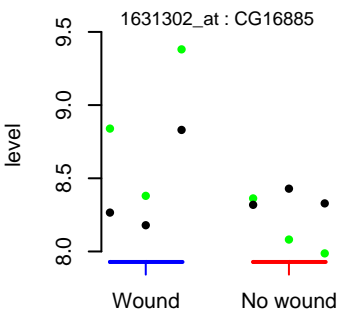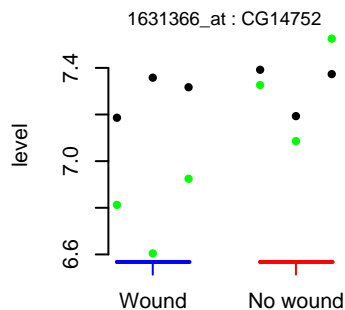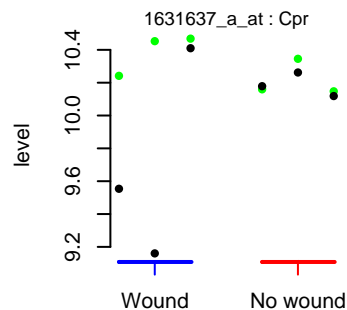

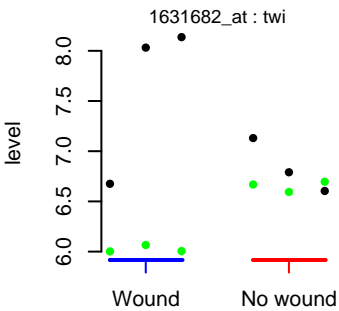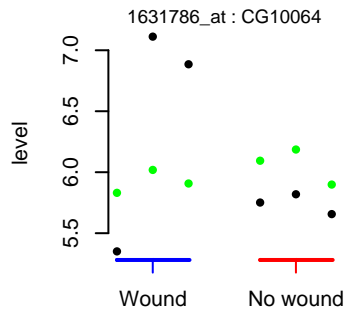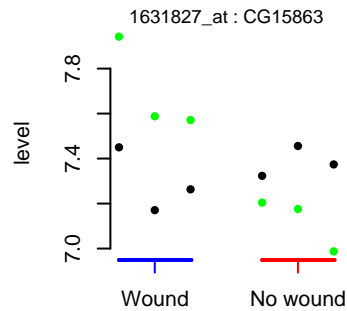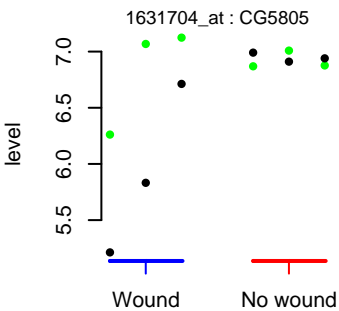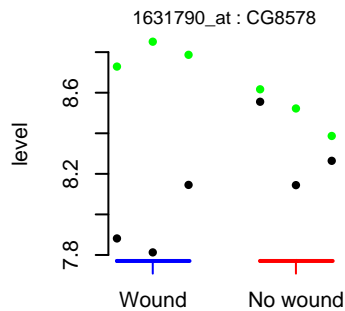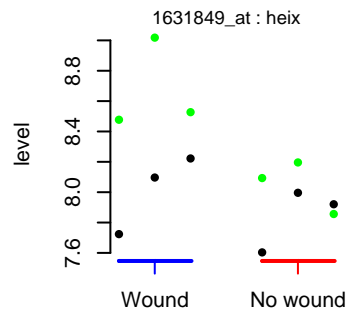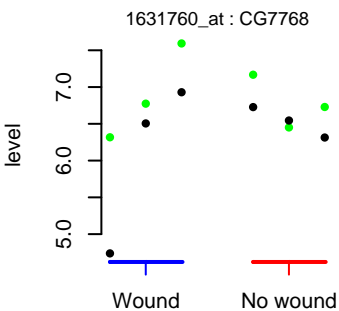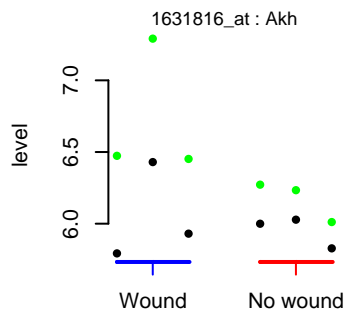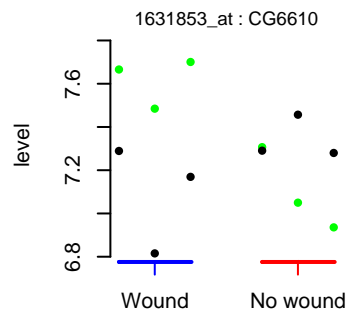

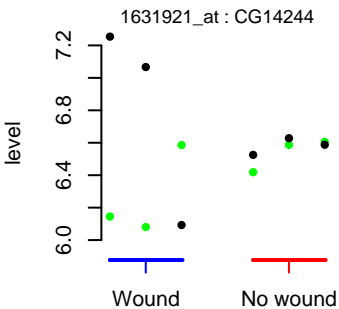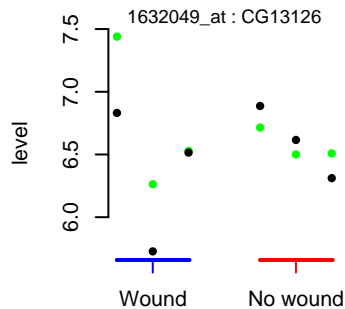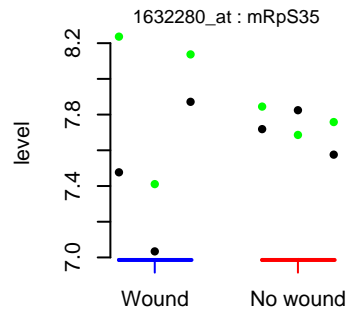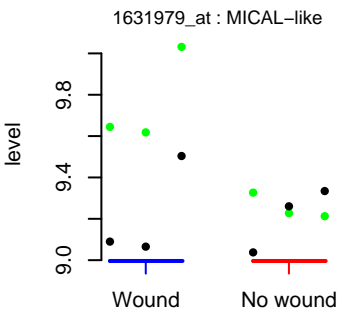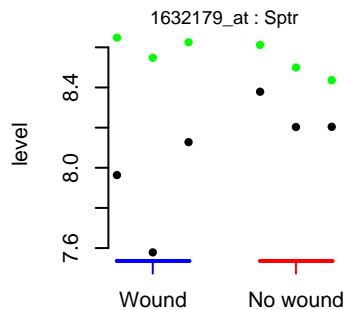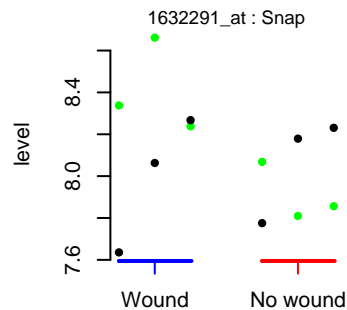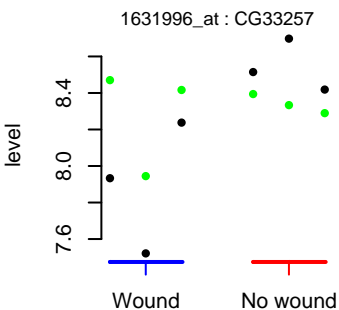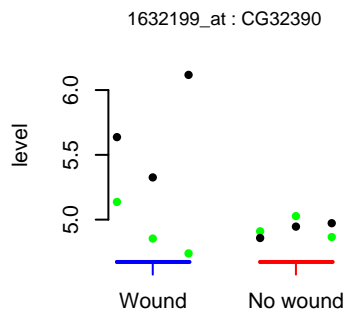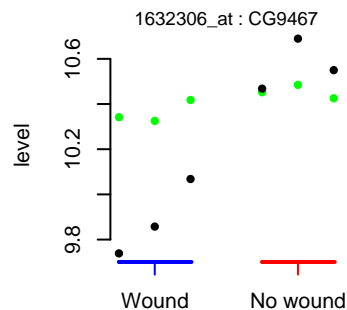

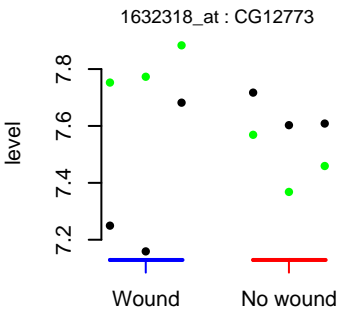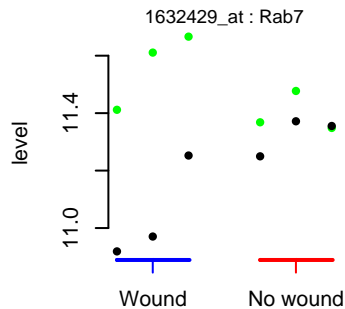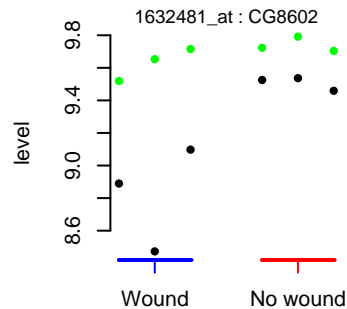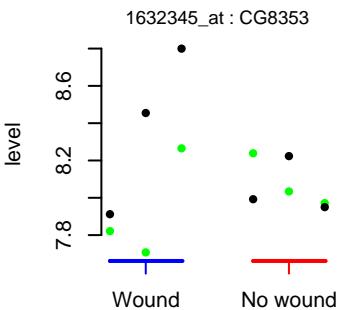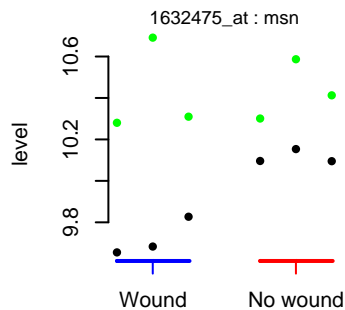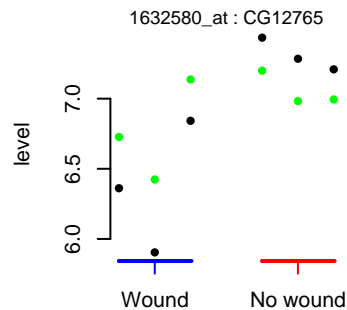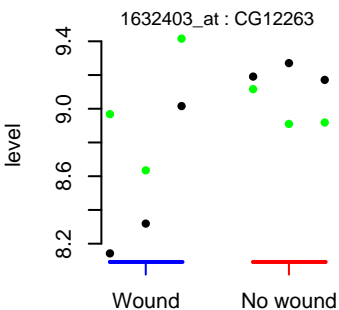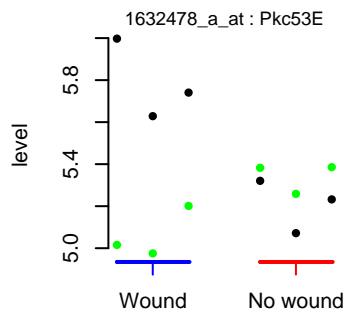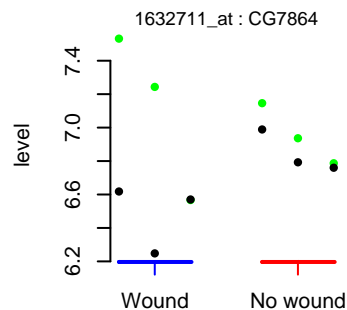

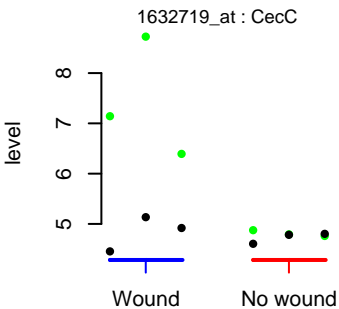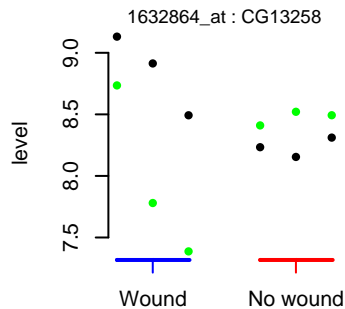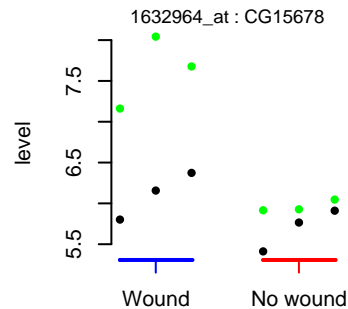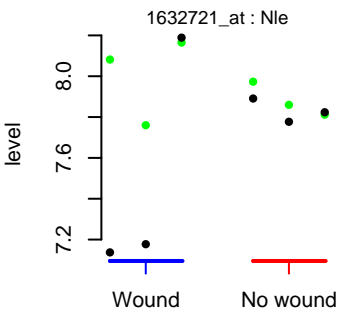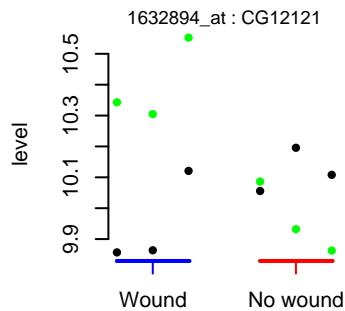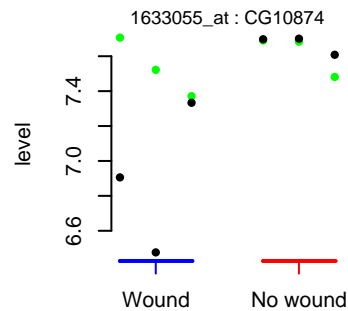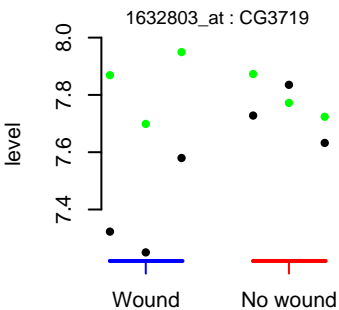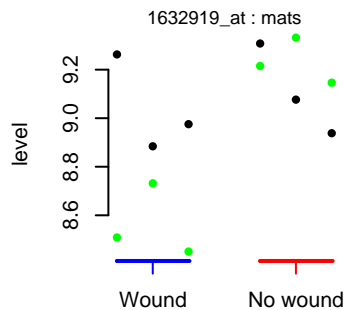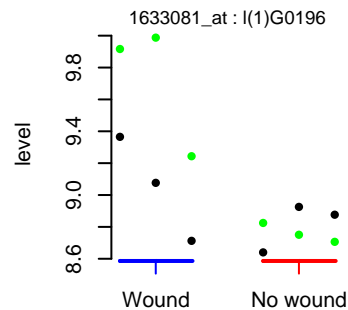

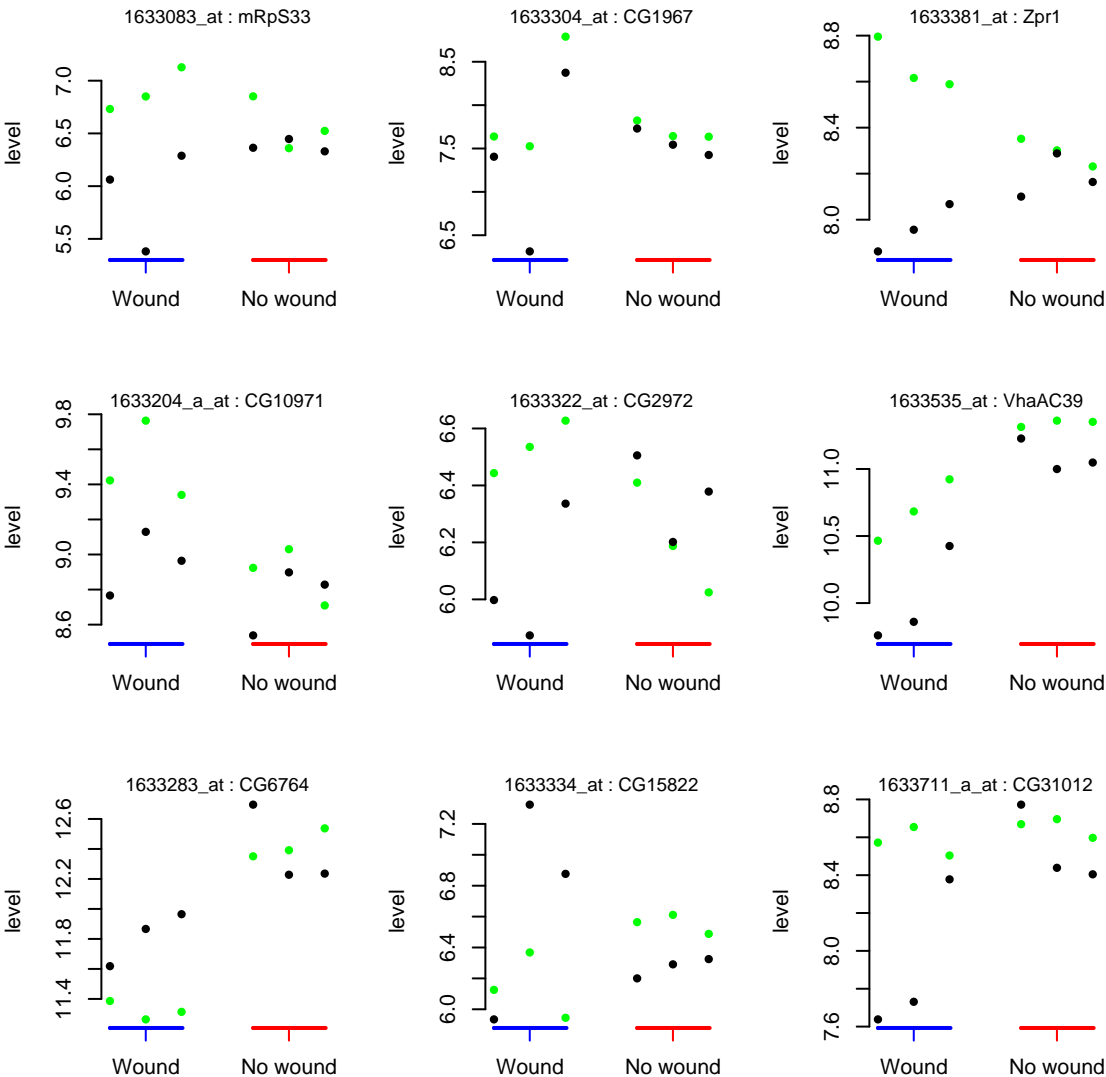

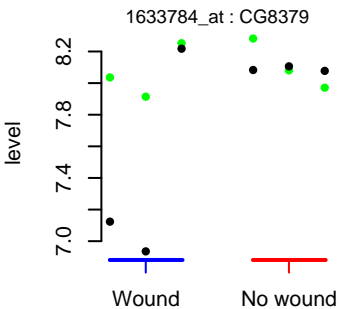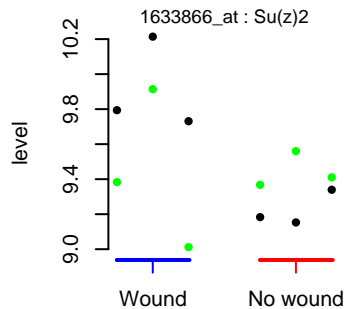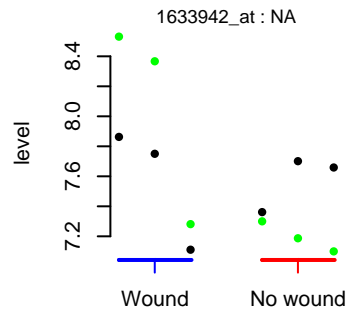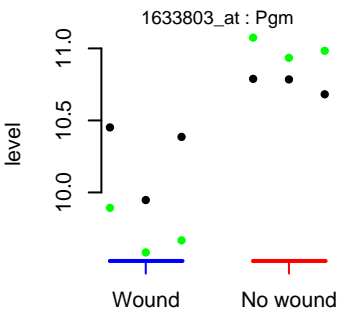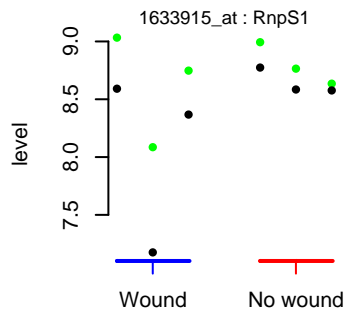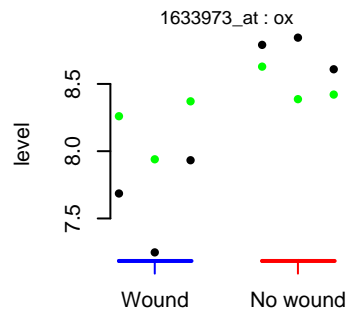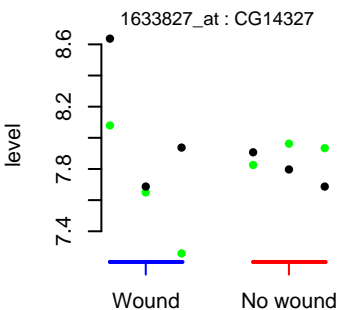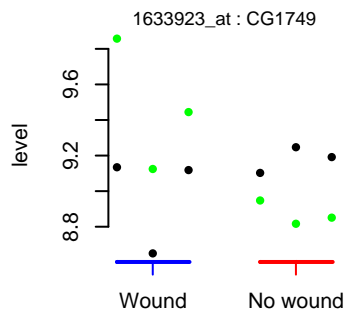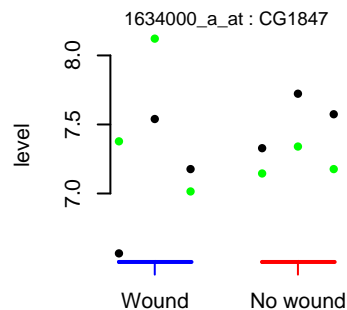



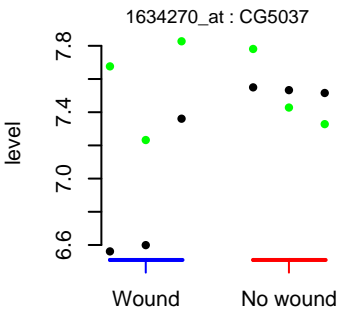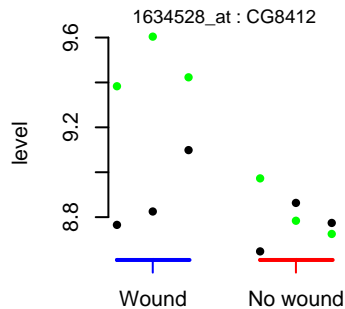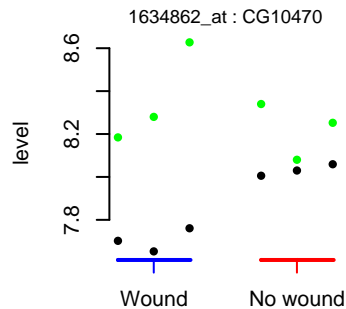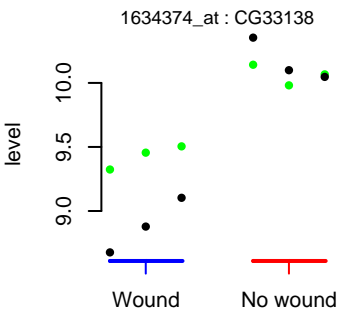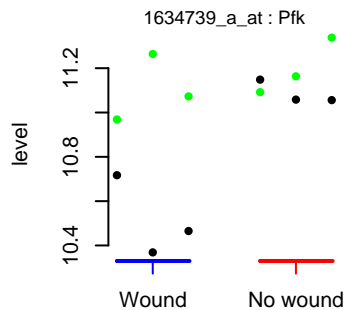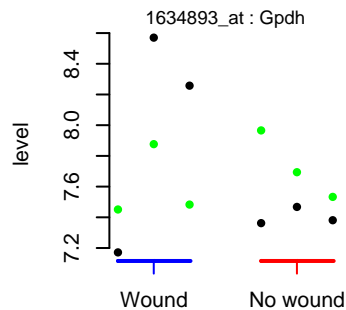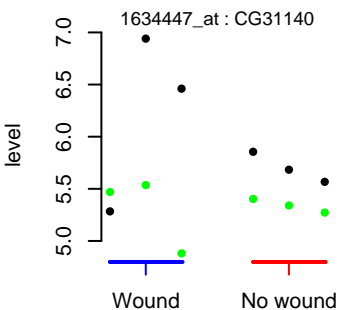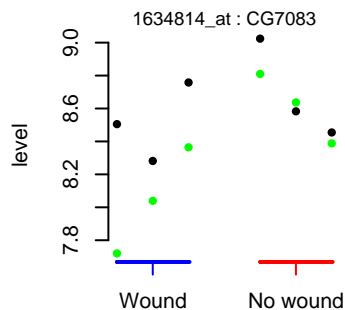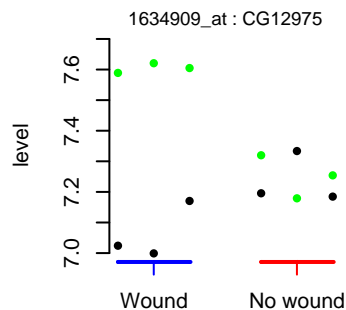

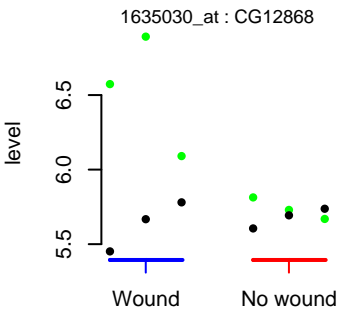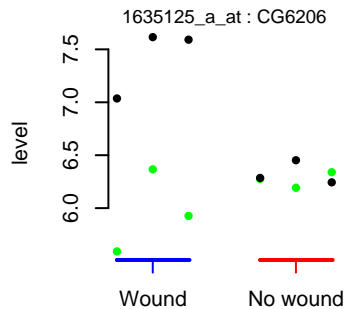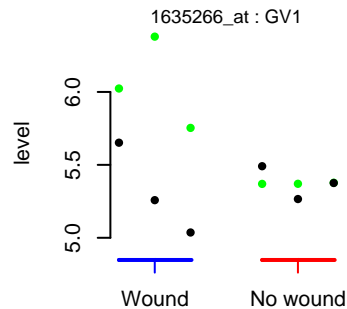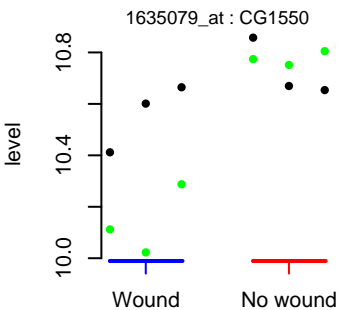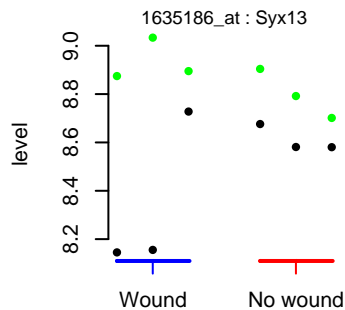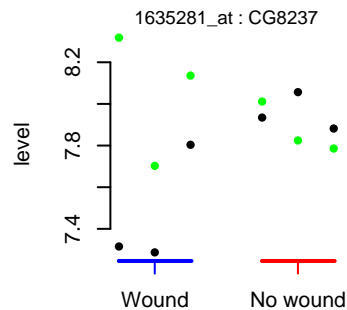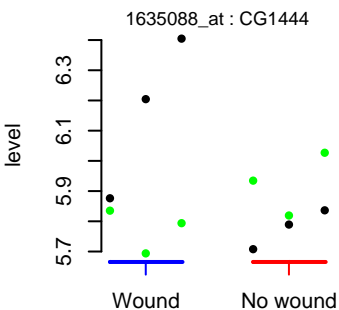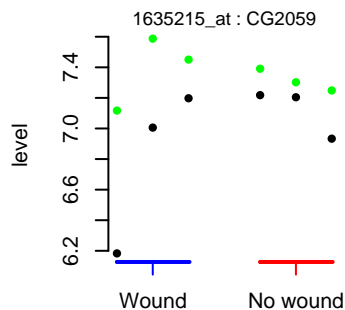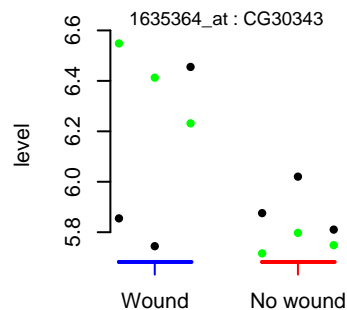

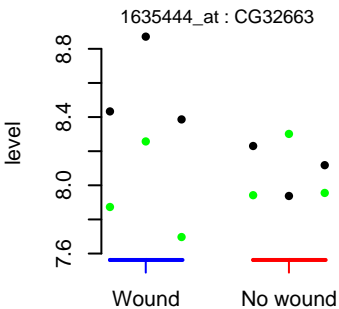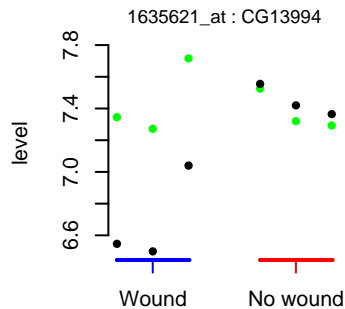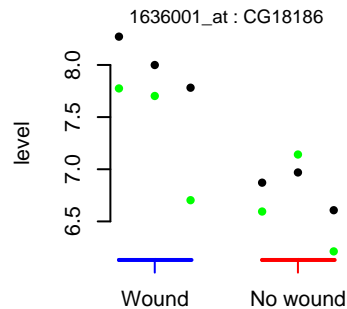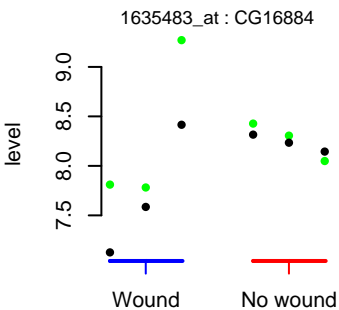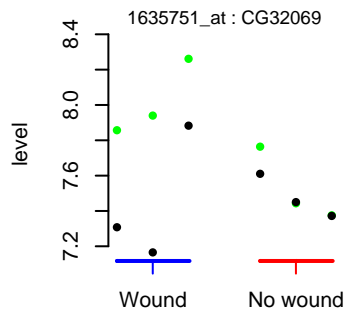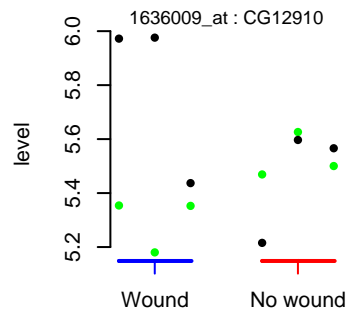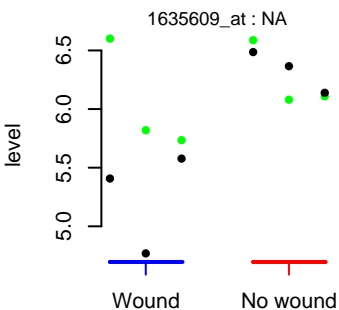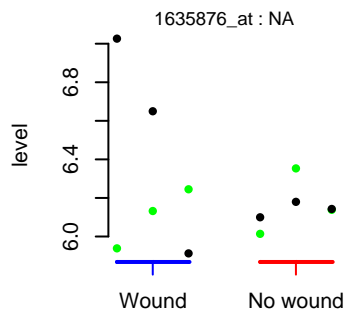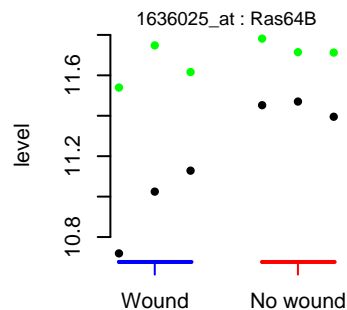

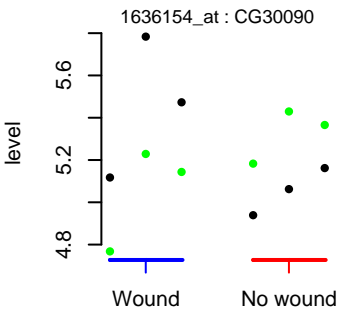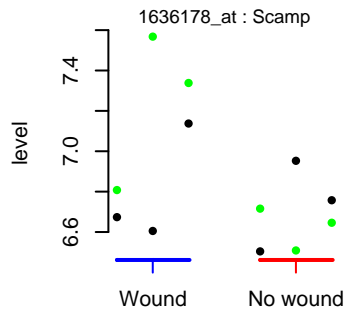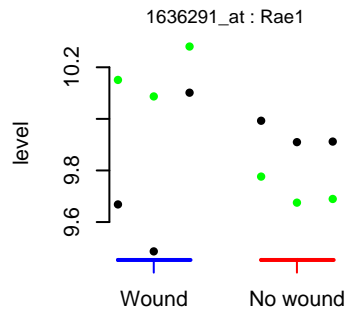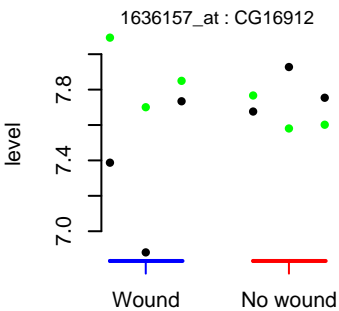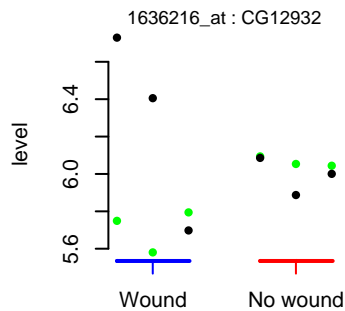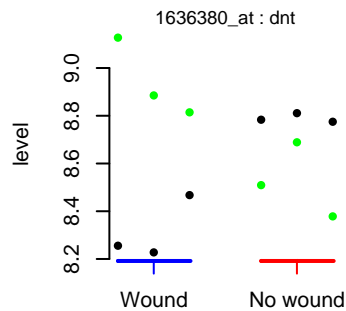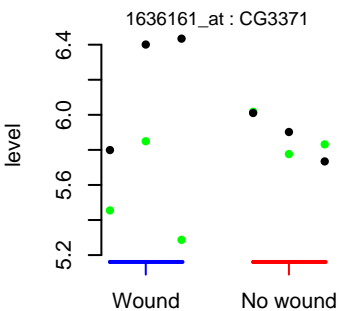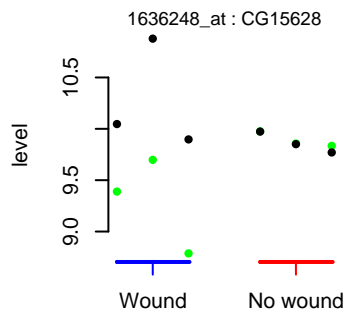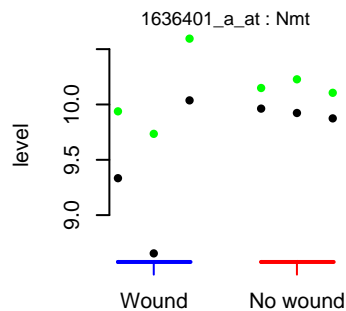

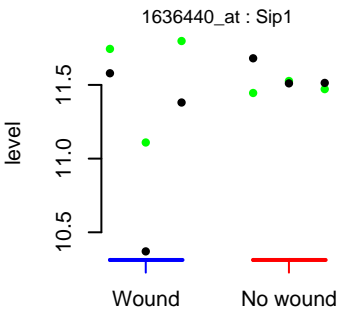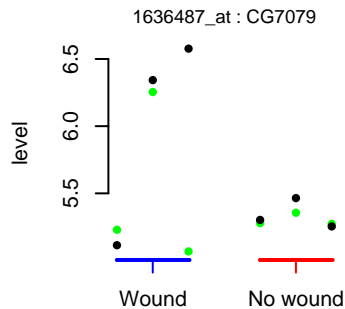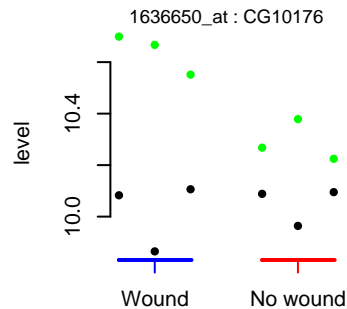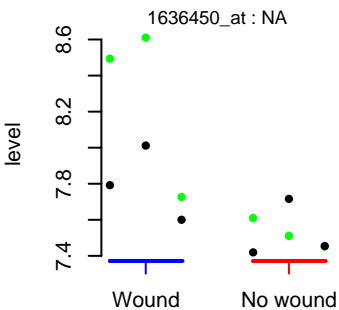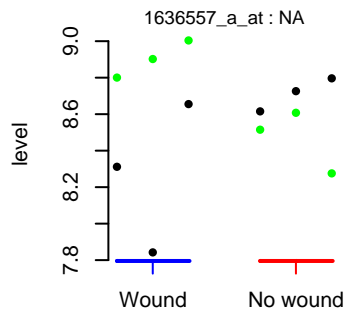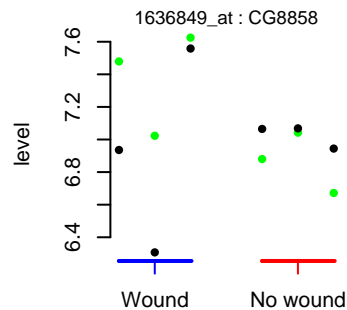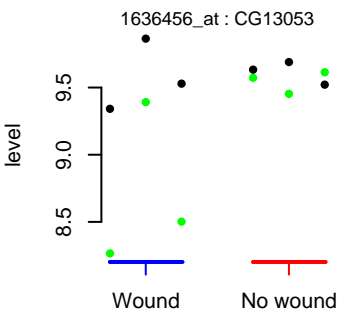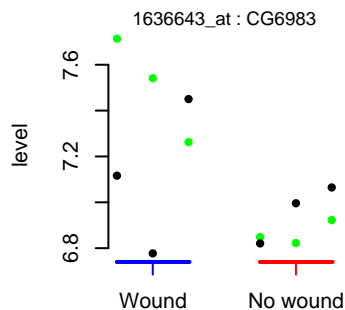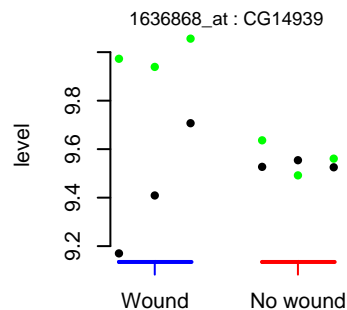

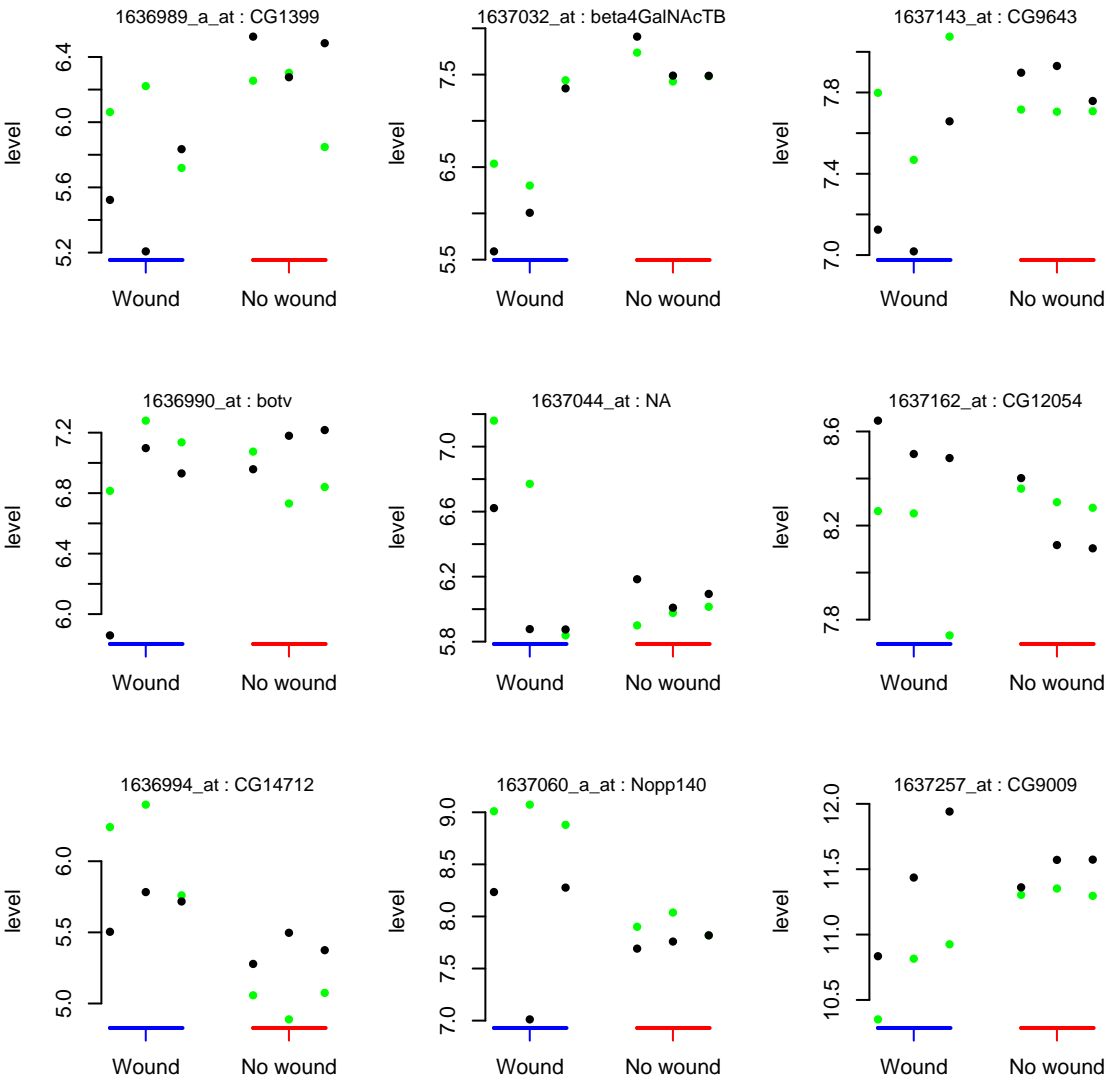

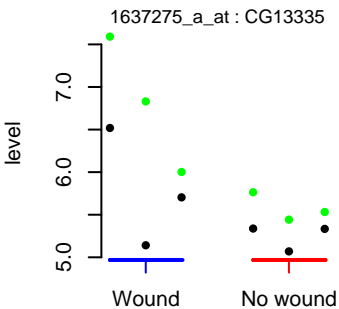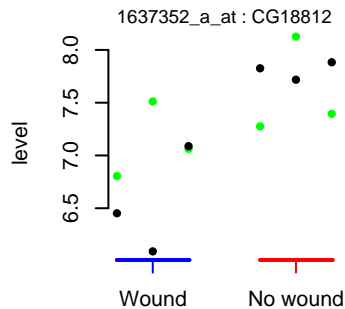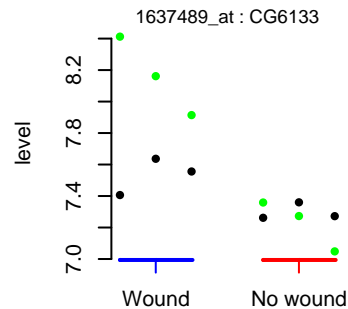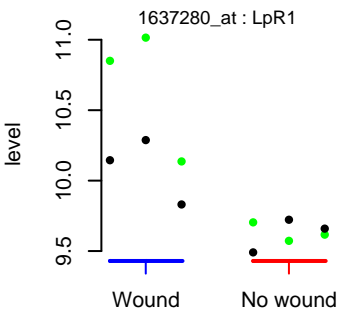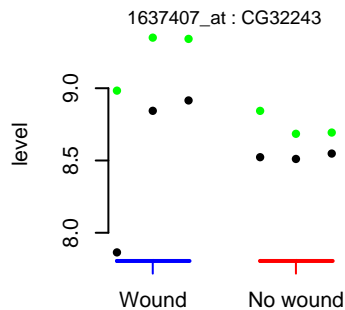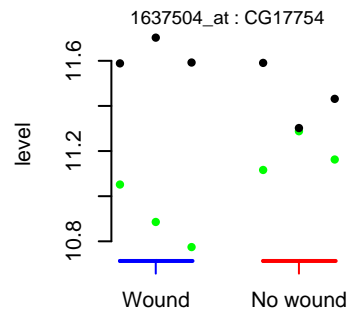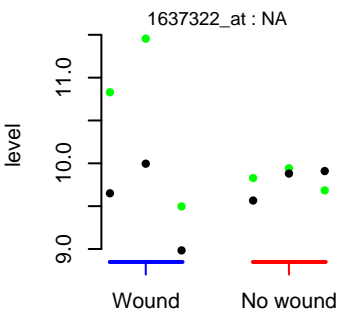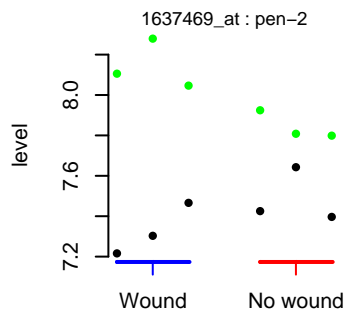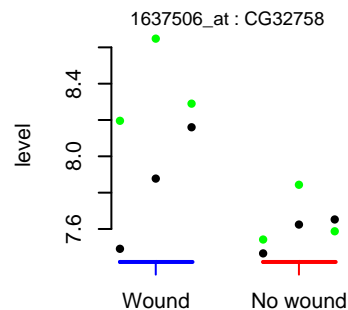



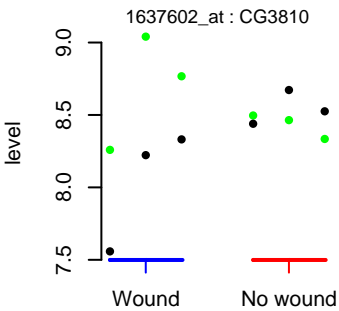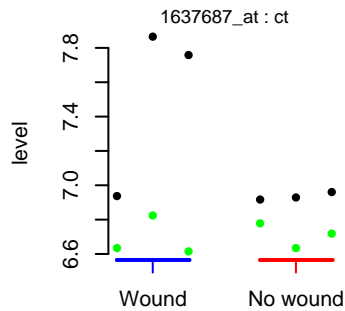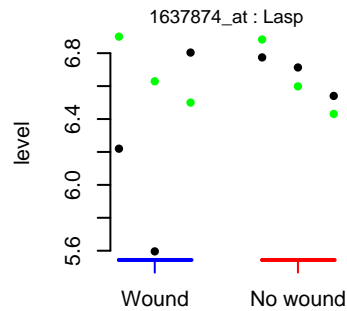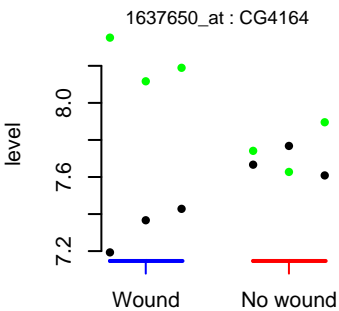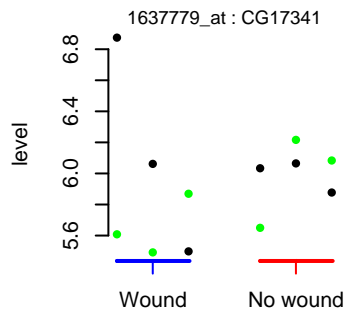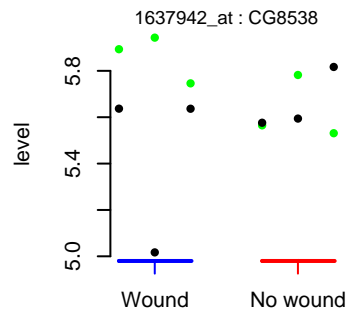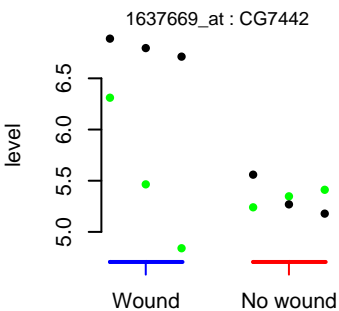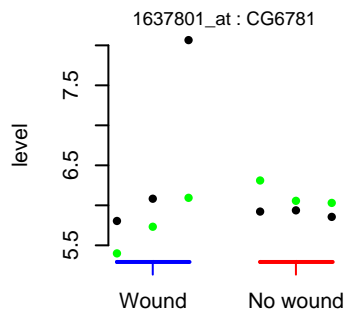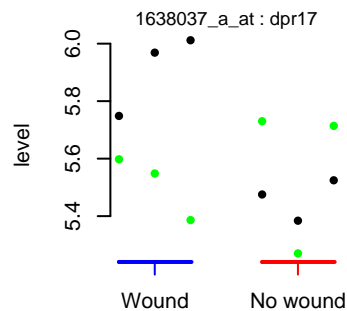

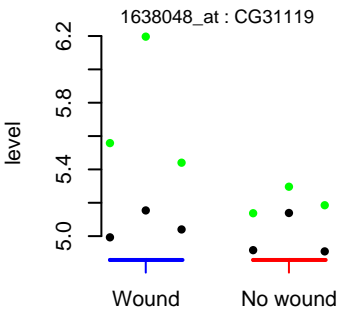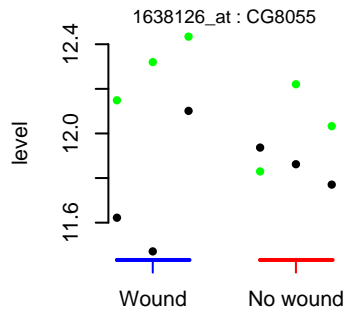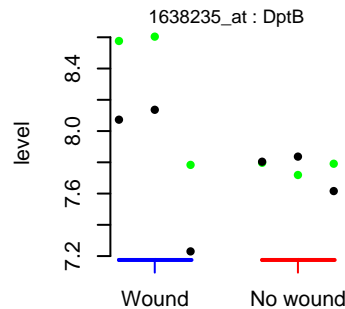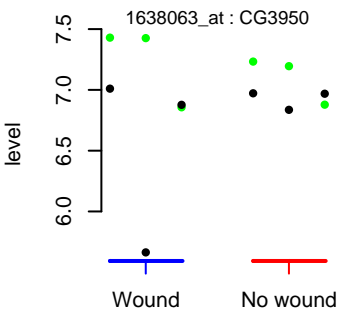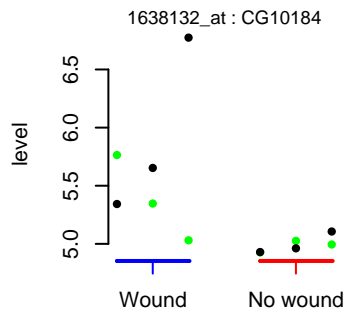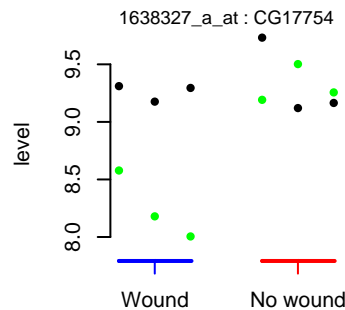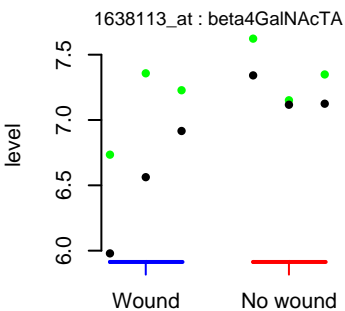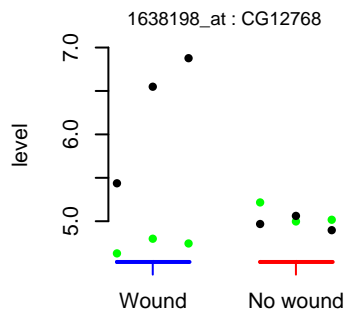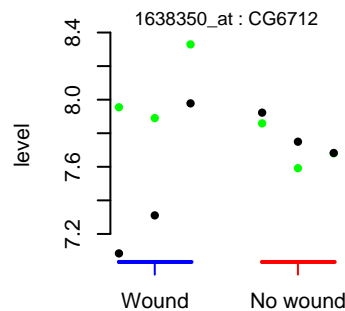

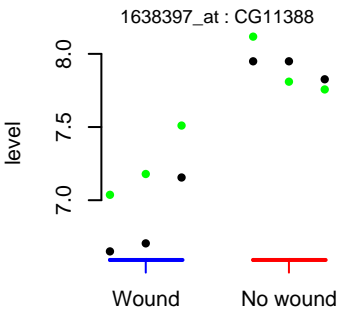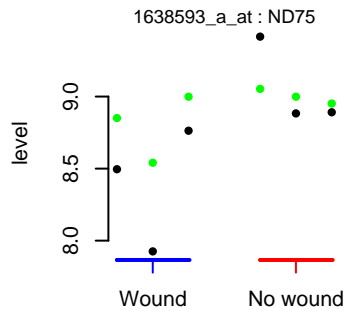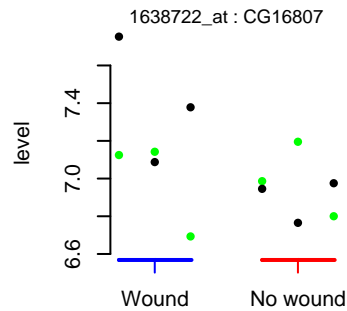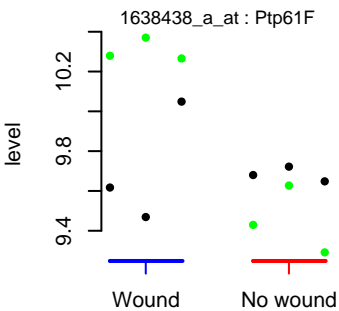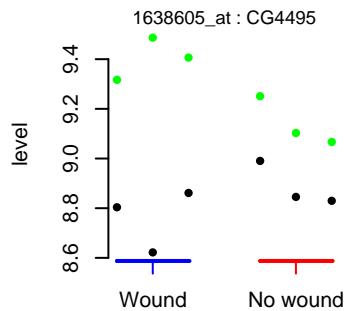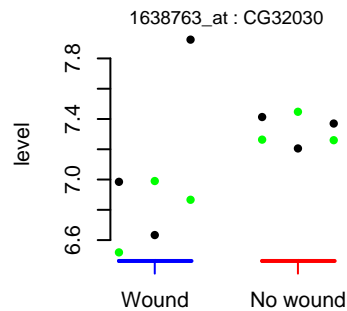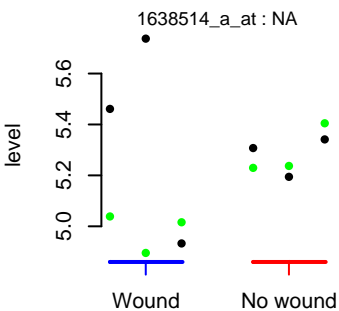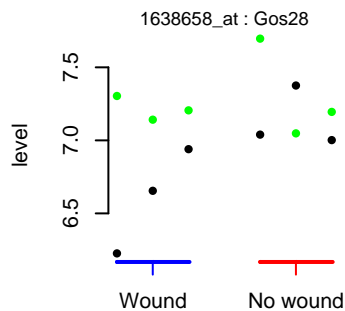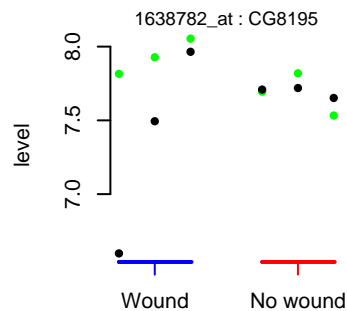

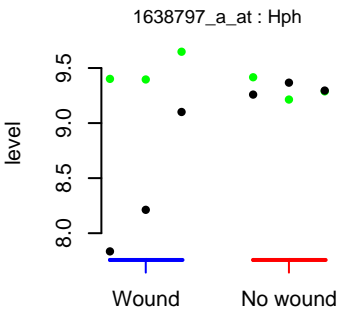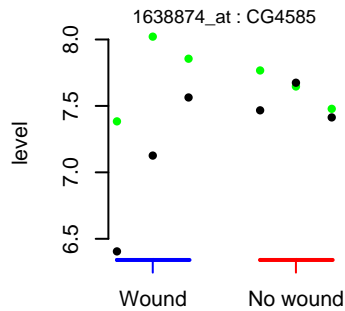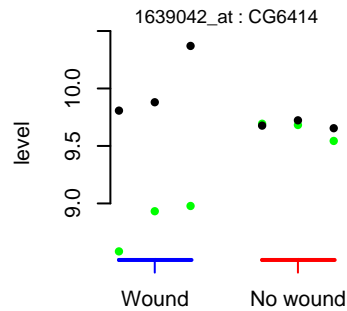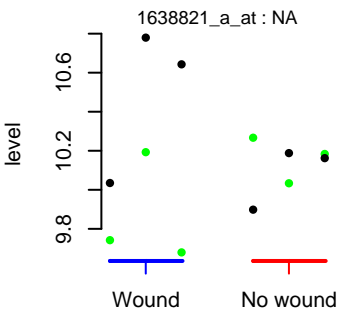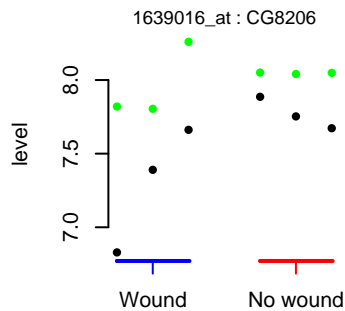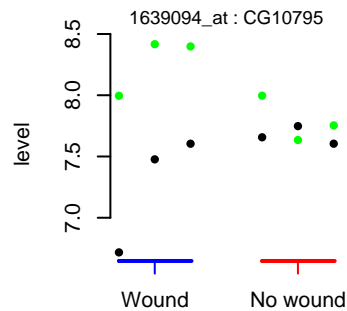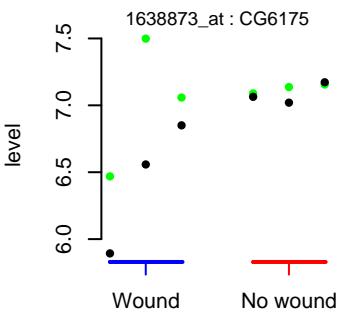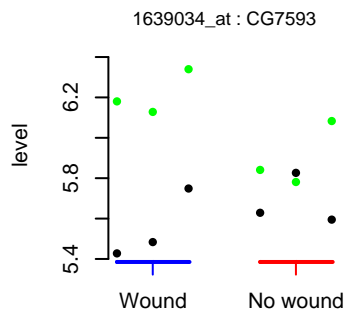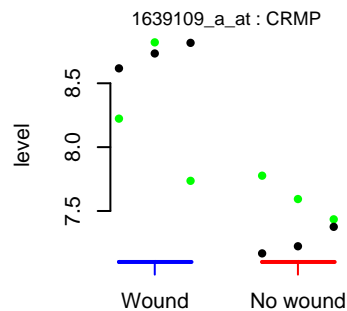

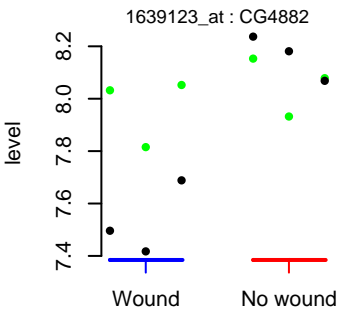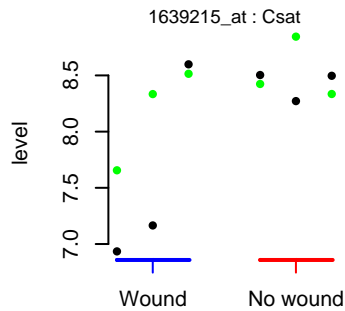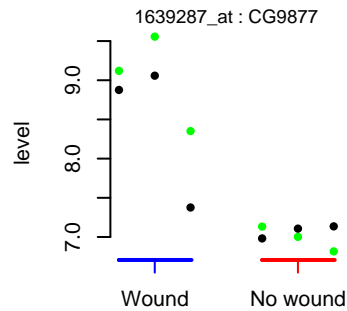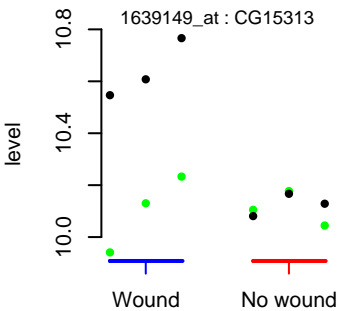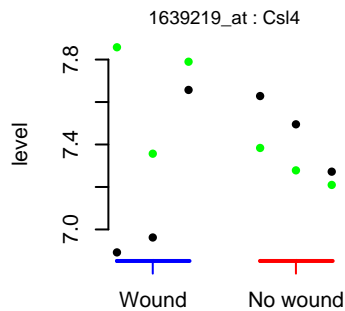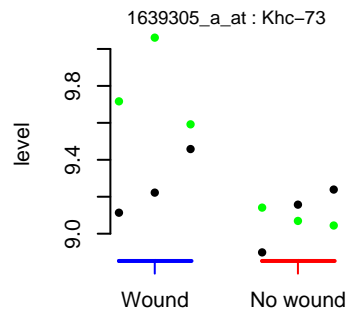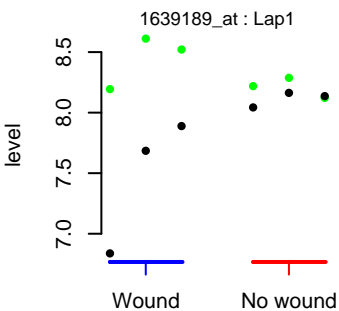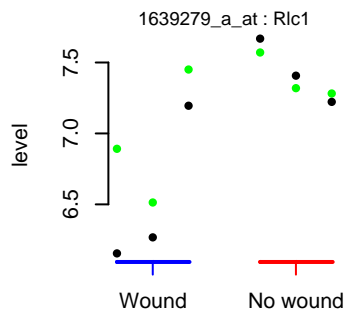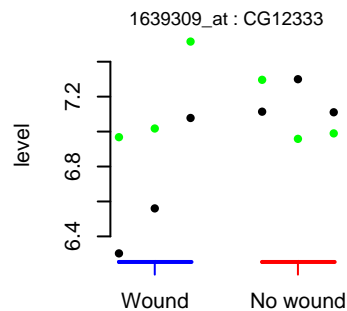

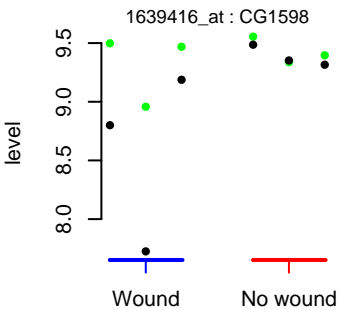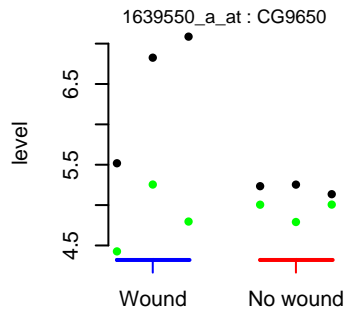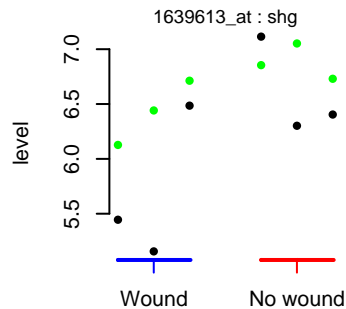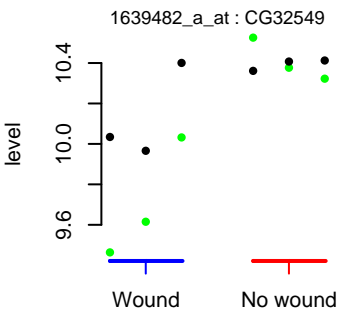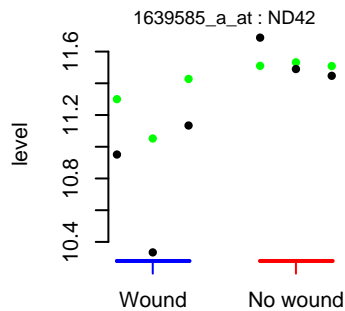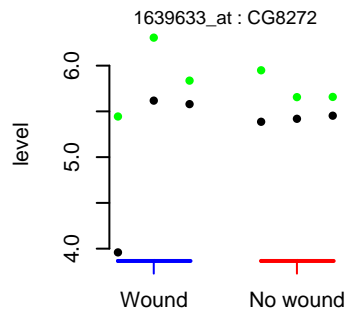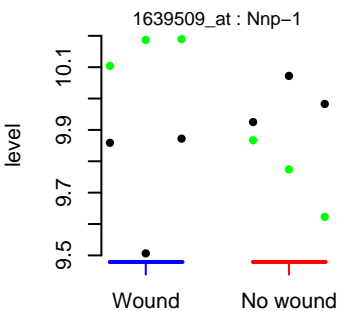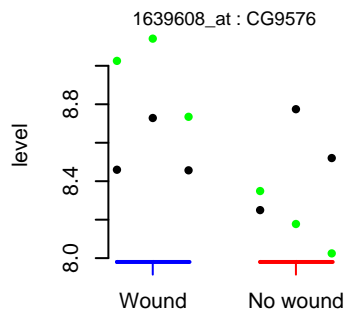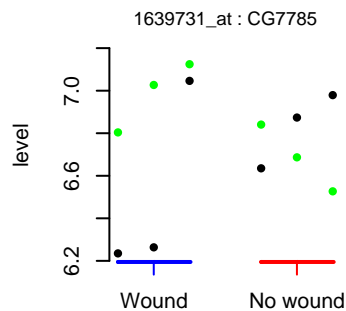

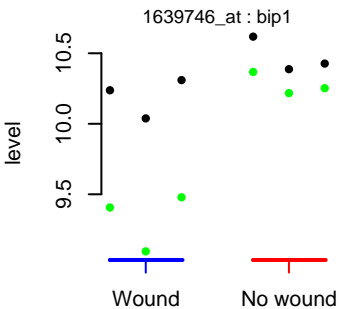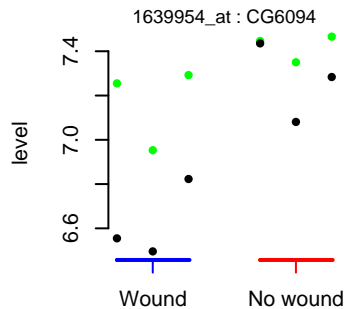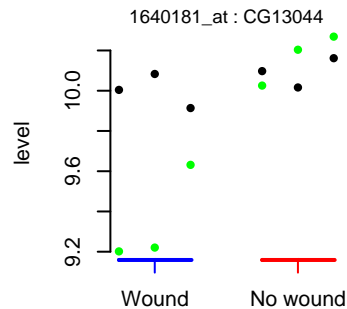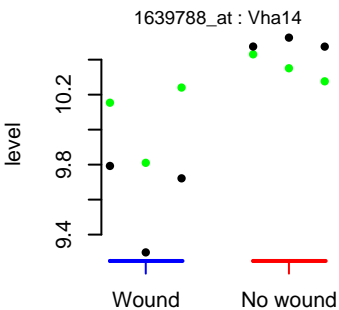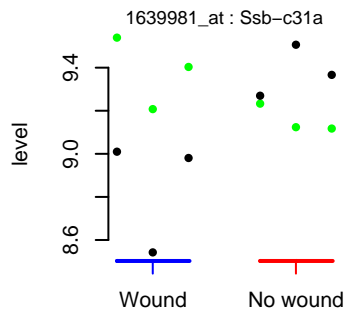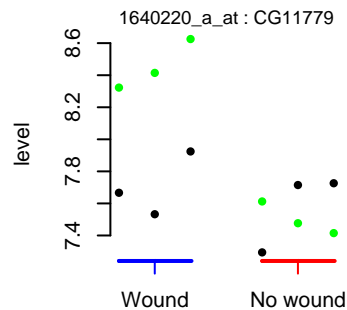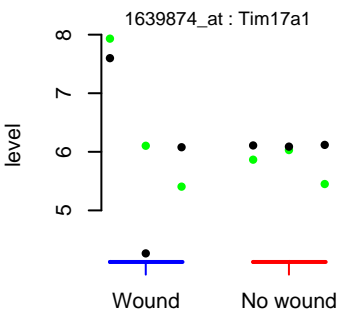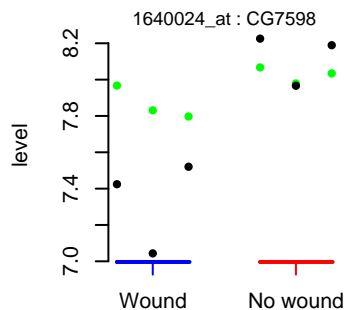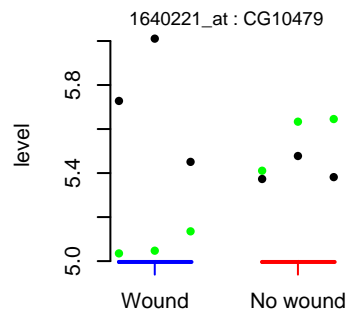

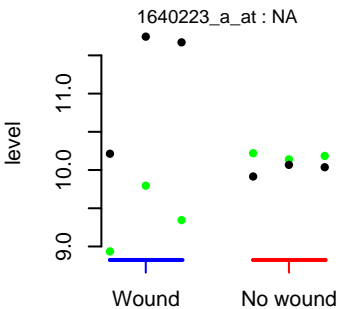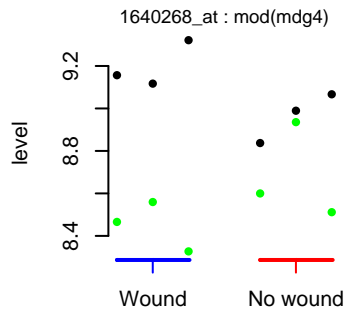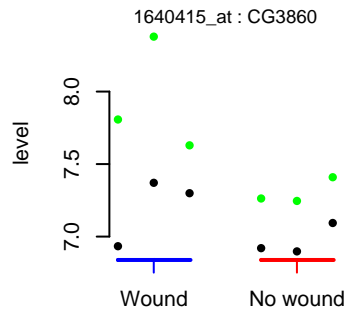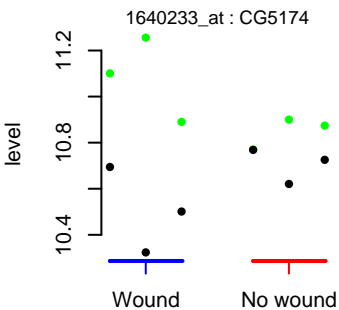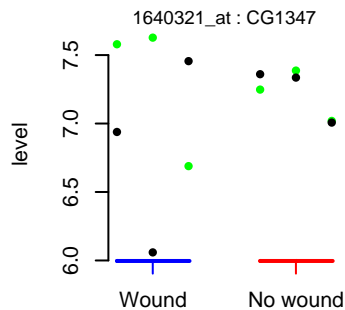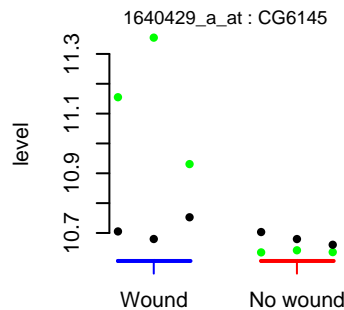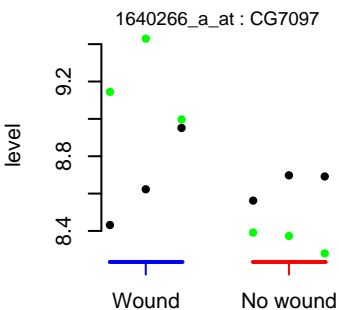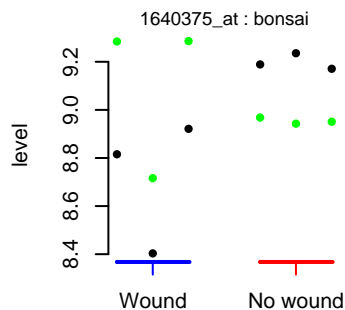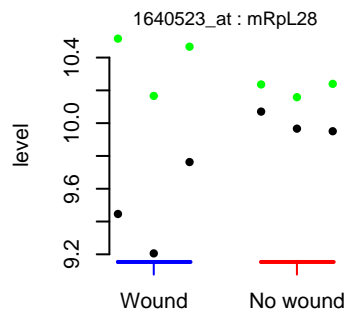

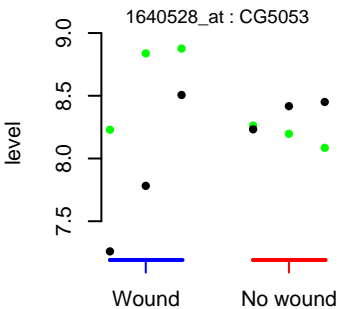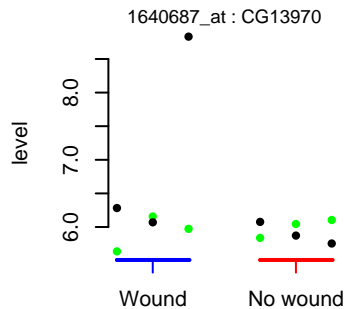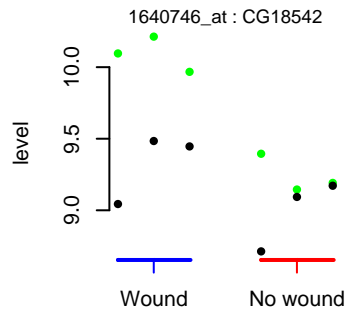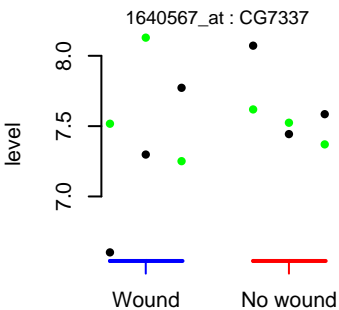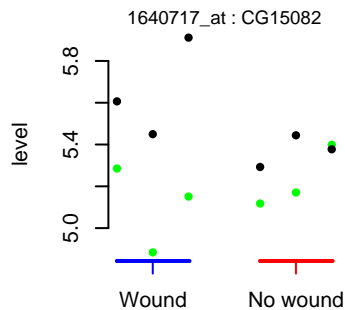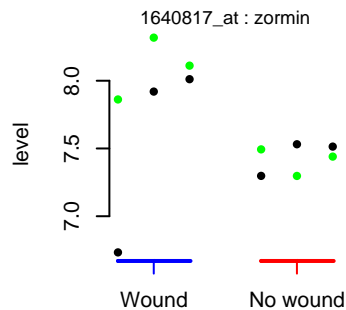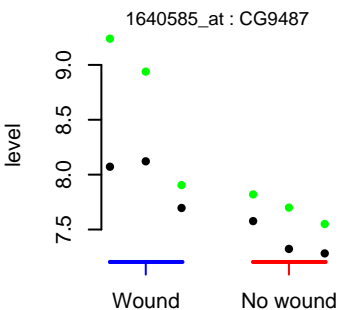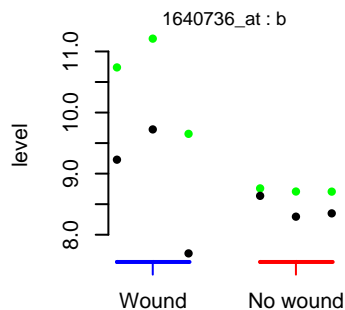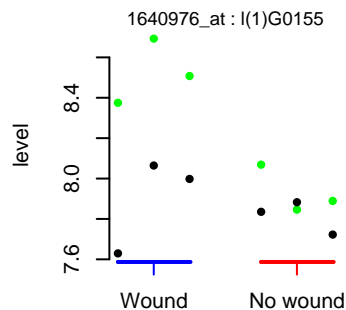

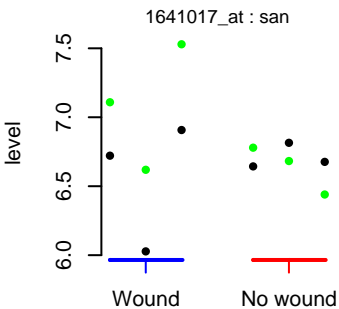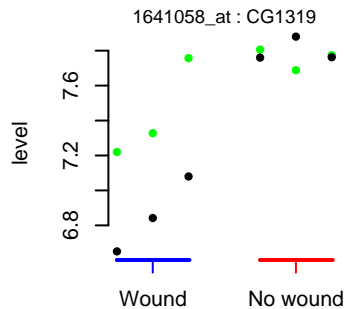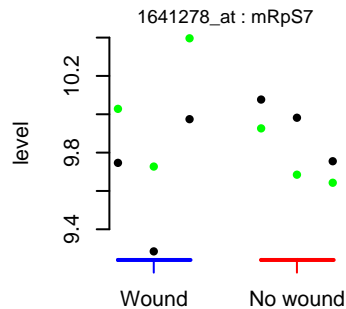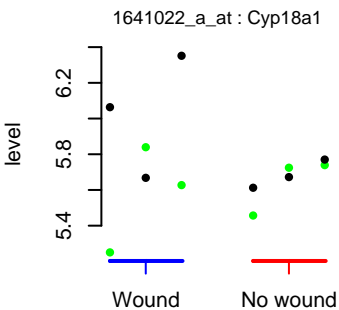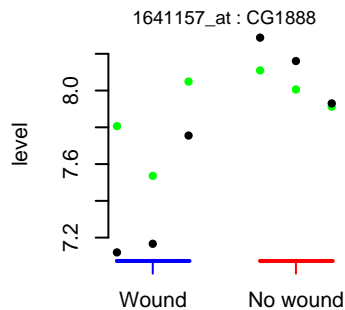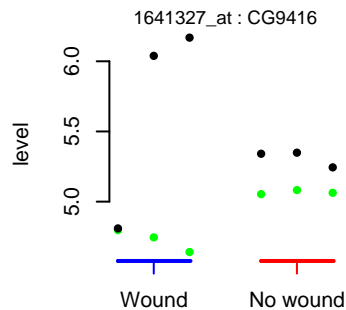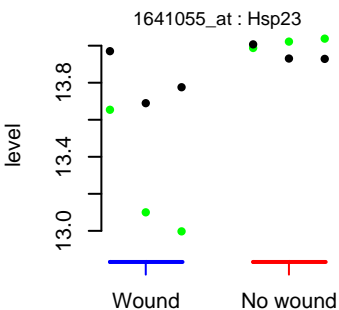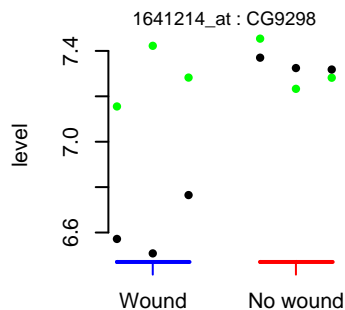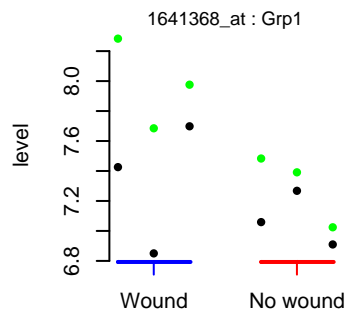

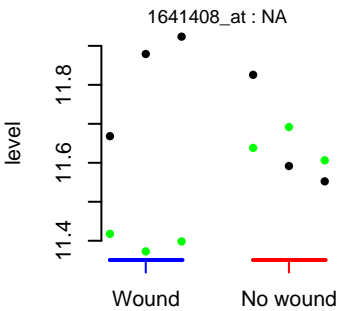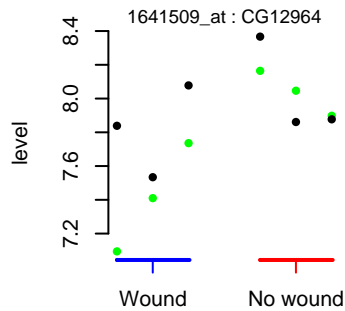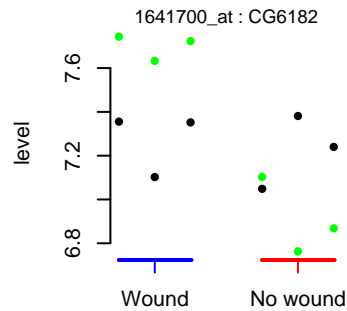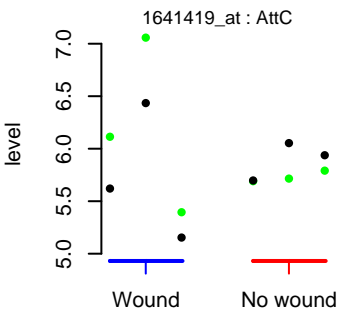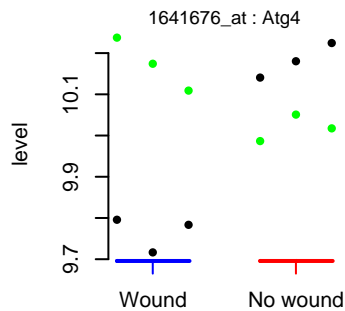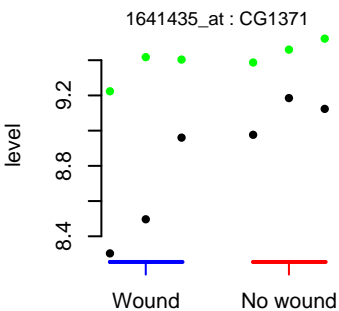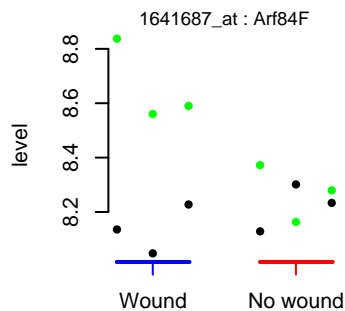

Supplement: S2 Fig — Overall changes in expression in wounded discs (for JNK-positive, negative or both cell types) relative to controls for individual genes in the WO subpopulation (genes differentially expressed in wounded discs only). Green spots show the level of expression for each replica in JNK-positive cells. Black spots show the levels of expression for each replica in JNK-negative cells. (PDF) [file pgen.1004965.s003.pdf]

## **Supplementary Figure 3**

W/NW/D Gene Set



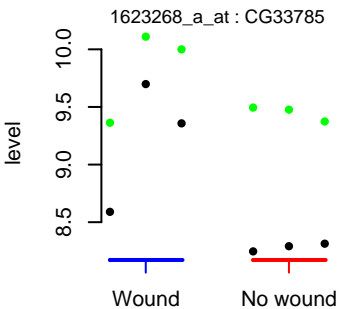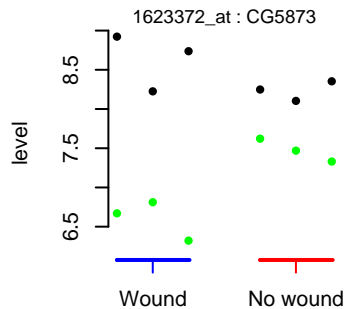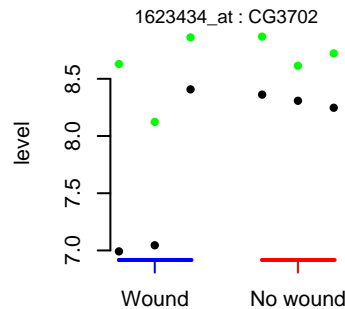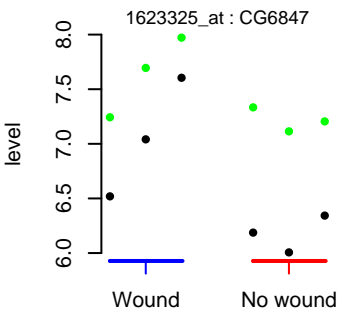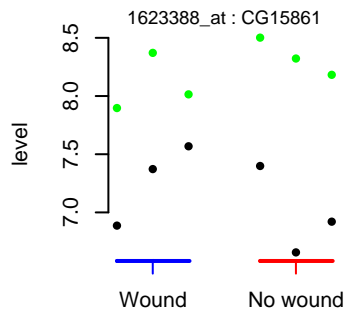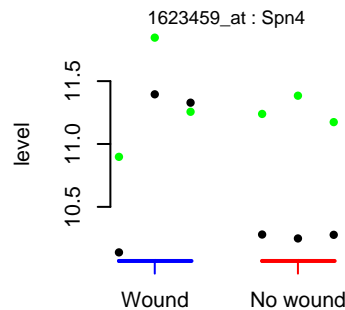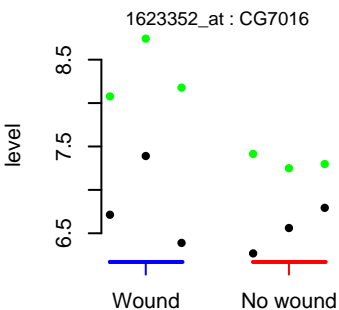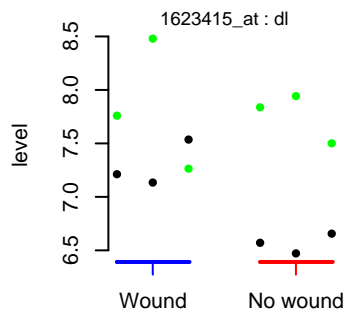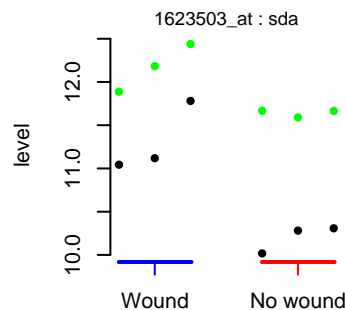

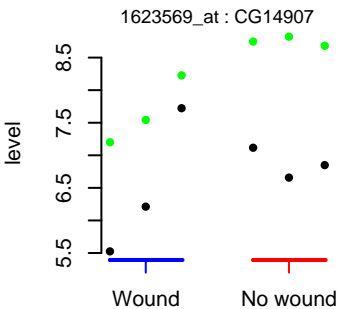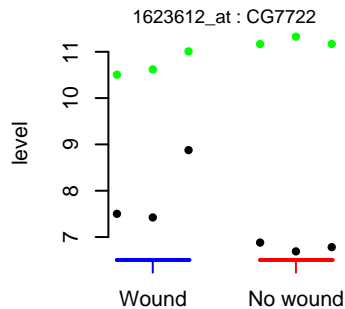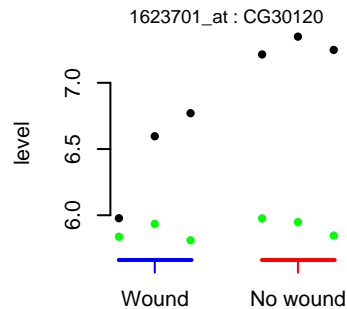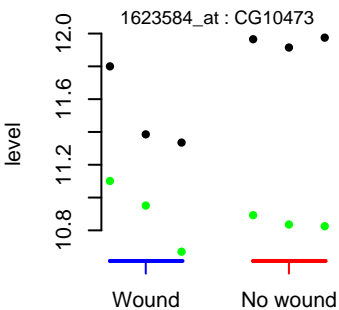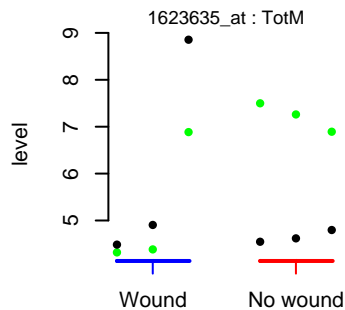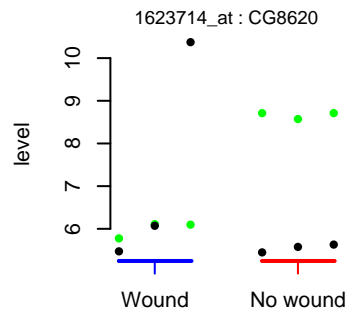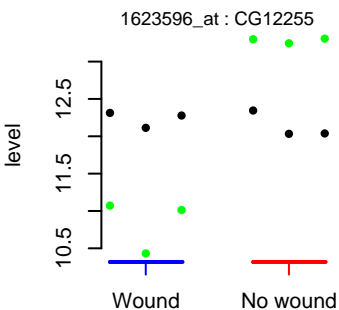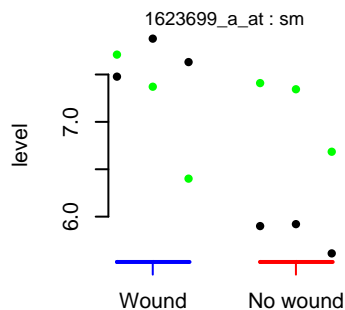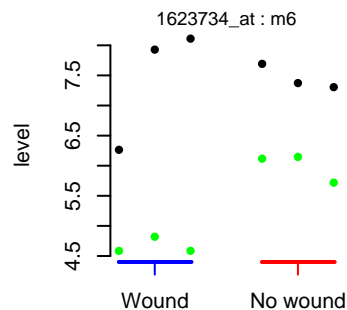

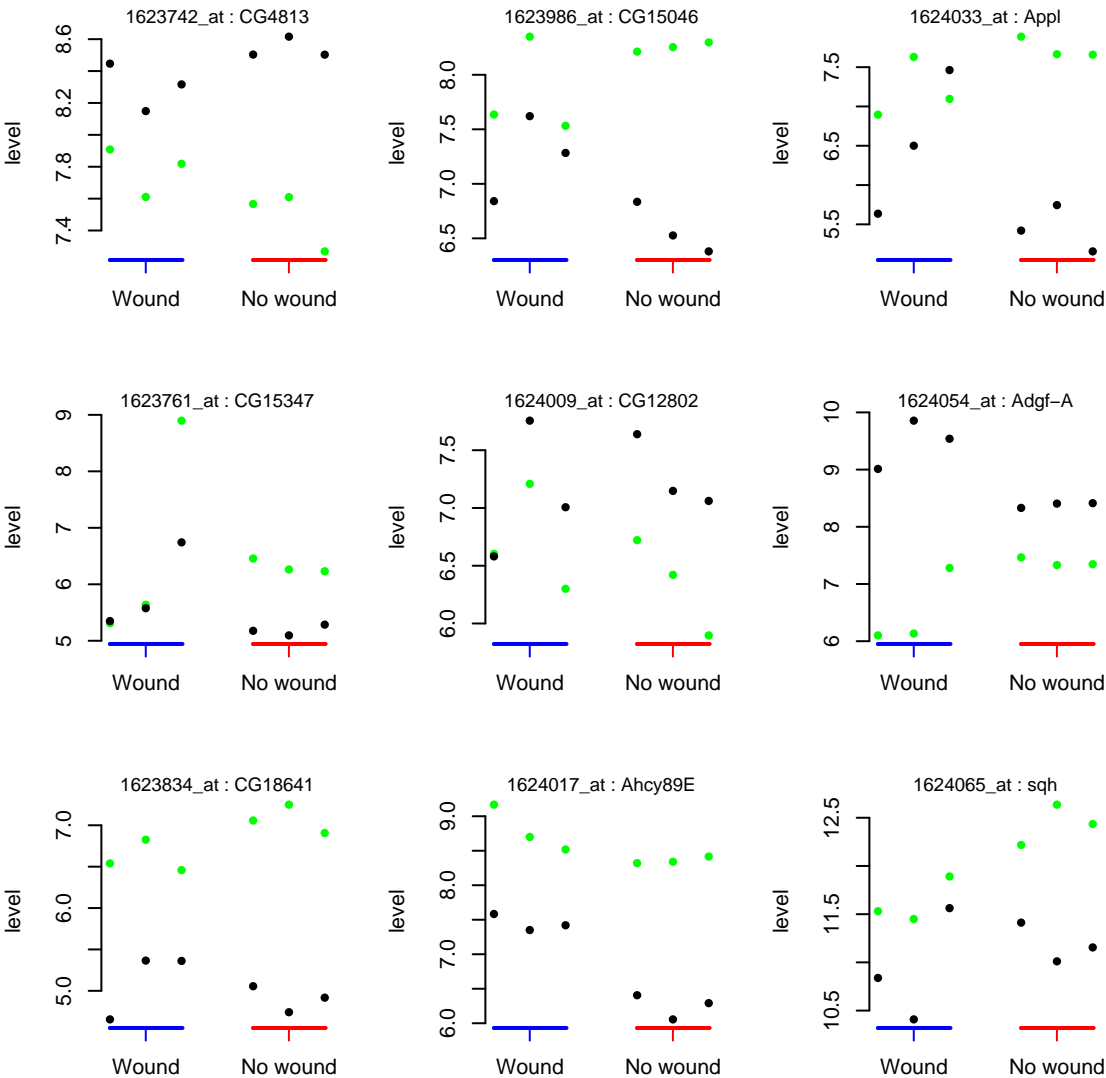

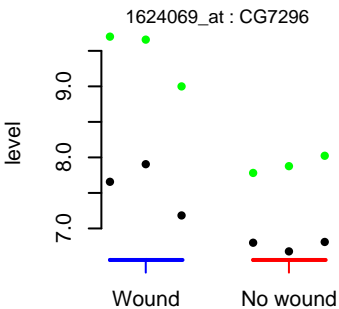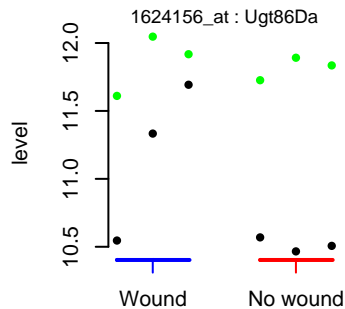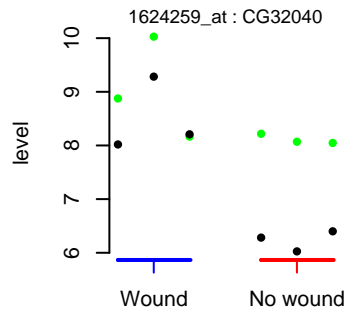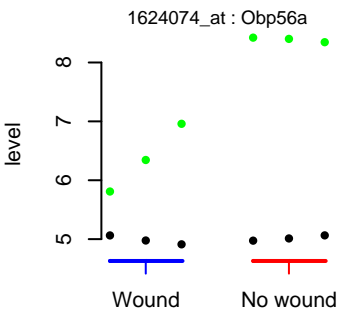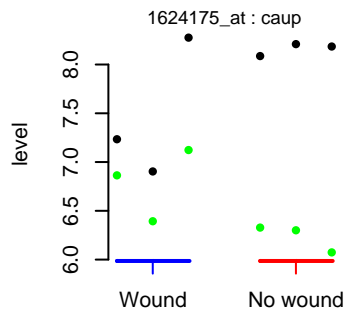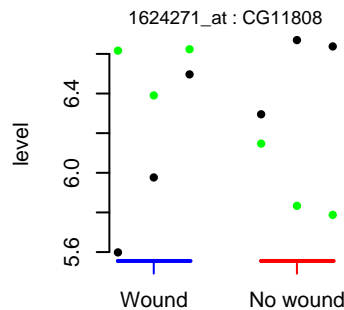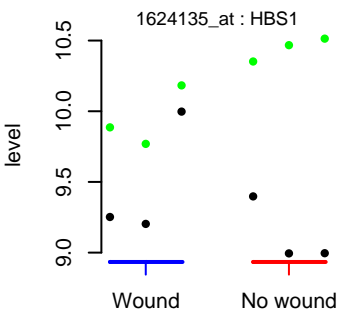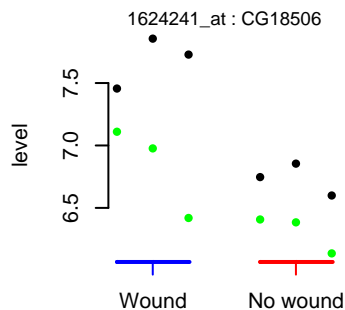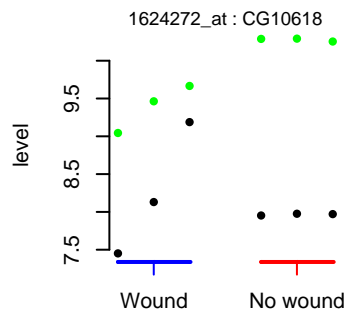

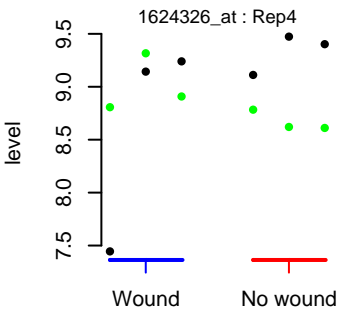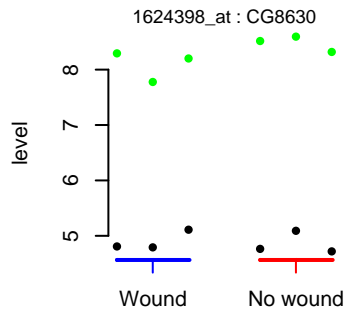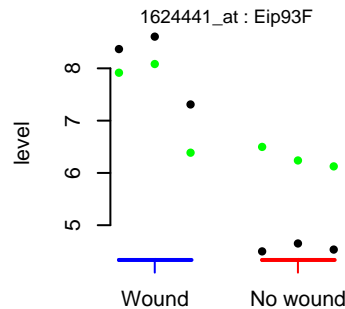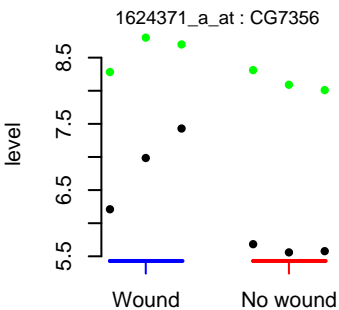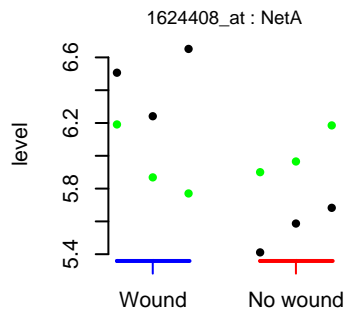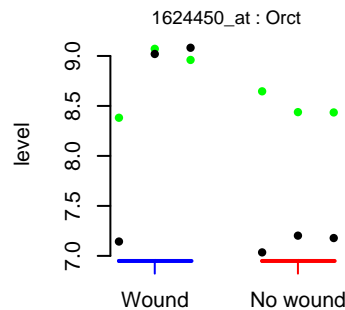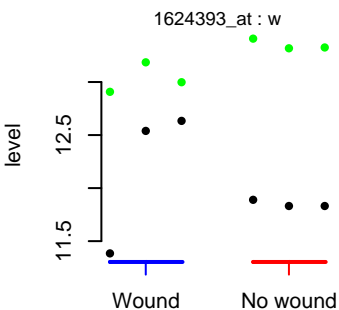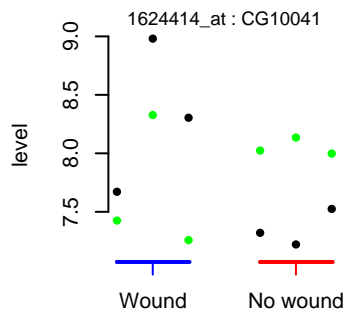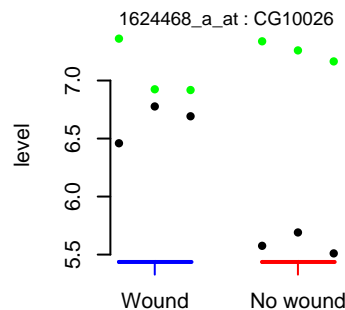

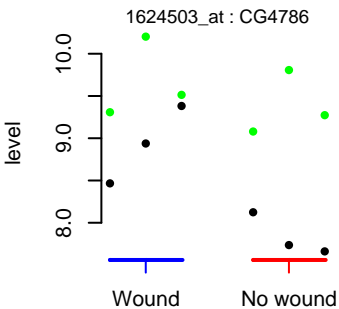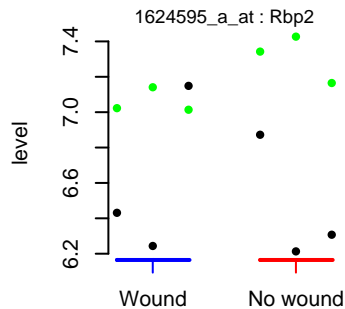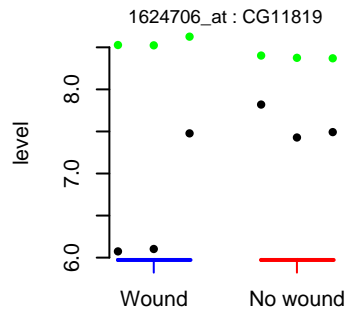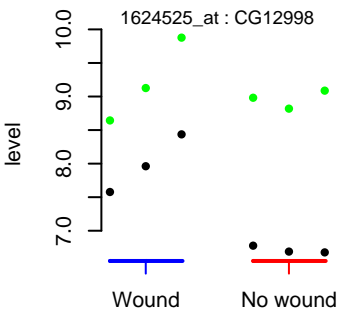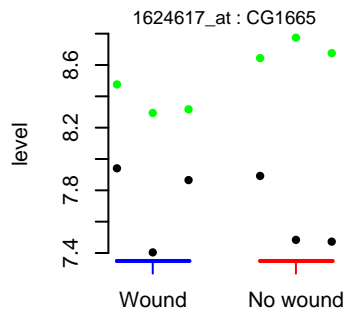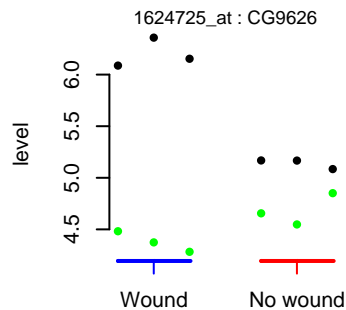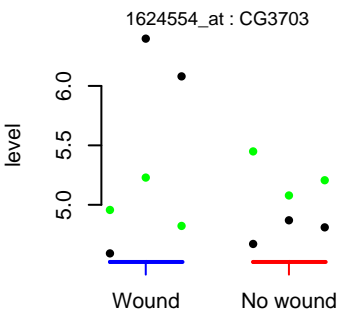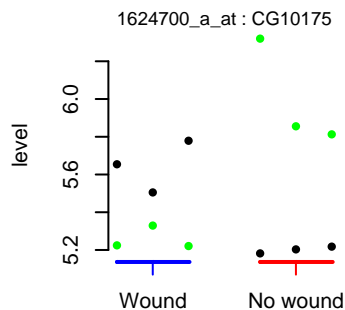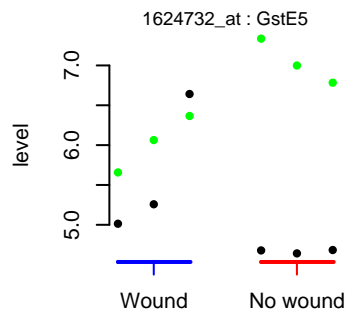

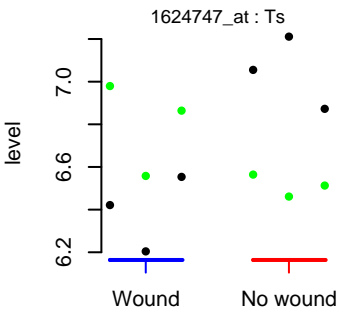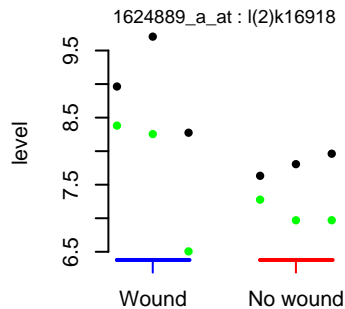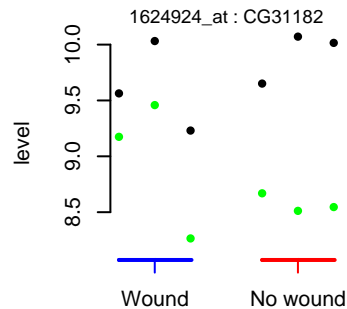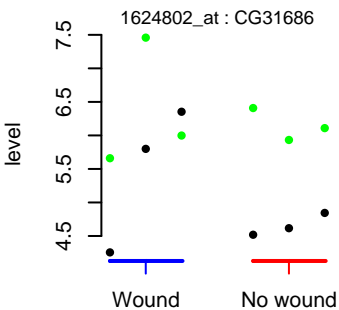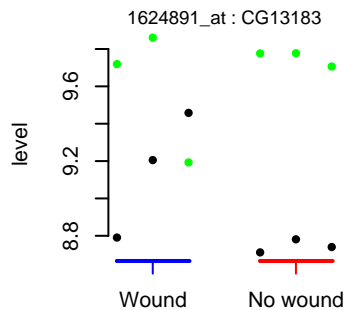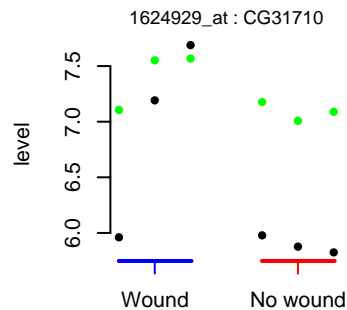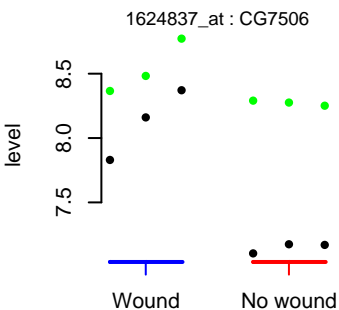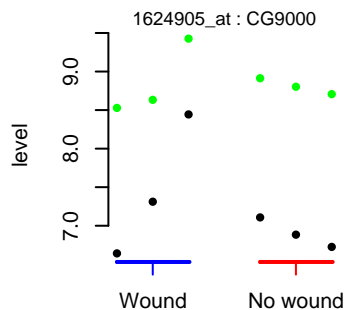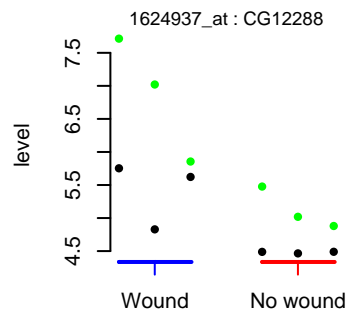

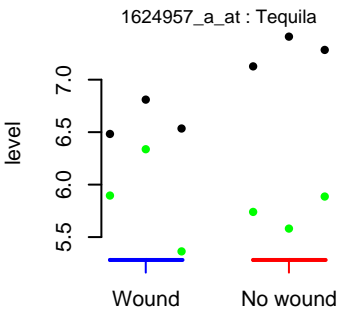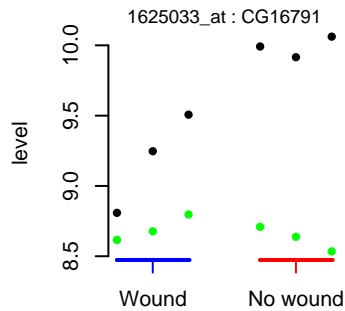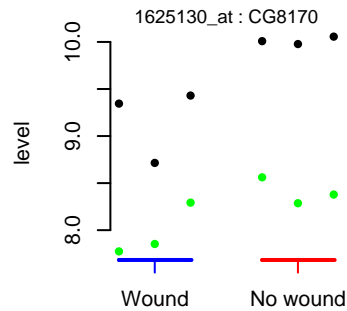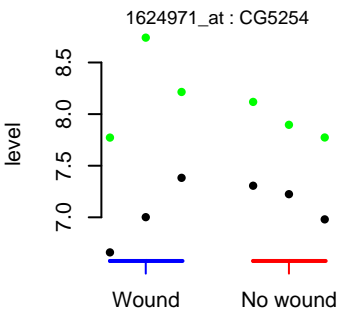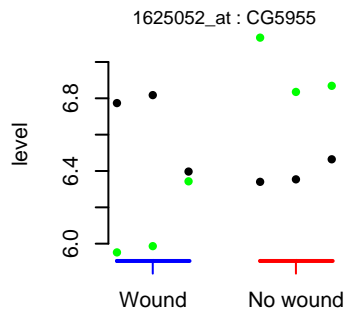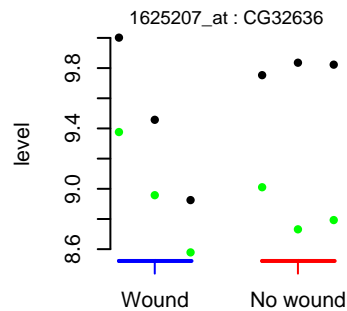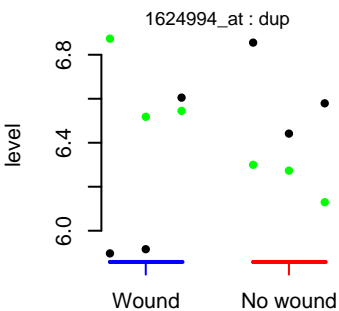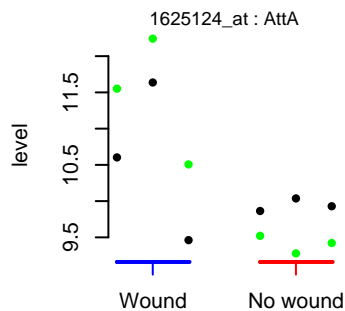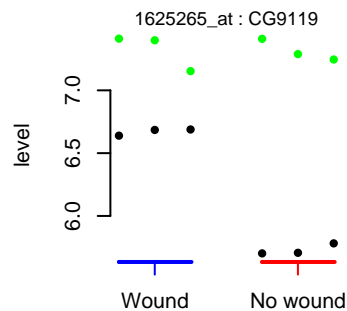

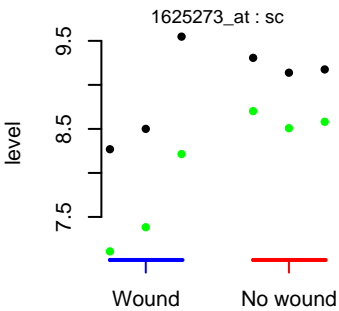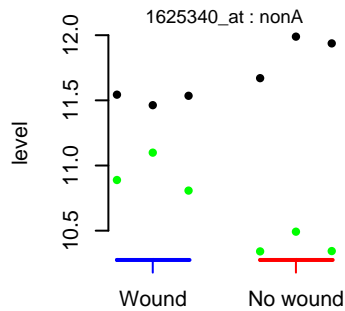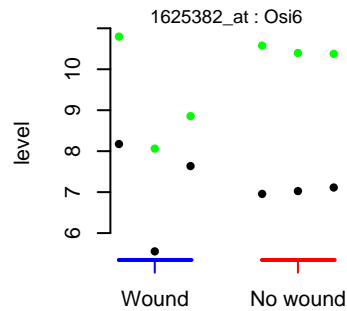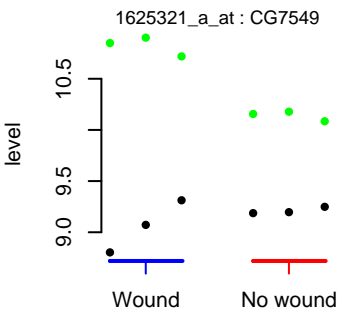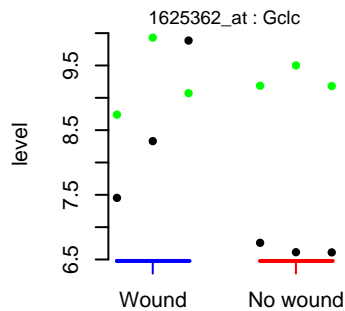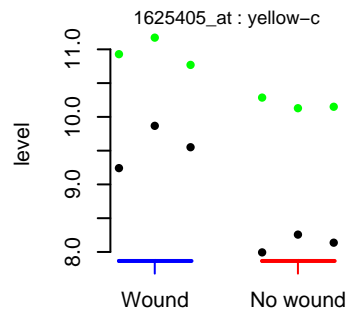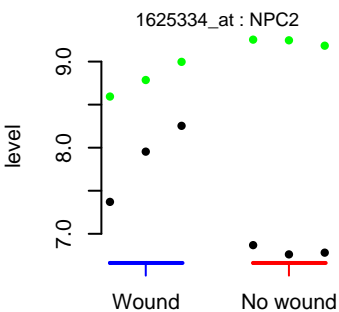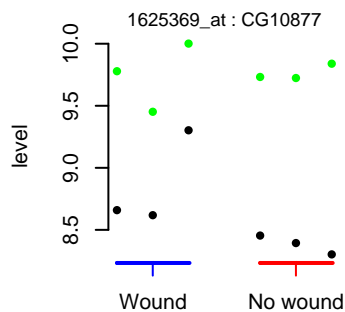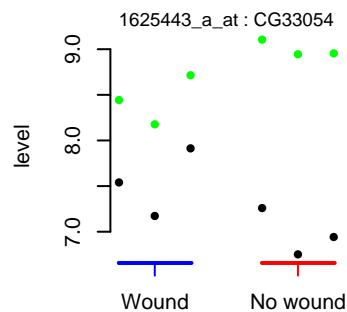

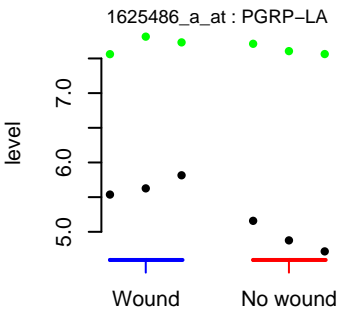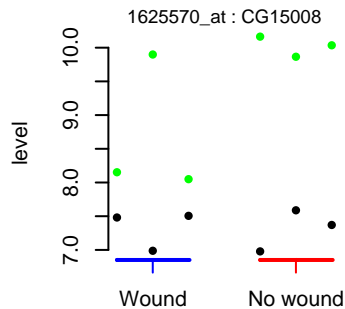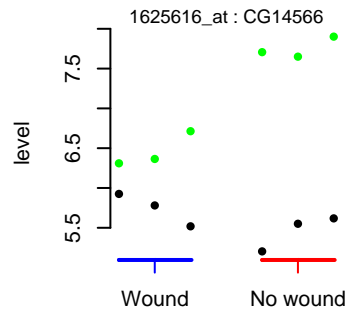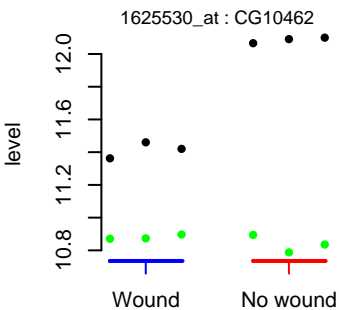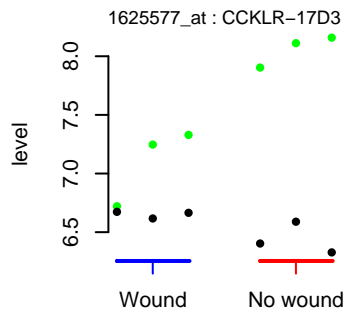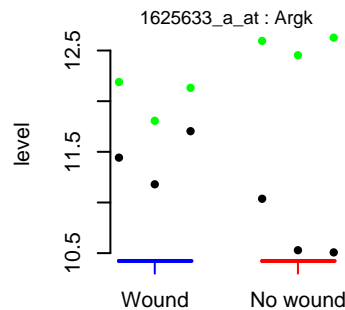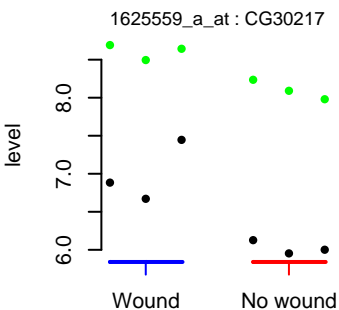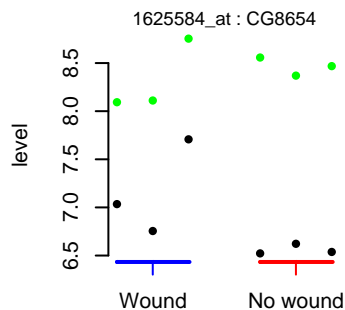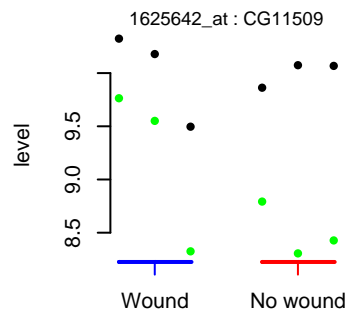

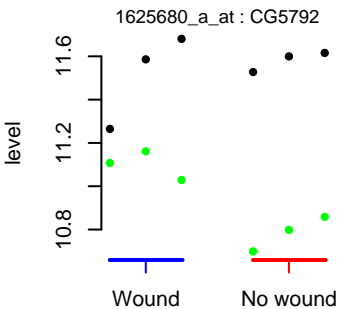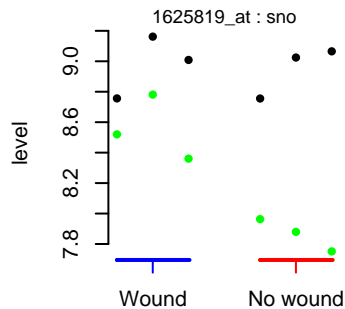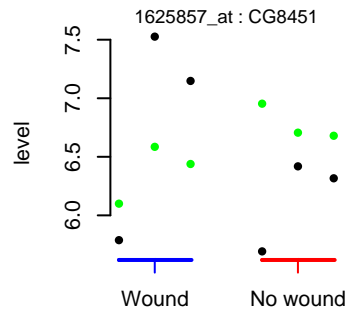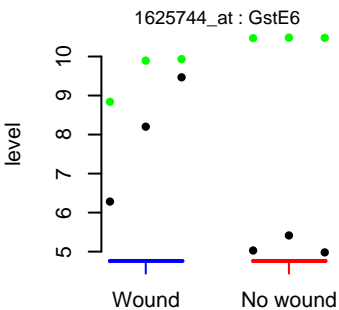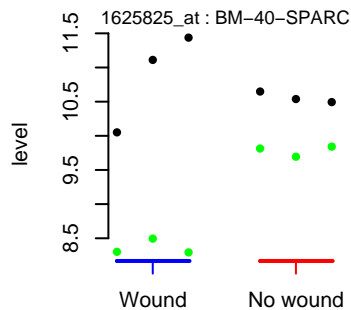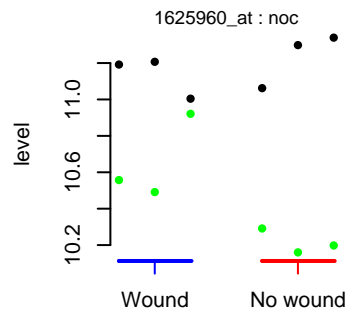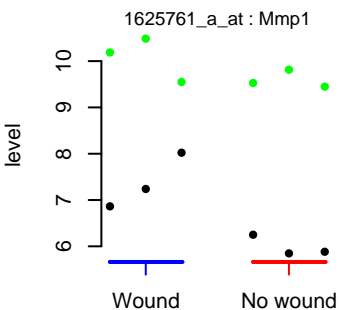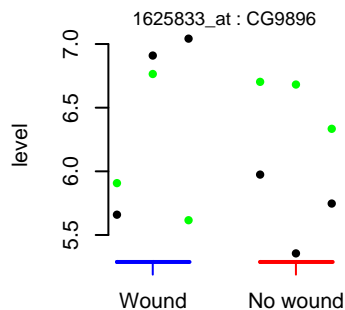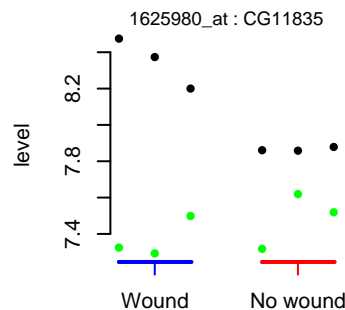

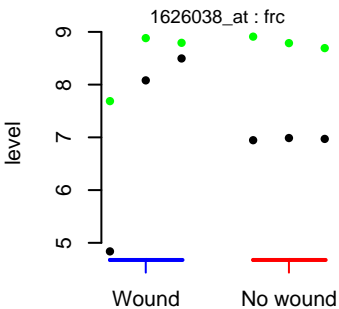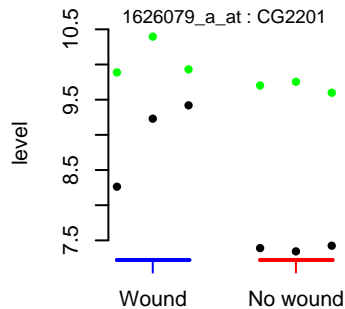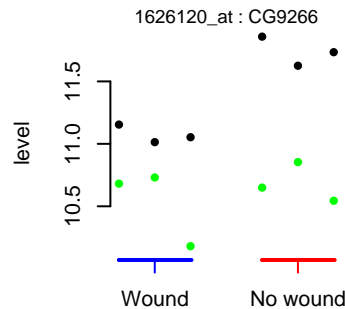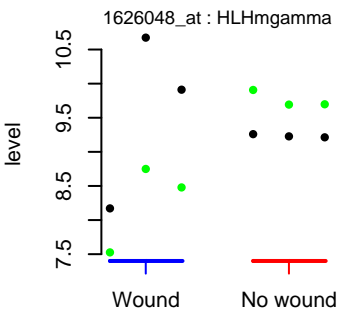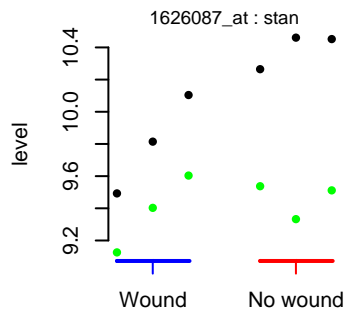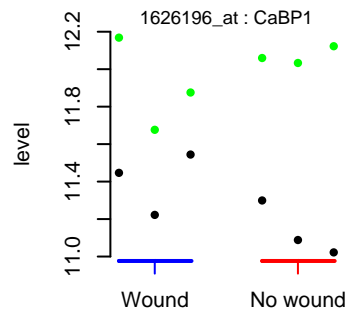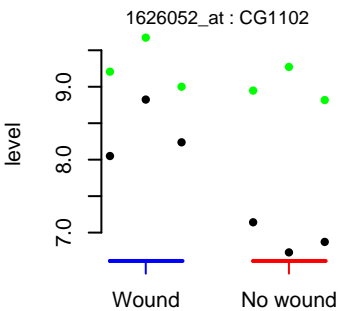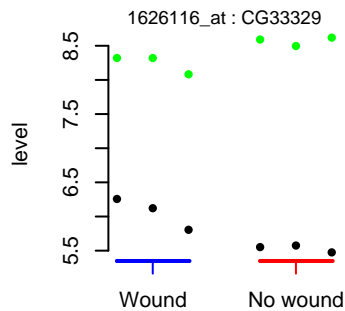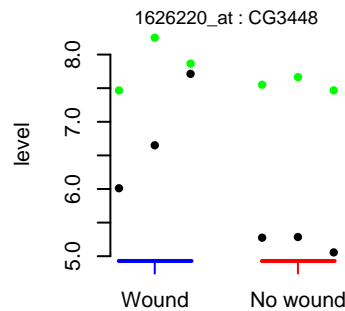

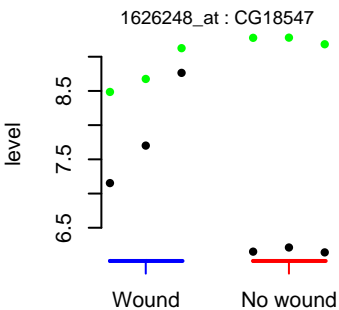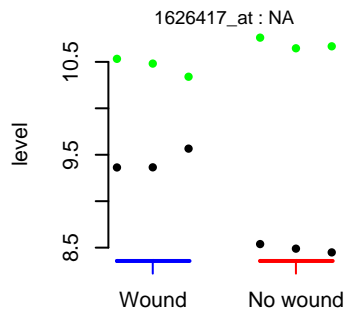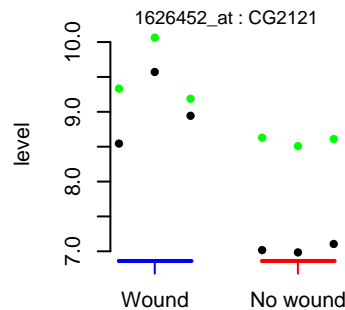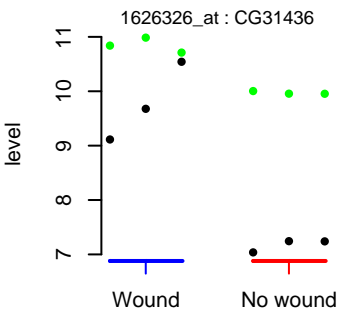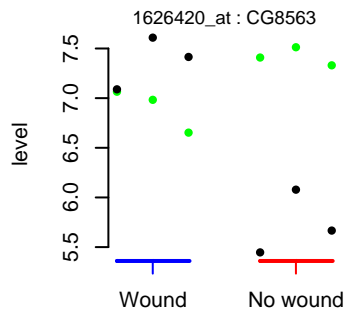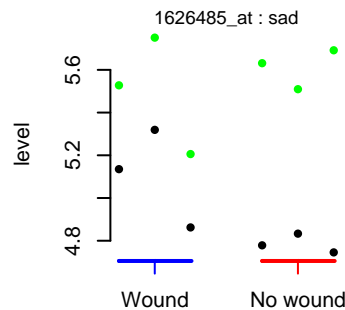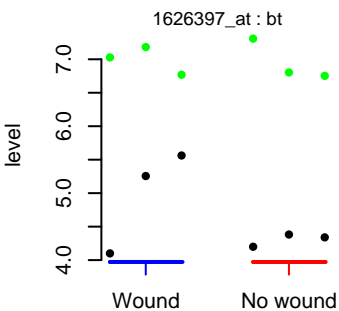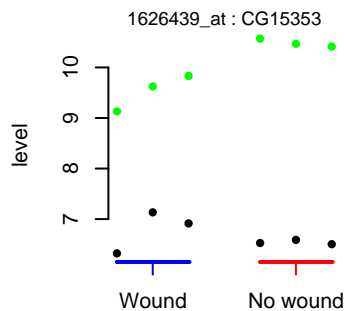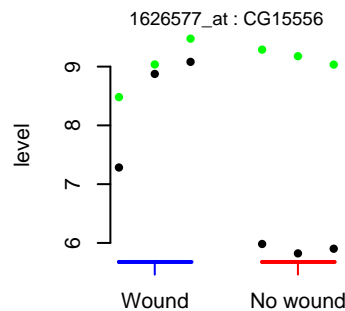

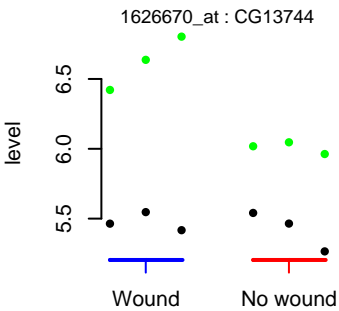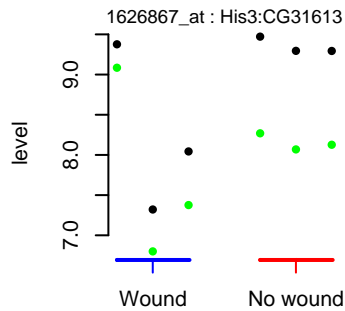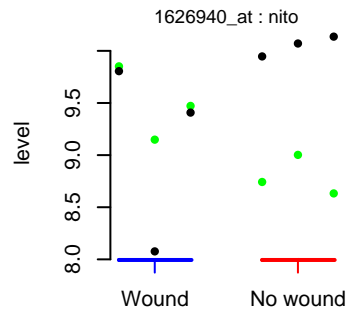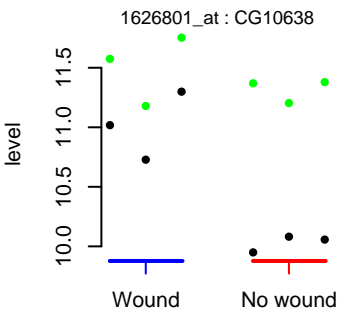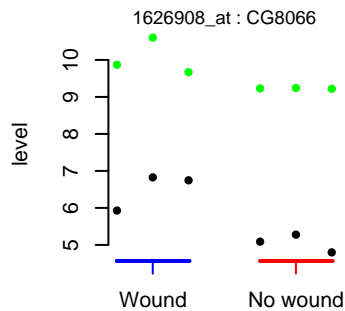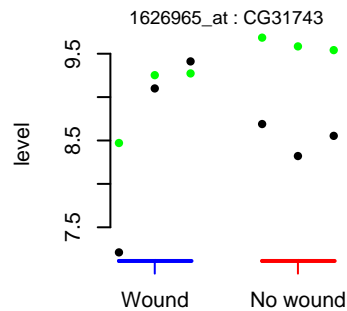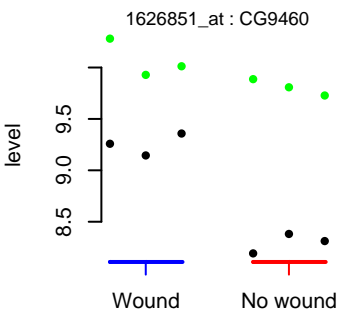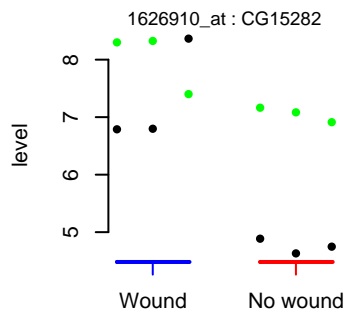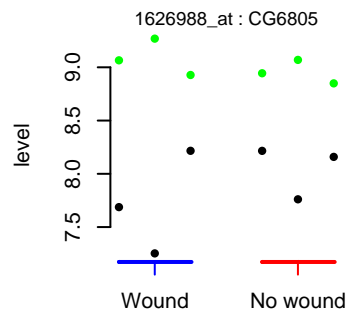



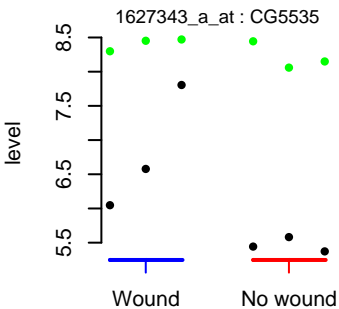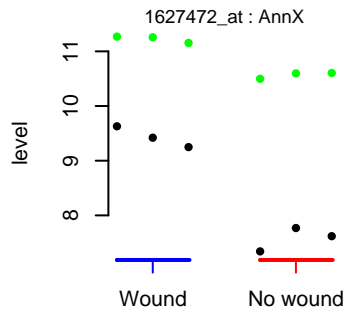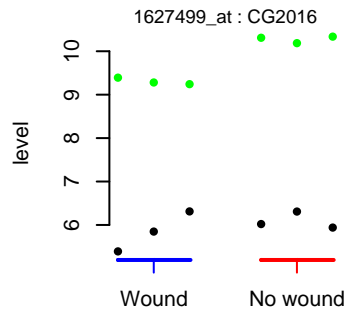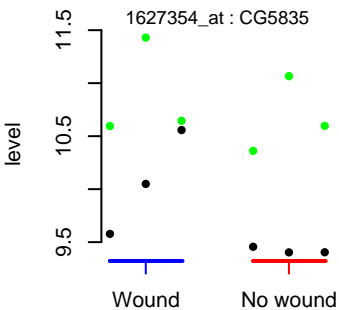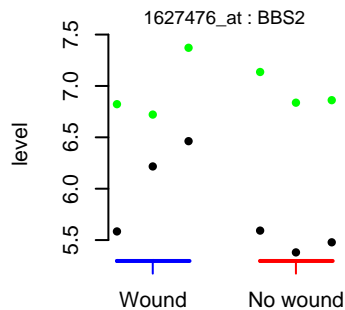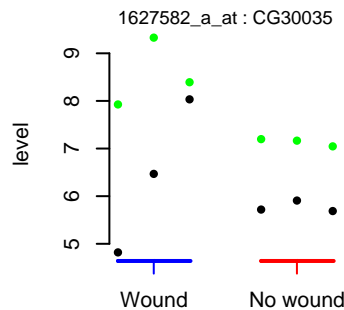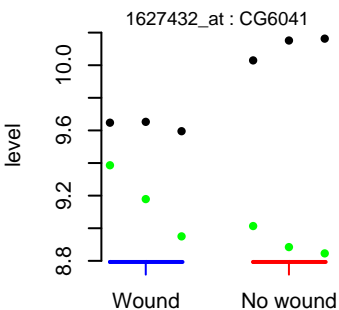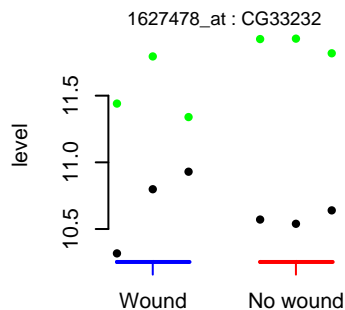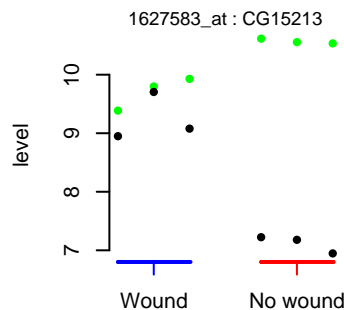

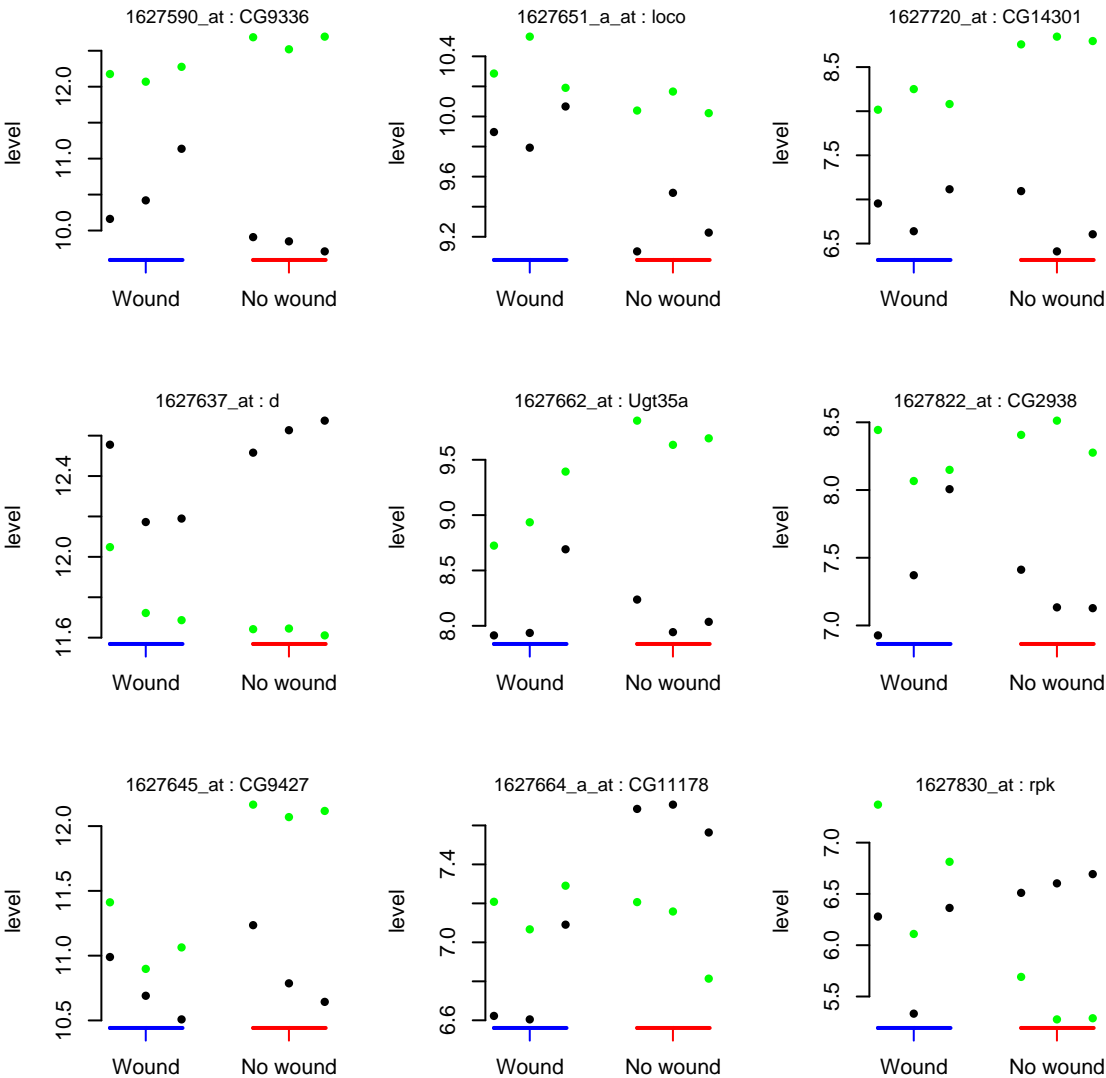

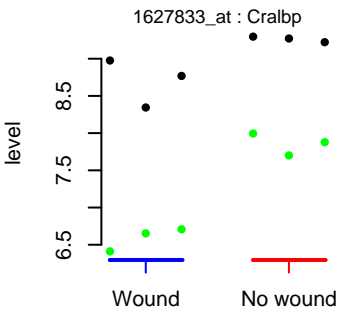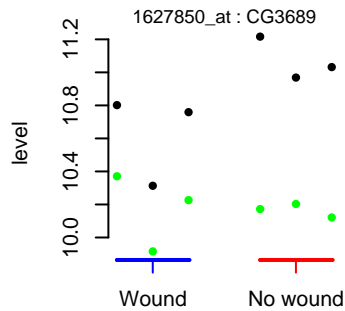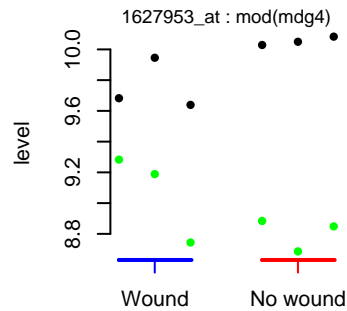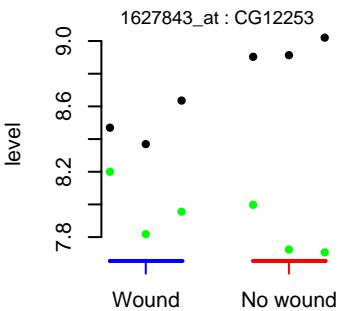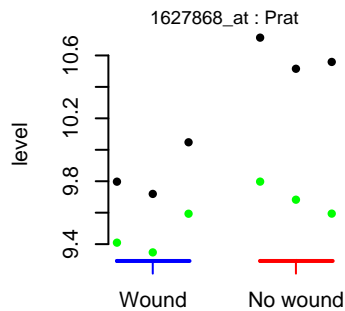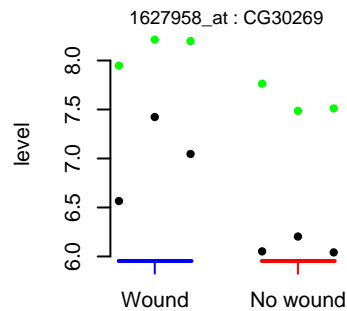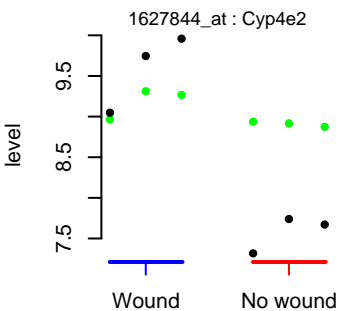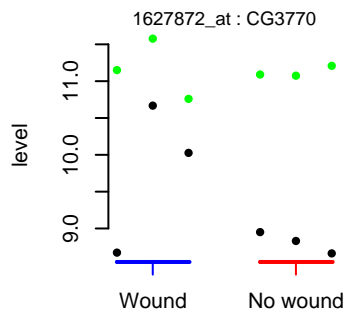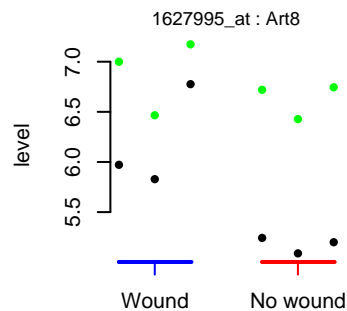

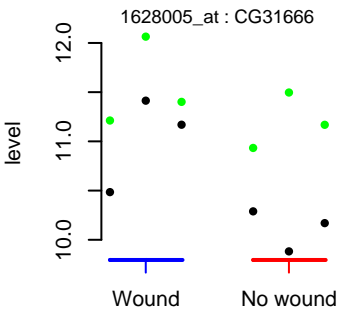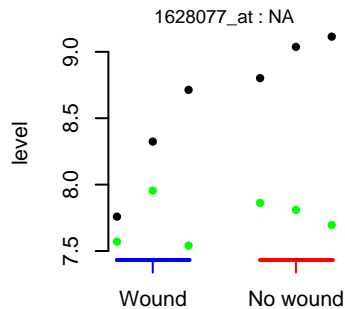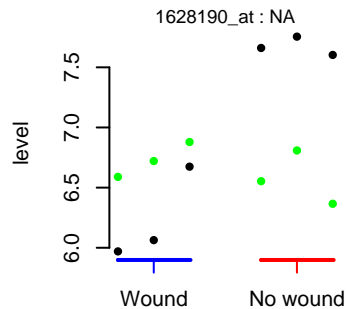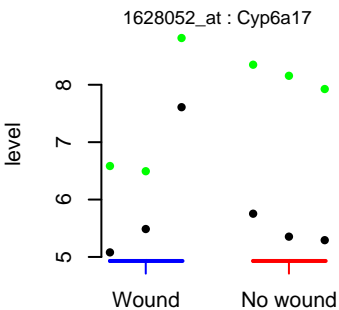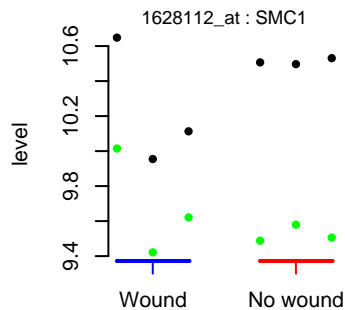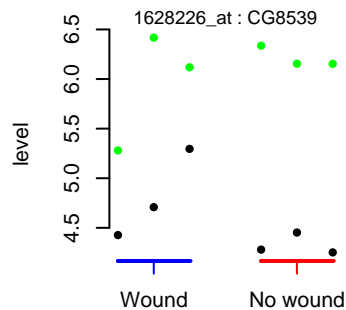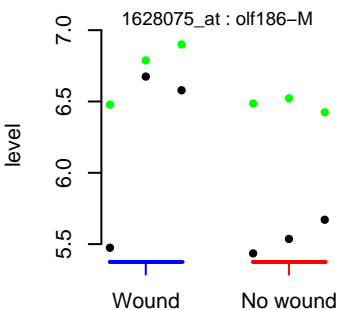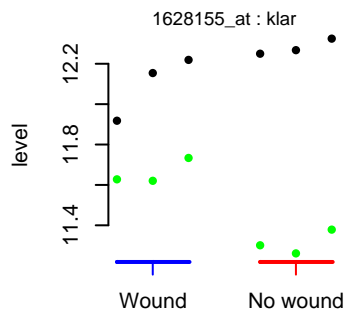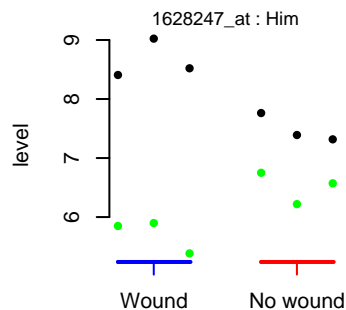

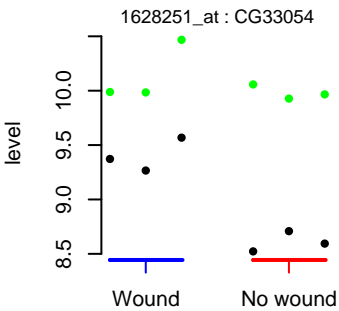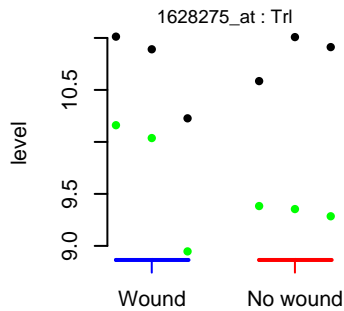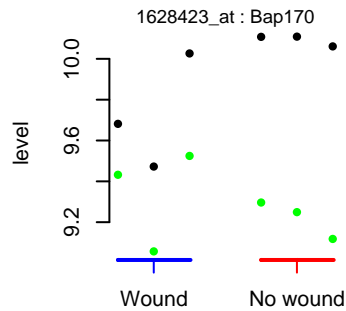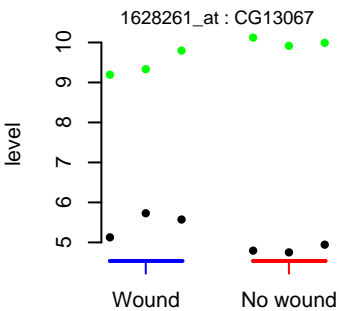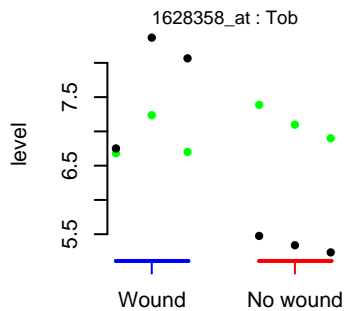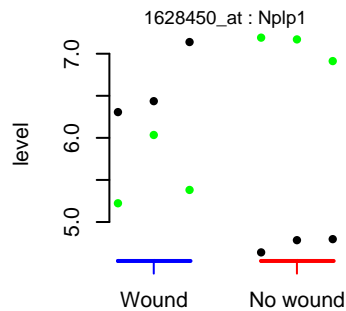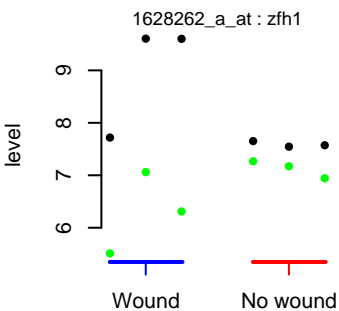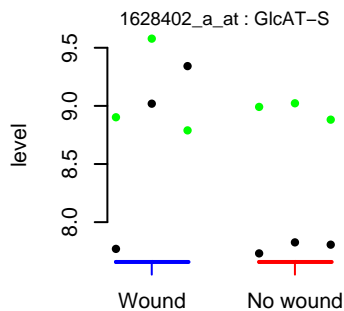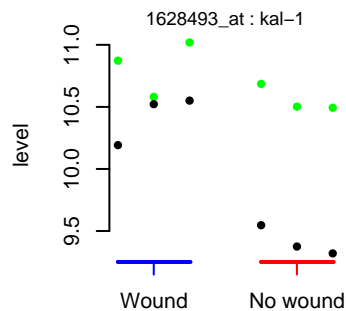

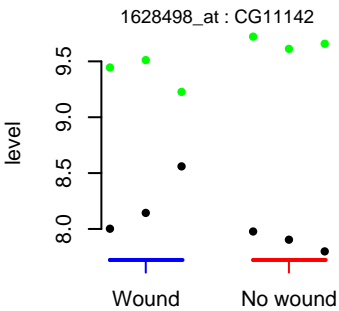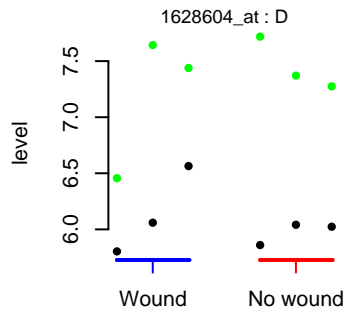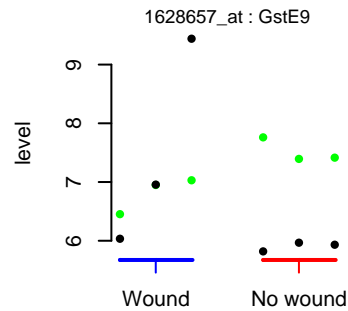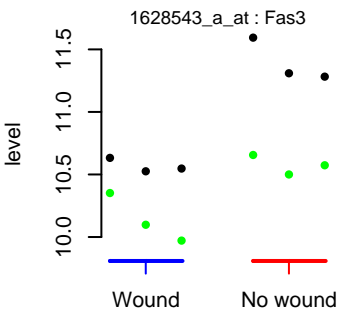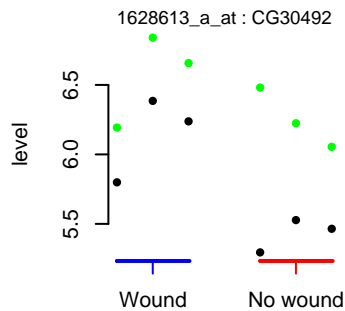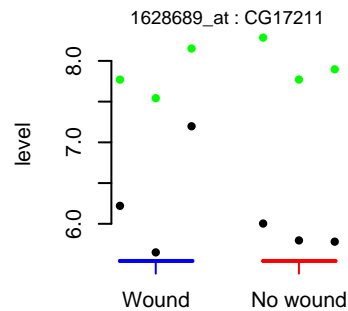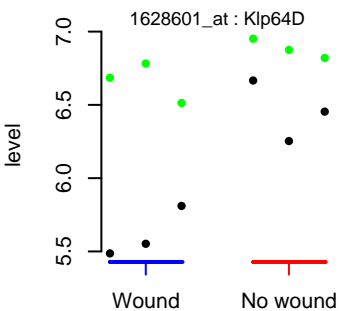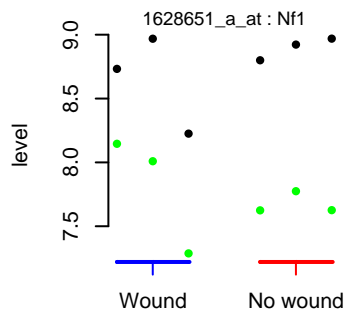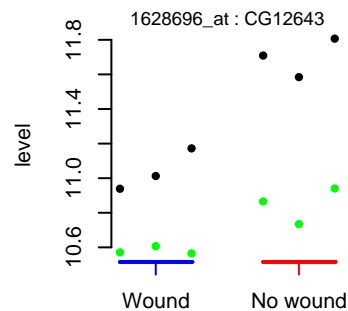

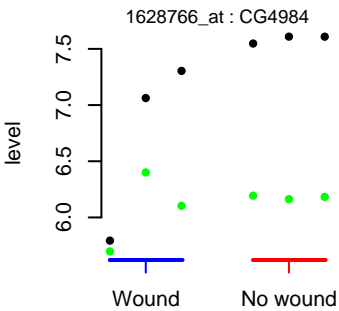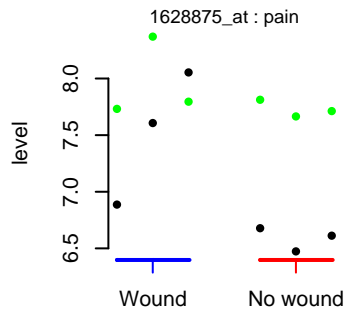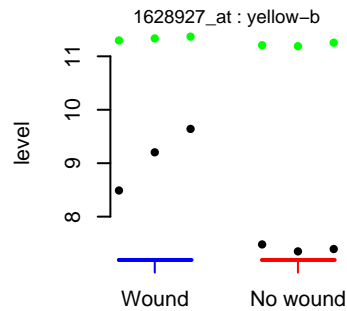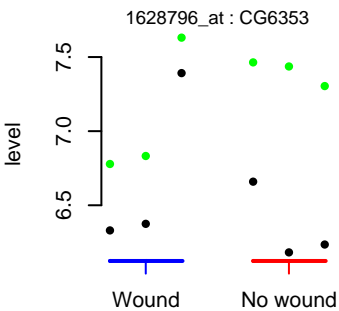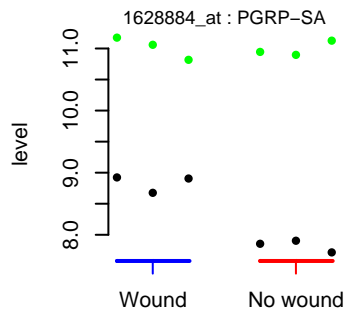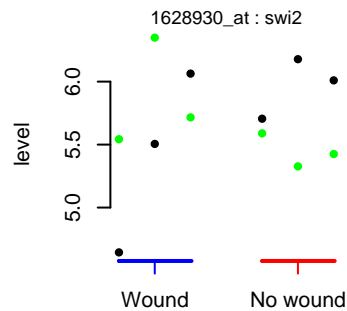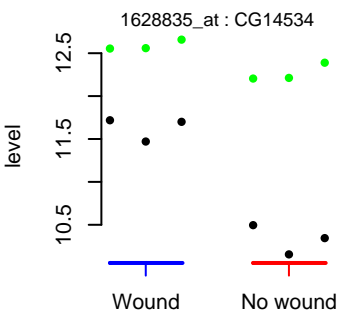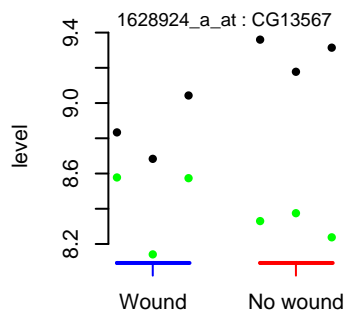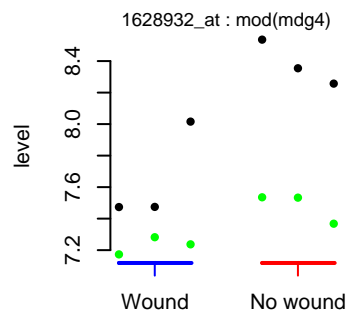

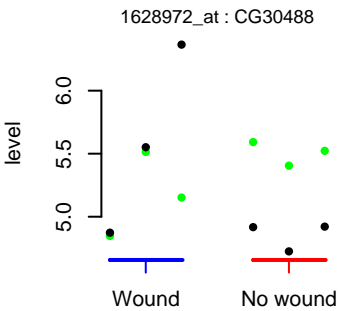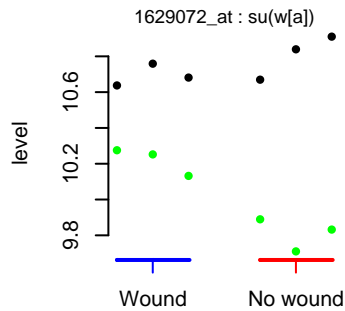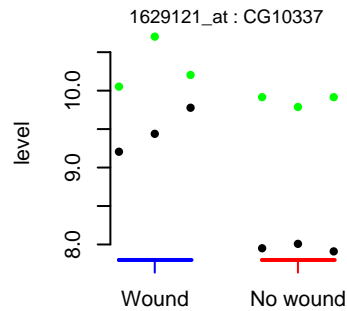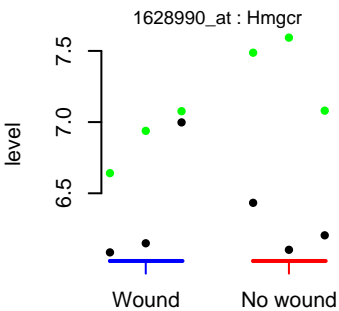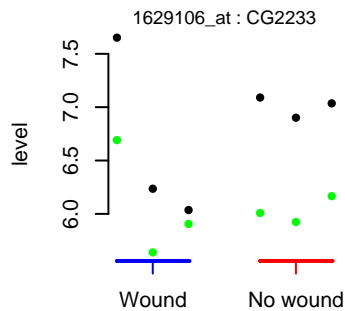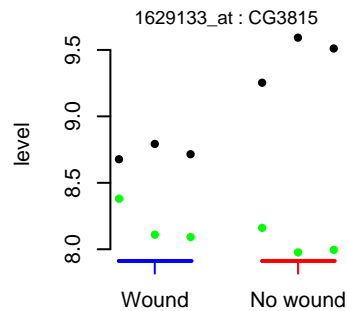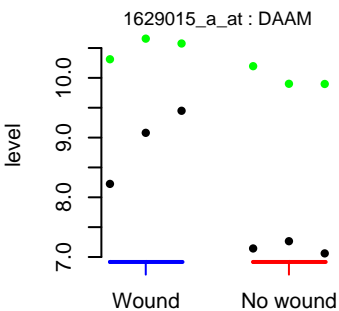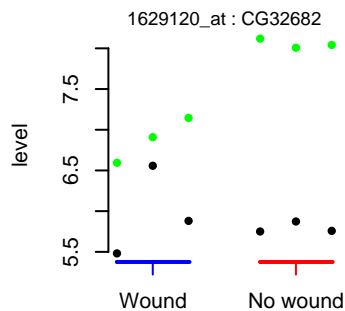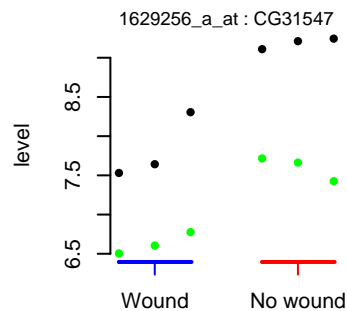

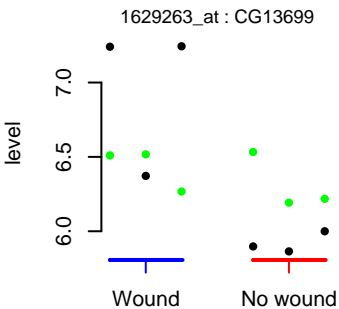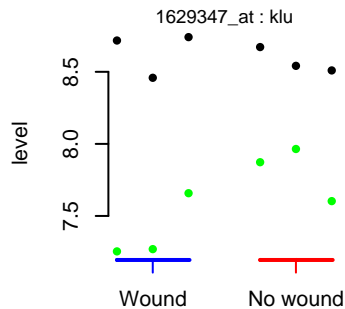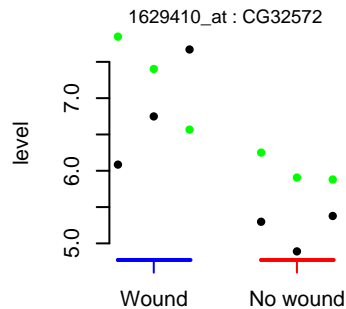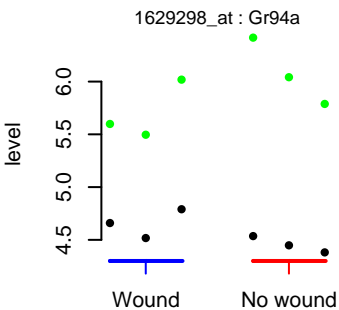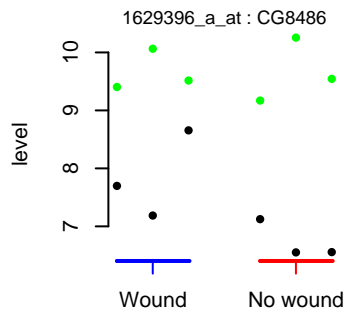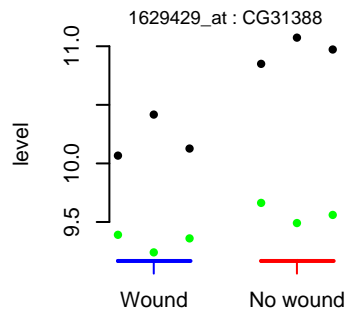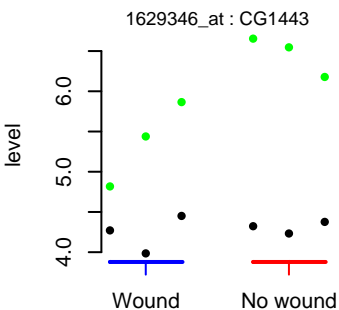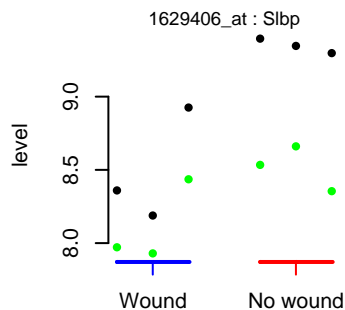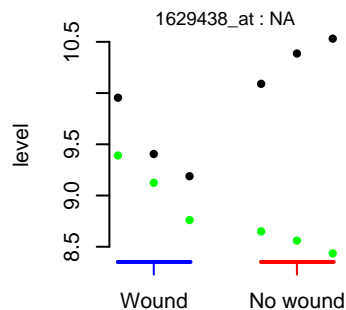

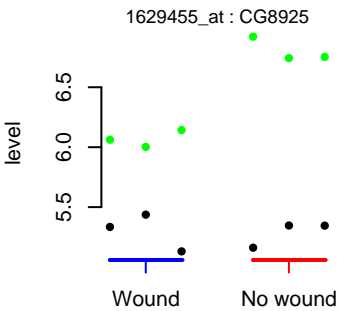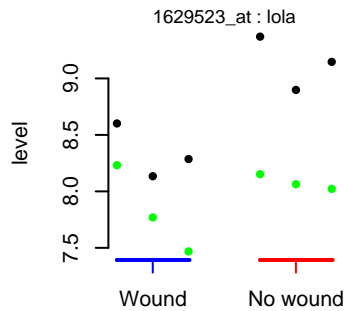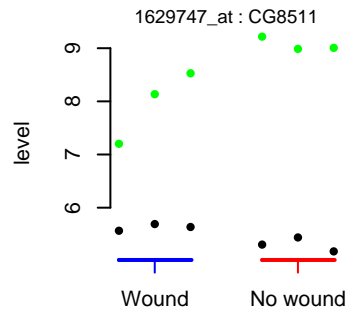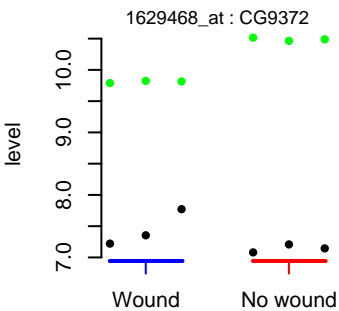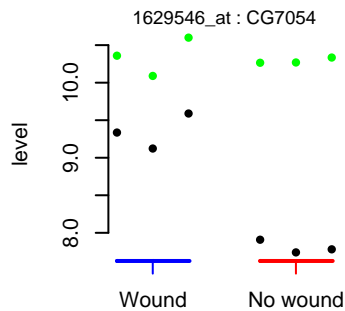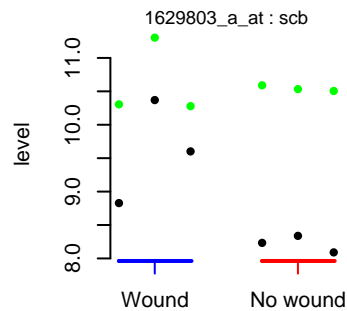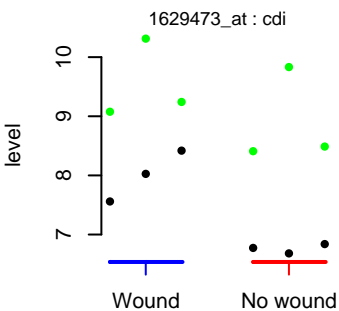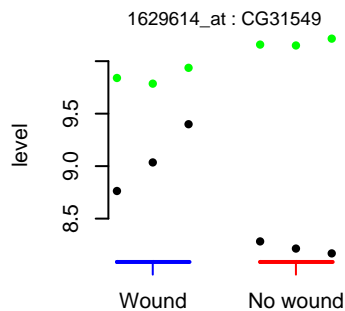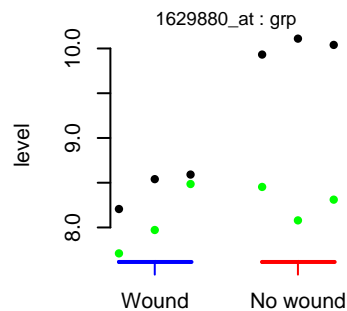

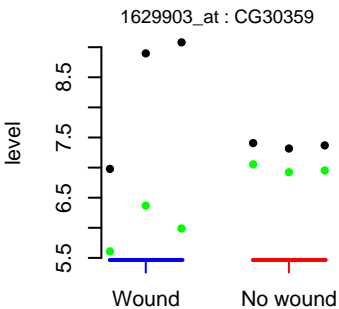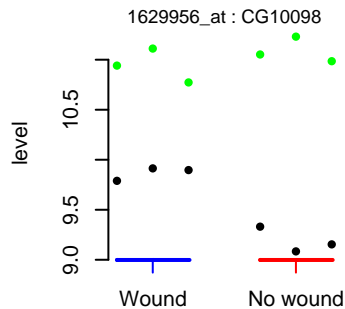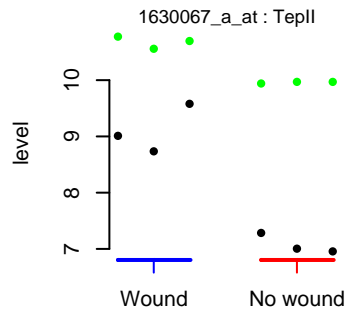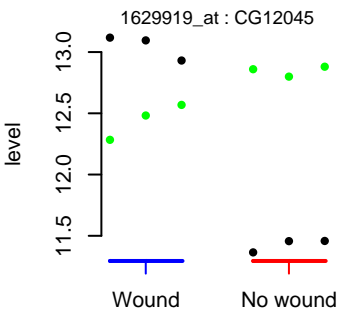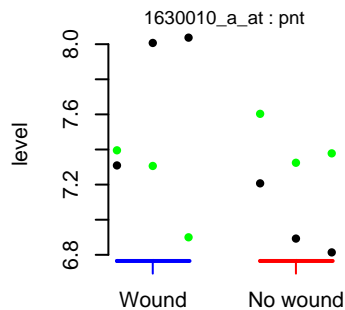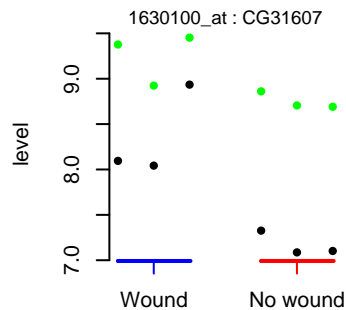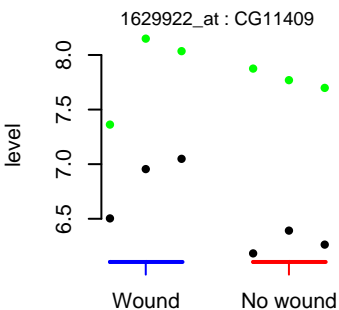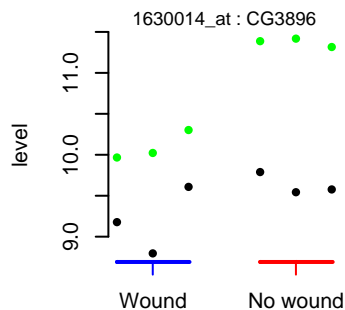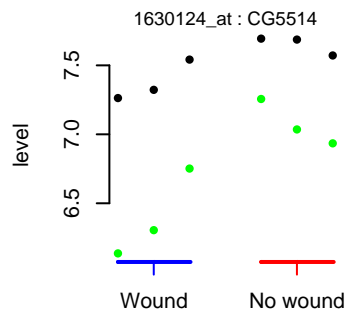

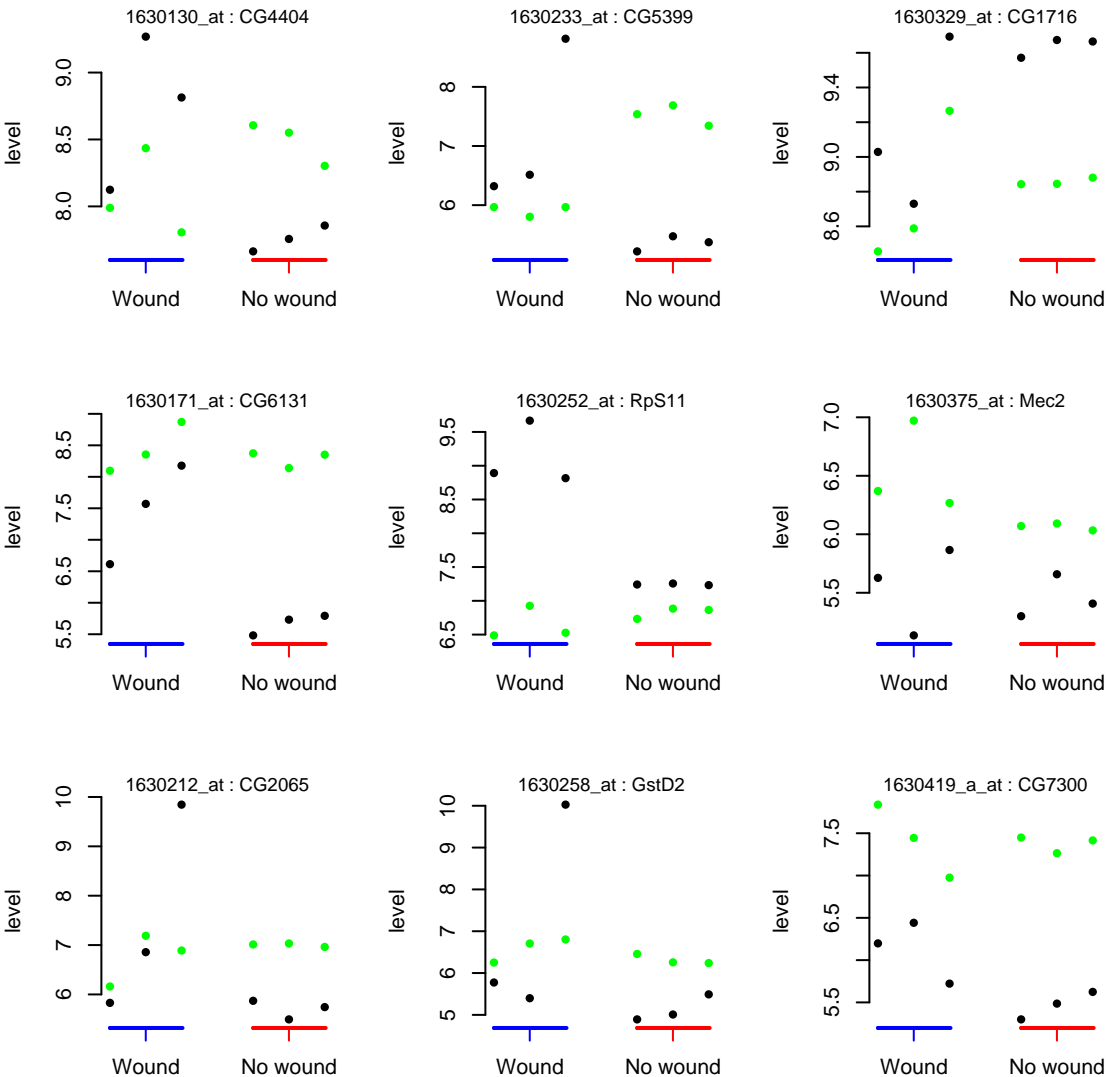

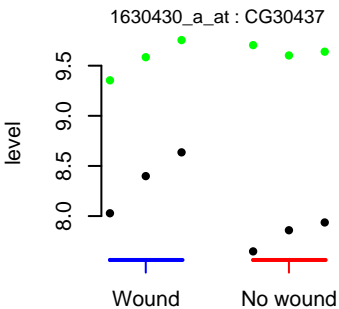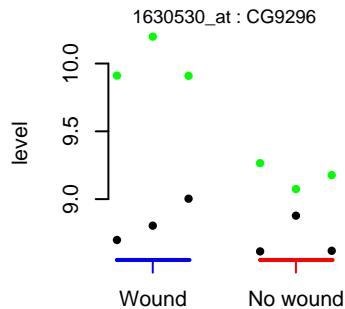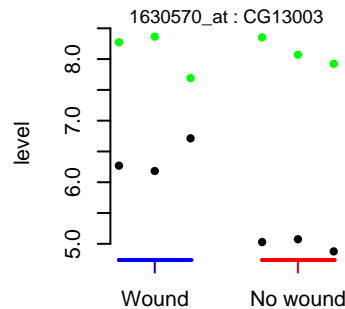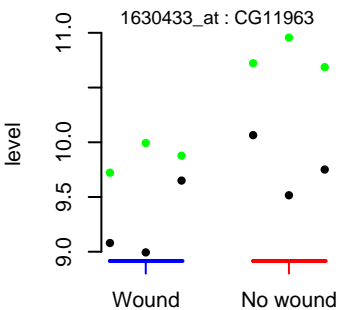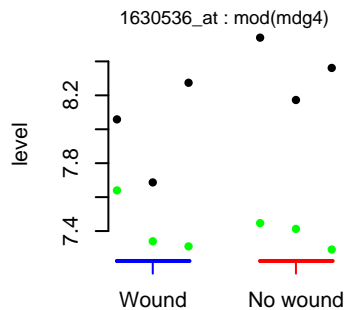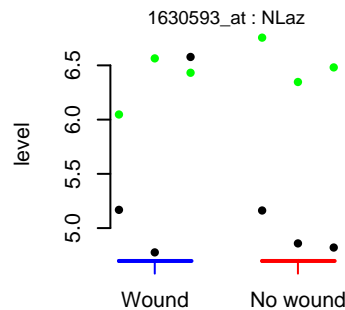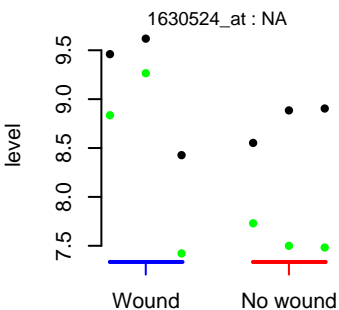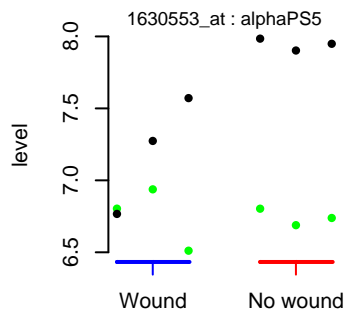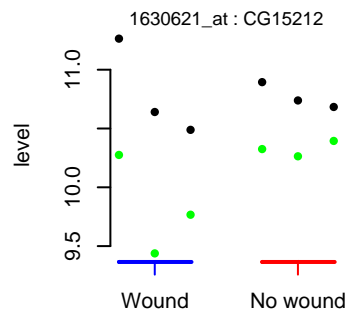

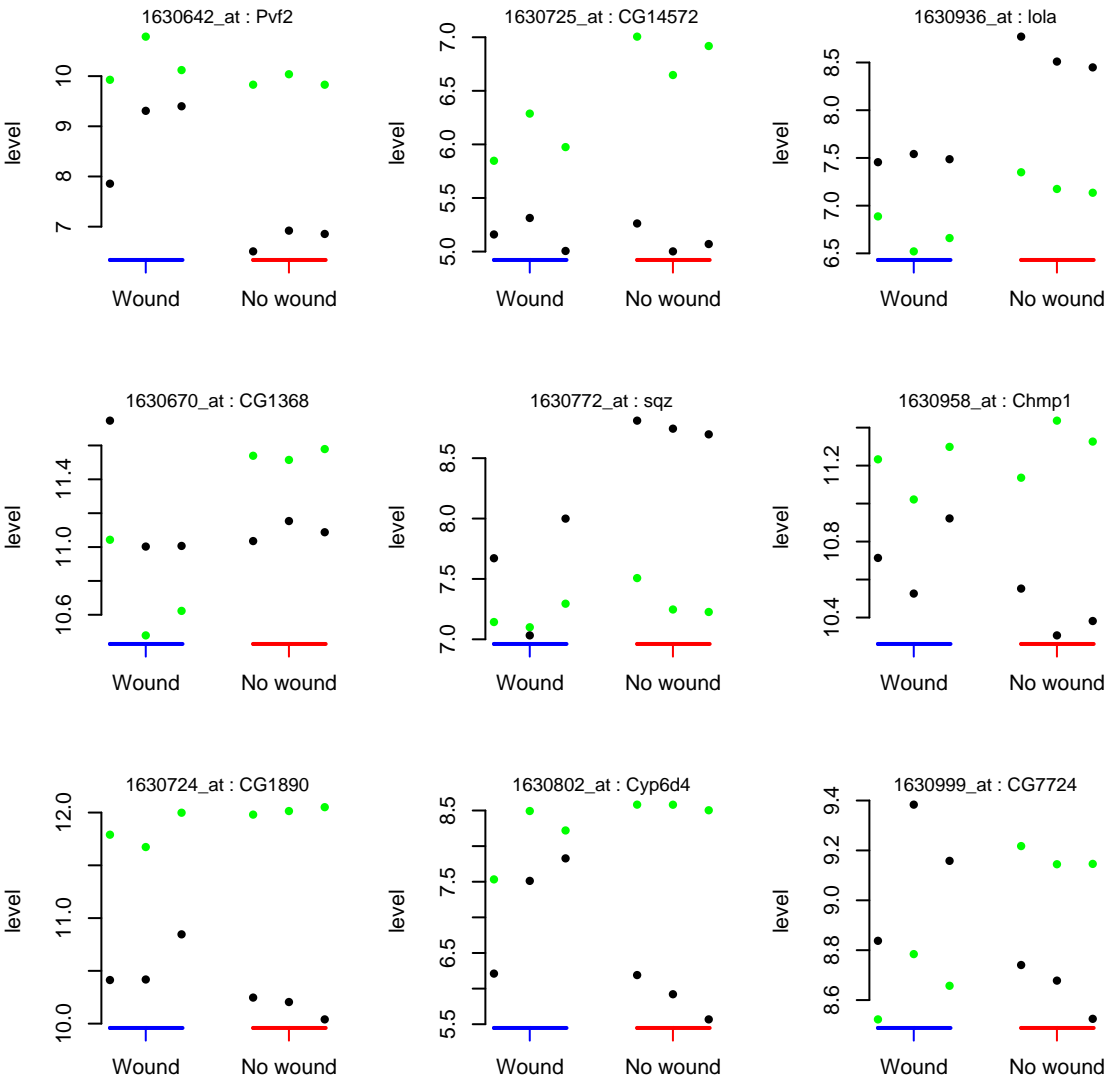

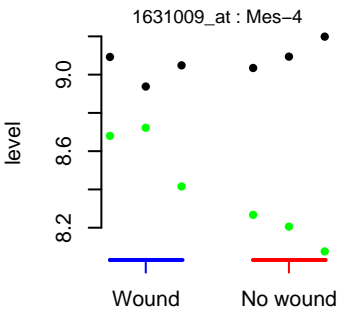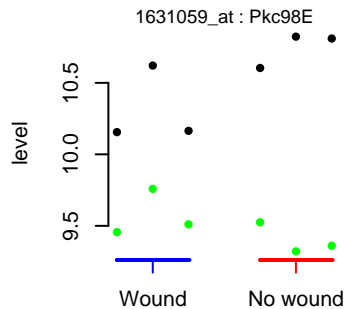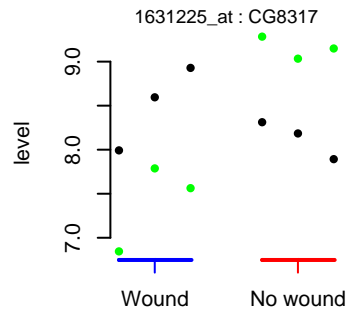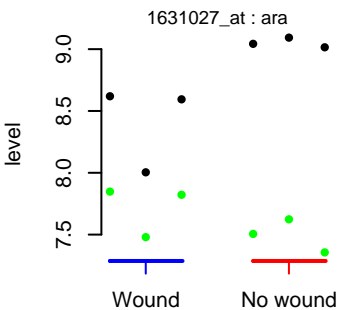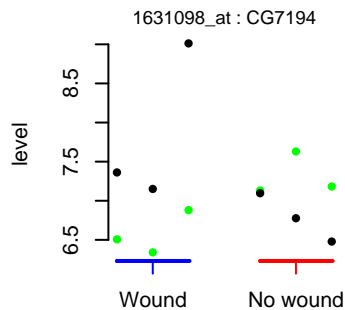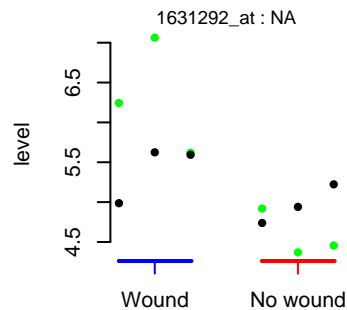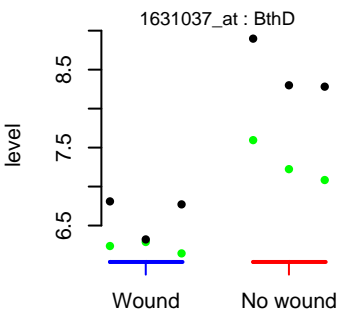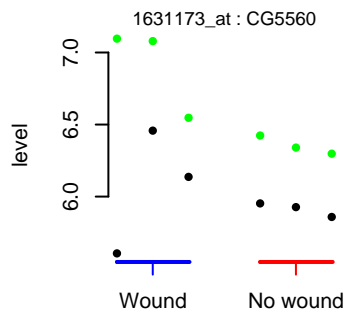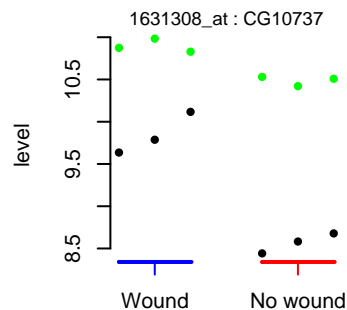

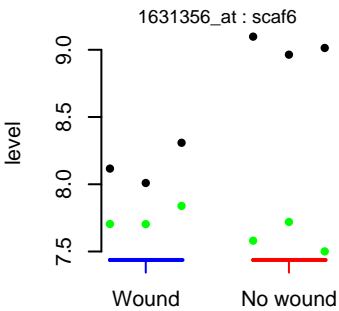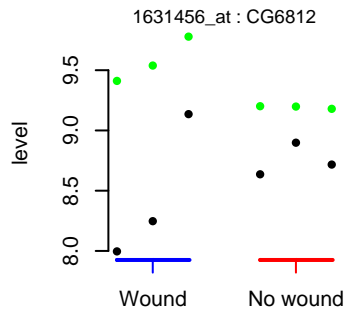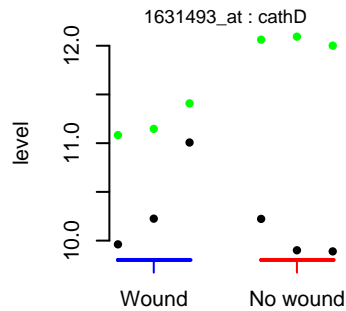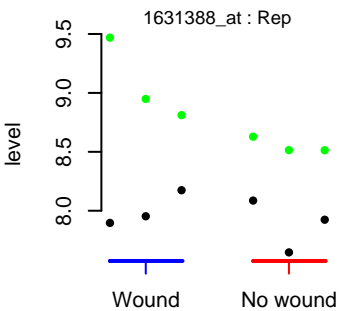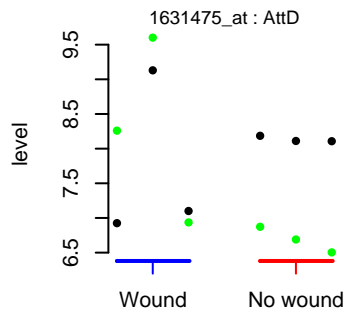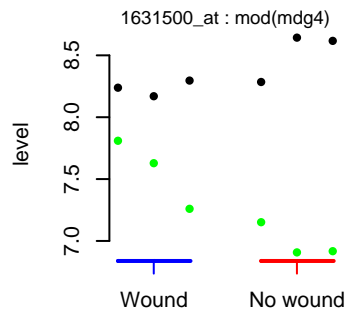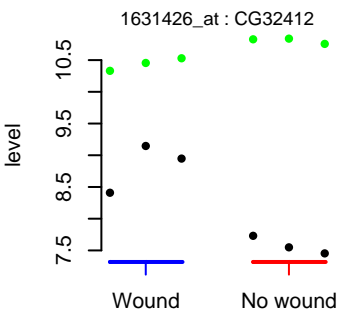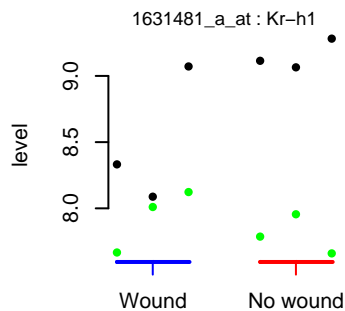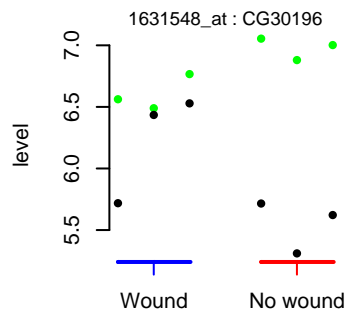

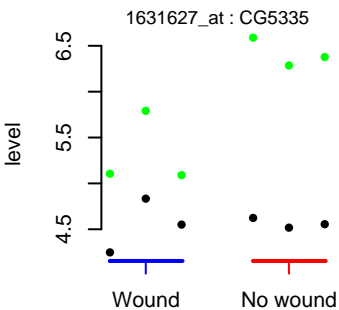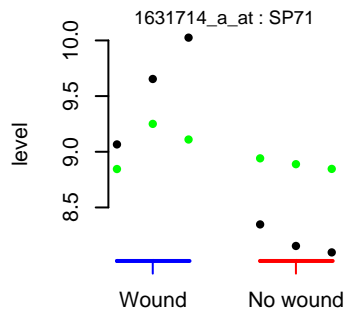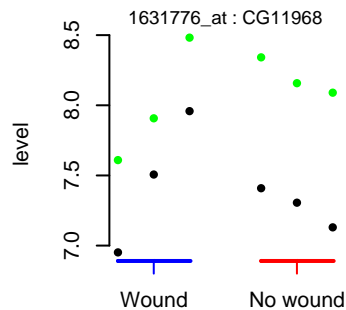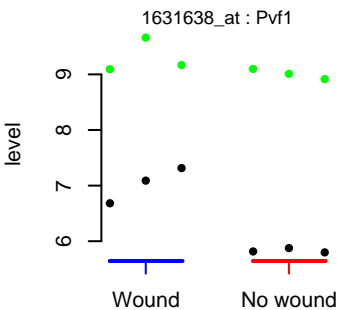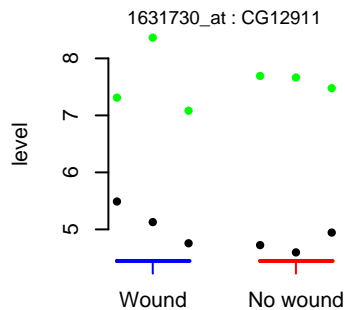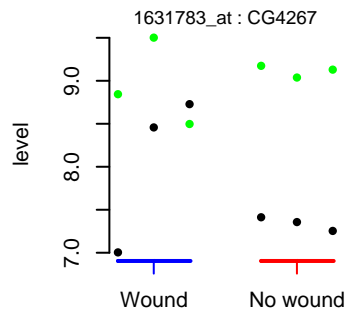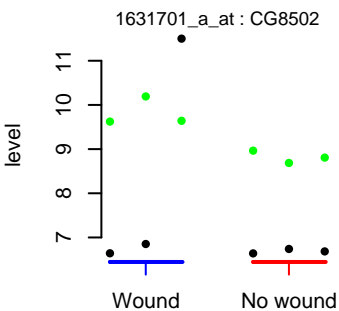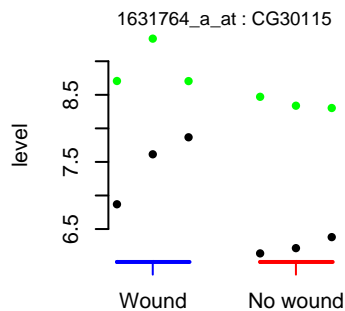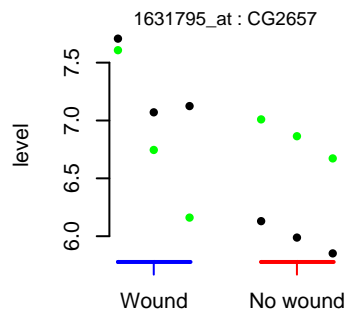

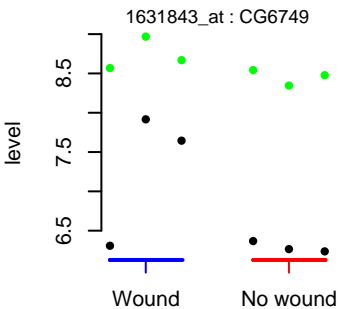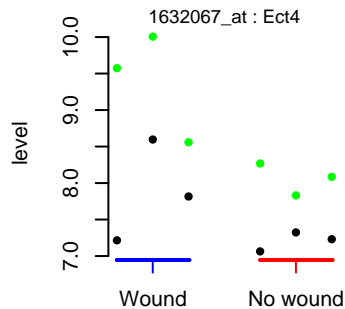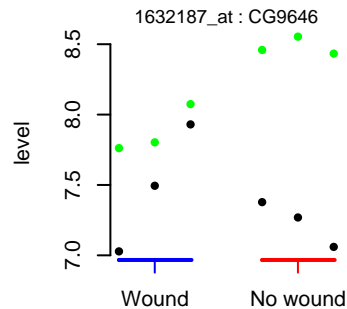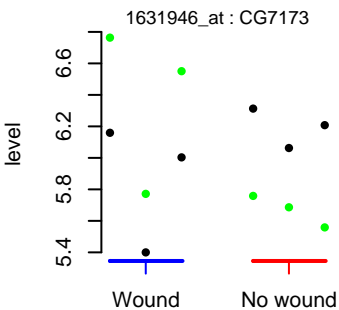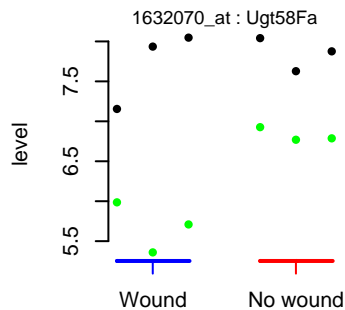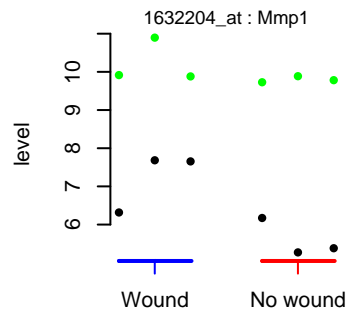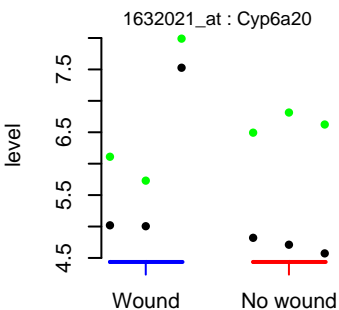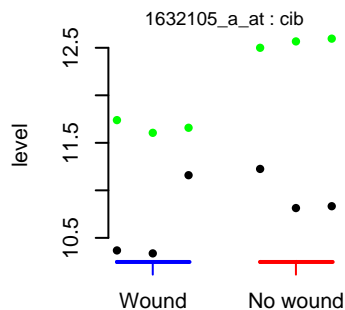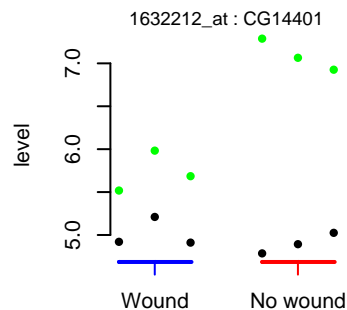

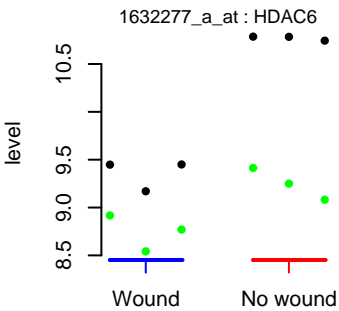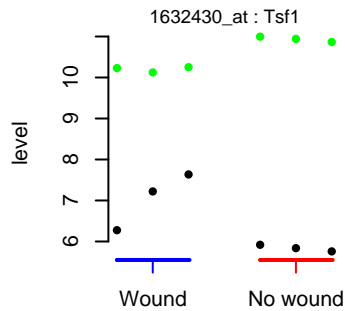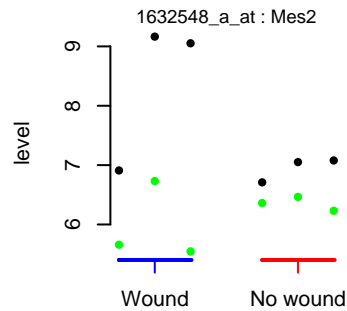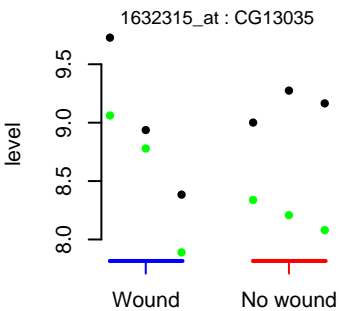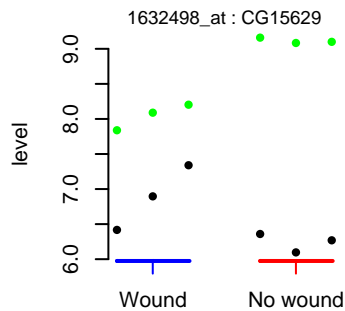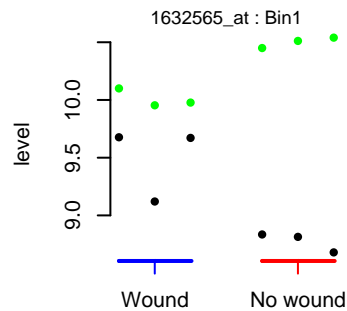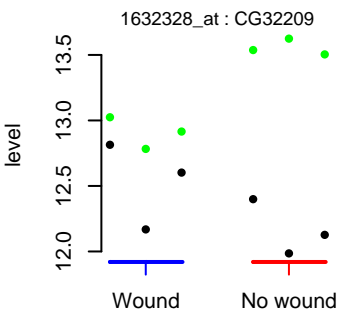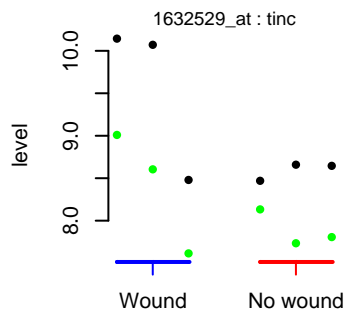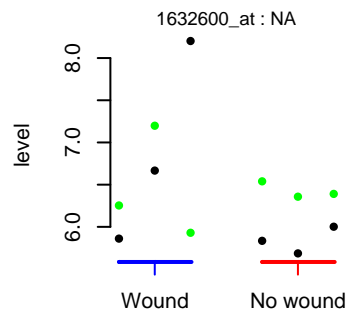

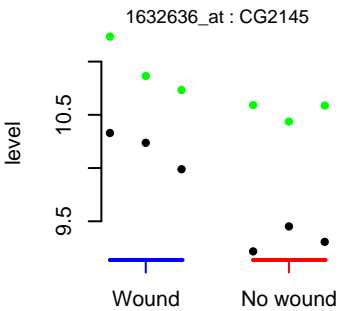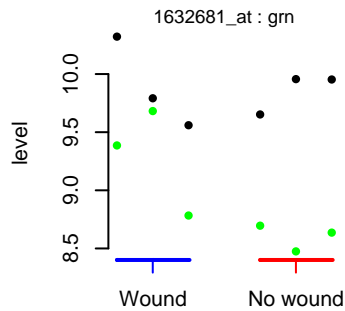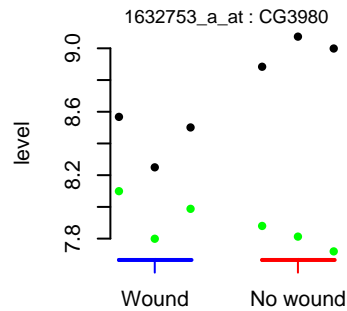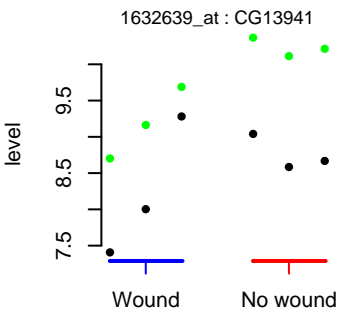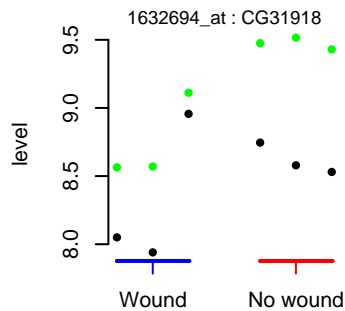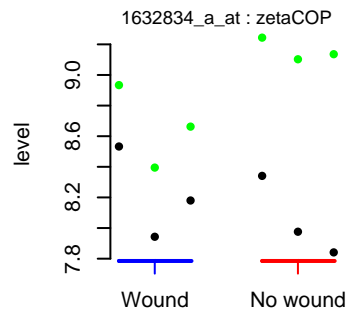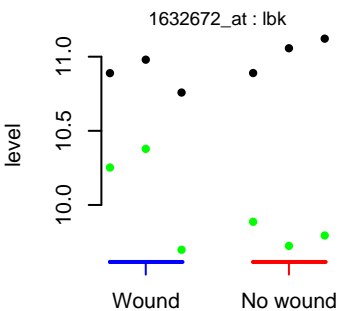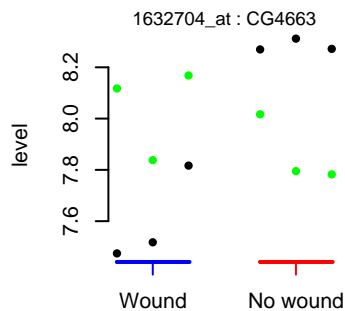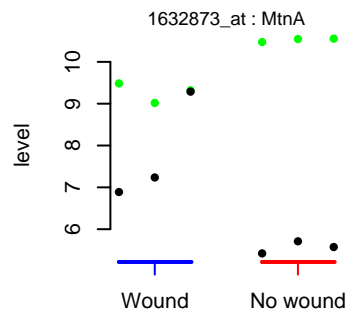

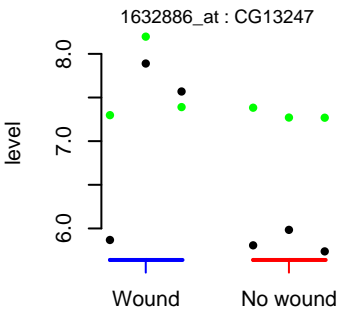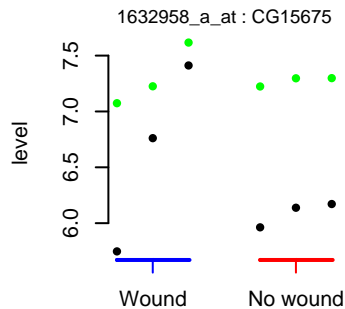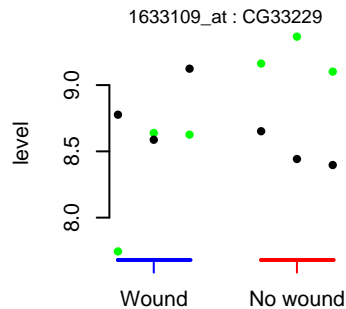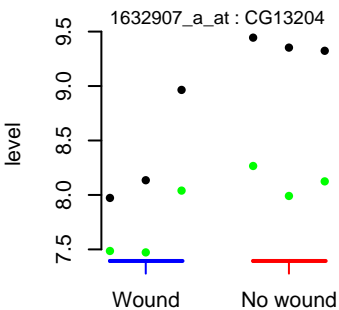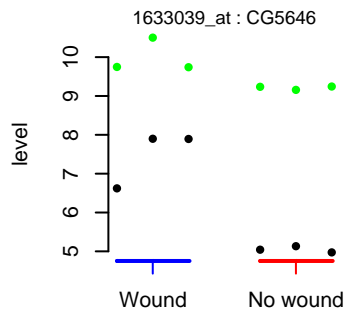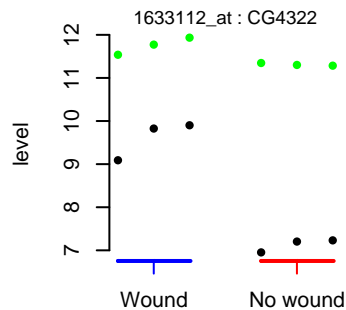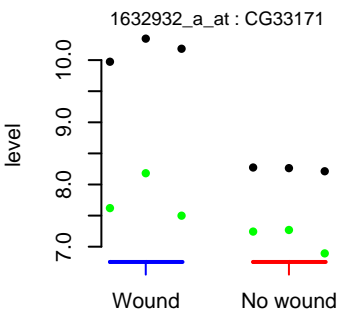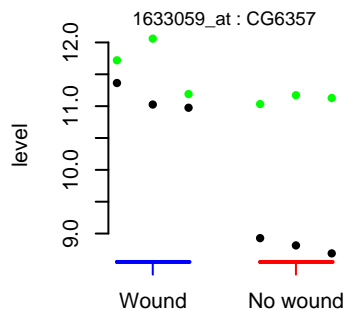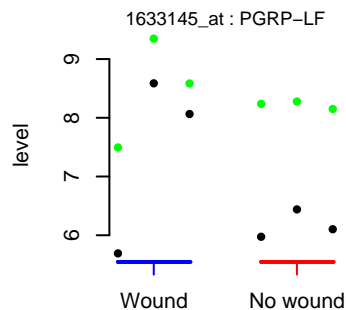

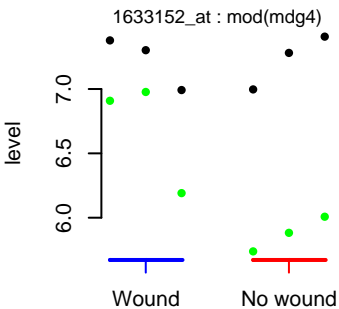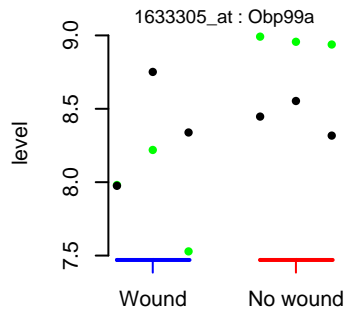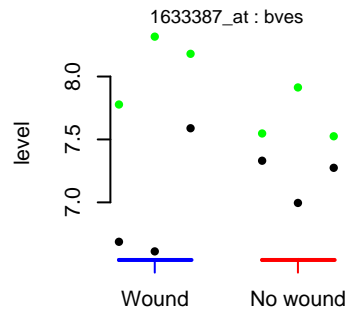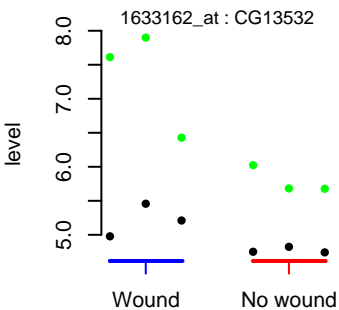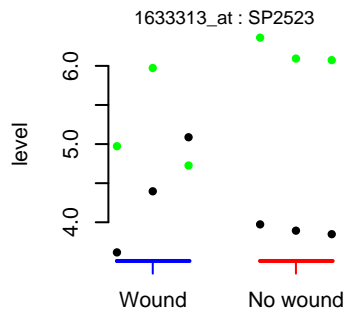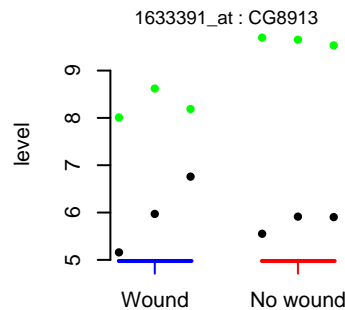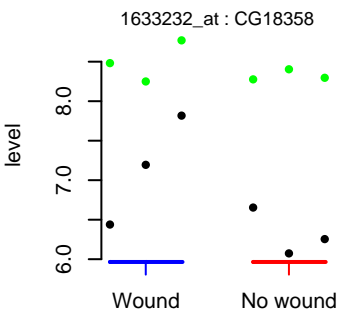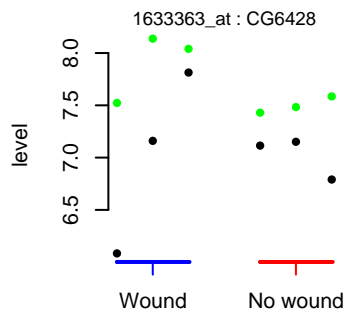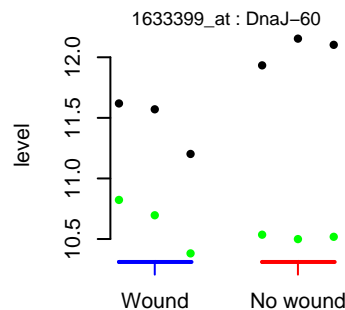

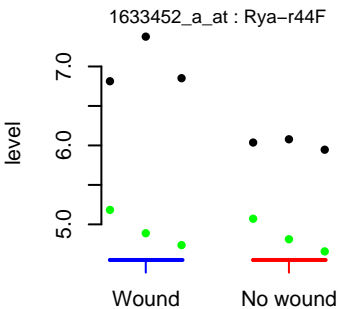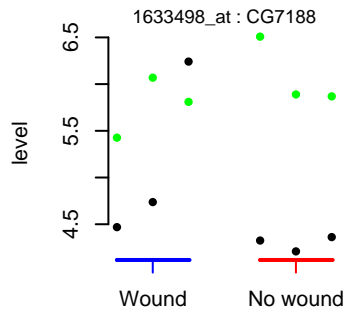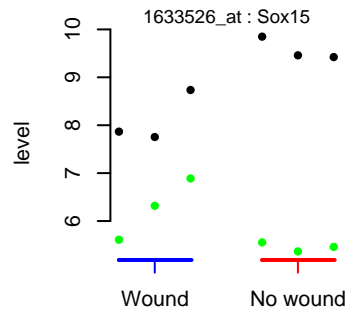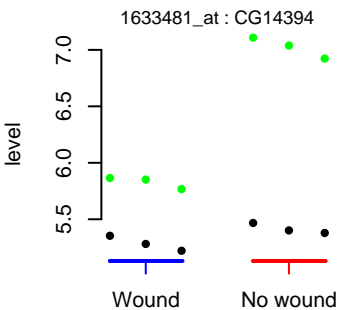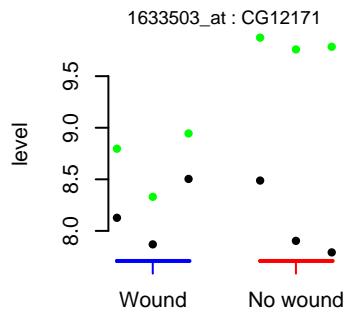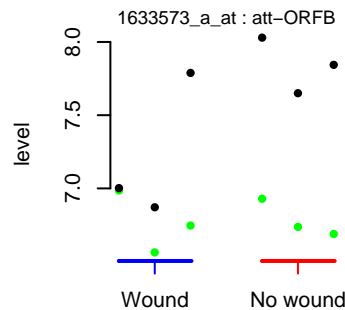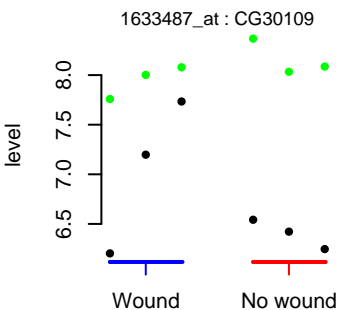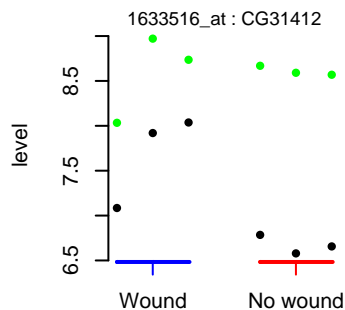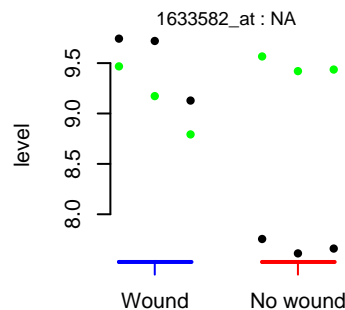

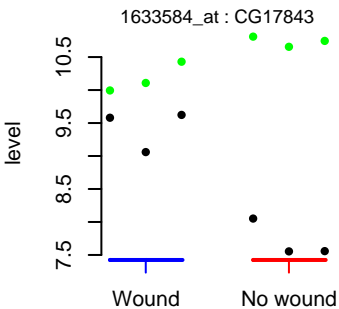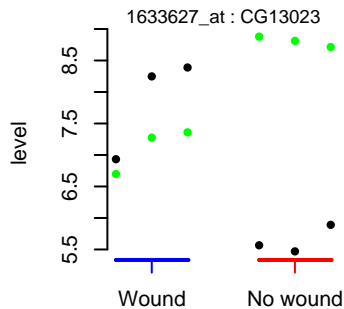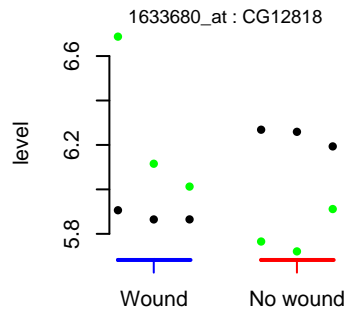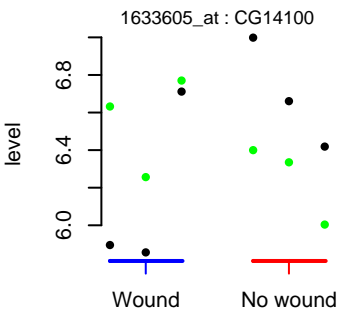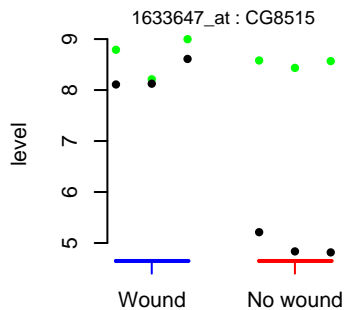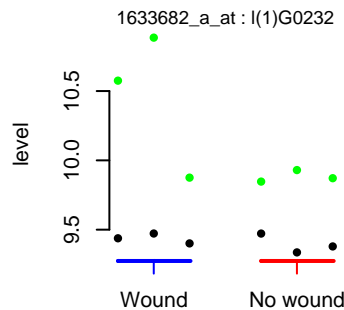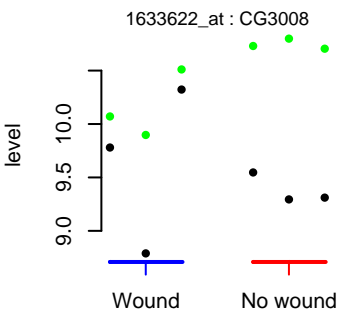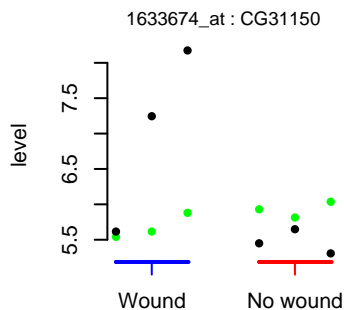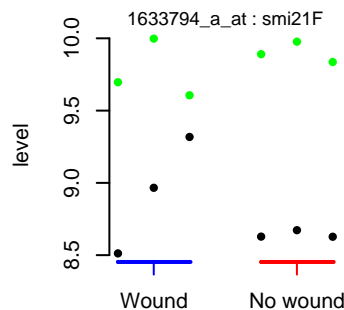

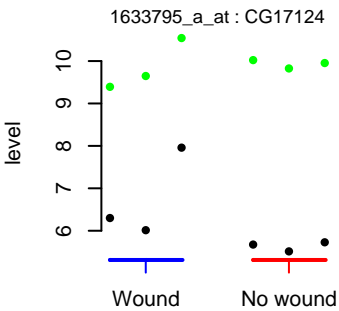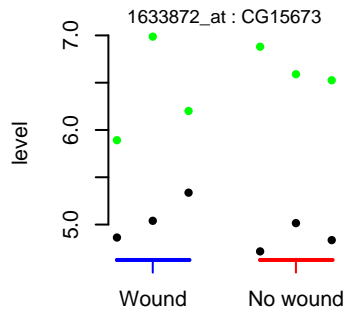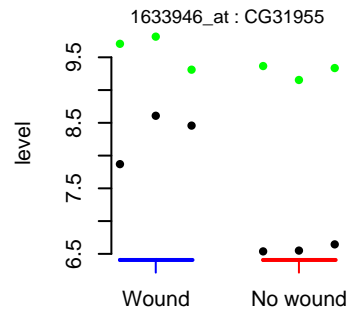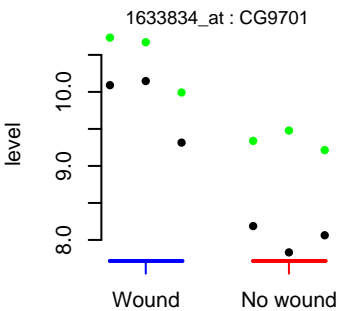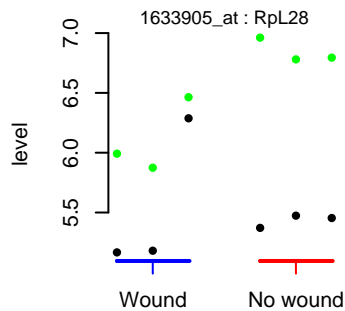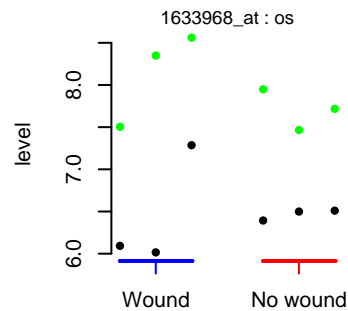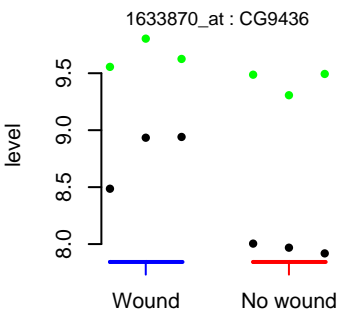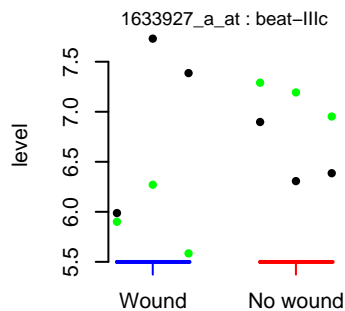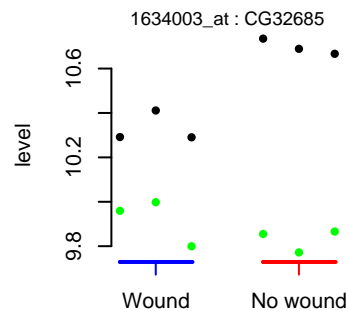

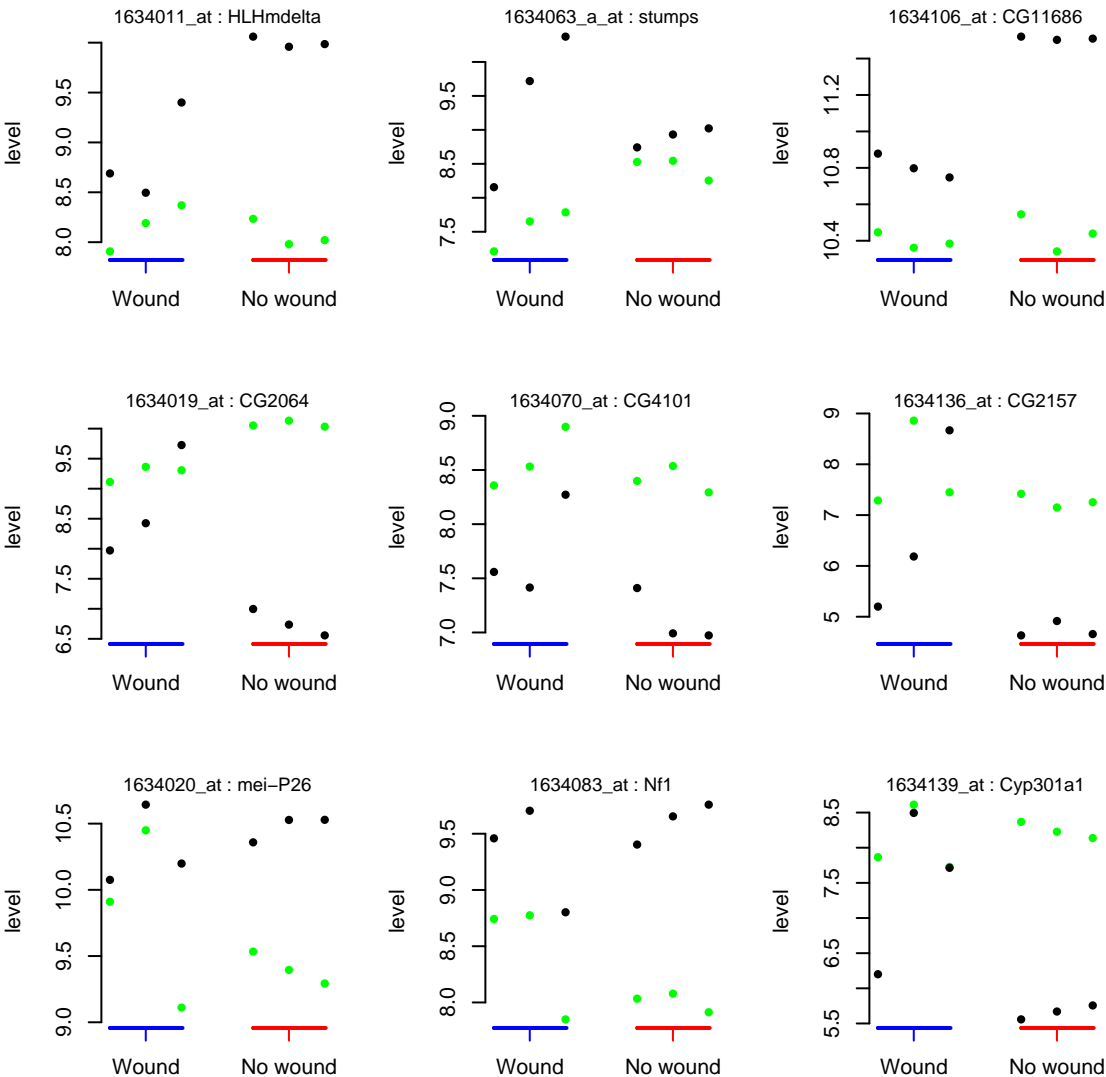

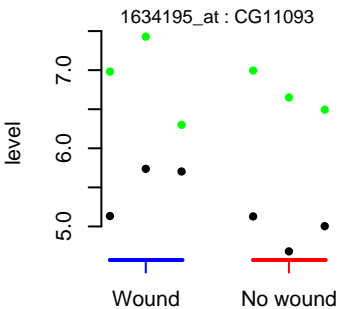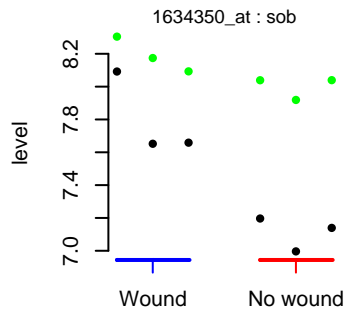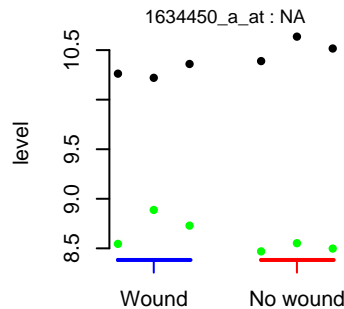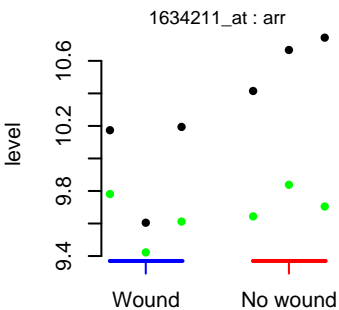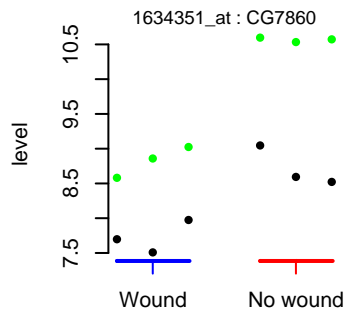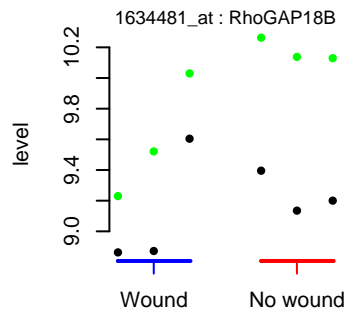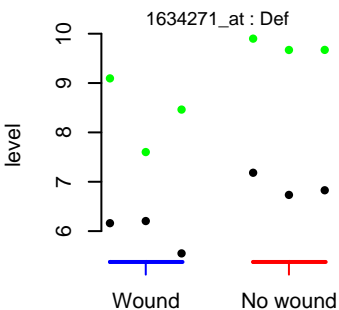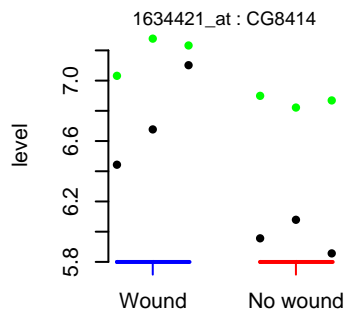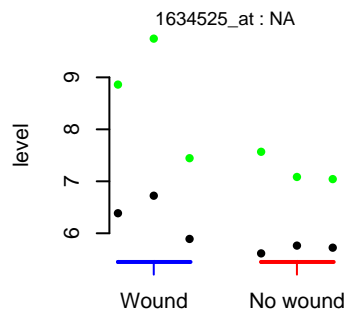

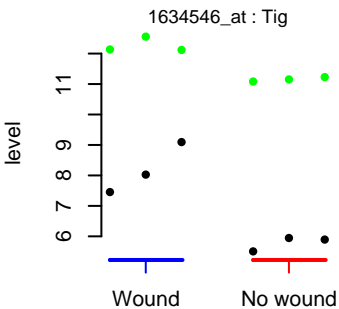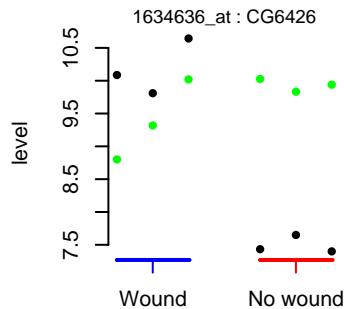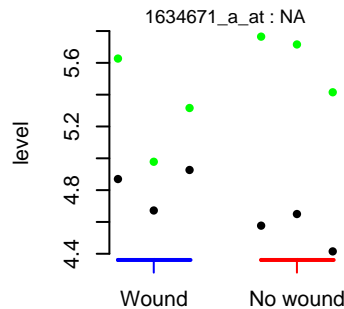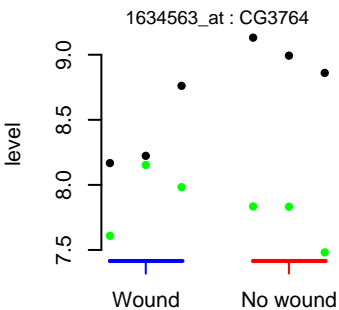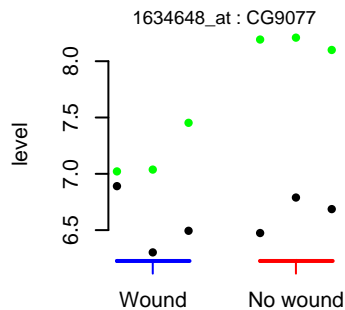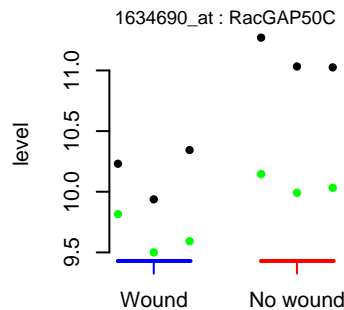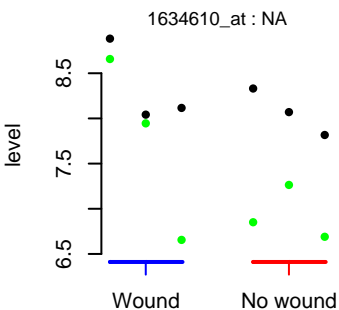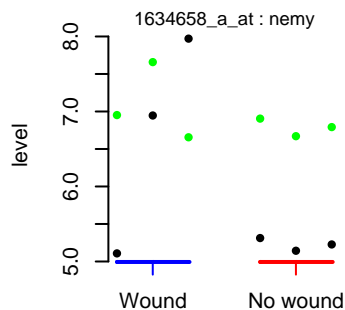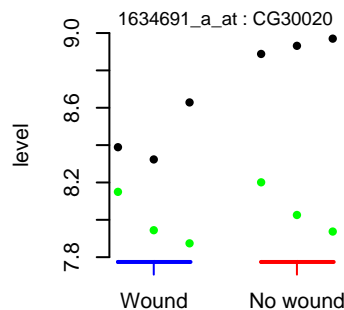

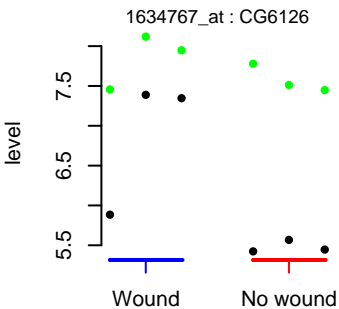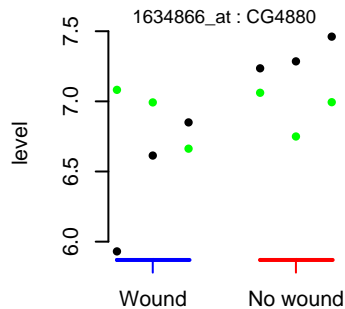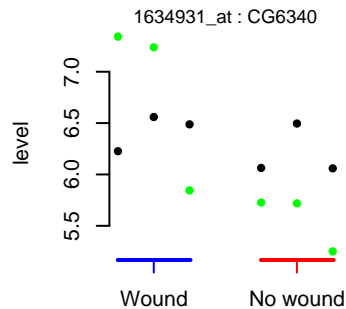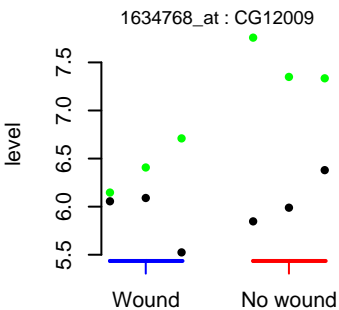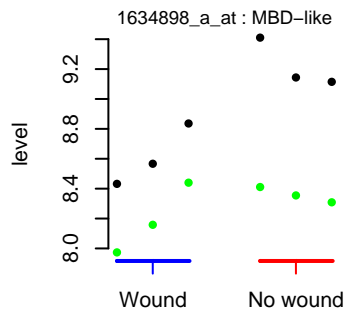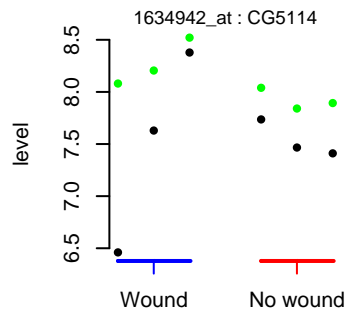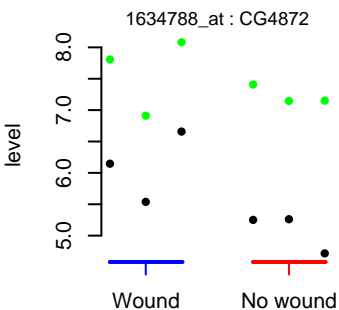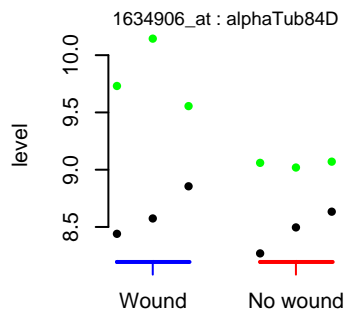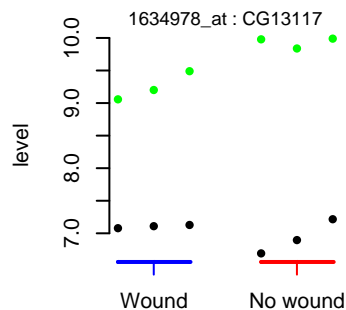

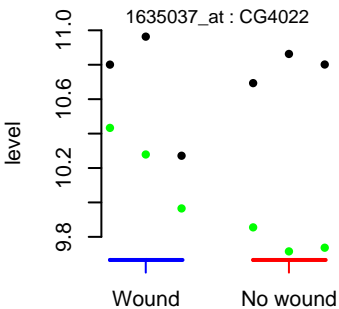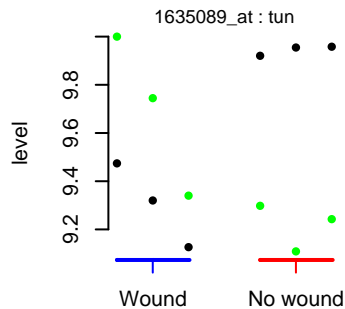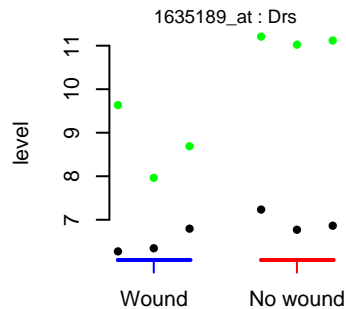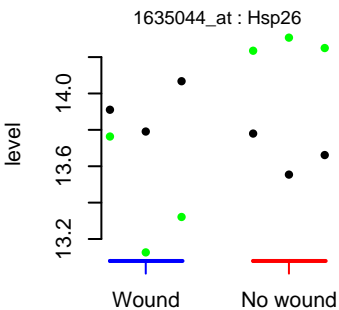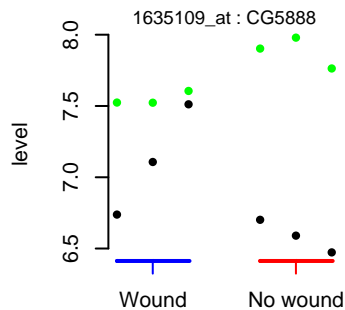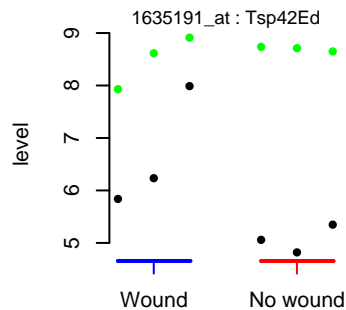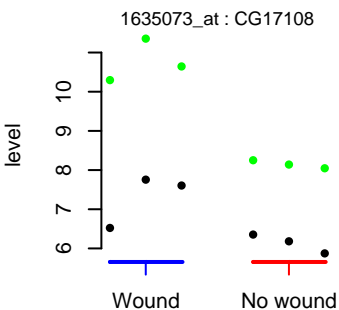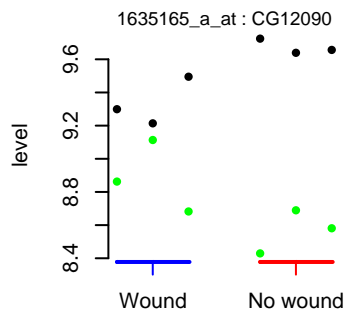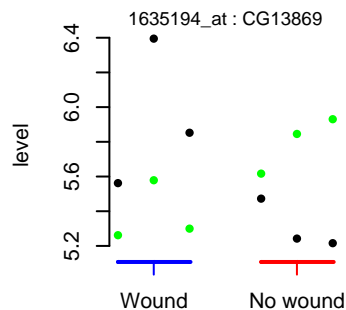



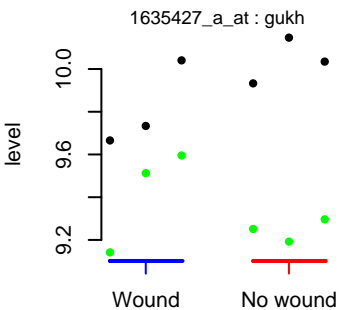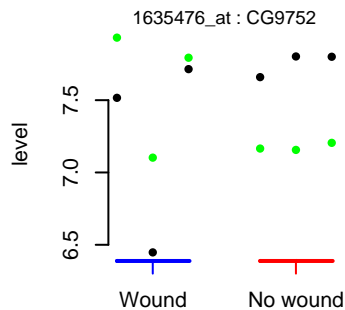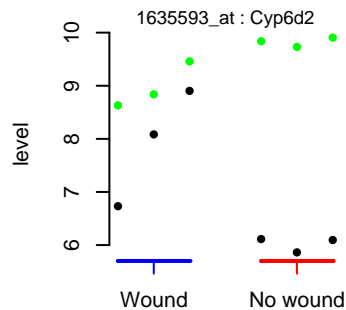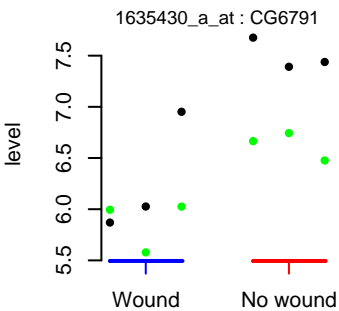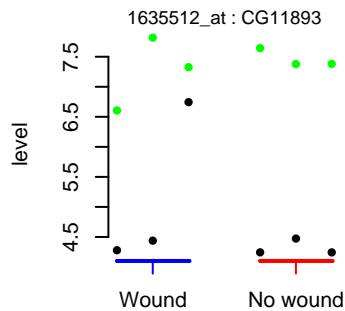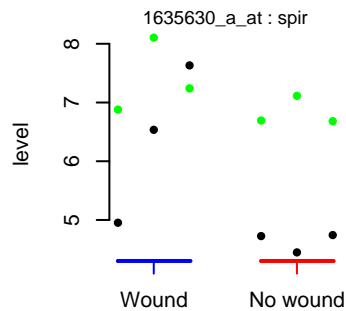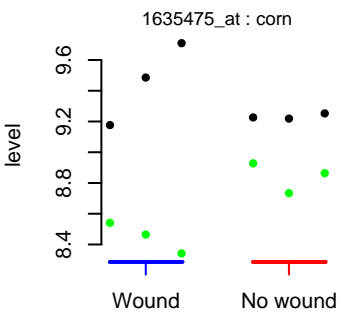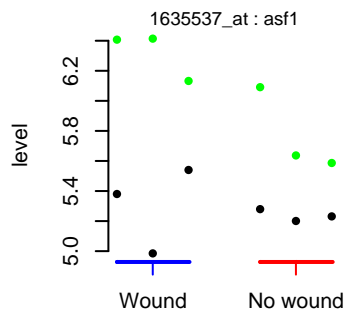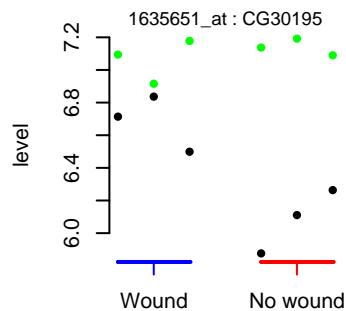

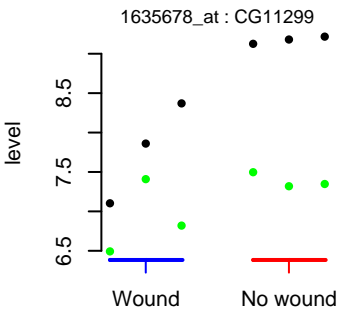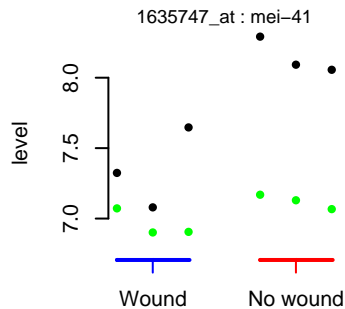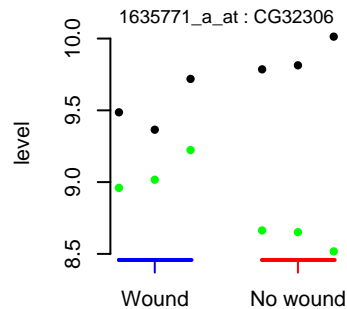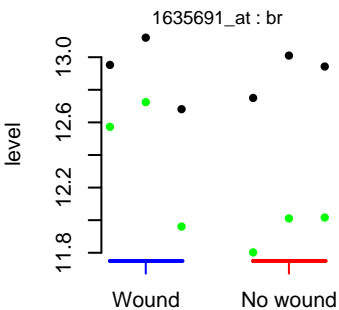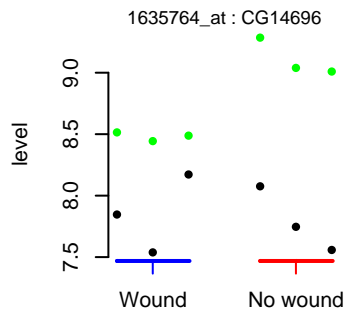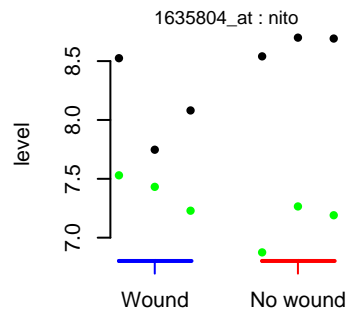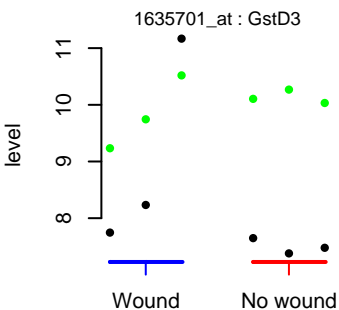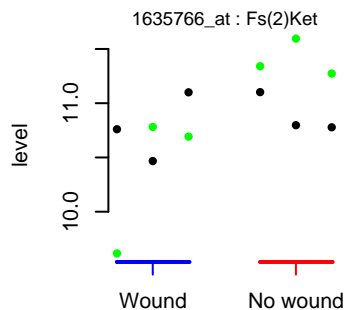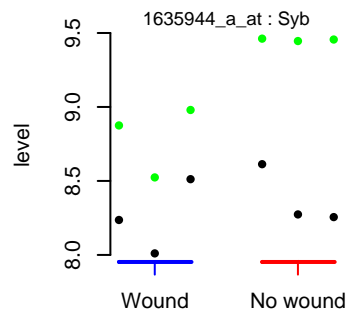

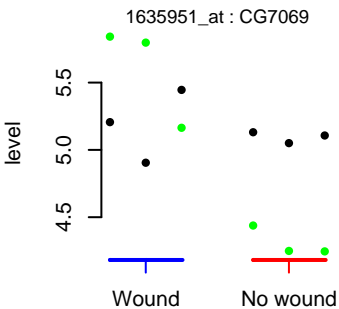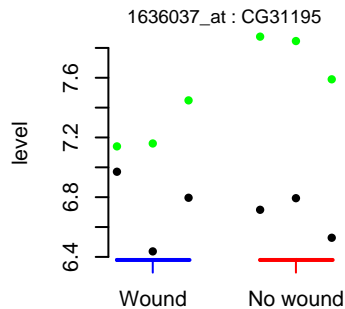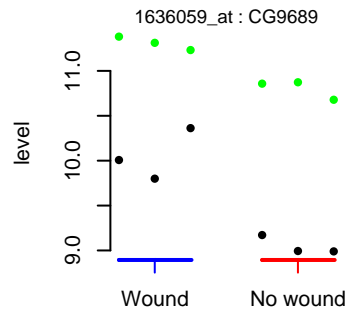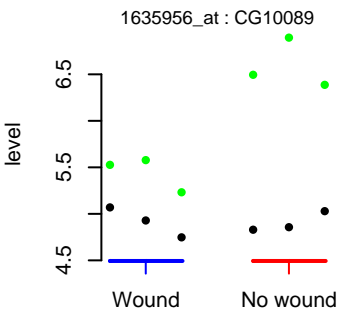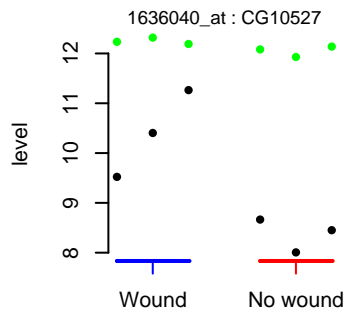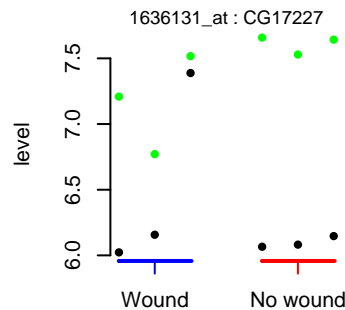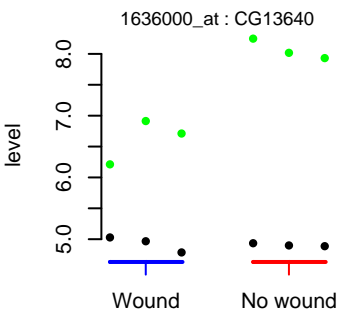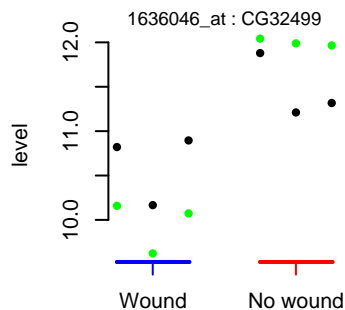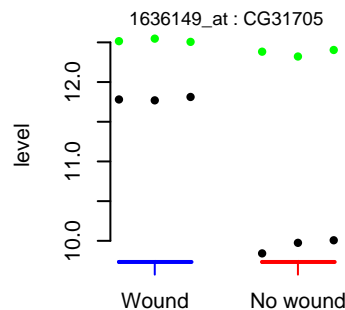

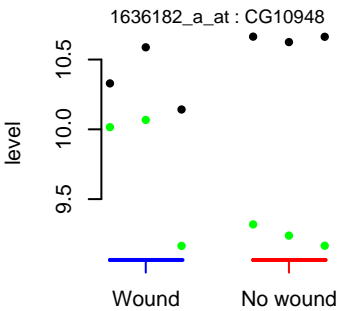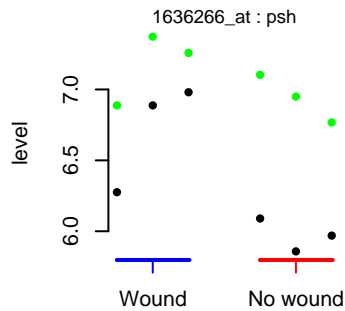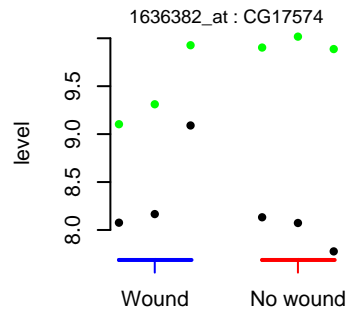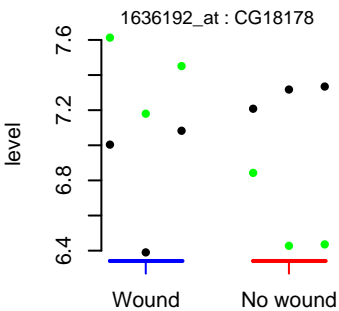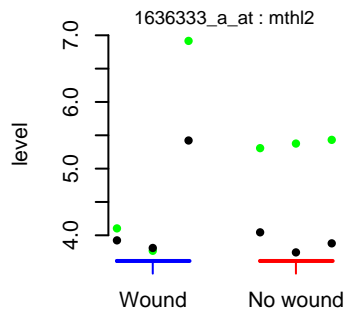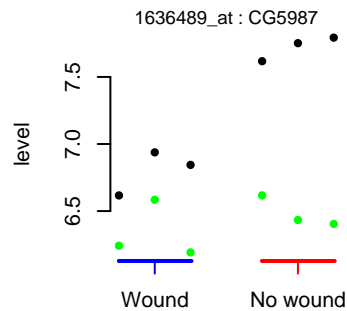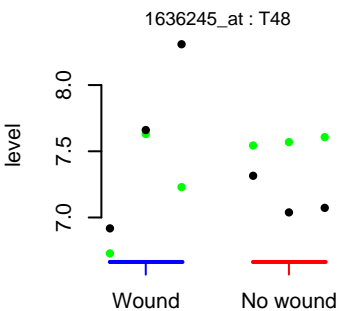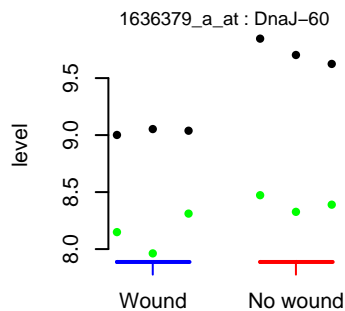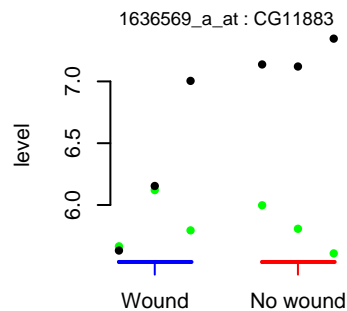

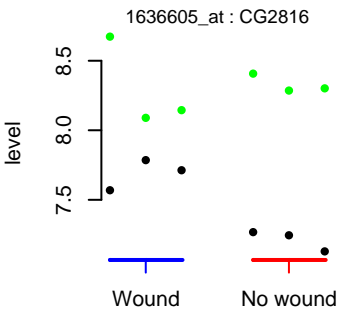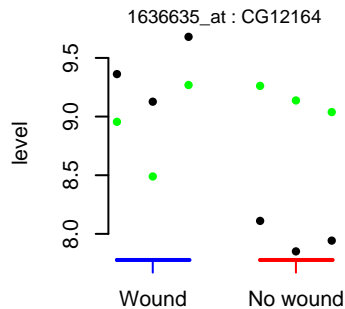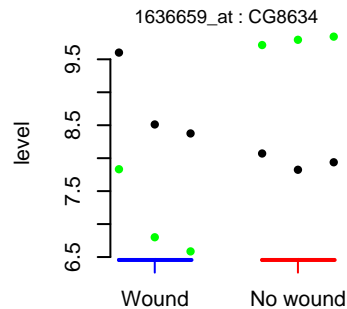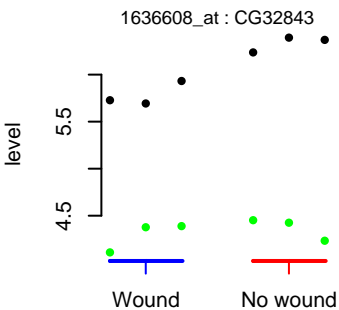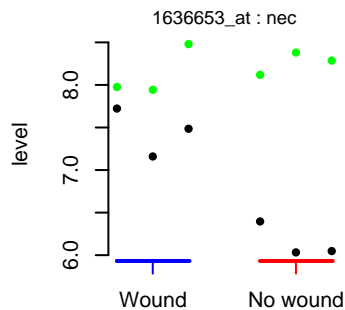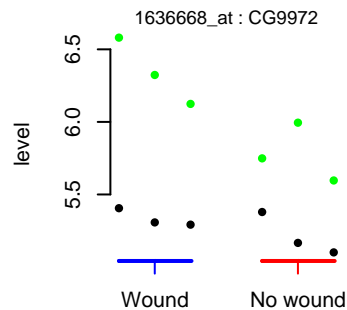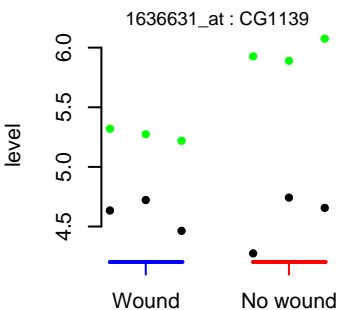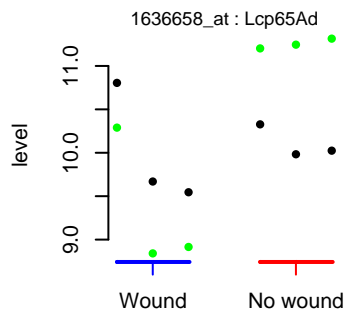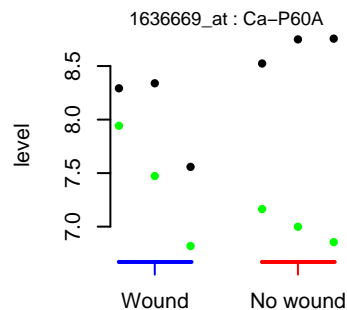

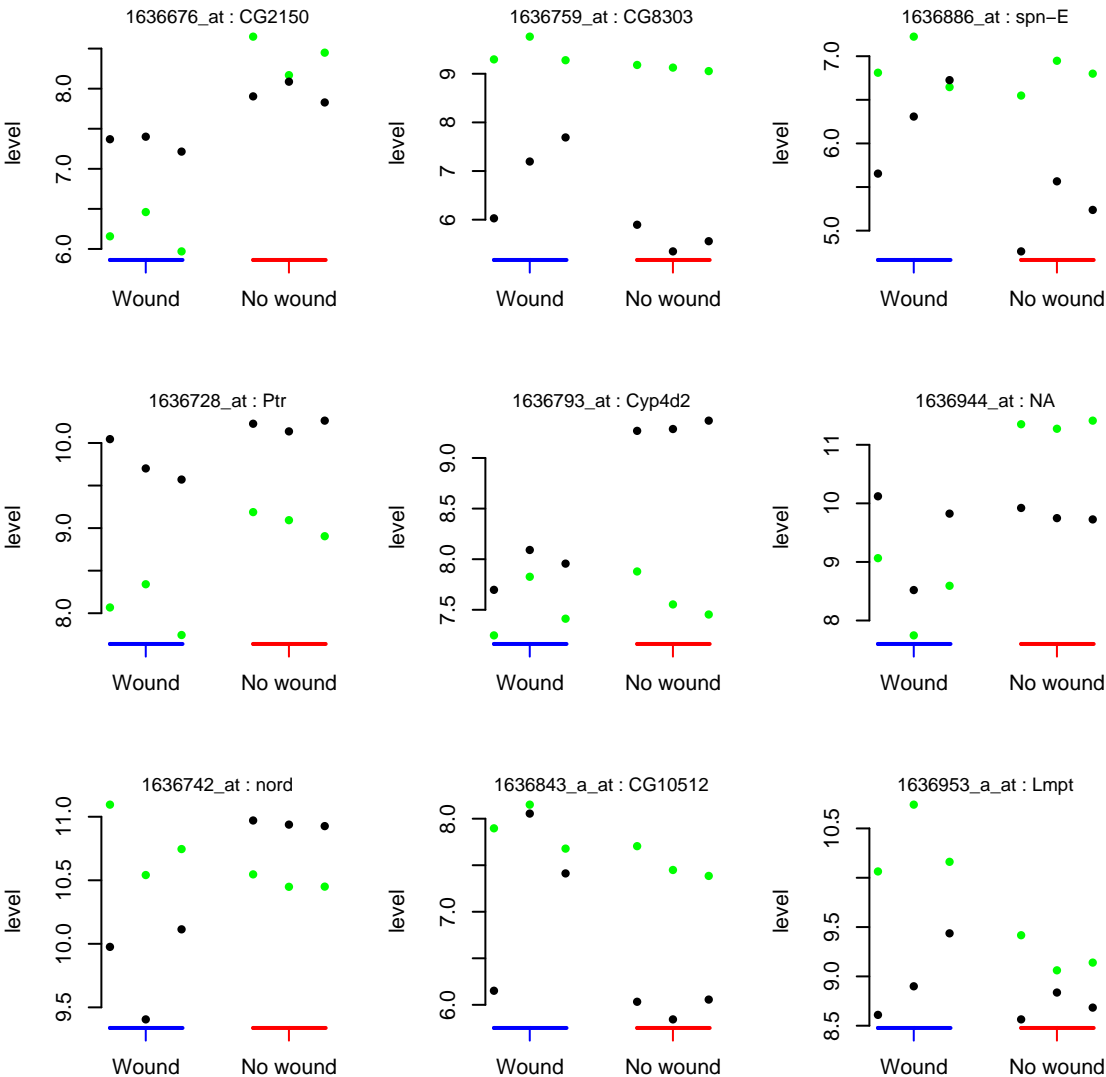

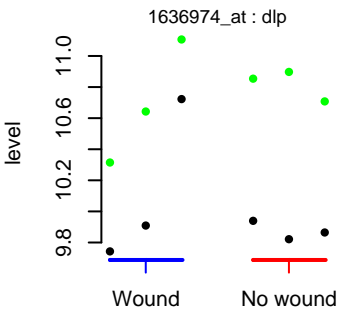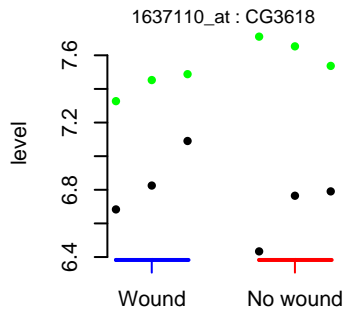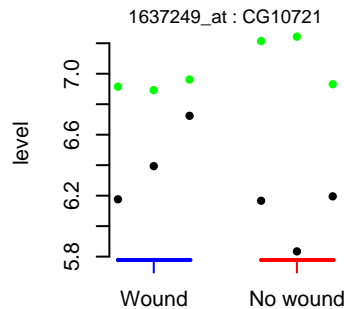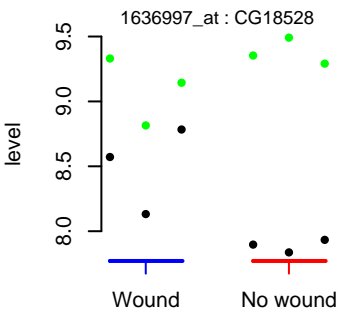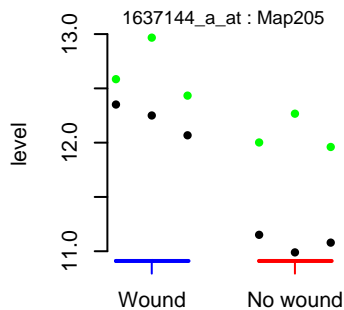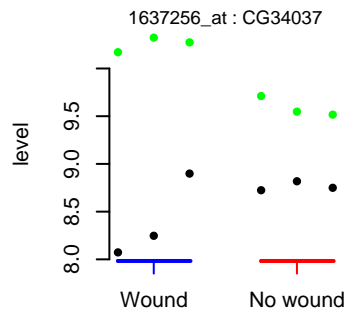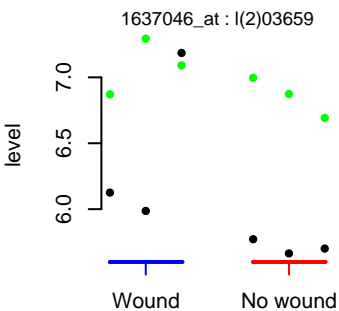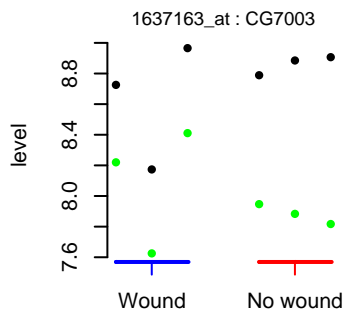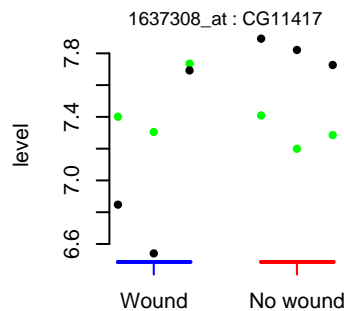

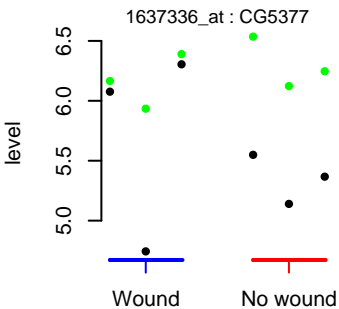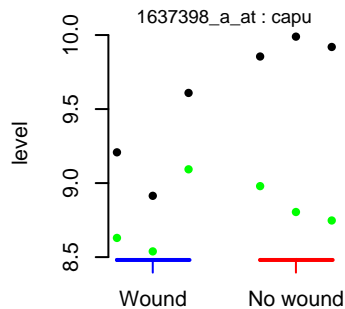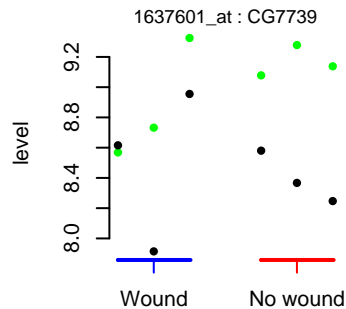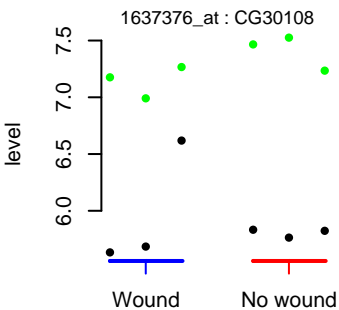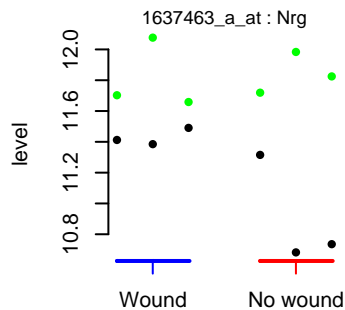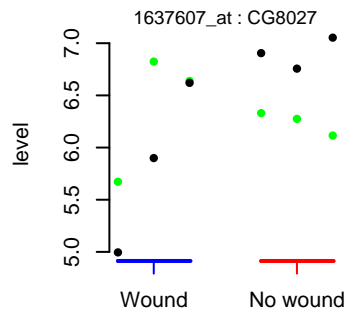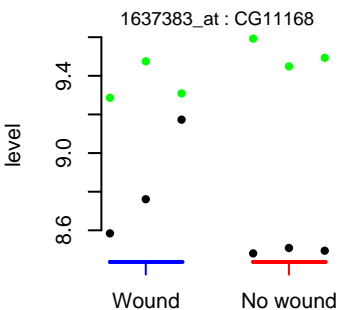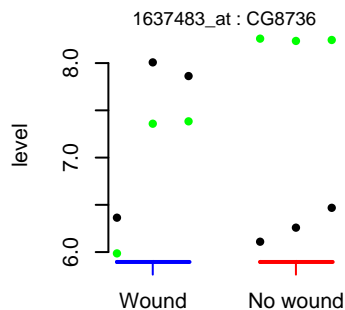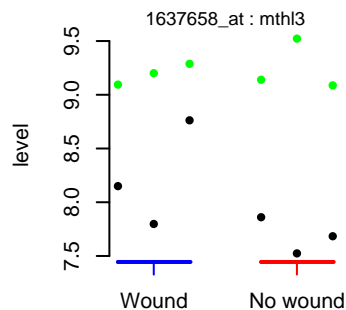

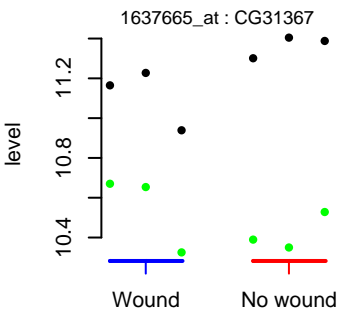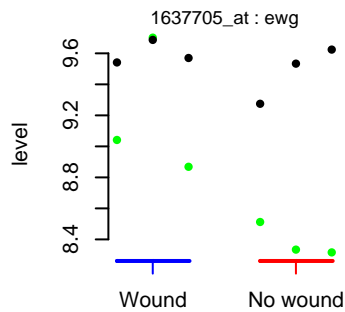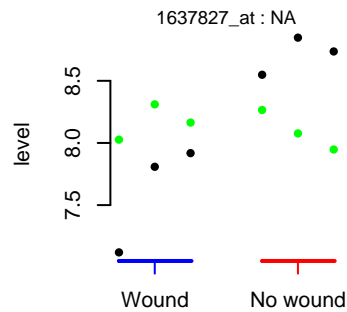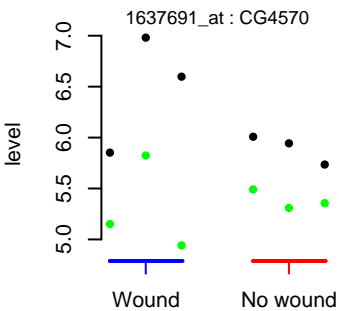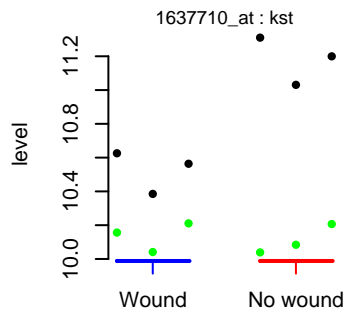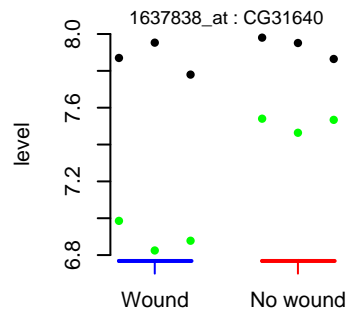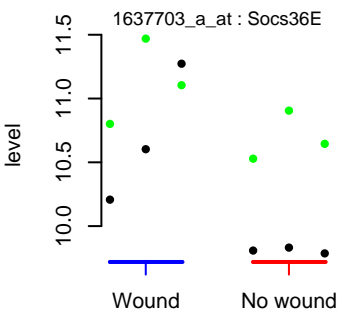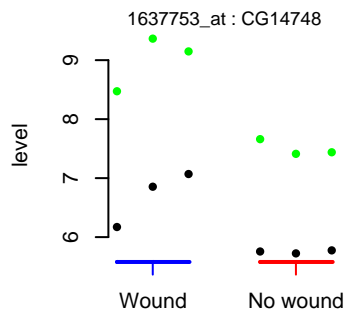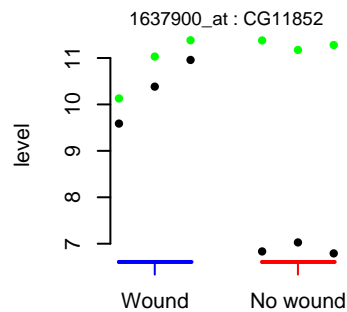

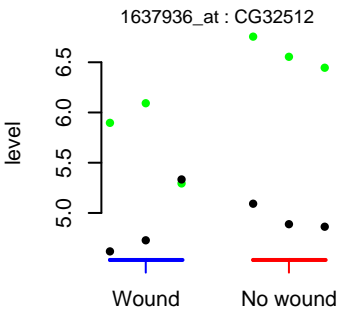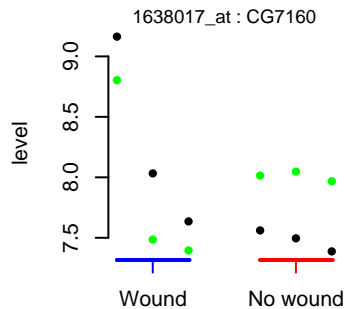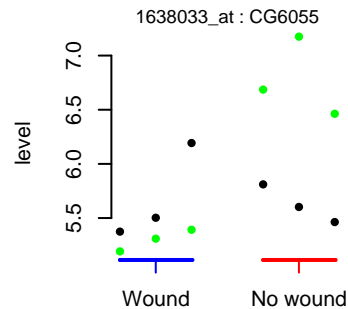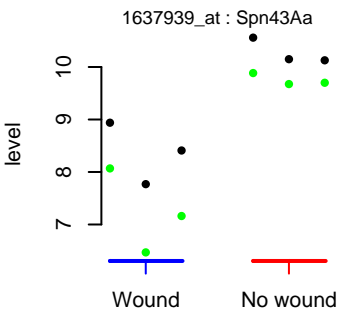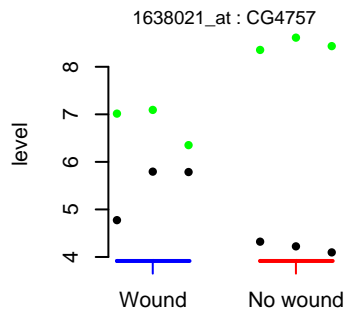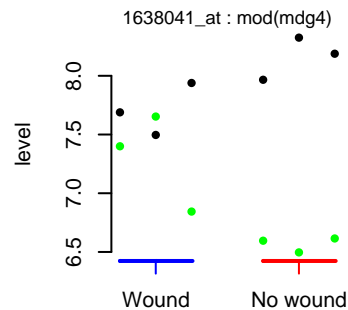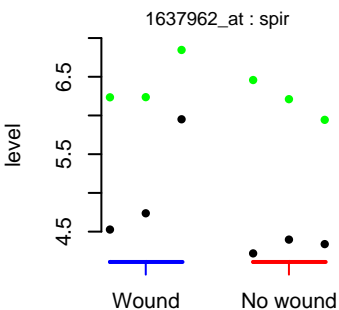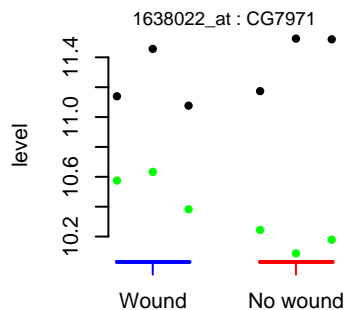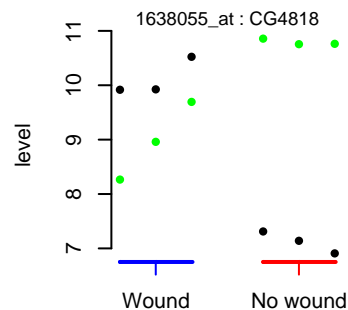

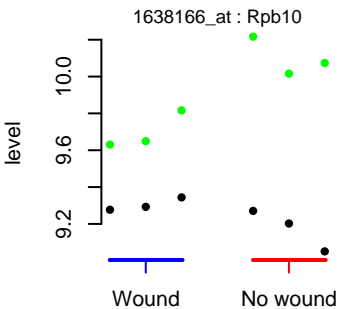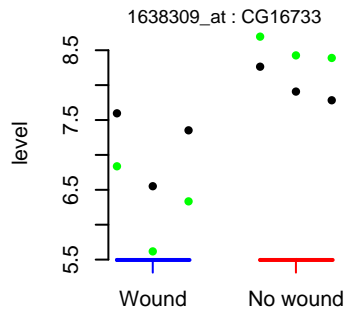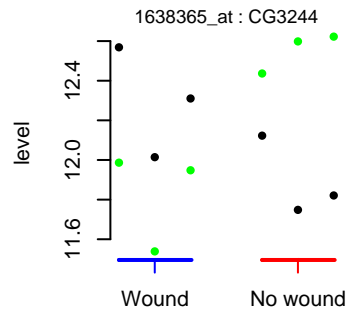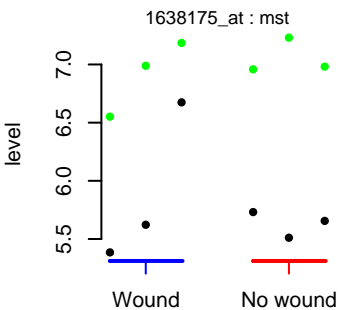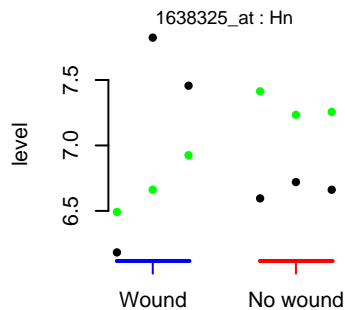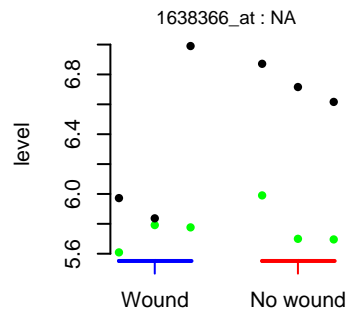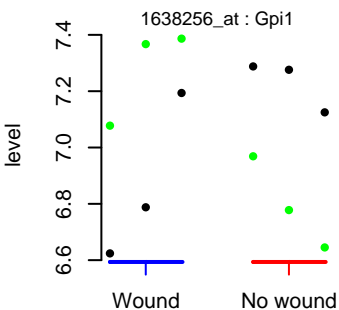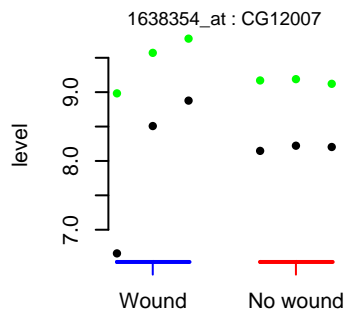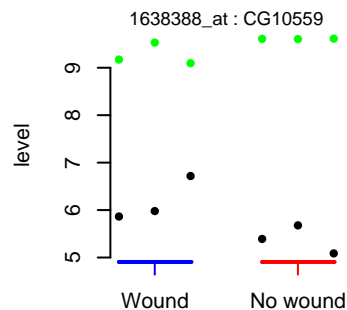

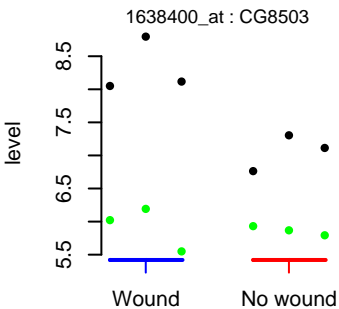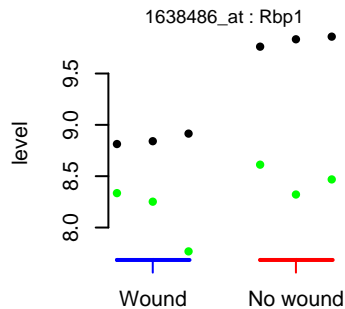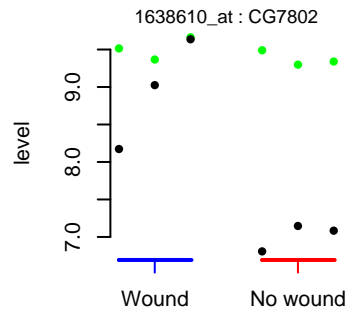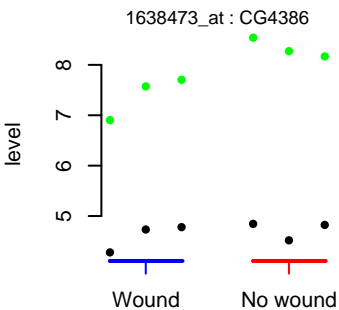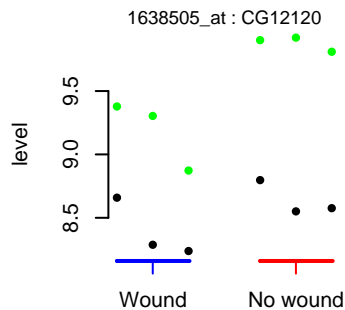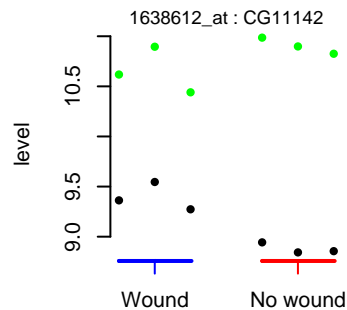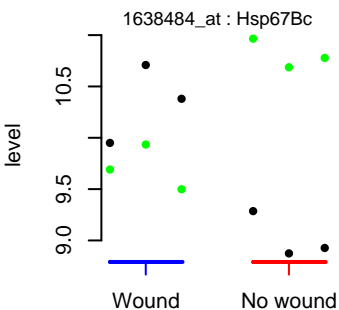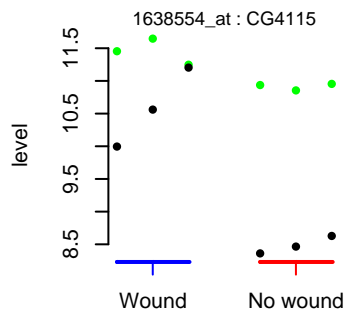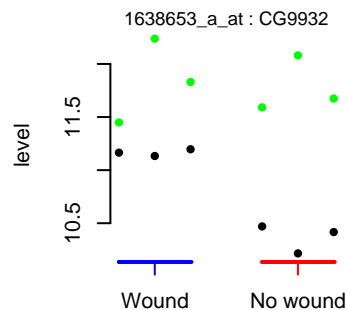

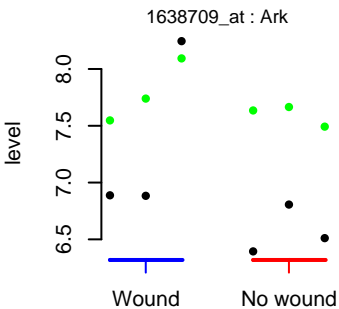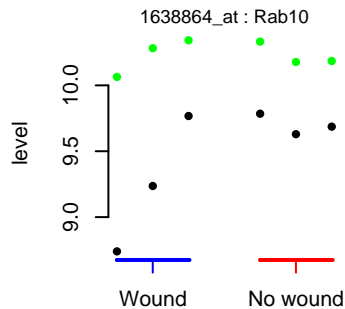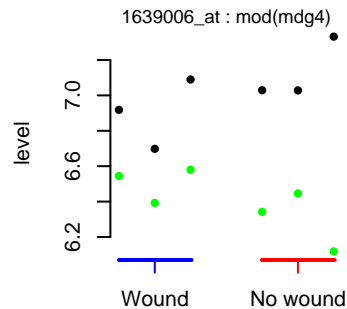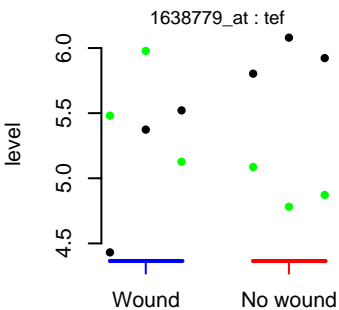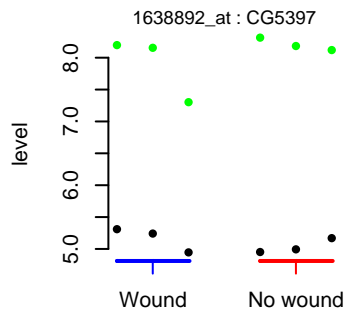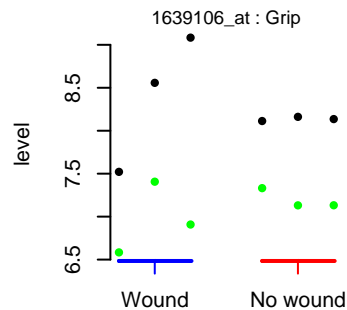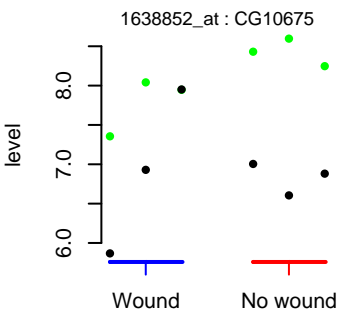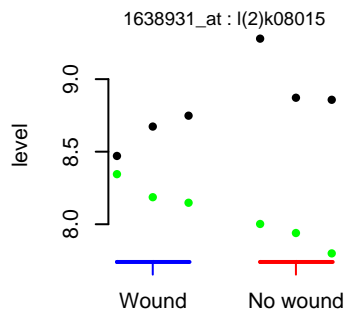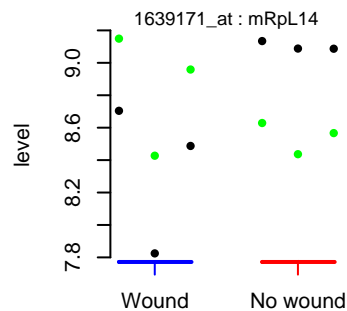

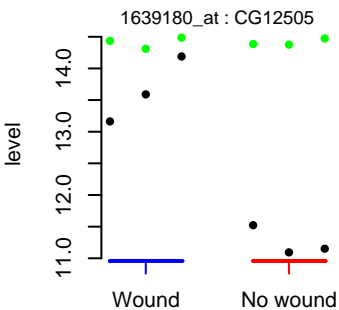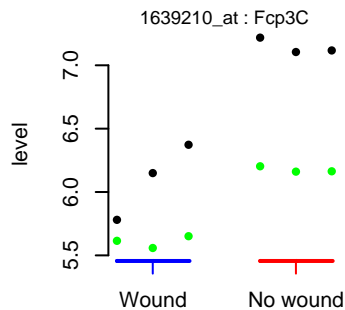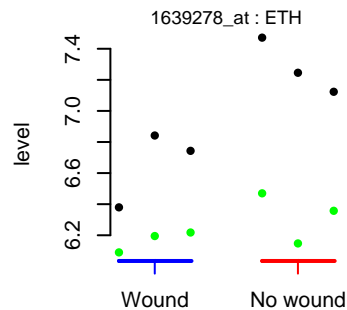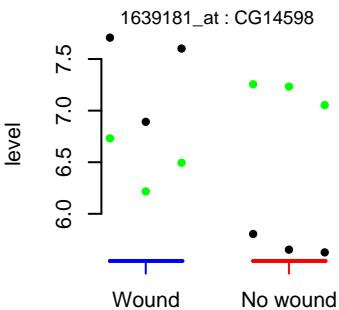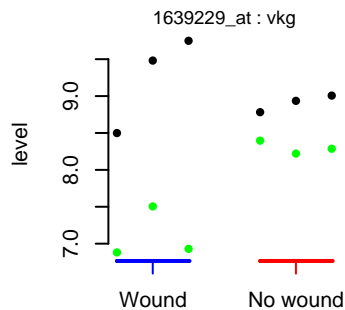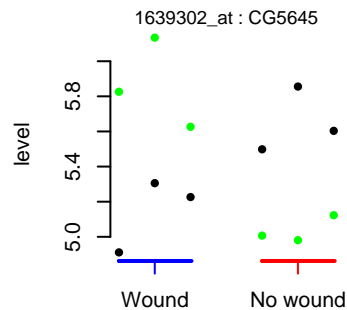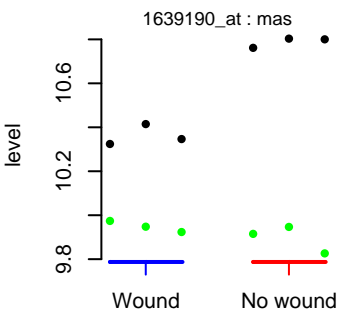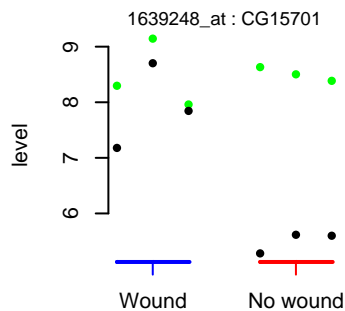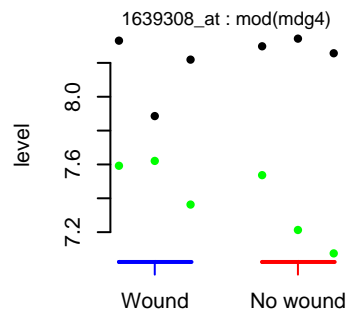

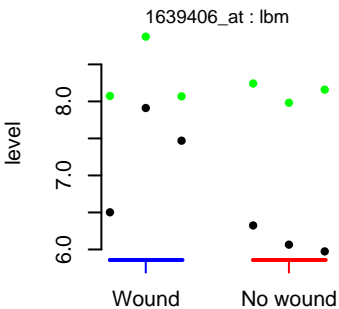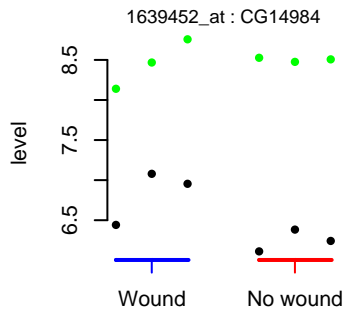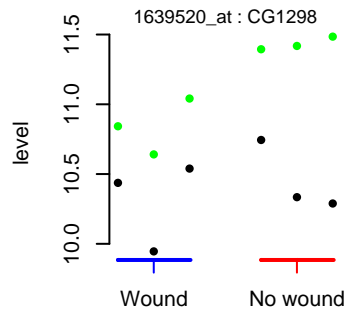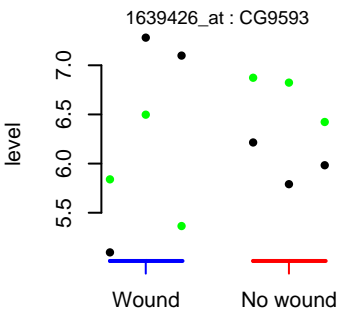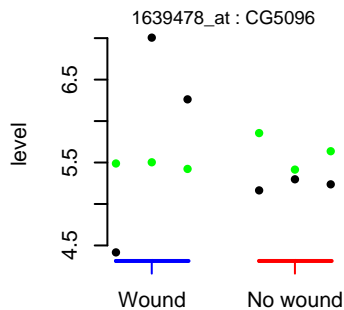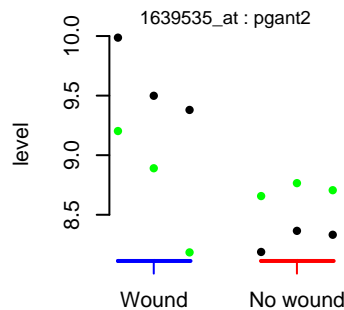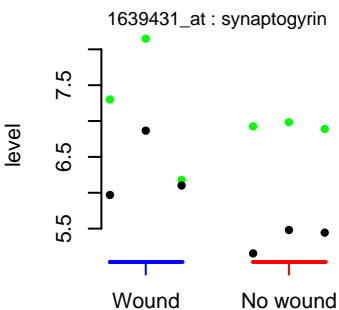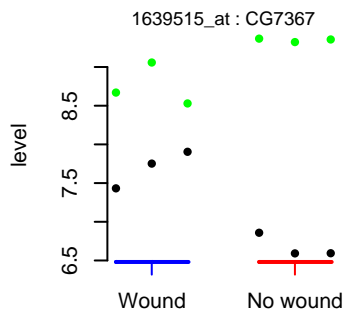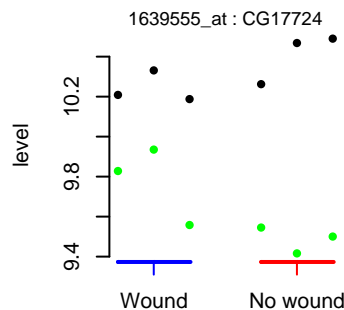

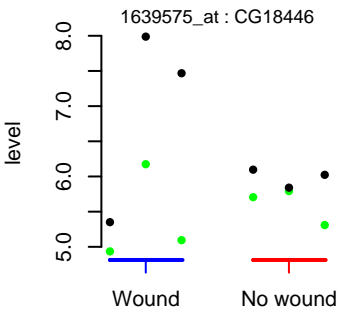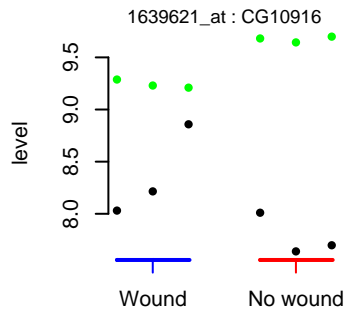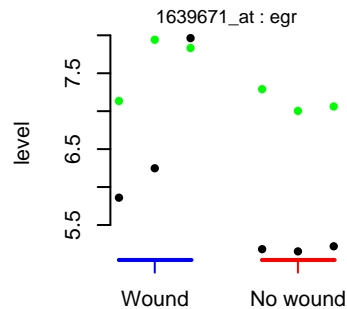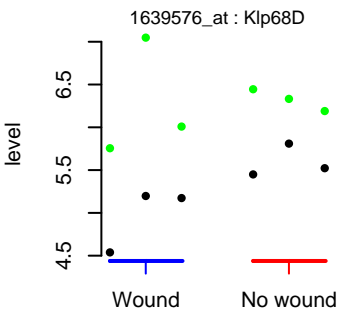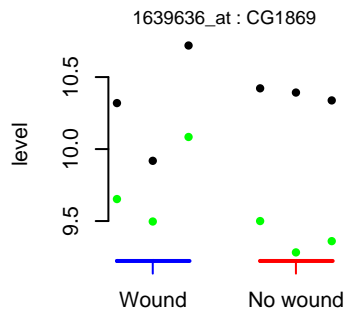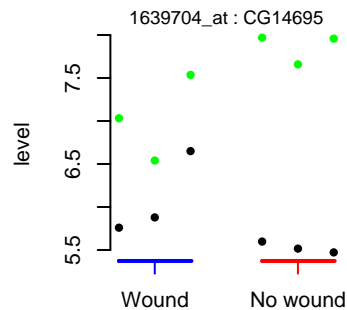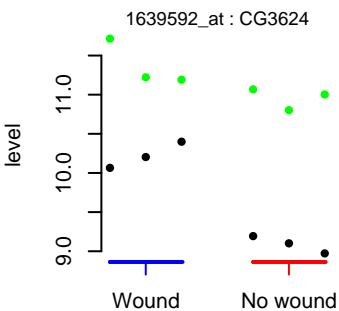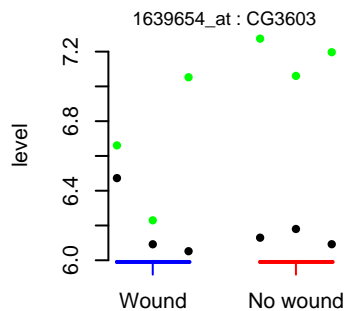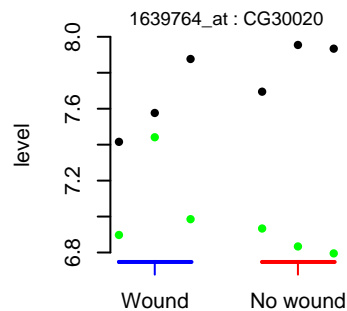

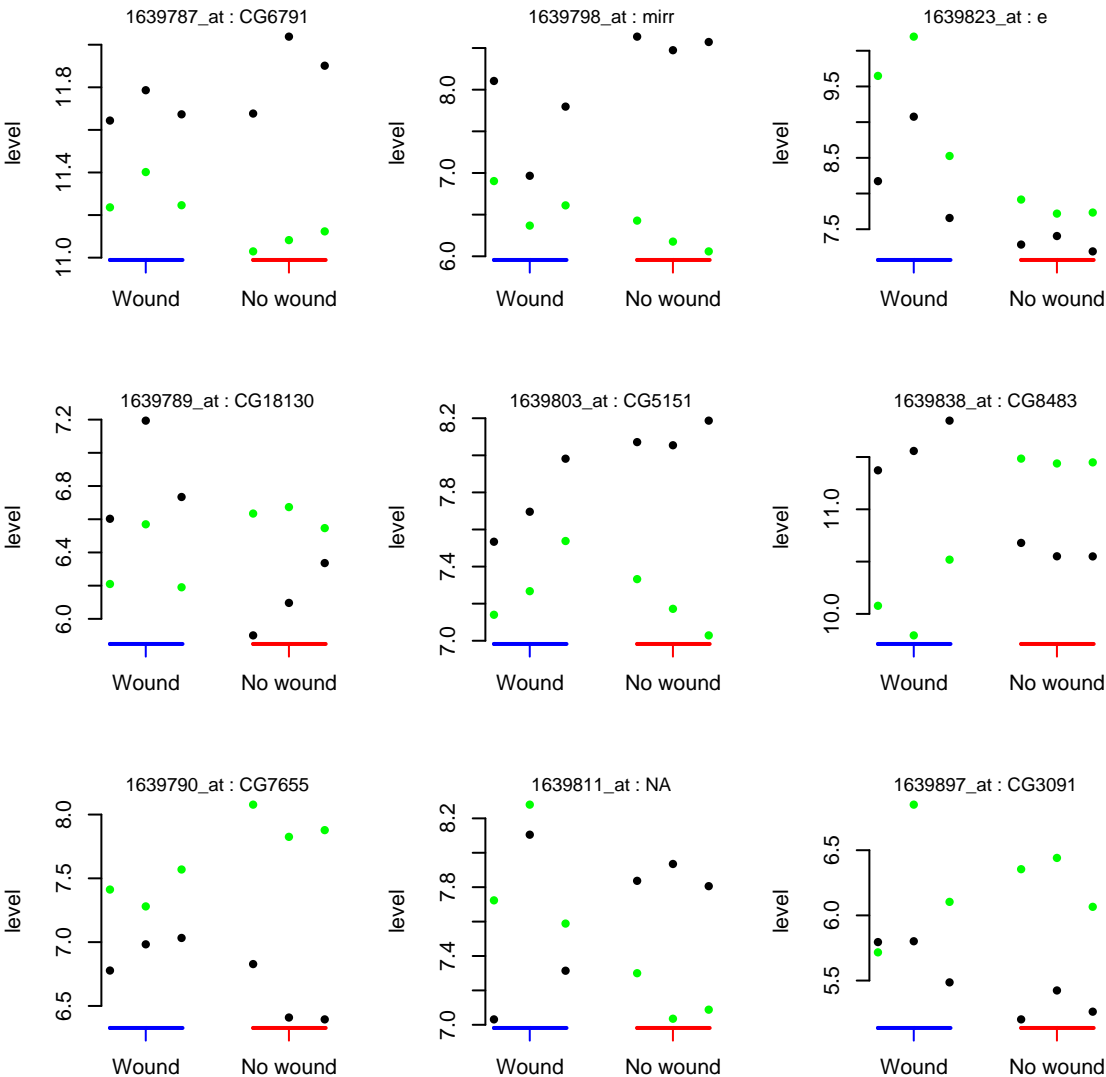

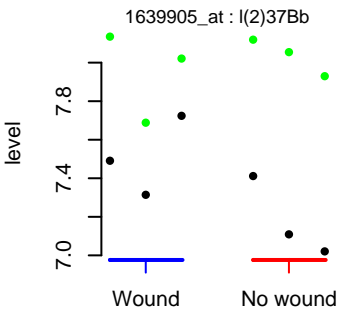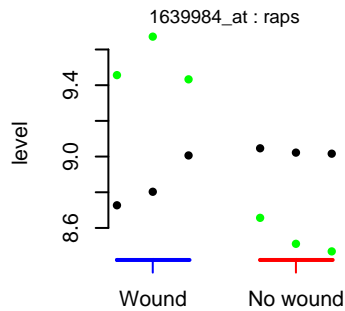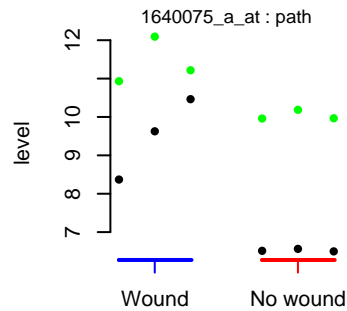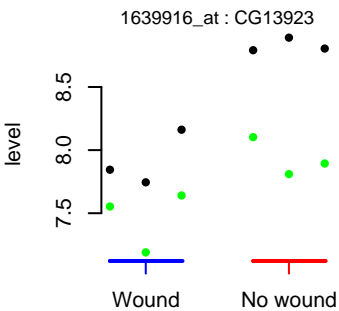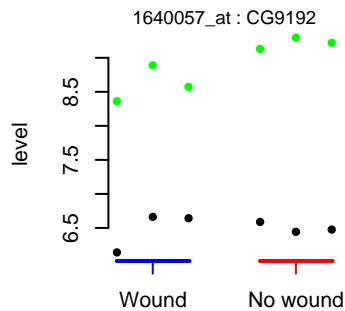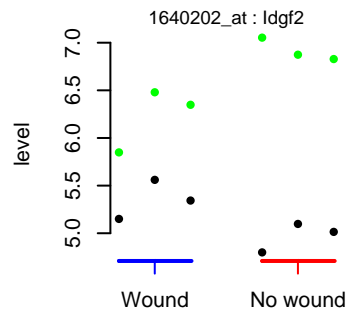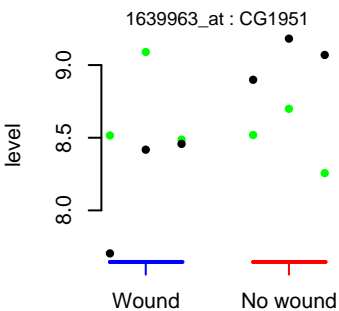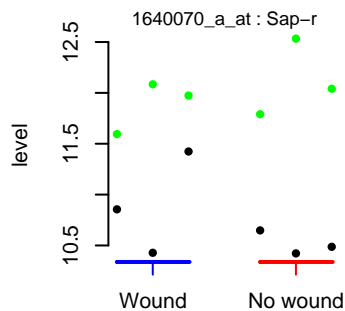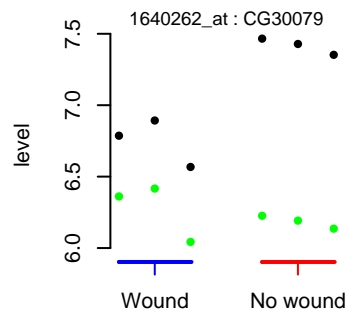

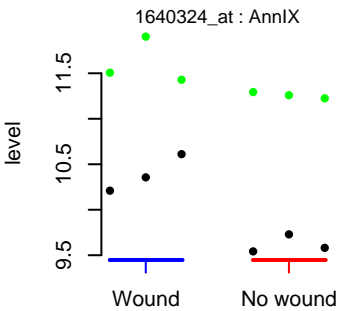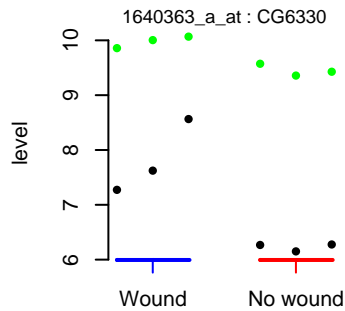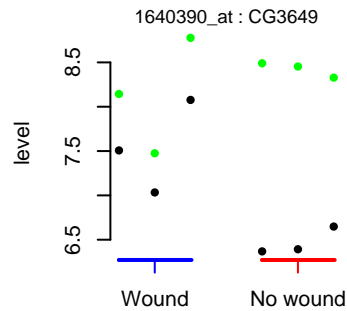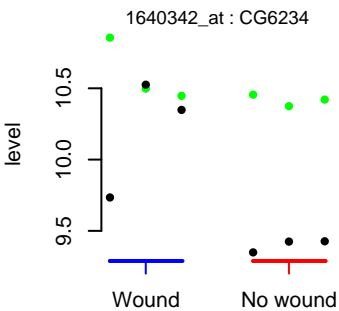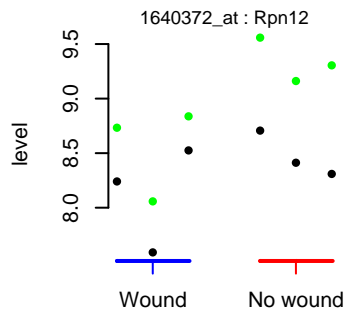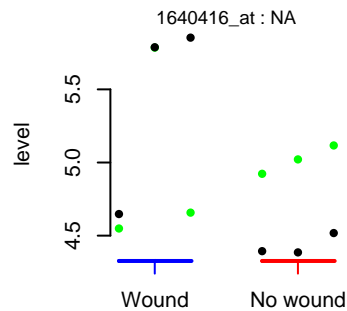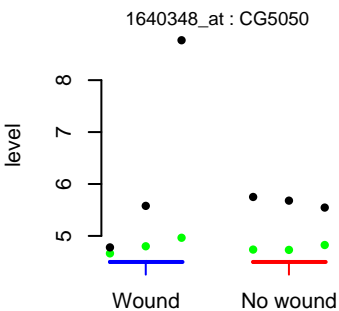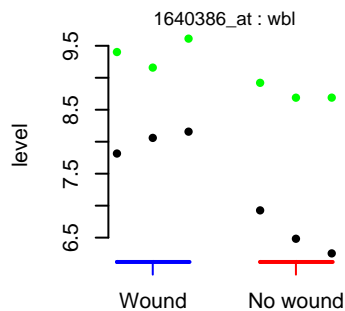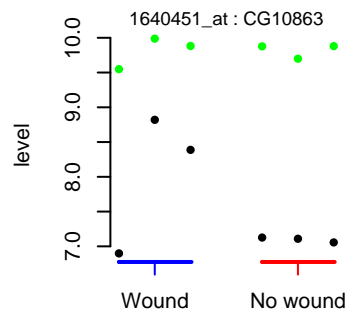

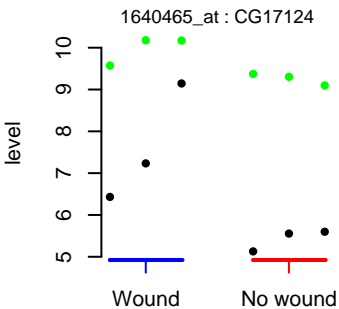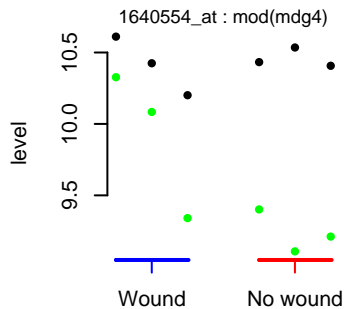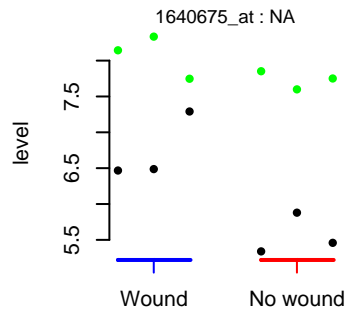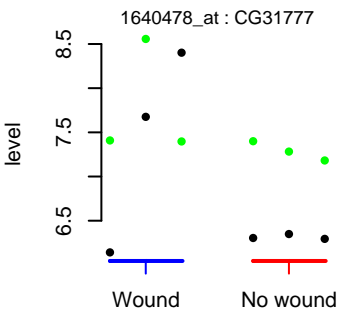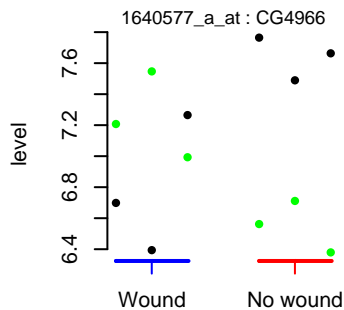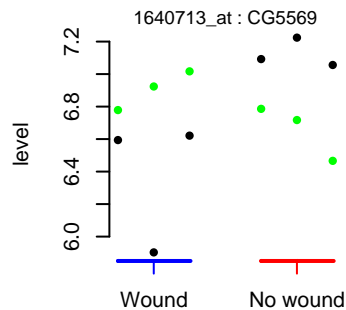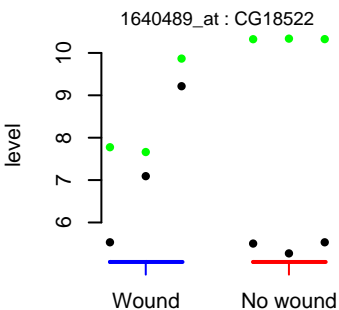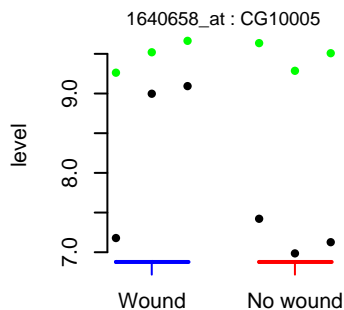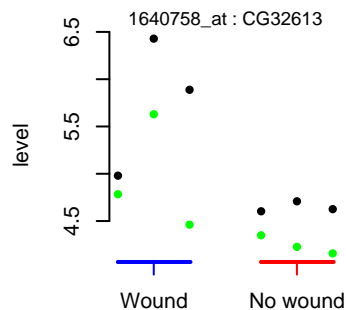

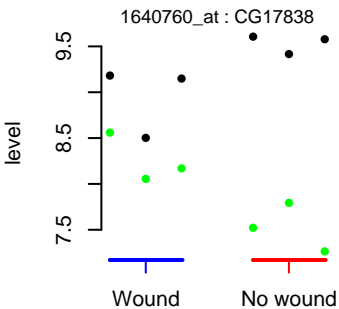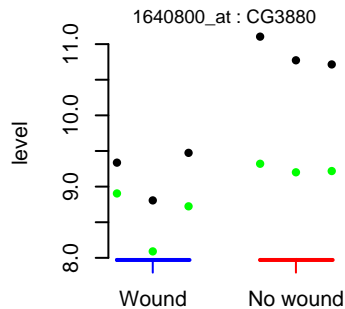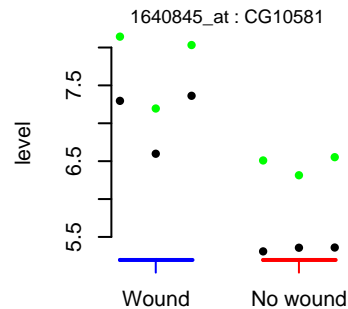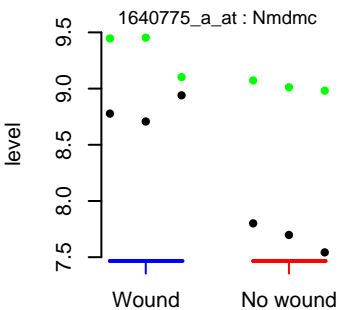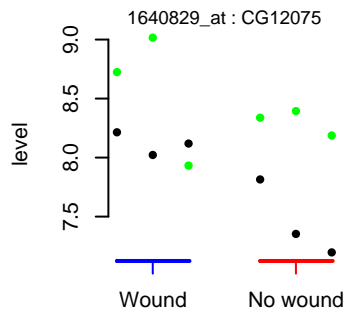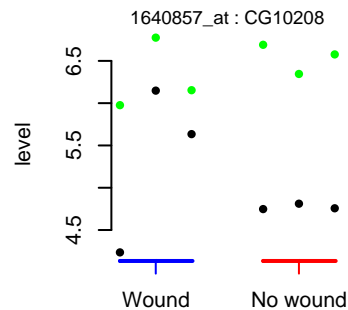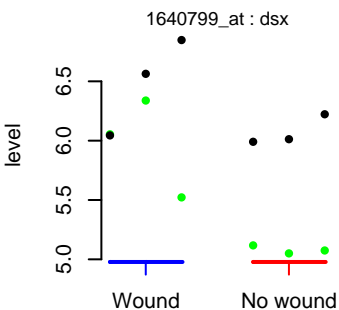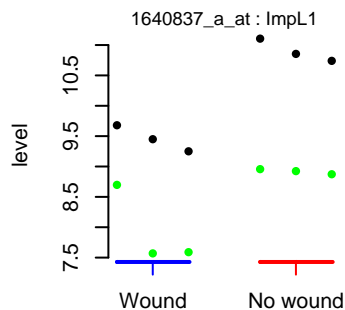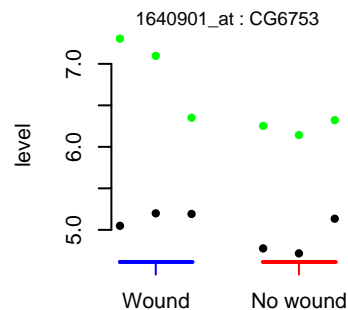

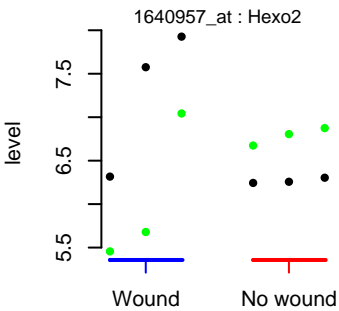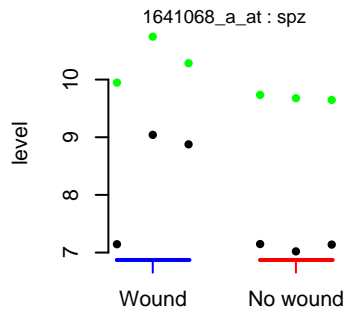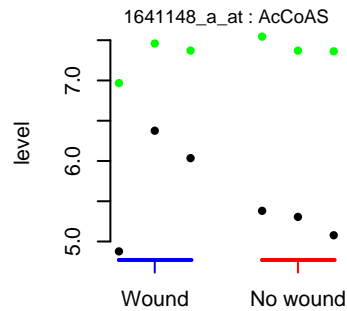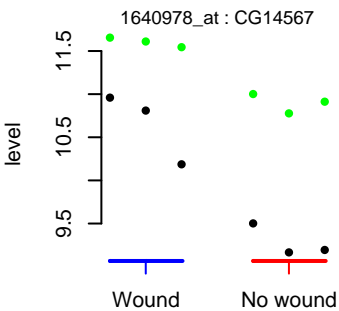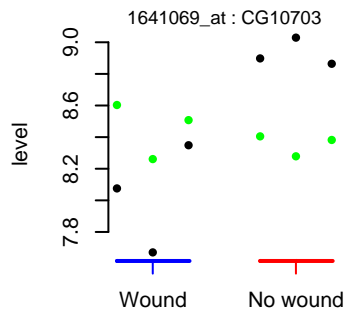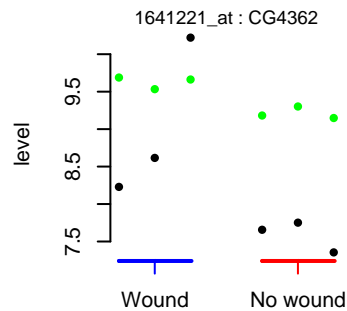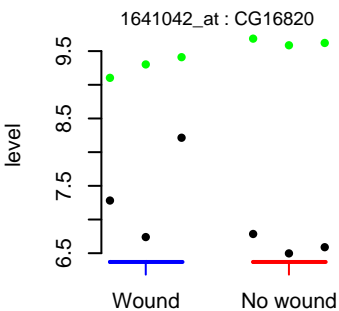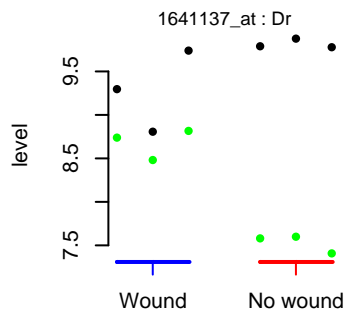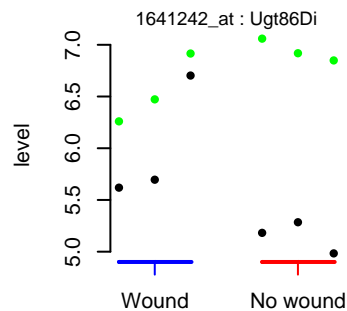



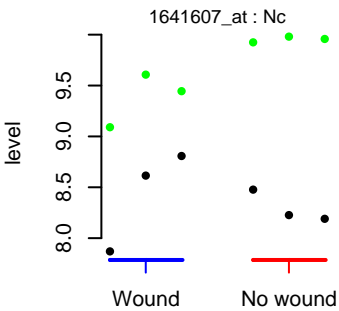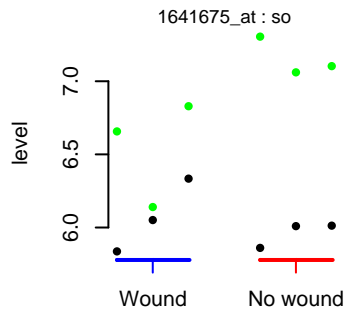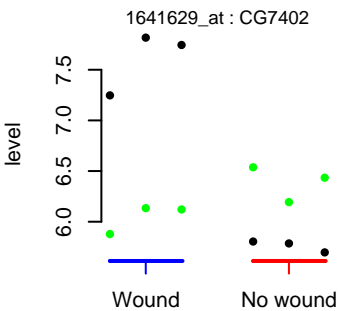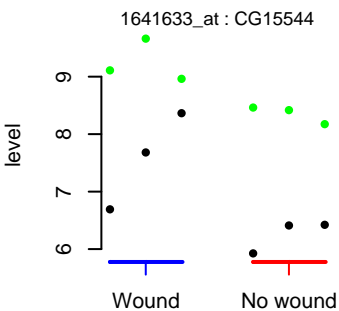

Supplement: S3 Fig — Overall changes in expression in wounded discs (for JNK-positive, negative or both cell types) relative to controls for individual genes in the W/NW/D subpopulation (genes differentially expressed in wounded and in non-wounded discs but distinctly in both conditions). Green spots show the level of expression for each replica in JNK-positive cells. Black spots show the levels of expression for each replica in JNK-negative cells. (PDF) [file pgen.1004965.s004.pdf]

## **Supplementary Figure 4**

Clustering by absolute expression value

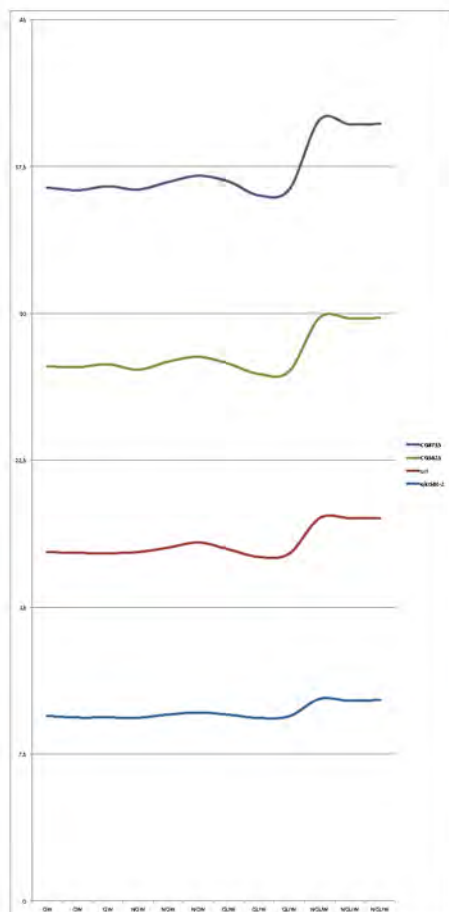

1112

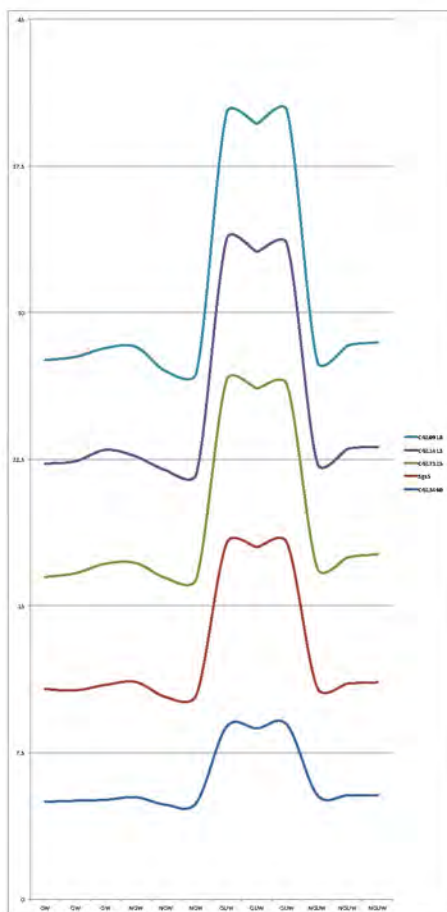

1121

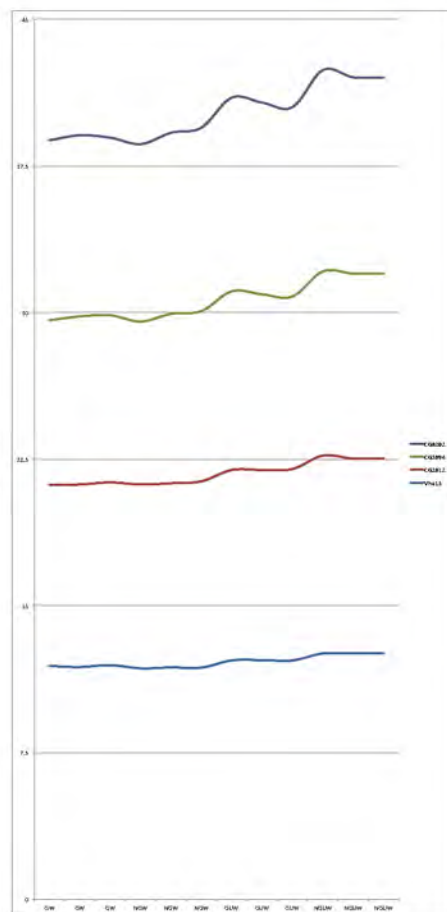

1123

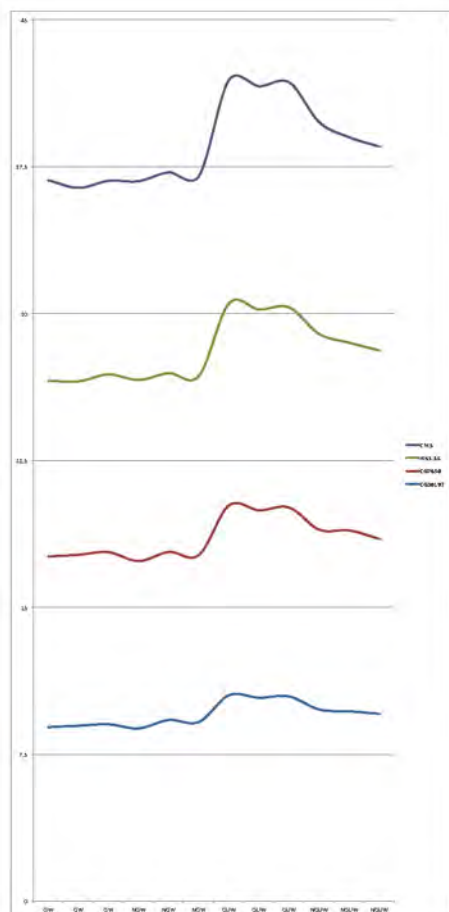

1132

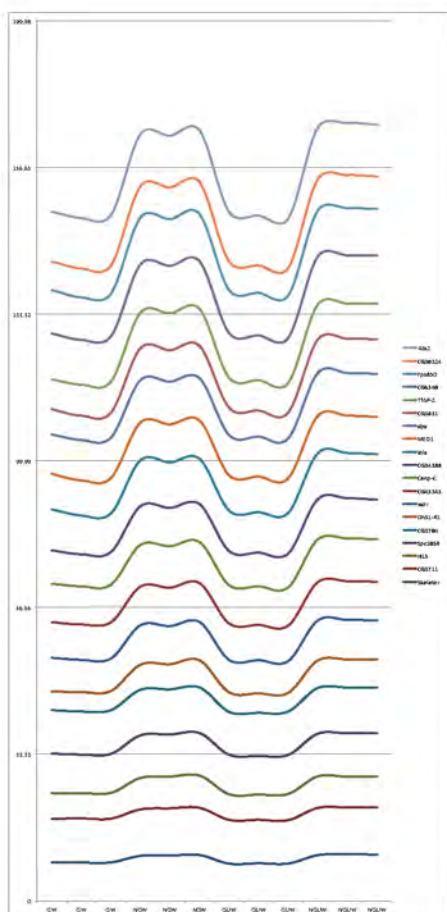

1212

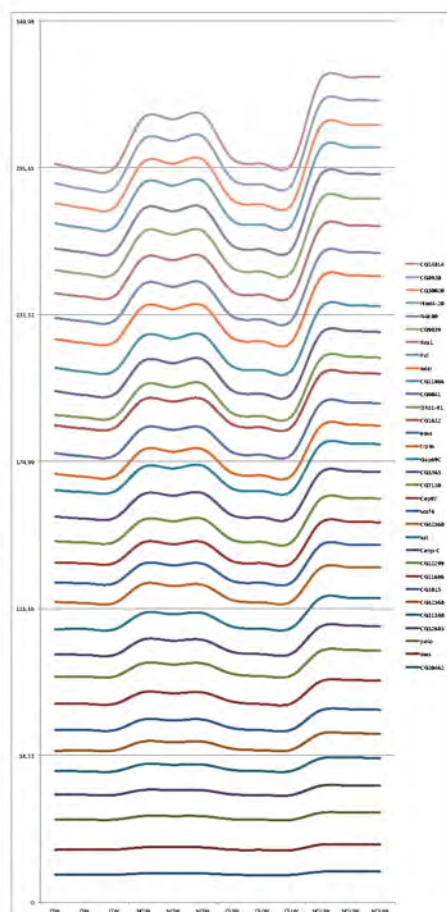

1213

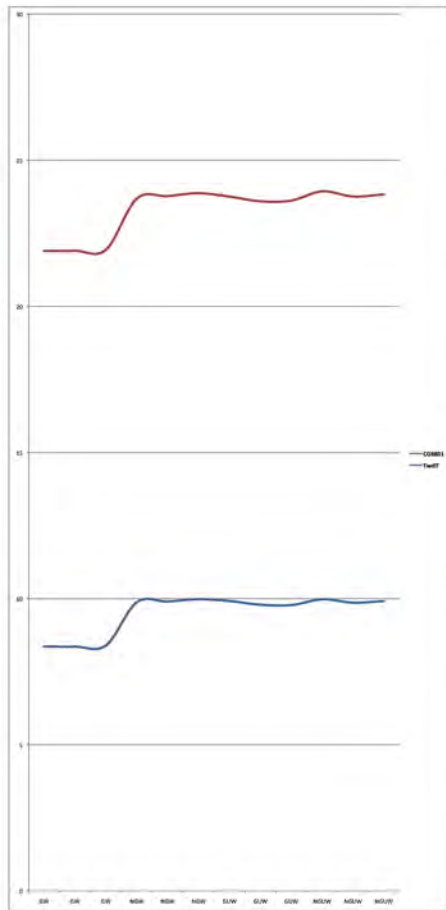

1222

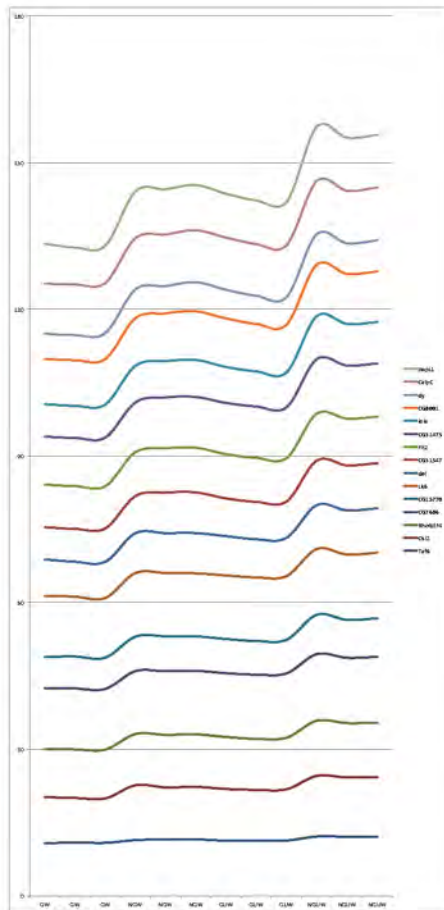

1223

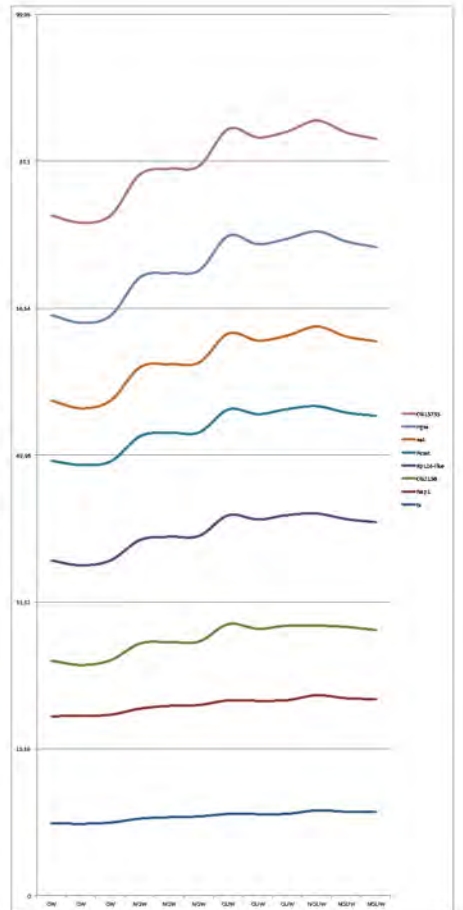

1233

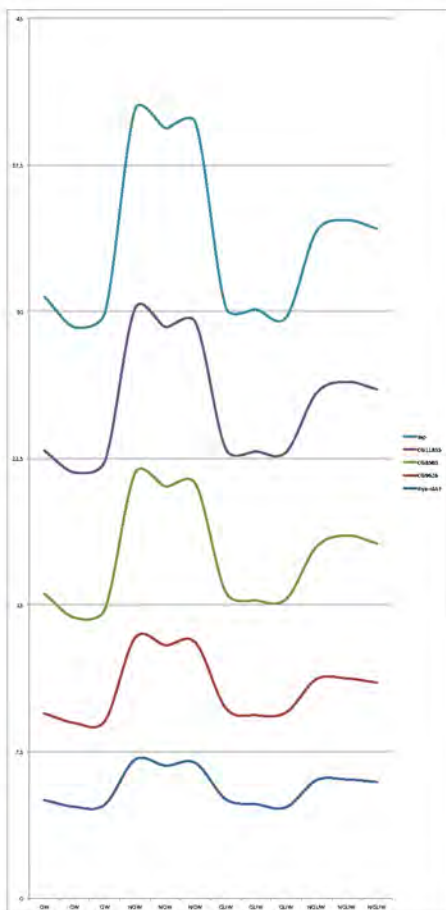

1312

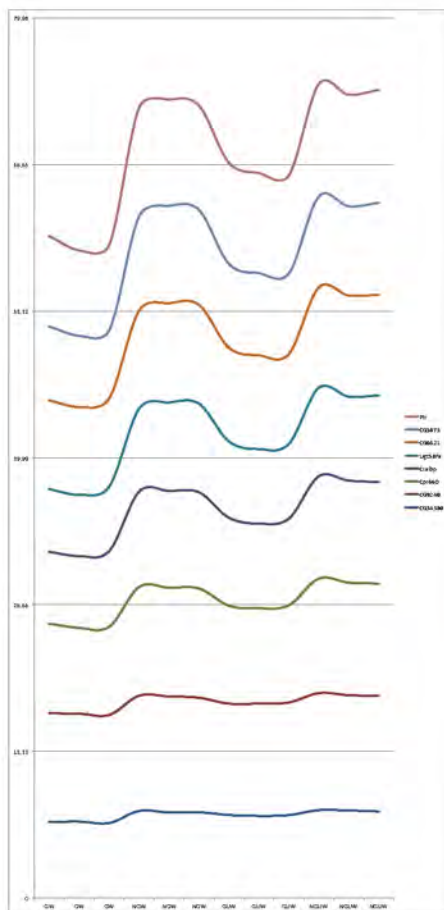

1323

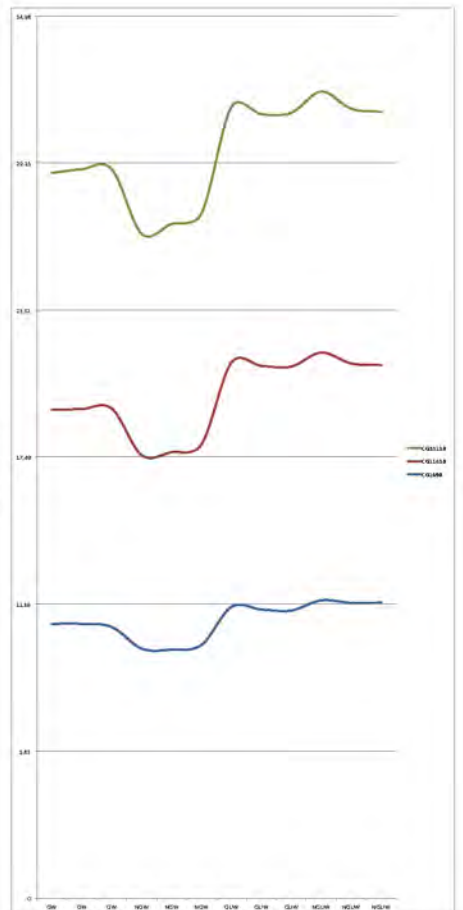

2133

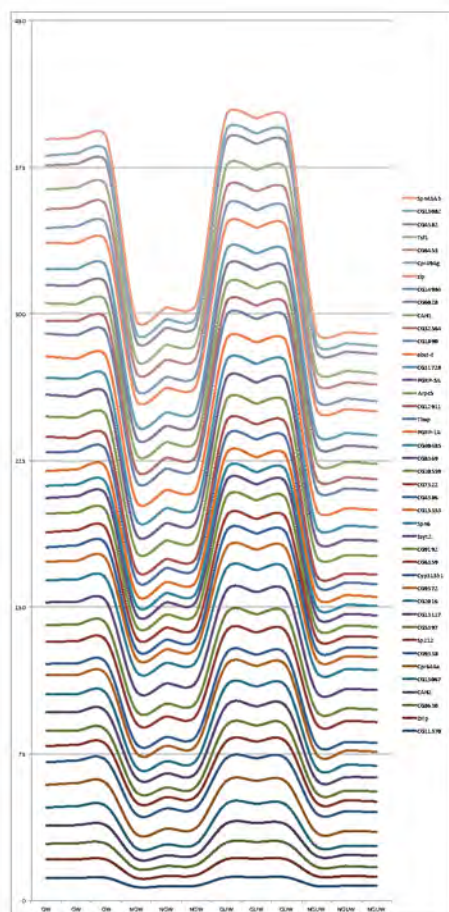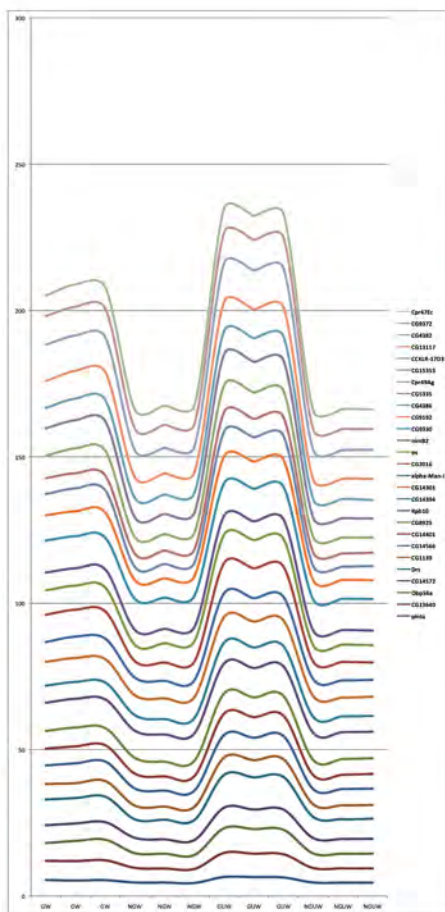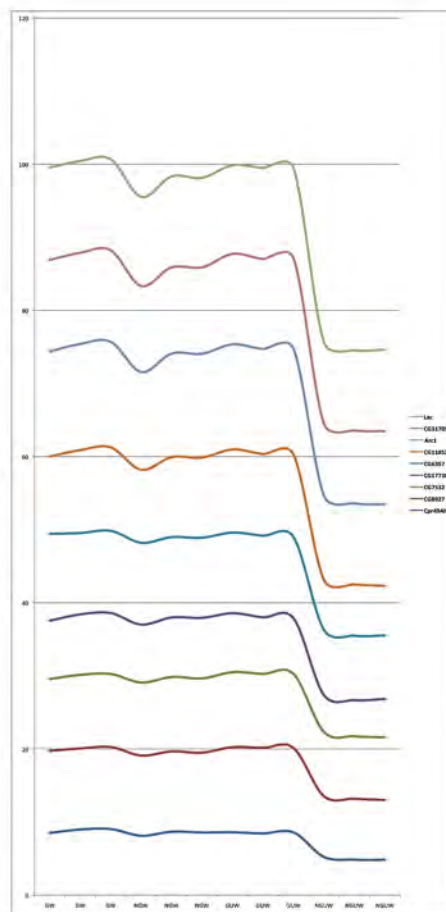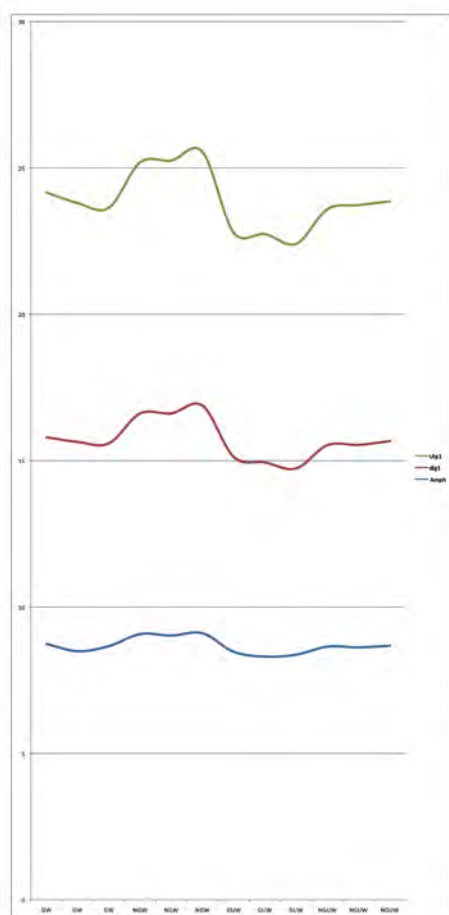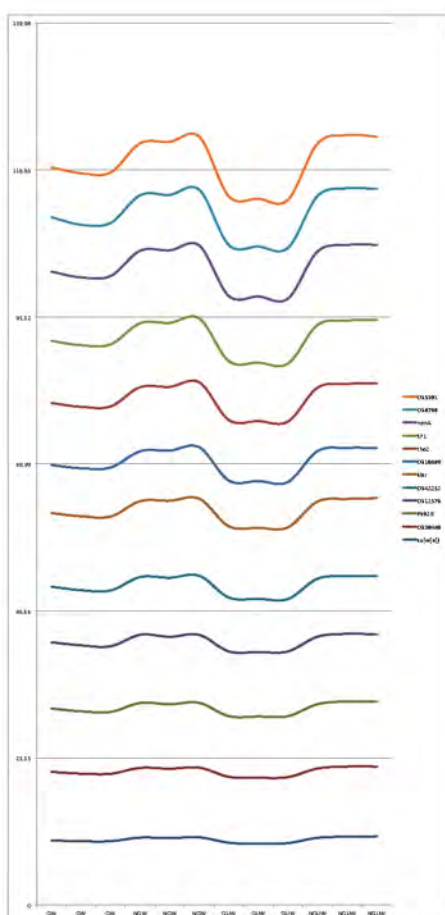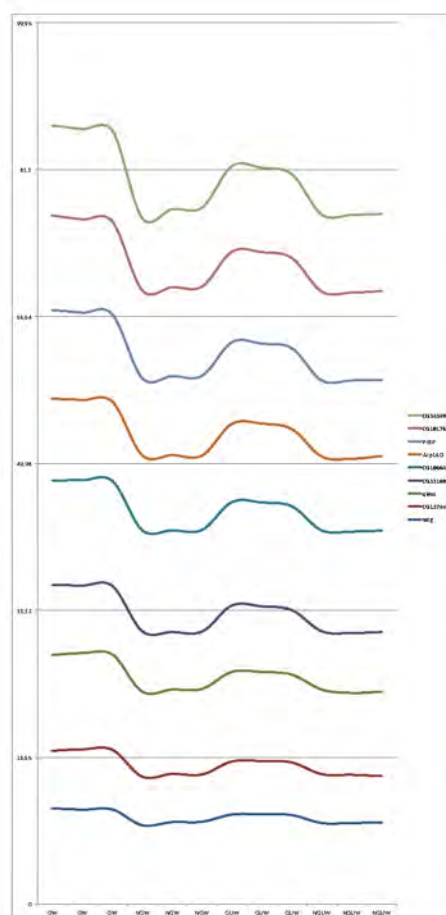

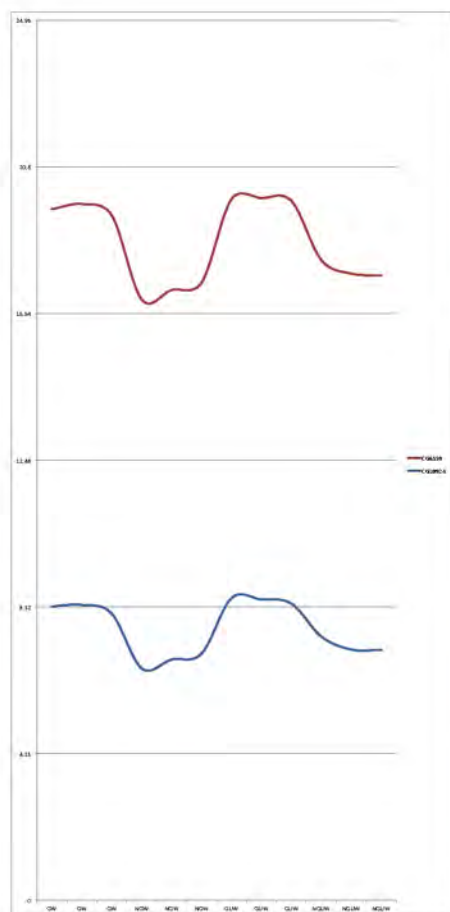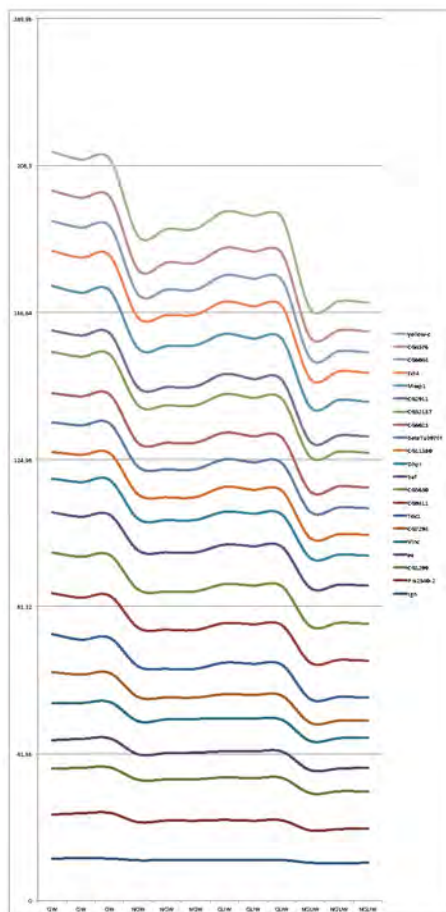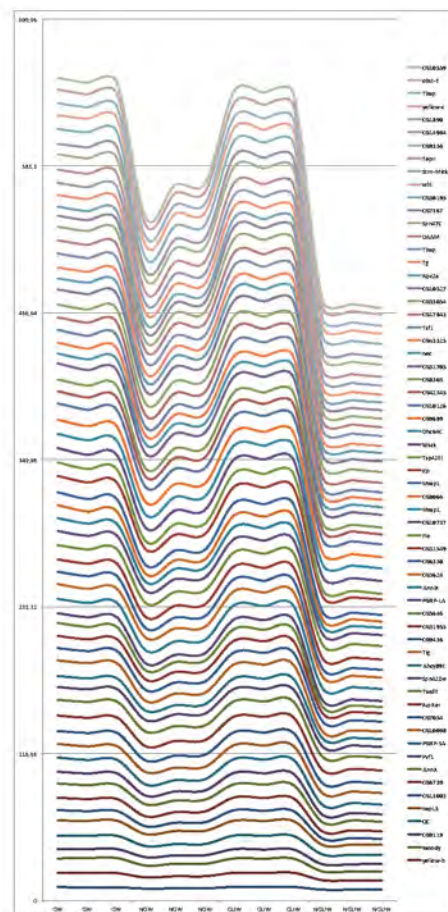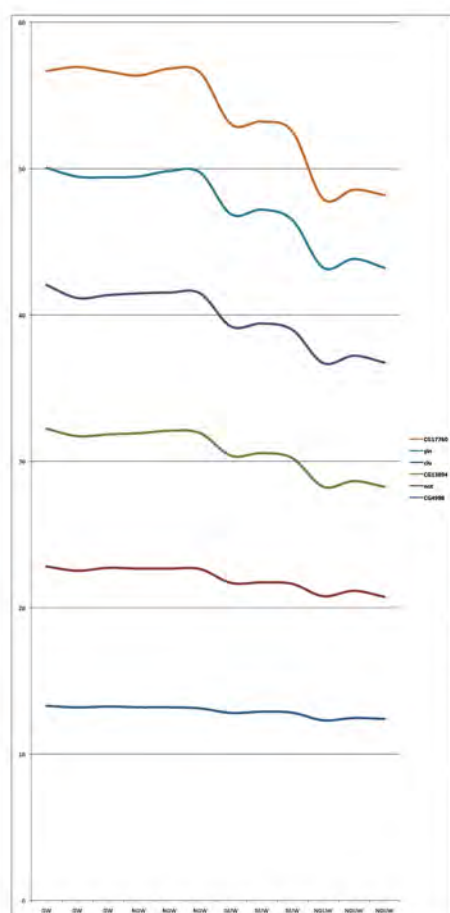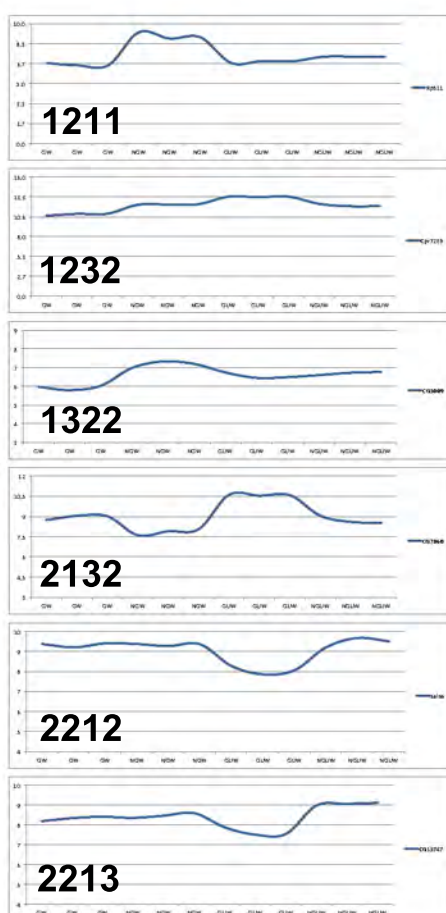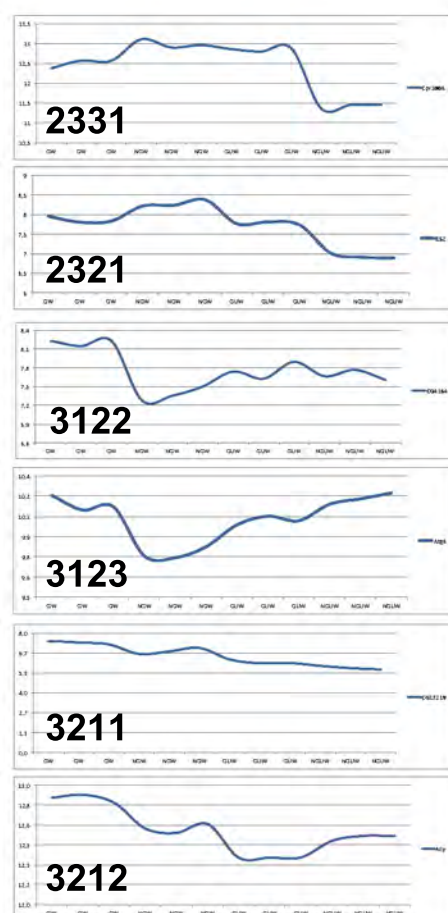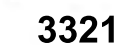

Supplement: S4 Fig — Representation of gene clusters by absolute expression scores. Each absolute value of expression for each gene in each replica (3) for the four conditions studied (JNK+ W, JNK- W, JNK+ and JNK-) was scored. Relative relationships in expression [classified in 3 levels (1/2/3) from lower to higher] between the different conditions were employed to cluster the different genes. For each cluster, all genes absolute expression values were described and represented as cumulative continuous lines in different colors across replicas and conditions (JNK+ W—1/2/3; JNK- W—1/2/3; JNK+—1/2/3; and JNK-—1/2/3). 34 distinct clusters of different sizes comprising 313 probes are represented. (PDF) [file pgen.1004965.s005.pdf]

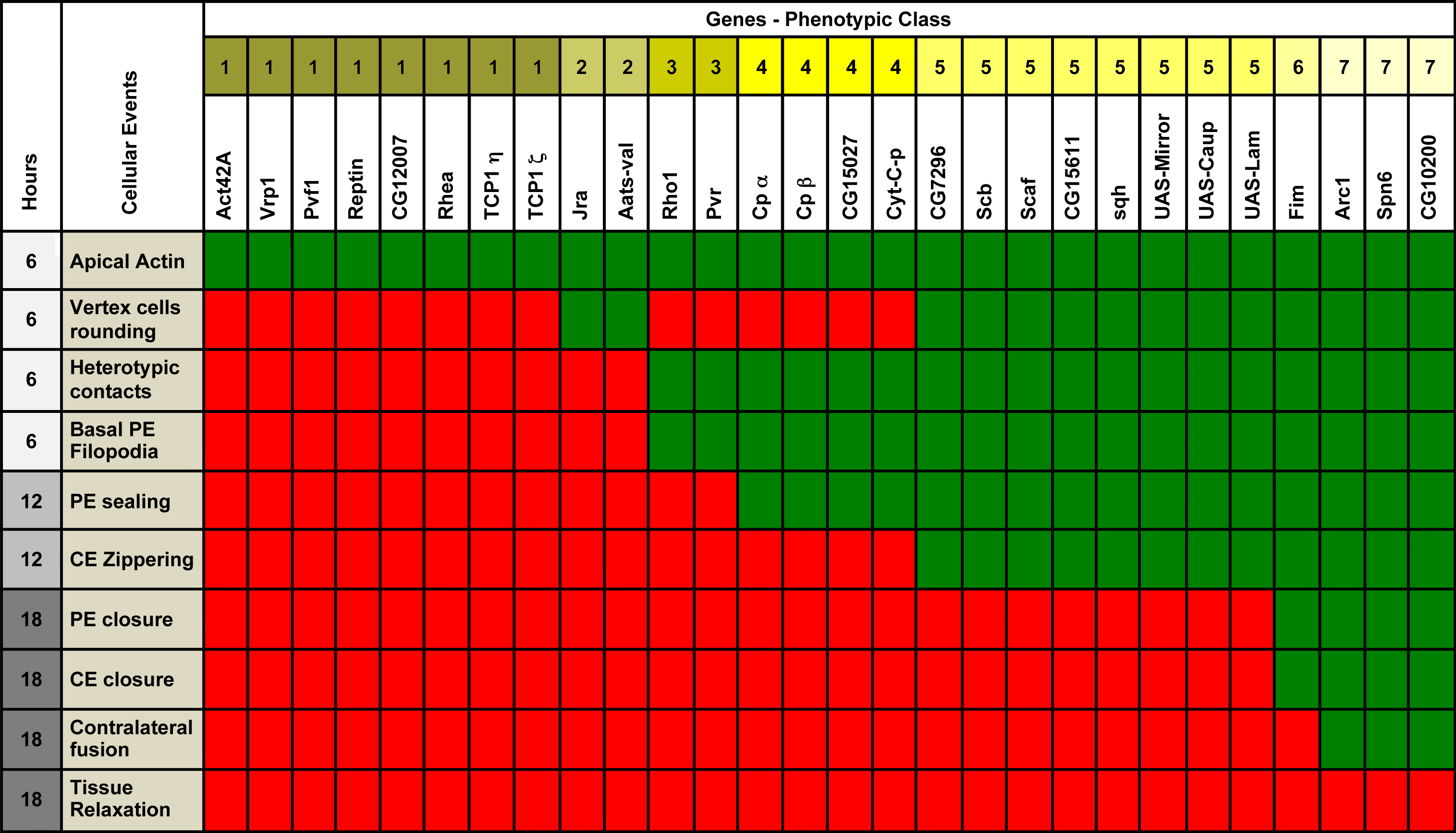

Supplement: S5 Fig — Healing was assayed at 25°C with different Gal4 lines (En or Pnr). Healing phenotypic classes are coded as in the text (1 -Early (6 hours) defects—Apical Actin; 2—Early (6 hours) defects—Unstructured Actin and vertex cells rounding; 3—Early (6 hours) defects—Actin and basal filopodia present but not vertex cells rounding; 4—Intermediate (12 hours) defects—Vertex cells rounding and CE zippering fails; 5—Intermediate (12 hours) defects—Gaps between the epithelia and no PE closure; 6—Late (18 hours) defects—Incomplete closure and 7—No tissue Relaxation—Tissue folds) (see Functional analysis of “healing” genes section). In green are shown the cellular events achieved in each interference assay. In red are shown those that failed. (TIF) [file pgen.1004965.s006.tif]

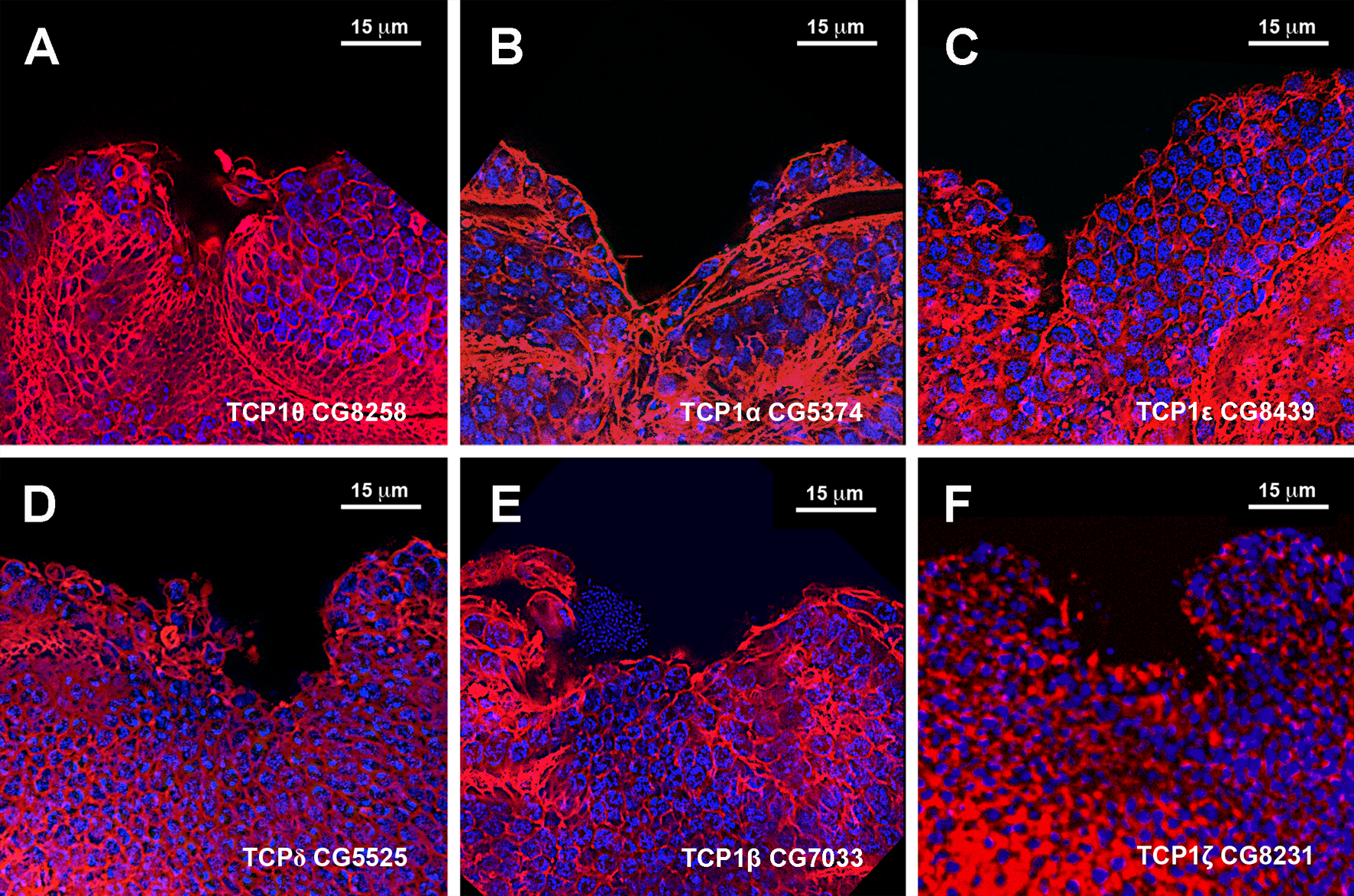

Supplement: S6 Fig — A to F) TCP1 subunits RNAi knockdowns result in impaired healing after 20–24 hours of culture of wounded imaginal wing discs in vitro. An early phenotype and an absence of filopodia formation and aberrant actin-rich structures were observed after interference (RNAi) with the expression of different TCP1 subunits as labeled (tested with an En-Gal4 driver). Phalloidin (actin) is shown in red; DAPI (nuclei) in blue. Scale bars are indicated for each panel. (TIF) [file pgen.1004965.s007.tif]

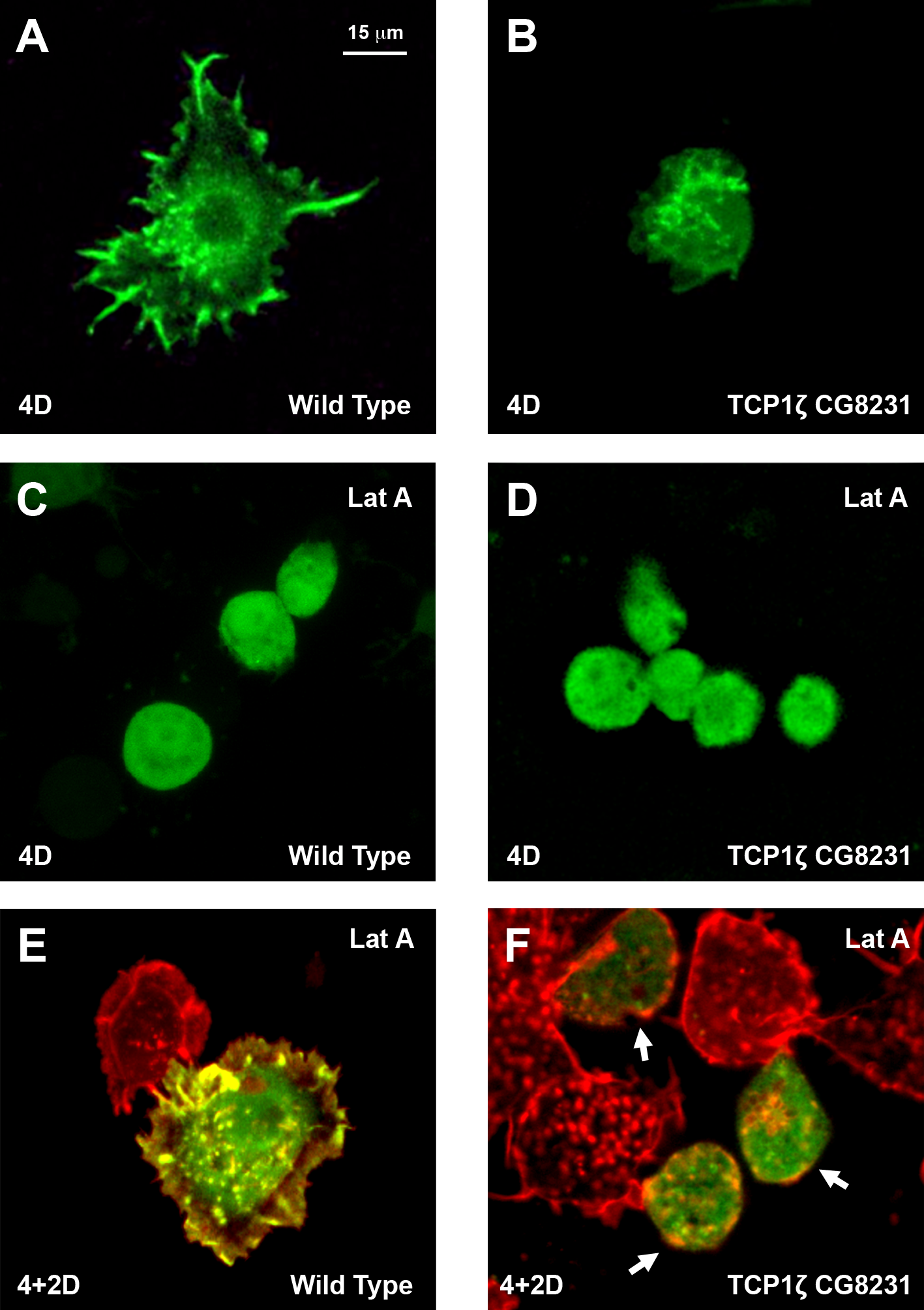

Supplement: S7 Fig — A and B) Drosophila S2R+ cells were transfected with pMT-Act-GFP +/- TCP1ζ dsRNA. Four days after transfection control cells (A) display a flat morphology and conspicuous filopodia and lamellipodia, while TCP1ζ depleted cells (B) show a defective actin cytoskeleton and lack filopodia. C and D) S2R+ cells were transfected with pMT-Act-GFP +/- TCP1ζ dsRNA and treated with four days after transfection LatA. In both cases, the actin cytoskeleton completely collapses in 1 hour and the cells become fully rounded. E and F) pMT-Act-GFP +/- TCP1ζ dsRNA transfected cells treated with LatA four days after transfection for 1 hour were let to recover for two days. The control wild type cells (E) fully restore the regular arrangement of their cytoskeleton and show a flat morphology. TCP1ζ depleted cells (green in F), on the contrary, failed to reassemble their actin cytoskeleton and display low level of filamentous actin (arrows), while their untransfected siblings fully recover. pMT-Act-GFP expression is shown in green and Phalloidin (F-actin) is shown in red. Scale bars are indicated. (TIF) [file pgen.1004965.s008.tif]
