# Supplementary material for: Identification and Functional Analysis of Healing Regulators in Drosophila
Source: PLoS Genet. 2015 Feb 3;11(2):e1004965. doi: 10.1371/journal.pgen.1004965 (PMC4315591; doi:10.1371/journal.pgen.1004965)
Supplement: S6 Table — 34 distinct gene clusters by level of expression for the four conditions studied (JNK+ W, JNK- W, JNK+ and JNK-) were scored (from 81 potential combinatorial possibilities). The numbers of genes in each cluster varied from 1 to 63. They are represented color coded in a concentric Pie Chart. For each cluster, all genes absolute expression values are displayed by their level of expression for each condition (JNK+ W—1/2/3; JNK- W—1/2/3; JNK+—1/2/3; and JNK-—1/2/3). (PDF) [file pgen.1004965.s014.pdf]

Absolute Expression Clusters

Probes Numbers

CLUSTER	NUMBER
1112	4
1121	5
1123	4
1132	4
1211	1
1212	19
1213	32
1222	2
1223	15
1232	1
1233	8
1312	5
1322	1
1323	8
2121	41
2131	26
2132	1
2133	3
2212	1
2213	1
2221	9
2312	3
2313	12
2321	1
2331	1
3121	9
3122	1
3123	1
3132	2
3211	1
3212	1
3221	21
3231	63
3321	6

Centripetal Pie Chart

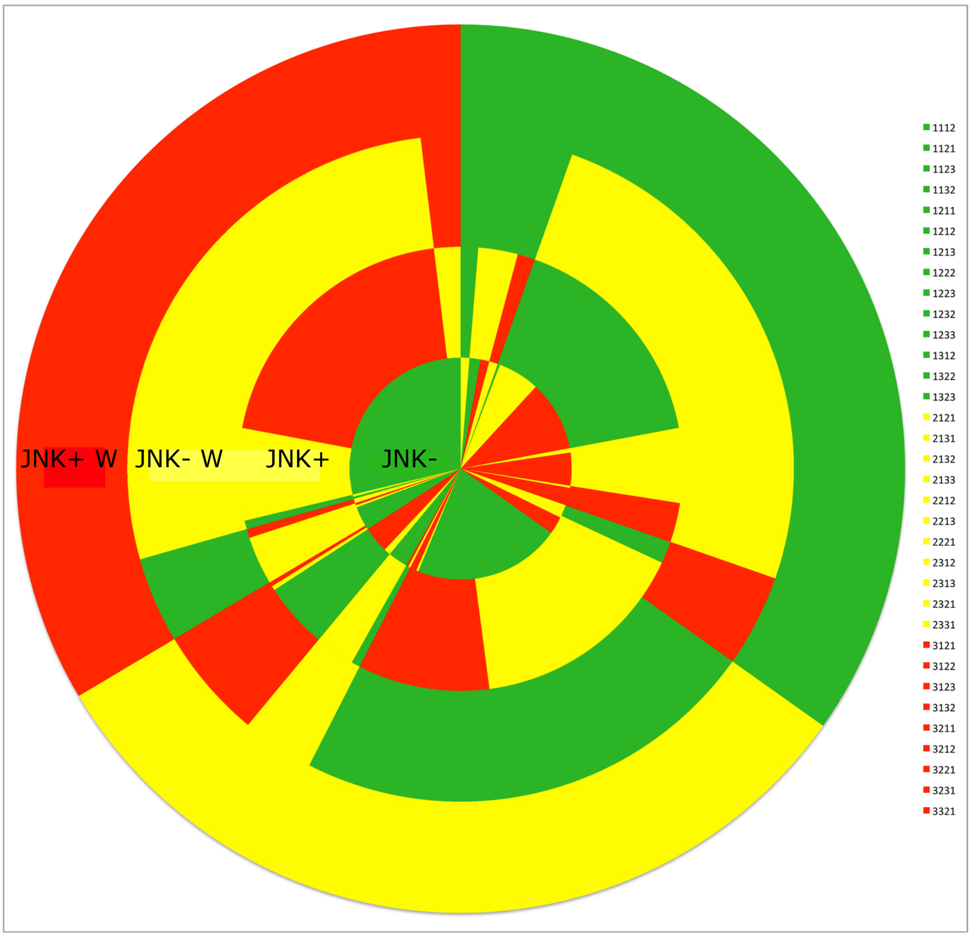

(High Expression) - 3
(Medium Expression) - 2
(Low Expression) - 1

CLUSTER	JNK+ W	JNK- W	JNK+	JNK-	ID
1112	1	1	1	2	CG5823
1112	1	1	1	2	CG8735
1112	1	1	1	2	qkr58E-2
1112	1	1	1	2	uri
1121	1	1	2	1	CG10918
1121	1	1	2	1	CG11413
1121	1	1	2	1	CG13460
1121	1	1	2	1	CG17325
1121	1	1	2	1	Sgs5
1123	1	1	2	3	CG2812
1123	1	1	2	3	CG3894
1123	1	1	2	3	CG8202
1123	1	1	2	3	Vha13
1132	1	1	3	2	CG30197
1132	1	1	3	2	CG7630
1132	1	1	3	2	Cht5
1132	1	1	3	2	His3.3A
1211	1	2	1	1	RpS11
1212	1	2	1	2	Atx2
1212	1	2	1	2	Cenp-C
1212	1	2	1	2	CG30324
1212	1	2	1	2	CG34388
1212	1	2	1	2	CG3704
1212	1	2	1	2	CG3711
1212	1	2	1	2	CG3831
1212	1	2	1	2	CG43343
1212	1	2	1	2	CG6340
1212	1	2	1	2	Dh31-R1
1212	1	2	1	2	dpa
1212	1	2	1	2	Fps85D
1212	1	2	1	2	H15
1212	1	2	1	2	lola
1212	1	2	1	2	MED1
1212	1	2	1	2	mirr
1212	1	2	1	2	Skeletor
1212	1	2	1	2	Spc105R
1212	1	2	1	2	TfAP-2
1213	1	2	1	3	Cenp-C
1213	1	2	1	3	Cep97
1213	1	2	1	3	CG10462
1213	1	2	1	3	CG11686
1213	1	2	1	3	CG11866
1213	1	2	1	3	CG12299
1213	1	2	1	3	CG14814
1213	1	2	1	3	CG1622
1213	1	2	1	3	CG30020
1213	1	2	1	3	CG31388
1213	1	2	1	3	CG32685
1213	1	2	1	3	CG3363
1213	1	2	1	3	CG3815
1213	1	2	1	3	CG42568
1213	1	2	1	3	CG42568
1213	1	2	1	3	CG6041
1213	1	2	1	3	CG7110
1213	1	2	1	3	CG8920
1213	1	2	1	3	CG9839
1213	1	2	1	3	Dh31-R1
1213	1	2	1	3	ema
1213	1	2	1	3	Gap69C
1213	1	2	1	3	Hmt4-20
1213	1	2	1	3	kst
1213	1	2	1	3	mas
1213	1	2	1	3	micr
1213	1	2	1	3	Ndc80
1213	1	2	1	3	Pcl
1213	1	2	1	3	polo
1213	1	2	1	3	Rca1
1213	1	2	1	3	scaf6
1213	1	2	1	3	trpm
1222	1	2	2	2	CG8801
1222	1	2	2	2	TwdlT
1223	1	2	2	3	CalpC
1223	1	2	2	3	CG15770
1223	1	2	2	3	CG31475
1223	1	2	2	3	CG31547
1223	1	2	2	3	CG7686
1223	1	2	2	3	CG8001
1223	1	2	2	3	del
1223	1	2	2	3	dy
1223	1	2	2	3	Fit2
1223	1	2	2	3	ImpL1
1223	1	2	2	3	Lk6
1223	1	2	2	3	lola
1223	1	2	2	3	Osi2
1223	1	2	2	3	RhoGEF4
1223	1	2	2	3	Taf6
1232	1	2	3	3	Cpr72Eb
1233	1	2	3	3	CG15735
1233	1	2	3	3	CG2150
1233	1	2	3	3	m4
1233	1	2	3	3	Nap1
1233	1	2	3	3	Pcmt
1233	1	2	3	3	Pgm
1233	1	2	3	3	RpL24-like
1233	1	2	3	3	tx
1312	1	3	1	2	CG11835
1312	1	3	1	2	CG8503
1312	1	3	1	2	CG9626
1312	1	3	1	2	mp
1312	1	3	1	2	Rya-r44F
1322	1	3	2	2	CG3009
1323	1	3	2	3	CG34380
1323	1	3	2	3	CG5873
1323	1	3	2	3	CG6621
1323	1	3	2	3	CG9240
1323	1	3	2	3	Cpr66D
1323	1	3	2	3	Cralbp
1323	1	3	2	3	Ptr

1323	1	3	2	3	Ugt58Fa
2121	2	1	2	1	Arpc5
2121	2	1	2	1	CAH1
2121	2	1	2	1	CAH2
2121	2	1	2	1	CG10559
2121	2	1	2	1	CG11370
2121	2	1	2	1	CG12911
2121	2	1	2	1	CG13067
2121	2	1	2	1	CG13082
2121	2	1	2	1	CG13117
2121	2	1	2	1	CG14984
2121	2	1	2	1	CG15353
2121	2	1	2	1	CG1890
2121	2	1	2	1	CG2016
2121	2	1	2	1	CG31728
2121	2	1	2	1	CG32564
2121	2	1	2	1	CG40485
2121	2	1	2	1	CG4382
2121	2	1	2	1	CG4386
2121	2	1	2	1	CG5397
2121	2	1	2	1	CG6028
2121	2	1	2	1	CG6359
2121	2	1	2	1	CG6453
2121	2	1	2	1	CG7322
2121	2	1	2	1	CG8369
2121	2	1	2	1	CG8630
2121	2	1	2	1	CG9192
2121	2	1	2	1	CG9338
2121	2	1	2	1	CG9372
2121	2	1	2	1	Cpr49Ag
2121	2	1	2	1	Cpr64Aa
2121	2	1	2	1	Cyp313b1
2121	2	1	2	1	Drip
2121	2	1	2	1	Esyt2
2121	2	1	2	1	obst-E
2121	2	1	2	1	PGRP-LA
2121	2	1	2	1	PGRP-SA
2121	2	1	2	1	Sp212
2121	2	1	2	1	Spn43Ab
2121	2	1	2	1	Spn6
2121	2	1	2	1	Timp
2121	2	1	2	1	Tsf1
2121	2	1	2	1	zip
2131	2	1	3	1	alpha-Man-I
2131	2	1	3	1	CCKLR-17D3
2131	2	1	3	1	CG1139
2131	2	1	3	1	CG13117
2131	2	1	3	1	CG13640
2131	2	1	3	1	CG14301
2131	2	1	3	1	CG14394
2131	2	1	3	1	CG14401
2131	2	1	3	1	CG14566
2131	2	1	3	1	CG14572
2131	2	1	3	1	CG15353
2131	2	1	3	1	CG2016
2131	2	1	3	1	CG4382
2131	2	1	3	1	CG4386
2131	2	1	3	1	CG5335
2131	2	1	3	1	CG8925
2131	2	1	3	1	CG9192
2131	2	1	3	1	CG9330
2131	2	1	3	1	CG9372
2131	2	1	3	1	Cpr47Ec
2131	2	1	3	1	Cpr49Ag
2131	2	1	3	1	Drs
2131	2	1	3	1	Irc
2131	2	1	3	1	nimB2
2131	2	1	3	1	Obp56a
2131	2	1	3	1	pinta
2131	2	1	3	1	Rpb10
2132	2	1	3	2	CG7860
2133	2	1	3	3	CG11438
2133	2	1	3	3	CG1698
2133	2	1	3	3	CG33138
2212	2	2	1	2	salm
2213	2	2	1	3	CG15747
2221	2	2	2	1	Arc1
2221	2	2	2	1	CG11852
2221	2	2	2	1	CG17738
2221	2	2	2	1	CG31705
2221	2	2	2	1	CG6357
2221	2	2	2	1	CG7532
2221	2	2	2	1	CG8927
2221	2	2	2	1	Cpr49Ah
2221	2	2	2	1	Lac
2312	2	3	1	2	Amph
2312	2	3	1	2	dlg1
2312	2	3	1	2	Ulp1
2313	2	3	1	3	CG10889
2313	2	3	1	3	CG12576
2313	2	3	1	3	CG30440
2313	2	3	1	3	CG42232
2313	2	3	1	3	CG5591
2313	2	3	1	3	CG8798
2313	2	3	1	3	klar
2313	2	3	1	3	nonA
2313	2	3	1	3	Pk92B
2313	2	3	1	3	SF1
2313	2	3	1	3	su(w[a])
2313	2	3	1	3	tho2
2321	2	3	2	1	B52
2331	2	3	3	1	Cpr100A
3121	3	1	2	1	Arp14D
3121	3	1	2	1	CG10176
3121	3	1	2	1	CG10664
3121	3	1	2	1	CG13744
3121	3	1	2	1	CG33169
3121	3	1	2	1	CG34349
3121	3	1	2	1	mtg
3121	3	1	2	1	PrBP
3121	3	1	2	1	qless

3122	3	1	2	2	CG4164
3123	3	1	2	3	Atg4
3132	3	1	3	2	CG10924
3132	3	1	3	2	CG6359
3211	3	2	1	1	CG17219
3212	3	2	1	2	Nlp
3221	3	2	2	1	baf
3221	3	2	2	1	betaTub97EF
3221	3	2	2	1	CG11380
3221	3	2	2	1	CG1299
3221	3	2	2	1	CG2911
3221	3	2	2	1	CG32137
3221	3	2	2	1	CG4576
3221	3	2	2	1	CG5630
3221	3	2	2	1	CG6023
3221	3	2	2	1	CG6044
3221	3	2	2	1	CG7294
3221	3	2	2	1	CG9411
3221	3	2	2	1	Dhpr
3221	3	2	2	1	ea
3221	3	2	2	1	Ect4
3221	3	2	2	1	Mmp1
3221	3	2	2	1	Prx2540-2
3221	3	2	2	1	rgn
3221	3	2	2	1	Tdc1
3221	3	2	2	1	Vinc
3221	3	2	2	1	yellow-c
3231	3	2	3	1	Ahcy89E
3231	3	2	3	1	AnnIX
3231	3	2	3	1	AnnX
3231	3	2	3	1	CG10098
3231	3	2	3	1	CG10126
3231	3	2	3	1	CG10527
3231	3	2	3	1	CG10559
3231	3	2	3	1	CG10737
3231	3	2	3	1	CG13003
3231	3	2	3	1	CG14984
3231	3	2	3	1	CG17843
3231	3	2	3	1	CG1890
3231	3	2	3	1	CG30195
3231	3	2	3	1	CG31549
3231	3	2	3	1	CG31705
3231	3	2	3	1	CG31955
3231	3	2	3	1	CG33054
3231	3	2	3	1	CG3624
3231	3	2	3	1	CG42345
3231	3	2	3	1	CG43325
3231	3	2	3	1	CG5646
3231	3	2	3	1	CG6330
3231	3	2	3	1	CG6739
3231	3	2	3	1	CG7054
3231	3	2	3	1	CG7367
3231	3	2	3	1	CG8066
3231	3	2	3	1	CG8303
3231	3	2	3	1	CG9119
3231	3	2	3	1	CG9336
3231	3	2	3	1	CG9436
3231	3	2	3	1	CG9689
3231	3	2	3	1	Cht3
3231	3	2	3	1	DAAM
3231	3	2	3	1	Dhc64C
3231	3	2	3	1	Flo
3231	3	2	3	1	ImpL3
3231	3	2	3	1	Jupiter
3231	3	2	3	1	Mmp1
3231	3	2	3	1	Mmp1
3231	3	2	3	1	moody
3231	3	2	3	1	nec
3231	3	2	3	1	Npc2a
3231	3	2	3	1	obst-E
3231	3	2	3	1	PGRP-LA
3231	3	2	3	1	PGRP-SA
3231	3	2	3	1	Pvf1
3231	3	2	3	1	QC
3231	3	2	3	1	Spn42De
3231	3	2	3	1	Spn47C
3231	3	2	3	1	Strn-Mlck
3231	3	2	3	1	TepII
3231	3	2	3	1	Tg
3231	3	2	3	1	Tig
3231	3	2	3	1	Timp
3231	3	2	3	1	Timp
3231	3	2	3	1	Tsf1
3231	3	2	3	1	Tsp42El
3231	3	2	3	1	TwdlE
3231	3	2	3	1	wbl
3231	3	2	3	1	Wsck
3231	3	2	3	1	yellow-b
3231	3	2	3	1	yellow-c
3231	3	2	3	1	zip
3321	3	3	2	1	CG13894
3321	3	3	2	1	CG17760
3321	3	3	2	1	CG4998
3321	3	3	2	1	clu
3321	3	3	2	1	not
3321	3	3	2	1	yin
