# Supplementary material for: Identification and Functional Analysis of Healing Regulators in Drosophila
Source: PLoS Genet. 2015 Feb 3;11(2):e1004965. doi: 10.1371/journal.pgen.1004965 (PMC4315591; doi:10.1371/journal.pgen.1004965)
Supplement: S7 Table — Chromosomal clusters of upregulated genes for the global comparison (33) are described by their chromosomal location, number of genes, number of co regulated genes, identity of each gene and each gene’s GO Terms. Genes highlighted in orange are those transcriptionally co regulated during healing. (PDF) [file pgen.1004965.s015.pdf]

globalUp – chr2L: 1129234 - 1158264

Genomic components: 3 coregulated genes, 8 genes

CHR	Strand	Start	End	RefSeq	Name	Exons	Description
CHR2L	+	1129234	1132413	NM_134725	CG4552	3	CG4552-PA
CHR2L	-	1132329	1134120	NM_134726	Iris	1	Iris CG4715-PA
CHR2L	+	1134701	1141156	NM_134727	CG4577	5	CG4577-PA
CHR2L	+	1143202	1147395	NM_134728	CG4726	4	CG4726-PA
CHR2L	+	1147832	1149449	NM_134729	CG4749	1	CG4749-PA
CHR2L	-	1149444	1150673	NM_134730	Tfb4	5	Tfb4 CG5041-PA
CHR2L	+	1150782	1151764	NM_134731	CG4764	1	CG4764-PA
CHR2L	-	1151677	1158264	NM_001038780	capt	5	capulet CG33979-PA, isoform A

Cluster size: 29031 nucleotides

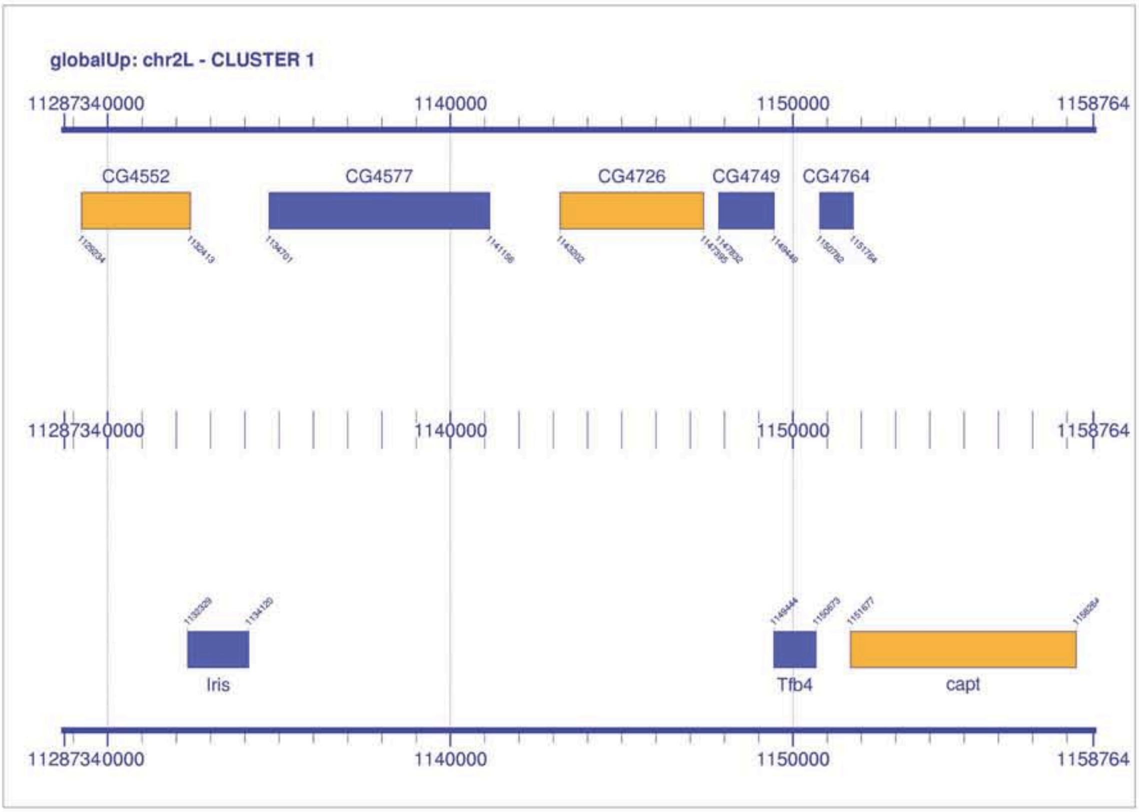

globalUp – cluster 1

Genomic components:

Name	RefSeq	Function	
CG4552	NM_134725		
Iris	NM_134726	GO:0009993	oogenesis (sensu Insecta)
CG4577	NM_134727		
CG4726	NM_134728	GO:0005316	high affinity inorganic phosphate:sodium symporter activity
		GO:0005975	carbohydrate metabolic process
		GO:0006796	phosphate metabolic process
		GO:0006812	cation transport
		GO:0006817	phosphate transport
		GO:0006858	extracellular transport
		GO:0008643	carbohydrate transport
		GO:0016021	integral to membrane
CG4749	NM_134729	GO:0003676	nucleic acid binding
		GO:0006139	nucleobase, nucleoside, nucleotide and nucleic acid metabolic process
		GO:0016072	rRNA metabolic process
Tfb4	NM_134730	GO:0003700	transcription factor activity
		GO:0005675	transcription factor TFIIH complex
		GO:0006281	DNA repair
		GO:0006355	regulation of transcription, DNA-dependent
		GO:0006367	transcription initiation from RNA polymerase II promoter
		GO:0016251	general RNA polymerase II transcription factor activity
CG4764	NM_134731	GO:0006886	intracellular protein transport
		GO:0016787	hydrolase activity
capt	NM_001038780	GO:0000902	cell morphogenesis
		GO:0003779	actin binding
		GO:0005200	structural constituent of cytoskeleton
		GO:0007015	actin filament organization
		GO:0008103	oocyte microtubule cytoskeleton polarization
		GO:0008154	actin polymerization and/or depolymerization
		GO:0008179	adenylate cyclase binding
		GO:0008360	regulation of cell shape
		GO:0008407	bristle morphogenesis
		GO:0009993	oogenesis (sensu Insecta)
		GO:0015630	microtubule cytoskeleton
		GO:0016020	membrane
		GO:0048190	wing disc dorsal/ventral pattern formation

GO density (8 genes):

Ranking	GO id	Function	Frequency
1	GO:0009993	oogenesis (sensu Insecta)	25 %
2	GO:0006367	transcription initiation from RNA polymerase II promoter	12 %
3	GO:0006817	phosphate transport	12 %
4	GO:0008643	carbohydrate transport	12 %
5	GO:0005675	transcription factor TFIIH complex	12 %
6	GO:0006886	intracellular protein transport	12 %
7	GO:0003779	actin binding	12 %
8	GO:0003676	nucleic acid binding	12 %
9	GO:0005316	high affinity inorganic phosphate:sodium symporter activity	12 %
10	GO:0008179	adenylate cyclase binding	12 %
11	GO:0008360	regulation of cell shape	12 %
12	GO:0006139	nucleobase, nucleoside, nucleotide and nucleic acid metabolic process	12 %
13	GO:0006858	extracellular transport	12 %
14	GO:0006812	cation transport	12 %
15	GO:0005200	structural constituent of cytoskeleton	12 %
16	GO:0048190	wing disc dorsal/ventral pattern formation	12 %
17	GO:0015630	microtubule cytoskeleton	12 %
18	GO:0006281	DNA repair	12 %
19	GO:0000902	cell morphogenesis	12 %
20	GO:0006796	phosphate metabolic process	12 %
21	GO:0003700	transcription factor activity	12 %
22	GO:0008103	oocyte microtubule cytoskeleton polarization	12 %
23	GO:0005975	carbohydrate metabolic process	12 %
24	GO:0016021	integral to membrane	12 %
25	GO:0016787	hydrolase activity	12 %
26	GO:0007015	actin filament organization	12 %
27	GO:0008154	actin polymerization and/or depolymerization	12 %
28	GO:0006355	regulation of transcription, DNA-dependent	12 %
29	GO:0016251	general RNA polymerase II transcription factor activity	12 %
30	GO:0008407	bristle morphogenesis	12 %
31	GO:0016072	rRNA metabolic process	12 %
32	GO:0016020	membrane	12 %

globalUp – chr2L: 8148949 - 8189610

Genomic components: 3 coregulated genes, 6 genes

Chr	Strand	Start	End	RefSeq	Name	Exons	Description
chr2L	+	8148949	8155607	NM_164793	CG31607	5	CG31607-PA
chr2L	+	8156826	8158711	NM_164794	CG31900	5	CG31900-PA
chr2L	+	8159838	8161797	NM_135342	CG8506	3	CG8506-PA
chr2L	-	8161920	8162325	NM_135343	CG8498	3	CG8498-PA
chr2L	+	8163297	8189610	NM_001042881	CG8486	30	CG8486-PC, isoform C
chr2L	+	8180713	8181330	NM_205924	fos28F	2	fos-related gene at 28F CG18103-PA

Cluster size: 40662 nucleotides

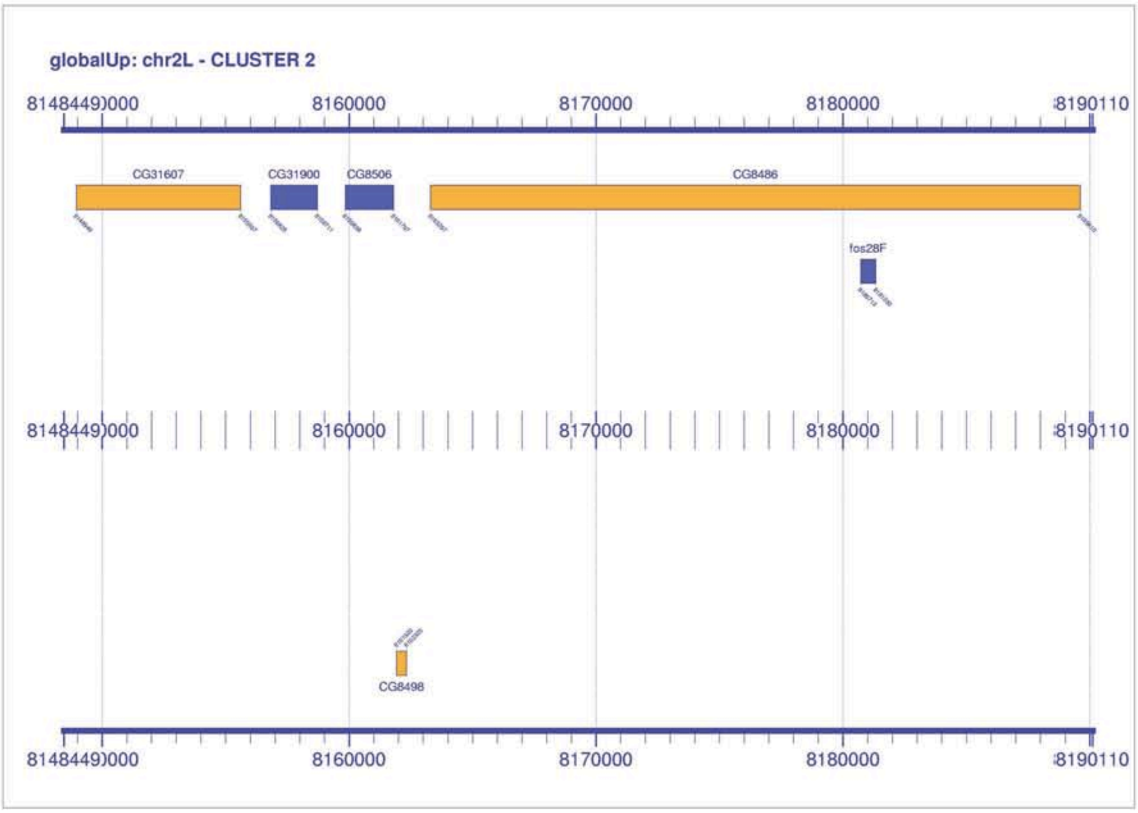

globalUp – cluster 2

Genomic components:

NAME	RefSeq	Function	
CG31607	NM_164793	GO:0003674	molecular_function
		GO:0005575	cellular_component
		GO:0008150	biological_process
CG31900	NM_164794	GO:0003674	molecular_function
		GO:0005575	cellular_component
		GO:0008150	biological_process
CG8506	NM_135342	GO:0003676	nucleic acid binding
		GO:0008270	zinc ion binding
CG8498	NM_135343	GO:0000062	acyl-CoA binding
		GO:0005386	carrier activity
		GO:0006869	lipid transport
		GO:0042049	cell acyl-CoA homeostasis
CG8486	NM_001042881		
FOS28F	NM_205924		

GO density (6 genes):

RANKING	GO id	Function	Frequency
1	GO:0008150	biological_process	33 %
2	GO:0005575	cellular_component	33 %
3	GO:0003674	molecular_function	33 %
4	GO:0005386	carrier activity	16 %
5	GO:0003676	nucleic acid binding	16 %
6	GO:0000062	acyl-CoA binding	16 %
7	GO:0042049	cell acyl-CoA homeostasis	16 %
8	GO:0008270	zinc ion binding	16 %
9	GO:0006869	lipid transport	16 %

globalUp – chr2L: 10314794 - 10333129

Genomic components: 3 coregulated genes, 6 genes

Chr	Strand	Start	End	RefSeq	Name	Exons	Description
chr2L	+	10314794	10317573	NM_135534	CG4972	6	CG4972-PA
chr2L	-	10317493	10321800	NM_135535	CG5381	7	CG5381-PA
chr2L	+	10322475	10324990	NM_135536	CG4995	5	CG4995-PA, isoform A
chr2L	-	10325017	10327986	NM_057679	RnrL	4	Ribonucleoside diphosphate reductase large
chr2L	-	10328383	10330564	NM_135537	CG5375	5	CG5375-PA
chr2L	+	10331462	10333129	NM_135538	CG5037	4	CG5037-PA

Cluster size: 18336 nucleotides

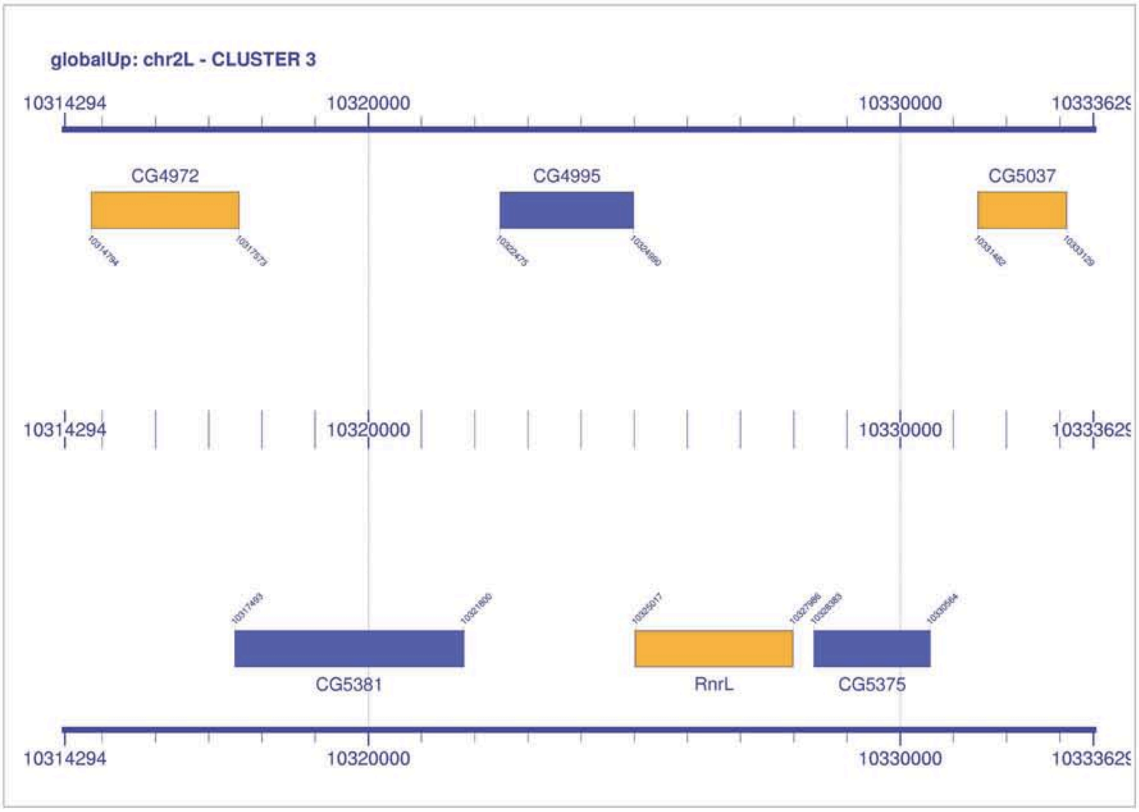

globalUp – cluster 3

Genomic components:

Name	RefSeq	Function	
CG4972	NM_135534		
CG5381	NM_135535		
CG4995	NM_135536	GO:0005386	carrier activity
		GO:0005488	binding
		GO:0005740	mitochondrial envelope
		GO:0005743	mitochondrial inner membrane
		GO:0006629	lipid metabolic process
		GO:0006869	lipid transport
RnrL	NM_057679	GO:0004748	ribonucleoside-diphosphate reductase activity
		GO:0005971	ribonucleoside-diphosphate reductase complex
		GO:0006144	purine base metabolic process
		GO:0006206	pyrimidine base metabolic process
		GO:0006260	DNA replication
CG5375	NM_135537		
CG5037	NM_135538	GO:0005740	mitochondrial envelope
		GO:0006732	coenzyme metabolic process
		GO:0008415	acyltransferase activity
		GO:0008495	protoheme IX farnesyltransferase activity
		GO:0016021	integral to membrane
		GO:0048034	heme o biosynthetic process

GO density (6 genes):

Ranking	GO id	Function	Frequency
1	GO:0005740	mitochondrial envelope	33 %
2	GO:0006629	lipid metabolic process	16 %
3	GO:0005386	carrier activity	16 %
4	GO:0005743	mitochondrial inner membrane	16 %
5	GO:0008415	acyltransferase activity	16 %
6	GO:0048034	heme o biosynthetic process	16 %
7	GO:0005488	binding	16 %
8	GO:0005971	ribonucleoside-diphosphate reductase complex	16 %
9	GO:0006206	pyrimidine base metabolic process	16 %
10	GO:0006144	purine base metabolic process	16 %
11	GO:0016021	integral to membrane	16 %
12	GO:0006732	coenzyme metabolic process	16 %
13	GO:0004748	ribonucleoside-diphosphate reductase activity	16 %
14	GO:0008495	protoheme IX farnesyltransferase activity	16 %
15	GO:0006869	lipid transport	16 %
16	GO:0006260	DNA replication	16 %

globalUp – chr2L: 10677369 - 10694103

Genomic components: 4 coregulated genes, 7 genes

CHR	Strand	Start	End	RefSeq	Name	Exons	Description
CHR2L	+	10677369	10679507	NM_135589	CG17104	3	CG17104-PA
CHR2L	+	10681046	10681356	NM_135590	CG17105	1	CG17105-PA
CHR2L	+	10681972	10682403	NM_135591	CG17107	1	CG17107-PA
CHR2L	-	10683760	10684407	NM_135592	CG7299	1	CG7299-PA
CHR2L	-	10686375	10686988	NM_135593	CG7296	1	CG7296-PA
CHR2L	-	10688731	10689269	NM_135594	CG7294	1	CG7294-PA
CHR2L	-	10692939	10694103	NM_135595	CG17108	1	CG17108-PA

Cluster size: 16735 nucleotides

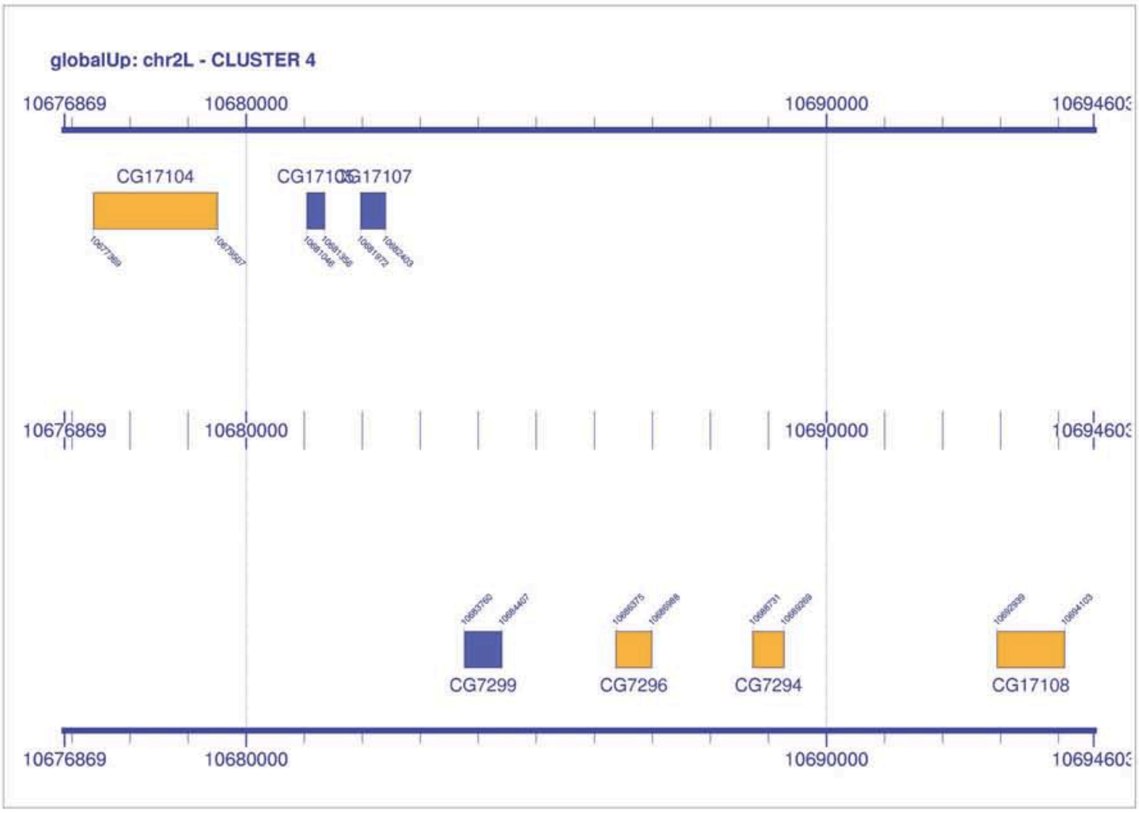

globalUp – cluster 4

Genomic components:

Name	RefSeq	Function	
CG17104	NM_135589		
CG17105	NM_135590		
CG17107	NM_135591		
CG7299	NM_135592		
CG7296	NM_135593		
CG7294	NM_135594		
CG17108	NM_135595	GO:0003989	acetyl-CoA carboxylase activity

GO density (7 genes):

Ranking	GO id	Function	Frequency
1	GO:0003989	acetyl-CoA carboxylase activity	14 %

globalUp – chr2L: 12036002 - 12055982

Genomic components: 3 coregulated genes, 7 genes

CHR	Strand	Start	End	RefSeq	Name	Exons	Description
CHR2L	-	12036002	12038543	NM_135692	Tom70	3	Translocase of outer membrane 70 CG6756-PA,
CHR2L	-	12038614	12040682	NM_135693	CG6766	4	CG6766-PA
CHR2L	-	12041048	12045200	NM_135694	CG6785	3	CG6785-PA
CHR2L	-	12045349	12046100	NM_135695	CG6770	1	CG6770-PA
CHR2L	-	12046856	12048918	NM_135696	CG6792	2	CG6792-PA
CHR2L	+	12049422	12052049	NM_164987	CG14945	3	CG14945-PB, isoform B
CHR2L	-	12051747	12055982	NM_135698	JhI-21	8	JhI-21 CG12317-PA, isoform A

Cluster size: 19981 nucleotides

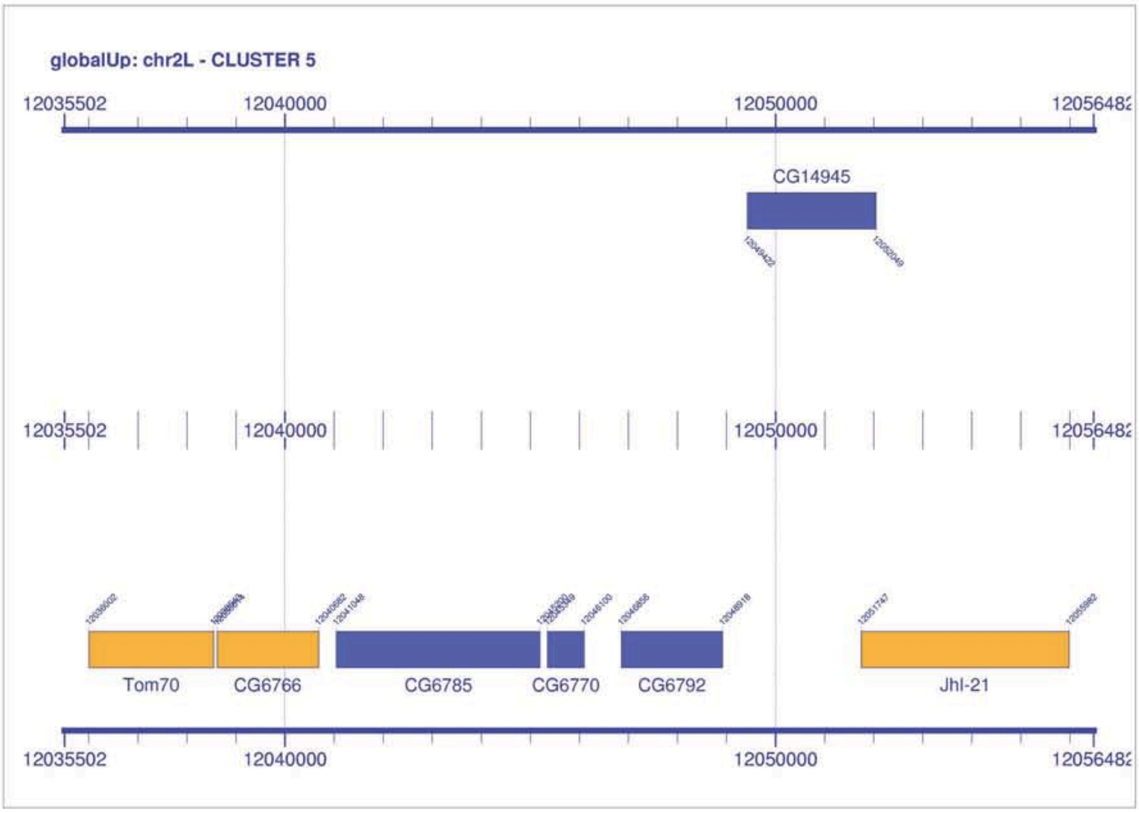

globalUp – cluster 5

Genomic components:

Name	RefSeq	Function	
Tom70	NM_135692	GO:0005386	carrier activity
		GO:0005488	binding
		GO:0005742	mitochondrial outer membrane translocase complex
		GO:0006626	protein targeting to mitochondrion
		GO:0015450	protein translocase activity
		GO:0016757	transferase activity, transferring glycosyl groups
CG6766	NM_135693		
CG6785	NM_135694		
CG6770	NM_135695		
CG6792	NM_135696	GO:0003676	nucleic acid binding
		GO:0005515	protein binding
		GO:0006139	nucleobase, nucleoside, nucleotide and nucleic acid metabolic process
		GO:0006357	regulation of transcription from RNA polymerase II promoter
		GO:0006366	transcription from RNA polymerase II promoter
		GO:0008270	zinc ion binding
		GO:0008283	cell proliferation
		GO:0030528	transcription regulator activity
CG14945	NM_164987		
JhI-21	NM_135698	GO:0005279	amino acid-polyamine transporter activity
		GO:0006520	amino acid metabolic process
		GO:0006865	amino acid transport
		GO:0015171	amino acid transporter activity
		GO:0015179	L-amino acid transporter activity
		GO:0016020	membrane

GO density (7 genes):

Ranking	GO id	Function	Frequency
1	GO:0015171	amino acid transporter activity	14 %
2	GO:0005386	carrier activity	14 %
3	GO:0015450	protein translocase activity	14 %
4	GO:0016757	transferase activity, transferring glycosyl groups	14 %
5	GO:0005742	mitochondrial outer membrane translocase complex	14 %
6	GO:0006626	protein targeting to mitochondrion	14 %
7	GO:0006865	amino acid transport	14 %
8	GO:0003676	nucleic acid binding	14 %
9	GO:0006366	transcription from RNA polymerase II promoter	14 %
10	GO:0005488	binding	14 %
11	GO:0006139	nucleobase, nucleoside, nucleotide and nucleic acid metabolic process	14 %
12	GO:0006520	amino acid metabolic process	14 %
13	GO:0008270	zinc ion binding	14 %
14	GO:0005279	amino acid-polyamine transporter activity	14 %
15	GO:0005515	protein binding	14 %
16	GO:0008283	cell proliferation	14 %
17	GO:0006357	regulation of transcription from RNA polymerase II promoter	14 %
18	GO:0030528	transcription regulator activity	14 %
19	GO:0015179	L-amino acid transporter activity	14 %
20	GO:0016020	membrane	14 %

globalUp – chr2L: 13246230 - 13283986

Genomic components: 3 coregulated genes, 7 genes

CHR	Strand	Start	End	RefSeq	Name	Exons	Description
CHR2L	+	13246230	13250189	NM_135783	CG16820	4	CG16820-PA
CHR2L	+	13255636	13257426	NM_165031	CG31728	4	CG31728-PA
CHR2L	+	13257476	13258292	NM_165032	CG31851	1	CG31851-PA
CHR2L	-	13259147	13259635	NM_165033	CG31730	1	CG31730-PA
CHR2L	+	13261009	13283986	NM_135785	CG6043	11	CG6043-PD, isoform D
CHR2L	-	13265080	13265665	NM_165036	CG31848	1	CG31848-PA
CHR2L	+	13277375	13281617	NM_135786	CG6108	1	CG6108-PA

Cluster size: 37757 nucleotides

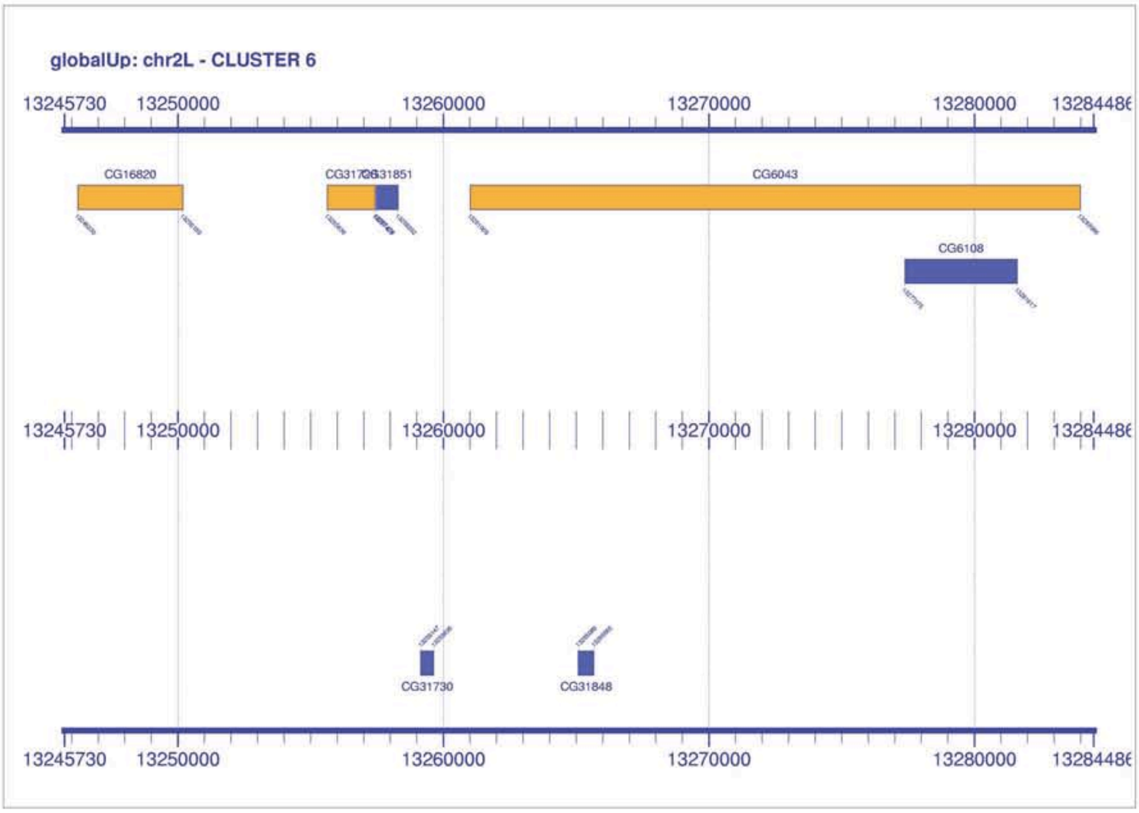

globalUp – cluster 6

Genomic components:

Name	RefSeq	Function	
CG16820	NM_135783		
CG31728	NM_165031	GO:0004295<br>GO:0006508<br>GO:0006952	trypsin activity<br>proteolysis<br>defense response
CG31851	NM_165032		
CG31730	NM_165033	GO:0006464<br>GO:0006473<br>GO:0008080<br>GO:0019538	protein modification<br>protein amino acid acetylation<br>N-acetyltransferase activity<br>protein metabolic process
CG6043	NM_135785		
CG31848	NM_165036	GO:0003674<br>GO:0005575<br>GO:0008150	molecular_function<br>cellular_component<br>biological_process
CG6108	NM_135786		

GO density (7 genes):

Ranking	GO id	Function	Frequency
1	GO:0008150	biological_process	14 %
2	GO:0004295	trypsin activity	14 %
3	GO:0006952	defense response	14 %
4	GO:0019538	protein metabolic process	14 %
5	GO:0006508	proteolysis	14 %
6	GO:0006464	protein modification	14 %
7	GO:0006473	protein amino acid acetylation	14 %
8	GO:0005575	cellular_component	14 %
9	GO:0008080	N-acetyltransferase activity	14 %
10	GO:0003674	molecular_function	14 %

globalUp – chr2L: 18134596 - 18154605

Genomic components: 3 coregulated genes, 5 genes

CHR	Strand	Start	End	RefSeq	Name	Exons	Description
CHR2L	-	18134596	18148339	NM_078869	Socs36E	5	Suppressor of cytokine signaling at 36E
CHR2L	+	18149345	18150339	NM_165244	CG17681	3	CG17681-PA
CHR2L	+	18150794	18151752	NM_136024	CG15155	4	CG15155-PA
CHR2L	+	18151971	18153091	NM_136025	CG5783	4	CG5783-PA
CHR2L	-	18153038	18154605	NM_136026	CG7200	2	CG7200-PA

Cluster size: 20010 nucleotides

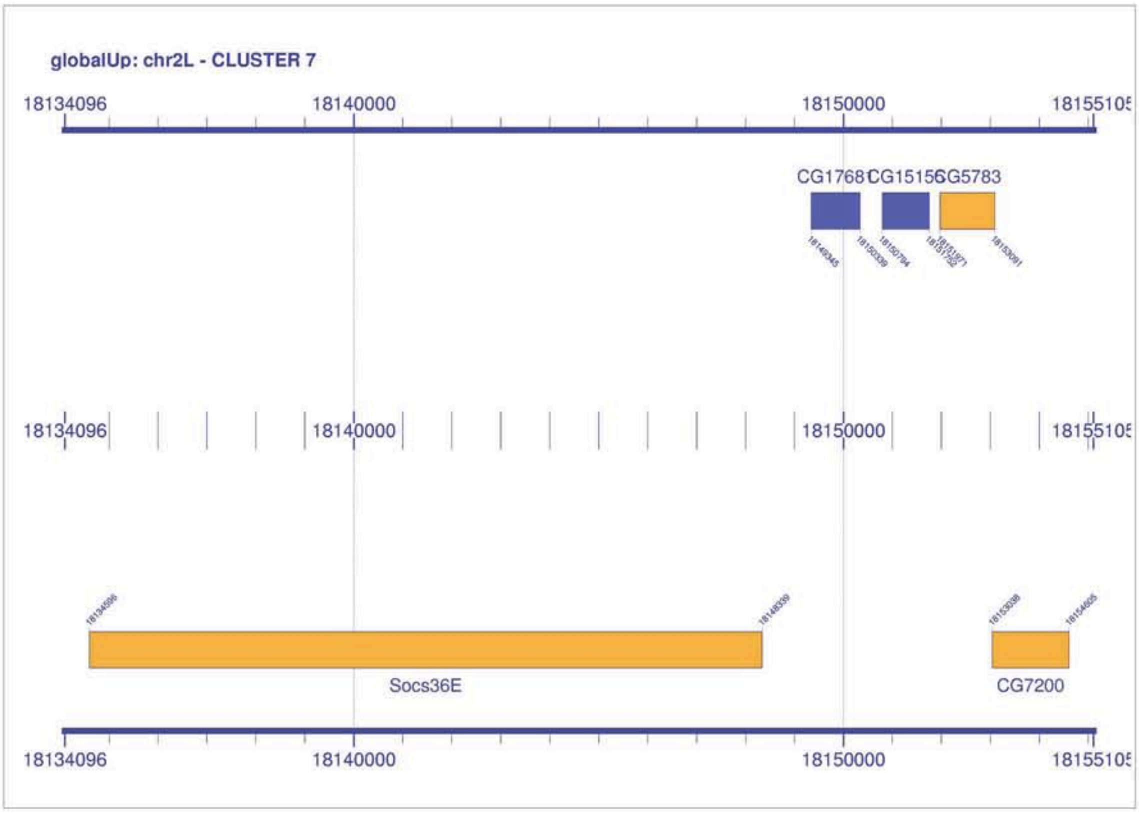

globalUp – cluster 7

Genomic components:

NAME	RefSeq	Function	
Socs36E	NM_078869	GO:0007259	JAK-STAT cascade
		GO:0007482	haltere development
		GO:0008586	imaginal disc-derived wing vein morphogenesis
		GO:0042059	negative regulation of epidermal growth factor receptor signaling pathway
		GO:0046425	regulation of JAK-STAT cascade
		GO:0046426	negative regulation of JAK-STAT cascade
		GO:0048072	compound eye pigmentation
		GO:0048802	notum morphogenesis
CG17681	NM_165244		
CG15155	NM_136024		
CG5783	NM_136025		
CG7200	NM_136026		

GO density (5 genes):

RANKING	GO id	Function	Frequency
1	GO:0048072	compound eye pigmentation	20 %
2	GO:0046426	negative regulation of JAK-STAT cascade	20 %
3	GO:0007482	haltere development	20 %
4	GO:0042059	negative regulation of epidermal growth factor receptor signaling pathway	20 %
5	GO:0008586	imaginal disc-derived wing vein morphogenesis	20 %
6	GO:0048802	notum morphogenesis	20 %
7	GO:0007259	JAK-STAT cascade	20 %
8	GO:0046425	regulation of JAK-STAT cascade	20 %

globalUp – chr2L: 19379144 - 19419478

Genomic components: 3 coregulated genes, 8 genes

CHR	Strand	Start	End	RefSeq	Name	Exons	Description
CHR2L	-	19379144	19382131	NM_136115	CG15825	5	CG15825-PB, isoform B
CHR2L	-	19382978	19385728	NM_136116	CG17549	6	CG17549-PA, isoform A
CHR2L	-	19386296	19394420	NM_136117	CG17544	6	CG17544-PA, isoform A
CHR2L	+	19390088	19391749	NM_165292	CG31798	3	CG31798-PA
CHR2L	-	19396709	19419478	NM_001038824	Pax	9	Paxillin CG31794-PG, isoform G
CHR2L	+	19413375	19414203	NM_057852	Lectin-galC1	2	Galactose-specific C-type lectin CG9976-PA
CHR2L	+	19414241	19414869	NM_001014489	lectin-37Da	2	lectin-37Da CG33532-PA
CHR2L	+	19415025	19415609	NM_001014490	lectin-37Db	2	lectin-37Db CG33533-PA

Cluster size: 40335 nucleotides

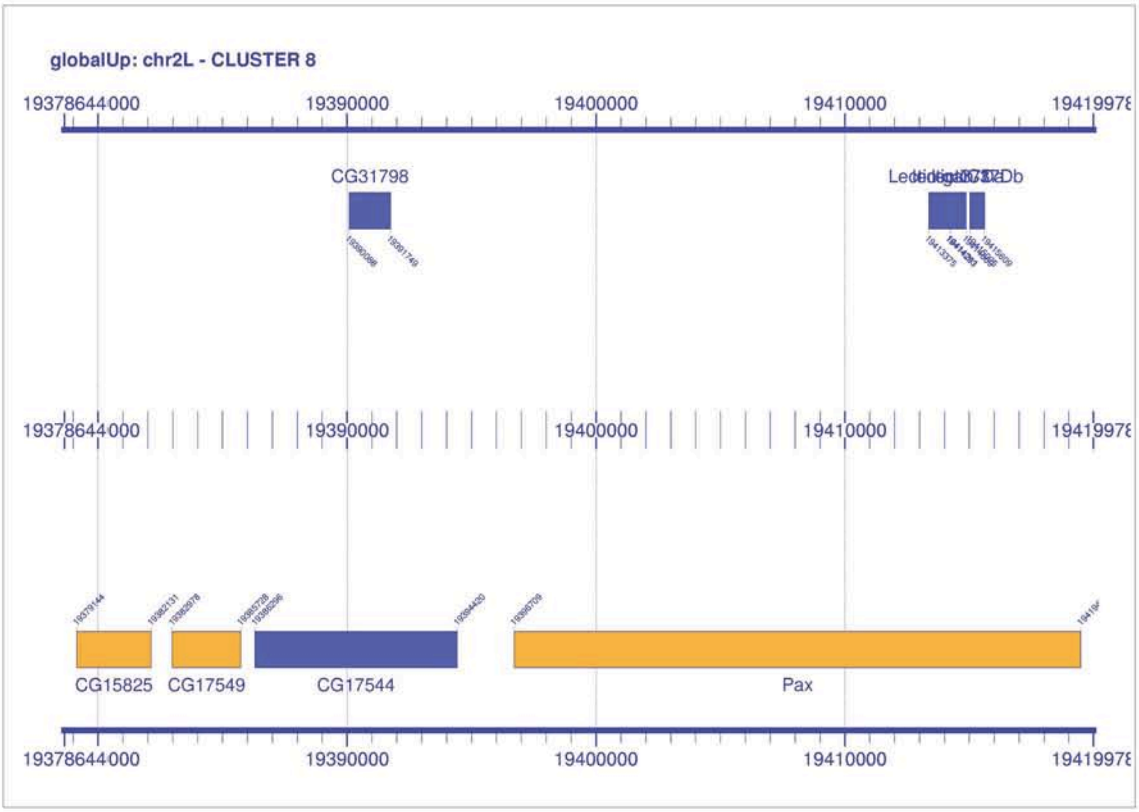

globalUp – cluster 8

Genomic components:

Name	RefSeq	Function	
CG15825	NM_136115		
CG17549	NM_136116		
CG17544	NM_136117	GO:0003995	acyl-CoA dehydrogenase activity
		GO:0005777	peroxisome
		GO:0006118	electron transport
		GO:0006635	fatty acid beta-oxidation
		GO:0016402	pristanoyl-CoA oxidase activity
		GO:0050660	FAD binding
CG31798	NM_165292	GO:0005488	binding
		GO:0005575	cellular_component
		GO:0008150	biological_process
Pax	NM_001038824	GO:0005200	structural constituent of cytoskeleton
		GO:0005925	focal adhesion
		GO:0006928	cell motility
		GO:0007016	cytoskeletal anchoring
		GO:0008270	zinc ion binding
Lectin-galC1	NM_057852	GO:0004872	receptor activity
		GO:0005529	sugar binding
		GO:0005534	galactose binding
		GO:0006952	defense response
lectin-37Da	NM_001014489	GO:0005534	galactose binding
lectin-37Db	NM_001014490	GO:0005534	galactose binding

GO density (8 genes):

Ranking	GO id	Function	Frequency
1	GO:0005534	galactose binding	37 %
2	GO:0008150	biological_process	12 %
3	GO:0003995	acyl-CoA dehydrogenase activity	12 %
4	GO:0006952	defense response	12 %
5	GO:0006928	cell motility	12 %
6	GO:0005488	binding	12 %
7	GO:0005200	structural constituent of cytoskeleton	12 %
8	GO:0004872	receptor activity	12 %
9	GO:0005529	sugar binding	12 %
10	GO:0016402	pristanoyl-CoA oxidase activity	12 %
11	GO:0005925	focal adhesion	12 %
12	GO:0005575	cellular_component	12 %
13	GO:0008270	zinc ion binding	12 %
14	GO:0006635	fatty acid beta-oxidation	12 %
15	GO:0005777	peroxisome	12 %
16	GO:0006118	electron transport	12 %
17	GO:0050660	FAD binding	12 %
18	GO:0007016	cytoskeletal anchoring	12 %

globalUp – chr2L: 20846761 - 20906521

Genomic components: 3 coregulated genes, 6 genes

CHR	Strand	Start	End	RefSeq	Name	Exons	Description
CHR2L	+	20846761	20848609	NM_136225	CG9336	3	CG9336-PA
CHR2L	+	20852819	20854904	NM_136227	CG9338	3	CG9338-PA
CHR2L	+	20855349	20857243	NM_165347	CG31675	3	CG31675-PA
CHR2L	+	20857439	20858646	NM_136228	CG14401	3	CG14401-PA
CHR2L	-	20858927	20906521	NM_136229	CG9339	12	CG9339-PA, isoform A
CHR2L	+	20888726	20889117	NM_206018	CG33317	2	CG33317-PA

Cluster size: 59761 nucleotides

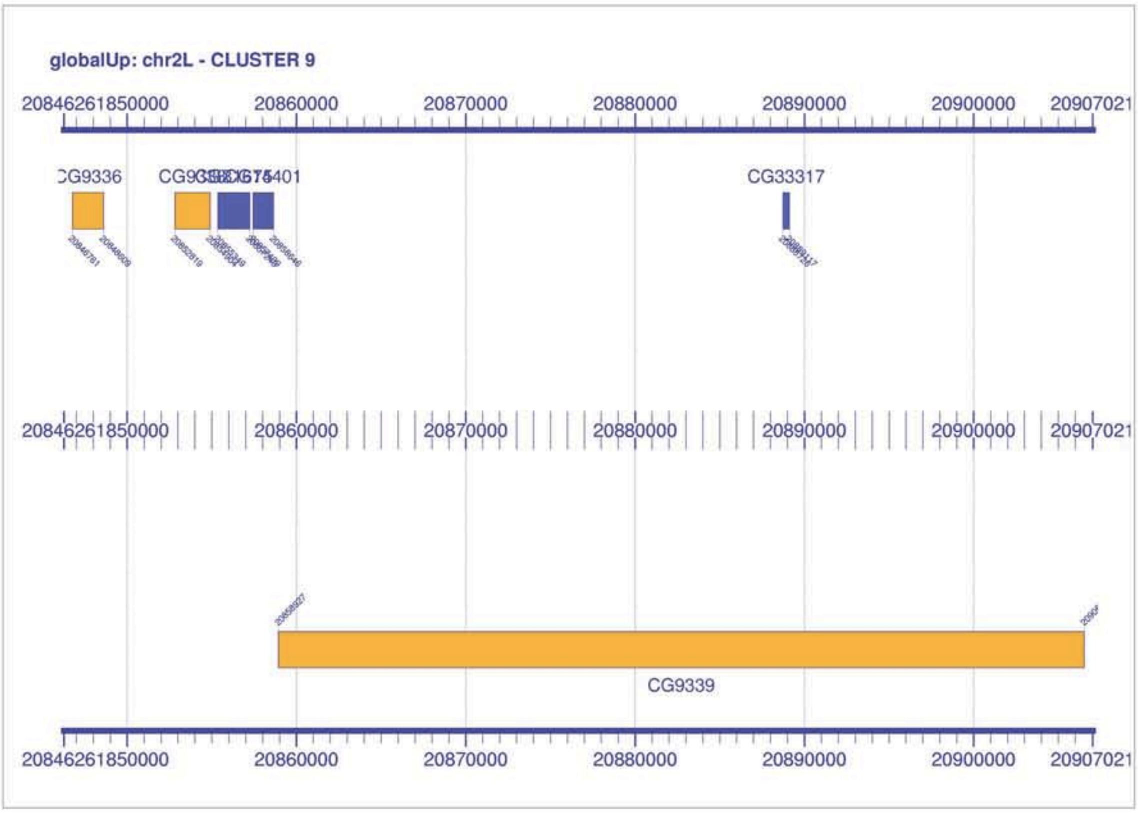

globalUp – cluster 9

Genomic components:

Name	RefSeq	Function	
CG9336	NM_136225		
CG9338	NM_136227		
CG31675	NM_165347	GO:0003674<br>GO:0005575<br>GO:0008150	molecular_function<br>cellular_component<br>biological_process
CG14401	NM_136228		
CG9339	NM_136229		
CG33317	NM_206018	GO:0003729	mRNA binding

GO density (6 genes):

Ranking	GO id	Function	Frequency
1	GO:0008150	biological_process	16 %
2	GO:0003729	mRNA binding	16 %
3	GO:0005575	cellular_component	16 %
4	GO:0003674	molecular_function	16 %

globalUp – chr3L: 4120317 - 4136008

Genomic components: 3 coregulated genes, 5 genes

Chr	Strand	Start	End	RefSeq	Name	Exons	Description
chr3L	-	4120317	4122733	NM_079192	Rop	1	Ras opposite CG15811-PA
chr3L	+	4122609	4124930	NM_079193	Ras64B	3	Ras oncogene at 64B CG1167-PA
chr3L	+	4125117	4125425	NM_079194	Akh	2	Adipokinetic hormone-like CG1171-PA
chr3L	+	4126623	4129384	NM_168065	CG32260	7	CG32260-PA
chr3L	+	4132228	4136008	NM_139605	CG1299	6	CG1299-PA

Cluster size: 15692 nucleotides

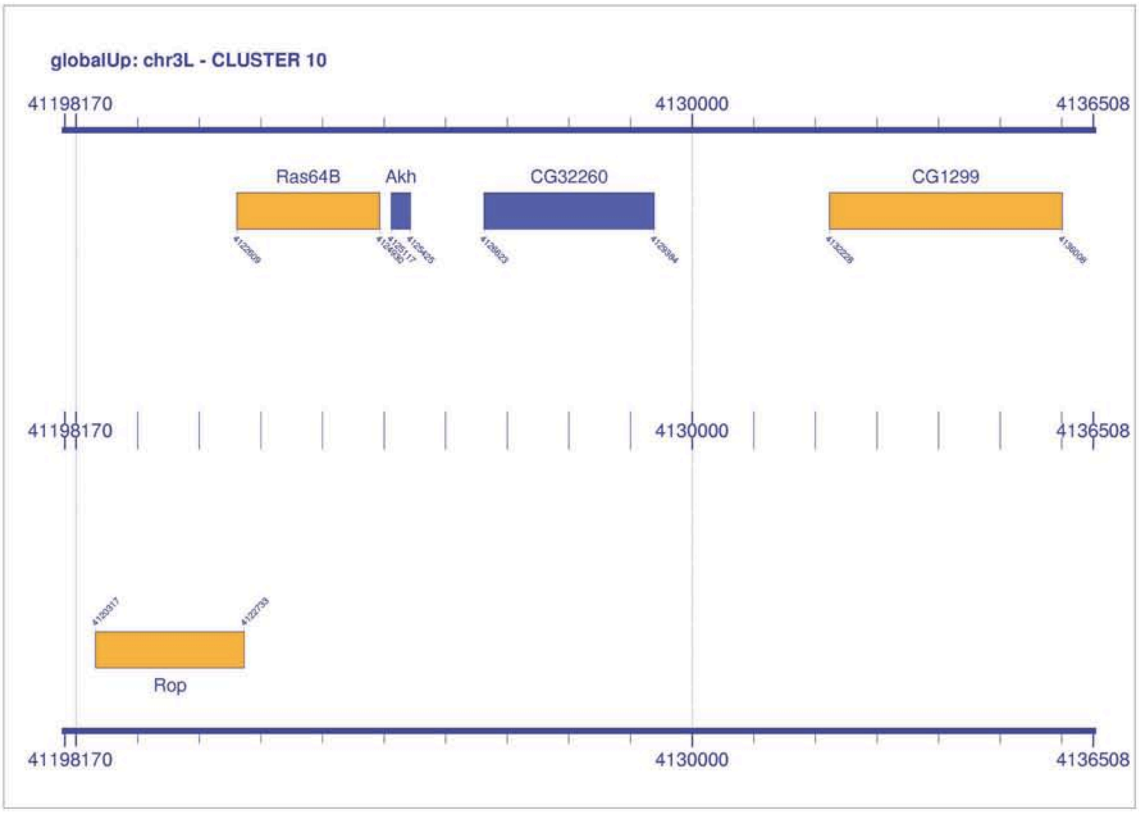

globalUp – cluster 10

Genomic components:

Name	RefSeq	Function	
Rop	NM_079192	GO:0000149	SNARE binding
		GO:0000910	cytokinesis
		GO:0005737	cytoplasm
		GO:0005829	cytosol
		GO:0006887	exocytosis
		GO:0006904	vesicle docking during exocytosis
		GO:0007268	synaptic transmission
		GO:0007269	neurotransmitter secretion
		GO:0007317	regulation of pole plasm oskar mRNA localization
		GO:0009416	response to light stimulus
		GO:0012506	vesicle membrane
		GO:0016020	membrane
		GO:0016082	synaptic vesicle priming
		GO:0016192	vesicle-mediated transport
		GO:0019905	syntaxin binding
		GO:0045045	secretory pathway
		GO:0048489	synaptic vesicle transport
Ras64B	NM_079193	GO:0003924	GTPase activity
		GO:0005525	GTP binding
		GO:0005622	intracellular
		GO:0006886	intracellular protein transport
		GO:0006898	receptor-mediated endocytosis
		GO:0006936	muscle contraction
		GO:0007010	cytoskeleton organization and biogenesis
		GO:0007186	G-protein coupled receptor protein signaling pathway
		GO:0007264	small GTPase mediated signal transduction
		GO:0007268	synaptic transmission
		GO:0008283	cell proliferation
		GO:0017157	regulation of exocytosis
		GO:0019003	GDP binding
Akh	NM_079194	GO:0005184	neuropeptide hormone activity
		GO:0005576	extracellular region
		GO:0007218	neuropeptide signaling pathway
CG32260	NM_168065	GO:0004252	serine-type endopeptidase activity
		GO:0006508	proteolysis
		GO:0006520	amino acid metabolic process
		GO:0006521	regulation of amino acid metabolic process
		GO:0008233	peptidase activity
		GO:0008236	serine-type peptidase activity
		GO:0008652	amino acid biosynthetic process
		GO:0019538	protein metabolic process
CG1299	NM_139605	GO:0004295	trypsin activity
		GO:0006508	proteolysis

GO density (5 genes):

RANKING	GO id	Function	Frequency
1	GO:0007268	synaptic transmission	40 %
2	GO:0006508	proteolysis	40 %
3	GO:0006887	exocytosis	20 %
4	GO:0019905	syntaxin binding	20 %
5	GO:0004295	trypsin activity	20 %
6	GO:0016082	synaptic vesicle priming	20 %
7	GO:0007317	regulation of pole plasm oskar mRNA localization	20 %
8	GO:0004252	serine-type endopeptidase activity	20 %
9	GO:0007186	G-protein coupled receptor protein signaling pathway	20 %
10	GO:0005576	extracellular region	20 %
11	GO:0007264	small GTPase mediated signal transduction	20 %
12	GO:0005829	cytosol	20 %
13	GO:0008283	cell proliferation	20 %
14	GO:0012506	vesicle membrane	20 %
15	GO:0016192	vesicle-mediated transport	20 %
16	GO:0009416	response to light stimulus	20 %
17	GO:0006904	vesicle docking during exocytosis	20 %
18	GO:0006521	regulation of amino acid metabolic process	20 %
19	GO:0048489	synaptic vesicle transport	20 %
20	GO:0008652	amino acid biosynthetic process	20 %
21	GO:0005525	GTP binding	20 %
22	GO:0045045	secretory pathway	20 %
23	GO:0007010	cytoskeleton organization and biogenesis	20 %
24	GO:0006898	receptor-mediated endocytosis	20 %
25	GO:0019538	protein metabolic process	20 %
26	GO:0006886	intracellular protein transport	20 %
27	GO:0003924	GTPase activity	20 %
28	GO:0000910	cytokinesis	20 %
29	GO:0005622	intracellular	20 %
30	GO:0006936	muscle contraction	20 %
31	GO:0019003	GDP binding	20 %
32	GO:0006520	amino acid metabolic process	20 %
33	GO:0007218	neuropeptide signaling pathway	20 %
34	GO:0007269	neurotransmitter secretion	20 %
35	GO:0017157	regulation of exocytosis	20 %
36	GO:0008236	serine-type peptidase activity	20 %
37	GO:0005737	cytoplasm	20 %
38	GO:0005184	neuropeptide hormone activity	20 %
39	GO:0008233	peptidase activity	20 %
40	GO:0000149	SNARE binding	20 %
41	GO:0016020	membrane	20 %

globalUp – chr3L: 5317569 - 5336745

Genomic components: 3 coregulated genes, 3 genes

Chr	Strand	Start	End	RefSeq	Name	Exons	Description
chr3L	-	5317569	5329236	NM_079209	lama	5	lamina ancestor CG10645-PB, isoform B
chr3L	-	5331168	5333201	NM_079210	Klp64D	1	Kinesin-like protein at 64D CG10642-PA
chr3L	+	5334136	5336745	NM_139701	CG4769	6	CG4769-PA

Cluster size: 19177 nucleotides

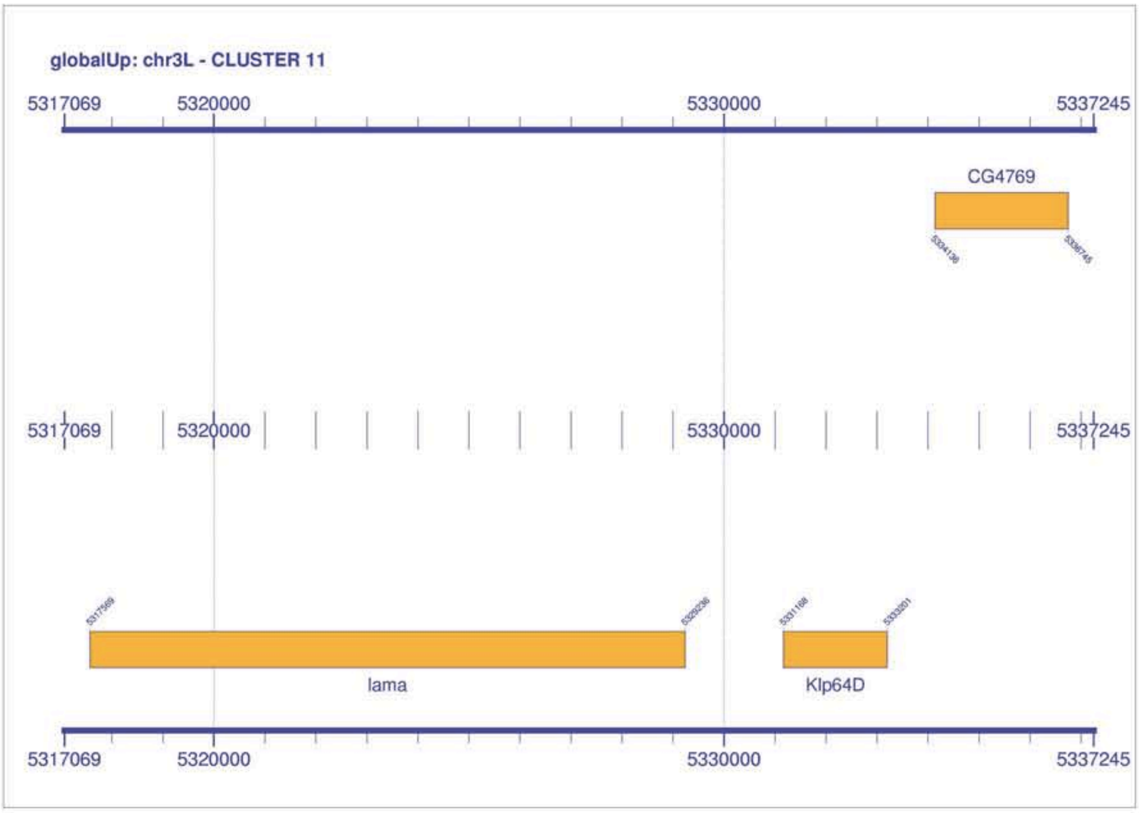

globalUp – cluster 11

Genomic components:

Name	RefSeq	Function	
LAMA	NM_079209	GO:0003674	molecular_function
		GO:0005575	cellular_component
		GO:0008150	biological_process
Klp64D	NM_079210	GO:0003777	microtubule motor activity
		GO:0005200	structural constituent of cytoskeleton
		GO:0005524	ATP binding
		GO:0005871	kinesin complex
		GO:0006605	protein targeting
		GO:0007018	microtubule-based movement
		GO:0035058	sensory cilium biogenesis
CG4769	NM_139701	GO:0005506	iron ion binding
		GO:0005750	mitochondrial respiratory chain complex III
		GO:0006119	oxidative phosphorylation
		GO:0006122	mitochondrial electron transport, ubiquinol to cytochrome c
		GO:0016491	oxidoreductase activity
		GO:0020037	heme binding
		GO:0045153	electron transporter, transferring electrons within CoQH2-cytochrome c reductase cor

GO density (3 genes):

Ranking	GO id	Function
1	GO:0045153	electron transporter, transferring electrons within CoQH2-cytochrome c reductase complex activity
2	GO:0005200	structural constituent of cytoskeleton
3	GO:0007018	microtubule-based movement
4	GO:0005575	cellular_component
5	GO:0005871	kinesin complex
6	GO:0016491	oxidoreductase activity
7	GO:0003674	molecular_function
8	GO:0006119	oxidative phosphorylation
9	GO:0005506	iron ion binding
10	GO:0005524	ATP binding
11	GO:0008150	biological_process
12	GO:0003777	microtubule motor activity
13	GO:0035058	sensory cilium biogenesis
14	GO:0005750	mitochondrial respiratory chain complex III
15	GO:0020037	heme binding
16	GO:0006605	protein targeting
17	GO:0006122	mitochondrial electron transport, ubiquinol to cytochrome c

globalUp – chr3L: 9684361 - 9707754

Genomic components: 3 coregulated genes, 9 genes

CHR	Strand	Start	End	RefSeq	Name	Exons	Description
CHR3L	-	9684361	9687770	NM_140091	SH3PX1	3	SH3PX1 CG6757-PA
CHR3L	+	9688266	9691663	NM_168362	vsg	2	visgun CG16707-PD, isoform D
CHR3L	-	9691732	9693088	NM_140093	CG18178	2	CG18178-PA
CHR3L	+	9693274	9694414	NM_140094	CG14174	2	CG14174-PA
CHR3L	-	9694358	9697395	NM_143716	nbs	4	nbs CG6754-PB
CHR3L	+	9697373	9700656	NM_140095	defl	2	deflated CG18176-PA
CHR3L	+	9700876	9703597	NM_140096	CG18177	6	CG18177-PB, isoform B
CHR3L	+	9703923	9705171	NM_079287	ATPsyn-b	3	ATP synthase, subunit b CG8189-PA, isoform A
CHR3L	-	9705184	9707754	NM_140097	CG6749	3	CG6749-PA

Cluster size: 23394 nucleotides

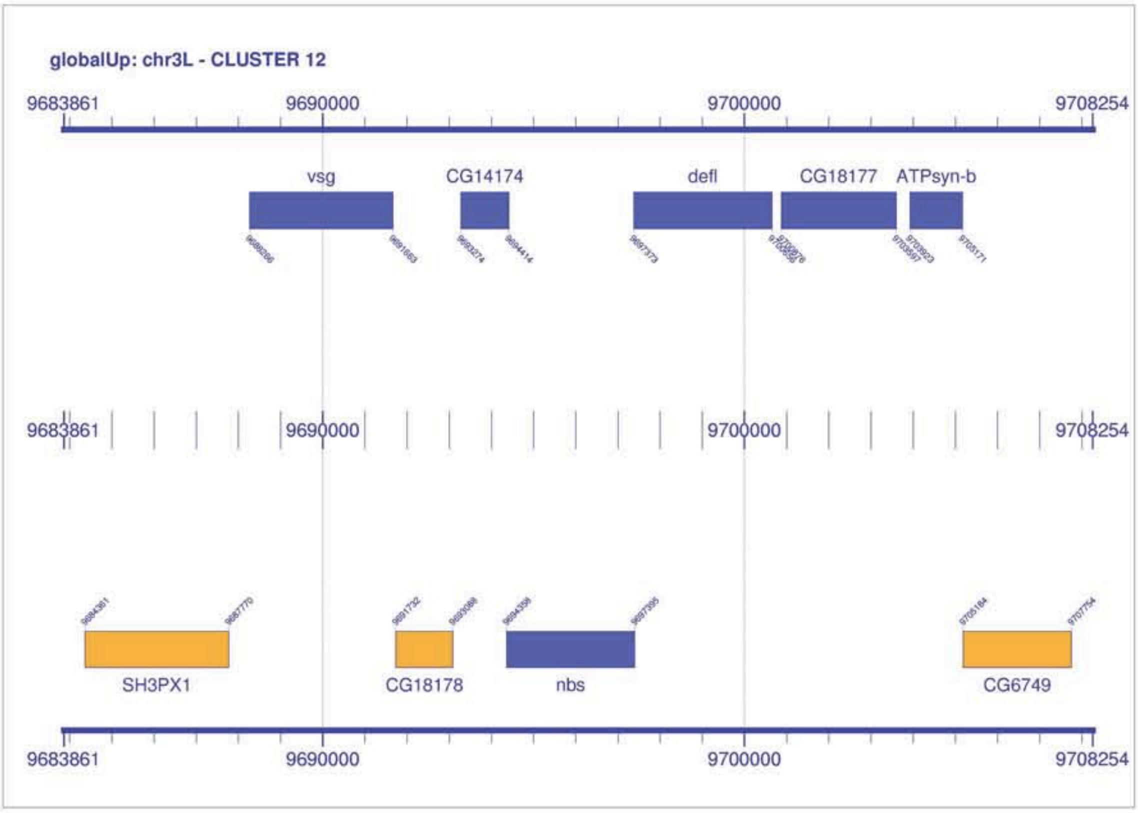

globalUp – cluster 12

Genomic components:

Name	RefSeq	Function	
SH3PX1	NM_140091	GO:0005515	protein binding
		GO:0006886	intracellular protein transport
		GO:0007242	intracellular signaling cascade
		GO:0035091	phosphoinositide binding
VSG	NM_168362	GO:0003674	molecular_function
		GO:0005575	cellular_component
		GO:0007611	learning and/or memory
		GO:0008355	olfactory learning
CG18178	NM_140093		
CG14174	NM_140094		
NBS	NM_143716	GO:0003676	nucleic acid binding
		GO:0003684	damaged DNA binding
		GO:0005622	intracellular
		GO:0006139	nucleobase, nucleoside, nucleotide and nucleic acid metabolic process
		GO:0006259	DNA metabolic process
		GO:0006281	DNA repair
		GO:0006310	DNA recombination
DEFL	NM_140095	GO:0003674	molecular_function
		GO:0005575	cellular_component
		GO:0008150	biological_process
CG18177	NM_140096		
ATPsyn-b	NM_079287	GO:0000276	mitochondrial proton-transporting ATP synthase complex, coupling factor F(o)
		GO:0008553	hydrogen-exporting ATPase activity, phosphorylative mechanism
		GO:0015986	ATP synthesis coupled proton transport
		GO:0015992	proton transport
		GO:0046933	hydrogen ion transporting ATP synthase activity, rotational mechanism
		GO:0046961	hydrogen ion transporting ATPase activity, rotational mechanism
CG6749	NM_140097	GO:0004872	receptor activity
		GO:0004930	G-protein coupled receptor activity
		GO:0006952	defense response
		GO:0007155	cell adhesion
		GO:0007165	signal transduction
		GO:0007166	cell surface receptor linked signal transduction
		GO:0007186	G-protein coupled receptor protein signaling pathway
		GO:0019221	cytokine and chemokine mediated signaling pathway
		GO:0019226	transmission of nerve impulse

GO density (9 genes):

Ranking	GO id	Function	Frequency
1	GO:0005575	cellular_component	22 %
2	GO:0003674	molecular_function	22 %
3	GO:0006952	defense response	11 %
4	GO:0015986	ATP synthesis coupled proton transport	11 %
5	GO:0008355	olfactory learning	11 %
6	GO:0003676	nucleic acid binding	11 %
7	GO:0004872	receptor activity	11 %
8	GO:0006281	DNA repair	11 %
9	GO:0007186	G-protein coupled receptor protein signaling pathway	11 %
10	GO:0046961	hydrogen ion transporting ATPase activity, rotational mechanism	11 %
11	GO:0007611	learning and/or memory	11 %
12	GO:0015992	proton transport	11 %
13	GO:0000276	mitochondrial proton-transporting ATP synthase complex, coupling factor F(o)	11 %
14	GO:0004930	G-protein coupled receptor activity	11 %
15	GO:0019226	transmission of nerve impulse	11 %
16	GO:0006310	DNA recombination	11 %
17	GO:0008150	biological_process	11 %
18	GO:0008553	hydrogen-exporting ATPase activity, phosphorylative mechanism	11 %
19	GO:0019221	cytokine and chemokine mediated signaling pathway	11 %
20	GO:0003684	damaged DNA binding	11 %
21	GO:0007155	cell adhesion	11 %
22	GO:0006886	intracellular protein transport	11 %
23	GO:0035091	phosphoinositide binding	11 %
24	GO:0006139	nucleobase, nucleoside, nucleotide and nucleic acid metabolic process	11 %
25	GO:0005622	intracellular	11 %
26	GO:0007165	signal transduction	11 %
27	GO:0046933	hydrogen ion transporting ATP synthase activity, rotational mechanism	11 %
28	GO:0007242	intracellular signaling cascade	11 %
29	GO:0005515	protein binding	11 %
30	GO:0006259	DNA metabolic process	11 %
31	GO:0007166	cell surface receptor linked signal transduction	11 %

globalUp – chr3L: 21131251 - 21153085

Genomic components: 3 coregulated genes, 7 genes

CHR	Strand	Start	End	RefSeq	Name	Exons	Description
CHR3L	-	21131251	21132013	NM_176375	CG33054	3	CG33054-PB, isoform B
CHR3L	-	21132097	21134524	NM_176377	CG33056	5	CG33056-PC, isoform C
CHR3L	+	21136760	21141933	NM_141032	CG10512	3	CG10512-PA, isoform A
CHR3L	-	21141993	21143925	NM_141034	CG10510	3	CG10510-PA
CHR3L	+	21144069	21149892	NM_176382	CG10508	9	CG10508-PD, isoform D
CHR3L	-	21149832	21150665	NM_141036	CG12975	3	CG12975-PA
CHR3L	+	21150734	21153085	NM_080262	Ilk	4	Integrin linked kinase CG10504-PA

Cluster size: 21835 nucleotides

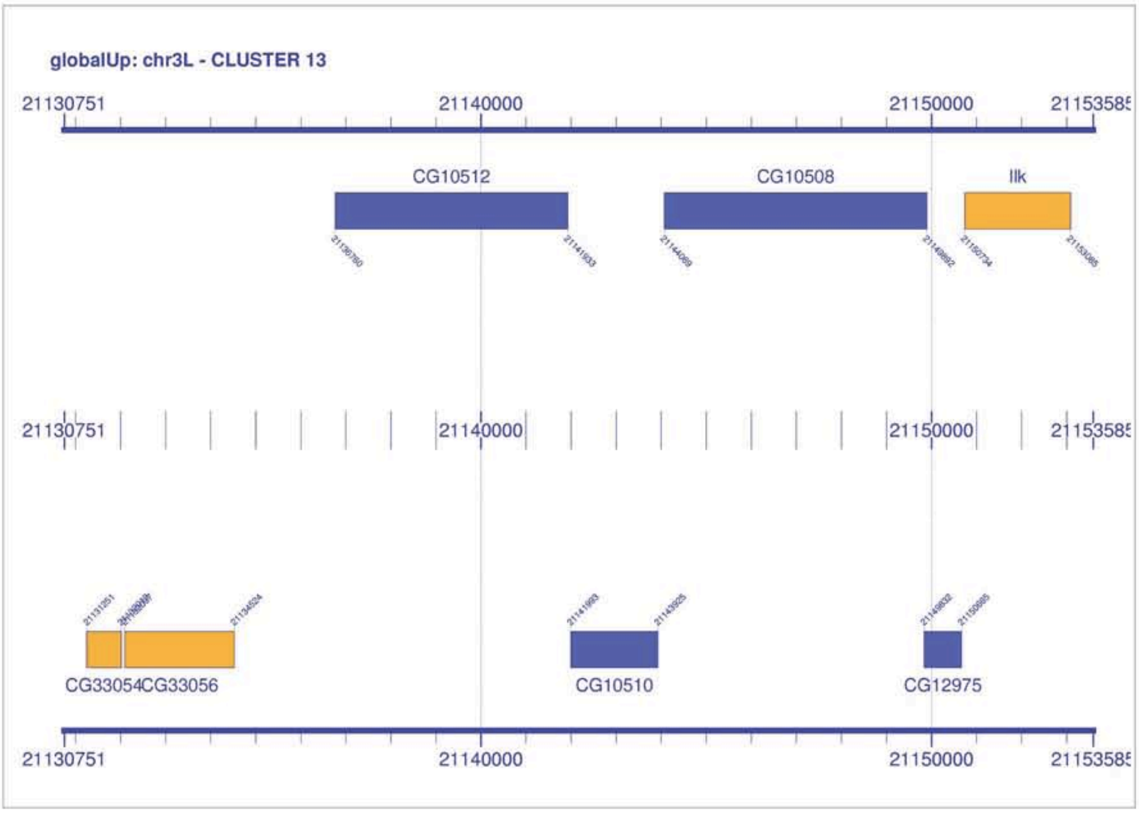

globalUp – cluster 13

Genomic components:

Name	RefSeq	Function	
CG33054	NM_176375		
CG33056	NM_176377		
CG10512	NM_141032	GO:0005975	carbohydrate metabolic process
		GO:0006099	tricarboxylic acid cycle
		GO:0016491	oxidoreductase activity
CG10510	NM_141034		
CG10508	NM_176382		
CG12975	NM_141036	GO:0003674	molecular_function
		GO:0005575	cellular_component
		GO:0008150	biological_process
Ilk	NM_080262	GO:0000165	MAPKKK cascade
		GO:0004672	protein kinase activity
		GO:0004674	protein serine/threonine kinase activity
		GO:0004702	receptor signaling protein serine/threonine kinase activity
		GO:0004713	protein-tyrosine kinase activity
		GO:0005524	ATP binding
		GO:0005925	focal adhesion
		GO:0006468	protein amino acid phosphorylation
		GO:0007016	cytoskeletal anchoring
		GO:0007155	cell adhesion
		GO:0007160	cell-matrix adhesion
		GO:0007229	integrin-mediated signaling pathway
		GO:0007475	apposition of dorsal and ventral imaginal disc-derived wing surfaces
		GO:0016203	muscle attachment

GO density (7 genes):

Ranking	GO id	Function	Frequency
1	GO:0004674	protein serine/threonine kinase activity	14 %
2	GO:0000165	MAPKKK cascade	14 %
3	GO:0005575	cellular_component	14 %
4	GO:0016491	oxidoreductase activity	14 %
5	GO:0005975	carbohydrate metabolic process	14 %
6	GO:0007229	integrin-mediated signaling pathway	14 %
7	GO:0006099	tricarboxylic acid cycle	14 %
8	GO:0003674	molecular_function	14 %
9	GO:0005524	ATP binding	14 %
10	GO:0008150	biological_process	14 %
11	GO:0007155	cell adhesion	14 %
12	GO:0007160	cell-matrix adhesion	14 %
13	GO:0006468	protein amino acid phosphorylation	14 %
14	GO:0007475	apposition of dorsal and ventral imaginal disc-derived wing surfaces	14 %
15	GO:0005925	focal adhesion	14 %
16	GO:0016203	muscle attachment	14 %
17	GO:0004702	receptor signaling protein serine/threonine kinase activity	14 %
18	GO:0004672	protein kinase activity	14 %
19	GO:0004713	protein-tyrosine kinase activity	14 %
20	GO:0007016	cytoskeletal anchoring	14 %

globalUp – chr3L: 22797706 - 22807564

Genomic components: 3 coregulated genes, 5 genes

CHR	Strand	Start	End	RefSeq	Name	Exons	Description
CHR3L	+	22797706	22799050	NM_176389	CG33169	5	CG33169-PB, isoform B
CHR3L	+	22799326	22801386	NM_176391	CG33170	4	CG33170-PA
CHR3L	-	22801323	22802075	NM_141167	CG11137	3	CG11137-PA
CHR3L	-	22802344	22805596	NM_057607	Arf79F	5	ADP ribosylation factor 79F CG8385-PB, isoform
CHR3L	+	22806046	22807564	NM_168974	CG11109	2	CG11109-PB, isoform B

Cluster size: 9859 nucleotides

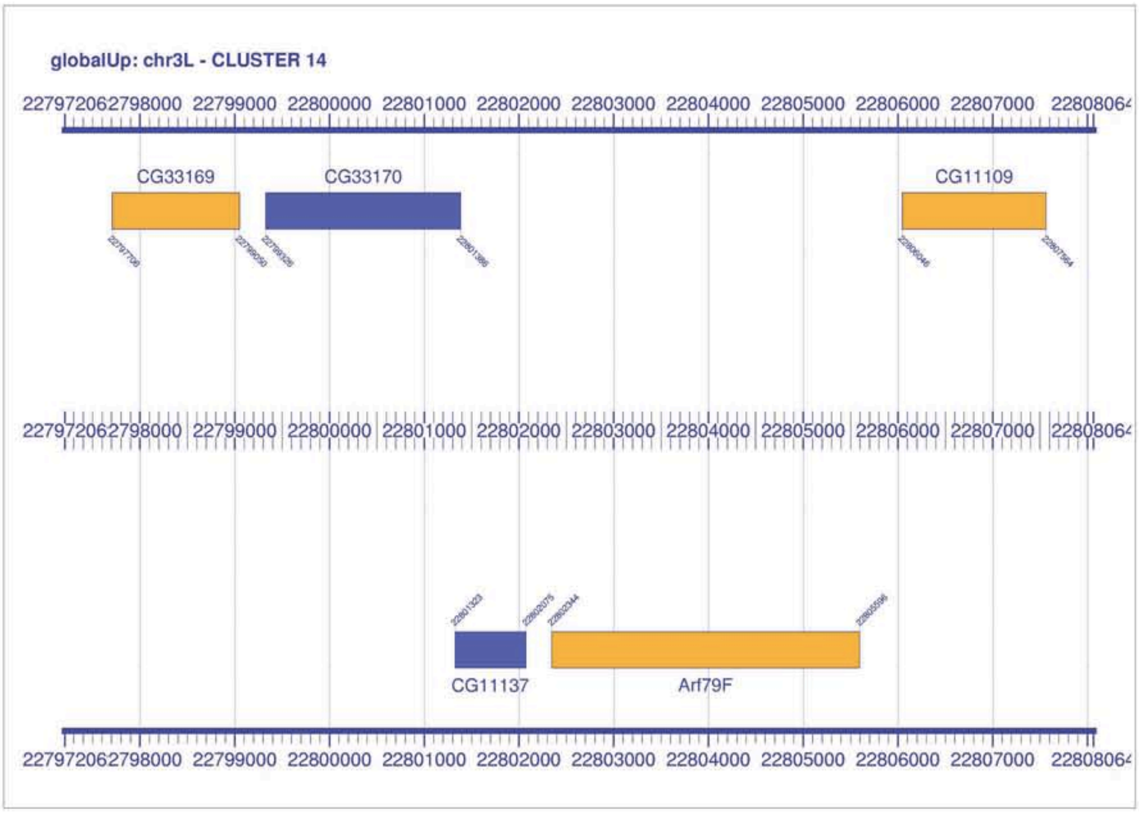

globalUp – cluster 14

Genomic components:

Name	RefSeq	Function	
CG33169	NM_176389		
CG33170	NM_176391		
CG11137	NM_141167		
Arf79F	NM_057607	GO:0003924	GTPase activity
		GO:0005525	GTP binding
		GO:0005622	intracellular
		GO:0006364	rRNA processing
		GO:0006471	protein amino acid ADP-ribosylation
		GO:0006886	intracellular protein transport
		GO:0007155	cell adhesion
		GO:0007166	cell surface receptor linked signal transduction
		GO:0007264	small GTPase mediated signal transduction
		GO:0007269	neurotransmitter secretion
		GO:0008360	regulation of cell shape
		GO:0016197	endosome transport
		GO:0048488	synaptic vesicle endocytosis
CG11109	NM_168974	GO:0003676	nucleic acid binding
		GO:0005730	nucleolus
		GO:0008649	rRNA methyltransferase activity
		GO:0016072	rRNA metabolic process

GO density (5 genes):

Ranking	GO id	Function	Frequency
1	GO:0003676	nucleic acid binding	20 %
2	GO:0048488	synaptic vesicle endocytosis	20 %
3	GO:0016197	endosome transport	20 %
4	GO:0006471	protein amino acid ADP-ribosylation	20 %
5	GO:0007264	small GTPase mediated signal transduction	20 %
6	GO:0005730	nucleolus	20 %
7	GO:0016072	rRNA metabolic process	20 %
8	GO:0005525	GTP binding	20 %
9	GO:0008649	rRNA methyltransferase activity	20 %
10	GO:0007155	cell adhesion	20 %
11	GO:0006886	intracellular protein transport	20 %
12	GO:0003924	GTPase activity	20 %
13	GO:0006364	rRNA processing	20 %
14	GO:0008360	regulation of cell shape	20 %
15	GO:0005622	intracellular	20 %
16	GO:0007269	neurotransmitter secretion	20 %
17	GO:0007166	cell surface receptor linked signal transduction	20 %

globalUp – chr2R: 2536824 - 2563777

Genomic components: 5 coregulated genes, 10 genes

CHR	Strand	Start	End	RefSeq	Name	Exons	Description
CHR2R	+	2536824	2539895	NM_078908	Tsp42Ef	5	Tetraspanin 42Ef CG12845-PA
CHR2R	+	2540725	2545046	NM_078909	Tsp42Eg	5	Tetraspanin 42Eg CG12142-PA
CHR2R	+	2545375	2546732	NM_165505	Tsp42Eh	4	Tetraspanin 42Eh CG12844-PA, isoform A
CHR2R	+	2546939	2548767	NM_078911	Tsp42Ei	4	Tetraspanin 42Ei CG12843-PA
CHR2R	-	2548744	2549482	NM_001032223	CG33914	4	CG33914-PA
CHR2R	+	2551044	2551556	NM_136411	CG12842	2	CG12842-PA
CHR2R	+	2551951	2554575	NM_078912	Tsp42Ej	4	Tetraspanin 42Ej CG12143-PA
CHR2R	+	2554844	2556342	NM_078913	Tsp42Ek	5	Tetraspanin 42Ek CG12841-PA
CHR2R	+	2557585	2560357	NM_078914	Tsp42El	5	Tetraspanin 42El CG12840-PA
CHR2R	+	2561608	2563777	NM_078915	lbm	5	late bloomer CG2374-PA

Cluster size: 26954 nucleotides

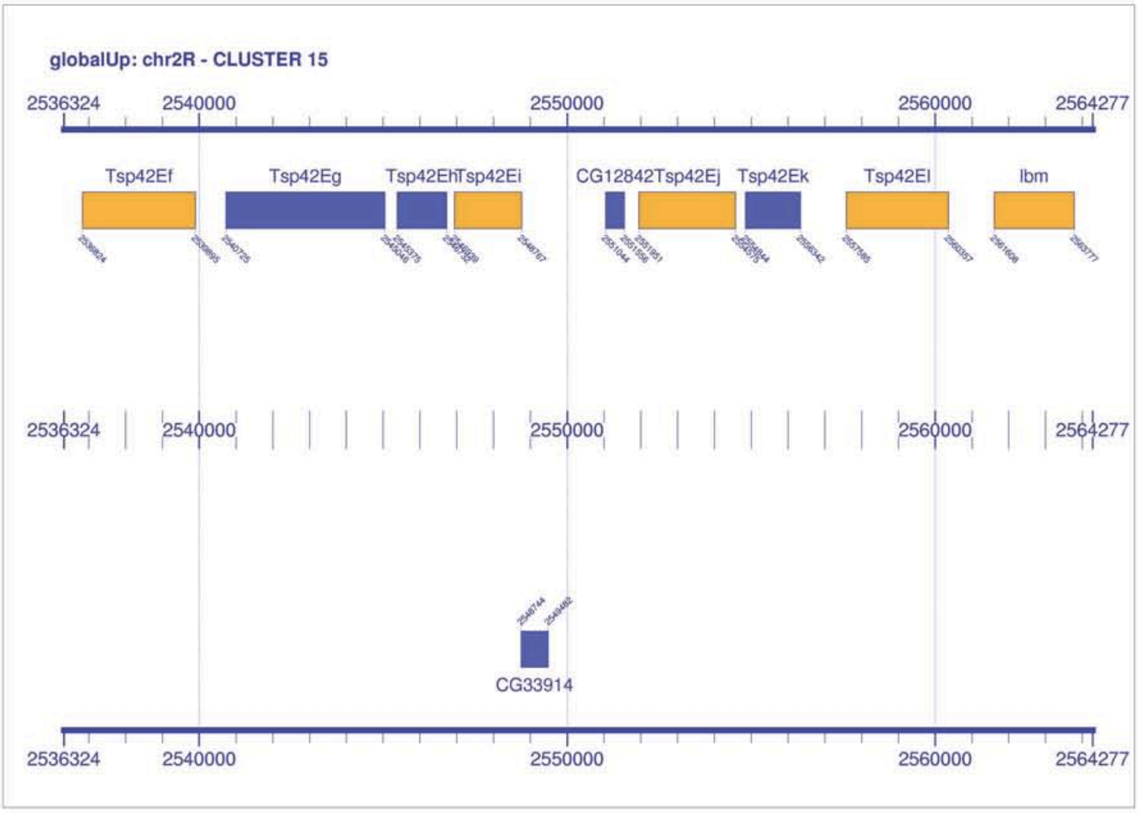

globalUp – cluster 15

Genomic components:

Name	RefSeq	Function	
Tsp42Ef	NM_078908	GO:0016021	integral to membrane
Tsp42Eg	NM_078909	GO:0016021	integral to membrane
Tsp42Eh	NM_165505	GO:0016021	integral to membrane
Tsp42Ei	NM_078911	GO:0016021	integral to membrane
CG33914	NM_001032223	GO:0003674<br>GO:0005575<br>GO:0008150	molecular_function<br>cellular_component<br>biological_process
CG12842	NM_136411		
Tsp42Ej	NM_078912	GO:0016021	integral to membrane
Tsp42Ek	NM_078913	GO:0005057<br>GO:0007398<br>GO:0007399<br>GO:0016021<br>GO:0019226	receptor signaling protein activity<br>ectoderm development<br>nervous system development<br>integral to membrane<br>transmission of nerve impulse
Tsp42El	NM_078914	GO:0005057<br>GO:0007398<br>GO:0007399<br>GO:0016021<br>GO:0019226	receptor signaling protein activity<br>ectoderm development<br>nervous system development<br>integral to membrane<br>transmission of nerve impulse
lbm	NM_078915	GO:0005057<br>GO:0005576<br>GO:0007398<br>GO:0007399<br>GO:0007416<br>GO:0016021<br>GO:0019226	receptor signaling protein activity<br>extracellular region<br>ectoderm development<br>nervous system development<br>synaptogenesis<br>integral to membrane<br>transmission of nerve impulse

GO density (10 genes):

Ranking	GO id	Function	Frequency
1	GO:0016021	integral to membrane	80 %
2	GO:0005057	receptor signaling protein activity	30 %
3	GO:0019226	transmission of nerve impulse	30 %
4	GO:0007399	nervous system development	30 %
5	GO:0007398	ectoderm development	30 %
6	GO:0005575	cellular_component	10 %
7	GO:0005576	extracellular region	10 %
8	GO:0003674	molecular_function	10 %
9	GO:0007416	synaptogenesis	10 %
10	GO:0008150	biological_process	10 %

globalUp – chr2R: 3303710 - 3335381

Genomic components: 4 coregulated genes, 7 genes

CHR	Strand	Start	End	RefSeq	Name	Exons	Description
CHR2R	-	3303710	3304995	NM_001038847	Gapdh1	1	Glyceraldehyde 3 phosphate dehydrogenase 1
CHR2R	+	3305064	3312857	NM_080142	mus205	12	mutagen-sensitive 205 CG1925-PA
CHR2R	-	3312593	3318212	NM_136480	CG1553	5	CG1553-PA, isoform A
CHR2R	+	3318290	3321880	NM_078928	sax	7	saxophone CG1891-PA, isoform A
CHR2R	-	3321887	3323848	NM_136481	CG1550	1	CG1550-PA
CHR2R	+	3324318	3326558	NM_136482	CG1882	2	CG1882-PA, isoform A
CHR2R	-	3333923	3335381	NM_143756	cathD	2	cathD CG1548-PA

Cluster size: 31672 nucleotides

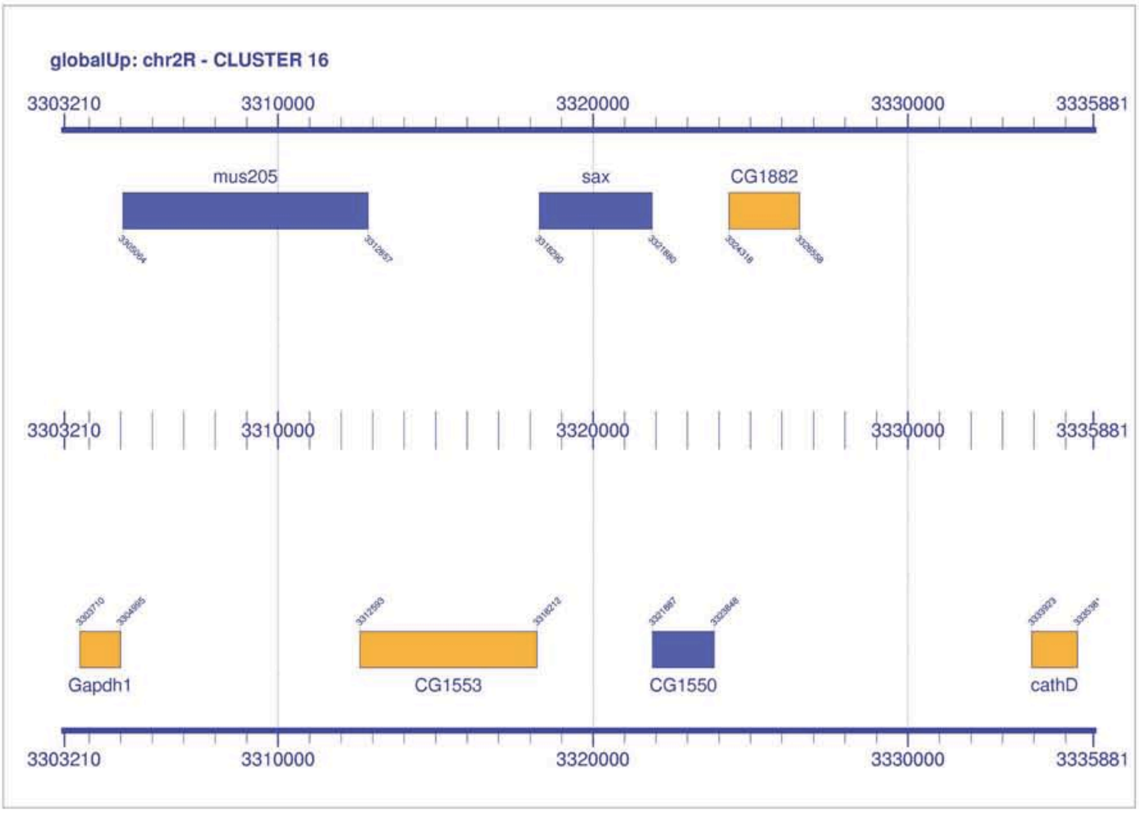

globalUp – cluster 16

Genomic components:

Name	RefSeq	Function	
Gapdh1	NM_001038847	GO:0004365	glyceraldehyde-3-phosphate dehydrogenase (phosphorylating) activity
		GO:0005737	cytoplasm
		GO:0006096	glycolysis
		GO:0051287	NAD binding
mus205	NM_080142	GO:0000166	nucleotide binding
		GO:0003676	nucleic acid binding
		GO:0003677	DNA binding
		GO:0003887	DNA-directed DNA polymerase activity
		GO:0003894	zeta DNA polymerase activity
		GO:0006260	DNA replication
		GO:0008408	3’-5’ exonuclease activity
		GO:0016035	zeta DNA polymerase complex
		GO:0019985	bypass DNA synthesis
CG1553	NM_136480		
sax	NM_078928	GO:0001745	compound eye morphogenesis
		GO:0004672	protein kinase activity
		GO:0004674	protein serine/threonine kinase activity
		GO:0004896	hematopoietin/interferon-class (D200-domain) cytokine receptor activity
		GO:0005025	transforming growth factor beta receptor activity, type I
		GO:0005524	ATP binding
		GO:0005886	plasma membrane
		GO:0006468	protein amino acid phosphorylation
		GO:0007179	transforming growth factor beta receptor signaling pathway
		GO:0007300	nurse cell to oocyte transport (sensu Insecta)
		GO:0007448	anterior/posterior pattern formation, imaginal disc
		GO:0007476	imaginal disc-derived wing morphogenesis
		GO:0007498	mesoderm development
		GO:0008358	maternal determination of anterior/posterior axis, embryo
		GO:0009993	oogenesis (sensu Insecta)
		GO:0019221	cytokine and chemokine mediated signaling pathway
		GO:0030509	BMP signaling pathway
		GO:0030707	ovarian follicle cell development (sensu Insecta)
		GO:0030718	germ-line stem cell maintenance
		GO:0042078	germ-line stem cell division
		GO:0045887	positive regulation of synaptic growth at neuromuscular junction
CG1550	NM_136481	GO:0004835	tubulin-tyrosine ligase activity
		GO:0006464	protein modification
CG1882	NM_136482	GO:0006725	aromatic compound metabolic process
		GO:0016787	hydrolase activity
cathD	NM_143756	GO:0004192	cathepsin D activity
		GO:0004194	pepsin A activity
		GO:0006508	proteolysis
		GO:0035071	salivary gland cell autophagic cell death
		GO:0048102	autophagic cell death

GO density (7 genes):

Ranking	GO id	Function	Frequency
1	GO:0007300	nurse cell to oocyte transport (sensu Insecta)	14 %
2	GO:0004674	protein serine/threonine kinase activity	14 %
3	GO:0006464	protein modification	14 %
4	GO:0003676	nucleic acid binding	14 %
5	GO:0035071	salivary gland cell autophagic cell death	14 %
6	GO:0051287	NAD binding	14 %
7	GO:0016035	zeta DNA polymerase complex	14 %
8	GO:0030707	ovarian follicle cell development (sensu Insecta)	14 %
9	GO:0004194	pepsin A activity	14 %
10	GO:0008358	maternal determination of anterior/posterior axis, embryo	14 %
11	GO:0001745	compound eye morphogenesis	14 %
12	GO:0007498	mesoderm development	14 %
13	GO:0005886	plasma membrane	14 %
14	GO:0048102	autophagic cell death	14 %
15	GO:0030718	germ-line stem cell maintenance	14 %
16	GO:0004192	cathepsin D activity	14 %
17	GO:0000166	nucleotide binding	14 %
18	GO:0008408	3'-5' exonuclease activity	14 %
19	GO:0007179	transforming growth factor beta receptor signaling pathway	14 %
20	GO:0009993	oogenesis (sensu Insecta)	14 %
21	GO:0005524	ATP binding	14 %
22	GO:0003677	DNA binding	14 %
23	GO:0004365	glyceraldehyde-3-phosphate dehydrogenase (phosphorylating) activity	14 %
24	GO:0045887	positive regulation of synaptic growth at neuromuscular junction	14 %
25	GO:0019221	cytokine and chemokine mediated signaling pathway	14 %
26	GO:0006508	proteolysis	14 %
27	GO:0003887	DNA-directed DNA polymerase activity	14 %
28	GO:0019985	bypass DNA synthesis	14 %
29	GO:0007476	imaginal disc-derived wing morphogenesis	14 %
30	GO:0030509	BMP signaling pathway	14 %
31	GO:0006468	protein amino acid phosphorylation	14 %
32	GO:0007448	anterior/posterior pattern formation, imaginal disc	14 %
33	GO:0042078	germ-line stem cell division	14 %
34	GO:0004672	protein kinase activity	14 %
35	GO:0016787	hydrolase activity	14 %
36	GO:0003894	zeta DNA polymerase activity	14 %
37	GO:0004835	tubulin-tyrosine ligase activity	14 %
38	GO:0005737	cytoplasm	14 %
39	GO:0006096	glycolysis	14 %
40	GO:0004896	hematopoietin/interferon-class (D200-domain) cytokine receptor activity	14 %
41	GO:0006725	aromatic compound metabolic process	14 %
42	GO:0006260	DNA replication	14 %
43	GO:0005025	transforming growth factor beta receptor activity, type I	14 %

globalUp – chr2R: 5590177 - 5611399

Genomic components: 3 coregulated genes, 5 genes

CHR	Strand	Start	End	RefSeq	Name	Exons	Description
CHR2R	+	5590177	5596030	NM_165735	egr	7	eiger CG12919-PA, isoform A
CHR2R	-	5597254	5598877	NM_078951	sut4	1	sugar transporter 4 CG1380-PA, isoform A
CHR2R	+	5599383	5608229	NM_165738	CG2269	6	CG2269-PC, isoform C
CHR2R	+	5608292	5610418	NM_165739	Jra	3	Jun-related antigen CG2275-PB, isoform B
CHR2R	-	5610328	5611399	NM_136710	CG1381	3	CG1381-PA

Cluster size: 21223 nucleotides

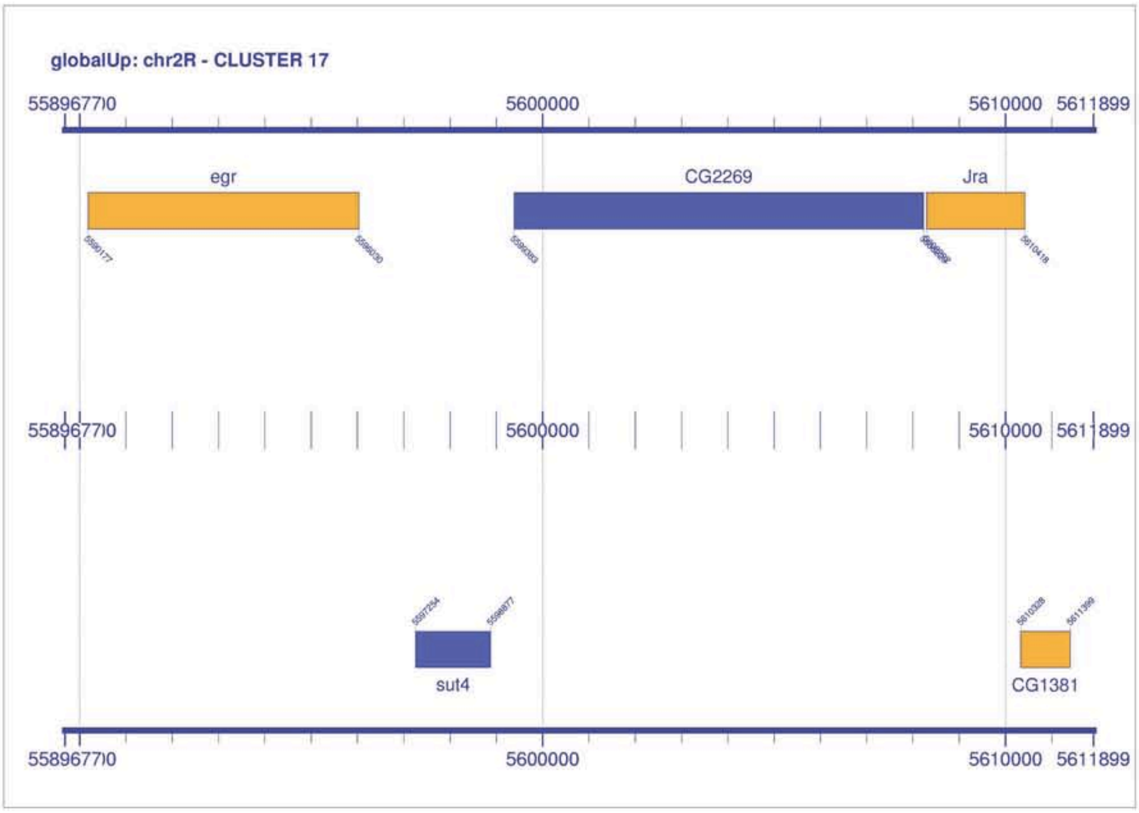

globalUp – cluster 17

Genomic components:

NAME	RefSeq	Function	
EGR	NM_165735	GO:0005164	tumor necrosis factor receptor binding
		GO:0005515	protein binding
		GO:0006915	apoptosis
		GO:0006917	induction of apoptosis
		GO:0006955	immune response
		GO:0007254	JNK cascade
		GO:0016020	membrane
SUT4	NM_078951	GO:0005351	sugar porter activity
		GO:0005355	glucose transporter activity
		GO:0005975	carbohydrate metabolic process
		GO:0008643	carbohydrate transport
		GO:0016020	membrane
		GO:0016021	integral to membrane
CG2269	NM_165738		
JRA	NM_165739	GO:0000165	MAPKKK cascade
		GO:0001736	establishment of planar polarity
		GO:0003700	transcription factor activity
		GO:0003702	RNA polymerase II transcription factor activity
		GO:0003704	specific RNA polymerase II transcription factor activity
		GO:0005515	protein binding
		GO:0005634	nucleus
		GO:0005737	cytoplasm
		GO:0006357	regulation of transcription from RNA polymerase II promoter
		GO:0007254	JNK cascade
		GO:0007391	dorsal closure
		GO:0007464	R3/R4 cell fate commitment
		GO:0007465	R7 cell fate commitment
		GO:0008134	transcription factor binding
		GO:0043565	sequence-specific DNA binding
		GO:0046843	dorsal appendage formation
		GO:0046844	micropyle formation
		GO:0046982	protein heterodimerization activity
CG1381	NM_136710	GO:0003676	nucleic acid binding
		GO:0003735	structural constituent of ribosome
		GO:0006412	translation
		GO:0019538	protein metabolic process
		GO:0042254	ribosome biogenesis and assembly

GO density (5 genes):

RANKING	GO id	Function	Frequency
1	GO:0007254	JNK cascade	40 %
2	GO:0005515	protein binding	40 %
3	GO:0016020	membrane	40 %
4	GO:0008134	transcription factor binding	20 %
5	GO:0006955	immune response	20 %
6	GO:0001736	establishment of planar polarity	20 %
7	GO:0003676	nucleic acid binding	20 %
8	GO:0005351	sugar porter activity	20 %
9	GO:0003702	RNA polymerase II transcription factor activity	20 %
10	GO:0000165	MAPKKK cascade	20 %
11	GO:0005634	nucleus	20 %
12	GO:0005975	carbohydrate metabolic process	20 %
13	GO:0016021	integral to membrane	20 %
14	GO:0043565	sequence-specific DNA binding	20 %
15	GO:0006357	regulation of transcription from RNA polymerase II promoter	20 %
16	GO:0042254	ribosome biogenesis and assembly	20 %
17	GO:0005164	tumor necrosis factor receptor binding	20 %
18	GO:0046982	protein heterodimerization activity	20 %
19	GO:0005355	glucose transporter activity	20 %
20	GO:0006412	translation	20 %
21	GO:0046844	micropyle formation	20 %
22	GO:0019538	protein metabolic process	20 %
23	GO:0008643	carbohydrate transport	20 %
24	GO:0003704	specific RNA polymerase II transcription factor activity	20 %
25	GO:0006915	apoptosis	20 %
26	GO:0007464	R3/R4 cell fate commitment	20 %
27	GO:0003700	transcription factor activity	20 %
28	GO:0003735	structural constituent of ribosome	20 %
29	GO:0006917	induction of apoptosis	20 %
30	GO:0007465	R7 cell fate commitment	20 %
31	GO:0005737	cytoplasm	20 %
32	GO:0007391	dorsal closure	20 %
33	GO:0046843	dorsal appendage formation	20 %

globalUp – chr2R: 6757647 - 6771890

Genomic components: 3 coregulated genes, 7 genes

Chr	Strand	Start	End	RefSeq	Name	Exons	Description
chr2R	-	6757647	6758102	NM_136806	CG9080	1	CG9080-PA
chr2R	+	6760196	6760910	NM_136807	CG13226	1	CG13226-PA
chr2R	+	6762023	6762561	NM_165812	CG30029	1	CG30029-PA
chr2R	+	6764032	6764517	NM_136809	CG7738	1	CG7738-PA
chr2R	+	6766274	6767820	NM_078965	Or47a	4	Odorant receptor 47a CG13225-PA
chr2R	-	6768035	6768798	NM_136810	CG9079	3	CG9079-PA
chr2R	+	6771075	6771890	NM_136811	CG13224	1	CG13224-PA

Cluster size: 14244 nucleotides

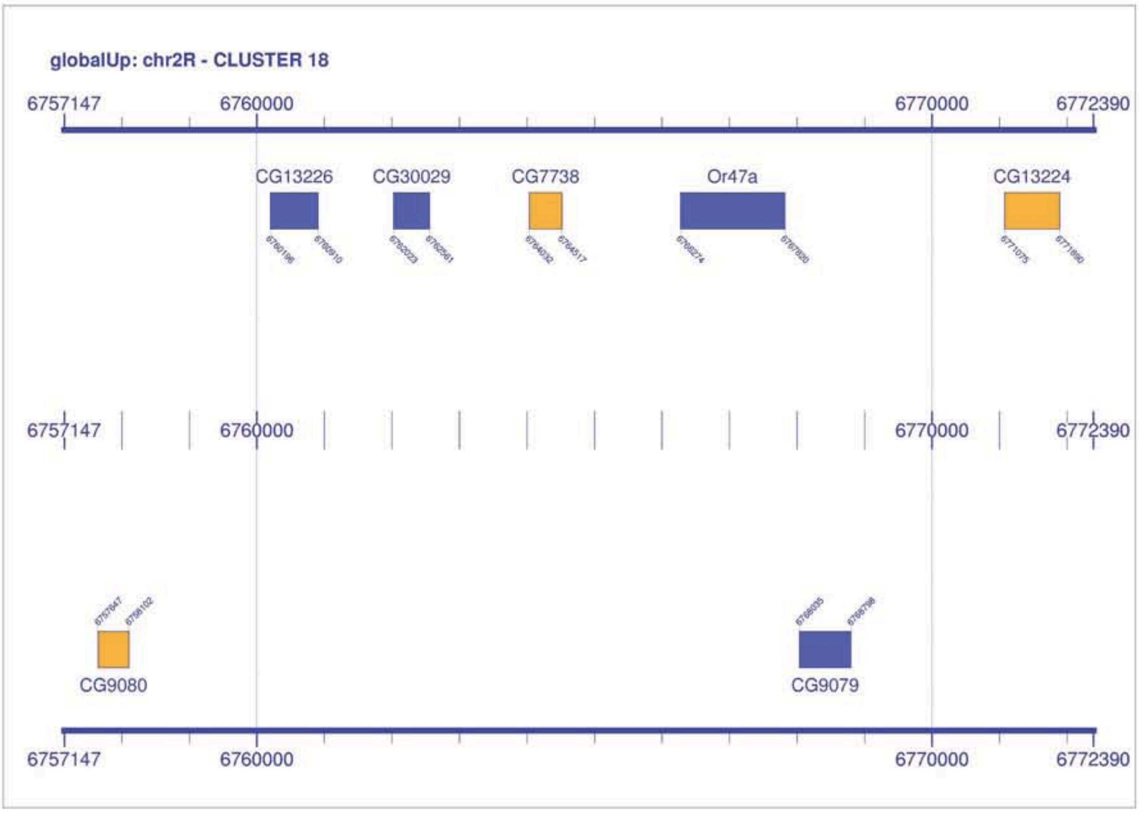

globalUp – cluster 18

Genomic components:

Name	RefSeq	Function	
CG9080	NM_136806		
CG13226	NM_136807		
CG30029	NM_165812		
CG7738	NM_136809		
Or47a	NM_078965	GO:0004984<br>GO:0005549<br>GO:0007608<br>GO:0016021	olfactory receptor activity<br>odorant binding<br>sensory perception of smell<br>integral to membrane
CG9079	NM_136810	GO:0005214	structural constituent of chitin-based cuticle
CG13224	NM_136811	GO:0008010	structural constituent of chitin-based larval cuticle

GO density (7 genes):

Ranking	GO id	Function	Frequency
1	GO:0004984	olfactory receptor activity	14 %
2	GO:0007608	sensory perception of smell	14 %
3	GO:0016021	integral to membrane	14 %
4	GO:0005214	structural constituent of chitin-based cuticle	14 %
5	GO:0005549	odorant binding	14 %
6	GO:0008010	structural constituent of chitin-based larval cuticle	14 %

globalUp – chr2R: 9342316 - 9362159

Genomic components: 3 coregulated genes, 5 genes

CHR	Strand	Start	End	RefSeq	Name	Exons	Description
CHR2R	+	9342316	9347280	NM_137060	CG6329	4	CG6329-PC, isoform C
CHR2R	-	9349760	9350995	NM_137061	CG6337	2	CG6337-PA
CHR2R	+	9352327	9355270	NM_137062	CG6347	3	CG6347-PA
CHR2R	+	9355982	9357650	NM_137063	CG6357	5	CG6357-PA
CHR2R	-	9357658	9362159	NM_137064	synaptogyrin	5	synaptogyrin CG10808-PA

Cluster size: 19844 nucleotides

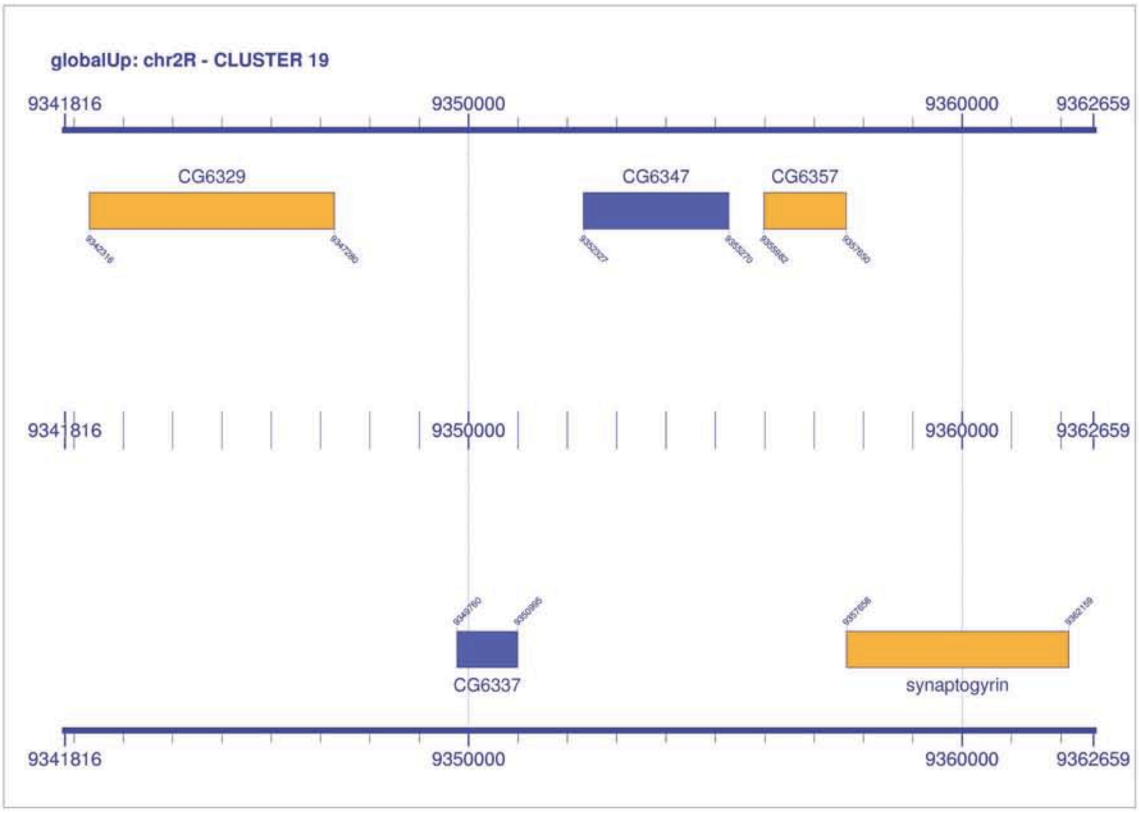

globalUp – cluster 19

Genomic components:

Name	RefSeq	Function	
CG6329	NM_137060		
CG6337	NM_137061	GO:0004197	cysteine-type endopeptidase activity
		GO:0006508	proteolysis
		GO:0019538	protein metabolic process
CG6347	NM_137062	GO:0006508	proteolysis
CG6357	NM_137063	GO:0004217	cathepsin L activity
SYNAPTOGYRIN	NM_137064	GO:0016020	membrane
		GO:0016079	synaptic vesicle exocytosis
		GO:0017158	regulation of calcium ion-dependent exocytosis

GO density (5 genes):

Ranking	GO id	Function	Frequency
1	GO:0006508	proteolysis	40 %
2	GO:0017158	regulation of calcium ion-dependent exocytosis	20 %
3	GO:0016079	synaptic vesicle exocytosis	20 %
4	GO:0004197	cysteine-type endopeptidase activity	20 %
5	GO:0019538	protein metabolic process	20 %
6	GO:0004217	cathepsin L activity	20 %
7	GO:0016020	membrane	20 %

globalUp – chr2R: 12513472 - 12522466

Genomic components: 3 coregulated genes, 5 genes

Chr	Strand	Start	End	RefSeq	Name	Exons	Description
chr2R	-	12513472	12514989	NM_137330	CG9001	2	CG9001-PA
chr2R	-	12515102	12516789	NM_137331	CG9000	2	CG9000-PA
chr2R	+	12516876	12518464	NM_137332	CG6796	3	CG6796-PA
chr2R	-	12518420	12519962	NM_137333	NiPp1	5	Nuclear inhibitor of Protein phosphatase 1
chr2R	+	12520315	12522466	NM_137334	CG6805	2	CG6805-PA

Cluster size: 8995 nucleotides

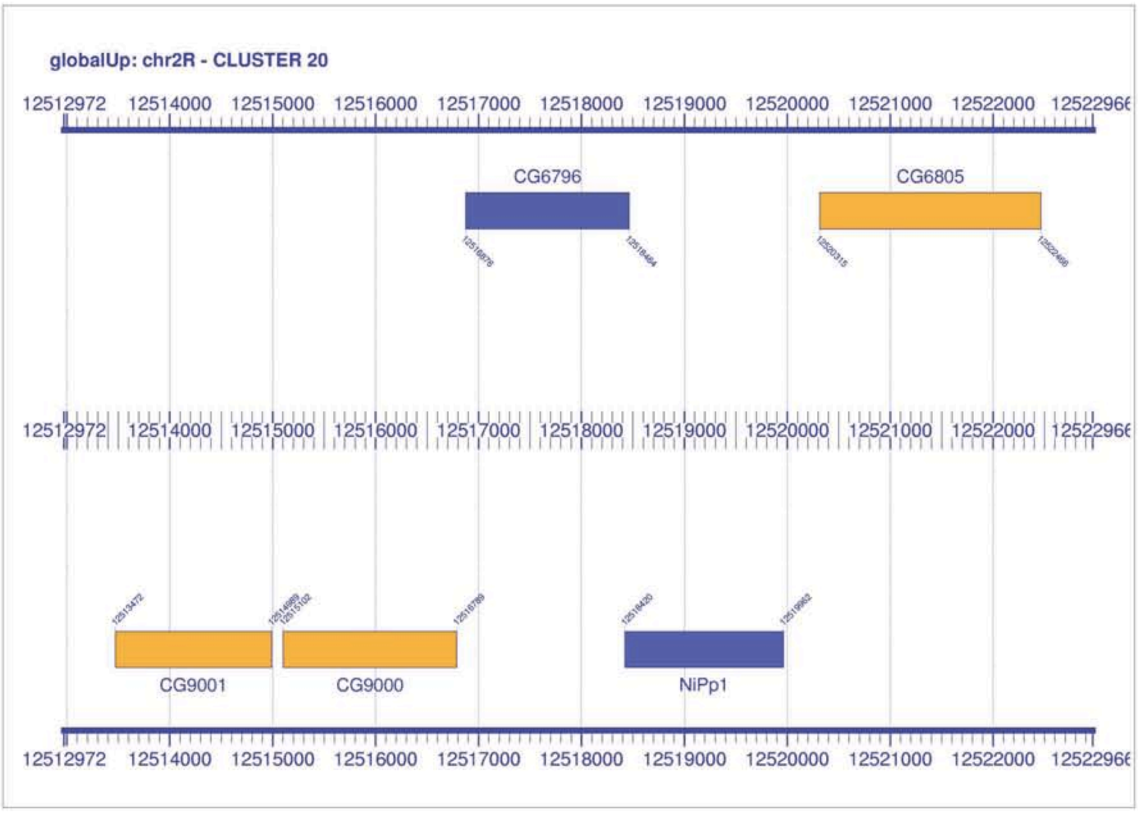

globalUp – cluster 20

Genomic components:

NAME	RefSeq	Function	
CG9001	NM_137330	GO:0006508	proteolysis
		GO:0007166	cell surface receptor linked signal transduction
		GO:0008487	prenyl-dependent CAAX protease activity
		GO:0016020	membrane
CG9000	NM_137331	GO:0006508	proteolysis
		GO:0007166	cell surface receptor linked signal transduction
		GO:0008487	prenyl-dependent CAAX protease activity
		GO:0016020	membrane
CG6796	NM_137332	GO:0003729	mRNA binding
		GO:0004815	aspartate-tRNA ligase activity
		GO:0004816	asparagine-tRNA ligase activity
		GO:0005524	ATP binding
		GO:0005737	cytoplasm
		GO:0006418	tRNA aminoacylation for protein translation
		GO:0006421	asparaginyl-tRNA aminoacylation
		GO:0006422	aspartyl-tRNA aminoacylation
NiPp1	NM_137333	GO:0003729	mRNA binding
		GO:0004864	protein phosphatase inhibitor activity
		GO:0004865	type 1 serine/threonine specific protein phosphatase inhibitor activity
		GO:0005634	nucleus
		GO:0007165	signal transduction
		GO:0016607	nuclear speck
		GO:0035308	negative regulation of protein amino acid dephosphorylation
CG6805	NM_137334	GO:0006629	lipid metabolic process
		GO:0007010	cytoskeleton organization and biogenesis
		GO:0016311	dephosphorylation
		GO:0019722	calcium-mediated signaling
		GO:0046030	inositol trisphosphate phosphatase activity

GO density (5 genes):

RANKING	GO id	Function	Frequency
1	GO:0006508	proteolysis	40 %
2	GO:0003729	mRNA binding	40 %
3	GO:0008487	prenyl-dependent CAAX protease activity	40 %
4	GO:0007166	cell surface receptor linked signal transduction	40 %
5	GO:0016020	membrane	40 %
6	GO:0004864	protein phosphatase inhibitor activity	20 %
7	GO:0005634	nucleus	20 %
8	GO:0016311	dephosphorylation	20 %
9	GO:0004815	aspartate-tRNA ligase activity	20 %
10	GO:0005524	ATP binding	20 %
11	GO:0006629	lipid metabolic process	20 %
12	GO:0004816	asparagine-tRNA ligase activity	20 %
13	GO:0007010	cytoskeleton organization and biogenesis	20 %
14	GO:0006418	tRNA aminoacylation for protein translation	20 %
15	GO:0035308	negative regulation of protein amino acid dephosphorylation	20 %
16	GO:0046030	inositol trisphosphate phosphatase activity	20 %
17	GO:0016607	nuclear speck	20 %
18	GO:0019722	calcium-mediated signaling	20 %
19	GO:0007165	signal transduction	20 %
20	GO:0004865	type 1 serine/threonine specific protein phosphatase inhibitor activity	20 %
21	GO:0006421	asparaginyl-tRNA aminoacylation	20 %
22	GO:0006422	aspartyl-tRNA aminoacylation	20 %
23	GO:0005737	cytoplasm	20 %

globalUp – chr2R: 16565372 - 16595808

Genomic components: 4 coregulated genes, 9 genes

Chr	Strand	Start	End	RefSeq	Name	Exons	Description
CHR2R	+	16565372	16567465	NM_137695	cpa	3	capping protein alpha CG10540-PA
CHR2R	-	16567481	16568050	NM_137696	CG15653	2	CG15653-PA
CHR2R	+	16568405	16569169	NM_137697	mRpL54	3	mitochondrial ribosomal protein L54 CG9353-PA
CHR2R	+	16569227	16570794	NM_137698	CG9357	3	CG9357-PA
CHR2R	-	16570759	16572545	NM_166420	CG30293	4	CG30293-PA
CHR2R	-	16572963	16574669	NM_080223	Cht4	5	Chitinase 4 CG3986-PA
CHR2R	-	16575573	16576854	NM_137699	CG10531	3	CG10531-PA
CHR2R	-	16577645	16580367	NM_137700	CG10527	3	CG10527-PA
CHR2R	+	16581606	16595808	NM_166422	Treh	7	Trehalase CG9364-PE, isoform E

Cluster size: 30437 nucleotides

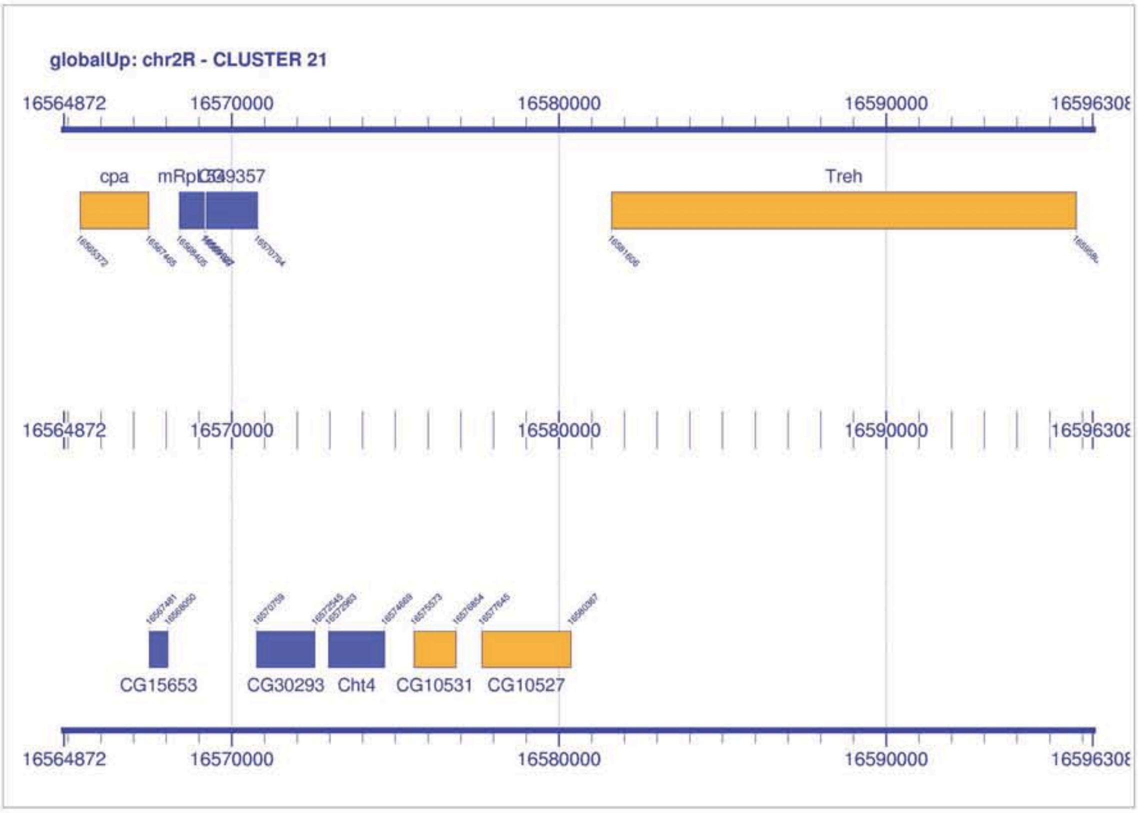

globalUp – cluster 21

Genomic components:

Name	RefSeq	Function	
CPA	NM_137695	GO:0003779	actin binding
		GO:0005200	structural constituent of cytoskeleton
		GO:0005869	dynactin complex
		GO:0005875	microtubule associated complex
		GO:0007015	actin filament organization
		GO:0007018	microtubule-based movement
		GO:0008290	F-actin capping protein complex
		GO:0030036	actin cytoskeleton organization and biogenesis
		GO:0051016	barbed-end actin filament capping
CG15653	NM_137696		
mRpL54	NM_137697	GO:0003735	structural constituent of ribosome
		GO:0005762	mitochondrial large ribosomal subunit
CG9357	NM_137698	GO:0004568	chitinase activity
		GO:0005576	extracellular region
		GO:0005976	polysaccharide metabolic process
		GO:0006030	chitin metabolic process
		GO:0006032	chitin catabolic process
		GO:0008061	chitin binding
		GO:0016799	hydrolase activity, hydrolyzing N-glycosyl compounds
CG30293	NM_166420	GO:0004568	chitinase activity
		GO:0005102	receptor binding
		GO:0005576	extracellular region
		GO:0005975	carbohydrate metabolic process
		GO:0005976	polysaccharide metabolic process
		GO:0006030	chitin metabolic process
		GO:0006032	chitin catabolic process
		GO:0007154	cell communication
		GO:0007165	signal transduction
		GO:0007267	cell-cell signaling
		GO:0007275	multicellular organismal development
		GO:0008061	chitin binding
		GO:0008083	growth factor activity
Cht4	NM_080223	GO:0004568	chitinase activity
		GO:0005576	extracellular region
		GO:0006036	cuticle chitin catabolic process
		GO:0007165	signal transduction
		GO:0007267	cell-cell signaling
		GO:0008061	chitin binding
		GO:0008083	growth factor activity
		GO:0016799	hydrolase activity, hydrolyzing N-glycosyl compounds
CG10531	NM_137699	GO:0004568	chitinase activity
		GO:0005976	polysaccharide metabolic process
		GO:0006032	chitin catabolic process
		GO:0007165	signal transduction
		GO:0007267	cell-cell signaling
		GO:0008083	growth factor activity
		GO:0016799	hydrolase activity, hydrolyzing N-glycosyl compounds
CG10527	NM_137700	GO:0019010	farnesoic acid O-methyltransferase activity
Treh	NM_166422	GO:0004555	alpha,alpha-trehalase activity
		GO:0005991	trehalose metabolic process
		GO:0016799	hydrolase activity, hydrolyzing N-glycosyl compounds
		GO:0046658	anchored to plasma membrane

GO density (9 genes):

RANKING	GO id	Function	Frequency
1	GO:0016799	hydrolase activity, hydrolyzing N-glycosyl compounds	44 %
2	GO:0004568	chitinase activity	44 %
3	GO:0007267	cell-cell signaling	33 %
4	GO:0005576	extracellular region	33 %
5	GO:0008061	chitin binding	33 %
6	GO:0005976	polysaccharide metabolic process	33 %
7	GO:0007165	signal transduction	33 %
8	GO:0006032	chitin catabolic process	33 %
9	GO:0008083	growth factor activity	33 %
10	GO:0006030	chitin metabolic process	22 %
11	GO:0006036	cuticle chitin catabolic process	11 %
12	GO:0005991	trehalose metabolic process	11 %
13	GO:0019010	farnesoic acid O-methyltransferase activity	11 %
14	GO:0003779	actin binding	11 %
15	GO:0005200	structural constituent of cytoskeleton	11 %
16	GO:0051016	barbed-end actin filament capping	11 %
17	GO:0007018	microtubule-based movement	11 %
18	GO:0046658	anchored to plasma membrane	11 %
19	GO:0005975	carbohydrate metabolic process	11 %
20	GO:0005875	microtubule associated complex	11 %
21	GO:0008290	F-actin capping protein complex	11 %
22	GO:0007275	multicellular organismal development	11 %
23	GO:0007154	cell communication	11 %
24	GO:0030036	actin cytoskeleton organization and biogenesis	11 %
25	GO:0004555	alpha,alpha-trehalase activity	11 %
26	GO:0003735	structural constituent of ribosome	11 %
27	GO:0005762	mitochondrial large ribosomal subunit	11 %
28	GO:0005869	dynactin complex	11 %
29	GO:0007015	actin filament organization	11 %
30	GO:0005102	receptor binding	11 %

globalUp – chr2R: 17587752 - 17620371

Genomic components: 4 coregulated genes, 5 genes

CHR	Strand	Start	End	RefSeq	Name	Exons	Description
CHR2R	+	17587752	17593349	NM_130462	synj	4	synaptojanin CG6562-PB, isoform B
CHR2R	-	17593250	17595213	NM_137793	CG13502	2	CG13502-PA, isoform A
CHR2R	-	17599065	17607308	NM_137794	CG11073	3	CG11073-PA
CHR2R	+	17610769	17613369	NM_137795	CG6613	6	CG6613-PA
CHR2R	-	17615162	17620371	NM_137796	CG13503	9	CG13503-PE, isoform E

Cluster size: 32620 nucleotides

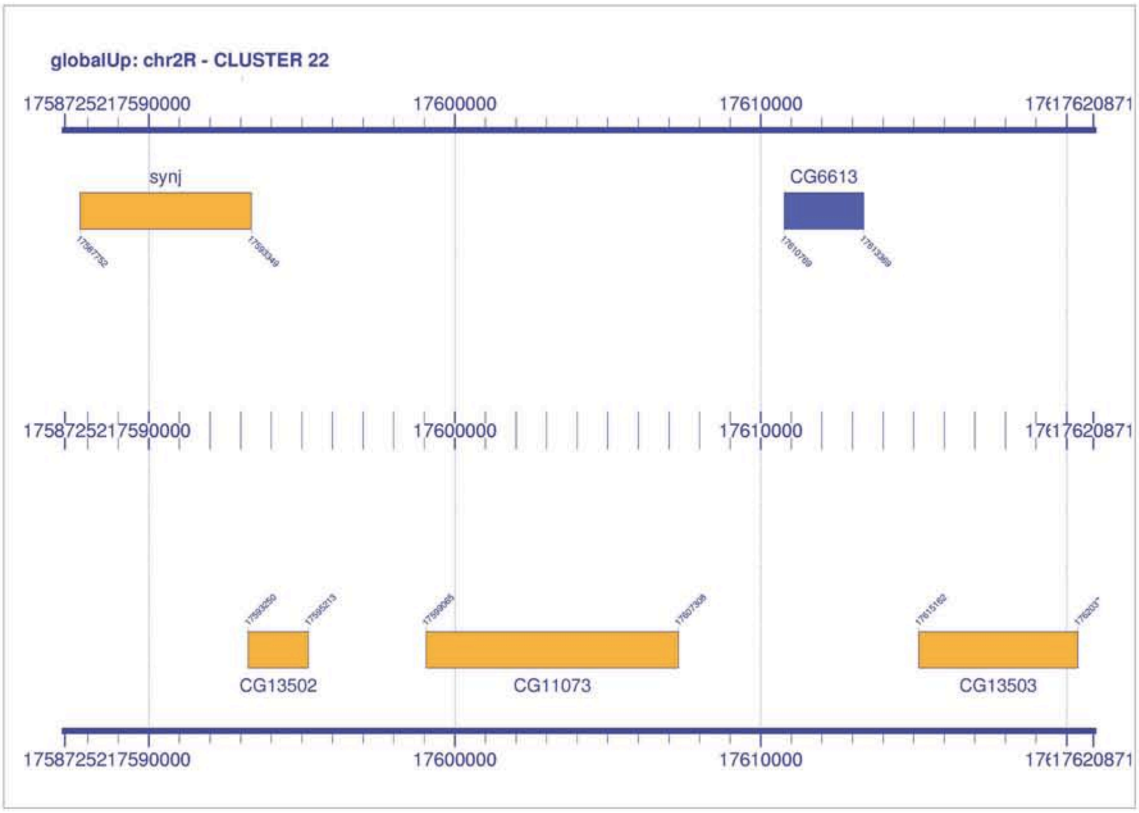

globalUp – cluster 22

Genomic components:

NAME	RefSeq	Function	
SYNJ	NM_130462	GO:0004445	inositol-polyphosphate 5-phosphatase activity
		GO:0006886	intracellular protein transport
		GO:0007269	neurotransmitter secretion
		GO:0012506	vesicle membrane
		GO:0016311	dephosphorylation
		GO:0019722	calcium-mediated signaling
		GO:0043195	terminal button
		GO:0046030	inositol trisphosphate phosphatase activity
		GO:0048488	synaptic vesicle endocytosis
CG13502	NM_137793	GO:0005488	binding
CG11073	NM_137794		
CG6613	NM_137795	GO:0005515	protein binding
		GO:0007242	intracellular signaling cascade
		GO:0008270	zinc ion binding
		GO:0019992	diacylglycerol binding
CG13503	NM_137796	GO:0007015	actin filament organization
		GO:0008360	regulation of cell shape

GO density (5 genes):

RANKING	GO id	Function	Frequency
1	GO:0043195	terminal button	20 %
2	GO:0019992	diacylglycerol binding	20 %
3	GO:0004445	inositol-polyphosphate 5-phosphatase activity	20 %
4	GO:0048488	synaptic vesicle endocytosis	20 %
5	GO:0016311	dephosphorylation	20 %
6	GO:0008270	zinc ion binding	20 %
7	GO:0012506	vesicle membrane	20 %
8	GO:0006886	intracellular protein transport	20 %
9	GO:0046030	inositol trisphosphate phosphatase activity	20 %
10	GO:0005488	binding	20 %
11	GO:0008360	regulation of cell shape	20 %
12	GO:0019722	calcium-mediated signaling	20 %
13	GO:0007242	intracellular signaling cascade	20 %
14	GO:0005515	protein binding	20 %
15	GO:0007269	neurotransmitter secretion	20 %
16	GO:0007015	actin filament organization	20 %

globalUp – chr3R: 6982821 - 6998386

Genomic components: 3 coregulated genes, 6 genes

CHR	Strand	Start	End	RefSeq	Name	Exons	Description
CHR3R	+	6982821	6985466	NM_144369	Ugt86Da	3	Ugt86Da CG18578-PA
CHR3R	+	6986923	6988866	NM_141786	CG4757	4	CG4757-PA
CHR3R	-	6990144	6991796	NM_144365	Ugt86Dg	2	Ugt86Dg CG17200-PA
CHR3R	-	6992021	6993699	NM_144366	Ugt86De	2	Ugt86De CG6653-PA
CHR3R	-	6994218	6995955	NM_079589	Ugt35b	2	UDP-glycosyltransferase 35b CG6649-PA
CHR3R	-	6996520	6998386	NM_079590	Ugt35a	2	UDP-glycosyltransferase 35a CG6644-PA

Cluster size: 15566 nucleotides

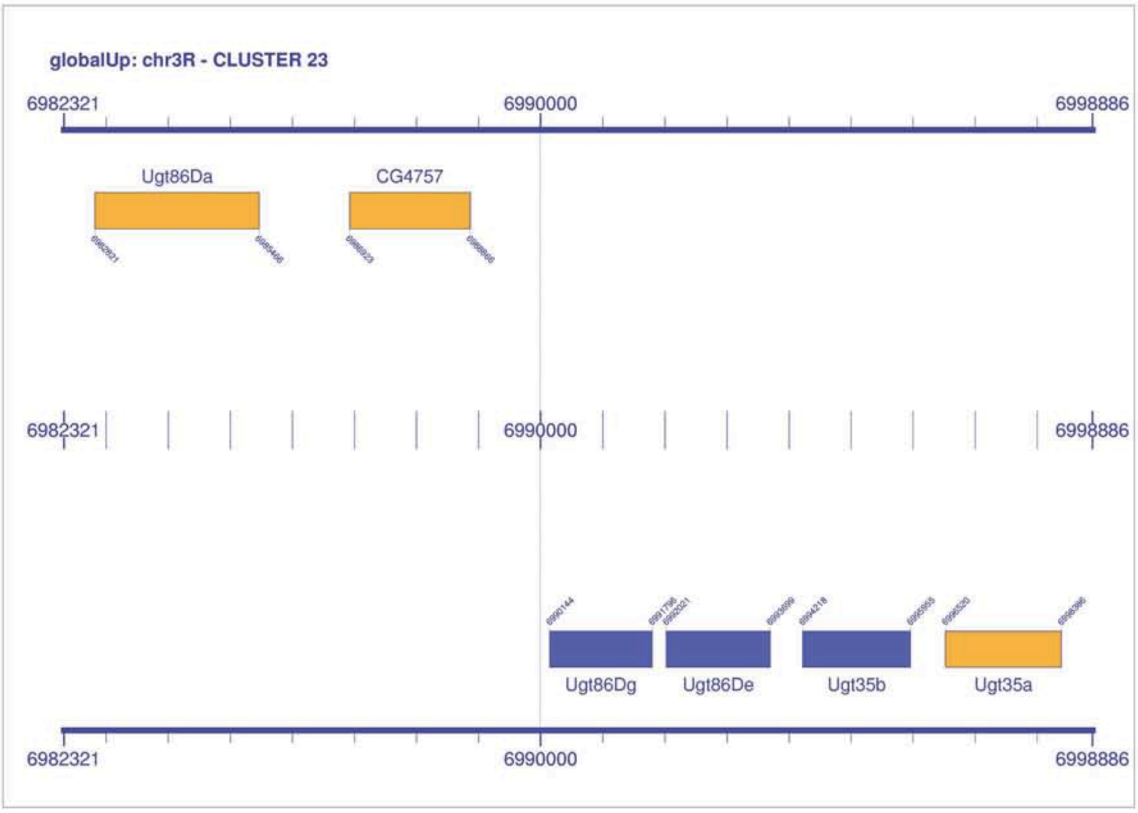

globalUp – cluster 23

Genomic components:

NAME	RefSeq	Function	
UGT86DA	NM_144369	GO:0005976	polysaccharide metabolic process
		GO:0006952	defense response
		GO:0008202	steroid metabolic process
		GO:0009636	response to toxin
		GO:0015020	glucuronosyltransferase activity
CG4757	NM_141786	GO:0004091	carboxylesterase activity
UGT86DG	NM_144365	GO:0005976	polysaccharide metabolic process
		GO:0006952	defense response
		GO:0008202	steroid metabolic process
		GO:0009636	response to toxin
		GO:0015020	glucuronosyltransferase activity
UGT86DE	NM_144366	GO:0005976	polysaccharide metabolic process
		GO:0006952	defense response
		GO:0008202	steroid metabolic process
		GO:0009636	response to toxin
		GO:0015020	glucuronosyltransferase activity
UGT35B	NM_079589	GO:0005976	polysaccharide metabolic process
		GO:0006952	defense response
		GO:0008202	steroid metabolic process
		GO:0009636	response to toxin
		GO:0015020	glucuronosyltransferase activity
UGT35A	NM_079590	GO:0005976	polysaccharide metabolic process
		GO:0006952	defense response
		GO:0008194	UDP-glycosyltransferase activity
		GO:0008202	steroid metabolic process
		GO:0009636	response to toxin
		GO:0015020	glucuronosyltransferase activity

GO density (6 genes):

RANKING	GO id	Function	Frequency
1	GO:0006952	defense response	83 %
2	GO:0008202	steroid metabolic process	83 %
3	GO:0009636	response to toxin	83 %
4	GO:0005976	polysaccharide metabolic process	83 %
5	GO:0015020	glucuronosyltransferase activity	83 %
6	GO:0004091	carboxylesterase activity	16 %
7	GO:0008194	UDP-glycosyltransferase activity	16 %

globalUp – chr3R: 8185732 - 8199535

Genomic components: 5 coregulated genes, 7 genes

CHR	Strand	Start	End	RefSeq	Name	Exons	Description
CHR3R	+	8185732	8188975	NM_141922	CG4115	3	CG4115-PA
CHR3R	-	8189388	8190402	NM_141923	Tim17a1	1	Tim17a1 CG10090-PA
CHR3R	-	8190635	8191267	NM_144456	GstD10	1	Glutathione S transferase D10 CG18548-PA
CHR3R	-	8191901	8193286	NM_141924	GstD9	2	Glutathione S transferase D9 CG10091-PA
CHR3R	-	8193268	8194417	NM_001038953	GstD1	1	Glutathione S transferase D1 CG10045-PB, isoform
CHR3R	+	8197719	8198366	NM_080173	GstD2	1	Glutathione S transferase D2 CG4181-PA
CHR3R	+	8198785	8199535	NM_176479	GstD3	1	Glutathione S transferase D3 CG4381-PA

Cluster size: 13804 nucleotides

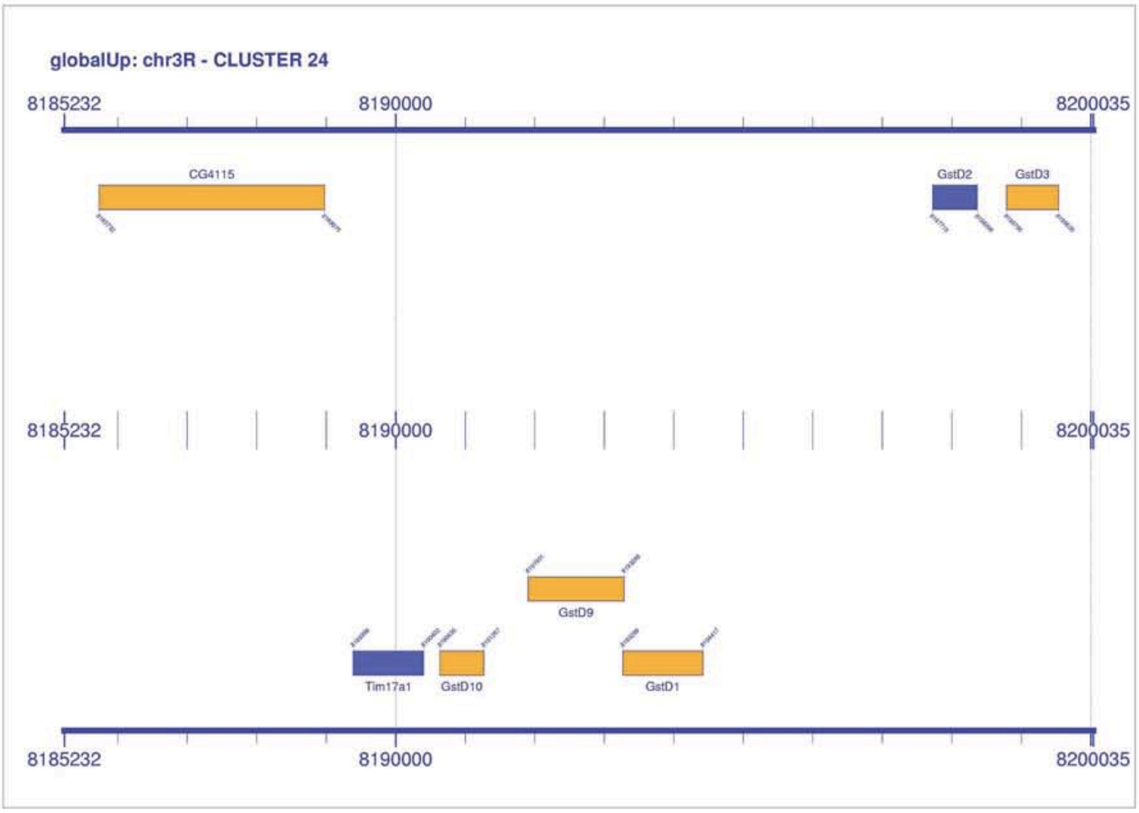

globalUp – cluster 24

Genomic components:

NAME	RefSeq	Function	
CG4115	NM_141922	GO:0005529	sugar binding
TIM17A1	NM_141923	GO:0005744	mitochondrial inner membrane presequence translocase complex
		GO:0006626	protein targeting to mitochondrion
		GO:0008565	protein transporter activity
		GO:0015450	protein translocase activity
		GO:0045039	protein import into mitochondrial inner membrane
GSTD10	NM_144456	GO:0004364	glutathione transferase activity
		GO:0006952	defense response
		GO:0009636	response to toxin
GSTD9	NM_141924	GO:0004364	glutathione transferase activity
		GO:0006952	defense response
		GO:0009636	response to toxin
GSTD1	NM_001038953	GO:0004364	glutathione transferase activity
		GO:0006952	defense response
		GO:0009636	response to toxin
GSTD2	NM_080173	GO:0004364	glutathione transferase activity
		GO:0004602	glutathione peroxidase activity
		GO:0006952	defense response
		GO:0009636	response to toxin
GSTD3	NM_176479	GO:0004364	glutathione transferase activity
		GO:0005575	cellular_component
		GO:0006952	defense response
		GO:0009636	response to toxin

GO density (7 genes):

RANKING	GO id	Function	Frequency
1	GO:0006952	defense response	71 %
2	GO:0009636	response to toxin	71 %
3	GO:0004364	glutathione transferase activity	71 %
4	GO:0015450	protein translocase activity	14 %
5	GO:0005529	sugar binding	14 %
6	GO:0005575	cellular_component	14 %
7	GO:0008565	protein transporter activity	14 %
8	GO:0006626	protein targeting to mitochondrion	14 %
9	GO:0045039	protein import into mitochondrial inner membrane	14 %
10	GO:0005744	mitochondrial inner membrane presequence translocase complex	14 %
11	GO:0004602	glutathione peroxidase activity	14 %

globalUp – chr3R: 8449482 - 8468295

Genomic components: 3 coregulated genes, 8 genes

Chr	Strand	Start	End	RefSeq	Name	Exons	Description
CHR3R	-	8449482	8453551	NM_057560	Vha55	4	Vacuolar H[+]-ATPase 55kD B subunit CG17369-PB,
CHR3R	+	8453762	8455783	NM_141957	CG6359	3	CG6359-PA, isoform A
CHR3R	+	8456388	8458754	NM_141958	CG18616	6	CG18616-PA
CHR3R	+	8459080	8460372	NM_144457	CG18530	3	CG18530-PA
CHR3R	+	8460638	8462005	NM_001043245	CG11598	3	CG11598-PB
CHR3R	+	8462289	8463602	NM_141960	CG11600	3	CG11600-PA
CHR3R	+	8464202	8465667	NM_141961	CG11608	3	CG11608-PA
CHR3R	-	8466063	8468295	NM_141962	CG6753	3	CG6753-PA

Cluster size: 18814 nucleotides

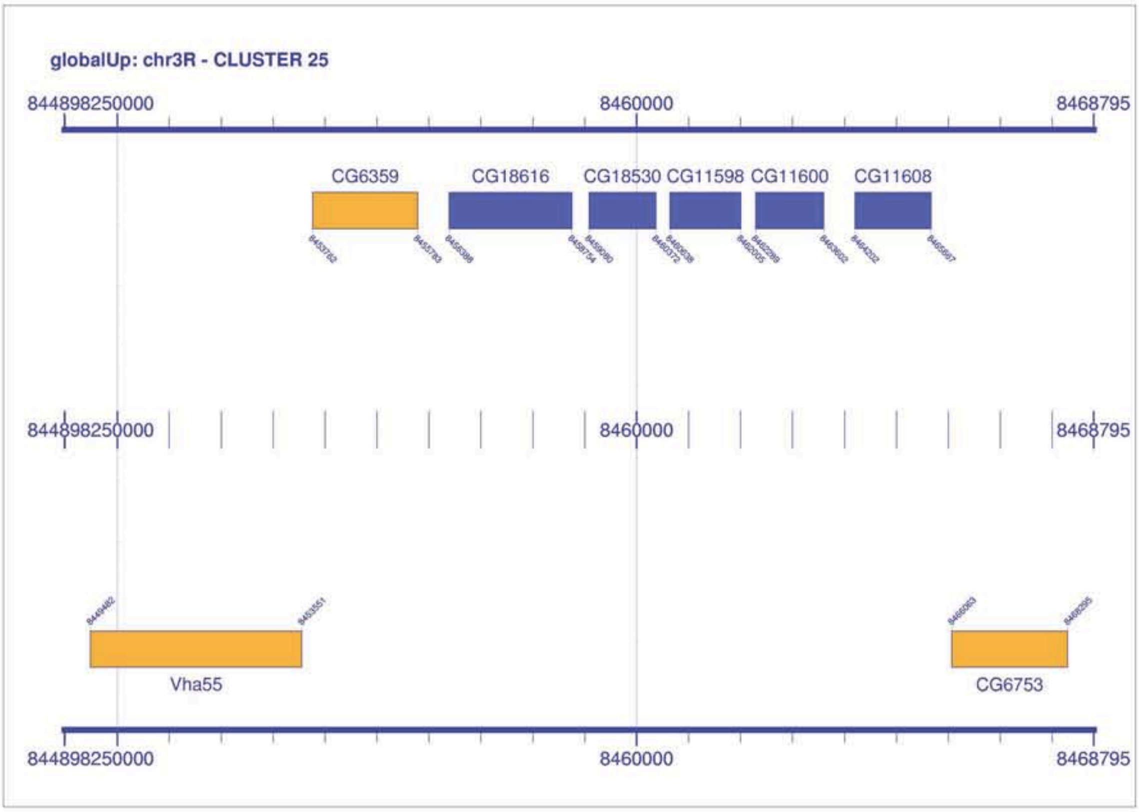

globalUp – cluster 25

Genomic components:

Name	RefSeq	Function	
Vha55	NM_057560	GO:0000221	hydrogen ion transporting ATPase V1 domain
		GO:0005524	ATP binding
		GO:0006144	purine base metabolic process
		GO:0006928	cell motility
		GO:0007010	cytoskeleton organization and biogenesis
		GO:0008553	hydrogen-exporting ATPase activity, phosphorylative mechanism
		GO:0015986	ATP synthesis coupled proton transport
		GO:0015988	energy coupled proton transport, against electrochemical gradient
		GO:0015992	proton transport
		GO:0016469	proton-transporting two-sector ATPase complex
		GO:0046933	hydrogen ion transporting ATP synthase activity, rotational mechanism
		GO:0046961	hydrogen ion transporting ATPase activity, rotational mechanism
CG6359	NM_141957	GO:0005515	protein binding
		GO:0006605	protein targeting
		GO:0006886	intracellular protein transport
		GO:0007242	intracellular signaling cascade
		GO:0035091	phosphoinositide binding
CG18616	NM_141958		
CG18530	NM_144457	GO:0004806	triacylglycerol lipase activity
		GO:0006629	lipid metabolic process
CG11598	NM_001043245	GO:0004806	triacylglycerol lipase activity
		GO:0006629	lipid metabolic process
		GO:0016298	lipase activity
CG11600	NM_141960	GO:0004806	triacylglycerol lipase activity
		GO:0006629	lipid metabolic process
CG11608	NM_141961	GO:0004806	triacylglycerol lipase activity
		GO:0006629	lipid metabolic process
CG6753	NM_141962	GO:0004806	triacylglycerol lipase activity
		GO:0006629	lipid metabolic process

GO density (8 genes):

RANKING	GO id	Function	Frequency
1	GO:0006629	lipid metabolic process	62 %
2	GO:0004806	triacylglycerol lipase activity	62 %
3	GO:0006928	cell motility	12 %
4	GO:0015986	ATP synthesis coupled proton transport	12 %
5	GO:0000221	hydrogen ion transporting ATPase V1 domain	12 %
6	GO:0016469	proton-transporting two-sector ATPase complex	12 %
7	GO:0046961	hydrogen ion transporting ATPase activity, rotational mechanism	12 %
8	GO:0015992	proton transport	12 %
9	GO:0006144	purine base metabolic process	12 %
10	GO:0016298	lipase activity	12 %
11	GO:0005524	ATP binding	12 %
12	GO:0008553	hydrogen-exporting ATPase activity, phosphorylative mechanism	12 %
13	GO:0007010	cytoskeleton organization and biogenesis	12 %
14	GO:0006886	intracellular protein transport	12 %
15	GO:0035091	phosphoinositide binding	12 %
16	GO:0006605	protein targeting	12 %
17	GO:0046933	hydrogen ion transporting ATP synthase activity, rotational mechanism	12 %
18	GO:0007242	intracellular signaling cascade	12 %
19	GO:0005515	protein binding	12 %
20	GO:0015988	energy coupled proton transport, against electrochemical gradient	12 %

globalUp – chr3R: 10392301 - 10395363

Genomic components: 3 coregulated genes, 3 genes

CHR	Strand	Start	End	RefSeq	Name	Exons	Description
CHR3R	-	10392301	10393148	NM_169586	CG31313	3	CG31313-PA
CHR3R	-	10393331	10394532	NM_142117	CG8066	2	CG8066-PA
CHR3R	-	10394757	10395363	NM_057508	Cys	2	Cystatin-like CG8050-PA

Cluster size: 3063 nucleotides

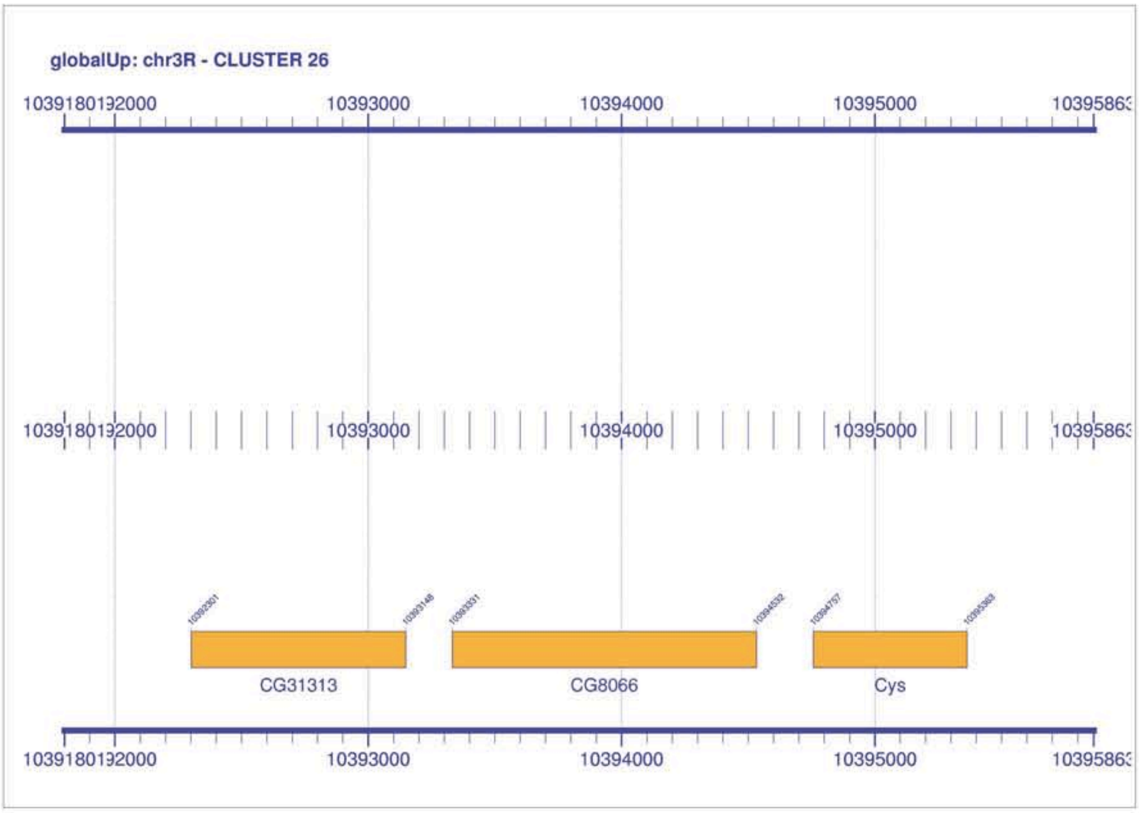

globalUp – cluster 26

Genomic components:

Name	RefSeq	Function	
CG31313	NM_169586	GO:0004869	cysteine protease inhibitor activity
CG8066	NM_142117	GO:0004869	cysteine protease inhibitor activity
Cys	NM_057508	GO:0004869	cysteine protease inhibitor activity

GO density (3 genes):

Ranking	GO id	Function	Frequency
1	GO:0004869	cysteine protease inhibitor activity	100 %

globalUp – chr3R: 11651470 - 11670137

Genomic components: 3 coregulated genes, 6 genes

CHR	Strand	Start	End	RefSeq	Name	Exons	Description
CHR3R	+	11651470	11656043	NM_142238	CG4576	8	CG4576-PA
CHR3R	-	11658491	11660147	NM_142240	CG14876	3	CG14876-PA
CHR3R	+	11660487	11661270	NM_142241	Arpc3A	3	Arpc3A CG4560-PB, isoform B
CHR3R	-	11661768	11662819	NM_079980	ND23	3	NADH:ubiquinone reductase 23kD subunit precursor
CHR3R	+	11663158	11670137	NM_057393	spn-E	11	spindle E CG3158-PA
CHR3R	-	11663867	11665193	NM_142242	CG9597	1	CG9597-PA

Cluster size: 18668 nucleotides

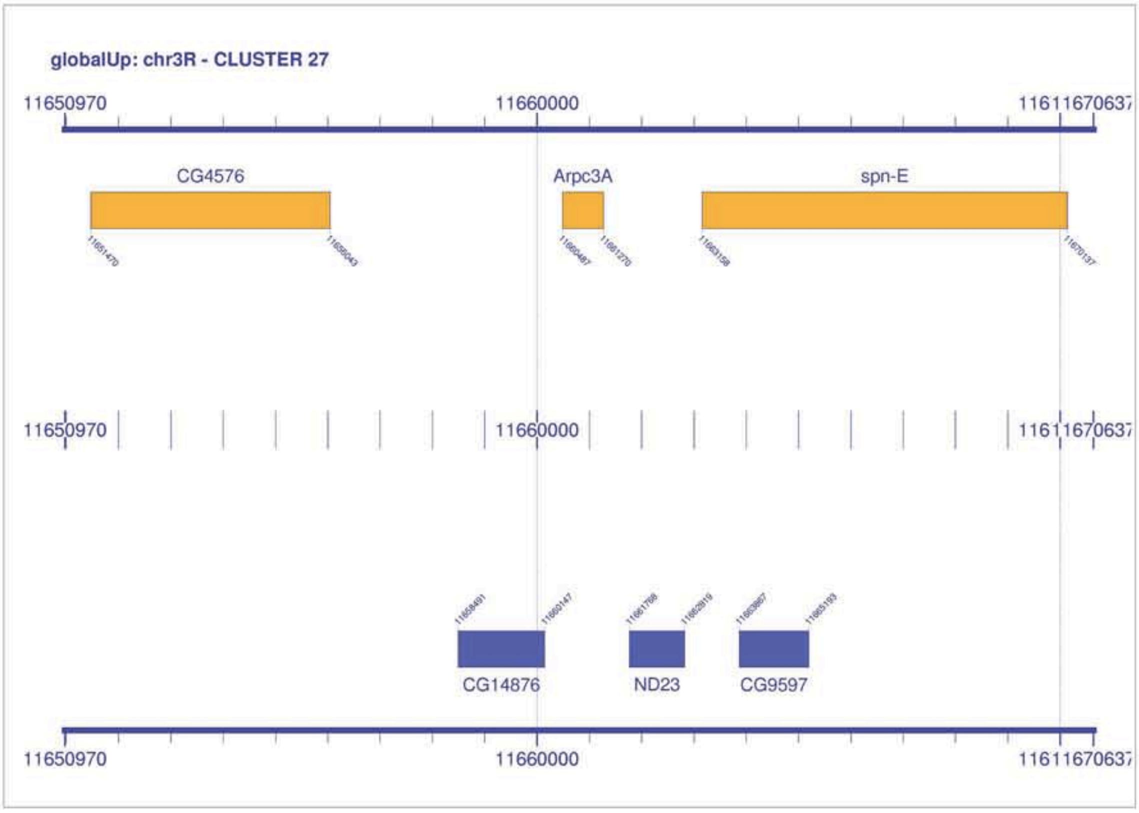

globalUp – cluster 27

Genomic components:

Name	RefSeq	Function	
CG4576	NM_142238		
CG14876	NM_142240		
Arpc3A	NM_142241	GO:0003779	actin binding
		GO:0005200	structural constituent of cytoskeleton
		GO:0005856	cytoskeleton
		GO:0007010	cytoskeleton organization and biogenesis
		GO:0030833	regulation of actin filament polymerization
ND23	NM_079980	GO:0003954	NADH dehydrogenase activity
		GO:0005506	iron ion binding
		GO:0005747	mitochondrial respiratory chain complex I
		GO:0006120	mitochondrial electron transport, NADH to ubiquinone
		GO:0008137	NADH dehydrogenase (ubiquinone) activity
spn-E	NM_057393	GO:0000398	nuclear mRNA splicing, via spliceosome
		GO:0001556	oocyte maturation
		GO:0003676	nucleic acid binding
		GO:0003724	RNA helicase activity
		GO:0004004	ATP-dependent RNA helicase activity
		GO:0004386	helicase activity
		GO:0005524	ATP binding
		GO:0005737	cytoplasm
		GO:0006342	chromatin silencing
		GO:0006403	RNA localization
		GO:0007294	oocyte fate determination (sensu Insecta)
		GO:0007315	pole plasm assembly
		GO:0007317	regulation of pole plasm oskar mRNA localization
		GO:0008186	RNA-dependent ATPase activity
		GO:0008270	zinc ion binding
		GO:0008298	intracellular mRNA localization
		GO:0009949	polarity specification of anterior/posterior axis
		GO:0009951	polarity specification of dorsal/ventral axis
		GO:0009993	oogenesis (sensu Insecta)
		GO:0009994	oocyte differentiation
		GO:0030423	RNA interference, targeting of mRNA for destruction
		GO:0030717	karyosome formation
		GO:0030720	oocyte localization during oogenesis
		GO:0045451	pole plasm oskar mRNA localization
CG9597	NM_142242		

GO density (6 genes):

RANKING	GO id	Function	Frequency
1	GO:0007315	pole plasm assembly	16 %
2	GO:0030717	karyosome formation	16 %
3	GO:0003779	actin binding	16 %
4	GO:0003676	nucleic acid binding	16 %
5	GO:0007317	regulation of pole plasm oskar mRNA localization	16 %
6	GO:0005200	structural constituent of cytoskeleton	16 %
7	GO:0003724	RNA helicase activity	16 %
8	GO:0008298	intracellular mRNA localization	16 %
9	GO:0007294	oocyte fate determination (sensu Insecta)	16 %
10	GO:0009951	polarity specification of dorsal/ventral axis	16 %
11	GO:0008270	zinc ion binding	16 %
12	GO:0006120	mitochondrial electron transport, NADH to ubiquinone	16 %
13	GO:0008186	RNA-dependent ATPase activity	16 %
14	GO:0006403	RNA localization	16 %
15	GO:0009993	oogenesis (sensu Insecta)	16 %
16	GO:0004386	helicase activity	16 %
17	GO:0005506	iron ion binding	16 %
18	GO:0009994	oocyte differentiation	16 %
19	GO:0005524	ATP binding	16 %
20	GO:0009949	polarity specification of anterior/posterior axis	16 %
21	GO:0030423	RNA interference, targeting of mRNA for destruction	16 %
22	GO:0007010	cytoskeleton organization and biogenesis	16 %
23	GO:0004004	ATP-dependent RNA helicase activity	16 %
24	GO:0001556	oocyte maturation	16 %
25	GO:0030720	oocyte localization during oogenesis	16 %
26	GO:0003954	NADH dehydrogenase activity	16 %
27	GO:0000398	nuclear mRNA splicing, via spliceosome	16 %
28	GO:0006342	chromatin silencing	16 %
29	GO:0005856	cytoskeleton	16 %
30	GO:0008137	NADH dehydrogenase (ubiquinone) activity	16 %
31	GO:0045451	pole plasm oskar mRNA localization	16 %
32	GO:0005747	mitochondrial respiratory chain complex I	16 %
33	GO:0005737	cytoplasm	16 %
34	GO:0030833	regulation of actin filament polymerization	16 %

globalUp – chr3R: 16887401 - 16923527

Genomic components: 3 coregulated genes, 6 genes

CHR	Strand	Start	End	RefSeq	Name	Exons	Description
CHR3R	-	16887401	16890185	NM_079702	Cortactin	4	Cortactin CG3637-PA
CHR3R	+	16890950	16896639	NM_057255	AnnIX	5	Annexin IX CG5730-PB, isoform B
CHR3R	-	16896274	16899039	NM_079703	r-l	3	rudimentary-like CG3593-PA
CHR3R	+	16899728	16902520	NM_079704	dmrt93B	3	doublesex-Mab related 93B CG5737-PA
CHR3R	-	16902636	16906252	NM_142681	CG7056	2	CG7056-PA
CHR3R	-	16908760	16923527	NM_142682	RhoGAP93B	14	RhoGAP93B CG3421-PA

Cluster size: 36127 nucleotides

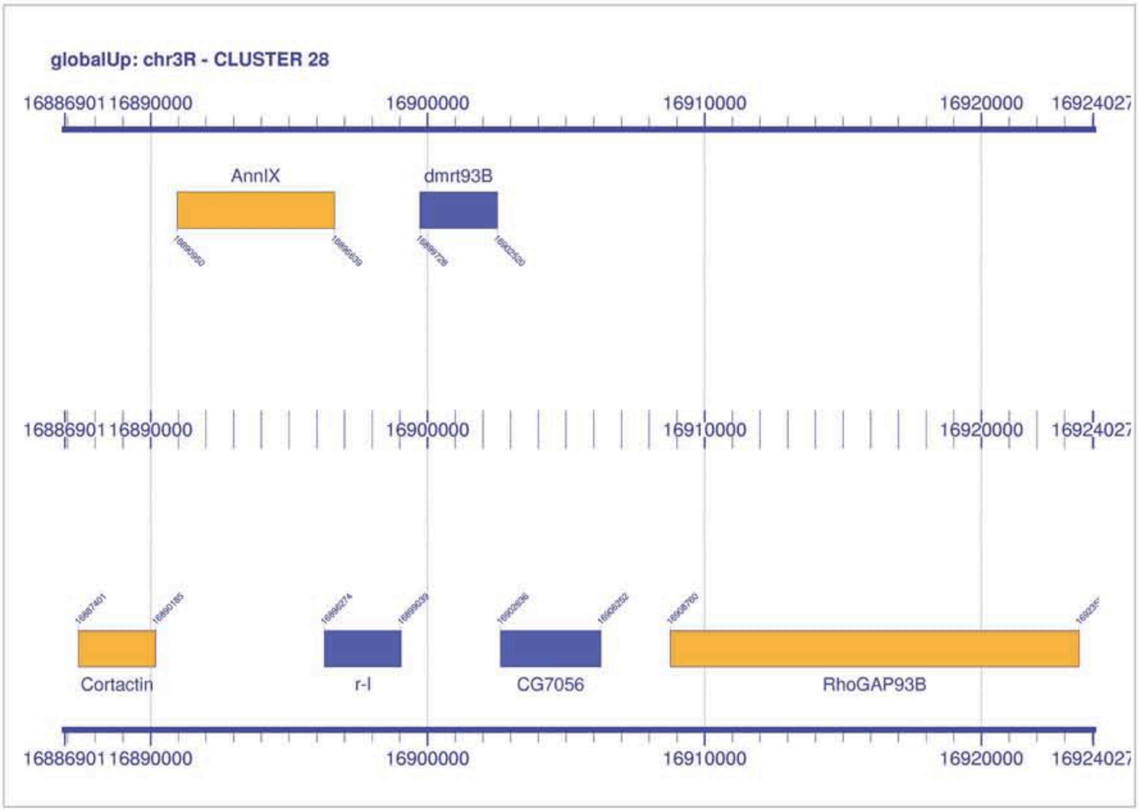

globalUp – cluster 28

Genomic components:

Name	RefSeq	Function	
Cortactin	NM_079702	GO:0003779	actin binding
		GO:0005200	structural constituent of cytoskeleton
		GO:0005856	cytoskeleton
		GO:0007010	cytoskeleton organization and biogenesis
		GO:0007298	border follicle cell migration (sensu Insecta)
		GO:0007301	ovarian ring canal formation
		GO:0008360	regulation of cell shape
AnnIX	NM_057255	GO:0003779	actin binding
		GO:0005509	calcium ion binding
		GO:0005543	phospholipid binding
		GO:0005544	calcium-dependent phospholipid binding
		GO:0006629	lipid metabolic process
R-L	NM_079703		
dmrt93B	NM_079704	GO:0001501	skeletal development
		GO:0003700	transcription factor activity
		GO:0005634	nucleus
		GO:0006355	regulation of transcription, DNA-dependent
		GO:0007498	mesoderm development
		GO:0007517	muscle development
		GO:0007548	sex differentiation
CG7056	NM_142681	GO:0003700	transcription factor activity
		GO:0005634	nucleus
		GO:0006357	regulation of transcription from RNA polymerase II promoter
		GO:0007498	mesoderm development
		GO:0030097	hemopoiesis
		GO:0043565	sequence-specific DNA binding
		GO:0045449	regulation of transcription
RhoGAP93B	NM_142682	GO:0005856	cytoskeleton
		GO:0007411	axon guidance

GO density (6 genes):

Ranking	GO id	Function	Frequency
1	GO:0003779	actin binding	33 %
2	GO:0005634	nucleus	33 %
3	GO:0007498	mesoderm development	33 %
4	GO:0005856	cytoskeleton	33 %
5	GO:0003700	transcription factor activity	33 %
6	GO:0001501	skeletal development	16 %
7	GO:0005509	calcium ion binding	16 %
8	GO:0005543	phospholipid binding	16 %
9	GO:0005200	structural constituent of cytoskeleton	16 %
10	GO:0007517	muscle development	16 %
11	GO:0030097	hemopoiesis	16 %
12	GO:0043565	sequence-specific DNA binding	16 %
13	GO:0006357	regulation of transcription from RNA polymerase II promoter	16 %
14	GO:0007301	ovarian ring canal formation	16 %
15	GO:0006355	regulation of transcription, DNA-dependent	16 %
16	GO:0005544	calcium-dependent phospholipid binding	16 %
17	GO:0006629	lipid metabolic process	16 %
18	GO:0045449	regulation of transcription	16 %
19	GO:0007010	cytoskeleton organization and biogenesis	16 %
20	GO:0007548	sex differentiation	16 %
21	GO:0008360	regulation of cell shape	16 %
22	GO:0007298	border follicle cell migration (sensu Insecta)	16 %
23	GO:0007411	axon guidance	16 %

globalUp – chr3R: 19586458 - 19627394

Genomic components: 3 coregulated genes, 8 genes

CHR	Strand	Start	End	RefSeq	Name	Exons	Description
CHR3R	-	19586458	19588951	NM_142925	CG10217	4	CG10217-PA, isoform A
CHR3R	+	19589578	19592386	NM_142926	Lsd-1	7	Lipid storage droplet-1 CG10374-PC, isoform C
CHR3R	-	19592240	19593174	NM_142927	CG10214	1	CG10214-PA
CHR3R	+	19593433	19594704	NM_142928	CG10375	3	CG10375-PA
CHR3R	-	19594120	19598529	NM_079741	tst	3	twister CG10210-PA
CHR3R	-	19598715	19599845	NM_142929	CG10208	2	CG10208-PA
CHR3R	-	19599946	19607366	NM_142930	Nup98	9	Nup98 CG10198-PA
CHR3R	+	19607507	19627394	NM_057796	mbc	14	myoblast city CG10379-PA

Cluster size: 40937 nucleotides

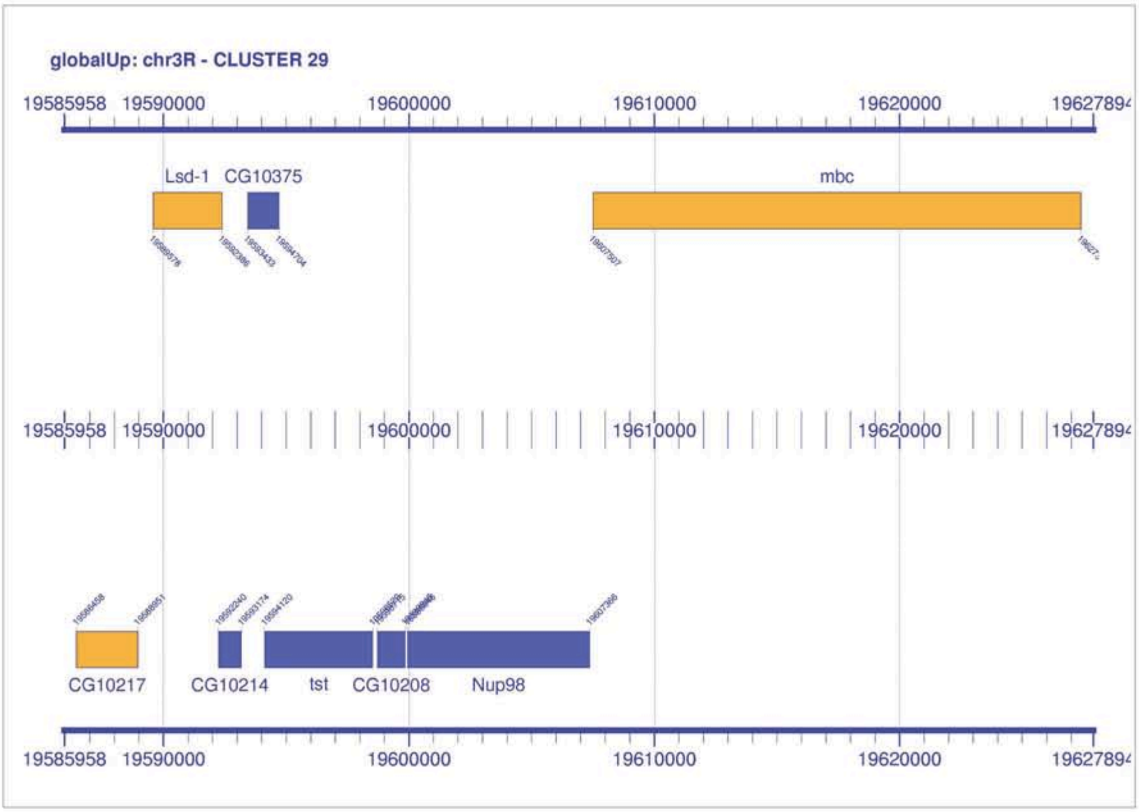

globalUp – cluster 29

Genomic components:

NAME	RefSeq	Function	
CG10217	NM_142925		
LSD-1	NM_142926	GO:0005811	lipid particle
		GO:0019915	sequestering of lipid
CG10214	NM_142927	GO:0000175	3'-5'-exoribonuclease activity
		GO:0003676	nucleic acid binding
		GO:0005634	nucleus
		GO:0005739	mitochondrion
		GO:0006401	RNA catabolic process
		GO:0008946	oligonucleotidase activity
CG10375	NM_142928	GO:0031072	heat shock protein binding
TST	NM_079741	GO:0000381	regulation of alternative nuclear mRNA splicing, via spliceosome
		GO:0003676	nucleic acid binding
		GO:0004386	helicase activity
		GO:0005524	ATP binding
		GO:0005634	nucleus
		GO:0006403	RNA localization
		GO:0006412	translation
		GO:0008026	ATP-dependent helicase activity
		GO:0019538	protein metabolic process
CG10208	NM_142929		
NUP98	NM_142930	GO:0005643	nuclear pore
		GO:0006605	protein targeting
MBC	NM_057796	GO:0000902	cell morphogenesis
		GO:0001726	ruffle
		GO:0005083	small GTPase regulator activity
		GO:0005085	guanyl-nucleotide exchange factor activity
		GO:0005515	protein binding
		GO:0005525	GTP binding
		GO:0006886	intracellular protein transport
		GO:0006909	phagocytosis
		GO:0007242	intracellular signaling cascade
		GO:0007391	dorsal closure
		GO:0007498	mesoderm development
		GO:0007519	striated muscle development
		GO:0007520	myoblast fusion
		GO:0007523	larval visceral muscle development
		GO:0051020	GTPase binding

GO density (8 genes):

Ranking	GO id	Function	Frequency
1	GO:0003676	nucleic acid binding	25 %
2	GO:0005634	nucleus	25 %
3	GO:0005083	small GTPase regulator activity	12 %
4	GO:0019915	sequestering of lipid	12 %
5	GO:0001726	ruffle	12 %
6	GO:0007520	myoblast fusion	12 %
7	GO:0007523	larval visceral muscle development	12 %
8	GO:0006401	RNA catabolic process	12 %
9	GO:0000902	cell morphogenesis	12 %
10	GO:0007498	mesoderm development	12 %
11	GO:0005739	mitochondrion	12 %
12	GO:0000175	3’-5’-exoribonuclease activity	12 %
13	GO:0006403	RNA localization	12 %
14	GO:0004386	helicase activity	12 %
15	GO:0031072	heat shock protein binding	12 %
16	GO:0005524	ATP binding	12 %
17	GO:0005643	nuclear pore	12 %
18	GO:0005525	GTP binding	12 %
19	GO:0007519	striated muscle development	12 %
20	GO:0006412	translation	12 %
21	GO:0051020	GTPase binding	12 %
22	GO:0005811	lipid particle	12 %
23	GO:0019538	protein metabolic process	12 %
24	GO:0006886	intracellular protein transport	12 %
25	GO:0008946	oligonucleotidase activity	12 %
26	GO:0006605	protein targeting	12 %
27	GO:0006909	phagocytosis	12 %
28	GO:0007242	intracellular signaling cascade	12 %
29	GO:0005515	protein binding	12 %
30	GO:0008026	ATP-dependent helicase activity	12 %
31	GO:0005085	guanyl-nucleotide exchange factor activity	12 %
32	GO:0007391	dorsal closure	12 %
33	GO:0000381	regulation of alternative nuclear mRNA splicing, via spliceosome	12 %

globalUp – chr3R: 21115598 - 21150108

Genomic components: 7 coregulated genes, 16 genes

Chr	Strand	Start	End	RefSeq	Name	Exons	Description
chr3R	+	21115598	21116961	NM_143118	CG11893	2	CG11893-PA
chr3R	-	21117217	21118693	NM_170212	CG31098	3	CG31098-PA
chr3R	-	21119179	21121312	NM_170213	CG31102	4	CG31102-PA
chr3R	-	21122059	21123508	NM_170214	CG31097	4	CG31097-PA
chr3R	-	21123715	21125222	NM_170215	CG31288	4	CG31288-PA
chr3R	+	21126632	21128010	NM_143121	CG13659	3	CG13659-PA
chr3R	+	21128282	21129586	NM_170216	CG31370	3	CG31370-PA
chr3R	+	21129920	21131309	NM_170217	CG31436	3	CG31436-PA
chr3R	-	21132128	21134109	NM_143123	CG10550	3	CG10550-PA, isoform A
chr3R	-	21135147	21136665	NM_170220	CG31099	3	CG31099-PA
chr3R	-	21136860	21138375	NM_170221	CG31087	5	CG31087-PA
chr3R	-	21140390	21141826	NM_143125	CG10559	4	CG10559-PA
chr3R	-	21142342	21143807	NM_143126	CG10553	4	CG10553-PA
chr3R	-	21144271	21145801	NM_143127	CG10560	4	CG10560-PA
chr3R	-	21146774	21148473	NM_143128	CG10562	4	CG10562-PA
chr3R	-	21149026	21150108	NM_170222	CHKov1	3	CHKov1 CG10618-PA

Cluster size: 34511 nucleotides

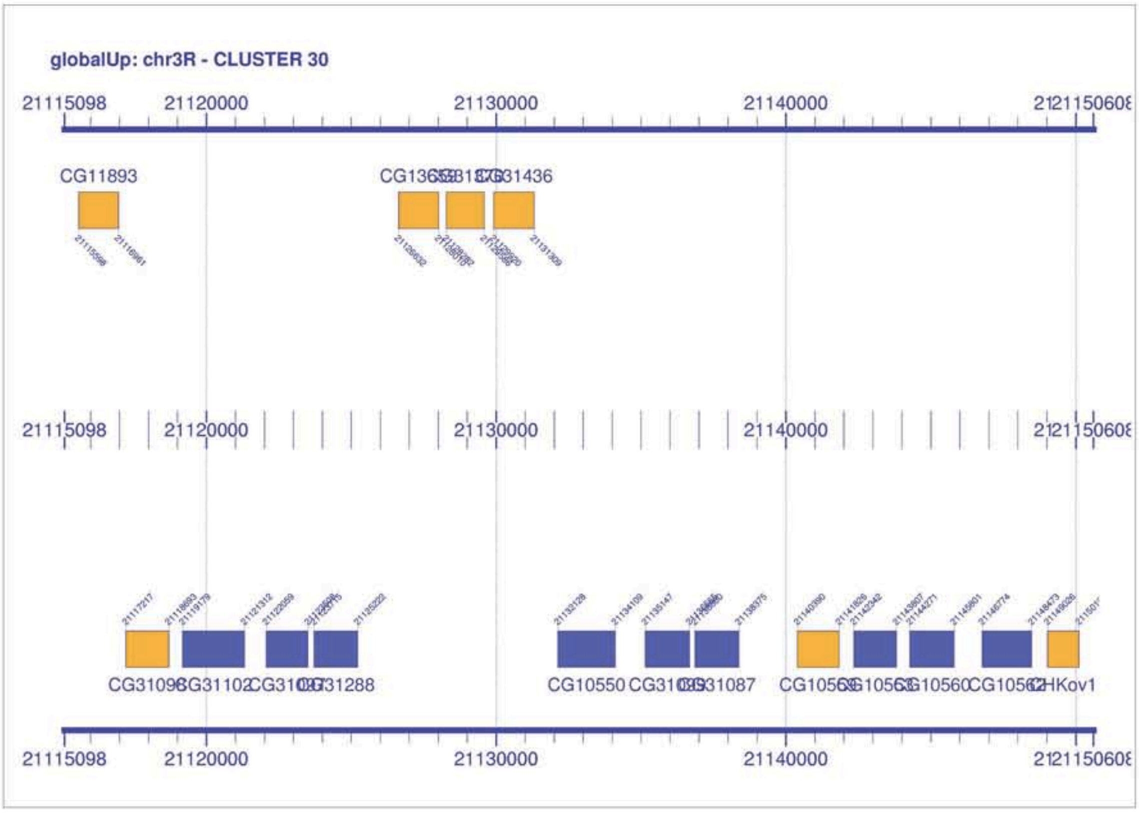

globalUp – cluster 30

Genomic components:

NAME	RefSeq	Function	
CG11893	NM_143118		
CG31098	NM_170212		
CG31102	NM_170213		
CG31097	NM_170214	GO:0004713<br>GO:0005524<br>GO:0006468	protein-tyrosine kinase activity<br>ATP binding<br>protein amino acid phosphorylation
CG31288	NM_170215		
CG13659	NM_143121		
CG31370	NM_170216	GO:0008152<br>GO:0016491	metabolic process<br>oxidoreductase activity
CG31436	NM_170217		
CG10550	NM_143123		
CG31099	NM_170220		
CG31087	NM_170221		
CG10559	NM_143125		
CG10553	NM_143126		
CG10560	NM_143127		
CG10562	NM_143128		
CHKov1	NM_170222		

GO density (16 genes):

RANKING	GO id	Function	Frequency
1	GO:0016491	oxidoreductase activity	6 %
2	GO:0005524	ATP binding	6 %
3	GO:0006468	protein amino acid phosphorylation	6 %
4	GO:0008152	metabolic process	6 %
5	GO:0004713	protein-tyrosine kinase activity	6 %

globalUp – chr3R: 25625374 - 25651522

Genomic components: 3 coregulated genes, 8 genes

Chr	Strand	Start	End	RefSeq	Name	Exons	Description
chr3R	+	25625374	25627306	NM_079827	Ice	1	Ice CG7788-PA
chr3R	-	25627447	25628675	NM_143470	CG7834	3	CG7834-PA, isoform A
chr3R	+	25628918	25630103	NM_143471	CG7789	2	CG7789-PA
chr3R	-	25630008	25632849	NM_057303	ncd	3	non-claret disjunctional CG7831-PA
chr3R	+	25632969	25640780	NM_170430	ca	15	claret CG31037-PA
chr3R	-	25640642	25642172	NM_143474	Vps16B	1	Vps16B CG18112-PA
chr3R	-	25642453	25643486	NM_143475	CG7829	2	CG7829-PA, isoform A
chr3R	+	25644833	25651522	NM_143476	CG7802	8	CG7802-PA, isoform A

Cluster size: 26149 nucleotides

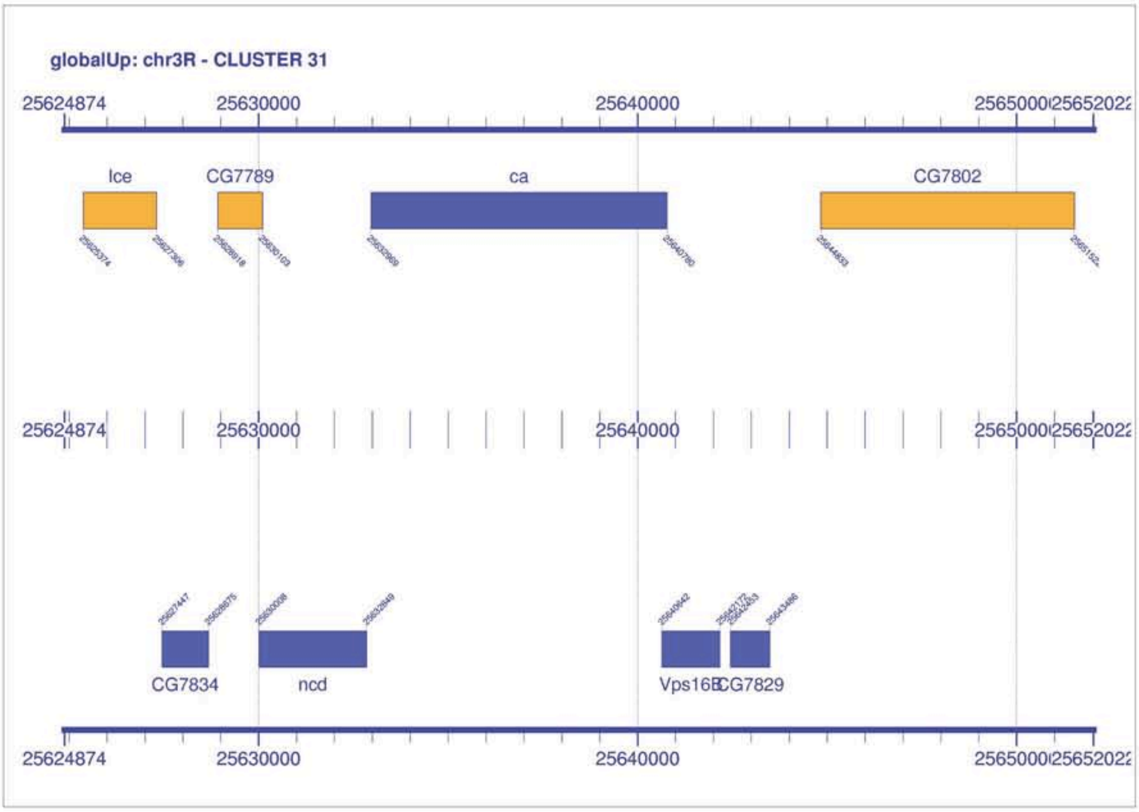

globalUp – cluster 31

Genomic components:

Name	RefSeq	Function	
Ice	NM_079827	GO:0006508	proteolysis
		GO:0006915	apoptosis
		GO:0008303	caspase complex
		GO:0030693	caspase activity
		GO:0046668	regulation of retinal programmed cell death
CG7834	NM_143470	GO:0006118	electron transport
		GO:0006119	oxidative phosphorylation
		GO:0009055	electron carrier activity
		GO:0016491	oxidoreductase activity
		GO:0017133	mitochondrial electron transfer flavoprotein complex
CG7789	NM_143471	GO:0004437	inositol or phosphatidylinositol phosphatase activity
		GO:0006644	phospholipid metabolic process
		GO:0008441	3'(2'),5'-bisphosphate nucleotidase activity
NCD	NM_057303	GO:0000022	mitotic spindle elongation
		GO:0000212	meiotic spindle organization and biogenesis
		GO:0003777	microtubule motor activity
		GO:0005200	structural constituent of cytoskeleton
		GO:0005524	ATP binding
		GO:0005819	spindle
		GO:0005871	kinesin complex
		GO:0005872	minus-end kinesin complex
		GO:0006605	protein targeting
		GO:0007018	microtubule-based movement
		GO:0007056	female meiotic spindle assembly (sensu Metazoa)
		GO:0007059	chromosome segregation
		GO:0007067	mitosis
		GO:0007100	mitotic centrosome separation
		GO:0008569	minus-end-directed microtubule motor activity
CA	NM_170430		
Vps16B	NM_143474	GO:0003674	molecular_function
		GO:0005575	cellular_component
		GO:0008150	biological_process
CG7829	NM_143475	GO:0004295	trypsin activity
		GO:0006508	proteolysis
CG7802	NM_143476		

GO density (8 genes):

Ranking	GO id	Function	Frequency
1	GO:0006508	proteolysis	25 %
2	GO:0004295	trypsin activity	12 %
3	GO:0006644	phospholipid metabolic process	12 %
4	GO:0000022	mitotic spindle elongation	12 %
5	GO:0007056	female meiotic spindle assembly (sensu Metazoa)	12 %
6	GO:0005200	structural constituent of cytoskeleton	12 %
7	GO:0007018	microtubule-based movement	12 %
8	GO:0005575	cellular_component	12 %
9	GO:0016491	oxidoreductase activity	12 %
10	GO:0005871	kinesin complex	12 %
11	GO:0005819	spindle	12 %
12	GO:0008441	3'(2'),5'-bisphosphate nucleotidase activity	12 %
13	GO:0006119	oxidative phosphorylation	12 %
14	GO:0003674	molecular_function	12 %
15	GO:0000212	meiotic spindle organization and biogenesis	12 %
16	GO:0007100	mitotic centrosome separation	12 %
17	GO:0005524	ATP binding	12 %
18	GO:0007059	chromosome segregation	12 %
19	GO:0008150	biological_process	12 %
20	GO:0003777	microtubule motor activity	12 %
21	GO:0005872	minus-end kinesin complex	12 %
22	GO:0046668	regulation of retinal programmed cell death	12 %
23	GO:0017133	mitochondrial electron transfer flavoprotein complex	12 %
24	GO:0006915	apoptosis	12 %
25	GO:0009055	electron carrier activity	12 %
26	GO:0006605	protein targeting	12 %
27	GO:0030693	caspase activity	12 %
28	GO:0004437	inositol or phosphatidylinositol phosphatase activity	12 %
29	GO:0007067	mitosis	12 %
30	GO:0006118	electron transport	12 %
31	GO:0008569	minus-end-directed microtubule motor activity	12 %
32	GO:0008303	caspase complex	12 %

globalUp – chrX: 10935598 - 10957526

Genomic components: 3 coregulated genes, 5 genes

CHR	Strand	Start	End	RefSeq	Name	Exons	Description
CHRX	+	10935598	10937312	NM_132453	CG2076	6	CG2076-PA
CHRX	+	10937710	10939880	NM_132454	CG2061	3	CG2061-PA, isoform A
CHRX	+	10948693	10952232	NM_132455	CG1961	8	CG1961-PA
CHRX	+	10952428	10953140	NM_132456	CG11126	3	CG11126-PA
CHRX	+	10953690	10957526	NM_078560	Hsp60	3	Heat shock protein 60 CG12101-PA, isoform A

Cluster size: 21929 nucleotides

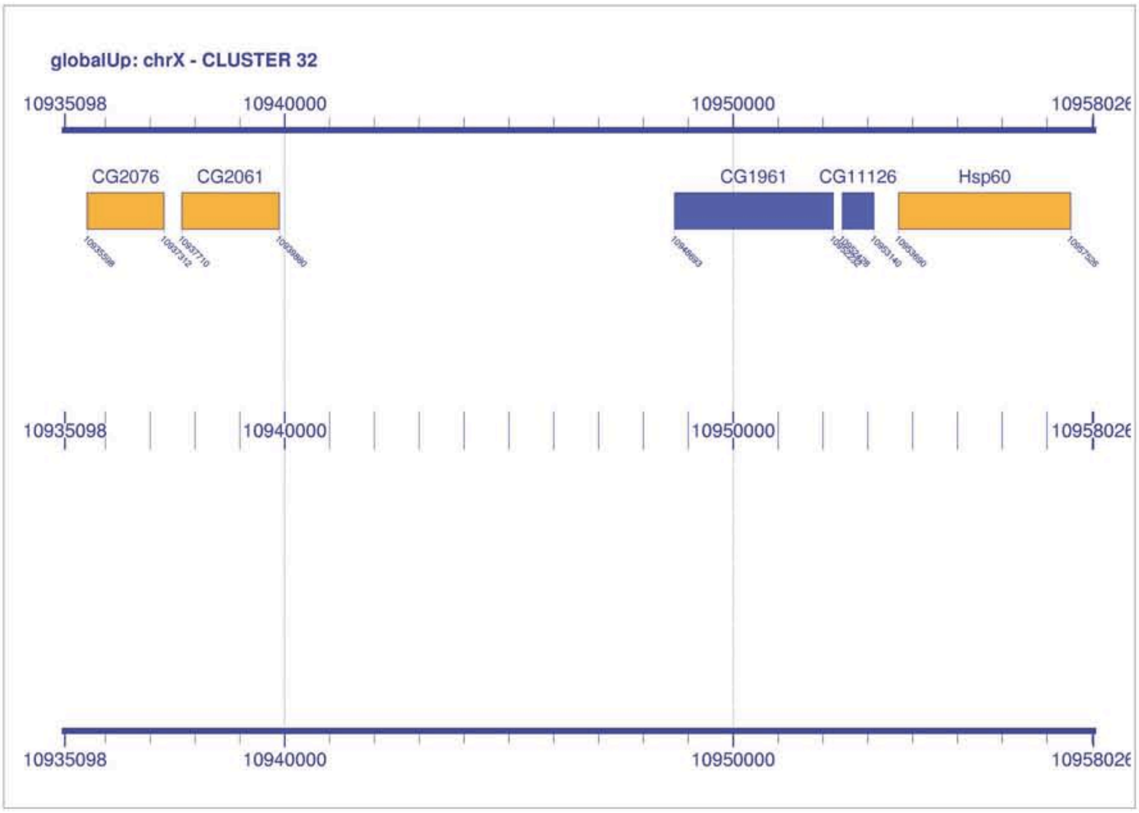

globalUp – cluster 32

Genomic components:

Name	RefSeq	Function	
CG2076	NM_132453		
CG2061	NM_132454	GO:0004930	G-protein coupled receptor activity
		GO:0007186	G-protein coupled receptor protein signaling pathway
		GO:0016021	integral to membrane
CG1961	NM_132455	GO:0006139	nucleobase, nucleoside, nucleotide and nucleic acid metabolic process
		GO:0008081	phosphoric diester hydrolase activity
		GO:0008253	5'-nucleotidase activity
		GO:0009166	nucleotide catabolic process
		GO:0019204	nucleotide phosphatase activity
CG11126	NM_132456	GO:0006139	nucleobase, nucleoside, nucleotide and nucleic acid metabolic process
		GO:0008253	5'-nucleotidase activity
		GO:0009166	nucleotide catabolic process
		GO:0019204	nucleotide phosphatase activity
Hsp60	NM_078560	GO:0005524	ATP binding
		GO:0005739	mitochondrion
		GO:0005759	mitochondrial matrix
		GO:0006457	protein folding
		GO:0006458	'de novo' protein folding
		GO:0006626	protein targeting to mitochondrion
		GO:0006950	response to stress
		GO:0009408	response to heat
		GO:0042026	protein refolding
		GO:0042623	ATPase activity, coupled
		GO:0051082	unfolded protein binding

GO density (5 genes):

Ranking	GO id	Function	Frequency
1	GO:0008253	5'-nucleotidase activity	40 %
2	GO:0009166	nucleotide catabolic process	40 %
3	GO:0006139	nucleobase, nucleoside, nucleotide and nucleic acid metabolic process	40 %
4	GO:0019204	nucleotide phosphatase activity	40 %
5	GO:0042026	protein refolding	20 %
6	GO:0051082	unfolded protein binding	20 %
7	GO:0008081	phosphoric diester hydrolase activity	20 %
8	GO:0042623	ATPase activity, coupled	20 %
9	GO:0007186	G-protein coupled receptor protein signaling pathway	20 %
10	GO:0005739	mitochondrion	20 %
11	GO:0016021	integral to membrane	20 %
12	GO:0004930	G-protein coupled receptor activity	20 %
13	GO:0006950	response to stress	20 %
14	GO:0005524	ATP binding	20 %
15	GO:0005759	mitochondrial matrix	20 %
16	GO:0006626	protein targeting to mitochondrion	20 %
17	GO:0009408	response to heat	20 %
18	GO:0006458	'de novo' protein folding	20 %
19	GO:0006457	protein folding	20 %

globalUp – chrX: 16249005 - 16275217

Genomic components: 3 coregulated genes, 4 genes

CHR	Strand	Start	End	RefSeq	Name	Exons	Description
CHRX	+	16249005	16251526	NM_132898	Traf3	4	Traf3 CG4394-PC, isoform C
CHRX	-	16251172	16256153	NM_078646	Anxb11	7	Annexin B11 CG9968-PA, isoform A
CHRX	+	16256780	16271415	NM_167520	hang	8	hangover CG32575-PB, isoform B
CHRX	-	16273084	16275217	NM_132900	CG9947	6	CG9947-PA

Cluster size: 26213 nucleotides

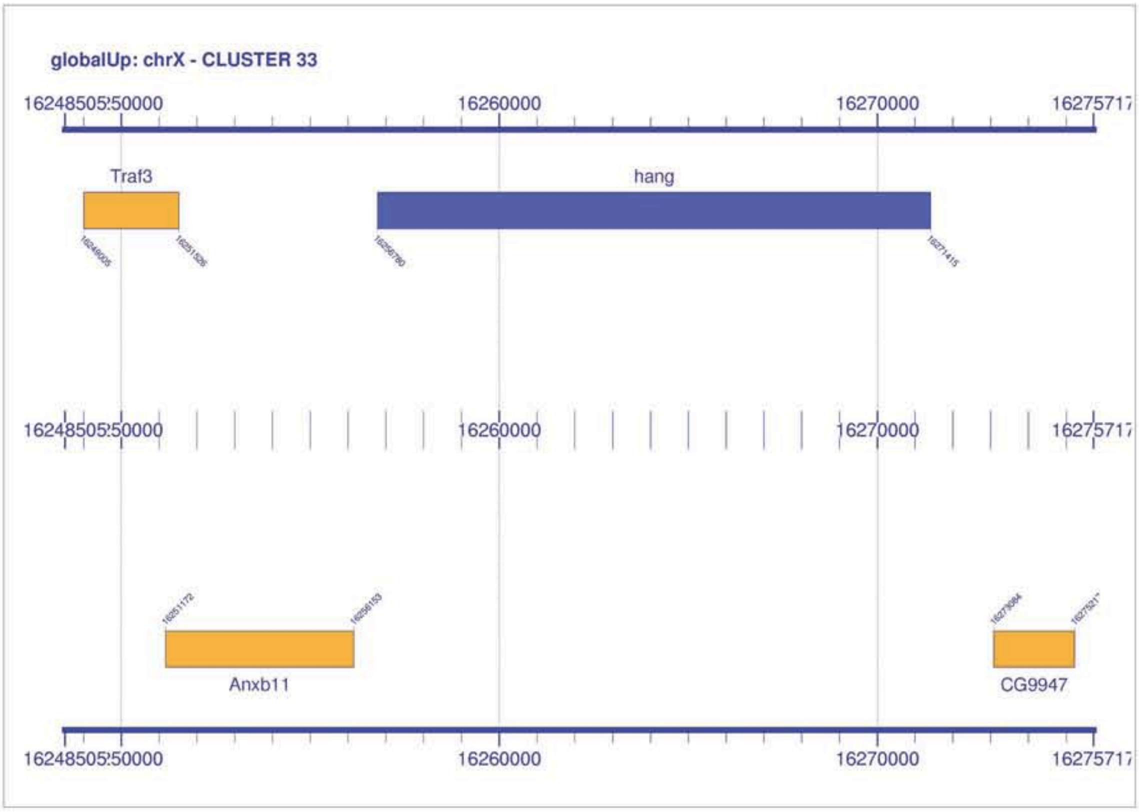

globalUp – cluster 33

Genomic components:

Name	RefSeq	Function	
Traf3	NM_132898	GO:0006952	defense response
		GO:0007165	signal transduction
Anxb11	NM_078646	GO:0003779	actin binding
		GO:0005509	calcium ion binding
		GO:0005544	calcium-dependent phospholipid binding
		GO:0006508	proteolysis
		GO:0006629	lipid metabolic process
		GO:0007155	cell adhesion
		GO:0008237	metallopeptidase activity
		GO:0008270	zinc ion binding
		GO:0008360	regulation of cell shape
hang	NM_167520	GO:0003676	nucleic acid binding
		GO:0005509	calcium ion binding
		GO:0005634	nucleus
		GO:0006950	response to stress
		GO:0008270	zinc ion binding
		GO:0009408	response to heat
		GO:0045471	response to ethanol
		GO:0048149	behavioral response to ethanol
CG9947	NM_132900	GO:0000074	regulation of progression through cell cycle
		GO:0007049	cell cycle
		GO:0016020	membrane

GO density (4 genes):

Ranking	GO id	Function	Frequency
1	GO:0005509	calcium ion binding	50 %
2	GO:0008270	zinc ion binding	50 %
3	GO:0007049	cell cycle	25 %
4	GO:0006952	defense response	25 %
5	GO:0003779	actin binding	25 %
6	GO:0003676	nucleic acid binding	25 %
7	GO:0000074	regulation of progression through cell cycle	25 %
8	GO:0005634	nucleus	25 %
9	GO:0048149	behavioral response to ethanol	25 %
10	GO:0008237	metallopeptidase activity	25 %
11	GO:0006950	response to stress	25 %
12	GO:0005544	calcium-dependent phospholipid binding	25 %
13	GO:0006629	lipid metabolic process	25 %
14	GO:0006508	proteolysis	25 %
15	GO:0007155	cell adhesion	25 %
16	GO:0008360	regulation of cell shape	25 %
17	GO:0009408	response to heat	25 %
18	GO:0007165	signal transduction	25 %
19	GO:0045471	response to ethanol	25 %
20	GO:0016020	membrane	25 %
