# Supplementary material for: Identification and Functional Analysis of Healing Regulators in Drosophila
Source: PLoS Genet. 2015 Feb 3;11(2):e1004965. doi: 10.1371/journal.pgen.1004965 (PMC4315591; doi:10.1371/journal.pgen.1004965)
Supplement: S8 Table — Chromosomal clusters of downregulated genes for the global comparison (19) are described by their chromosomal location, number of genes, number of co regulated genes, identity of each gene and each gene’s GO Terms. Genes highlighted in orange are those transcriptionally co regulated during healing. (PDF) [file pgen.1004965.s016.pdf]

globalDown – chr2L: 4442497 - 4468022

Genomic components: 3 coregulated genes, 14 genes

CHR	Strand	Start	End	RefSeq	Name	Exons	Description
CHR2L	+	4442497	4442853	NM_134988	CG15432	1	CG15432-PA
CHR2L	-	4442869	4444677	NM_134989	morgue	2	modifier of rpr and grim, ubiquitously expressed
CHR2L	+	4444731	4446769	NM_134990	CG15433	4	CG15433-PA
CHR2L	-	4446663	4448637	NM_134991	CG15438	3	CG15438-PA
CHR2L	-	4449290	4453004	NM_134992	CG15439	2	CG15439-PA
CHR2L	-	4453167	4454457	NM_134993	CG15440	2	CG15440-PA
CHR2L	+	4454664	4455160	NM_144084	CG15434	3	CG15434-PA
CHR2L	-	4455220	4456822	NM_057881	Gs1l	4	GS1-like CG15441-PB, isoform B
CHR2L	-	4457175	4458364	NM_057615	RpL27A	4	Ribosomal protein L27A CG15442-PA
CHR2L	-	4459003	4459532	NM_175957	mRpL27	3	mitochondrial ribosomal protein L27 CG33002-PA
CHR2L	+	4459834	4461746	NM_134994	CG15435	2	CG15435-PA
CHR2L	-	4461630	4463437	NM_134995	CG15443	2	CG15443-PA
CHR2L	+	4463641	4465080	NM_134996	CG15436	2	CG15436-PA
CHR2L	+	4465199	4468022	NM_134997	CG17840	3	CG17840-PA

Cluster size: 25526 nucleotides

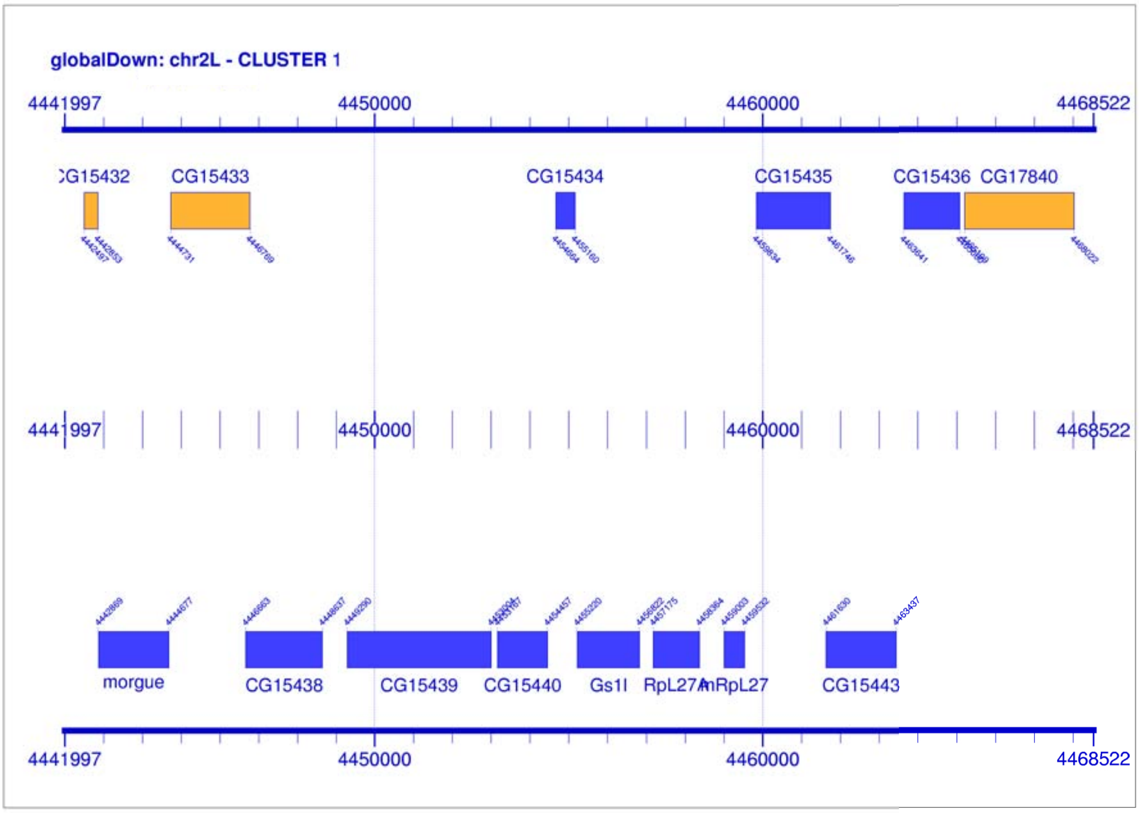

globalDown – cluster 1

Genomic components:

Name	RefSeq	Function	
CG15432	NM_134988		
MORGUE	NM_134989	GO:0004842	ubiquitin-protein ligase activity
		GO:0006915	apoptosis
		GO:0006917	induction of apoptosis
		GO:0016567	protein ubiquitination
		GO:0019005	SCF ubiquitin ligase complex
		GO:0019538	protein metabolic process
		GO:0046668	regulation of retinal programmed cell death
		GO:0046675	induction of compound eye retinal cell programmed cell death
CG15433	NM_134990	GO:0005506	iron ion binding
		GO:0006139	nucleobase, nucleoside, nucleotide and nucleic acid metaboli
		GO:0006333	chromatin assembly or disassembly
		GO:0008080	N-acetyltransferase activity
		GO:0015986	ATP synthesis coupled proton transport
		GO:0016407	acetyltransferase activity
		GO:0016469	proton-transporting two-sector ATPase complex
		GO:0046933	hydrogen ion transporting ATP synthase activity, rotational
		GO:0046961	hydrogen ion transporting ATPase activity, rotational mechan
CG15438	NM_134991	GO:0003676	nucleic acid binding
		GO:0006796	phosphate metabolic process
		GO:0006812	cation transport
		GO:0006817	phosphate transport
		GO:0006858	extracellular transport
		GO:0008270	zinc ion binding
		GO:0015321	sodium-dependent phosphate transporter activity
		GO:0016021	integral to membrane
CG15439	NM_134992	GO:0005515	protein binding
		GO:0006355	regulation of transcription, DNA-dependent
		GO:0008270	zinc ion binding
CG15440	NM_134993	GO:0000166	nucleotide binding
		GO:0000398	nuclear mRNA splicing, via spliceosome
		GO:0019538	protein metabolic process
CG15434	NM_144084	GO:0003954	NADH dehydrogenase activity
		GO:0005747	mitochondrial respiratory chain complex I
		GO:0006120	mitochondrial electron transport, NADH to ubiquinone
Gs1l	NM_057881	GO:0004356	glutamate-ammonia ligase activity
		GO:0008152	metabolic process
		GO:0016787	hydrolase activity
RpL27A	NM_057615	GO:0003676	nucleic acid binding
		GO:0003735	structural constituent of ribosome
		GO:0005840	ribosome
		GO:0005842	cytosolic large ribosomal subunit (sensu Eukaryota)
		GO:0006412	translation
mRpL27	NM_175957	GO:0003735	structural constituent of ribosome
		GO:0005762	mitochondrial large ribosomal subunit
		GO:0006412	translation

NAME	RefSeq	Function	
CG15435	NM_134994	GO:0003676	nucleic acid binding
		GO:0005634	nucleus
		GO:0008270	zinc ion binding
CG15443	NM_134995	GO:0005488	binding
CG15436	NM_134996	GO:0003676	nucleic acid binding
		GO:0005634	nucleus
		GO:0006139	nucleobase, nucleoside, nucleotide and nucleic acid metaboli
		GO:0006357	regulation of transcription from RNA polymerase II promoter
		GO:0006366	transcription from RNA polymerase II promoter
		GO:0008270	zinc ion binding
		GO:0008283	cell proliferation
		GO:0030528	transcription regulator activity
CG17840	NM_134997		

GO density (14 genes):

RANKING	GO id	Function	Frequency
1	GO:0003676	nucleic acid binding	28 %
2	GO:0008270	zinc ion binding	28 %
3	GO:0005634	nucleus	14 %
4	GO:0006412	translation	14 %
5	GO:0019538	protein metabolic process	14 %
6	GO:0006139	nucleobase, nucleoside, nucleotide and nucleic acid metaboli	14 %
7	GO:0003735	structural constituent of ribosome	14 %
8	GO:0015986	ATP synthesis coupled proton transport	7 %
9	GO:0004842	ubiquitin-protein ligase activity	7 %
10	GO:0006366	transcription from RNA polymerase II promoter	7 %
11	GO:0006858	extracellular transport	7 %
12	GO:0016469	proton-transporting two-sector ATPase complex	7 %
13	GO:0046961	hydrogen ion transporting ATPase activity, rotational mechan	7 %
14	GO:0016407	acetyltransferase activity	7 %
15	GO:0016021	integral to membrane	7 %
16	GO:0000166	nucleotide binding	7 %
17	GO:0006120	mitochondrial electron transport, NADH to ubiquinone	7 %
18	GO:0008283	cell proliferation	7 %
19	GO:0006357	regulation of transcription from RNA polymerase II promoter	7 %
20	GO:0016567	protein ubiquitination	7 %
21	GO:0030528	transcription regulator activity	7 %
22	GO:0006355	regulation of transcription, DNA-dependent	7 %
23	GO:0005506	iron ion binding	7 %
24	GO:0006333	chromatin assembly or disassembly	7 %
25	GO:0005840	ribosome	7 %
26	GO:0006817	phosphate transport	7 %
27	GO:0046668	regulation of retinal programmed cell death	7 %
28	GO:0006915	apoptosis	7 %
29	GO:0005488	binding	7 %
30	GO:0006812	cation transport	7 %
31	GO:0000398	nuclear mRNA splicing, via spliceosome	7 %
32	GO:0003954	NADH dehydrogenase activity	7 %
33	GO:0008152	metabolic process	7 %
34	GO:0046933	hydrogen ion transporting ATP synthase activity, rotational	7 %
35	GO:0015321	sodium-dependent phosphate transporter activity	7 %
36	GO:0006796	phosphate metabolic process	7 %
37	GO:0008080	N-acetyltransferase activity	7 %
38	GO:0006917	induction of apoptosis	7 %
39	GO:0005515	protein binding	7 %
40	GO:0005747	mitochondrial respiratory chain complex I	7 %
41	GO:0016787	hydrolase activity	7 %
42	GO:0005762	mitochondrial large ribosomal subunit	7 %
43	GO:0005842	cytosolic large ribosomal subunit (sensu Eukaryota)	7 %
44	GO:0019005	SCF ubiquitin ligase complex	7 %
45	GO:0046675	induction of compound eye retinal cell programmed cell death	7 %
46	GO:0004356	glutamate-ammonia ligase activity	7 %

globalDown – chr2L: 8190286 - 8215239

Genomic components: 3 coregulated genes, 11 genes

CHR	Strand	Start	End	RefSeq	Name	Exons	Description
CHR2L	-	8190286	8190781	NM_001042882	CG34134	1	CG34134-PA
CHR2L	-	8190953	8196893	NM_135345	CG8475	10	CG8475-PA, isoform A
CHR2L	+	8197195	8198820	NM_135346	CG8460	5	CG8460-PA
CHR2L	+	8198958	8200651	NM_135347	CG8455	4	CG8455-PB, isoform B
CHR2L	-	8200464	8205128	NM_135348	CG8451	10	CG8451-PA
CHR2L	+	8206205	8209637	NM_135349	CG8419	5	CG8419-PA
CHR2L	-	8209625	8210920	NM_164799	Su(var)205	4	Suppressor of variegation 205 CG8409-PB, isoform
CHR2L	+	8211362	8211929	NM_057788	Ssb-c31a	2	Single stranded-binding protein c31A CG8396-PA
CHR2L	-	8212038	8212881	NM_135350	CG8372	2	CG8372-PA, isoform A
CHR2L	+	8213178	8214134	NM_135351	CG8360	3	CG8360-PA
CHR2L	+	8214304	8215239	NM_135352	CG8353	2	CG8353-PA

Cluster size: 24954 nucleotides

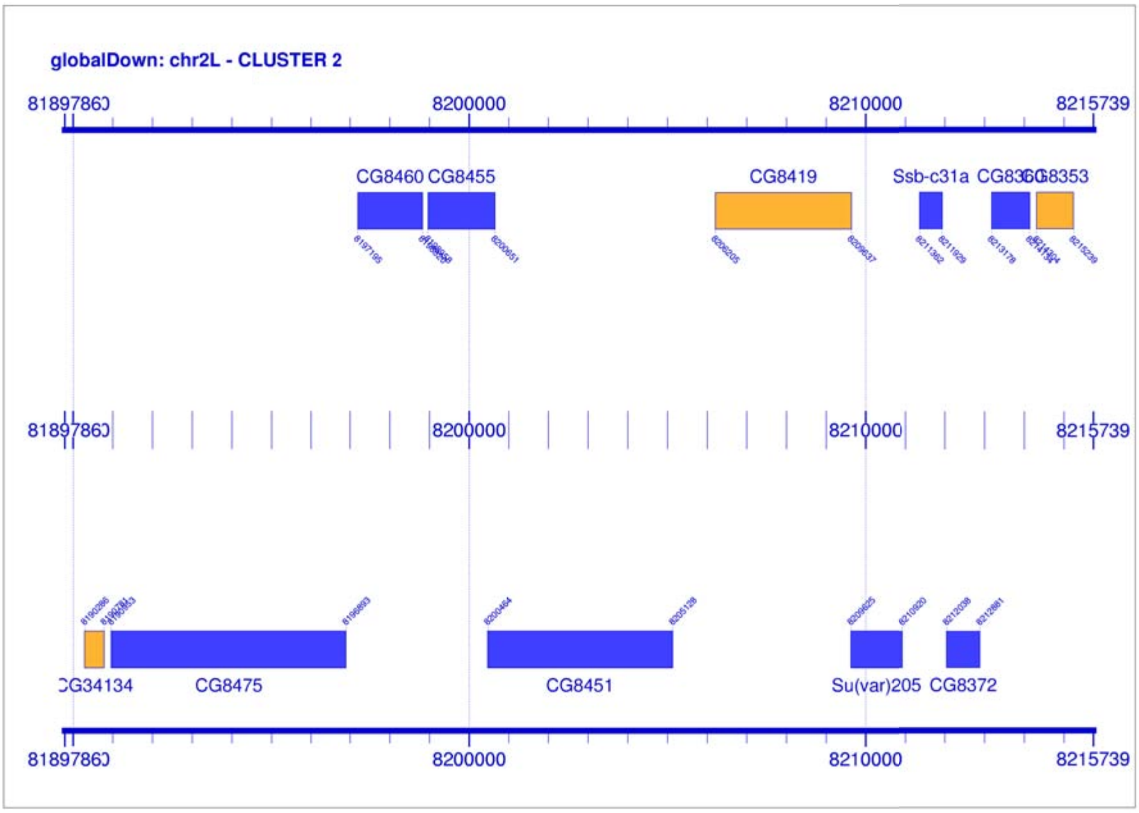

globalDown – cluster 2

Genomic components:

Name	RefSeq	Function	
CG34134	NM_001042882	GO:0003674	molecular_function
		GO:0005575	cellular_component
		GO:0008150	biological_process
CG8475	NM_135345	GO:0005516	calmodulin binding
		GO:0005964	phosphorylase kinase complex
		GO:0005977	glycogen metabolic process
		GO:0008607	phosphorylase kinase regulator activity
		GO:0019209	kinase activator activity
CG8460	NM_135346		
CG8455	NM_135347	GO:0016787	hydrolase activity
CG8451	NM_135348	GO:0006732	coenzyme metabolic process
		GO:0006812	cation transport
		GO:0006858	extracellular transport
		GO:0008324	cation transporter activity
		GO:0008523	sodium-dependent multivitamin transporter activity
		GO:0016020	membrane
		GO:0051189	prosthetic group metabolic process
CG8419	NM_135349	GO:0005515	protein binding
		GO:0005524	ATP binding
		GO:0008270	zinc ion binding
		GO:0015986	ATP synthesis coupled proton transport
		GO:0016469	proton-transporting two-sector ATPase complex
		GO:0046933	hydrogen ion transporting ATP synthase activity, rotational
		GO:0046961	hydrogen ion transporting ATPase activity, rotational mechan
Su(var)205	NM_164799	GO:0000723	telomere maintenance
		GO:0000775	chromosome, pericentric region
		GO:0000781	chromosome, telomeric region
		GO:0000792	heterochromatin
		GO:0003682	chromatin binding
		GO:0003729	mRNA binding
		GO:0005634	nucleus
		GO:0005701	polytene chromosome chromocenter
		GO:0005703	polytene chromosome puff
		GO:0005720	nuclear heterochromatin
		GO:0005721	centric heterochromatin
		GO:0006333	chromatin assembly or disassembly
		GO:0006342	chromatin silencing
		GO:0006343	establishment of chromatin silencing
		GO:0006357	regulation of transcription from RNA polymerase II promoter
		GO:0016563	transcriptional activator activity
		GO:0016564	transcriptional repressor activity
		GO:0030702	chromatin silencing at centromere
		GO:0035012	polytene chromosome, telomeric region
		GO:0035064	methylated histone residue binding
		GO:0042393	histone binding
		GO:0045892	negative regulation of transcription, DNA-dependent
		GO:0045893	positive regulation of transcription, DNA-dependent
Ssb-c31a	NM_057788	GO:0003697	single-stranded DNA binding
		GO:0003713	transcription coactivator activity
		GO:0005667	transcription factor complex
		GO:0006357	regulation of transcription from RNA polymerase II promoter
CG8372	NM_135350		

NAME	RefSeq	Function	
CG8360	NM_135351	GO:0004126	cytidine deaminase activity
		GO:0006206	pyrimidine base metabolic process
		GO:0008270	zinc ion binding
		GO:0046087	cytidine metabolic process
CG8353	NM_135352	GO:0004126	cytidine deaminase activity
		GO:0006206	pyrimidine base metabolic process
		GO:0008270	zinc ion binding
		GO:0046087	cytidine metabolic process

GO density (11 genes):

RANKING	GO id	Function	Frequency
1	GO:0008270	zinc ion binding	27 %
2	GO:0046087	cytidine metabolic process	18 %
3	GO:0006206	pyrimidine base metabolic process	18 %
4	GO:0006357	regulation of transcription from RNA polymerase II promoter	18 %
5	GO:0004126	cytidine deaminase activity	18 %
6	GO:0000723	telomere maintenance	9 %
7	GO:0008324	cation transporter activity	9 %
8	GO:0008523	sodium-dependent multivitamin transporter activity	9 %
9	GO:0005701	polytene chromosome chromocenter	9 %
10	GO:0003713	transcription coactivator activity	9 %
11	GO:0051189	prosthetic group metabolic process	9 %
12	GO:0015986	ATP synthesis coupled proton transport	9 %
13	GO:0000775	chromosome, pericentric region	9 %
14	GO:0006858	extracellular transport	9 %
15	GO:0016469	proton-transporting two-sector ATPase complex	9 %
16	GO:0005634	nucleus	9 %
17	GO:0005575	cellular_component	9 %
18	GO:0005964	phosphorylase kinase complex	9 %
19	GO:0046961	hydrogen ion transporting ATPase activity, rotational mechan	9 %
20	GO:0005720	nuclear heterochromatin	9 %
21	GO:0005516	calmodulin binding	9 %
22	GO:0008607	phosphorylase kinase regulator activity	9 %
23	GO:0005667	transcription factor complex	9 %
24	GO:0006732	coenzyme metabolic process	9 %
25	GO:0005977	glycogen metabolic process	9 %
26	GO:0003674	molecular_function	9 %
27	GO:0030702	chromatin silencing at centromere	9 %
28	GO:0045893	positive regulation of transcription, DNA-dependent	9 %
29	GO:0005524	ATP binding	9 %
30	GO:0006333	chromatin assembly or disassembly	9 %
31	GO:0042393	histone binding	9 %
32	GO:0008150	biological_process	9 %
33	GO:0016564	transcriptional repressor activity	9 %
34	GO:0005721	centric heterochromatin	9 %
35	GO:0016563	transcriptional activator activity	9 %
36	GO:0006812	cation transport	9 %
37	GO:0019209	kinase activator activity	9 %
38	GO:0003729	mRNA binding	9 %
39	GO:0006342	chromatin silencing	9 %
40	GO:0045892	negative regulation of transcription, DNA-dependent	9 %
41	GO:0046933	hydrogen ion transporting ATP synthase activity, rotational	9 %
42	GO:0000792	heterochromatin	9 %
43	GO:0003682	chromatin binding	9 %
44	GO:0016787	hydrolase activity	9 %
45	GO:0005515	protein binding	9 %
46	GO:0003697	single-stranded DNA binding	9 %
47	GO:0035064	methylated histone residue binding	9 %
48	GO:0035012	polytene chromosome, telomeric region	9 %
49	GO:0000781	chromosome, telomeric region	9 %
50	GO:0016020	membrane	9 %
51	GO:0005703	polytene chromosome puff	9 %
52	GO:0006343	establishment of chromatin silencing	9 %

globalDown – chr3L: 7097159 - 7131020

Genomic components: 3 coregulated genes, 9 genes

CHR	Strand	Start	End	RefSeq	Name	Exons	Description
CHR3L	-	7097159	7099470	NM_079227	msl-3	6	male-specific lethal 3 CG8631-PA, isoform A
CHR3L	+	7099577	7101833	NM_139823	BBS1	3	BBS1 CG14825-PA
CHR3L	-	7101859	7102207	NM_139824	CG8629	1	CG8629-PA
CHR3L	+	7102898	7103219	NM_139825	CG15829	2	CG15829-PA
CHR3L	+	7104948	7105334	NM_139826	CG8628	2	CG8628-PA
CHR3L	+	7105817	7107410	NM_168192	Dbi	3	Diazepam-binding inhibitor CG8627-PA, isoform A
CHR3L	+	7108464	7116682	NM_001014572	melt	10	melted CG8624-PB, isoform B
CHR3L	-	7117723	7118309	NM_168193	CG32390	2	CG32390-PA
CHR3L	-	7118470	7131020	NM_079230	corn	6	cornetto CG32386-PA

Cluster size: 33862 nucleotides

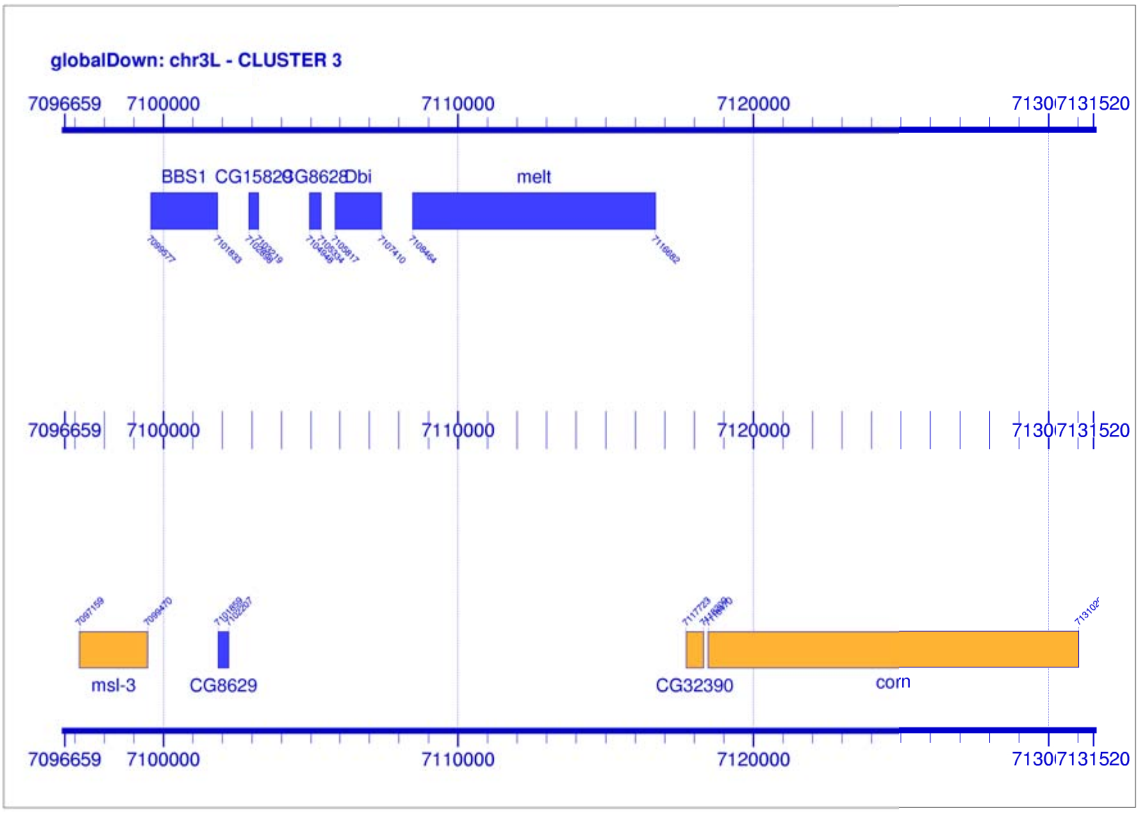

globalDown – cluster 3

Genomic components:

NAME	RefSeq	Function	
MSL-3	NM_079227	GO:0000785	chromatin
		GO:0000805	X chromosome
		GO:0003682	chromatin binding
		GO:0003729	mRNA binding
		GO:0005515	protein binding
		GO:0005634	nucleus
		GO:0005694	chromosome
		GO:0006333	chromatin assembly or disassembly
		GO:0006357	regulation of transcription from RNA polymerase II promoter
		GO:0007549	dosage compensation
		GO:0009047	dosage compensation, by hyperactivation of X chromosome
		GO:0016456	X chromosome located dosage compensation complex, transcript
		GO:0030528	transcription regulator activity
BBS1	NM_139823	GO:0003674	molecular_function
		GO:0005575	cellular_component
		GO:0042384	cilium biogenesis
CG8629	NM_139824	GO:0000062	acyl-CoA binding
		GO:0004857	enzyme inhibitor activity
		GO:0005386	carrier activity
		GO:0006869	lipid transport
		GO:0042049	cell acyl-CoA homeostasis
		GO:0050809	diazepam binding
CG15829	NM_139825	GO:0000062	acyl-CoA binding
		GO:0004857	enzyme inhibitor activity
		GO:0005386	carrier activity
		GO:0006869	lipid transport
		GO:0042049	cell acyl-CoA homeostasis
		GO:0050809	diazepam binding
CG8628	NM_139826	GO:0000062	acyl-CoA binding
		GO:0004857	enzyme inhibitor activity
		GO:0005386	carrier activity
		GO:0006869	lipid transport
		GO:0042049	cell acyl-CoA homeostasis
		GO:0050809	diazepam binding
DBI	NM_168192	GO:0000062	acyl-CoA binding
		GO:0004857	enzyme inhibitor activity
		GO:0005386	carrier activity
		GO:0006869	lipid transport
		GO:0042049	cell acyl-CoA homeostasis
		GO:0050809	diazepam binding
MELT	NM_1014572	GO:0005488	binding
		GO:0007398	ectoderm development
		GO:0007422	peripheral nervous system development
CG32390	NM_168193		
CORN	NM_079230	GO:0005515	protein binding
		GO:0005737	cytoplasm
		GO:0005938	cell cortex
		GO:0008017	microtubule binding
		GO:0045177	apical part of cell

GO density (9 genes):

RANKING	GO id	Function	Frequency
1	GO:0006869	lipid transport	44 %
2	GO:0005386	carrier activity	44 %
3	GO:0000062	acyl-CoA binding	44 %
4	GO:0042049	cell acyl-CoA homeostasis	44 %
5	GO:0050809	diazepam binding	44 %
6	GO:0004857	enzyme inhibitor activity	44 %
7	GO:0005515	protein binding	22 %
8	GO:0042384	cilium biogenesis	11 %
9	GO:0000785	chromatin	11 %
10	GO:0005634	nucleus	11 %
11	GO:0005575	cellular_component	11 %
12	GO:0006357	regulation of transcription from RNA polymerase II promoter	11 %
13	GO:0003674	molecular_function	11 %
14	GO:0030528	transcription regulator activity	11 %
15	GO:0000805	X chromosome	11 %
16	GO:0006333	chromatin assembly or disassembly	11 %
17	GO:0016456	X chromosome located dosage compensation complex, transcript	11 %
18	GO:0007422	peripheral nervous system development	11 %
19	GO:0045177	apical part of cell	11 %
20	GO:0009047	dosage compensation, by hyperactivation of X chromosome	11 %
21	GO:0007398	ectoderm development	11 %
22	GO:0005488	binding	11 %
23	GO:0008017	microtubule binding	11 %
24	GO:0005694	chromosome	11 %
25	GO:0007549	dosage compensation	11 %
26	GO:0003729	mRNA binding	11 %
27	GO:0003682	chromatin binding	11 %
28	GO:0005737	cytoplasm	11 %
29	GO:0005938	cell cortex	11 %

globalDown – chr3L: 9350364 - 9356712

Genomic components: 3 coregulated genes, 3 genes

CHR	Strand	Start	End	RefSeq	Name	Exons	Description
CHR3L	-	9350364	9351374	NM_079273	Hsp26	1	Heat shock protein 26 CG4183-PA
CHR3L	-	9352049	9353386	NM_079274	Hsp67Ba	1	Heat shock gene 67Ba CG4167-PA
CHR3L	+	9355830	9356712	NM_079275	Hsp23	1	Heat shock protein 23 CG4463-PA

Cluster size: 6349 nucleotides

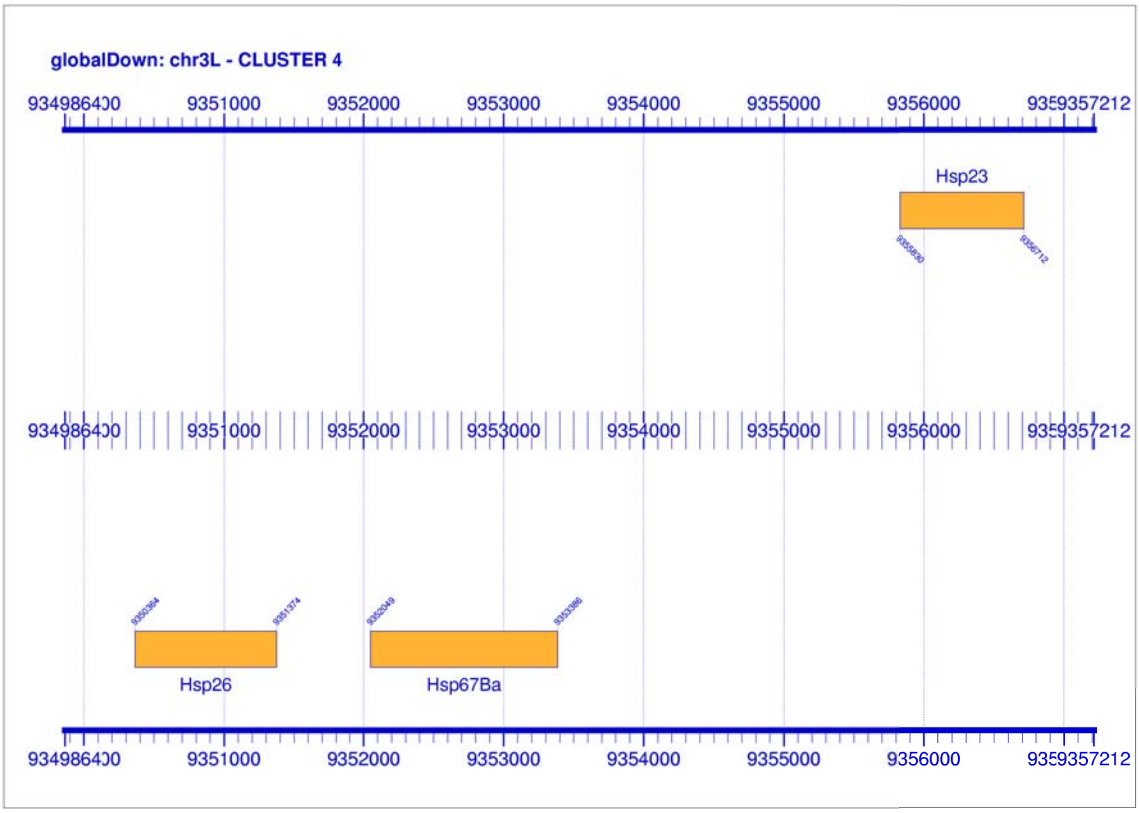

globalDown – cluster 4

Genomic components:

Name	RefSeq	Function	
Hsp26	NM_079273	GO:0006457	protein folding
		GO:0006952	defense response
		GO:0008340	determination of adult life span
		GO:0009408	response to heat
Hsp67Ba	NM_079274	GO:0006457	protein folding
		GO:0006950	response to stress
		GO:0006952	defense response
		GO:0009408	response to heat
Hsp23	NM_079275	GO:0003779	actin binding
		GO:0006457	protein folding
		GO:0006952	defense response
		GO:0009408	response to heat

GO density (3 genes):

Ranking	GO id	Function	Frequency
1	GO:0006952	defense response	100 %
2	GO:0009408	response to heat	100 %
3	GO:0006457	protein folding	100 %
4	GO:0008340	determination of adult life span	33 %
5	GO:0003779	actin binding	33 %
6	GO:0006950	response to stress	33 %

globalDown – chr3L: 9382122 - 9403764

Genomic components: 4 coregulated genes, 7 genes

CHR	Strand	Start	End	RefSeq	Name	Exons	Description
CHR3L	-	9382122	9384778	NM_140050	CG4022	4	CG4022-PA
CHR3L	+	9385005	9387582	NM_001043132	CG3689	5	CG3689-PC, isoform C
CHR3L	-	9387542	9393308	NM_140052	CG3967	6	CG3967-PA, isoform A
CHR3L	-	9389701	9391335	NM_170633	CG3982	4	CG3982-PA
CHR3L	+	9397199	9398749	NM_079277	aay	1	astray CG3705-PA
CHR3L	+	9400946	9402625	NM_079944	Shc	2	SHC-adaptor protein CG3715-PA
CHR3L	-	9402635	9403764	NM_079278	RpS17	4	Ribosomal protein S17 CG3922-PB

Cluster size: 21643 nucleotides

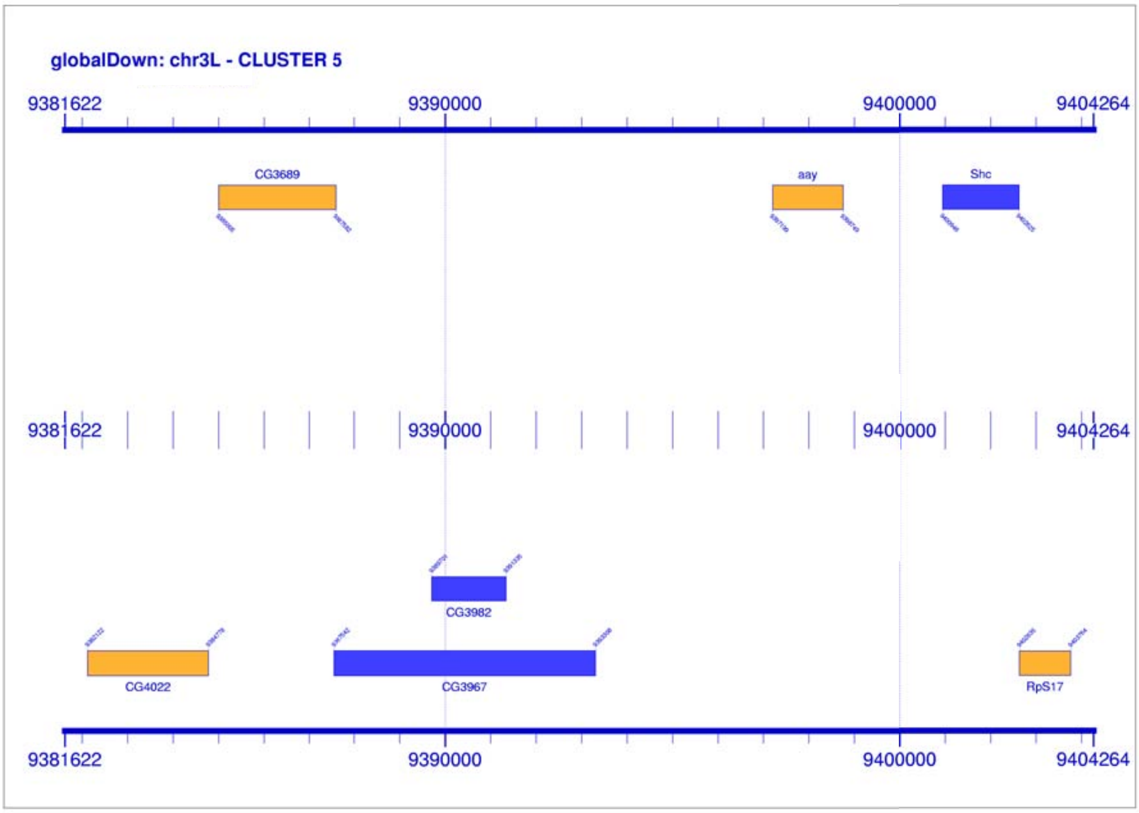

globalDown – cluster 5

Genomic components:

NAME	RefSeq	Function	
CG4022	NM_140050		
CG3689	NM_001043132	GO:0000398	nuclear mRNA splicing, via spliceosome
		GO:0005849	mRNA cleavage factor complex
		GO:0006379	mRNA cleavage
CG3967	NM_140052		
CG3982	NM_170633		
AAY	NM_079277	GO:0004647	phosphoserine phosphatase activity
		GO:0005575	cellular_component
		GO:0006564	L-serine biosynthetic process
		GO:0007411	axon guidance
		GO:0007422	peripheral nervous system development
SHC	NM_079944	GO:0000074	regulation of progression through cell cycle
		GO:0000165	MAPKKK cascade
		GO:0005102	receptor binding
		GO:0007173	epidermal growth factor receptor signaling pathway
		GO:0008293	torso signaling pathway
		GO:0019226	transmission of nerve impulse
RPS17	NM_079278	GO:0003676	nucleic acid binding
		GO:0003735	structural constituent of ribosome
		GO:0005840	ribosome
		GO:0005843	cytosolic small ribosomal subunit (sensu Eukaryota)
		GO:0006412	translation

GO density (7 genes):

RANKING	GO id	Function	Frequency
1	GO:0006629	lipid metabolic process	28 %
2	GO:0017059	serine C-palmitoyltransferase complex	14 %
3	GO:0003676	nucleic acid binding	14 %
4	GO:0048813	dendrite morphogenesis	14 %
5	GO:0006366	transcription from RNA polymerase II promoter	14 %
6	GO:0005634	nucleus	14 %
7	GO:0008270	zinc ion binding	14 %
8	GO:0003712	transcription cofactor activity	14 %
9	GO:0004758	serine C-palmitoyltransferase activity	14 %
10	GO:0030517	negative regulation of axon extension	14 %
11	GO:0005977	glycogen metabolic process	14 %
12	GO:0006357	regulation of transcription from RNA polymerase II promoter	14 %
13	GO:0003844	1,4-alpha-glucan branching enzyme activity	14 %
14	GO:0030528	transcription regulator activity	14 %
15	GO:0006869	lipid transport	14 %
16	GO:0016769	transferase activity, transferring nitrogenous groups	14 %
17	GO:0005386	carrier activity	14 %
18	GO:0043169	cation binding	14 %
19	GO:0007409	axonogenesis	14 %
20	GO:0005488	binding	14 %
21	GO:0006139	nucleobase, nucleoside, nucleotide and nucleic acid metaboli	14 %
22	GO:0007154	cell communication	14 %
23	GO:0007165	signal transduction	14 %
24	GO:0046879	hormone secretion	14 %
25	GO:0009058	biosynthetic process	14 %
26	GO:0005615	extracellular space	14 %

globalDown – chr3L: 16327861 - 16343909

Genomic components: 3 coregulated genes, 6 genes

CHR	Strand	Start	End	RefSeq	Name	Exons	Description
CHR3L	+	16327861	16328939	NM_140625	CG4818	1	CG4818-PA
CHR3L	+	16330255	16330907	NM_140626	CG12255	1	CG12255-PA
CHR3L	-	16331152	16332585	NM_140627	CG4784	1	CG4784-PA
CHR3L	+	16333665	16334777	NM_140628	CG4842	4	CG4842-PA
CHR3L	+	16335134	16336101	NM_144416	CG18814	3	CG18814-PA
CHR3L	-	16338311	16343909	NM_140629	CG16807	4	CG16807-PA

Cluster size: 16049 nucleotides

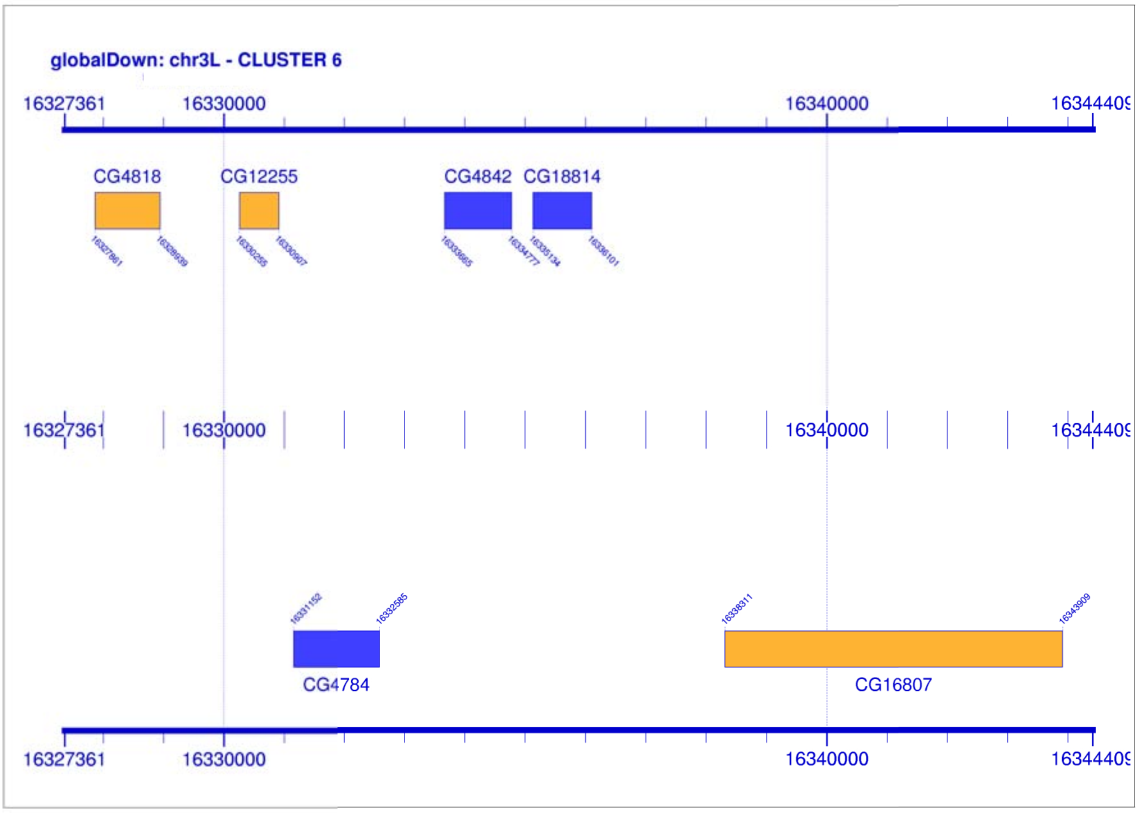

globalDown – cluster 6

Genomic components:

Name	RefSeq	Function	
CG4818	NM_140625	GO:0005214	structural constituent of chitin-based cuticle
CG12255	NM_140626	GO:0042302	structural constituent of cuticle
CG4784	NM_140627	GO:0042302	structural constituent of cuticle
CG4842	NM_140628	GO:0004022<br>GO:0006629	alcohol dehydrogenase activity<br>lipid metabolic process
CG18814	NM_144416	GO:0004022<br>GO:0006066<br>GO:0006629<br>GO:0008152<br>GO:0016491	alcohol dehydrogenase activity<br>alcohol metabolic process<br>lipid metabolic process<br>metabolic process<br>oxidoreductase activity
CG16807	NM_140629	GO:0003676<br>GO:0005515<br>GO:0008270	nucleic acid binding<br>protein binding<br>zinc ion binding

GO density (6 genes):

Ranking	GO id	Function	Frequency
1	GO:0042302	structural constituent of cuticle	33 %
2	GO:0006629	lipid metabolic process	33 %
3	GO:0004022	alcohol dehydrogenase activity	33 %
4	GO:0003676	nucleic acid binding	16 %
5	GO:0016491	oxidoreductase activity	16 %
6	GO:0008270	zinc ion binding	16 %
7	GO:0005214	structural constituent of chitin-based cuticle	16 %
8	GO:0008152	metabolic process	16 %
9	GO:0005515	protein binding	16 %
10	GO:0006066	alcohol metabolic process	16 %

globalDown – chr2R: 2952505 - 2970676

Genomic components: 4 coregulated genes, 7 genes

Chr	Strand	Start	End	RefSeq	Name	Exons	Description
chr2R	+	2952505	2954698	NM_136432	CG11123	2	CG11123-PA
chr2R	+	2954676	2955655	NM_001014501	sPLA2	2	secretory Phospholipase A2 CG11124-PC, isoform
chr2R	-	2955636	2956439	NM_165524	CG30503	2	CG30503-PA
chr2R	-	2956835	2957574	NM_136434	kappaB-Ras	2	kappaB-Ras CG1669-PA
chr2R	-	2957988	2963364	NM_136435	CG30502	4	CG30502-PA
chr2R	+	2959291	2961228	NM_136436	CG11125	5	CG11125-PA
chr2R	-	2966729	2970676	NM_058057	Inos	5	Inos CG11143-PA

Cluster size: 18172 nucleotides

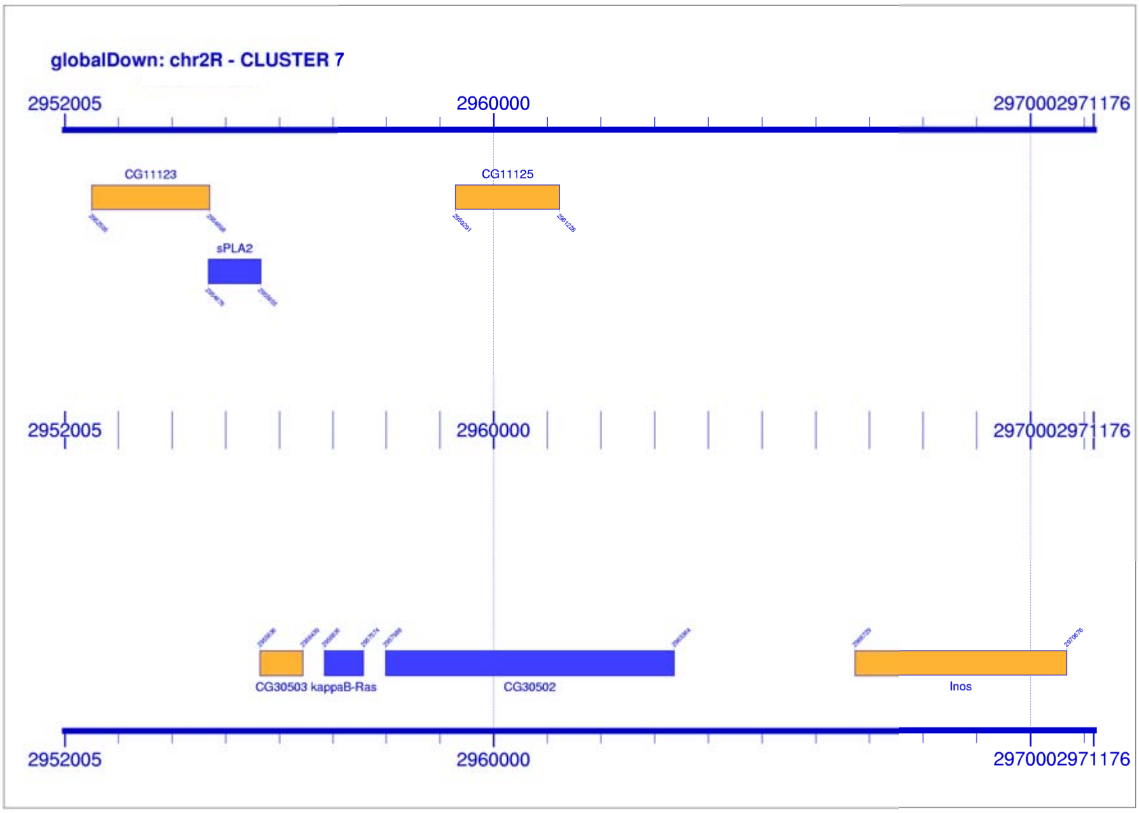

globalDown – cluster 7

Genomic components:

Name	RefSeq	Function	
CG11123	NM_136432		
sPLA2	NM_001014501	GO:0004623	phospholipase A2 activity
		GO:0005509	calcium ion binding
		GO:0005576	extracellular region
		GO:0006644	phospholipid metabolic process
		GO:0016042	lipid catabolic process
		GO:0047498	calcium-dependent phospholipase A2 activity
CG30503	NM_165524		
kappaB-Ras	NM_136434	GO:0005515	protein binding
		GO:0005525	GTP binding
		GO:0005575	cellular_component
		GO:0007264	small GTPase mediated signal transduction
		GO:0043124	negative regulation of I-kappaB kinase/NF-kappaB cascade
CG30502	NM_136435	GO:0003824	catalytic activity
		GO:0008152	metabolic process
CG11125	NM_136436		
Inos	NM_058057	GO:0004512	inositol-3-phosphate synthase activity
		GO:0005737	cytoplasm
		GO:0006021	inositol biosynthetic process
		GO:0006644	phospholipid metabolic process
		GO:0008654	phospholipid biosynthetic process
		GO:0016849	phosphorus-oxygen lyase activity

GO density (7 genes):

Ranking	GO id	Function	Frequency
1	GO:0006644	phospholipid metabolic process	28 %
2	GO:0005509	calcium ion binding	14 %
3	GO:0016849	phosphorus-oxygen lyase activity	14 %
4	GO:0005575	cellular_component	14 %
5	GO:0043124	negative regulation of I-kappaB kinase/NF-kappaB cascade	14 %
6	GO:0005576	extracellular region	14 %
7	GO:0007264	small GTPase mediated signal transduction	14 %
8	GO:0004512	inositol-3-phosphate synthase activity	14 %
9	GO:0016042	lipid catabolic process	14 %
10	GO:0005525	GTP binding	14 %
11	GO:0004623	phospholipase A2 activity	14 %
12	GO:0003824	catalytic activity	14 %
13	GO:0047498	calcium-dependent phospholipase A2 activity	14 %
14	GO:0008152	metabolic process	14 %
15	GO:0005515	protein binding	14 %
16	GO:0006021	inositol biosynthetic process	14 %
17	GO:0008654	phospholipid biosynthetic process	14 %
18	GO:0005737	cytoplasm	14 %

globalDown – chr2R: 3008513 - 3037521

Genomic components: 4 coregulated genes, 8 genes

CHR	Strand	Start	End	RefSeq	Name	Exons	Description
CHR2R	-	3008513	3011687	NM_057837	dpa	7	disc proliferation abnormal CG1616-PA
CHR2R	+	3011967	3020438	NM_057838	didum	17	dilute class unconventional myosin CG2146-PA,
CHR2R	-	3020339	3022267	NM_136444	CG12736	3	CG12736-PA
CHR2R	-	3022423	3024537	NM_136445	az2	3	az2 CG1605-PA
CHR2R	-	3025884	3028183	NM_136446	CG1603	4	CG1603-PA
CHR2R	-	3028792	3030724	NM_136447	CG1602	3	CG1602-PA
CHR2R	+	3031092	3034359	NM_136448	CG2144	7	CG2144-PA
CHR2R	-	3034249	3037521	NM_057955	Orc1	3	Origin recognition complex subunit 1 CG10667-PA

Cluster size: 29009 nucleotides

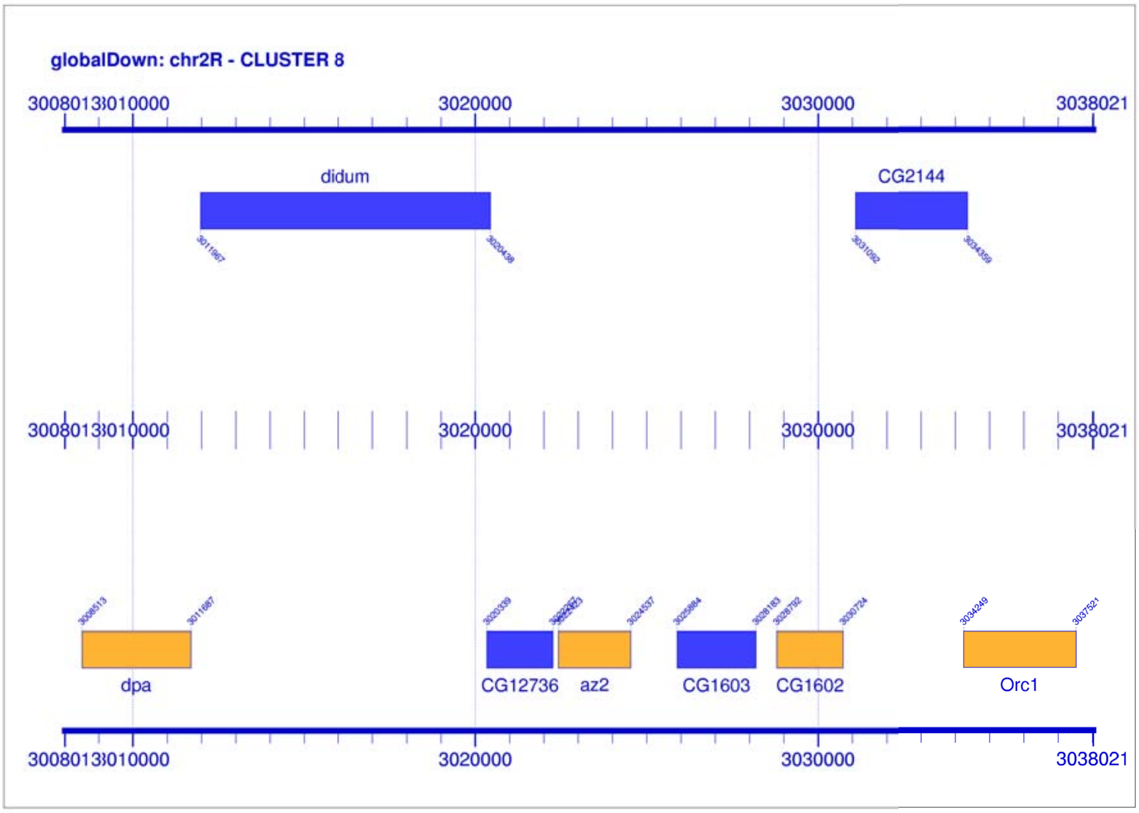

globalDown – cluster 8

Genomic components:

Name	RefSeq	Function	
DPA	NM_057837	GO:0003677	DNA binding
		GO:0003678	DNA helicase activity
		GO:0003688	DNA replication origin binding
		GO:0005524	ATP binding
		GO:0005529	sugar binding
		GO:0005634	nucleus
		GO:0006260	DNA replication
		GO:0006270	DNA replication initiation
		GO:0007052	mitotic spindle organization and biogenesis
		GO:0008094	DNA-dependent ATPase activity
DIDUM	NM_057838	GO:0003774	motor activity
		GO:0003779	actin binding
		GO:0005200	structural constituent of cytoskeleton
		GO:0005516	calmodulin binding
		GO:0005524	ATP binding
		GO:0006403	RNA localization
		GO:0006886	intracellular protein transport
		GO:0007291	sperm individualization
		GO:0008092	cytoskeletal protein binding
		GO:0016192	vesicle-mediated transport
		GO:0016459	myosin complex
		GO:0042623	ATPase activity, coupled
CG12736	NM_136444	GO:0003746	translation elongation factor activity
		GO:0003924	GTPase activity
		GO:0005525	GTP binding
		GO:0005739	mitochondrion
		GO:0006412	translation
		GO:0006414	translational elongation
AZ2	NM_136445	GO:0003676	nucleic acid binding
		GO:0004867	serine-type endopeptidase inhibitor activity
		GO:0006357	regulation of transcription from RNA polymerase II promoter
		GO:0008270	zinc ion binding
		GO:0008283	cell proliferation
		GO:0030528	transcription regulator activity
CG1603	NM_136446	GO:0003676	nucleic acid binding
		GO:0008270	zinc ion binding
CG1602	NM_136447	GO:0003676	nucleic acid binding
		GO:0006139	nucleobase, nucleoside, nucleotide and nucleic acid metaboli
		GO:0006357	regulation of transcription from RNA polymerase II promoter
		GO:0006366	transcription from RNA polymerase II promoter
		GO:0008270	zinc ion binding
		GO:0008283	cell proliferation
		GO:0030528	transcription regulator activity
CG2144	NM_136448	GO:0006464	protein modification
		GO:0006497	protein amino acid lipidation
		GO:0019538	protein metabolic process
		GO:0046872	metal ion binding
ORC1	NM_057955	GO:0000074	regulation of progression through cell cycle
		GO:0003677	DNA binding
		GO:0003688	DNA replication origin binding
		GO:0005524	ATP binding
		GO:0005634	nucleus
		GO:0005664	nuclear origin of replication recognition complex
		GO:0006261	DNA-dependent DNA replication
		GO:0006270	DNA replication initiation
		GO:0006342	chromatin silencing
		GO:0017111	nucleoside-triphosphatase activity

NAME	RefSeq	Function	
CG1603	NM_136446	GO:0003676	nucleic acid binding
		GO:0008270	zinc ion binding
CG1602	NM_136447	GO:0003676	nucleic acid binding
		GO:0006139	nucleobase, nucleoside, nucleotide and nucleic acid metaboli
		GO:0006357	regulation of transcription from RNA polymerase II promoter
		GO:0006366	transcription from RNA polymerase II promoter
		GO:0008270	zinc ion binding
		GO:0008283	cell proliferation
		GO:0030528	transcription regulator activity
CG2144	NM_136448	GO:0006464	protein modification
		GO:0006497	protein amino acid lipidation
		GO:0019538	protein metabolic process
		GO:0046872	metal ion binding
ORC1	NM_057955	GO:0000074	regulation of progression through cell cycle
		GO:0003677	DNA binding
		GO:0003688	DNA replication origin binding
		GO:0005524	ATP binding
		GO:0005634	nucleus
		GO:0005664	nuclear origin of replication recognition complex
		GO:0006261	DNA-dependent DNA replication
		GO:0006270	DNA replication initiation
		GO:0006342	chromatin silencing
		GO:0017111	nucleoside-triphosphatase activity

GO density (8 genes):

Ranking	GO id	Function	Frequency
1	GO:0003676	nucleic acid binding	37 %
2	GO:0008270	zinc ion binding	37 %
3	GO:0005524	ATP binding	37 %
4	GO:0005634	nucleus	25 %
5	GO:0008283	cell proliferation	25 %
6	GO:0006357	regulation of transcription from RNA polymerase II promoter	25 %
7	GO:0030528	transcription regulator activity	25 %
8	GO:0003677	DNA binding	25 %
9	GO:0003688	DNA replication origin binding	25 %
10	GO:0006270	DNA replication initiation	25 %
11	GO:0007291	sperm individualization	12 %
12	GO:0006464	protein modification	12 %
13	GO:0006497	protein amino acid lipidation	12 %
14	GO:0046872	metal ion binding	12 %
15	GO:0003779	actin binding	12 %
16	GO:0042623	ATPase activity, coupled	12 %
17	GO:0006366	transcription from RNA polymerase II promoter	12 %
18	GO:0005200	structural constituent of cytoskeleton	12 %
19	GO:0005529	sugar binding	12 %
20	GO:0005664	nuclear origin of replication recognition complex	12 %
21	GO:0004867	serine-type endopeptidase inhibitor activity	12 %
22	GO:0000074	regulation of progression through cell cycle	12 %
23	GO:0005516	calmodulin binding	12 %
24	GO:0005739	mitochondrion	12 %
25	GO:0017111	nucleoside-triphosphatase activity	12 %
26	GO:0016192	vesicle-mediated transport	12 %
27	GO:0006403	RNA localization	12 %
28	GO:0006414	translational elongation	12 %
29	GO:0003678	DNA helicase activity	12 %
30	GO:0008094	DNA-dependent ATPase activity	12 %
31	GO:0005525	GTP binding	12 %
32	GO:0007052	mitotic spindle organization and biogenesis	12 %
33	GO:0006412	translation	12 %
34	GO:0006261	DNA-dependent DNA replication	12 %
35	GO:0016459	myosin complex	12 %
36	GO:0019538	protein metabolic process	12 %
37	GO:0006886	intracellular protein transport	12 %
38	GO:0003924	GTPase activity	12 %
39	GO:0006139	nucleobase, nucleoside, nucleotide and nucleic acid metaboli	12 %
40	GO:0008092	cytoskeletal protein binding	12 %
41	GO:0006342	chromatin silencing	12 %
42	GO:0003746	translation elongation factor activity	12 %
43	GO:0006260	DNA replication	12 %
44	GO:0003774	motor activity	12 %

globalDown – chr2R: 8679963 - 8714183

Genomic components: 4 coregulated genes, 7 genes

CHR	Strand	Start	End	RefSeq	Name	Exons	Description
CHR2R	-	8679963	8682513	NM_079003	GLaz	4	Glial Lazarillo CG4604-PA
CHR2R	+	8684406	8686445	NM_165967	Spt-I	6	Serine palmitoyltransferase subunit I CG4016-PB,
CHR2R	+	8687307	8690703	NM_176162	CG33138	4	CG33138-PA
CHR2R	+	8691100	8691980	NM_176163	CG33137	3	CG33137-PA
CHR2R	-	8691999	8701719	NM_143700	CG17724	4	CG17724-PA, isoform A
CHR2R	-	8695588	8705981	NM_001032244	seq	4	sequoia CG32904-PA, isoform A
CHR2R	-	8702179	8714183	NM_176164	CG33182	5	CG33182-PA

Cluster size: 34221 nucleotides

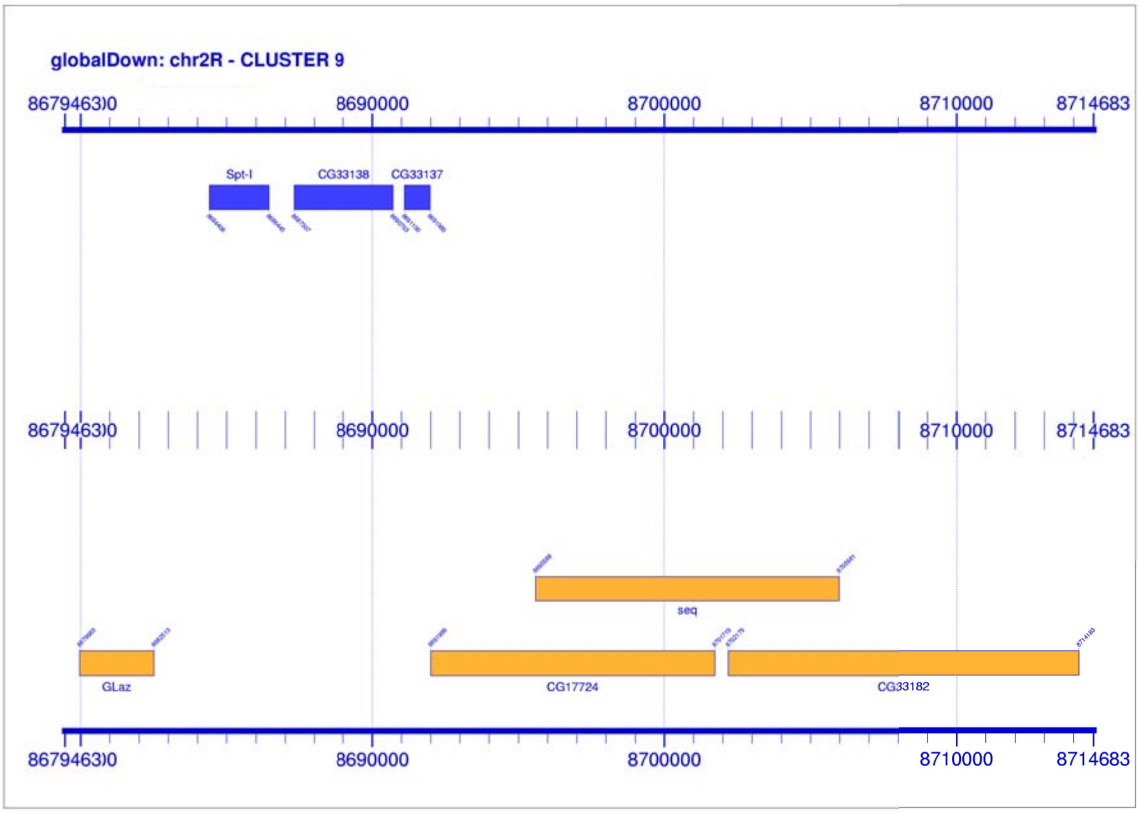

globalDown – cluster 9

Genomic components:

NAME	RefSeq	Function	
GLAZ	NM_079003	GO:0005386	carrier activity
		GO:0005488	binding
		GO:0005615	extracellular space
		GO:0006629	lipid metabolic process
		GO:0006869	lipid transport
SPT-I	NM_165967	GO:0004758	serine C-palmitoyltransferase activity
		GO:0006629	lipid metabolic process
		GO:0009058	biosynthetic process
		GO:0016769	transferase activity, transferring nitrogenous groups
		GO:0017059	serine C-palmitoyltransferase complex
CG33138	NM_176162	GO:0003844	1,4-alpha-glucan branching enzyme activity
		GO:0005977	glycogen metabolic process
		GO:0043169	cation binding
CG33137	NM_176163		
CG17724	NM_143700		
SEQ	NM_001032244	GO:0003676	nucleic acid binding
		GO:0005634	nucleus
		GO:0007409	axonogenesis
		GO:0008270	zinc ion binding
		GO:0030517	negative regulation of axon extension
		GO:0048813	dendrite morphogenesis
CG33182	NM_176164	GO:0003712	transcription cofactor activity
		GO:0006139	nucleobase, nucleoside, nucleotide and nucleic acid metaboli
		GO:0006357	regulation of transcription from RNA polymerase II promoter
		GO:0006366	transcription from RNA polymerase II promoter
		GO:0007154	cell communication
		GO:0007165	signal transduction
		GO:0030528	transcription regulator activity
		GO:0046879	hormone secretion

GO density (7 genes):

Ranking	GO id	Function	Frequency
1	GO:0006629	lipid metabolic process	28 %
2	GO:0017059	serine C-palmitoyltransferase complex	14 %
3	GO:0003676	nucleic acid binding	14 %
4	GO:0048813	dendrite morphogenesis	14 %
5	GO:0006366	transcription from RNA polymerase II promoter	14 %
6	GO:0005634	nucleus	14 %
7	GO:0008270	zinc ion binding	14 %
8	GO:0003712	transcription cofactor activity	14 %
9	GO:0004758	serine C-palmitoyltransferase activity	14 %
10	GO:0030517	negative regulation of axon extension	14 %
11	GO:0005977	glycogen metabolic process	14 %
12	GO:0006357	regulation of transcription from RNA polymerase II promoter	14 %
13	GO:0003844	1,4-alpha-glucan branching enzyme activity	14 %
14	GO:0030528	transcription regulator activity	14 %
15	GO:0006869	lipid transport	14 %
16	GO:0016769	transferase activity, transferring nitrogenous groups	14 %
17	GO:0005386	carrier activity	14 %
18	GO:0043169	cation binding	14 %
19	GO:0007409	axonogenesis	14 %
20	GO:0005488	binding	14 %
21	GO:0006139	nucleobase, nucleoside, nucleotide and nucleic acid metaboli	14 %
22	GO:0007154	cell communication	14 %
23	GO:0007165	signal transduction	14 %
24	GO:0046879	hormone secretion	14 %
25	GO:0009058	biosynthetic process	14 %
26	GO:0005615	extracellular space	14 %

globalDown – chr2R: 13650291 - 13675173

Genomic components: 3 coregulated genes, 7 genes

CHR	Strand	Start	End	RefSeq	Name	Exons	Description
CHR2R	+	13650291	13654319	NM_057324	Pcl	4	Polycomblike CG5109-PA
CHR2R	+	13654940	13661098	NM_166265	pAbp	5	polyA-binding protein CG5119-PC, isoform C
CHR2R	-	13662159	13662857	NM_137450	CG17680	1	CG17680-PA
CHR2R	-	13663309	13666250	NM_137451	CG5742	1	CG5742-PA
CHR2R	+	13666566	13668709	NM_137452	adp	2	adipose CG5124-PA
CHR2R	-	13669183	13672835	NM_080039	lolal	4	lola like CG5738-PA, isoform A
CHR2R	+	13673129	13675173	NM_137453	CG10914	1	CG10914-PA

Cluster size: 24883 nucleotides

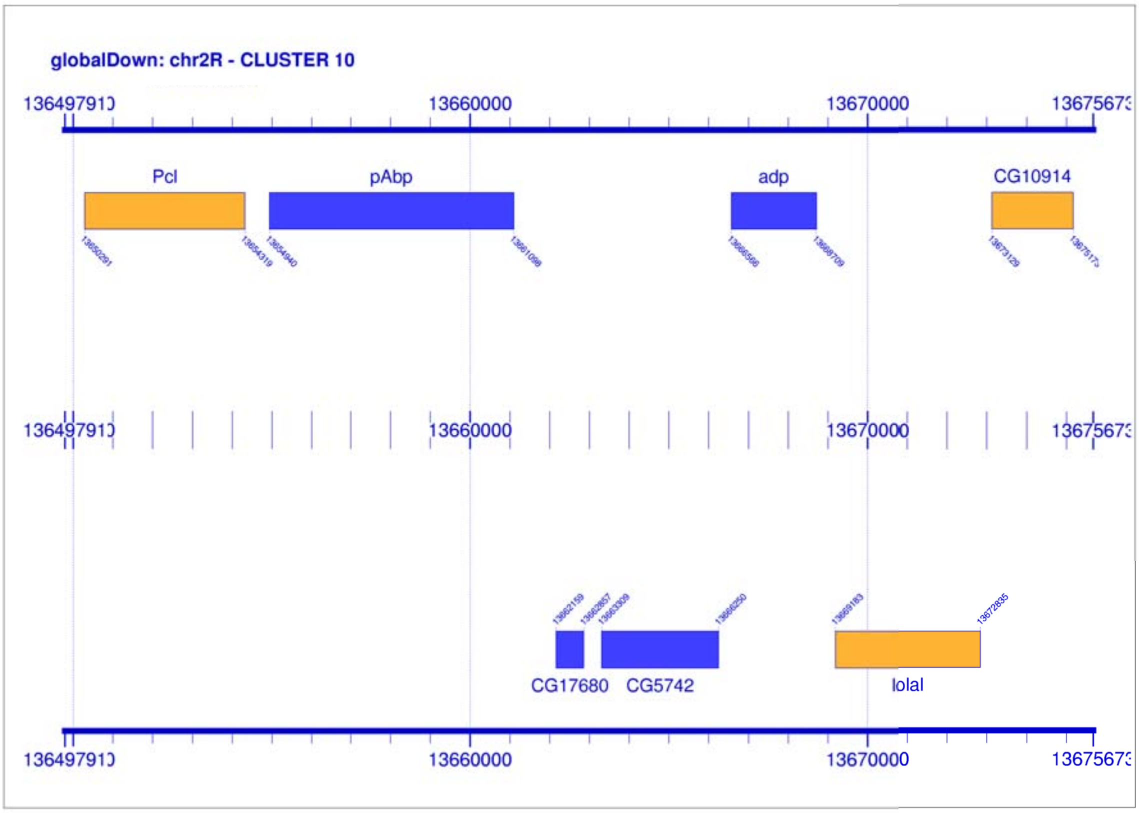

globalDown – cluster 10

Genomic components:

Name	RefSeq	Function	
PCL	NM_057324	GO:0003677	DNA binding
		GO:0003700	transcription factor activity
		GO:0005515	protein binding
		GO:0005634	nucleus
		GO:0006357	regulation of transcription from RNA polymerase II promoter
		GO:0008270	zinc ion binding
		GO:0016568	chromatin modification
		GO:0030528	transcription regulator activity
		GO:0045892	negative regulation of transcription, DNA-dependent
pABP	NM_166265	GO:0000166	nucleotide binding
		GO:0003729	mRNA binding
		GO:0005515	protein binding
		GO:0005737	cytoplasm
		GO:0006398	histone mRNA 3'-end processing
		GO:0007268	synaptic transmission
		GO:0008143	poly(A) binding
CG17680	NM_137450		
CG5742	NM_137451		
ADP	NM_137452	GO:0005507	copper ion binding
		GO:0005975	carbohydrate metabolic process
		GO:0006118	electron transport
		GO:0006629	lipid metabolic process
		GO:0009055	electron carrier activity
		GO:0009269	response to desiccation
		GO:0030730	sequestering of triacylglycerol
LOLAL	NM_080039	GO:0003704	specific RNA polymerase II transcription factor activity
		GO:0005515	protein binding
		GO:0005634	nucleus
		GO:0006333	chromatin assembly or disassembly
		GO:0006342	chromatin silencing
		GO:0006357	regulation of transcription from RNA polymerase II promoter
		GO:0007530	sex determination
		GO:0019226	transmission of nerve impulse
CG10914	NM_137453		

GO density (7 genes):

Ranking	GO id	Function	Frequency
1	GO:0005515	protein binding	42 %
2	GO:0005634	nucleus	28 %
3	GO:0006357	regulation of transcription from RNA polymerase II promoter	28 %
4	GO:0007268	synaptic transmission	14 %
5	GO:0016568	chromatin modification	14 %
6	GO:0009269	response to desiccation	14 %
7	GO:0008270	zinc ion binding	14 %
8	GO:0005975	carbohydrate metabolic process	14 %
9	GO:0030730	sequestering of triacylglycerol	14 %
10	GO:0000166	nucleotide binding	14 %
11	GO:0006398	histone mRNA 3'-end processing	14 %
12	GO:0030528	transcription regulator activity	14 %
13	GO:0019226	transmission of nerve impulse	14 %
14	GO:0003677	DNA binding	14 %
15	GO:0006333	chromatin assembly or disassembly	14 %
16	GO:0008143	poly(A) binding	14 %
17	GO:0006629	lipid metabolic process	14 %
18	GO:0003704	specific RNA polymerase II transcription factor activity	14 %
19	GO:0007530	sex determination	14 %
20	GO:0003729	mRNA binding	14 %
21	GO:0006342	chromatin silencing	14 %
22	GO:0045892	negative regulation of transcription, DNA-dependent	14 %
23	GO:0009055	electron carrier activity	14 %
24	GO:0003700	transcription factor activity	14 %
25	GO:0005507	copper ion binding	14 %
26	GO:0005737	cytoplasm	14 %
27	GO:0006118	electron transport	14 %

globalDown – chr2R: 17044563 - 17079034

Genomic components: 3 coregulated genes, 5 genes

CHR	Strand	Start	End	RefSeq	Name	Exons	Description
CHR2R	-	17044563	17045583	NM_166461	CG30287	4	CG30287-PA
CHR2R	-	17045831	17046875	NM_206195	CG33226	3	CG33226-PA
CHR2R	-	17047134	17048155	NM_137743	CG30283	3	CG30283-PA
CHR2R	+	17055150	17067009	NM_057411	Egfr	5	Epidermal growth factor receptor CG10079-PB,
CHR2R	-	17067827	17079034	NM_137744	CG10440	5	CG10440-PA

Cluster size: 34472 nucleotides

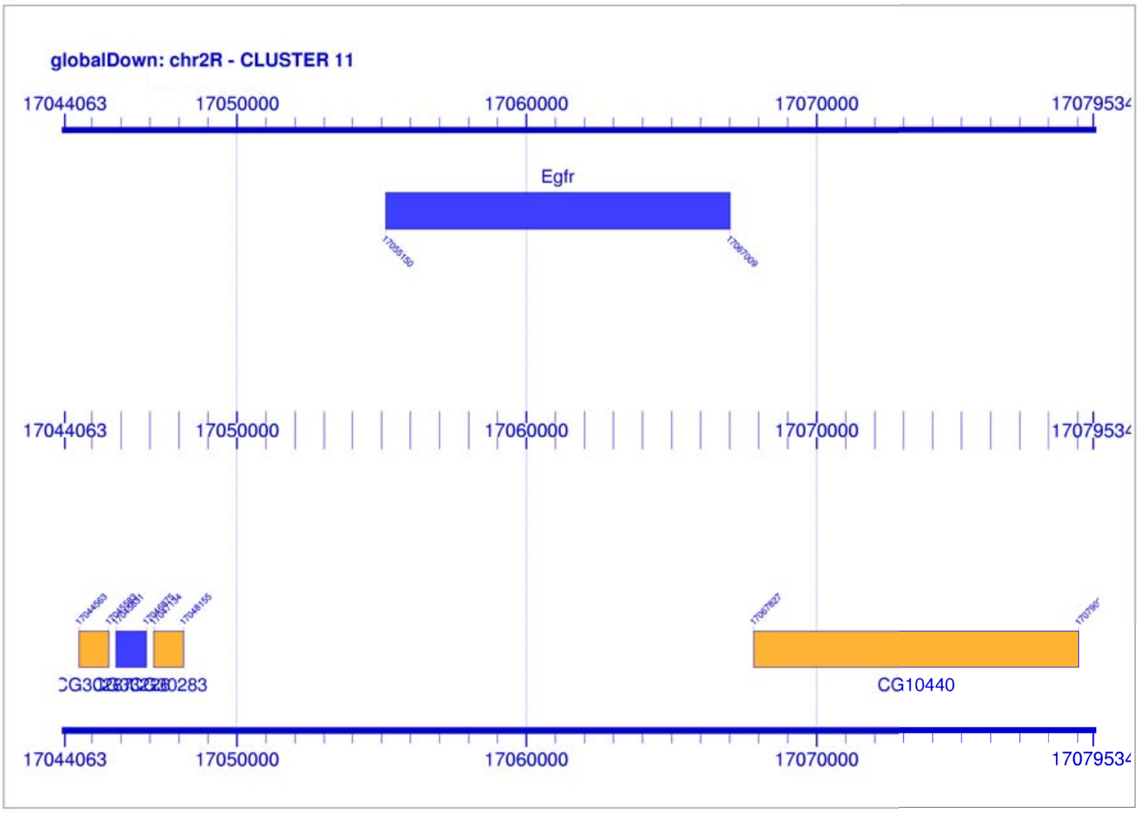

globalDown – cluster 11

Genomic components:

Name	RefSeq	Function	
CG30287	NM_166461	GO:0006508	proteolysis
		GO:0006520	amino acid metabolic process
		GO:0006521	regulation of amino acid metabolic process
		GO:0008233	peptidase activity
		GO:0008236	serine-type peptidase activity
		GO:0008652	amino acid biosynthetic process
		GO:0019538	protein metabolic process
CG33226	NM_206195	GO:0004252	serine-type endopeptidase activity
		GO:0005575	cellular_component
		GO:0006508	proteolysis
CG30283	NM_137743	GO:0006508	proteolysis
Egfr	NM_057411	GO:0000086	G2/M transition of mitotic cell cycle
		GO:0000578	embryonic axis specification
		GO:0001654	eye development
		GO:0001709	cell fate determination
		GO:0001742	oenocyte differentiation
		GO:0001745	compound eye morphogenesis
		GO:0002009	morphogenesis of an epithelium
		GO:0003676	nucleic acid binding
		GO:0004623	phospholipase A2 activity
		GO:0004713	protein-tyrosine kinase activity
		GO:0004888	transmembrane receptor activity
		GO:0005006	epidermal growth factor receptor activity
		GO:0005524	ATP binding
		GO:0005622	intracellular
		GO:0005886	plasma membrane
		GO:0005887	integral to plasma membrane
		GO:0006468	protein amino acid phosphorylation
		GO:0006644	phospholipid metabolic process
		GO:0006916	anti-apoptosis
		GO:0006952	defense response
		GO:0007173	epidermal growth factor receptor signaling pathway
		GO:0007298	border follicle cell migration (sensu Insecta)
		GO:0007310	oocyte dorsal/ventral axis determination
		GO:0007314	oocyte anterior/posterior axis determination
		GO:0007346	regulation of progression through mitotic cell cycle
		GO:0007350	blastoderm segmentation
		GO:0007367	segment polarity determination
		GO:0007369	gastrulation
		GO:0007390	germ-band shortening
		GO:0007391	dorsal closure
		GO:0007420	brain development
		GO:0007421	stomatogastric nervous system development
		GO:0007422	peripheral nervous system development
		GO:0007424	open tracheal system development
		GO:0007431	salivary gland development
		GO:0007443	Malpighian tubule morphogenesis
		GO:0007444	imaginal disc development
		GO:0007447	imaginal disc pattern formation
		GO:0007458	progression of morphogenetic furrow during compound eye morp
		GO:0007469	antennal development
		GO:0007472	wing disc morphogenesis
		GO:0007473	wing disc proximal/distal pattern formation
		GO:0007474	imaginal disc-derived wing vein specification
		GO:0007476	imaginal disc-derived wing morphogenesis

Name	RefSeq	Function	
Egfr	NM_057411	GO:0007477	notum development
		GO:0007479	leg disc proximal/distal pattern formation
		GO:0008071	maternal determination of dorsal/ventral axis, follicular ep
		GO:0008270	zinc ion binding
		GO:0008313	gurken receptor activity
		GO:0008314	gurken receptor signaling pathway
		GO:0008586	imaginal disc-derived wing vein morphogenesis
		GO:0009880	embryonic pattern specification
		GO:0009950	dorsal/ventral axis specification
CG10440	NM_137744	GO:0005249	voltage-gated potassium channel activity
		GO:0005515	protein binding
		GO:0006813	potassium ion transport
		GO:0008076	voltage-gated potassium channel complex

GO density (5 genes):

Ranking	GO id	Function	Frequency
1	GO:0006508	proteolysis	60 %
2	GO:0016318	ommatidial rotation	20 %
3	GO:0006644	phospholipid metabolic process	20 %
4	GO:0007314	oocyte anterior/posterior axis determination	20 %
5	GO:0005006	epidermal growth factor receptor activity	20 %
6	GO:0019904	melanosome localization	20 %
7	GO:0007469	antennal development	20 %
8	GO:0007424	open tracheal system development	20 %
9	GO:0009950	dorsal/ventral axis specification	20 %
10	GO:0007421	stomatogastric nervous system development	20 %
11	GO:0046845	branched duct epithelial cell fate determination, open trach	20 %
12	GO:0006916	anti-apoptosis	20 %
13	GO:0005249	voltage-gated potassium channel activity	20 %
14	GO:0001745	compound eye morphogenesis	20 %
15	GO:0004252	serine-type endopeptidase activity	20 %
16	GO:0045165	cell fate commitment	20 %
17	GO:0030031	cell projection biogenesis	20 %
18	GO:0008270	zinc ion binding	20 %
19	GO:0007477	notum development	20 %
20	GO:0009880	embryonic pattern specification	20 %
21	GO:0007472	wing disc morphogenesis	20 %
22	GO:0007458	progression of morphogenetic furrow during compound eye morp	20 %
23	GO:0048112	oocyte anterior/posterior axis determination (sensu Insecta)	20 %
24	GO:0009993	oogenesis (sensu Insecta)	20 %
25	GO:0007310	oocyte dorsal/ventral axis determination	20 %
26	GO:0045468	regulation of R8 spacing	20 %
27	GO:0016042	lipid catabolic process	20 %
28	GO:0008652	amino acid biosynthetic process	20 %
29	GO:0009952	anterior/posterior pattern formation	20 %
30	GO:0042694	muscle cell fate specification	20 %
31	GO:0007476	imaginal disc-derived wing morphogenesis	20 %
32	GO:0006468	protein amino acid phosphorylation	20 %
33	GO:0001654	eye development	20 %
34	GO:0035277	spiracle morphogenesis, open tracheal system	20 %
35	GO:0007369	gastrulation	20 %
36	GO:0000578	embryonic axis specification	20 %
37	GO:0007367	segment polarity determination	20 %
38	GO:0004888	transmembrane receptor activity	20 %
39	GO:0007173	epidermal growth factor receptor signaling pathway	20 %
40	GO:0005515	protein binding	20 %
41	GO:0007431	salivary gland development	20 %
42	GO:0004713	protein-tyrosine kinase activity	20 %
43	GO:0008236	serine-type peptidase activity	20 %
44	GO:0048140	male germ-line cyst encapsulation	20 %
45	GO:0007350	blastoderm segmentation	20 %
46	GO:0007346	regulation of progression through mitotic cell cycle	20 %
47	GO:0002009	morphogenesis of an epithelium	20 %
48	GO:0035225	determination of genital disc primordium	20 %
49	GO:0000086	G2/M transition of mitotic cell cycle	20 %

Ranking	GO id	Function	Frequency
50	GO:0006952	defense response	20 %
51	GO:0003676	nucleic acid binding	20 %
52	GO:0008313	gurken receptor activity	20 %
53	GO:0030381	eggshell pattern formation (sensu Insecta)	20 %
54	GO:0007479	leg disc proximal/distal pattern formation	20 %
55	GO:0035310	notum cell fate specification	20 %
56	GO:0005575	cellular_component	20 %
57	GO:0001709	cell fate determination	20 %
58	GO:0008076	voltage-gated potassium channel complex	20 %
59	GO:0005886	plasma membrane	20 %
60	GO:0016330	second mitotic wave during compound eye morphogenesis	20 %
61	GO:0016333	morphogenesis of follicular epithelium	20 %
62	GO:0008586	imaginal disc-derived wing vein morphogenesis	20 %
63	GO:0045610	regulation of hemocyte differentiation	20 %
64	GO:0006521	regulation of amino acid metabolic process	20 %
65	GO:0046673	negative regulation of compound eye retinal cell programmed	20 %
66	GO:0005524	ATP binding	20 %
67	GO:0007422	peripheral nervous system development	20 %
68	GO:0008071	maternal determination of dorsal/ventral axis, follicular ep	20 %
69	GO:0019538	protein metabolic process	20 %
70	GO:0007447	imaginal disc pattern formation	20 %
71	GO:0004623	phospholipase A2 activity	20 %
72	GO:0007473	wing disc proximal/distal pattern formation	20 %
73	GO:0035309	wing and notum subfield formation	20 %
74	GO:0005622	intracellular	20 %
75	GO:0006520	amino acid metabolic process	20 %
76	GO:0048139	female germ-line cyst encapsulation	20 %
77	GO:0009953	dorsal/ventral pattern formation	20 %
78	GO:0006813	potassium ion transport	20 %
79	GO:0008314	gurken receptor signaling pathway	20 %
80	GO:0016203	muscle attachment	20 %
81	GO:0007298	border follicle cell migration (sensu Insecta)	20 %
82	GO:0035202	sac formation, open tracheal system	20 %
83	GO:0001742	oenocyte differentiation	20 %
84	GO:0007390	germ-band shortening	20 %
85	GO:0005887	integral to plasma membrane	20 %
86	GO:0007420	brain development	20 %
87	GO:0045466	R7 cell differentiation	20 %
88	GO:0007391	dorsal closure	20 %
89	GO:0008233	peptidase activity	20 %
90	GO:0007443	Malpighian tubule morphogenesis	20 %
91	GO:0007474	imaginal disc-derived wing vein specification	20 %
92	GO:0007444	imaginal disc development	20 %

globalDown – chr3R: 4541955 - 4550434

Genomic components: 3 coregulated genes, 4 genes

CHR	Strand	Start	End	RefSeq	Name	Exons	Description
CHR3R	+	4541955	4543783	NM_141561	CG8116	3	CG8116-PA
CHR3R	+	4543943	4547042	NM_169236	CG11760	2	CG11760-PA, isoform A
CHR3R	+	4547092	4548776	NM_141563	CG8136	2	CG8136-PA, isoform A
CHR3R	+	4548945	4550434	NM_141564	CG8145	4	CG8145-PA

Cluster size: 8480 nucleotides

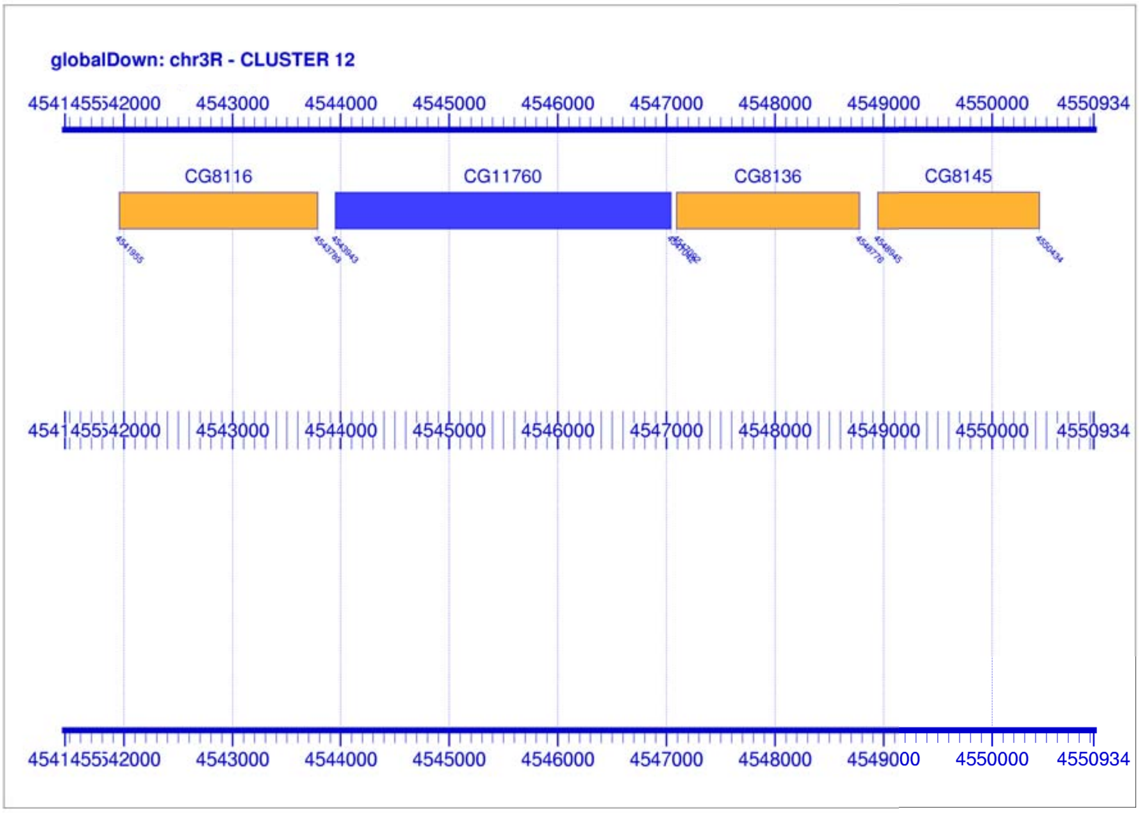

globalDown – cluster 12

Genomic components:

NAME	RefSeq	Function	
CG8116	NM_141561		
CG11760	NM_169236		
CG8136	NM_141563		
CG8145	NM_141564	GO:0003676	nucleic acid binding
		GO:0005634	nucleus
		GO:0006139	nucleobase, nucleoside, nucleotide and nucleic acid metaboli
		GO:0006357	regulation of transcription from RNA polymerase II promoter
		GO:0006366	transcription from RNA polymerase II promoter
		GO:0008270	zinc ion binding
		GO:0008283	cell proliferation
		GO:0030528	transcription regulator activity

GO density (4 genes):

RANKING	GO id	Function	Frequency
1	GO:0006366	transcription from RNA polymerase II promoter	25 %
2	GO:0005634	nucleus	25 %
3	GO:0008270	zinc ion binding	25 %
4	GO:0008283	cell proliferation	25 %
5	GO:0006357	regulation of transcription from RNA polymerase II promoter	25 %
6	GO:0006139	nucleobase, nucleoside, nucleotide and nucleic acid metaboli	25 %
7	GO:0003676	nucleic acid binding	25 %
8	GO:0030528	transcription regulator activity	25 %

globalDown – chr3R: 6682512 - 6719252

Genomic components: 4 coregulated genes, 13 genes

CHR	Strand	Start	End	RefSeq	Name	Exons	Description
CHR3R	+	6682512	6683794	NM_141770	CG4570	1	CG4570-PA
CHR3R	-	6683943	6685525	NM_141771	CG14694	5	CG14694-PA
CHR3R	-	6686505	6688335	NM_141772	CG6574	5	CG6574-PA
CHR3R	-	6689070	6694379	NM_141773	SelR	4	SelR CG6584-PA, isoform A
CHR3R	+	6694433	6695686	NM_079585	Tsp86D	2	Tetraspanin 86D CG4591-PA
CHR3R	-	6696627	6698102	NM_079586	Fdh	3	Formaldehyde dehydrogenase CG6598-PA
CHR3R	+	6698761	6701308	NM_141774	CG4596	2	CG4596-PA
CHR3R	+	6702105	6703986	NM_079587	Sodh-2	6	Sorbitol dehydrogenase-2 CG4649-PA
CHR3R	-	6704125	6704995	NM_141775	CG14695	1	CG14695-PA
CHR3R	-	6705287	6707888	NM_141777	CG6608	3	CG6608-PA, isoform A
CHR3R	-	6708255	6710556	NM_141778	CG14696	4	CG14696-PA
CHR3R	-	6713383	6714835	NM_079588	Adk3	2	Adenylate kinase-3 CG6612-PA, isoform A
CHR3R	-	6715489	6719252	NM_141779	CG6621	8	CG6621-PA

Cluster size: 36741 nucleotides

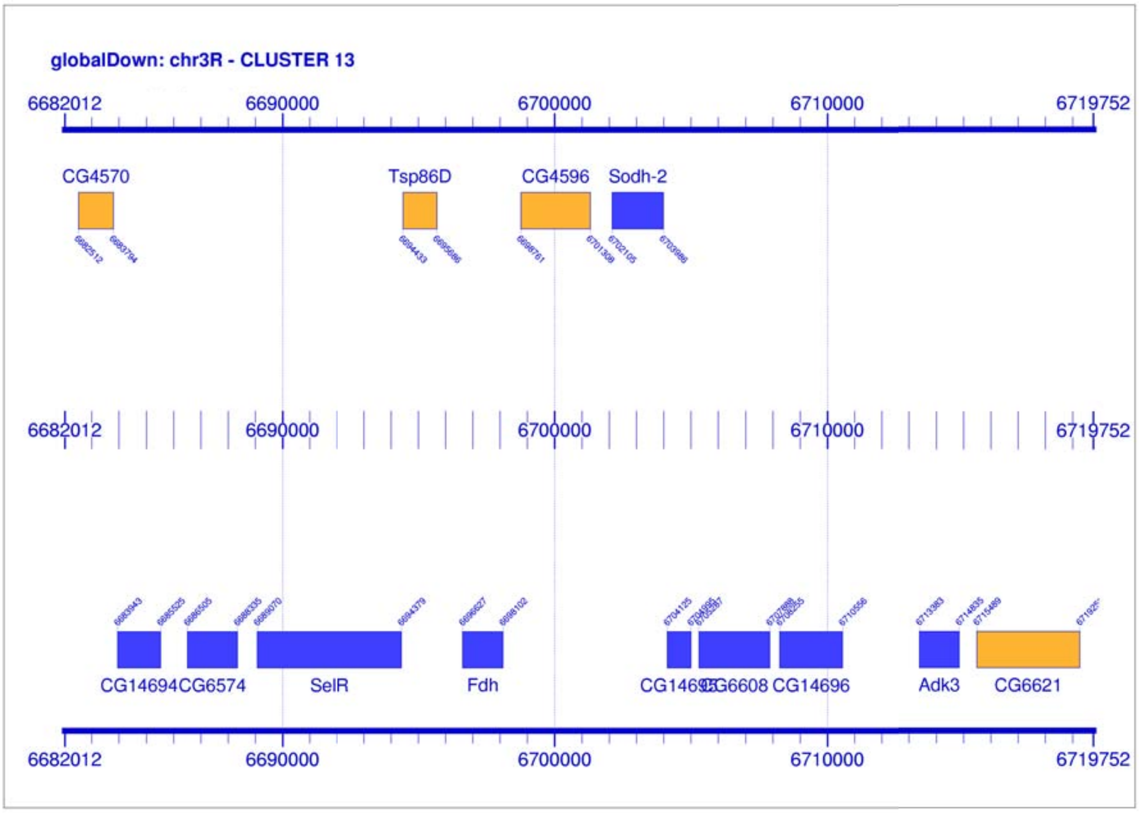

globalDown – cluster 13

Genomic components:

NAME	RefSeq	Function	
CG4570	NM_141770	GO:0003677	DNA binding
		GO:0004803	transposase activity
		GO:0005634	nucleus
		GO:0015074	DNA integration
CG14694	NM_141771	GO:0005542	folic acid binding
		GO:0006732	coenzyme metabolic process
		GO:0006810	transport
		GO:0008518	reduced folate carrier activity
		GO:0016020	membrane
		GO:0051189	prosthetic group metabolic process
CG6574	NM_141772	GO:0005542	folic acid binding
		GO:0006732	coenzyme metabolic process
		GO:0006810	transport
		GO:0008518	reduced folate carrier activity
		GO:0016020	membrane
		GO:0051189	prosthetic group metabolic process
SelR	NM_141773	GO:0000318	protein-methionine-R-oxide reductase activity
		GO:0006800	oxygen and reactive oxygen species metabolic process
		GO:0006952	defense response
		GO:0008270	zinc ion binding
		GO:0018206	peptidyl-methionine modification
Tsp86D	NM_079585	GO:0005102	receptor binding
		GO:0007165	signal transduction
		GO:0016021	integral to membrane
		GO:0016337	cell-cell adhesion
Fdh	NM_079586	GO:0004022	alcohol dehydrogenase activity
		GO:0004327	formaldehyde dehydrogenase (glutathione) activity
		GO:0004552	octanol dehydrogenase activity
		GO:0005975	carbohydrate metabolic process
		GO:0008270	zinc ion binding
CG4596	NM_141774		
Sodh-2	NM_079587	GO:0003939	L-iditol 2-dehydrogenase activity
		GO:0005975	carbohydrate metabolic process
		GO:0008270	zinc ion binding
CG14695	NM_141775		
CG6608	NM_141777	GO:0005386	carrier activity
		GO:0005488	binding
		GO:0005740	mitochondrial envelope
		GO:0005743	mitochondrial inner membrane
		GO:0006812	cation transport
CG14696	NM_141778		
Adk3	NM_079588	GO:0004017	adenylate kinase activity
		GO:0005524	ATP binding
		GO:0005759	mitochondrial matrix
		GO:0006144	purine base metabolic process
		GO:0006172	ADP biosynthetic process
		GO:0046899	nucleoside triphosphate adenylate kinase activity
CG6621	NM_141779	GO:0005488	binding

GO density (13 genes):

Ranking	GO id	Function	Frequency
1	GO:0008270	zinc ion binding	23 %
2	GO:0051189	prosthetic group metabolic process	15 %
3	GO:0006810	transport	15 %
4	GO:0005975	carbohydrate metabolic process	15 %
5	GO:0006732	coenzyme metabolic process	15 %
6	GO:0008518	reduced folate carrier activity	15 %
7	GO:0005488	binding	15 %
8	GO:0005542	folic acid binding	15 %
9	GO:0016020	membrane	15 %
10	GO:0006800	oxygen and reactive oxygen species metabolic process	7 %
11	GO:0004552	octanol dehydrogenase activity	7 %
12	GO:0005634	nucleus	7 %
13	GO:0004327	formaldehyde dehydrogenase (glutathione) activity	7 %
14	GO:0016021	integral to membrane	7 %
15	GO:0006144	purine base metabolic process	7 %
16	GO:0015074	DNA integration	7 %
17	GO:0004022	alcohol dehydrogenase activity	7 %
18	GO:0006812	cation transport	7 %
19	GO:0005740	mitochondrial envelope	7 %
20	GO:0005102	receptor binding	7 %
21	GO:0018206	peptidyl-methionine modification	7 %
22	GO:0006952	defense response	7 %
23	GO:0046899	nucleoside triphosphate adenylate kinase activity	7 %
24	GO:0004803	transposase activity	7 %
25	GO:0000318	protein-methionine-R-oxide reductase activity	7 %
26	GO:0005524	ATP binding	7 %
27	GO:0003677	DNA binding	7 %
28	GO:0005759	mitochondrial matrix	7 %
29	GO:0005386	carrier activity	7 %
30	GO:0005743	mitochondrial inner membrane	7 %
31	GO:0007165	signal transduction	7 %
32	GO:0016337	cell-cell adhesion	7 %
33	GO:0003939	L-iditol 2-dehydrogenase activity	7 %
34	GO:0004017	adenylate kinase activity	7 %
35	GO:0006172	ADP biosynthetic process	7 %

globalDown – chr3R: 7042334 - 7065836

Genomic components: 3 coregulated genes, 13 genes

CHR	Strand	Start	End	RefSeq	Name	Exons	Description
CHR3R	+	7042334	7043620	NM_141793	CG4820	3	CG4820-PA
CHR3R	-	7043619	7045766	NM_141794	CG6689	5	CG6689-PA
CHR3R	-	7045989	7047361	NM_141795	CG6693	1	CG6693-PA
CHR3R	+	7047617	7050897	NM_079592	RpL3	6	Ribosomal protein L3 CG4863-PA, isoform A
CHR3R	+	7051241	7052312	NM_141796	scpr-C	2	SCP-containing protein C CG5106-PA
CHR3R	+	7052485	7053958	NM_141797	CG17726	2	CG17726-PA
CHR3R	-	7053964	7056290	NM_141798	CG10703	3	CG10703-PA
CHR3R	-	7056338	7057578	NM_141799	CG17187	1	CG17187-PA
CHR3R	+	7057738	7058138	NM_141800	CG14701	3	CG14701-PA
CHR3R	-	7058150	7060750	NM_141801	CG17184	5	CG17184-PA, isoform A
CHR3R	+	7061104	7061787	NM_141802	CG17721	2	CG17721-PA
CHR3R	+	7062007	7063346	NM_169382	CG31441	3	CG31441-PA
CHR3R	+	7063536	7065836	NM_141803	CG31388	6	CG31388-PA

Cluster size: 23503 nucleotides

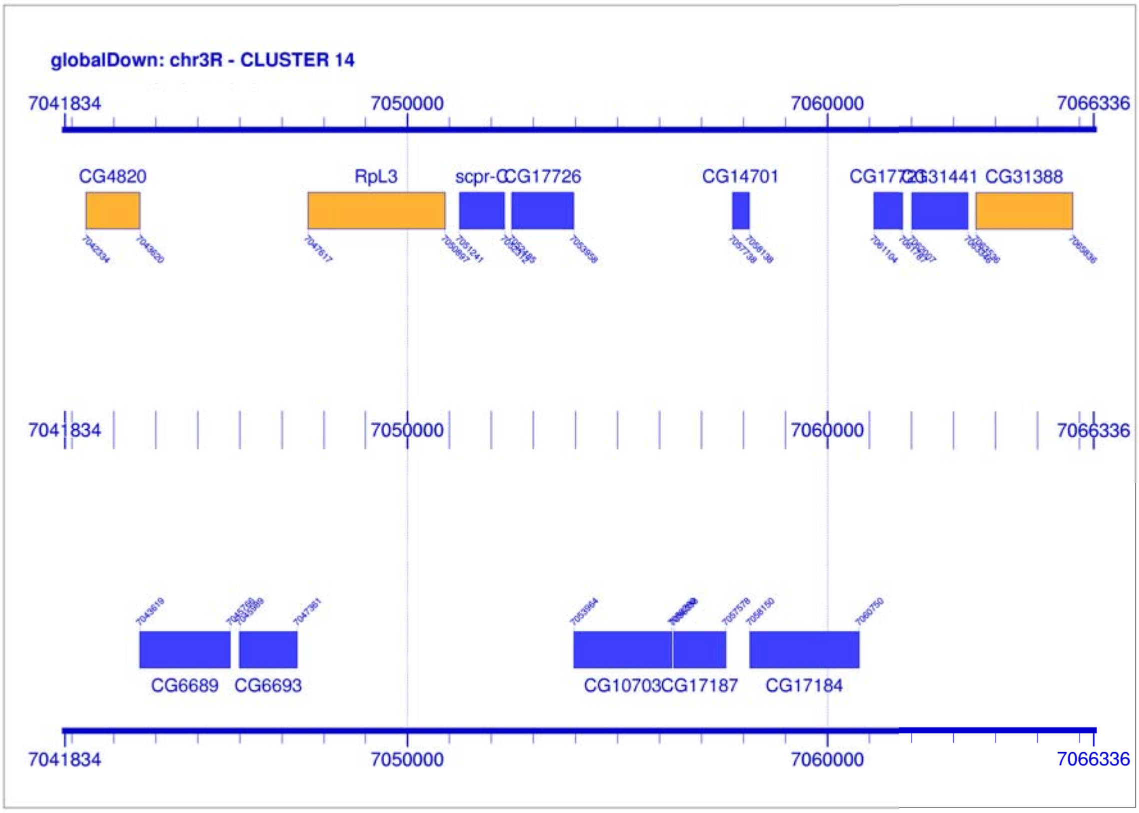

globalDown – cluster 14

Genomic components:

Name	RefSeq	Function	
CG4820	NM_141793	GO:0003676	nucleic acid binding
		GO:0005634	nucleus
		GO:0008270	zinc ion binding
CG6689	NM_141794	GO:0003676	nucleic acid binding
		GO:0005634	nucleus
		GO:0006139	nucleobase, nucleoside, nucleotide and nucleic acid metaboli
		GO:0006357	regulation of transcription from RNA polymerase II promoter
		GO:0006366	transcription from RNA polymerase II promoter
		GO:0008270	zinc ion binding
		GO:0030528	transcription regulator activity
CG6693	NM_141795	GO:0031072	heat shock protein binding
RpL3	NM_079592	GO:0003676	nucleic acid binding
		GO:0003735	structural constituent of ribosome
		GO:0005830	cytosolic ribosome (sensu Eukaryota)
		GO:0005842	cytosolic large ribosomal subunit (sensu Eukaryota)
		GO:0006412	translation
scpr-C	NM_141796		
CG17726	NM_141797		
CG10703	NM_141798		
CG17187	NM_141799	GO:0000166	nucleotide binding
		GO:0006457	protein folding
		GO:0031072	heat shock protein binding
		GO:0051082	unfolded protein binding
CG14701	NM_141800	GO:0003674	molecular_function
		GO:0005575	cellular_component
		GO:0008150	biological_process
CG17184	NM_141801	GO:0005083	small GTPase regulator activity
		GO:0006928	cell motility
		GO:0007010	cytoskeleton organization and biogenesis
CG17721	NM_141802		
CG31441	NM_169382	GO:0003676	nucleic acid binding
		GO:0005634	nucleus
		GO:0006357	regulation of transcription from RNA polymerase II promoter
		GO:0008270	zinc ion binding
		GO:0008283	cell proliferation
		GO:0030528	transcription regulator activity
CG31388	NM_141803	GO:0003676	nucleic acid binding
		GO:0005634	nucleus
		GO:0008270	zinc ion binding

GO density (13 genes):

RANKING	GO id	Function	Frequency
1	GO:0003676	nucleic acid binding	38 %
2	GO:0005634	nucleus	30 %
3	GO:0008270	zinc ion binding	30 %
4	GO:0006357	regulation of transcription from RNA polymerase II promoter	15 %
5	GO:0031072	heat shock protein binding	15 %
6	GO:0030528	transcription regulator activity	15 %
7	GO:0005083	small GTPase regulator activity	7 %
8	GO:0006928	cell motility	7 %
9	GO:0006366	transcription from RNA polymerase II promoter	7 %
10	GO:0008283	cell proliferation	7 %
11	GO:0007010	cytoskeleton organization and biogenesis	7 %
12	GO:0006139	nucleobase, nucleoside, nucleotide and nucleic acid metaboli	7 %
13	GO:0005830	cytosolic ribosome (sensu Eukaryota)	7 %
14	GO:0051082	unfolded protein binding	7 %
15	GO:0005575	cellular_component	7 %
16	GO:0000166	nucleotide binding	7 %
17	GO:0003674	molecular_function	7 %
18	GO:0008150	biological_process	7 %
19	GO:0006412	translation	7 %
20	GO:0006457	protein folding	7 %
21	GO:0003735	structural constituent of ribosome	7 %
22	GO:0005842	cytosolic large ribosomal subunit (sensu Eukaryota)	7 %

globalDown – chr3R: 19503079 - 19520376

Genomic components: 4 coregulated genes, 8 genes

CHR	Strand	Start	End	RefSeq	Name	Exons	Description
CHR3R	+	19503079	19504143	NM_142908	CG13822	3	CG13822-PA
CHR3R	+	19504612	19505596	NM_142909	CG10157	4	CG10157-PA
CHR3R	+	19505766	19508047	NM_079739	eIF-3p66	4	Eukaryotic initiation factor 3 p66 subunit
CHR3R	+	19508224	19509605	NM_142910	CG16710	3	CG16710-PA
CHR3R	+	19510105	19511596	NM_144395	CG18754	3	CG18754-PA
CHR3R	+	19511991	19513869	NM_142911	CG16705	4	CG16705-PA
CHR3R	-	19514098	19514596	NM_142912	CG13819	2	CG13819-PA
CHR3R	-	19514728	19520376	NM_142913	CG10254	9	CG10254-PA, isoform A

Cluster size: 17298 nucleotides

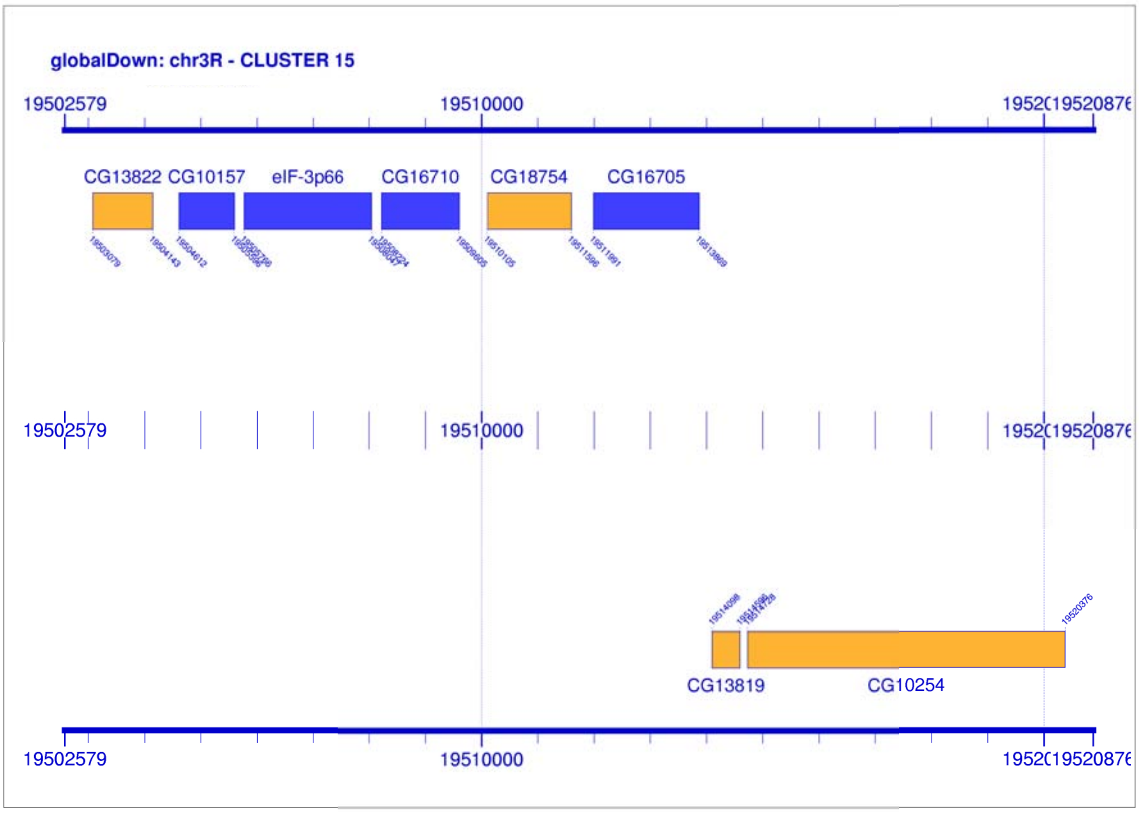

globalDown – cluster 15

Genomic components:

NAME	RefSeq	Function	
CG13822	NM_142908		
CG10157	NM_142909		
EIF-3P66	NM_079739	GO:0003743	translation initiation factor activity
		GO:0005829	cytosol
		GO:0005852	eukaryotic translation initiation factor 3 complex
		GO:0006412	translation
		GO:0006413	translational initiation
CG16710	NM_142910	GO:0004295	trypsin activity
		GO:0006508	proteolysis
CG18754	NM_144395	GO:0004295	trypsin activity
		GO:0006508	proteolysis
CG16705	NM_142911	GO:0004295	trypsin activity
		GO:0006508	proteolysis
		GO:0006521	regulation of amino acid metabolic process
		GO:0008439	monophenol monooxygenase activator activity
		GO:0008652	amino acid biosynthetic process
CG13819	NM_142912		
CG10254	NM_142913	GO:0004842	ubiquitin-protein ligase activity
		GO:0004857	enzyme inhibitor activity
		GO:0006508	proteolysis
		GO:0006512	ubiquitin cycle
		GO:0006916	anti-apoptosis
		GO:0016874	ligase activity

GO density (8 genes):

RANKING	GO id	Function	Frequency
1	GO:0006508	proteolysis	50 %
2	GO:0004295	trypsin activity	37 %
3	GO:0006916	anti-apoptosis	12 %
4	GO:0005829	cytosol	12 %
5	GO:0008652	amino acid biosynthetic process	12 %
6	GO:0004857	enzyme inhibitor activity	12 %
7	GO:0016874	ligase activity	12 %
8	GO:0008439	monophenol monooxygenase activator activity	12 %
9	GO:0005852	eukaryotic translation initiation factor 3 complex	12 %
10	GO:0004842	ubiquitin-protein ligase activity	12 %
11	GO:0006512	ubiquitin cycle	12 %
12	GO:0006521	regulation of amino acid metabolic process	12 %
13	GO:0006412	translation	12 %
14	GO:0006413	translational initiation	12 %
15	GO:0003743	translation initiation factor activity	12 %

globalDown – chr3R: 21849758 - 21866583

Genomic components: 4 coregulated genes, 5 genes

CHR	Strand	Start	End	RefSeq	Name	Exons	Description
CHR3R	-	21849758	21850215	NM_079786	m4	1	E(spl) region transcript m4 CG6099-PA
CHR3R	-	21854572	21855457	NM_079787	HLHm5	1	E(spl) region transcript m5 CG6096-PA
CHR3R	+	21858632	21859769	NM_079788	m6	1	E(spl) region transcript m6 CG8354-PA
CHR3R	+	21862759	21863482	NM_080505	HLHm7	1	E(spl) region transcript m7 CG8361-PA
CHR3R	+	21866045	21866583	NM_079789	E(spl)	1	Enhancer of split CG8365-PA

Cluster size: 16826 nucleotides

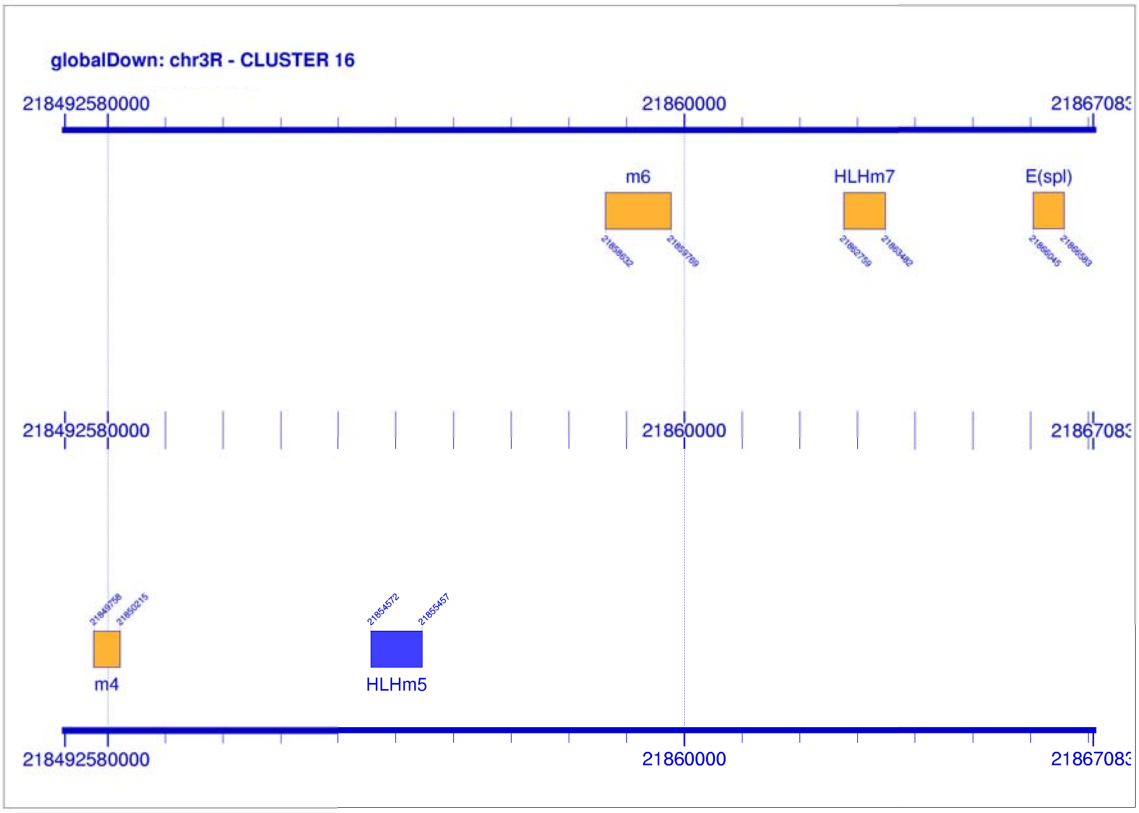

globalDown – cluster 16

Genomic components:

NAME	RefSeq	Function	
M4	NM_079786	GO:0001708	cell fate specification
		GO:0005634	nucleus
		GO:0007219	Notch signaling pathway
		GO:0007423	sensory organ development
HLHM5	NM_079787	GO:0003677	DNA binding
		GO:0003700	transcription factor activity
		GO:0005634	nucleus
		GO:0006357	regulation of transcription from RNA polymerase II promoter
		GO:0007398	ectoderm development
		GO:0007399	nervous system development
		GO:0008283	cell proliferation
		GO:0016481	negative regulation of transcription
		GO:0016566	specific transcriptional repressor activity
		GO:0045165	cell fate commitment
M6	NM_079788		
HLHM7	NM_080505	GO:0003700	transcription factor activity
		GO:0003704	specific RNA polymerase II transcription factor activity
		GO:0005634	nucleus
		GO:0006357	regulation of transcription from RNA polymerase II promoter
		GO:0007398	ectoderm development
		GO:0007399	nervous system development
		GO:0008283	cell proliferation
E(SPL)	NM_079789	GO:0000122	negative regulation of transcription from RNA polymerase II
		GO:0003677	DNA binding
		GO:0003700	transcription factor activity
		GO:0005634	nucleus
		GO:0007173	epidermal growth factor receptor signaling pathway
		GO:0007219	Notch signaling pathway
		GO:0007398	ectoderm development
		GO:0007422	peripheral nervous system development
		GO:0007498	mesoderm development
		GO:0008283	cell proliferation
		GO:0008587	imaginal disc-derived wing margin morphogenesis
		GO:0016360	sensory organ precursor cell fate determination
		GO:0016564	transcriptional repressor activity
		GO:0016566	specific transcriptional repressor activity
		GO:0045165	cell fate commitment
		GO:0045468	regulation of R8 spacing

GO density (5 genes):

Ranking	GO id	Function	Frequency
1	GO:0005634	nucleus	80 %
2	GO:0008283	cell proliferation	60 %
3	GO:0007398	ectoderm development	60 %
4	GO:0003700	transcription factor activity	60 %
5	GO:0045165	cell fate commitment	40 %
6	GO:0006357	regulation of transcription from RNA polymerase II promoter	40 %
7	GO:0016566	specific transcriptional repressor activity	40 %
8	GO:0003677	DNA binding	40 %
9	GO:0007399	nervous system development	40 %
10	GO:0007219	Notch signaling pathway	40 %
11	GO:0007423	sensory organ development	20 %
12	GO:0008587	imaginal disc-derived wing margin morphogenesis	20 %
13	GO:0045468	regulation of R8 spacing	20 %
14	GO:0016481	negative regulation of transcription	20 %
15	GO:0003704	specific RNA polymerase II transcription factor activity	20 %
16	GO:0007173	epidermal growth factor receptor signaling pathway	20 %
17	GO:0016360	sensory organ precursor cell fate determination	20 %
18	GO:0007498	mesoderm development	20 %
19	GO:0000122	negative regulation of transcription from RNA polymerase II	20 %
20	GO:0007422	peripheral nervous system development	20 %
21	GO:0016564	transcriptional repressor activity	20 %
22	GO:0001708	cell fate specification	20 %

globalDown – chrX: 9893150 - 9927703

Genomic components: 4 coregulated genes, 7 genes

CHR	Strand	Start	End	RefSeq	Name	Exons	Description
CHRX	+	9893150	9893724	NM_132371	CG15314	1	CG15314-PA
CHRX	+	9895019	9895636	NM_132372	CG15313	2	CG15313-PA
CHRX	-	9896168	9897857	NM_078547	Yp2	2	Yolk protein 2 CG2979-PA
CHRX	+	9899070	9901037	NM_078548	Yp1	2	Yolk protein 1 CG2985-PA
CHRX	-	9902314	9916087	NM_132373	CG15312	6	CG15312-PA, isoform A
CHRX	+	9906847	9908006	NM_167217	Gr9a	3	Gr9a CG32693-PA
CHRX	-	9917366	9927703	NM_167218	CG32685	11	CG32685-PC

Cluster size: 34554 nucleotides

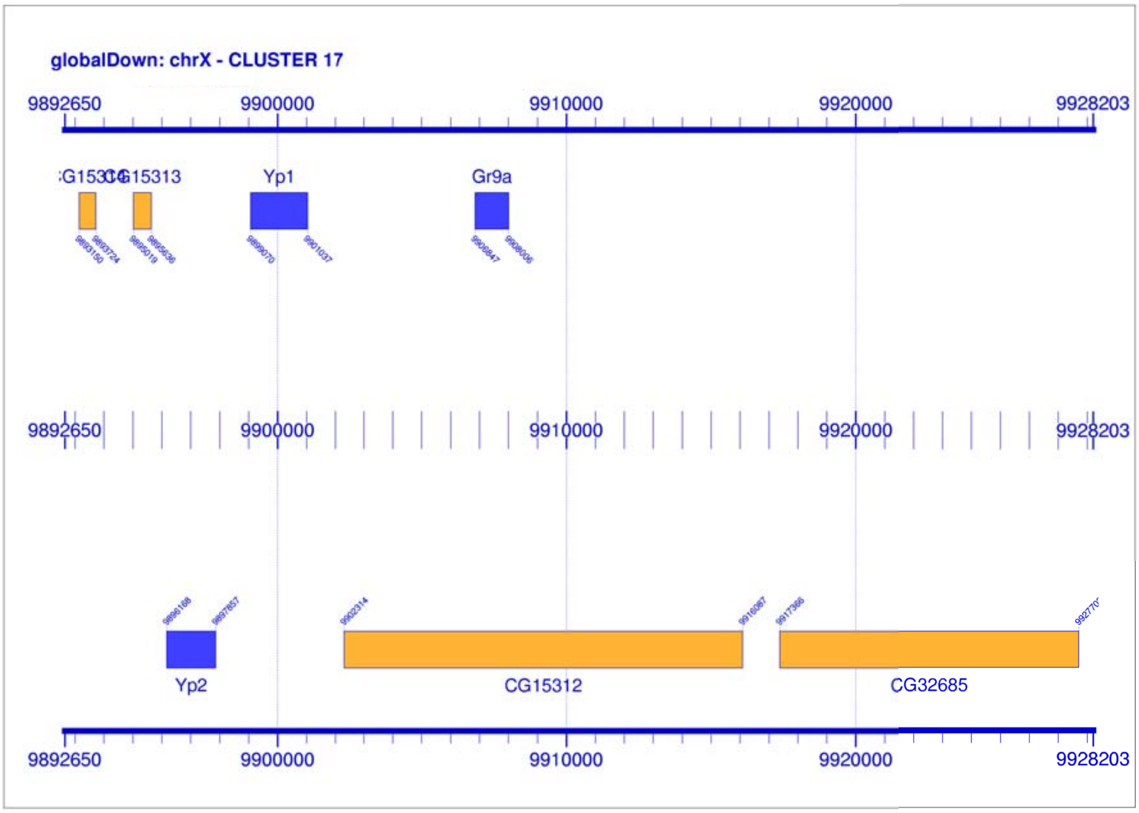

globalDown – cluster 17

Genomic components:

Name	RefSeq	Function	
CG15314	NM_132371		
CG15313	NM_132372		
Yp2	NM_078547	GO:0003824	catalytic activity
		GO:0005198	structural molecule activity
		GO:0006644	phospholipid metabolic process
		GO:0007296	vitellogenesis
		GO:0007548	sex differentiation
		GO:0009993	oogenesis (sensu Insecta)
Yp1	NM_078548	GO:0003824	catalytic activity
		GO:0005198	structural molecule activity
		GO:0006644	phospholipid metabolic process
		GO:0007296	vitellogenesis
		GO:0007548	sex differentiation
CG15312	NM_132373		
Gr9a	NM_167217	GO:0008527	taste receptor activity
		GO:0016021	integral to membrane
		GO:0050909	sensory perception of taste
CG32685	NM_167218		

GO density (7 genes):

Ranking	GO id	Function	Frequency
1	GO:0006644	phospholipid metabolic process	28 %
2	GO:0007548	sex differentiation	28 %
3	GO:0007296	vitellogenesis	28 %
4	GO:0005198	structural molecule activity	28 %
5	GO:0003824	catalytic activity	28 %
6	GO:0008527	taste receptor activity	14 %
7	GO:0016021	integral to membrane	14 %
8	GO:0009993	oogenesis (sensu Insecta)	14 %
9	GO:0050909	sensory perception of taste	14 %

globalDown – chrX: 13572079 - 13610673

Genomic components: 3 coregulated genes, 8 genes

CHR	Strand	Start	End	RefSeq	Name	Exons	Description
CHRX	+	13572079	13573114	NM_132688	CG11134	3	CG11134-PA
CHRX	+	13573680	13575040	NM_132689	CG11151	3	CG11151-PA
CHRX	-	13575591	13578592	NM_080180	l(1)dd4	4	lethal (1) discs degenerate 4 CG10988-PA
CHRX	-	13580604	13589325	NM_132690	CG10990	4	CG10990-PA
CHRX	-	13590097	13590740	NM_167381	CG32625	2	CG32625-PA
CHRX	+	13591113	13592538	NM_132691	Rtc1	3	Rtc1 CG11130-PA
CHRX	+	13593481	13595111	NM_078593	Yp3	3	Yolk protein 3 CG11129-PA
CHRX	+	13596706	13610673	NM_001014740	rdgB	12	retinal degeneration B CG11111-PD, isoform D

Cluster size: 38595 nucleotides

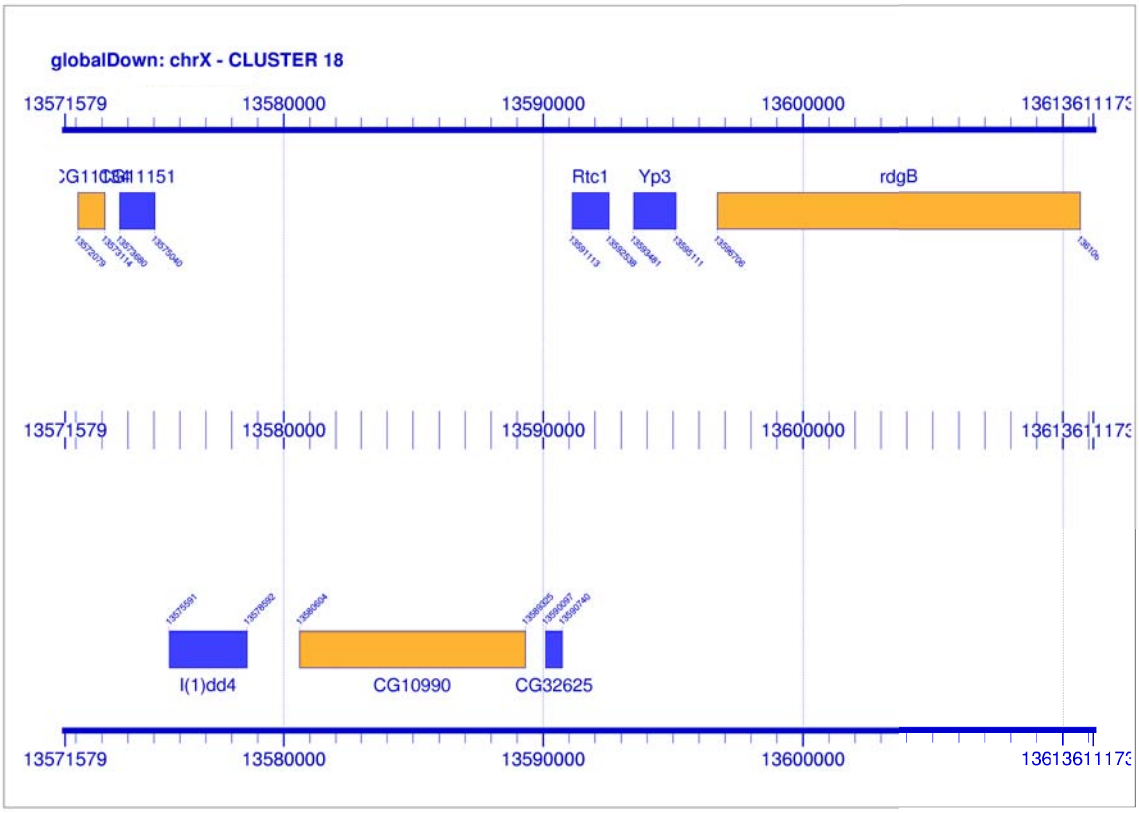

globalDown – cluster 18

Genomic components:

Name	RefSeq	Function	
CG11134	NM_132688	GO:0005975	carbohydrate metabolic process
		GO:0016854	racemase and epimerase activity
CG11151	NM_132689	GO:0004303	estradiol 17-beta-dehydrogenase activity
		GO:0005498	sterol carrier activity
		GO:0007292	female gamete generation
l(1)dd4	NM_080180	GO:0000922	spindle pole
		GO:0005200	structural constituent of cytoskeleton
		GO:0005875	microtubule associated complex
		GO:0006520	amino acid metabolic process
		GO:0007017	microtubule-based process
		GO:0007020	microtubule nucleation
		GO:0007049	cell cycle
		GO:0007126	meiosis
		GO:0007127	meiosis I
		GO:0008017	microtubule binding
		GO:0008275	gamma-tubulin small complex
		GO:0043015	gamma-tubulin binding
		GO:0051322	anaphase
CG10990	NM_132690	GO:0003676	nucleic acid binding
		GO:0003746	translation elongation factor activity
		GO:0006412	translation
		GO:0006915	apoptosis
		GO:0006917	induction of apoptosis
		GO:0008135	translation factor activity, nucleic acid binding
		GO:0019538	protein metabolic process
CG32625	NM_167381	GO:0003674	molecular_function
		GO:0005575	cellular_component
		GO:0008150	biological_process
Rtc1	NM_132691	GO:0000398	nuclear mRNA splicing, via spliceosome
		GO:0003729	mRNA binding
		GO:0003963	RNA-3'-phosphate cyclase activity
		GO:0005634	nucleus
		GO:0016849	phosphorus-oxygen lyase activity
Yp3	NM_078593	GO:0003824	catalytic activity
		GO:0005198	structural molecule activity
		GO:0005524	ATP binding
		GO:0006644	phospholipid metabolic process
		GO:0007296	vitellogenesis
		GO:0007548	sex differentiation
rdgB	NM_001014740	GO:0005388	calcium-transporting ATPase activity
		GO:0005509	calcium ion binding
		GO:0005622	intracellular
		GO:0005886	plasma membrane
		GO:0005887	integral to plasma membrane
		GO:0006629	lipid metabolic process
		GO:0006869	lipid transport
		GO:0007601	visual perception
		GO:0007602	phototransduction
		GO:0007608	sensory perception of smell
		GO:0008525	phosphatidylcholine transporter activity
		GO:0008526	phosphatidylinositol transporter activity
		GO:0016021	integral to membrane
		GO:0016029	subrhabdomeral cisterna
		GO:0016056	rhodopsin mediated signaling
		GO:0016059	deactivation of rhodopsin mediated signaling
		GO:0030384	phosphoinositide metabolic process
		GO:0035091	phosphoinositide binding
		GO:0045494	photoreceptor cell maintenance

GO density (8 genes):

RANKING	GO id	Function	Frequency
1	GO:0007049	cell cycle	12 %
2	GO:0008525	phosphatidylcholine transporter activity	12 %
3	GO:0006644	phospholipid metabolic process	12 %
4	GO:0007126	meiosis	12 %
5	GO:0008275	gamma-tubulin small complex	12 %
6	GO:0005200	structural constituent of cytoskeleton	12 %
7	GO:0005388	calcium-transporting ATPase activity	12 %
8	GO:0005634	nucleus	12 %
9	GO:0007017	microtubule-based process	12 %
10	GO:0016021	integral to membrane	12 %
11	GO:0008526	phosphatidylinositol transporter activity	12 %
12	GO:0007602	phototransduction	12 %
13	GO:0016029	subrhabdomeral cisterna	12 %
14	GO:0007548	sex differentiation	12 %
15	GO:0008017	microtubule binding	12 %
16	GO:0000398	nuclear mRNA splicing, via spliceosome	12 %
17	GO:0007127	meiosis I	12 %
18	GO:0003963	RNA-3'-phosphate cyclase activity	12 %
19	GO:0043015	gamma-tubulin binding	12 %
20	GO:0007296	vitellogenesis	12 %
21	GO:0007292	female gamete generation	12 %
22	GO:0016854	racemase and epimerase activity	12 %
23	GO:0003746	translation elongation factor activity	12 %
24	GO:0030384	phosphoinositide metabolic process	12 %
25	GO:0005198	structural molecule activity	12 %
26	GO:0005509	calcium ion binding	12 %
27	GO:0016849	phosphorus-oxygen lyase activity	12 %
28	GO:0007020	microtubule nucleation	12 %
29	GO:0003676	nucleic acid binding	12 %
30	GO:0008135	translation factor activity, nucleic acid binding	12 %
31	GO:0007608	sensory perception of smell	12 %
32	GO:0005575	cellular_component	12 %
33	GO:0005886	plasma membrane	12 %
34	GO:0005975	carbohydrate metabolic process	12 %
35	GO:0004303	estradiol 17-beta-dehydrogenase activity	12 %
36	GO:0005875	microtubule associated complex	12 %
37	GO:0003674	molecular_function	12 %
38	GO:0051322	anaphase	12 %
39	GO:0016059	deactivation of rhodopsin mediated signaling	12 %
40	GO:0006869	lipid transport	12 %
41	GO:0005524	ATP binding	12 %
42	GO:0000922	spindle pole	12 %
43	GO:0006629	lipid metabolic process	12 %
44	GO:0008150	biological_process	12 %
45	GO:0006412	translation	12 %
46	GO:0019538	protein metabolic process	12 %
47	GO:0006915	apoptosis	12 %
48	GO:0035091	phosphoinositide binding	12 %
49	GO:0045494	photoreceptor cell maintenance	12 %
50	GO:0003824	catalytic activity	12 %
51	GO:0005622	intracellular	12 %
52	GO:0006520	amino acid metabolic process	12 %
53	GO:0003729	mRNA binding	12 %
54	GO:0006917	induction of apoptosis	12 %
55	GO:0005887	integral to plasma membrane	12 %
56	GO:0007601	visual perception	12 %
57	GO:0005498	sterol carrier activity	12 %
58	GO:0016056	rhodopsin mediated signaling	12 %

globalDown – chrX: 17462100 - 17486489

Genomic components: 3 coregulated genes, 6 genes

CHR	Strand	Start	End	RefSeq	Name	Exons	Description
CHRX	-	17462100	17463413	NM_132998	CG8326	2	CG8326-PA
CHRX	-	17463721	17465957	NM_132999	CG8316	4	CG8316-PA
CHRX	+	17466025	17467443	NM_133000	CG5703	4	CG5703-PA
CHRX	-	17467627	17469979	NM_133001	CG8289	3	CG8289-PA
CHRX	+	17470219	17472940	NM_133002	CG5800	2	CG5800-PA
CHRX	-	17473083	17486489	NM_133003	RhoGAPp190	14	RhoGAPp190 CG32555-PB, isoform B

Cluster size: 24390 nucleotides

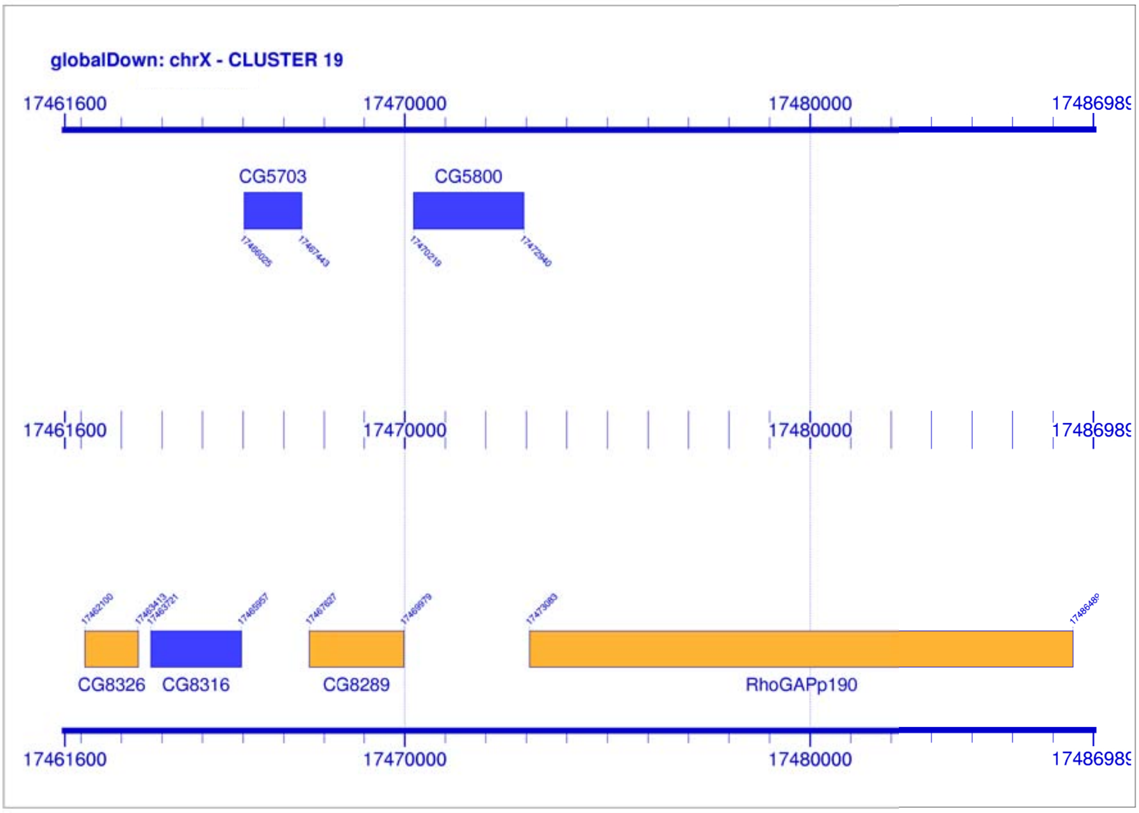

globalDown – cluster 19

Genomic components:

NAME	RefSeq	Function	
CG8326	NM_132998		
CG8316	NM_132999	GO:0003674	molecular_function
		GO:0005575	cellular_component
		GO:0008150	biological_process
CG5703	NM_133000	GO:0003954	NADH dehydrogenase activity
		GO:0005747	mitochondrial respiratory chain complex I
		GO:0006120	mitochondrial electron transport, NADH to ubiquinone
		GO:0008137	NADH dehydrogenase (ubiquinone) activity
CG8289	NM_133001		
CG5800	NM_133002	GO:0003676	nucleic acid binding
		GO:0003724	RNA helicase activity
		GO:0005524	ATP binding
		GO:0006139	nucleobase, nucleoside, nucleotide and nucleic acid metaboli
		GO:0008026	ATP-dependent helicase activity
RhoGAPp190	NM_133003	GO:0005100	Rho GTPase activator activity
		GO:0007242	intracellular signaling cascade
		GO:0007398	ectoderm development
		GO:0016319	mushroom body development
		GO:0019226	transmission of nerve impulse
		GO:0050770	regulation of axonogenesis

GO density (6 genes):

RANKING	GO id	Function	Frequency
1	GO:0050770	regulation of axonogenesis	16 %
2	GO:0019226	transmission of nerve impulse	16 %
3	GO:0007398	ectoderm development	16 %
4	GO:0006139	nucleobase, nucleoside, nucleotide and nucleic acid metaboli	16 %
5	GO:0007242	intracellular signaling cascade	16 %
6	GO:0016319	mushroom body development	16 %
7	GO:0003676	nucleic acid binding	16 %
8	GO:0003724	RNA helicase activity	16 %
9	GO:0005575	cellular_component	16 %
10	GO:0006120	mitochondrial electron transport, NADH to ubiquinone	16 %
11	GO:0003674	molecular_function	16 %
12	GO:0005524	ATP binding	16 %
13	GO:0008150	biological_process	16 %
14	GO:0003954	NADH dehydrogenase activity	16 %
15	GO:0008137	NADH dehydrogenase (ubiquinone) activity	16 %
16	GO:0005747	mitochondrial respiratory chain complex I	16 %
17	GO:0008026	ATP-dependent helicase activity	16 %
18	GO:0005100	Rho GTPase activator activity	16 %
