# Supplementary material for: Identification and Functional Analysis of Healing Regulators in Drosophila
Source: PLoS Genet. 2015 Feb 3;11(2):e1004965. doi: 10.1371/journal.pgen.1004965 (PMC4315591; doi:10.1371/journal.pgen.1004965)
Supplement: S9 Table — Chromosomal clusters of downregulated genes for the W/NW/D comparison (10) are described by their chromosomal location, number of genes, number of co regulated genes, identity of each gene and each gene’s GO Terms. Genes highlighted in orange are those transcriptionally co regulated during healing. (PDF) [file pgen.1004965.s017.pdf]

W/NW/D down - chr2L: 3703579-3712862

Genomic components: 4 coregulated genes, 5 genes

CHR	Strand	Start	End	RefSeq	Name	Exons	Description
CHR2L	+	3703579	3704037	NM_134959	CG2816	3	CG2816-PA
CHR2L	-	3704105	3705462	NM_164549	CG31778	2	CG31778-PA
CHR2L	-	3706544	3707060	NM_164550	CG31777	2	CG31777-PA
CHR2L	-	3707225	3711674	NM_175955	cutlet	12	cutlet CG33122-PA
CHR2L	-	3712155	3712862	NM_164552	CG31955	2	CG31955-PA

Cluster size: 9284 nucleotides

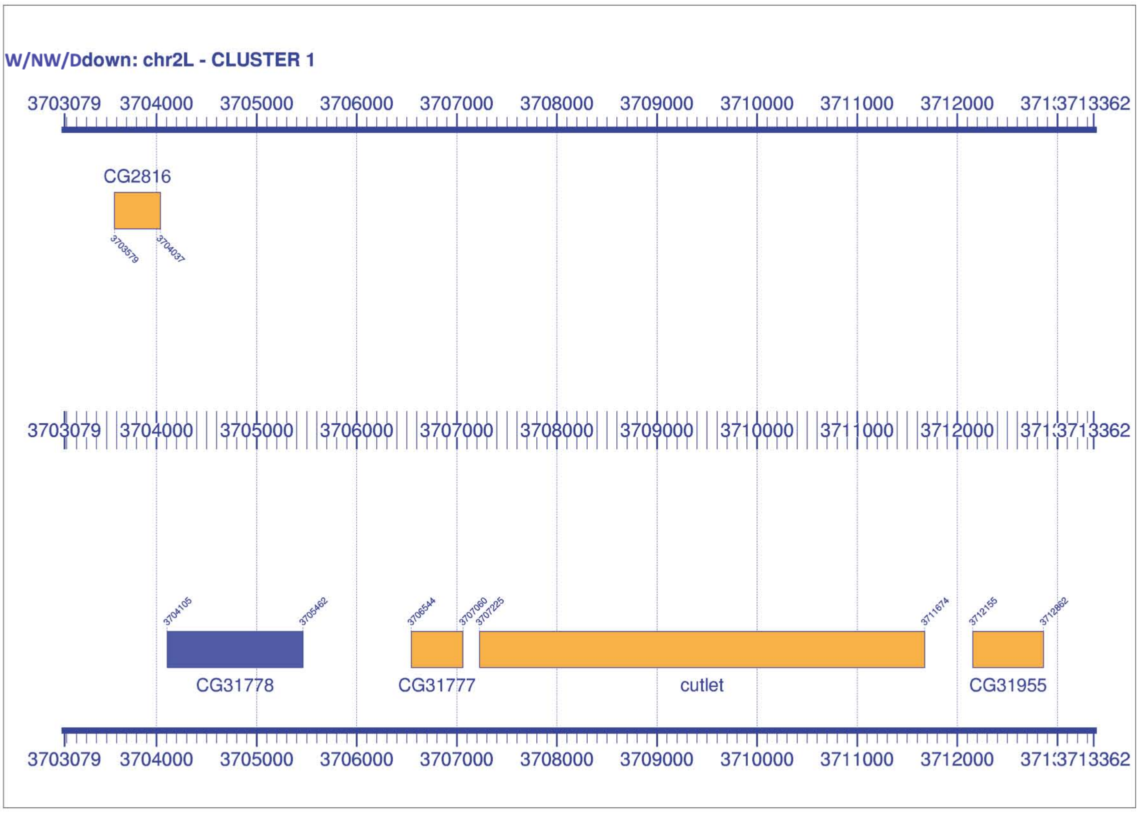

W / NW / D down - cluster 1

Genomic components:

NAME	RefSeq	Function	
CG2816	NM_134959	GO:0004867	serine-type endopeptidase inhibitor activity
CG31778	NM_164549	GO:0004867	serine-type endopeptidase inhibitor activity
CG31777	NM_164550	GO:0004867	serine-type endopeptidase inhibitor activity
CUTLET	NM_175955	GO:0003677	DNA binding
		GO:0004176	ATP-dependent peptidase activity
		GO:0004252	serine-type endopeptidase activity
		GO:0005524	ATP binding
		GO:0005634	nucleus
		GO:0006260	DNA replication
		GO:0006508	proteolysis
		GO:0008283	cell proliferation
		GO:0017111	nucleoside-triphosphatase activity
CG31955	NM_164552		

GO density (5 genes):

RANKING	GO id	Function	Frequency
1	GO:0004867	serine-type endopeptidase inhibitor activity	60 %
2	GO:0004176	ATP-dependent peptidase activity	20 %
3	GO:0017111	nucleoside-triphosphatase activity	20 %
4	GO:0006508	proteolysis	20 %
5	GO:0008283	cell proliferation	20 %
6	GO:0006260	DNA replication	20 %
7	GO:0005524	ATP binding	20 %
8	GO:0003677	DNA binding	20 %
9	GO:0005634	nucleus	20 %
10	GO:0004252	serine-type endopeptidase activity	20 %

W/NW/D down - chr3L: 9308278-9351374

Genomic components: 5 coregulated genes, 16 genes

CHR	Strand	Start	End	RefSeq	Name	Exons	Description
CHR3L	+	9308278	9312286	NM_206304	PGRP-LA	6	Peptidoglycan recognition protein LA CG32042-PE,
CHR3L	+	9314400	9322281	NM_140041	PGRP-LC	4	Peptidoglycan recognition protein LC CG4432-PB,
CHR3L	+	9323557	9325436	NM_140042	PGRP-LF	4	Peptidoglycan recognition protein LF CG4437-PA
CHR3L	-	9324813	9331838	NM_140043	UGP	8	UGP CG4347-PA, isoform A
CHR3L	+	9328282	9329283	NM_168326	CG32040	2	CG32040-PA
CHR3L	+	9332969	9333948	NM_168327	CG32039	3	CG32039-PA
CHR3L	+	9334258	9336616	NM_140044	CG4446	4	CG4446-PA, isoform A
CHR3L	-	9336577	9340887	NM_079268	Klp67A	5	Kinesin-like protein at 67A CG10923-PA
CHR3L	+	9338305	9339187	NM_140045	CG4447	1	CG4447-PA
CHR3L	+	9341272	9344245	NM_140046	CG4452	6	CG4452-PA, isoform A
CHR3L	-	9344145	9344984	NM_079269	Fdxh	3	Ferredoxin CG4205-PA
CHR3L	-	9345665	9346264	NM_079270	Hsp67Bc	1	Heat shock gene 67Bc CG4190-PA
CHR3L	+	9346877	9348916	NM_001031944	Hsp22	3	CG4460-PA, isoform A
CHR3L	+	9346877	9348916	NM_001031945	Hsp67Bb	3	CG4456-PB, isoform B
CHR3L	+	9349375	9350216	NM_140047	CG4461	1	CG4461-PA
CHR3L	-	9350364	9351374	NM_079273	Hsp26	1	Heat shock protein 26 CG4183-PA

Cluster size: 43097 nucleotides

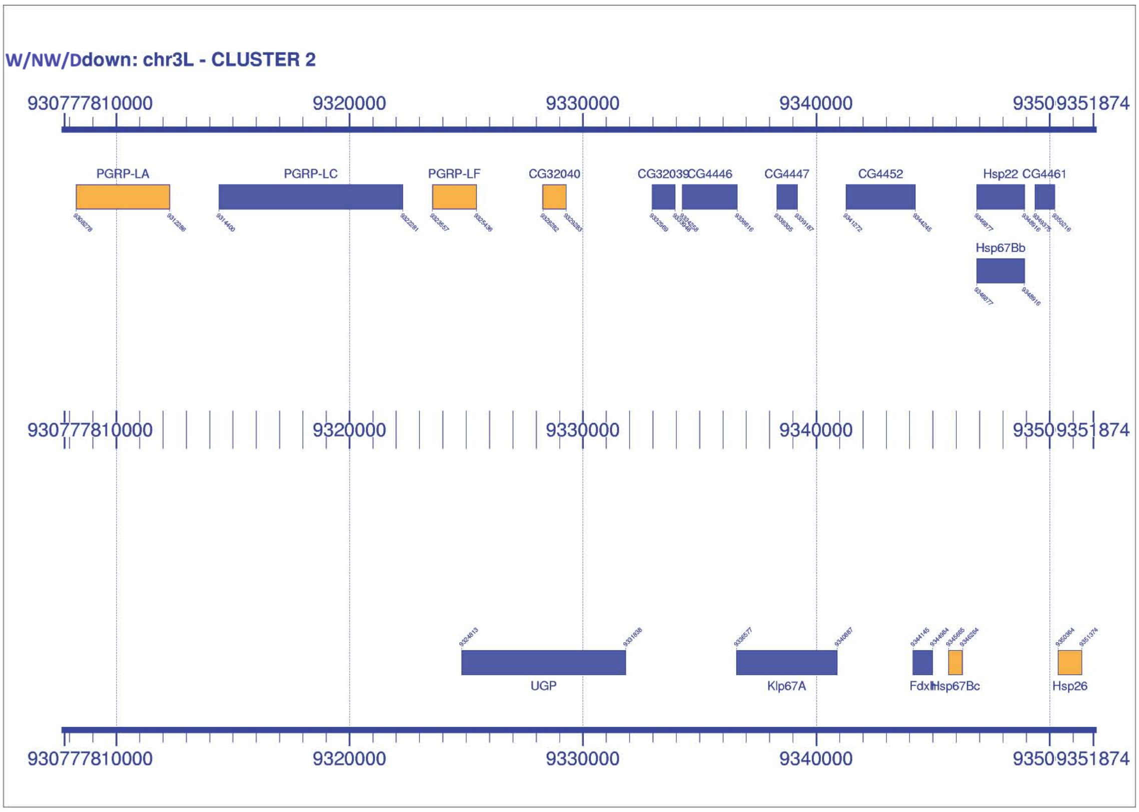

W / NW / D down - cluster 2

Genomic components:

Name	RefSeq	Function	
PGRP-LA	NM_206304	GO:0005515	protein binding
		GO:0005887	integral to plasma membrane
		GO:0006952	defense response
		GO:0006955	immune response
		GO:0008745	N-acetylmuramoyl-L-alanine amidase activity
		GO:0009253	peptidoglycan catabolic process
		GO:0016021	integral to membrane
		GO:0016045	detection of bacterium
		GO:0042834	peptidoglycan binding
		GO:0045087	innate immune response
PGRP-LC	NM_140041	GO:0003700	transcription factor activity
		GO:0005622	intracellular
		GO:0005887	integral to plasma membrane
		GO:0006355	regulation of transcription, DNA-dependent
		GO:0006909	phagocytosis
		GO:0006952	defense response
		GO:0006955	immune response
		GO:0006963	positive regulation of antibacterial peptide biosynthetic pr
		GO:0006964	positive regulation of biosynthetic process of antibacterial
		GO:0008329	pattern recognition receptor activity
		GO:0008368	Gram-negative bacterial binding
		GO:0008745	N-acetylmuramoyl-L-alanine amidase activity
		GO:0009253	peptidoglycan catabolic process
		GO:0009617	response to bacterium
		GO:0016019	peptidoglycan receptor activity
		GO:0016021	integral to membrane
		GO:0016045	detection of bacterium
		GO:0019730	antimicrobial humoral response
		GO:0019731	antibacterial humoral response
		GO:0042834	peptidoglycan binding
		GO:0043565	sequence-specific DNA binding
		GO:0045087	innate immune response
		GO:0046983	protein dimerization activity
		GO:0050829	defense response to Gram-negative bacterium
PGRP-LF	NM_140042	GO:0005515	protein binding
		GO:0005887	integral to plasma membrane
		GO:0006952	defense response
		GO:0008745	N-acetylmuramoyl-L-alanine amidase activity
		GO:0009253	peptidoglycan catabolic process
		GO:0042834	peptidoglycan binding
		GO:0045087	innate immune response

NAME	RefSeq	Function	
UGP	NM_140043	GO:0003983	UTP:glucose-1-phosphate uridylyltransferase activity
		GO:0005976	polysaccharide metabolic process
CG32040	NM_168326	GO:0003674	molecular_function
		GO:0005575	cellular_component
		GO:0008150	biological_process
CG32039	NM_168327	GO:0003674	molecular_function
		GO:0005575	cellular_component
		GO:0008150	biological_process
CG4446	NM_140044	GO:0006732	coenzyme metabolic process
		GO:0008478	pyridoxal kinase activity
		GO:0009111	vitamin catabolic process
		GO:0051189	prosthetic group metabolic process
Klp67A	NM_079268	GO:0000910	cytokinesis
		GO:0003774	motor activity
		GO:0003777	microtubule motor activity
		GO:0005200	structural constituent of cytoskeleton
		GO:0005524	ATP binding
		GO:0005634	nucleus
		GO:0005737	cytoplasm
		GO:0005871	kinesin complex
		GO:0005875	microtubule associated complex
		GO:0006605	protein targeting
		GO:0007018	microtubule-based movement
		GO:0007059	chromosome segregation
		GO:0007060	male meiosis chromosome segregation
		GO:0016346	male meiotic chromosome movement towards spindle pole
		GO:0031134	sister chromatid biorientation
		GO:0046785	microtubule polymerization
		GO:0051225	spindle assembly
		GO:0051226	meiotic spindle assembly
		GO:0051299	centrosome separation
CG4447	NM_140045	GO:0006412	translation
CG4452	NM_140046		
Fdxh	NM_079269	GO:0005506	iron ion binding
		GO:0006118	electron transport
		GO:0006124	ferredoxin metabolic process
		GO:0009055	electron carrier activity
		GO:0016491	oxidoreductase activity
Hsp67Bc	NM_079270	GO:0006457	protein folding
		GO:0006950	response to stress
		GO:0006952	defense response
		GO:0009408	response to heat
Hsp22	NM_1031944	GO:0008340	determination of adult life span
		GO:0009408	response to heat
		GO:0042026	protein refolding
Hsp67Bb	NM_1031945	GO:0009408	response to heat
CG4461	NM_140047	GO:0009408	response to heat
Hsp26	NM_079273	GO:0006457	protein folding
		GO:0006952	defense response
		GO:0008340	determination of adult life span
		GO:0009408	response to heat

GO density (16 genes):

Ranking	GO id	Function	Frequency
1	GO:0006952	defense response	31 %
2	GO:0009408	response to heat	31 %
3	GO:0008745	N-acetylmuramoyl-L-alanine amidase activity	18 %
4	GO:0042834	peptidoglycan binding	18 %
5	GO:0045087	innate immune response	18 %
6	GO:0005887	integral to plasma membrane	18 %
7	GO:0009253	peptidoglycan catabolic process	18 %
8	GO:0006955	immune response	12 %
9	GO:0008340	determination of adult life span	12 %
10	GO:0005575	cellular_component	12 %
11	GO:0016021	integral to membrane	12 %
12	GO:0003674	molecular_function	12 %
13	GO:0016045	detection of bacterium	12 %
14	GO:0008150	biological_process	12 %
15	GO:0006457	protein folding	12 %
16	GO:0005515	protein binding	12 %
17	GO:0007060	male meiosis chromosome segregation	6 %
18	GO:0042026	protein refolding	6 %
19	GO:0046785	microtubule polymerization	6 %
20	GO:0051189	prosthetic group metabolic process	6 %
21	GO:0009111	vitamin catabolic process	6 %
22	GO:0005200	structural constituent of cytoskeleton	6 %
23	GO:0005634	nucleus	6 %
24	GO:0007018	microtubule-based movement	6 %
25	GO:0005871	kinesin complex	6 %
26	GO:0016491	oxidoreductase activity	6 %
27	GO:0043565	sequence-specific DNA binding	6 %
28	GO:0006732	coenzyme metabolic process	6 %
29	GO:0005875	microtubule associated complex	6 %
30	GO:0051226	meiotic spindle assembly	6 %
31	GO:0031134	sister chromatid biorientation	6 %
32	GO:0019730	antimicrobial humoral response	6 %
33	GO:0006950	response to stress	6 %
34	GO:0006355	regulation of transcription, DNA-dependent	6 %
35	GO:0005506	iron ion binding	6 %
36	GO:0050829	defense response to Gram-negative bacterium	6 %
37	GO:0005524	ATP binding	6 %
38	GO:0007059	chromosome segregation	6 %
39	GO:0008329	pattern recognition receptor activity	6 %
40	GO:0006963	positive regulation of antibacterial peptide biosynthetic pr	6 %
41	GO:0009617	response to bacterium	6 %
42	GO:0019731	antibacterial humoral response	6 %
43	GO:0003777	microtubule motor activity	6 %
44	GO:0006412	translation	6 %
45	GO:0006964	positive regulation of biosynthetic process of antibacterial	6 %
46	GO:0006124	ferredoxin metabolic process	6 %
47	GO:0005976	polysaccharide metabolic process	6 %
48	GO:0003983	UTP:glucose-1-phosphate uridylyltransferase activity	6 %
49	GO:0005622	intracellular	6 %
50	GO:0008368	Gram-negative bacterial binding	6 %
51	GO:0000910	cytokinesis	6 %
52	GO:0051225	spindle assembly	6 %
53	GO:0006605	protein targeting	6 %
54	GO:0009055	electron carrier activity	6 %
55	GO:0006909	phagocytosis	6 %
56	GO:0003700	transcription factor activity	6 %
57	GO:0016346	male meiotic chromosome movement towards spindle pole	6 %
58	GO:0051299	centrosome separation	6 %
59	GO:0016019	peptidoglycan receptor activity	6 %
60	GO:0046983	protein dimerization activity	6 %
61	GO:0005737	cytoplasm	6 %
62	GO:0006118	electron transport	6 %
63	GO:0003774	motor activity	6 %
64	GO:0008478	pyridoxal kinase activity	6 %

W/NW/D down - chr3L: 21671604-21676282

Genomic components: 3 coregulated genes, 3 genes

CHR	Strand	Start	End	RefSeq	Name	Exons	Description
CHR3L	-	21671604	21672175	NM_141100	CG14567	1	CG14567-PA
CHR3L	-	21673606	21674107	NM_141101	CG14566	1	CG14566-PA
CHR3L	+	21675379	21676282	NM_141102	CG14572	1	CG14572-PA

Cluster size: 4679 nucleotides

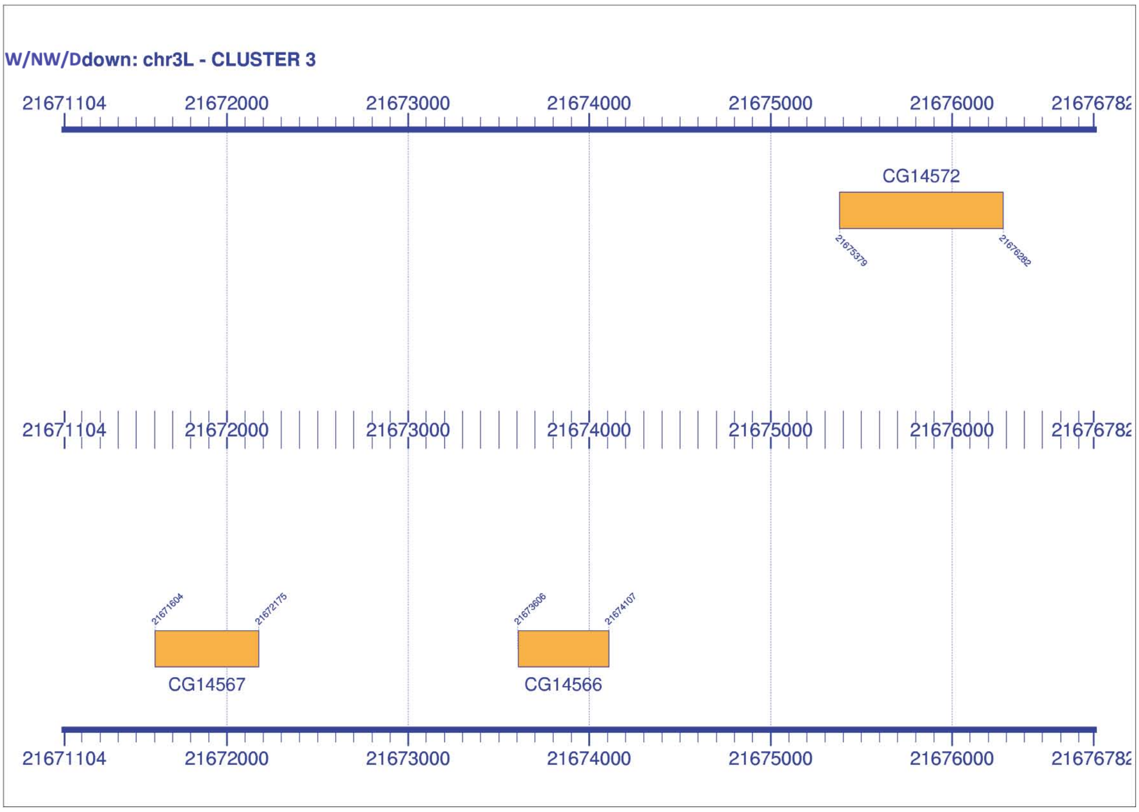

W/NW/D down - cluster 3

Genomic components:

Name	RefSeq	Function
CG14567	NM_141100	
CG14566	NM_141101	
CG14572	NM_141102	

GO density (3 genes):

Ranking	GO id	Function	Frequency

W/NW/D down - chr2R: 13913252-13924698

Genomic components: 4 coregulated genes, 9 genes

CHR	Strand	Start	End	RefSeq	Name	Exons	Description
CHR2R	+	13913252	13914086	NM_137479	GstE1	1	Glutathione S transferase E1 CG5164-PA
CHR2R	+	13914350	13915242	NM_137480	GstE2	1	Glutathione S transferase E2 CG17523-PA
CHR2R	+	13915579	13916331	NM_137481	GstE3	1	Glutathione S transferase E3 CG17524-PA
CHR2R	+	13918137	13918804	NM_137482	GstE4	1	Glutathione S transferase E4 CG17525-PA
CHR2R	+	13919496	13920165	NM_137483	GstE5	1	Glutathione S transferase E5 CG17527-PA
CHR2R	+	13920875	13921628	NM_137484	GstE6	1	Glutathione S transferase E6 CG17530-PA
CHR2R	+	13921797	13922551	NM_137485	GstE7	1	Glutathione S transferase E7 CG17531-PA
CHR2R	+	13922718	13923429	NM_137486	GstE8	1	Glutathione S transferase E8 CG17533-PA
CHR2R	+	13923873	13924698	NM_166279	GstE9	1	Glutathione S transferase E9 CG17534-PA

Cluster size: 11447 nucleotides

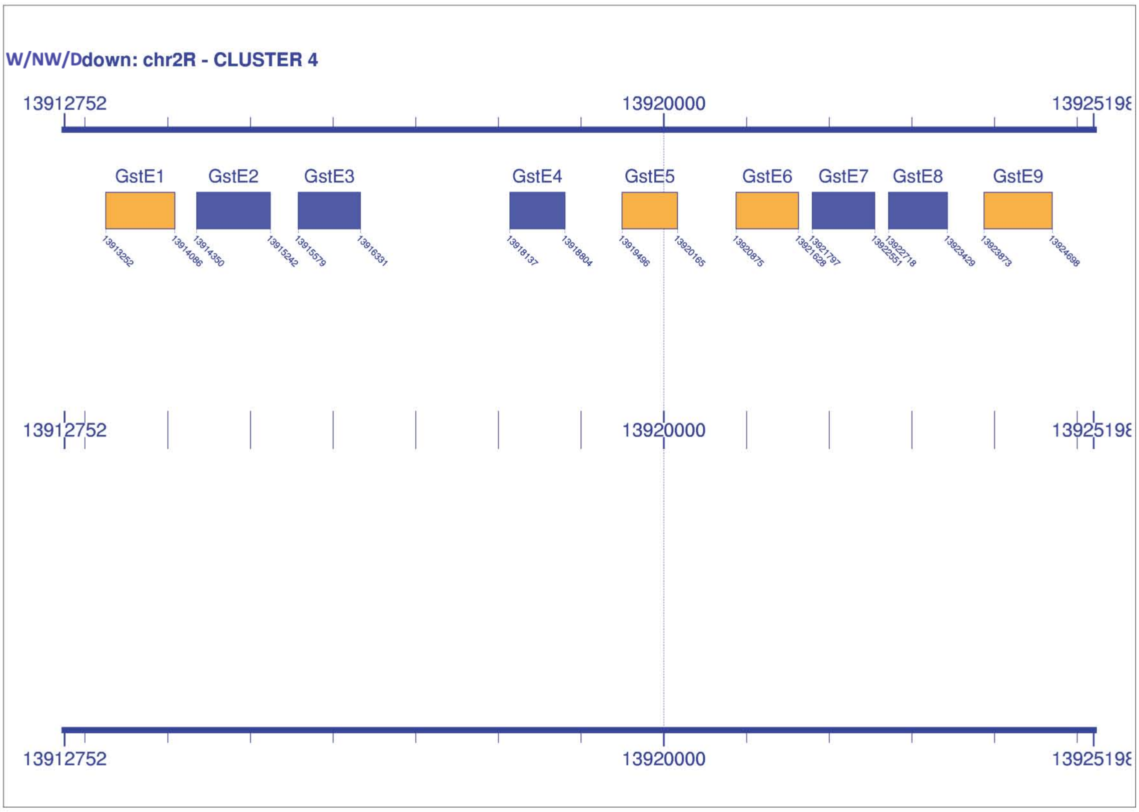

W / NW / D down - cluster 4

Genomic components:

NAME	RefSeq	Function	
GSTE1	NM_137479	GO:0004364	glutathione transferase activity
		GO:0006952	defense response
		GO:0006979	response to oxidative stress
		GO:0009636	response to toxin
GSTE2	NM_137480	GO:0004364	glutathione transferase activity
		GO:0006952	defense response
		GO:0009636	response to toxin
GSTE3	NM_137481	GO:0004364	glutathione transferase activity
		GO:0006800	oxygen and reactive oxygen species metabolic process
		GO:0006952	defense response
		GO:0009636	response to toxin
GSTE4	NM_137482	GO:0004364	glutathione transferase activity
		GO:0006800	oxygen and reactive oxygen species metabolic process
		GO:0006952	defense response
		GO:0009636	response to toxin
GSTE5	NM_137483	GO:0004364	glutathione transferase activity
		GO:0006800	oxygen and reactive oxygen species metabolic process
		GO:0006952	defense response
		GO:0009636	response to toxin
GSTE6	NM_137484	GO:0004364	glutathione transferase activity
		GO:0006800	oxygen and reactive oxygen species metabolic process
		GO:0006952	defense response
		GO:0009636	response to toxin
GSTE7	NM_137485	GO:0004364	glutathione transferase activity
		GO:0006800	oxygen and reactive oxygen species metabolic process
		GO:0006952	defense response
		GO:0009636	response to toxin
GSTE8	NM_137486		
GSTE9	NM_166279	GO:0004364	glutathione transferase activity
		GO:0006952	defense response
		GO:0009636	response to toxin

GO density (9 genes):

RANKING	GO id	Function	Frequency
1	GO:0006952	defense response	88 %
2	GO:0009636	response to toxin	88 %
3	GO:0004364	glutathione transferase activity	88 %
4	GO:0006800	oxygen and reactive oxygen species metabolic process	55 %
5	GO:0006979	response to oxidative stress	11 %

W/NW/D down - chr2R: 18149479-18176395

Genomic components: 6 coregulated genes, 15 genes

CHR	Strand	Start	End	RefSeq	Name	Exons	Description
CHR2R	-	18149479	18151497	NM_137850	Ugt58Fa	4	Ugt58Fa CG4414-PA
CHR2R	-	18152522	18153928	NM_137851	CG2852	3	CG2852-PA, isoform A
CHR2R	-	18154197	18154823	NM_137852	CG30195	2	CG30195-PA
CHR2R	-	18155159	18156035	NM_166540	CG30219	2	CG30219-PA
CHR2R	-	18156699	18157328	NM_137853	CG3746	2	CG3746-PA
CHR2R	+	18158155	18159911	NM_137838	CG3875	6	CG3875-PA
CHR2R	-	18160154	18162109	NM_137854	Cyp6d2	4	Cyp6d2 CG4373-PA
CHR2R	-	18162382	18162956	NM_166541	CG30196	2	CG30196-PA
CHR2R	-	18163130	18164576	NM_137855	CG30217	3	CG30217-PA, isoform A
CHR2R	+	18165486	18166795	NM_137856	CG13510	2	CG13510-PA
CHR2R	+	18167293	18167719	NM_137857	CG13511	2	CG13511-PA
CHR2R	+	18168088	18168967	NM_137858	CG13512	2	CG13512-PA
CHR2R	+	18171269	18172086	NM_137859	CG4250	3	CG4250-PA
CHR2R	+	18174752	18175522	NM_166542	CG30273	3	CG30273-PA
CHR2R	+	18175523	18176395	NM_137860	CG30269	2	CG30269-PA

Cluster size: 26917 nucleotides

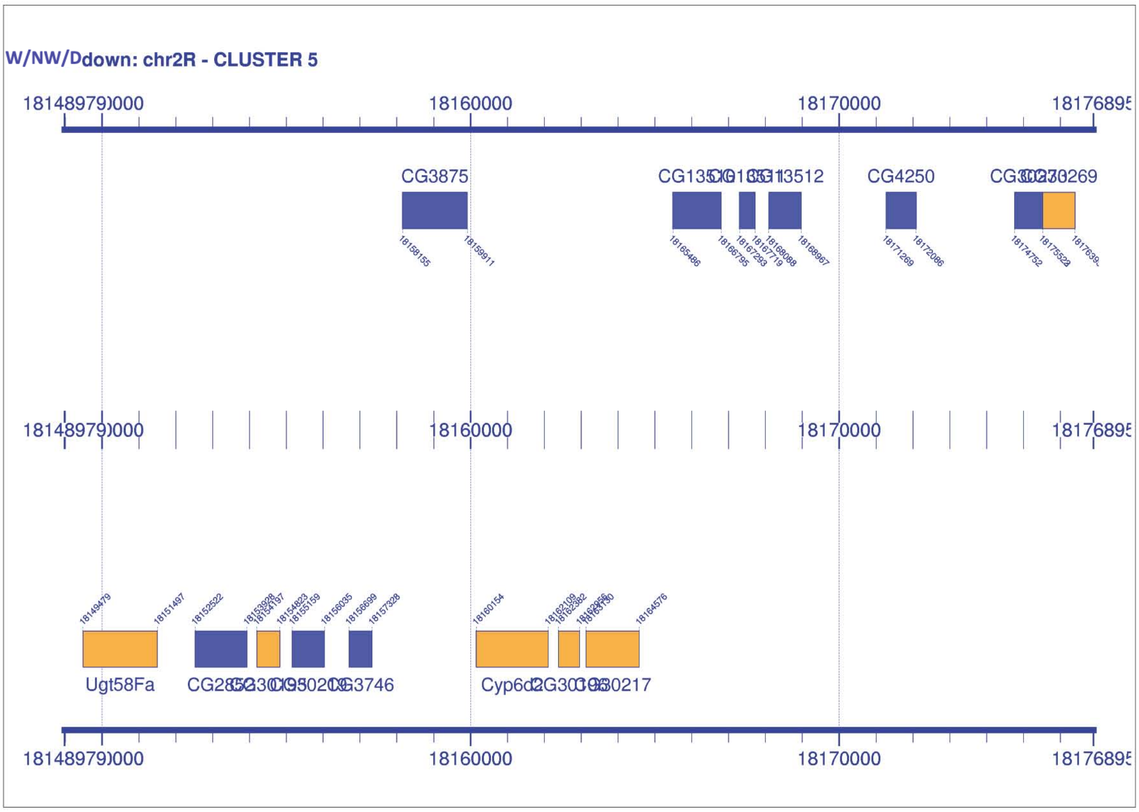

W/NW/D down - cluster 5

Genomic components:

NAME	RefSeq	Function	
UGT58FA	NM_137850	GO:0005976	polysaccharide metabolic process
		GO:0006952	defense response
		GO:0008202	steroid metabolic process
		GO:0009636	response to toxin
		GO:0015020	glucuronosyltransferase activity
CG2852	NM_137851	GO:0003755	peptidyl-prolyl cis-trans isomerase activity
		GO:0006457	protein folding
		GO:0006605	protein targeting
		GO:0006952	defense response
CG30195	NM_137852		
CG30219	NM_166540		
CG3746	NM_137853		
CG3875	NM_137838	GO:0003729	mRNA binding
		GO:0006139	nucleobase, nucleoside, nucleotide and nucleic acid metaboli
		GO:0006744	ubiquinone biosynthetic process
		GO:0006915	apoptosis
		GO:0007283	spermatogenesis
		GO:0008283	cell proliferation
CYP6D2	NM_137854	GO:0004497	monooxygenase activity
		GO:0005506	iron ion binding
		GO:0005792	microsome
		GO:0006118	electron transport
		GO:0008202	steroid metabolic process
		GO:0009055	electron carrier activity
		GO:0016020	membrane
		GO:0016712	oxidoreductase activity, acting on paired donors, with incor
		GO:0020037	heme binding
CG30196	NM_166541		
CG30217	NM_137855		
CG13510	NM_137856		
CG13511	NM_137857		
CG13512	NM_137858		
CG4250	NM_137859		
CG30273	NM_166542		
CG30269	NM_137860		

GO density (15 genes):

Ranking	GO id	Function	Frequency
1	GO:0006952	defense response	13 %
2	GO:0008202	steroid metabolic process	13 %
3	GO:0016712	oxidoreductase activity, acting on paired donors, with incor	6 %
4	GO:0008283	cell proliferation	6 %
5	GO:0009636	response to toxin	6 %
6	GO:0007283	spermatogenesis	6 %
7	GO:0006744	ubiquinone biosynthetic process	6 %
8	GO:0005506	iron ion binding	6 %
9	GO:0003755	peptidyl-prolyl cis-trans isomerase activity	6 %
10	GO:0004497	monooxygenase activity	6 %
11	GO:0005976	polysaccharide metabolic process	6 %
12	GO:0006915	apoptosis	6 %
13	GO:0006139	nucleobase, nucleoside, nucleotide and nucleic acid metaboli	6 %
14	GO:0015020	glucuronosyltransferase activity	6 %
15	GO:0003729	mRNA binding	6 %
16	GO:0005792	microsome	6 %
17	GO:0020037	heme binding	6 %
18	GO:0006457	protein folding	6 %
19	GO:0006605	protein targeting	6 %
20	GO:0009055	electron carrier activity	6 %
21	GO:0006118	electron transport	6 %
22	GO:0016020	membrane	6 %

W/NW/D down - chr2R: 20429104-20454236

Genomic components: 3 coregulated genes, 7 genes

CHR	Strand	Start	End	RefSeq	Name	Exons	Description
CHR2R	-	20429104	20435871	NM_138135	pain	4	painless CG15860-PA
CHR2R	-	20436346	20445052	NM_166697	CG30427	8	CG30427-PC, isoform C
CHR2R	+	20445938	20447369	NM_138137	CG3760	4	CG3760-PB, isoform B
CHR2R	-	20447302	20448070	NM_138138	CG2811	2	CG2811-PA
CHR2R	-	20448224	20449709	NM_001038885	Tina-1	2	Troponin C-akin-1 CG2803-PB, isoform B
CHR2R	-	20450991	20452197	NM_138140	CG15861	3	CG15861-PA
CHR2R	+	20452742	20454236	NM_138141	CG3770	3	CG3770-PA

Cluster size: 25133 nucleotides

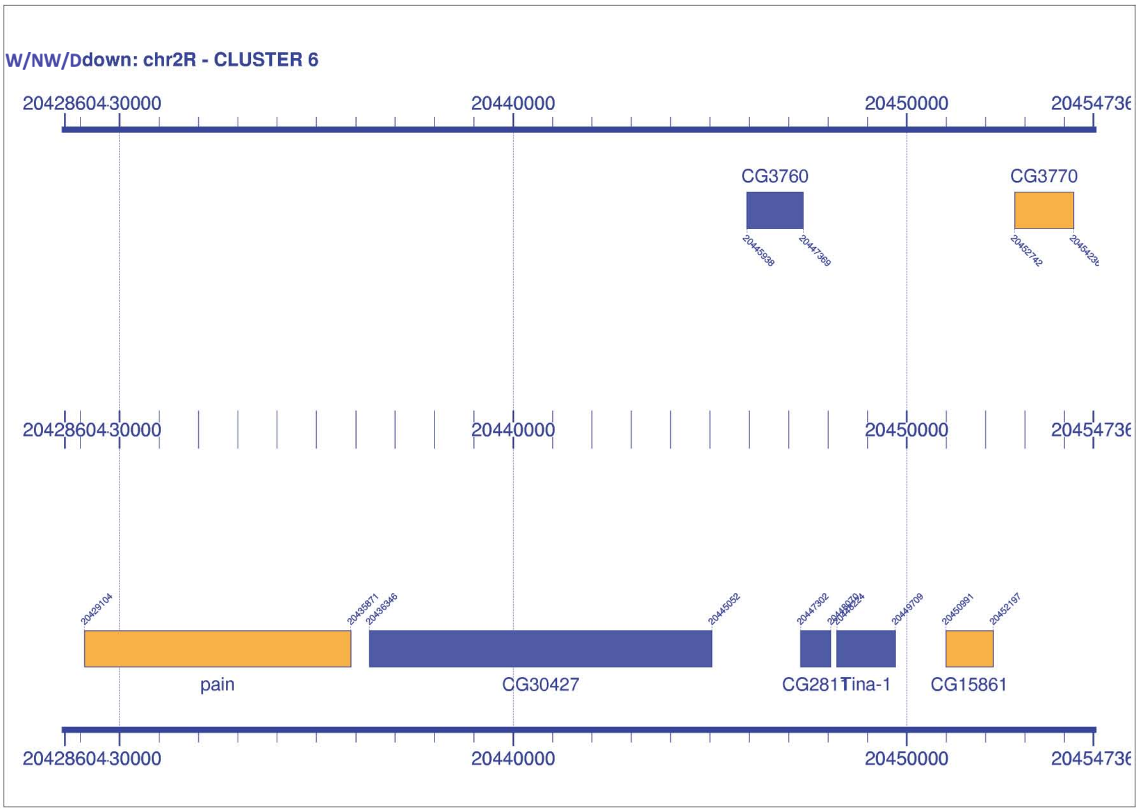

W / NW / D down - cluster 6

Genomic components:

NAME	RefSeq	Function	
PAIN	NM_138135	GO:0005216	ion channel activity
		GO:0005262	calcium channel activity
		GO:0006816	calcium ion transport
		GO:0009408	response to heat
		GO:0009612	response to mechanical stimulus
		GO:0016021	integral to membrane
		GO:0019233	sensory perception of pain
		GO:0050974	detection of mechanical stimulus during sensory perception
CG30427	NM_166697	GO:0016491	oxidoreductase activity
CG3760	NM_138137		
CG2811	NM_138138	GO:0003674	molecular_function
		GO:0005575	cellular_component
		GO:0008150	biological_process
TINA-1	NM_001038885	GO:0005575	cellular_component
		GO:0007507	heart development
		GO:0008307	structural constituent of muscle
CG15861	NM_138140		
CG3770	NM_138141	GO:0007163	establishment and/or maintenance of cell polarity

GO density (7 genes):

RANKING	GO id	Function	Frequency
1	GO:0005575	cellular_component	28 %
2	GO:0005262	calcium channel activity	14 %
3	GO:0016491	oxidoreductase activity	14 %
4	GO:0016021	integral to membrane	14 %
5	GO:0007163	establishment and/or maintenance of cell polarity	14 %
6	GO:0003674	molecular_function	14 %
7	GO:0008150	biological_process	14 %
8	GO:0050974	detection of mechanical stimulus during sensory perception	14 %
9	GO:0005216	ion channel activity	14 %
10	GO:0009408	response to heat	14 %
11	GO:0006816	calcium ion transport	14 %
12	GO:0007507	heart development	14 %
13	GO:0009612	response to mechanical stimulus	14 %
14	GO:0008307	structural constituent of muscle	14 %
15	GO:0019233	sensory perception of pain	14 %

W / NW / D down - chr3R: 6977690-6998386

Genomic components: 4 coregulated genes, 8 genes

CHR	Strand	Start	End	RefSeq	Name	Exons	Description
CHR3R	-	6977690	6979518	NM_144363	Ugt86Di	3	Ugt86Di CG6658-PA
CHR3R	+	6980376	6982235	NM_144368	Ugt86Dc	2	Ugt86Dc CG4739-PA
CHR3R	+	6982821	6985466	NM_144369	Ugt86Da	3	Ugt86Da CG18578-PA
CHR3R	+	6986923	6988866	NM_141786	CG4757	4	CG4757-PA
CHR3R	-	6990144	6991796	NM_144365	Ugt86Dg	2	Ugt86Dg CG17200-PA
CHR3R	-	6992021	6993699	NM_144366	Ugt86De	2	Ugt86De CG6653-PA
CHR3R	-	6994218	6995955	NM_079589	Ugt35b	2	UDP-glycosyltransferase 35b CG6649-PA
CHR3R	-	6996520	6998386	NM_079590	Ugt35a	2	UDP-glycosyltransferase 35a CG6644-PA

Cluster size: 20697 nucleotides

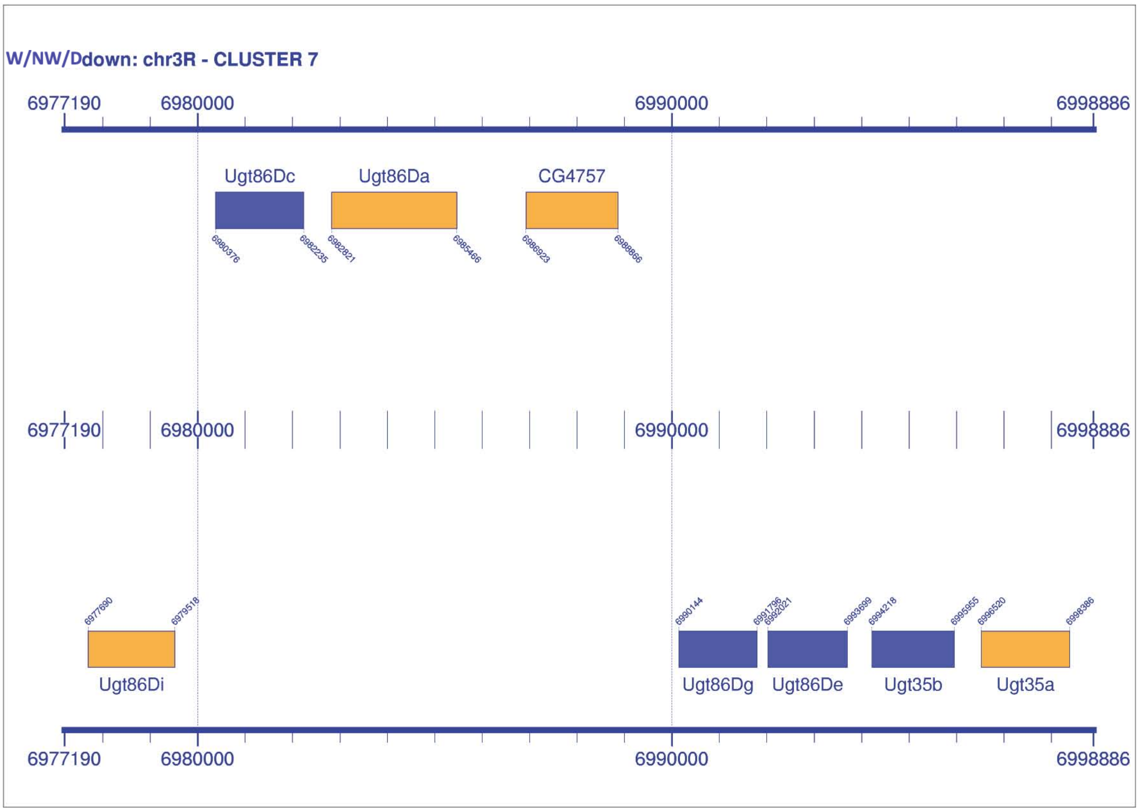

W / NW / D down - cluster 7

Genomic components:

Name	RefSeq	Function	
Ugt86Di	NM_144363	GO:0005976	polysaccharide metabolic process
		GO:0006952	defense response
		GO:0008202	steroid metabolic process
		GO:0009636	response to toxin
		GO:0015020	glucuronosyltransferase activity
Ugt86Dc	NM_144368	GO:0003851	2-hydroxyacylsphingosine 1-beta-galactosyltransferase activi
		GO:0005976	polysaccharide metabolic process
		GO:0006952	defense response
		GO:0008202	steroid metabolic process
		GO:0009636	response to toxin
		GO:0015020	glucuronosyltransferase activity
Ugt86Da	NM_144369	GO:0005976	polysaccharide metabolic process
		GO:0006952	defense response
		GO:0008202	steroid metabolic process
		GO:0009636	response to toxin
		GO:0015020	glucuronosyltransferase activity
CG4757	NM_141786	GO:0004091	carboxylesterase activity
Ugt86Dg	NM_144365	GO:0005976	polysaccharide metabolic process
		GO:0006952	defense response
		GO:0008202	steroid metabolic process
		GO:0009636	response to toxin
		GO:0015020	glucuronosyltransferase activity
Ugt86De	NM_144366	GO:0005976	polysaccharide metabolic process
		GO:0006952	defense response
		GO:0008202	steroid metabolic process
		GO:0009636	response to toxin
		GO:0015020	glucuronosyltransferase activity
Ugt35b	NM_079589	GO:0005976	polysaccharide metabolic process
		GO:0006952	defense response
		GO:0008202	steroid metabolic process
		GO:0009636	response to toxin
		GO:0015020	glucuronosyltransferase activity
Ugt35a	NM_079590	GO:0005976	polysaccharide metabolic process
		GO:0006952	defense response
		GO:0008194	UDP-glycosyltransferase activity
		GO:0008202	steroid metabolic process
		GO:0009636	response to toxin
		GO:0015020	glucuronosyltransferase activity

GO density (8 genes):

Ranking	GO id	Function	Frequency
1	GO:0006952	defense response	87 %
2	GO:0008202	steroid metabolic process	87 %
3	GO:0009636	response to toxin	87 %
4	GO:0005976	polysaccharide metabolic process	87 %
5	GO:0015020	glucuronosyltransferase activity	87 %
6	GO:0004091	carboxylesterase activity	12 %
7	GO:0003851	2-hydroxyacylsphingosine 1-beta-galactosyltransferase activi	12 %
8	GO:0008194	UDP-glycosyltransferase activity	12 %

W/NW/D down - chr3R: 8176608-8199535

Genomic components: 4 coregulated genes, 9 genes

CHR	Strand	Start	End	RefSeq	Name	Exons	Description
CHR3R	-	8176608	8177740	NM_141920	CG10041	2	CG10041-PA
CHR3R	-	8177864	8181961	NM_141921	MBD-R2	6	MBD-R2 CG10042-PA, isoform A
CHR3R	+	8185732	8188975	NM_141922	CG4115	3	CG4115-PA
CHR3R	-	8189388	8190402	NM_141923	Tim17a1	1	Tim17a1 CG10090-PA
CHR3R	-	8190635	8191267	NM_144456	GstD10	1	Glutathione S transferase D10 CG18548-PA
CHR3R	-	8191901	8193286	NM_141924	GstD9	2	Glutathione S transferase D9 CG10091-PA
CHR3R	-	8193268	8194417	NM_001038953	GstD1	1	Glutathione S transferase D1 CG10045-PB, isoform
CHR3R	+	8197719	8198366	NM_080173	GstD2	1	Glutathione S transferase D2 CG4181-PA
CHR3R	+	8198785	8199535	NM_176479	GstD3	1	Glutathione S transferase D3 CG4381-PA

Cluster size: 22928 nucleotides

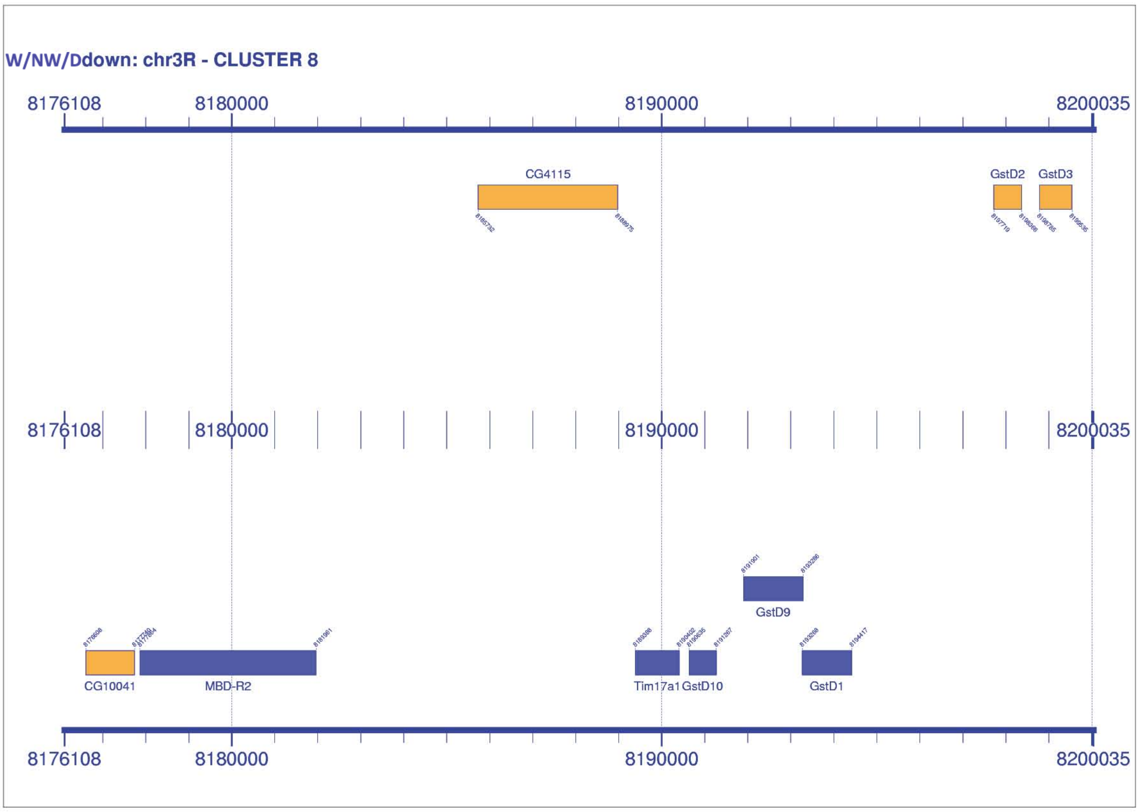

W / NW / D down - cluster 8

Genomic components:

Name	RefSeq	Function	
CG10041	NM_141920	GO:0004252	serine-type endopeptidase activity
		GO:0006508	proteolysis
MBD-R2	NM_141921	GO:0003677	DNA binding
		GO:0005515	protein binding
		GO:0006355	regulation of transcription, DNA-dependent
		GO:0008270	zinc ion binding
CG4115	NM_141922	GO:0005529	sugar binding
Tim17a1	NM_141923	GO:0005744	mitochondrial inner membrane presequence translocase complex
		GO:0006626	protein targeting to mitochondrion
		GO:0008565	protein transporter activity
		GO:0015450	protein translocase activity
		GO:0045039	protein import into mitochondrial inner membrane
GstD10	NM_144456	GO:0004364	glutathione transferase activity
		GO:0006952	defense response
		GO:0009636	response to toxin
GstD9	NM_141924	GO:0004364	glutathione transferase activity
		GO:0006952	defense response
		GO:0009636	response to toxin
GstD1	NM_001038953	GO:0004364	glutathione transferase activity
		GO:0006952	defense response
		GO:0009636	response to toxin
GstD2	NM_080173	GO:0004364	glutathione transferase activity
		GO:0004602	glutathione peroxidase activity
		GO:0006952	defense response
		GO:0009636	response to toxin
GstD3	NM_176479	GO:0004364	glutathione transferase activity
		GO:0005575	cellular_component
		GO:0006952	defense response
		GO:0009636	response to toxin

GO density (9 genes):

Ranking	GO id	Function	Frequency
1	GO:0006952	defense response	55 %
2	GO:0009636	response to toxin	55 %
3	GO:0004364	glutathione transferase activity	55 %
4	GO:0015450	protein translocase activity	11 %
5	GO:0005529	sugar binding	11 %
6	GO:0004252	serine-type endopeptidase activity	11 %
7	GO:0005575	cellular_component	11 %
8	GO:0008270	zinc ion binding	11 %
9	GO:0006355	regulation of transcription, DNA-dependent	11 %
10	GO:0008565	protein transporter activity	11 %
11	GO:0003677	DNA binding	11 %
12	GO:0006508	proteolysis	11 %
13	GO:0006626	protein targeting to mitochondrion	11 %
14	GO:0045039	protein import into mitochondrial inner membrane	11 %
15	GO:0005515	protein binding	11 %
16	GO:0005744	mitochondrial inner membrane presequence translocase complex	11 %
17	GO:0004602	glutathione peroxidase activity	11 %

W/NW/D down - chr3R: 11651470-11679301

Genomic components: 3 coregulated genes, 8 genes

CHR	Strand	Start	End	RefSeq	Name	Exons	Description
CHR3R	+	11651470	11656043	NM_142238	CG4576	8	CG4576-PA
CHR3R	-	11658491	11660147	NM_142240	CG14876	3	CG14876-PA
CHR3R	+	11660487	11661270	NM_142241	Arpc3A	3	Arpc3A CG4560-PB, isoform B
CHR3R	-	11661768	11662819	NM_079980	ND23	3	NADH:ubiquinone reductase 23kD subunit precursor
CHR3R	+	11663158	11670137	NM_057393	spn-E	11	spindle E CG3158-PA
CHR3R	-	11663867	11665193	NM_142242	CG9597	1	CG9597-PA
CHR3R	-	11670187	11679301	NM_169676	CG31150	15	CG31150-PA
CHR3R	+	11672229	11674158	NM_142244	CG4546	3	CG4546-PA

Cluster size: 27832 nucleotides

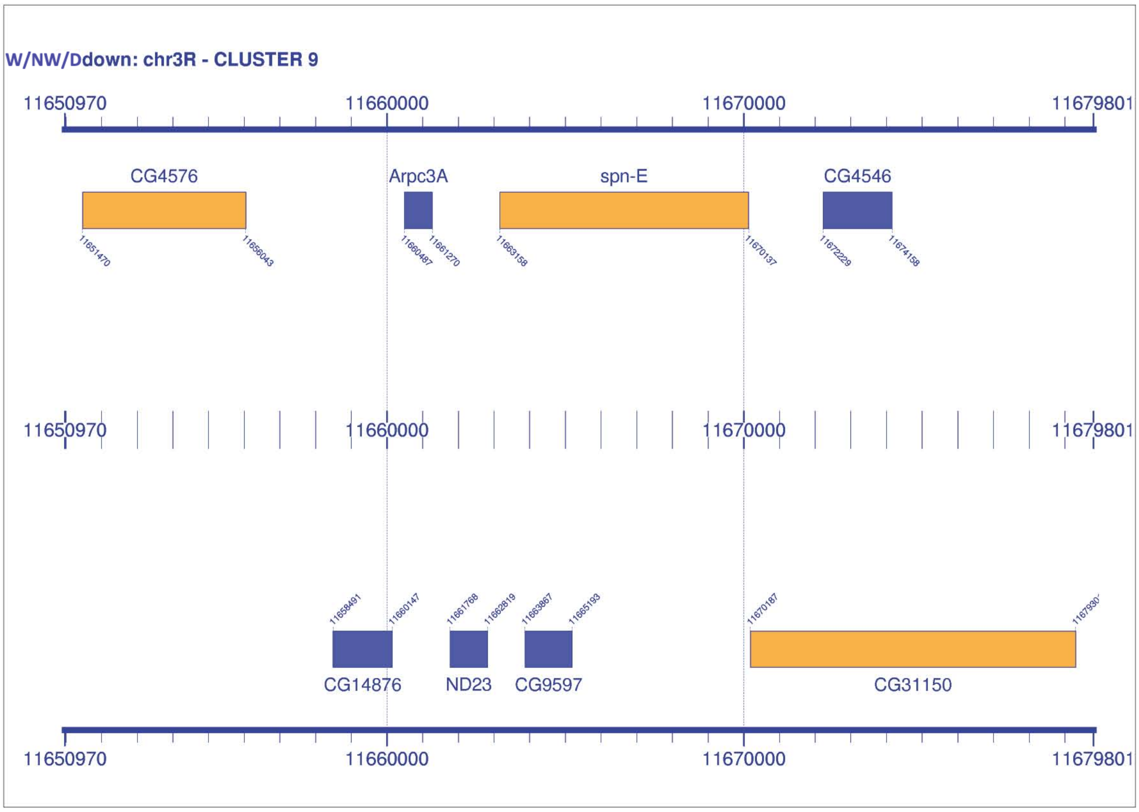

W / NW / D down - cluster 9

Genomic components:

NAME	RefSeq	Function	
CG4576	NM_142238		
CG14876	NM_142240		
ARPC3A	NM_142241	GO:0003779	actin binding
		GO:0005200	structural constituent of cytoskeleton
		GO:0005856	cytoskeleton
		GO:0007010	cytoskeleton organization and biogenesis
		GO:0030833	regulation of actin filament polymerization
ND23	NM_079980	GO:0003954	NADH dehydrogenase activity
		GO:0005506	iron ion binding
		GO:0005747	mitochondrial respiratory chain complex I
		GO:0006120	mitochondrial electron transport, NADH to ubiquinone
		GO:0008137	NADH dehydrogenase (ubiquinone) activity
SPN-E	NM_057393	GO:0000398	nuclear mRNA splicing, via spliceosome
		GO:0001556	oocyte maturation
		GO:0003676	nucleic acid binding
		GO:0003724	RNA helicase activity
		GO:0004004	ATP-dependent RNA helicase activity
		GO:0004386	helicase activity
		GO:0005524	ATP binding
		GO:0005737	cytoplasm
		GO:0006342	chromatin silencing
		GO:0006403	RNA localization
		GO:0007294	oocyte fate determination (sensu Insecta)
		GO:0007315	pole plasm assembly
		GO:0007317	regulation of pole plasm oskar mRNA localization
		GO:0008186	RNA-dependent ATPase activity
		GO:0008270	zinc ion binding
		GO:0008298	intracellular mRNA localization
		GO:0009949	polarity specification of anterior/posterior axis
		GO:0009951	polarity specification of dorsal/ventral axis
		GO:0009993	oogenesis (sensu Insecta)
		GO:0009994	oocyte differentiation
		GO:0030423	RNA interference, targeting of mRNA for destruction
		GO:0030717	karyosome formation
		GO:0030720	oocyte localization during oogenesis
		GO:0045451	pole plasm oskar mRNA localization
CG9597	NM_142242		
CG31150	NM_169676	GO:0005319	lipid transporter activity
		GO:0006869	lipid transport
CG4546	NM_142244	GO:0004054	arginine kinase activity
		GO:0019202	amino acid kinase activity

GO density (8 genes):

RANKING	GO id	Function	Frequency
1	GO:0007315	pole plasm assembly	12 %
2	GO:0030717	karyosome formation	12 %
3	GO:0003779	actin binding	12 %
4	GO:0019202	amino acid kinase activity	12 %
5	GO:0003676	nucleic acid binding	12 %
6	GO:0007317	regulation of pole plasm oskar mRNA localization	12 %
7	GO:0005200	structural constituent of cytoskeleton	12 %
8	GO:0003724	RNA helicase activity	12 %
9	GO:0008298	intracellular mRNA localization	12 %
10	GO:0007294	oocyte fate determination (sensu Insecta)	12 %
11	GO:0004054	arginine kinase activity	12 %
12	GO:0009951	polarity specification of dorsal/ventral axis	12 %
13	GO:0008270	zinc ion binding	12 %
14	GO:0006120	mitochondrial electron transport, NADH to ubiquinone	12 %
15	GO:0008186	RNA-dependent ATPase activity	12 %
16	GO:0006403	RNA localization	12 %
17	GO:0009993	oogenesis (sensu Insecta)	12 %
18	GO:0004386	helicase activity	12 %
19	GO:0005506	iron ion binding	12 %
20	GO:0009994	oocyte differentiation	12 %
21	GO:0006869	lipid transport	12 %
22	GO:0005524	ATP binding	12 %
23	GO:0009949	polarity specification of anterior/posterior axis	12 %
24	GO:0030423	RNA interference, targeting of mRNA for destruction	12 %
25	GO:0007010	cytoskeleton organization and biogenesis	12 %
26	GO:0004004	ATP-dependent RNA helicase activity	12 %
27	GO:0001556	oocyte maturation	12 %
28	GO:0030720	oocyte localization during oogenesis	12 %
29	GO:0003954	NADH dehydrogenase activity	12 %
30	GO:0000398	nuclear mRNA splicing, via spliceosome	12 %
31	GO:0006342	chromatin silencing	12 %
32	GO:0005856	cytoskeleton	12 %
33	GO:0008137	NADH dehydrogenase (ubiquinone) activity	12 %
34	GO:0045451	pole plasm oskar mRNA localization	12 %
35	GO:0005747	mitochondrial respiratory chain complex I	12 %
36	GO:0005319	lipid transporter activity	12 %
37	GO:0005737	cytoplasm	12 %
38	GO:0030833	regulation of actin filament polymerization	12 %

W/NW/D down - chr3R: 12000027-12028802

Genomic components: 3 coregulated genes, 5 genes

CHR	Strand	Start	End	RefSeq	Name	Exons	Description
CHR3R	+	12000027	12005976	NM_142269	CG8925	6	CG8925-PA
CHR3R	+	12006741	12011957	NM_142270	CG8927	4	CG8927-PA, isoform A
CHR3R	-	12011880	12012615	NM_079653	Bin1	2	Bicoid interacting protein 1 CG6046-PA
CHR3R	+	12012655	12023310	NM_079654	sra	2	sarah CG6072-PA
CHR3R	-	12023723	12028802	NM_142271	CG6126	3	CG6126-PA

Cluster size: 28776 nucleotides

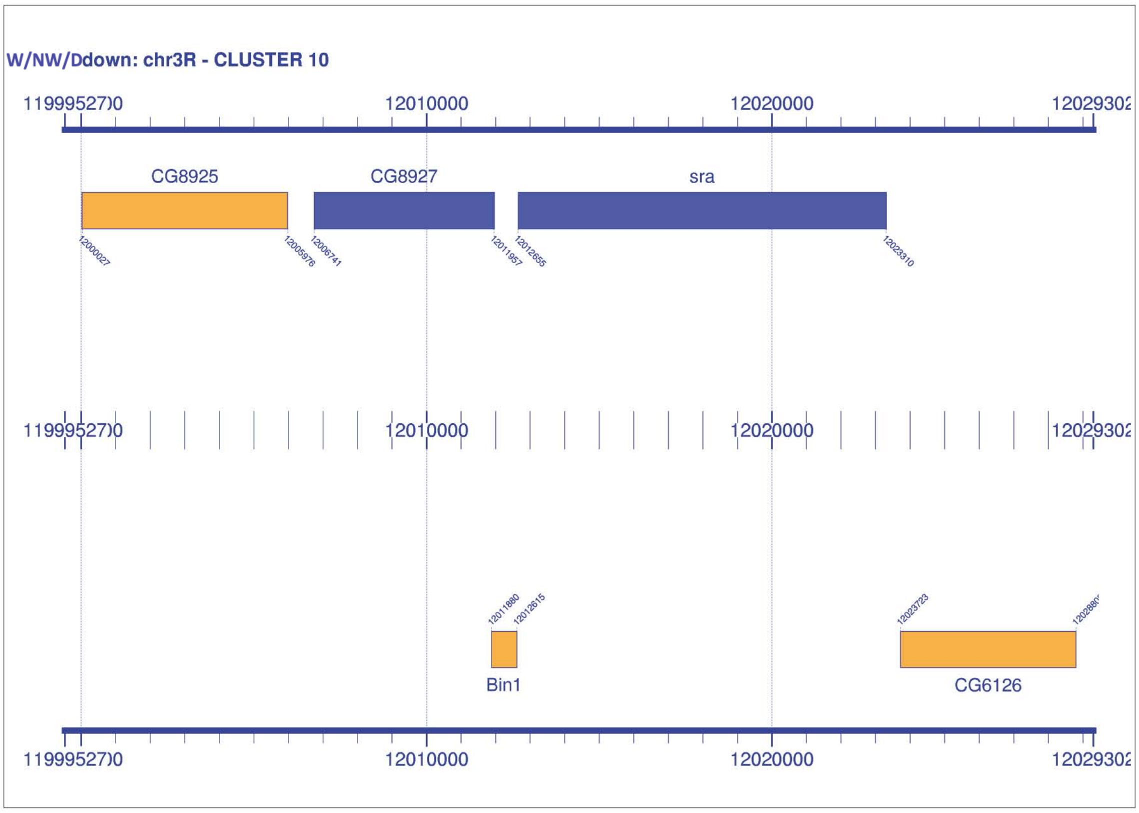

W/NW/D down - cluster 10

Genomic components:

Name	RefSeq	Function	
CG8925	NM_142269	GO:0006812	cation transport
		GO:0006858	extracellular transport
		GO:0008324	cation transporter activity
		GO:0015226	carnitine transporter activity
		GO:0016021	integral to membrane
CG8927	NM_142270		
Bin1	NM_079653	GO:0000122	negative regulation of transcription from RNA polymerase II
		GO:0003682	chromatin binding
		GO:0003714	transcription corepressor activity
		GO:0005515	protein binding
		GO:0005634	nucleus
		GO:0016580	Sin3 complex
		GO:0045892	negative regulation of transcription, DNA-dependent
sra	NM_079654	GO:0000166	nucleotide binding
		GO:0005102	receptor binding
		GO:0007127	meiosis I
		GO:0007616	long-term memory
		GO:0008355	olfactory learning
		GO:0019722	calcium-mediated signaling
		GO:0045924	regulation of female receptivity
		GO:0046008	regulation of female receptivity, post-mating
CG6126	NM_142271	GO:0005975	carbohydrate metabolic process
		GO:0006812	cation transport
		GO:0006858	extracellular transport
		GO:0008643	carbohydrate transport
		GO:0015101	organic cation transporter activity
		GO:0015144	carbohydrate transporter activity
		GO:0016021	integral to membrane

GO density (5 genes):

RANKING	GO id	Function	Frequency
1	GO:0006858	extracellular transport	40 %
2	GO:0016021	integral to membrane	40 %
3	GO:0006812	cation transport	40 %
4	GO:0008324	cation transporter activity	20 %
5	GO:0045924	regulation of female receptivity	20 %
6	GO:0008355	olfactory learning	20 %
7	GO:0005634	nucleus	20 %
8	GO:0046008	regulation of female receptivity, post-mating	20 %
9	GO:0005975	carbohydrate metabolic process	20 %
10	GO:0000166	nucleotide binding	20 %
11	GO:0015101	organic cation transporter activity	20 %
12	GO:0000122	negative regulation of transcription from RNA polymerase II	20 %
13	GO:0007616	long-term memory	20 %
14	GO:0015226	carnitine transporter activity	20 %
15	GO:0003714	transcription corepressor activity	20 %
16	GO:0008643	carbohydrate transport	20 %
17	GO:0016580	Sin3 complex	20 %
18	GO:0019722	calcium-mediated signaling	20 %
19	GO:0015144	carbohydrate transporter activity	20 %
20	GO:0007127	meiosis I	20 %
21	GO:0045892	negative regulation of transcription, DNA-dependent	20 %
22	GO:0003682	chromatin binding	20 %
23	GO:0005515	protein binding	20 %
24	GO:0005102	receptor binding	20 %
