# Supplementary material for: Identification and Functional Analysis of Healing Regulators in Drosophila
Source: PLoS Genet. 2015 Feb 3;11(2):e1004965. doi: 10.1371/journal.pgen.1004965 (PMC4315591; doi:10.1371/journal.pgen.1004965)
Supplement: S10 Table — CC (Cellular Component), BP (Biological Process) and MF (Molecular Function) GO enrichment analysis were performed. GO IDs, GO terms, node size, expected number, experimental number, p-value for enrichment and gene symbols are displayed. (PDF) [file pgen.1004965.s018.pdf]

Gene Ontology Terms Enrichment

Global Analysis: JNK+ W vs JNK- W (2 FC, p-value < 0.01)

- **CC (Cellular Component) GO Analysis**
- **BP (Biological Process) GO Analysis**
- **MF (Molecular Function) GO Analysis**

CC GO Analysis

GO Analysis

GOID	GOTerm	NodeSize	Exp.Count	Count	Pvalue	GeneSymb
GO:0005587	collagen type IV	2	0.065	2	0.001	vkg , Cg25C
GO:0035183	ring canal inner rim	3	0.097	2	0.0031	cher , kel
GO:0005576	extracellular region	380	12	30	2.7e-06	Ance , Tsf3 , CG11142 , Mmp1 , Tsp , Idgf3 , cdi , cher , ImpL2 , PGRP-SA , Timp , CecB , CecC , Pvf1 , bnl , Idgf4 , Tsf1 , Tig , CG3009 , kel , Nplp1 , CG16704 , rad , CG8483 , GLaz , vkg , CG33171 , BM-40-SPARC , Adgf-A , Cg25C
GO:0005581	collagen	3	0.097	3	3.3e-05	vkg , CG33171 , Cg25C
GO:0005578	extracellular matrix (sensu Metazoa)	43	1.3	5	0.0088	Mmp1 , Tsp , Timp , Tig , vkg , CG33171 , BM-40-SPARC , Cg25C

BP GO Analysis

coagulation

GOID	GOTerm	NodeSize	Exp.Count	Count	Pvalue	GeneSymb
GO:0050819	negative regulation of coagulation	3	0.11	3	4.6e-05	Anxb11 , AnnIX , AnnX
GO:0050817	coagulation	4	0.14	3	0.00018	Anxb11 , AnnIX , AnnX

lipid metabolism

GOID	GOTerm	NodeSize	Exp.Count	Count	Pvalue	GeneSymb
GO:0006629	lipid metabolism	480	17	33	0.00024	CG7461 , CG8498 , CG11438 , CG10877 , CG7367 , CG4267 , CG6805 , vib , Anxb11 , Pten , Sap-r , CG3523 , AnnIX , AnnX , CG14741 , CG1516 , wun , CG5646 , wun2 , CG7322 , CG18641 , CG8630 , CG6753 , rdgB , Gpdh , CG3009 , mdy , norpA , rad , Fbp2 , CDase , GLaz , Ugt58Fa

limb morphogenesis

GOID	GOTerm	NodeSize	Exp.Count	Count	Pvalue	GeneSymb
GO:0035108	limb morphogenesis	29	1	6	0.00047	drm , Dll , dac , Lim1 , AP-2 , bab1
GO:0007480	leg morphogenesis (sensu Endopterygota)	21	0.75	4	0.006	drm , Lim1 , AP-2 , bab1

carboxylic acid metabolism

GOID	GOTerm	NodeSize	Exp.Count	Count	Pvalue	GeneSymb
GO:0019752	carboxylic acid metabolism	390	14	27	0.00063	CG7461 , CG8498 , CG10877 , CG17259 , CG10924 , e , CG3523 , CG15279 , Aats-trp , Pepck , JhI-21 , Men , CG32412 , b , CG5535 , Aats-val , Tdc1 , CG1516 , CG8630 , CG10802 , aay , CG11241 , mdy , disco-r , CG33092 , CG1461 , Sox15
GO:0006094	gluconeogenesis	6	0.21	3	0.00084	CG10924 , Pepck , CG1516

development

GOID	GOTerm	NodeSize	Exp.Count	Count	Pvalue	GeneSymb
GO:0007399	nervous system development	510	18	32	0.0011	Tm1 , Rho1 , insc , Pvr , cib , CG31694 , tup , sn , mfas , Tsp42El , Tig , mirr , Dll , aay , E(spl) , lbl , lola , dac , br , Lim1 , mira , m4 , disco , CG11641 , svp , HLHm7 , tutl , alphaTub67C , hbs , Sox15 , Con , BM-40-SPARC
	cell					Ance , Tm1 , Klp64D , Pten , Rho1 , insc , Pvr , Rop , tup , spir , wbl , mfas , knrl , wun2 ,

GO:0030154	differentiation	500	18	29	0.0074	Tig , aay , E(spl) , lbl , lola , dac , br , mira , m4 , svp , hbs , tinc , twi , Con , pip

germ cell migration

GOID	GOTerm	NodeSize	Exp.Count	Count	Pvalue	GeneSymb
GO:0035233	germ cell repulsion	2	0.072	2	0.0013	wun , wun2
GO:0035234	germ cell programmed cell death	3	0.11	2	0.0037	wun , wun2

actin filament-based process

GOID	GOTerm	NodeSize	Exp.Count	Count	Pvalue	GeneSymb
GO:0030029	actin filament-based process	97	3.5	10	0.0024	Pten , Rho1 , Pvr , cpb , sn , spir , CalpA , CG13503 , cher , DAAM
GO:0007015	actin filament organization	61	2.2	7	0.0059	Pten , Rho1 , Pvr , cpb , sn , CG13503 , cher

imaginal disc development

GOID	GOTerm	NodeSize	Exp.Count	Count	Pvalue	GeneSymb
GO:0035218	leg disc development	39	1.4	6	0.0024	drm , Dll , dac , Lim1 , AP-2 , bab1
GO:0007561	imaginal disc eversion	4	0.14	2	0.0073	ImpE1 , ImpE3

maternal determination of dorsal/ventral axis, oocyte, soma encoded

GOID	GOTerm	NodeSize	Exp.Count	Count	Pvalue	GeneSymb
GO:0007313	maternal determination of dorsal/ventral axis, oocyte, soma encoded	3	0.11	2	0.0037	wbl , pip

lipid transport

GOID	GOTerm	NodeSize	Exp.Count	Count	Pvalue	GeneSymb
GO:0006869	lipid transport	57	2	7	0.004	CG8498 , vib , Sap-r , CG14741 , CG5646 , rdgB , GLaz

signal transduction

GOID	GOTerm	NodeSize	Exp.Count	Count	Pvalue	GeneSymb
GO:0007169	transmembrane receptor protein tyrosine kinase signaling pathway	120	4.4	11	0.0042	Pten , Pvr , aru , tup , bnl , pyr , E(spl) , Gap1 , CG7526 , CG5522 , Asph
						CG6453 , Rab30 , CG11438 , Stam , CG6805 , CG10737 , CG2061 , l(1)G0232 , Pten , Rho1 , Pvr , CG6749 , CG10089 , egr , spri ,

GO:0007165	signal transduction	1300	45	61	0.0083	aru , tup , CG30115 , Idgf3 , wbl , cdi , DAAM , Ac76E , mfas , CG10531 , knrl , PGRP-SA , wun , wun2 , CG4322 , bnl , Idgf4 , bt , mirr , pyr , E(spl) , Nep1 , Gap1 , CG11451 , Ac13E , Pkc98E , CG7526 , m4 , Nf1 , norpA , Nplp1 , svp , tutl , CG5036 , hbs , klu , CG5522 , Asph , CG11835 , CG4733 , Ptr , CG32843 , Con , pip , CG33171 , BM-40-SPARC

ectoderm development

GOID	GOTerm	NodeSize	Exp.Count	Count	Pvalue	GeneSymb
GO:0007398	ectoderm development	240	8.4	17	0.0045	Rho1 , Pvr , CG31694 , tup , mfas , Tsp42El , CG5397 , mirr , Dll , E(spl) , dac , Lim1 , CG11641 , HLHm7 , hbs , Sox15 , BM-40-SPARC

monosaccharide biosynthesis

GOID	GOTerm	NodeSize	Exp.Count	Count	Pvalue	GeneSymb
GO:0046364	monosaccharide biosynthesis	10	0.36	3	0.0045	CG10924 , Pepck , CG1516

morphogenesis

GOID	GOTerm	NodeSize	Exp.Count	Count	Pvalue	GeneSymb
GO:0009653	morphogenesis	630	11	20	0.0048	Ance , Klp64D , Anxb11 , ImpE1 , Pten , Rho1 , Pvr , cib , Lmpt , tup , sn , Syb , CG13503 , cher , mfas , knrl , drm , Strn-Mlck , Pvf1 , bnl , Tig , mirr , pyr , Dll , aay , E(spl) , lbl , lola , dac , br , Lim1 , Nf1 , svp , klu , ImpE3 , tinc , AP-2 , twi , bab1 , Con , Cg25C
GO:0009887	organ morphogenesis	330	12	21	0.0075	ImpE1 , Pten , Rho1 , Lmpt , drm , Strn-Mlck , mirr , pyr , Dll , E(spl) , lbl , dac , br , Lim1 , svp , klu , ImpE3 , tinc , AP-2 , twi , bab1

amino acid activation

GOID	GOTerm	NodeSize	Exp.Count	Count	Pvalue	GeneSymb
GO:0043038	amino acid activation	59	2.1	7	0.0049	CG17259 , e , Aats-trp , Aats-val , CG10802 , mdy , Sox15

cell ion homeostasis

GOID	GOTerm	NodeSize	Exp.Count	Count	Pvalue	GeneSymb
GO:0006875	metal ion homeostasis	23	0.82	4	0.0083	Tsf3 , Tsf1 , norpA , Rya-r44F
GO:0030005	di-, tri-valent inorganic cation homeostasis	21	0.75	4	0.006	Tsf3 , Tsf1 , norpA , Rya-r44F

GO:0006873	cell ion homeostasis	23	0.82	4	0.0083	Tsf3 , Tsf1 , norpA , Rya-r44F

response to biotic stimulus

GOID	GOTerm	NodeSize	Exp.Count	Count	Pvalue	GeneSymb
GO:0009607	response to biotic stimulus	500	18	29	0.0065	CG11652 , CG1102 , Tsf3 , e , CG6749 , egr , CG30437 , CG31728 , GstD3 , CG30022 , Prx2540-2 , AnnX , CG6045 , PGRP-SA , PGRP-LA , TepII , CecB , CG8913 , CecC , Tsf1 , CG5397 , CG6426 , gem , br , CG2887 , CG8170 , CG8483 , Ugt58Fa , CG5873
GO:0016045	detection of bacteria	4	0.14	2	0.0073	PGRP-SA , PGRP-LA

tracheal lumen formation

GOID	GOTerm	NodeSize	Exp.Count	Count	Pvalue	GeneSymb
GO:0035149	tracheal lumen formation	4	0.14	2	0.0073	Rho1 , Syb

acylglycerol biosynthesis

GOID	GOTerm	NodeSize	Exp.Count	Count	Pvalue	GeneSymb
GO:0046463	acylglycerol biosynthesis	4	0.14	2	0.0073	mdy , norpA

iron ion transport

GOID	GOTerm	NodeSize	Exp.Count	Count	Pvalue	GeneSymb
GO:0006826	iron ion transport	4	0.14	2	0.0073	Tsf3 , Tsf1

taxis

GOID	GOTerm	NodeSize	Exp.Count	Count	Pvalue	GeneSymb
GO:0042330	taxis	12	0.43	3	0.0079	wun , wun2 , bnl

MF GO Analysis

protein binding

GOID	GOTerm	NodeSize	Exp.Count	Count	Pvalue	GeneSymb
GO:0003779	actin binding	120	4.6	15	5e-05	CG33232 , Tm1 , Anxb11 , Pten , cib , cpb , sn , spir , AnnIX , Fim , cher , DAAM , AnnX , kel , mira
GO:0008083	growth factor activity	32	1.2	7	0.00014	Idgf3 , CG10531 , Pvf1 , bnl , Idgf4 , BM-40-SPARC , Adgf-A
GO:0005104	fibroblast growth factor receptor binding	3	0.11	2	0.0041	bnl , pyr

binding

GOID	GOTerm	NodeSize	Exp.Count	Count	Pvalue	GeneSymb
GO:0005509	calcium ion binding	210	8	20	0.00014	CG10126 , CG6453 , Anxb11 , Tsp , CG7447 , AnnIX , CalpA , Fim , AnnX , rdgB , CG6426 , CG3009 , lola , tok , CG7526 , norpA , rad , CG4733 , BM-40-SPARC , Rya-r44F
GO:0008289	lipid binding	68	2.5	8	0.0036	CG8498 , vib , Anxb11 , CG10737 , AnnIX , AnnX , rdgB , Pkc98E
GO:0005544	calcium-dependent phospholipid binding	12	0.45	3	0.0088	Anxb11 , AnnIX , AnnX

protease inhibitor activity

GOID	GOTerm	NodeSize	Exp.Count	Count	Pvalue	GeneSymb
GO:0030414	protease inhibitor activity	83	3.1	11	0.00024	Spn5 , Pten , Spn6 , Spn43Ab , TepII , Timp , CG31313 , CG7722 , CG8066 , CG14470 , CG16704
GO:0004867	serine-type endopeptidase inhibitor activity	68	2.5	8	0.0036	Spn5 , Pten , Spn6 , Spn43Ab , TepII , CG7722 , CG14470 , CG16704

molecular_function

GOID	GOTerm	NodeSize	Exp.Count	Count	Pvalue	GeneSymb
GO:0003704	specific RNA polymerase II transcription factor activity	76	2.8	8	0.0072	tup , Dll , lbl , Trl , lola , br , HLHm7 , twi
GO:0005201	extracellular matrix structural constituent	6	0.22	3	0.00095	vkg , CG33171 , Cg25C
GO:0005214	structural constituent of cuticle (sensu Insecta)	76	2.8	9	0.002	CG13224 , CG8511 , CG8502 , CG15006 , CG33302 , CG4818 , CG3474 , CG1919 ,

CG32029

lyase activity

GOID	GOTerm	NodeSize	Exp.Count	Count	Pvalue	GeneSymb
GO:0016829	lyase activity	160	5.9	14	0.0022	CG10877 , CG10924 , l(1)G0030 , ferrochelatase , Pepck , Men , b , CG30022 , Ac76E , Tdc1 , Ac13E , CG16733 , CG10899 , CG1461
GO:0004613	phosphoenolpyruvate carboxykinase (GTP) activity	2	0.075	2	0.0014	CG10924 , Pepck
GO:0016831	carboxy-lyase activity	34	1.3	5	0.008	CG10924 , Pepck , Men , b , Tdc1

protein kinase activity

GOID	GOTerm	NodeSize	Exp.Count	Count	Pvalue	GeneSymb
GO:0005021	vascular endothelial growth factor receptor activity	2	0.075	2	0.0014	Pvr , Strn-Mlck
GO:0004687	myosin light chain kinase activity	4	0.15	2	0.0079	Strn-Mlck , bt

hydrolase activity, acting on ester bonds

GOID	GOTerm	NodeSize	Exp.Count	Count	Pvalue	GeneSymb
GO:0004726	non-membrane spanning protein tyrosine phosphatase activity	4	0.15	2	0.0079	l(1)G0232 , Pten
GO:0008195	phosphatidate phosphatase activity	9	0.34	3	0.0037	CG11438 , wun , wun2
GO:0016789	carboxylic ester hydrolase activity	120	4.6	11	0.0058	CG4757 , CG7367 , CG4267 , CG4382 , CG18641 , CG6753 , CG3009 , norpA , rad , CDase , CG6414

sterol O-acyltransferase activity

GOID	GOTerm	NodeSize	Exp.Count	Count	Pvalue	GeneSymb
GO:0004772	sterol O-acyltransferase activity	3	0.11	2	0.0041	CG5397 , mdy
