# Supplementary material for: Identification and Functional Analysis of Healing Regulators in Drosophila
Source: PLoS Genet. 2015 Feb 3;11(2):e1004965. doi: 10.1371/journal.pgen.1004965 (PMC4315591; doi:10.1371/journal.pgen.1004965)
Supplement: S11 Table — CC (Cellular Component), BP (Biological Process) and MF (Molecular Function) GO enrichment analysis were performed. GO IDs, GO terms, node size, expected number, experimental number, p-value for enrichment and gene symbols are displayed. (PDF) [file pgen.1004965.s019.pdf]

Gene Ontology Terms Enrichment

WO: (1.3 FC, p-value < 0.05)

- **CC (Cellular Component) GO Analysis**
- **BP (Biological Process) GO Analysis**
- **MF (Molecular Function) GO Analysis**

CC GO Analysis

cytoplasm

GOID	GOTerm	NodeSize	Exp.Count	Count	Pvalue	GeneSymb
GO:0044431	Golgi apparatus part	39	2.6	7	0.014	gammaCop , Grasp65 , alpha-Man-I , pgant3 , Gos28 , Csat , CG9298
GO:0015935	small ribosomal subunit	35	2.4	7	0.0075	mRpS30 , RpS9 , mRpS2 , mRpS35 , mRpS33 , bonsai , mRpS7
GO:0005737	cytoplasm	810	55	77	0.00019	sec10 , Lrr47 , mRpS30 , CG1673 , RpS9 , CG4169 , Aats-asn , Tom , CG5599 , gammaCop , Surf1 , CG4769 , ifc , csw , Pp1alpha-96A , CG10635 , ird5 , Roe1 , Gl , CG7263 , Aats-arg , ND23 , bsf , Ero1L , CG6404 , mRpL35 , bor , CalpA , Grasp65 , CG3476 , mRpS2 , Asph , alpha-Man-I , CG31229 , pip , aub , Cpr , CG5805 , mRpS35 , Snap , Rab7 , CG3719 , mRpS33 , CG6764 , CG1967 , VhaAC39 , ox , mRpL17 , CG5037 , CG8412 , Pfk , Gpdh , CG6206 , CG16912 , Scamp , CG8858 , botv , pgant3 , CG6781 , Ptp61F , ND75 , Gos28 , Csat , Csl4 , Rlc1 , CG32549 , ND42 , Vha14 , Tim17a1 , CG7598 , CG11779 , bonsai , mRpL28 , san , CG1319 , CG9298 , mRpS7
GO:0005739	mitochondrion	270	18	33	0.00047	mRpS30 , CG1673 , CG4169 , CG5599 , Surf1 , CG4769 , ifc , Roe1 , CG7263 , ND23 , CG6404 , mRpL35 , CG3476 , mRpS2 , CG31229 , CG5805 , mRpS35 , CG3719 , mRpS33 , ox , mRpL17 , CG5037 , CG16912 , ND75 , Rlc1 , ND42 , Tim17a1 , CG7598 , CG11779 , bonsai , mRpL28 , CG1319 , mRpS7

mitochondrial part

GOID	GOTerm	NodeSize	Exp.Count	Count	Pvalue	GeneSymb
GO:0005759	mitochondrial matrix	100	6.8	14	0.0068	mRpS30 , CG5599 , Roe1 , mRpL35 , mRpS2 , mRpS35 , mRpS33 , mRpL17 , Rlc1 , ND42 , bonsai , mRpL28 , CG1319 , mRpS7
GO:0005740	mitochondrial envelope	110	7.4	16	0.0023	CG4169 , Surf1 , CG4769 , CG7263 , ND23 , CG6404 , CG3476 , CG31229 , CG5805 , ox , CG5037 , ND75 , ND42 , Tim17a1 , CG7598 , CG11779
GO:0005763	mitochondrial small ribosomal subunit	23	1.5	6	0.0033	mRpS30 , mRpS2 , mRpS35 , mRpS33 , bonsai , mRpS7

mitochondrial inner membrane

GOID	GOTerm	NodeSize	Exp.Count	Count	Pvalue	GeneSymb
GO:0005750	respiratory chain complex III (sensu Eukaryota)	6	0.4	3	0.0052	CG4169 , CG4769 , ox
GO:0005744	mitochondrial inner membrane presequence translocase complex	6	0.4	3	0.0052	CG31229 , Tim17a1 , CG11779
GO:0005743	mitochondrial inner membrane	91	5.7	11	0.025	CG4169 , Surf1 , CG4769 , ND23 , CG6404 , CG3476 , CG31229 , CG5805 , ox , ND75 , ND42 , Tim17a1 , CG7598 , CG11779

organellar ribosome

GOID	GOTerm	NodeSize	Exp.Count	Count	Pvalue	GeneSymb
GO:0000313	organellar ribosome	61	4.1	10	0.0066	mRpS30 , mRpL35 , mRpS2 , mRpS35 , mRpS33 , mRpL17 , Rlc1 , bonsai , mRpL28 , mRpS7

membrane part

GOID	GOTerm	NodeSize	Exp.Count	Count	Pvalue	GeneSymb
GO:0044425	membrane part	480	32	44	0.013	SerT , CG1698 , CG4169 , gammaCop , CG4769 , Gr61a , CG10440 , CG6230 , ND23 , CG14439 , Grasp65 , CG1208 , Gr63a , Asph , alpha-Man-I , CG31229 , Nhe1 , Tsp5D , heix , Snap , CG9467 , CG8602 , Nle , CG12121 , VhaAC39 , Pgm , ox , kek1 , CG5037 , Scamp , dnt , botv , Lar , CG7442 , ND75 , Gos28 , Lap1 , Csat , ND42 , shg , Vha14 , Tim17a1 , CG7598 , CG11779
GO:0008076	voltage-gated potassium channel complex	5	0.34	2	0.039	CG10440 , CG9467

envelope

GOID	GOTerm	NodeSize	Exp.Count	Count	Pvalue	GeneSymb
GO:0031975	envelope	150	10	17	0.024	CG4169 , Surf1 , CG4769 , CG7263 , ND23 , CG6404 , CG3476 , CG31229 , CG5805 , ox , CG5037 , GV1 , ND75 , ND42 , Tim17a1 , CG7598 , CG11779

actin cytoskeleton

GOID	GOTerm	NodeSize	Exp.Count	Count	Pvalue	GeneSymb
GO:0015629	actin cytoskeleton	37	2.5	6	0.035	Arc-p34 , CG10540 , Gl , Myo10A , Act57B , Lasp

organelle membrane

GOID	GOTerm	NodeSize	Exp.Count	Count	Pvalue	GeneSymb
GO:0031090	organelle membrane	210	14	21	0.041	CG4169 , gammaCop , Surf1 , CG4769 , ND23 , CG6404 , Grasp65 , CG3476 , Asph , alpha-Man-I , CG31229 , CG5805 , VhaAC39 , ox , ND75 , Csat , ND42 , Vha14 , Tim17a1 , CG7598 , CG11779

organelle part

GOID	GOTerm	NodeSize	Exp.Count	Count	Pvalue	GeneSymb
GO:0044422	organelle part	840	57	68	0.042	usp , Arc-p34 , mRpS30 , alphaTub85E , bab2 , RpS9 , CSN1b , CG4169 , CG11583 , CG6751 , CG10540 , CG5599 , gammaCop , Surf1 , CG4769 , Roe1 , Gl , CG7263 , Caf1-105 , ND23 , Mes4 , CG6404 , mRpL35 , Rrp42 , Myo10A , Grasp65 , CG3476 , mRpS2 , Asph , alpha-Man-I , CG31229 , CG5805 , CG6610 , mRpS35 , mRpS33 , CG6764 , Zpr1 , VhaAC39 , Su(z)2 , ox , mRpL17 , Act57B , CG5037 , GV1 , CG3371 , Nopp140 , pgant3 , CG31119 , CG6712 , ND75 , Gos28 , Csat , Csl4 , Rlc1 , Khc-73 , ND42 , Vha14 , Tim17a1 , Ssb-c31a , CG7598 , CG11779 , mod(mdg4) , bonsai , mRpL28 , san , CG1319 , CG9298 , mRpS7

BP GO Analysis

cellular protein metabolism

GOID	GOTerm	NodeSize	Exp.Count	Count	Pvalue	GeneSymb
GO:0006124	ferredoxin metabolism	4	0.27	2	0.026	CG7263 , CG1319
GO:0009100	glycoprotein metabolism	49	3.4	9	0.0053	CG1597 , alpha-Man-I , CG33145 , CG8412 , beta4GalNAcTB , pgant3 , CG3810 , beta4GalNAcTA , Csat
GO:0006412	protein biosynthesis	280	19	29	0.013	mRpS30 , RpS9 , Aats-asn , CG1597 , Aats-arg , mRpL35 , mRpS2 , alpha-Man-I , CG33145 , CG1750 , pelo , aub , mRpS35 , CG12263 , mRpS33 , CG6764 , mRpL17 , CG8412 , CG16912 , Nmt , CG14712 , beta4GalNAcTB , pgant3 , CG3810 , beta4GalNAcTA , Csat , bonsai , mRpL28 , mRpS7

cellular biosynthesis

GOID	GOTerm	NodeSize	Exp.Count	Count	Pvalue	GeneSymb
GO:0044249	cellular biosynthesis	430	30	43	0.0059	mRpS30 , CG1673 , RpS9 , Aats-asn , Glycogenin , CG1597 , Aats-arg , mRpL35 , CG6287 , mRpS2 , alpha-Man-I , CG33145 , SpdS , CG1750 , pelo , aub , heix , Sptr , mRpS35 , CG12263 , mRpS33 , CG6764 , VhaAC39 , CG1749 , mRpL17 , Act57B , CG5037 , CG8412 , CG30090 , CG16912 , Nmt , botv , CG14712 , beta4GalNAcTB , pgant3 , CG3810 , beta4GalNAcTA , Csat , Vha14 , bonsai , mRpL28 , b , mRpS7
GO:0009309	amine biosynthesis	37	2.5	6	0.038	CG1673 , CG6287 , SpdS , CG1749 , CG30090 , b

humoral defense mechanism (sensu Protostomia)

GOID	GOTerm	NodeSize	Exp.Count	Count	Pvalue	GeneSymb
GO:0006961	antibacterial humoral response (sensu Protostomia)	18	1.2	5	0.0059	ird5 , CecB , CecC , DptB , AttC
GO:0016065	humoral defense mechanism (sensu Protostomia)	28	1.9	5	0.039	ird5 , CecB , CecC , DptB , AttC

sex differentiation

GOID	GOTerm	NodeSize	Exp.Count	Count	Pvalue	GeneSymb
GO:0045137	development of primary sexual characteristics	16	1.1	4	0.02	bab1 , bab2 , ct , shg
GO:0008585	female gonad development	7	0.48	3	0.0091	bab1 , bab2 , ct
GO:0046660	female sex differentiation	9	0.62	3	0.02	bab1 , bab2 , ct

ATP synthesis coupled electron transport

GOID	GOTerm	NodeSize	Exp.Count	Count	Pvalue	GeneSymb
GO:0006122	mitochondrial electron transport, ubiquinol to cytochrome c	7	0.48	3	0.0091	CG4169 , CG4769 , ox
GO:0042773	ATP synthesis coupled electron transport	39	2.7	6	0.047	CG4169 , CG4769 , ND23 , ox , ND75 , ND42

localization

GOID	GOTerm	NodeSize	Exp.Count	Count	Pvalue	GeneSymb
GO:0051179	localization	940	64	80	0.012	usp , SerT , CG1698 , sec10 , CG3191 , alphaTub85E , Fas1 , CG4169 , CG10540 , gammaCop , CG4769 , csw , Roe1 , CG10440 , Gl , CG6230 , ena , ND23 , CG14439 , Ero1L , Rab39 , opa , CG3476 , CG1208 , CG16704 , kni , CG8931 , Rab8 , CG31229 , CG10657 , Nhe1 , TpnC73F , Tsp5D , aub , Cpr , CG5805 , CG7768 , Snap , CG9467 , CG12773 , Rab7 , msn , CG8602 , CG7864 , CG3719 , CG10971 , CG1967 , VhaAC39 , ox , Gpdh , Syx13 , Ras64B , Scamp , Rae1 , dnt , Sip1 , LpR1 , Lar , CG7442 , CG8055 , Ptp61F , ND75 , Gos28 , Hph , Lap1 , Csat , Khc-73 , CG1598 , ND42 , shg , Vha14 , Tim17a1 , CG11779 , mod(mdg4) , CG3860 , l(1)G0155 , Cyp18a1 , CG9298 , Grp1 , Arf84F
GO:0046903	secretion	120	8.2	14	0.033	sec10 , gammaCop , Rab8 , Snap , Rab7 , CG1967 , Syx13 , Ras64B , Scamp , Gos28 , Khc-73 , CG9298 , Grp1 , Arf84F
GO:0015031	protein transport	300	20	29	0.028	sec10 , alphaTub85E , gammaCop , Roe1 , Gl , Ero1L , Rab39 , Rab8 , CG31229 , CG7768 , Snap , Rab7 , CG7864 , CG10971 , CG1967 , Syx13 , Ras64B , Scamp , LpR1 , CG8055 , Gos28 , Lap1 , Khc-73 , Tim17a1 , CG11779 , l(1)G0155 , CG9298 , Grp1 , Arf84F
GO:0006836	neurotransmitter transport	3	0.21	2	0.013	SerT , CG1698
GO:0016192	vesicle-mediated transport	160	11	18	0.025	sec10 , gammaCop , Gl , Ero1L , Rab39 , Rab8 , Snap , Rab7 , CG10971 , CG1967 , Syx13 , Ras64B , Scamp , Gos28 , Khc-73 , CG9298 , Grp1 , Arf84F

secretory pathway

GOID	GOTerm	NodeSize	Exp.Count	Count	Pvalue	GeneSymb
	ER to Golgi vesicle-mediated					

GO:0006888	transport	8	0.55	3	0.014	Snap , Gos28 , CG9298
GO:0017157	regulation of exocytosis	20	1.4	4	0.044	Rab8 , Rab7 , Ras64B , Arf84F

cell organization and biogenesis

GOID	GOTerm	NodeSize	Exp.Count	Count	Pvalue	GeneSymb
GO:0006997	nuclear organization and biogenesis	9	0.62	3	0.02	pelo , Nopp140 , mod(mdg4)
GO:0051128	regulation of cell organization and biogenesis	20	1.4	4	0.044	Arc-p34 , CG10540 , ena , mod(mdg4)

protein targeting to mitochondrion

GOID	GOTerm	NodeSize	Exp.Count	Count	Pvalue	GeneSymb
GO:0006626	protein targeting to mitochondrion	17	1.2	4	0.025	Roe1 , CG31229 , Tim17a1 , CG11779

protein amino acid glycosylation

GOID	GOTerm	NodeSize	Exp.Count	Count	Pvalue	GeneSymb
GO:0006487	protein amino acid N-linked glycosylation	11	0.75	3	0.035	alpha-Man-I , CG8412 , CG3810
GO:0006486	protein amino acid glycosylation	48	2.5	6	0.038	CG1597 , alpha-Man-I , CG33145 , CG8412 , beta4GalNAcTB , pgant3 , CG3810 , beta4GalNAcTA , Csat

spiracle morphogenesis

GOID	GOTerm	NodeSize	Exp.Count	Count	Pvalue	GeneSymb
GO:0035277	spiracle morphogenesis	5	0.34	2	0.041	ct , ems

gastrulation (sensu Insecta)

GOID	GOTerm	NodeSize	Exp.Count	Count	Pvalue	GeneSymb
GO:0010004	gastrulation (sensu Insecta)	12	0.82	3	0.044	twi , Ptp61F , shg

MF GO Analysis

transporter activity

GOID	GOTerm	NodeSize	Exp.Count	Count	Pvalue	GeneSymb
GO:0005328	neurotransmitter:sodium symporter activity	2	0.13	2	0.0045	SerT , CG1698
GO:0051184	cofactor transporter activity	5	0.34	2	0.04	Surf1 , CG3476
GO:0015291	porter activity	70	4.7	10	0.018	SerT , CG1698 , CG3476 , CG1208 , Nhe1 , CG12773 , Sip1 , CG7442 , Csat , CG1598
GO:0005386	carrier activity	180	9	16	0.016	SerT , CG1698 , CG6230 , CG6404 , CG3476 , CG1208 , CG31229 , CG10657 , Nhe1 , Tsp5D , CG5805 , CG12773 , CG1967 , VhaAC39 , Scamp , Sip1 , CG7442 , CG8055 , Csat , CG1598 , Vha14 , Tim17a1 , CG11779
GO:0015399	primary active transporter activity	49	3.3	7	0.044	CG6230 , CG31229 , VhaAC39 , CG1598 , Vha14 , Tim17a1 , CG11779

Ras GTPase binding

GOID	GOTerm	NodeSize	Exp.Count	Count	Pvalue	GeneSymb
GO:0017016	Ras GTPase binding	2	0.13	2	0.0045	Lrr47 , Lap1

mannosidase activity

GOID	GOTerm	NodeSize	Exp.Count	Count	Pvalue	GeneSymb
GO:0015923	mannosidase activity	7	0.47	3	0.0086	alpha-Man-I , CG6206 , CG3810
GO:0004571	mannosyl-oligosaccharide 1,2-alpha-mannosidase activity	4	0.27	2	0.025	alpha-Man-I , CG3810

cation transporter activity

GOID	GOTerm	NodeSize	Exp.Count	Count	Pvalue	GeneSymb
GO:0008121	ubiquinol-cytochrome-c reductase activity	5	0.34	2	0.04	CG4169 , ox
GO:0015385	sodium:hydrogen antiporter activity	3	0.2	2	0.013	Nhe1 , Sip1
GO:0015491	cation:cation antiporter activity	3	0.2	2	0.013	Nhe1 , Sip1

transferase activity, transferring glycosyl groups

GOID	GOTerm	NodeSize	Exp.Count	Count	Pvalue	GeneSymb
	acetylgalactosaminyltransferase					beta4GalNAcTB , pgant3 ,

GO:0008376	activity	8	0.54	3	0.013	beta4GalNAcTA
GO:0016757	transferase activity, transferring glycosyl groups	72	4.9	10	0.021	Glycogenin , CG4433 , CG33145 , Act57B , CG33138 , CG8412 , botv , beta4GalNAcTB , pgant3 , beta4GalNAcTA

oxidoreductase activity

GOID	GOTerm	NodeSize	Exp.Count	Count	Pvalue	GeneSymb
GO:0016706	oxidoreductase activity, acting on paired donors, with incorporation or reduction of molecular oxygen, 2-oxoglutarate as one donor, and incorporation of one atom each of oxygen into both donors	9	0.61	3	0.019	CG31120 , Asph , Hph
GO:0016616	oxidoreductase activity, acting on the CH-OH group of donors, NAD or NADP as acceptor	50	3.4	8	0.017	Zw , CG6287 , CG6084 , Sptr , CG1749 , CG4842 , Gpdh , CG1444
GO:0009055	electron carrier activity	32	2.2	6	0.018	CG4769 , ND23 , Cpr , ND75 , ND42 , CG1319

structural constituent of ribosome

GOID	GOTerm	NodeSize	Exp.Count	Count	Pvalue	GeneSymb
GO:0003735	structural constituent of ribosome	96	6.5	12	0.026	mRpS30 , RpS9 , mRpL35 , mRpS2 , mRpS35 , mRpS33 , CG6764 , mRpL17 , Rlc1 , bonsai , mRpL28 , mRpS7

SNAP receptor activity

GOID	GOTerm	NodeSize	Exp.Count	Count	Pvalue	GeneSymb
GO:0005484	SNAP receptor activity	11	0.74	3	0.033	Syx13 , Gos28 , l(1)G0155

binding

GOID	GOTerm	NodeSize	Exp.Count	Count	Pvalue	GeneSymb
GO:0051087	chaperone binding	5	0.34	2	0.04	CG10635 , Roe1
GO:0042562	hormone binding	5	0.34	2	0.04	usp , kek1

protein translocase activity

GOID	GOTerm	NodeSize	Exp.Count	Count	Pvalue	GeneSymb
GO:0015450	protein translocase activity	12	0.81	3	0.042	CG31229 , Tim17a1 , CG11779

3'-5'-exoribonuclease activity

GOID	GOTerm	NodeSize	Exp.Count	Count	Pvalue	GeneSymb
GO:0000175	3'-5'-exoribonuclease activity	12	0.81	3	0.042	Rrp42 , Csl4 , mRpL28
