# Supplementary material for: Identification and Functional Analysis of Healing Regulators in Drosophila
Source: PLoS Genet. 2015 Feb 3;11(2):e1004965. doi: 10.1371/journal.pgen.1004965 (PMC4315591; doi:10.1371/journal.pgen.1004965)
Supplement: S12 Table — CC (Cellular Component), BP (Biological Process) and MF (Molecular Function) GO enrichment analysis were performed. GO IDs, GO terms, node size, expected number, experimental number, p-value for enrichment and gene symbols are displayed. (PDF) [file pgen.1004965.s020.pdf]

Gene Ontology Terms Enrichment

W/NW/D: (1.3 FC, p-value < 0.05)

- **CC (Cellular Component) GO Analysis**
- **BP (Biological Process) GO Analysis**
- **MF (Molecular Function) GO Analysis**

CC GO Analysis

extracellular region

GOID	GOTerm	NodeSize	Exp.Count	Count	Pvalue	GeneSymb
GO:0005576	extracellular region	150	14	39	3.1e-10	Mmp1 , Appl , Adgf-A , Obp56a , NetA , Tequila , AttA , NPC2 , BM-40-SPARC , bnl , CG6041 , Nplp1 , kal-1 , CG11142 , PGRP-SA , CG30488 , cdi , NLaz , AttD , Pvf1 , CG32209 , Tsf1 , CG33171 , Obp99a , beat-IIIc , os , Tig , CG12009 , Drs , CG31839 , CG32499 , dlp , vkg , lbm , CG1869 , CG8483 , Idgf2 , ImpL1 , spz
GO:0005578	extracellular matrix (sensu Metazoa)	28	2.4	7	0.0078	Mmp1 , NetA , BM-40-SPARC , kal-1 , CG33171 , os , Tig , dlp , vkg
GO:0005581	collagen	2	0.19	2	0.0086	CG33171 , vkg

integral to membrane

GOID	GOTerm	NodeSize	Exp.Count	Count	Pvalue	GeneSymb
GO:0016021	integral to membrane	330	31	43	0.011	Appl , w , Orct , Tequila , PGRP-LA , CCKLR-17D3 , CG8654 , CG11835 , stan , CG15556 , CG30035 , Fas3 , pain , PGRP-SA , Hmgcr , CG31547 , Gr94a , CG8925 , scb , alphaPS5 , CG4663 , CG4322 , PGRP-LF , Rya-r44F , CG7188 , l(1)G0232 , CG4101 , CG6126 , CG17108 , Tsp42Ed , Tsp42El , Fs(2)Ket , mthl2 , CG32843 , Ca-P60A , l(2)03659 , mthl3 , Gpi1 , mas , lbm , CG3603 , CG3649 , wun2

microsome

GOID	GOTerm	NodeSize	Exp.Count	Count	Pvalue	GeneSymb
GO:0005792	microsome	23	2.1	6	0.016	Cyp4e2 , Cyp6a17 , Cyp6d4 , Cyp6a20 , Cyp6d2 , Cyp4d2

membrane

GOID	GOTerm	NodeSize	Exp.Count	Count	Pvalue	GeneSymb
GO:0016020	membrane	750	70	84	0.023	Ucp4A , mod(mdg4) , Appl , w , CG8630 , Orct , CG9000 , Tequila , CG5254 , PGRP-LA , CCKLR-17D3 , CG8654 , CG8451 , CG11835 , stan , sad , CG15556 , CG5535 , CG30035 , rpk , Cyp4e2 , Cyp6a17 , klar , GlcAT-S , Fas3 , pain , PGRP-SA , Hmgcr , CG31547 , Gr94a , CG8925 , scb , CG3896 , Mec2 , alphaPS5 , Pvf2 , Cyp6d4 , Rep , CG6812 , Pvf1 , Cyp6a20 , Ect4 , tinc , CG4663 , zetaCOP , CG4322 , PGRP-LF , bves , Rya-r44F , CG7188 , l(1)G0232 , CG4101 , arr ,

						CG6126 , CG17108 , Tsp42Ed , Tsp42El , dlg1 , gukh , Cyp6d2 , Fs(2)Ket , Syb , mthl2 , CG11883 , CG32843 , CG1139 , Ca-P60A , Ptr , Cyp4d2 , l(2)03659 , Nrg , CG8027 , mthl3 , kst , Gpi1 , mas , lbm , synaptogyrin , CG3603 , egr , path , CG3649 , CG6739 , wun2

septate junction

GOID	GOTerm	NodeSize	Exp.Count	Count	Pvalue	GeneSymb
GO:0005918	septate junction	9	0.84	3	0.044	Fas3 , dlg1 , Nrg

Rab-protein geranylgeranyltransferase complex

GOID	GOTerm	NodeSize	Exp.Count	Count	Pvalue	GeneSymb
GO:0005968	Rab-protein geranylgeranyltransferase complex	4	0.37	2	0.046	Rep , CG12007

lateral plasma membrane

GOID	GOTerm	NodeSize	Exp.Count	Count	Pvalue	GeneSymb
GO:0016328	lateral plasma membrane	4	0.37	2	0.046	Fas3 , Nrg

membrane fraction

GOID	GOTerm	NodeSize	Exp.Count	Count	Pvalue	GeneSymb
GO:0005624	membrane fraction	29	2.7	6	0.047	Cyp4e2 , Cyp6a17 , Cyp6d4 , Cyp6a20 , Cyp6d2 , Cyp4d2

BP GO Analysis

lipid metabolism

GOID	GOTerm	NodeSize	Exp.Count	Count	Pvalue	GeneSymb
GO:0008202	steroid metabolism	49	4.7	16	6.6e-06	Ugt86Da , sad , Ugt35a , Cyp4e2 , Cyp6a17 , Hmgcr , Cyp6d4 , CG7724 , Cyp6a20 , Ugt58Fa , Cyp301a1 , arr , Cyp6d2 , Cyp4d2 , Ugt86Di , CG31809
GO:0006629	lipid metabolism	220	22	40	6.4e-05	CG6847 , CG18641 , Ugt86Da , CG8630 , CG5254 , CG10877 , CG2201 , sad , CG6805 , AnnX , Ugt35a , Cyp4e2 , Cyp6a17 , GlcAT-S , Hmgcr , CG31549 , NLaz , Cyp6d4 , CG7724 , CG4267 , Cyp6a20 , Ugt58Fa , CG5646 , CG12171 , Cyp301a1 , arr , Cyp6d2 , Cyp4d2 , Gpi1 , CG7367 , CG3603 , Sap-r , AnnIX , CG6753 , AcCoAS , Ugt86Di , CG31809 , CG31005 , wun2 , CG7402

response to stimulus

GOID	GOTerm	NodeSize	Exp.Count	Count	Pvalue	GeneSymb
GO:0006935	chemotaxis	6	0.58	3	0.014	bnl , Hmgcr , wun2
GO:0042330	taxis	10	0.38	2	0.049	nonA , bnl , pain , Hmgcr , wun2
GO:0009612	response to mechanical stimulus	3	0.29	2	0.026	sda , pain
GO:0007610	behavior	92	8.8	16	0.013	sda , Obp56a , nonA , bnl , Fas3 , Nf1 , pain , Hmgcr , scb , Obp99a , nemy , tun , nord , e , dsx , wun2
GO:0009628	response to abiotic stimulus	180	17	34	5.3e-05	GstE1 , sda , Obp56a , Ugt86Da , Eip93F , GstE5 , nonA , GstE6 , bnl , Ugt35a , Fas3 , Nf1 , GstE9 , pain , swi2 , Hmgcr , scb , grp , GstD2 , CG30437 , BthD , Kr-h1 , Ugt58Fa , Obp99a , Hsp26 , tun , br , GstD3 , mei-41 , nord , CG18528 , l(2)03659 , CG5377 , Hsp67Bc , Ugt86Di , wun2
GO:0050896	response to stimulus	520	25	38	0.0051	GstE1 , CG5873 , dl , sda , TotM , Obp56a , Ugt86Da , Eip93F , GstE5 , AttA , CG8170 , nonA , PGRP-LA , GstE6 , CG11835 , CG1102 , CG13744 , CG9460 , bnl , AnnX , d , Ugt35a , Fas3 , D , Nf1 , GstE9 , pain , PGRP-SA , swi2 , CG30488 , Hmgcr , Gr94a , scb , grp , CG3896 , TepII , GstD2 , CG30437 , CG9296 , BthD , AttD , Kr-h1 , CG6749 , Ugt58Fa , Tsf1 , CG15629 , PGRP-LF , Obp99a , CG8913 , os , Def , CG6426 , nemy , Hsp26 , tun , Drs , CG31728 , br , GstD3 , mei-41 , CG17227 , psh , mthl2 , nec , nord , CG18528 , l(2)03659 , CG7003 , CG5377 , mthl3 , Hsp67Bc , Ark , CG5397 , mas , CG9593 , egr , e , CG8483 , CG18522 , dsx , spz , Ugt86Di , Lig4 , wun2

defense response

GOID	GOTerm	NodeSize	Exp.Count	Count	Pvalue	GeneSymb
GO:0050832	defense response to fungi	10	0.96	4	0.011	Drs , psh , nec , spz
GO:0006952	defense response	240	17	32	0.00017	GstE1 , CG5873 , dl , TotM , Ugt86Da , GstE5 , AttA , CG8170 , PGRP-LA , GstE6 , CG1102 , CG13744 , CG9460 , AnnX , Ugt35a , GstE9 , PGRP-SA , CG30488 , CG3896 , TepII , GstD2 , CG30437 , AttD , CG6749 , Ugt58Fa , Tsf1 , PGRP-LF , CG8913 , os , Def , CG6426 , Hsp26 , Drs , CG31728 , GstD3 , psh , nec , CG18528 , l(2)03659 , CG5377 , Hsp67Bc , CG5397 , CG9593 , egr , e , CG8483 , CG18522 , spz , Ugt86Di
GO:0042742	defense response to bacteria	37	3.6	9	0.0069	AttA , PGRP-SA , TepII , AttD , Def , CG6426 , Drs , CG9593 , spz

response to chemical stimulus

GOID	GOTerm	NodeSize	Exp.Count	Count	Pvalue	GeneSymb
GO:0042048	olfactory behavior	35	3.4	7	0.045	Obp56a , Fas3 , Nf1 , scb , Obp99a , tun , nord
GO:0009636	response to toxin	53	5.1	14	0.00033	GstE1 , Ugt86Da , GstE5 , GstE6 , Ugt35a , GstE9 , GstD2 , CG30437 , Ugt58Fa , GstD3 , CG18528 , l(2)03659 , CG5377 , Ugt86Di
GO:0009725	response to hormone stimulus	14	1.3	4	0.039	Eip93F , swi2 , Kr-h1 , br

biological_process

GOID	GOTerm	NodeSize	Exp.Count	Count	Pvalue	GeneSymb
GO:0019730	antimicrobial humoral response	34	3.3	9	0.0038	AttA , PGRP-SA , TepII , AttD , Def , Drs , psh , nec , spz
GO:0009613	response to pest, pathogen or parasite	50	4.8	13	0.00063	dl , TotM , AttA , PGRP-SA , TepII , AttD , Def , Drs , br , psh , nec , egr , spz
GO:0016065	humoral defense mechanism (sensu Protostomia)	28	2.7	8	0.0037	TotM , AttA , TepII , AttD , Def , Drs , nec , spz
						mod(mdg4) , dl , TotM , Appl , sqh , Obp56a , NetA , AttA , sc , nonA , PGRP-LA ,

GO:0050874	organismal physiological process	430	35	49	0.0097	CCKLR-17D3 , sno , CG11835 , stan , bt , CG2121 , lola , d , Prat , kal-1 , D , pain , PGRP-SA , Gr94a , pnt , CG3896 , TepII , CG9296 , ara , Pkc98E , Rep , AttD , CG6749 , CG15629 , lbk , PGRP-LF , Obp99a , Rya-r44F , Def , Hsp26 , Drs , Stam , Tsp42El , dlg1 , br , Syb , psh , mthl2 , CG32843 , nec , Ca-P60A , spn-E , mthl3 , mas , ETH , lbm , synaptogyrin , CG5096 , egr , e , raps , spz , Dr
GO:0045087	innate immune response	14	1.3	5	0.0077	PGRP-LA , PGRP-SA , PGRP-LF , Drs , spz
GO:0006955	immune response	62	4.6	10	0.014	dl , TotM , AttA , PGRP-LA , PGRP-SA , CG3896 , TepII , AttD , PGRP-LF , Def , Drs , psh , nec , egr , spz

regulation of signal transduction

GOID	GOTerm	NodeSize	Exp.Count	Count	Pvalue	GeneSymb
GO:0008592	regulation of Toll signaling pathway	3	0.29	3	0.00088	PGRP-SA , psh , nec
GO:0046425	regulation of JAK-STAT cascade	4	0.38	2	0.049	Stam , Socs36E

polysaccharide metabolism

GOID	GOTerm	NodeSize	Exp.Count	Count	Pvalue	GeneSymb
GO:0005976	polysaccharide metabolism	61	5.9	13	0.0044	Ugt86Da , Tequila , Ugt35a , CG11142 , Ugt58Fa , CG32209 , CG12009 , CG32499 , Gpi1 , pgant2 , CG1869 , Hexo2 , Ugt86Di
GO:0006030	chitin metabolism	19	1.8	6	0.007	Tequila , CG11142 , CG32209 , CG12009 , CG32499 , CG1869

G-protein coupled receptor protein signaling pathway

GOID	GOTerm	NodeSize	Exp.Count	Count	Pvalue	GeneSymb
GO:0007186	G-protein coupled receptor protein signaling pathway	100	10	19	0.0046	CCKLR-17D3 , CG11835 , stan , CG15556 , loco , Nplp1 , Nf1 , CG11968 , CG6749 , CG4322 , l(1)G0232 , mthl2 , CG32843 , mthl3 , mas , ETH , CG3603 , raps , wun2

development

GOID	GOTerm	NodeSize	Exp.Count	Count	Pvalue	GeneSymb
GO:0048731	system development	350	33	46	0.012	form3 , Mmp1 , dl , Appl , caup , NetA , Eip93F , CG5254 , sc , BM-40-SPARC , noc , frc , HLHmgamma , stan , sad , bnl , lola , loco , klar , zfh1 , Fas3 , D , scb , pnt , ara , cathD , cib , lbk , Sox15 , HLHmdelta , stumps , CG7860 , Tig , Tsp42El , dlg1 , br ,

						Syb , Nrg , ewg , lbm , mirr , raps , dsx , Dr , Nc , so
GO:0007530	sex determination	26	2.5	7	0.0094	mod(mdg4) , sc , lola , Mes-4 , os , br , dsx
GO:0009653	morphogenesis	390	38	53	0.0049	form3 , dl , sqh , caup , NetA , sc , sno , frc , stan , sad , bnl , lola , loco , CG3770 , klar , zfh1 , Fas3 , Klp64D , D , Nf1 , Hmgcr , klu , scb , pnt , ara , CG7194 , Kr-h1 , Pvf1 , cib , tinc , lbk , grn , os , stumps , arr , sob , Tig , dlg1 , br , mei-41 , Fs(2)Ket , Syb , Lmpt , CG3618 , Nrg , ewg , CG12007 , Ark , mirr , dsx , Dr , Nc , so
GO:0048513	organ development	400	38	53	0.0058	Mmp1 , dl , caup , Eip93F , CG5254 , sc , nonA , sno , frc , HLHmgamma , stan , sad , bnl , klar , zfh1 , D , Hmgcr , klu , scb , grp , pnt , Pvf2 , ara , CG7194 , cathD , Pvf1 , cib , tinc , Mes2 , grn , os , HLHmdelta , stumps , arr , sob , CG7860 , dlg1 , br , mei-41 , spn-E , Lmpt , dlp , Nrg , ewg , Ark , Grip , mirr , raps , Idgf2 , dsx , Dr , Nc , so

positive regulation of nucleobase, nucleoside, nucleotide and nucleic acid metabolism

GOID	GOTerm	NodeSize	Exp.Count	Count	Pvalue	GeneSymb
GO:0045893	positive regulation of transcription, DNA-dependent	36	3.5	9	0.0057	dl , Eip93F , sno , lola , Trl , Bap170 , D , ara , dsx
GO:0045935	positive regulation of nucleobase, nucleoside, nucleotide and nucleic acid metabolism	48	4.6	10	0.014	dl , Eip93F , sno , lola , Trl , Bap170 , D , ara , mirr , dsx

heterophilic cell adhesion

GOID	GOTerm	NodeSize	Exp.Count	Count	Pvalue	GeneSymb
GO:0007157	heterophilic cell adhesion	9	0.87	4	0.0072	scb , pnt , alphaPS5 , Nrg

antimicrobial humoral response (sensu Protostomia)

GOID	GOTerm	NodeSize	Exp.Count	Count	Pvalue	GeneSymb
GO:0006966	antifungal humoral response (sensu Protostomia)	5	0.48	3	0.0076	Drs , nec , spz
GO:0006961	antibacterial humoral response (sensu Protostomia)	18	1.7	5	0.024	AttA , TepII , AttD , Def , Drs

equator specification

GOID	GOTerm	NodeSize	Exp.Count	Count	Pvalue	GeneSymb

GO:0045317	equator specification	5	0.48	3	0.0076	caup , ara , mirr

anti-Gram-positive bacterial polypeptide induction

GOID	GOTerm	NodeSize	Exp.Count	Count	Pvalue	GeneSymb
GO:0006965	anti-Gram-positive bacterial polypeptide induction	2	0.19	2	0.0092	PGRP-SA , Def

mitotic cell cycle, embryonic

GOID	GOTerm	NodeSize	Exp.Count	Count	Pvalue	GeneSymb
GO:0007348	regulation of progression through syncytial blastoderm mitotic cell cycle	2	0.19	2	0.0092	grp , mei-41
GO:0045448	mitotic cell cycle, embryonic	6	0.58	3	0.014	Trl , grp , mei-41

DNA damage checkpoint

GOID	GOTerm	NodeSize	Exp.Count	Count	Pvalue	GeneSymb
GO:0000077	DNA damage checkpoint	2	0.19	2	0.0092	grp , mei-41

cell-matrix adhesion

GOID	GOTerm	NodeSize	Exp.Count	Count	Pvalue	GeneSymb
GO:0007160	cell-matrix adhesion	21	2	6	0.012	NetA , bt , scb , alphaPS5 , CG33171 , CG31839

peptidoglycan catabolism

GOID	GOTerm	NodeSize	Exp.Count	Count	Pvalue	GeneSymb
GO:0009253	peptidoglycan catabolism	6	0.58	3	0.014	PGRP-LA , PGRP-SA , PGRP-LF

calcium ion transport

GOID	GOTerm	NodeSize	Exp.Count	Count	Pvalue	GeneSymb
GO:0006816	calcium ion transport	6	0.58	3	0.014	pain , Rya-r44F , Ca-P60A

positive regulation of cellular process

GOID	GOTerm	NodeSize	Exp.Count	Count	Pvalue	GeneSymb
GO:0048522	positive regulation of cellular process	130	12	20	0.017	mod(mdg4) , dl , CG10473 , Appl , Eip93F , sno , stan , lola , Trl , Bap170 , D , klu , ara , br , dlp , Ark , egr , mirr , dsx , Nc
GO:0043068	positive regulation of programmed cell death	48	4.6	9	0.036	mod(mdg4) , CG10473 , Appl , Eip93F , klu , br , Ark , egr , Nc

reproduction

GOID	GOTerm	NodeSize	Exp.Count	Count	Pvalue	GeneSymb
GO:0007276	gametogenesis	260	25	35	0.018	mod(mdg4) , dl , sqh , dup , sno , loco , Prat , zfh1 , Fas3 , Klp64D , Hmgcr , DAAM , cdi , pnt , sqz , Pvf1 , lbk , DnaJ-60 , os , mei-P26 , stumps , dlg1 , spir , mei-41 , Fs(2)Ket , spn-E , capu , kst , Ark , wbl , dsx , spz , wun2 , Nc , so
GO:0000003	reproduction	270	26	36	0.028	mod(mdg4) , dl , sqh , dup , nonA , sno , loco , Prat , zfh1 , Fas3 , Klp64D , Hmgcr , DAAM , cdi , pnt , sqz , Pvf1 , lbk , DnaJ-60 , os , mei-P26 , stumps , dlg1 , spir , mei-41 , Fs(2)Ket , spn-E , capu , kst , Ark , wbl , dsx , spz , wun2 , Nc , so

positive regulation of metabolism

GOID	GOTerm	NodeSize	Exp.Count	Count	Pvalue	GeneSymb
GO:0009893	positive regulation of metabolism	51	4.9	10	0.021	dl , Eip93F , sno , lola , Trl , Bap170 , D , ara , mirr , dsx

apoptosis

GOID	GOTerm	NodeSize	Exp.Count	Count	Pvalue	GeneSymb
GO:0008632	apoptotic program	12	1.2	4	0.022	Appl , Rep4 , Ark , Nc
GO:0042981	regulation of apoptosis	62	6	11	0.032	mod(mdg4) , Mmp1 , dl , CG10473 , Appl , Eip93F , pnt , CG7188 , Ark , egr , Nc

cell communication

GOID	GOTerm	NodeSize	Exp.Count	Count	Pvalue	GeneSymb
GO:0007154	cell communication	710	68	83	0.022	form3 , mod(mdg4) , dl , Appl , sqh , NetA , CG9000 , CCKLR-17D3 , sno , BM-40-SPARC , CG11835 , frc , HLHmgamma , stan , bt , CG15556 , CG6805 , bnl , lola , loco , Tob , Nplp1 , kal-1 , Nf1 , PGRP-SA , swi2 , DAAM , klu , cdi , CG7054 , grp , pnt , Pkc98E , CG10737 , Rep , CG30115 , CG11968 , CG6749 , lbk , CG33171 , CG4322 , l(1)G0232 , os , HLHmdelta , stumps , arr , RhoGAP18B , RacGAP50C , Stam , CG31839 , Tsp42El , dlg1 , br , Syb , CG10089 , psh , mthl2 , CG32843 , nec , Ca-P60A , Ptr , dlp , capu , Nrg , mthl3 , Socs36E , CG31640 , Hn , Rab10 , mas , ETH , lbm , synaptogyrin , CG5096 , CG3603 , egr , mirr , e , raps , Idgf2 , wbl , spz , wun2

GO:0019226	transmission of nerve impulse	140	14	21	0.033	mod(mdg4) , Appl , NetA , CCKLR-17D3 , lola , kal-1 , Pkc98E , Rep , CG6749 , lbk , Stam , Tsp42El , dlg1 , br , Syb , CG32843 , Ca-P60A , lbm , synaptogyrin , CG5096 , e

glucosamine metabolism

GOID	GOTerm	NodeSize	Exp.Count	Count	Pvalue	GeneSymb
GO:0006041	glucosamine metabolism	24	2.3	6	0.023	Tequila , CG11142 , CG32209 , CG12009 , CG32499 , CG1869

eye morphogenesis

GOID	GOTerm	NodeSize	Exp.Count	Count	Pvalue	GeneSymb
GO:0046668	regulation of retinal programmed cell death	7	0.67	3	0.023	klu , Ark , Nc
GO:0048592	eye morphogenesis	97	9.3	15	0.042	caup , sno , frc , stan , klar , klu , pnt , ara , tinc , arr , br , Ark , mirr , Nc , so

protein amino acid prenylation

GOID	GOTerm	NodeSize	Exp.Count	Count	Pvalue	GeneSymb
GO:0018346	protein amino acid prenylation	7	0.67	3	0.023	Rep , CG33171 , CG12007

detection of bacteria

GOID	GOTerm	NodeSize	Exp.Count	Count	Pvalue	GeneSymb
GO:0016045	detection of bacteria	3	0.29	2	0.026	PGRP-LA , PGRP-SA

cell cycle arrest

GOID	GOTerm	NodeSize	Exp.Count	Count	Pvalue	GeneSymb
GO:0007050	cell cycle arrest	3	0.29	2	0.026	grp , CG11299

central nervous system development

GOID	GOTerm	NodeSize	Exp.Count	Count	Pvalue	GeneSymb
GO:0035193	central nervous system remodeling (sensu Insecta)	3	0.29	2	0.026	cib , br
GO:0007417	central nervous system development	66	6.1	11	0.035	dl , sc , stan , sad , zfh1 , D , cib , dlg1 , br , Nrg , ewg , dsx , Dr

coagulation

GOID	GOTerm	NodeSize	Exp.Count	Count	Pvalue	GeneSymb

GO:0050819	negative regulation of coagulation	3	0.29	2	0.026	AnnX , AnnIX
GO:0050817	coagulation	4	0.38	2	0.049	AnnX , AnnIX

secondary tracheal branching (sensu Insecta)

GOID	GOTerm	NodeSize	Exp.Count	Count	Pvalue	GeneSymb
GO:0007429	secondary tracheal branching (sensu Insecta)	3	0.29	2	0.026	bnl , pnt

nucleotide catabolism

GOID	GOTerm	NodeSize	Exp.Count	Count	Pvalue	GeneSymb
GO:0009166	nucleotide catabolism	3	0.29	2	0.026	CG11883 , CG6330

sex determination, establishment of X:A ratio

GOID	GOTerm	NodeSize	Exp.Count	Count	Pvalue	GeneSymb
GO:0007540	sex determination, establishment of X:A ratio	3	0.29	2	0.026	sc , os

short-term memory

GOID	GOTerm	NodeSize	Exp.Count	Count	Pvalue	GeneSymb
GO:0007614	short-term memory	3	0.29	2	0.026	Nf1 , scb

ectoderm development

GOID	GOTerm	NodeSize	Exp.Count	Count	Pvalue	GeneSymb
GO:0007398	ectoderm development	120	12	19	0.028	dl , Appl , caup , NetA , BM-40-SPARC , HLHmgamma , stan , zfh1 , D , ara , Sox15 , HLHmdelta , Tsp42El , Nrg , ewg , CG5397 , lbm , mirr , Dr

biological_process

GOID	GOTerm	NodeSize	Exp.Count	Count	Pvalue	GeneSymb
GO:0035075	response to ecdysone	13	1.2	4	0.03	Eip93F , swi2 , Kr-h1 , br
GO:0035081	induction of programmed cell death by hormones	9	0.87	3	0.048	Eip93F , br , Nc

cell migration

GOID	GOTerm	NodeSize	Exp.Count	Count	Pvalue	GeneSymb
GO:0016477	cell migration	110	11	17	0.032	mod(mdg4) , dl , sqh , NetA , bnl , lola , zfh1 , Fas3 , Hmgcr , scb , Pvf2 , Pvf1 , os , stumps , Tig , wun2 , so
GO:0007280	pole cell migration	9	0.87	3	0.048	zfh1 , Hmgcr , wun2

sensory perception

GOID	GOTerm	NodeSize	Exp.Count	Count	Pvalue	GeneSymb
GO:0007600	sensory perception	62	6	11	0.032	Obp56a , nonA , CG11835 , d , D , pain , Gr94a , CG9296 , CG15629 , Obp99a , mas
GO:0007601	visual perception	35	3.4	7	0.045	nonA , CG11835 , d , D , CG9296 , CG15629 , mas

nucleoside monophosphate biosynthesis

GOID	GOTerm	NodeSize	Exp.Count	Count	Pvalue	GeneSymb
GO:0009124	nucleoside monophosphate biosynthesis	8	0.77	3	0.034	Adgf-A , Ts , Prat

di-, tri-valent inorganic cation homeostasis

GOID	GOTerm	NodeSize	Exp.Count	Count	Pvalue	GeneSymb
GO:0030005	di-, tri-valent inorganic cation homeostasis	9	0.87	3	0.048	Tsf1 , Rya-r44F , Ca-P60A

antifungal polypeptide induction

GOID	GOTerm	NodeSize	Exp.Count	Count	Pvalue	GeneSymb
GO:0006967	antifungal polypeptide induction	4	0.38	2	0.049	psh , spz

basal protein localization

GOID	GOTerm	NodeSize	Exp.Count	Count	Pvalue	GeneSymb
GO:0045175	basal protein localization	4	0.38	2	0.049	dlg1 , raps

cellularization (sensu Metazoa)

GOID	GOTerm	NodeSize	Exp.Count	Count	Pvalue	GeneSymb
GO:0009796	cellularization (sensu Metazoa)	4	0.38	2	0.049	grp , mei-41

amino acid transport

GOID	GOTerm	NodeSize	Exp.Count	Count	Pvalue	GeneSymb
GO:0006865	amino acid transport	15	1.4	4	0.049	CG5535 , CG31547 , CG1139 , path

MF GO Analysis

pattern binding

GOID	GOTerm	NodeSize	Exp.Count	Count	Pvalue	GeneSymb
GO:0001871	pattern binding	25	1.9	7	0.0017	Tequila , PGRP-LA , CG11142 , PGRP-SA , Pvf1 , CG32209 , PGRP-LF , CG12009 , CG32499 , CG1869
GO:0042834	peptidoglycan binding	6	0.6	3	0.016	PGRP-LA , PGRP-SA , PGRP-LF

carbohydrate binding

GOID	GOTerm	NodeSize	Exp.Count	Count	Pvalue	GeneSymb
GO:0030246	carbohydrate binding	34	2	7	0.0024	Tequila , CG11142 , CG5335 , Pvf1 , CG32209 , CG12009 , CG32499 , CG6055 , CG3244 , CG4115 , CG1869
GO:0005529	sugar binding	14	1.4	4	0.045	CG5335 , CG6055 , CG3244 , CG4115

transmembrane receptor activity

GOID	GOTerm	NodeSize	Exp.Count	Count	Pvalue	GeneSymb
GO:0001584	rhodopsin-like receptor activity	31	3.1	9	0.0026	CCKLR-17D3 , CG11835 , Gr94a , CG4322 , l(1)G0232 , mthl2 , CG32843 , mas , CG3603
GO:0004888	transmembrane receptor activity	110	11	21	0.0041	w , Tequila , CCKLR-17D3 , CG11835 , stan , CG15556 , Gr94a , scb , CG6749 , Ect4 , CG4322 , l(1)G0232 , arr , CG5888 , mthl2 , CG32843 , Ptr , mthl3 , mas , CG3603 , CG6739

hydrolase activity, acting on carbon-nitrogen (but not peptide) bonds

GOID	GOTerm	NodeSize	Exp.Count	Count	Pvalue	GeneSymb
GO:0016810	hydrolase activity, acting on carbon-nitrogen (but not peptide) bonds	43	4.3	11	0.0027	Adgf-A , PGRP-LA , PGRP-SA , HDAC6 , CG32209 , PGRP-LF , CG6428 , CG7860 , nemy , CG32499 , Nmdmc
GO:0008745	N-acetylmuramoyl-L-alanine amidase activity	6	0.6	3	0.016	PGRP-LA , PGRP-SA , PGRP-LF

glucuronosyltransferase activity

GOID	GOTerm	NodeSize	Exp.Count	Count	Pvalue	GeneSymb
GO:0015020	glucuronosyltransferase activity	11	1.1	5	0.0028	Ugt86Da , Ugt35a , GlcAT-S , Ugt58Fa , Ugt86Di

structural constituent of cuticle

GOID	GOTerm	NodeSize	Exp.Count	Count	Pvalue	GeneSymb
GO:0005214	structural constituent of cuticle (sensu Insecta)	32	3.2	9	0.0033	Pcp , CG15008 , Edg78E , CG8511 , CG8502 , CG8515 , CG9077 , Lcp65Ad , CG4818
GO:0042302	structural constituent of cuticle	44	1.2	5	0.0044	Pcp , CG12255 , CG15008 , Edg78E , CG8511 , CG12045 , CG6131 , CG8502 , CG8515 , CG9077 , Lcp65Ad , CG8634 , CG7160 , CG4818

growth factor activity

GOID	GOTerm	NodeSize	Exp.Count	Count	Pvalue	GeneSymb
GO:0008083	growth factor activity	16	1.6	6	0.0033	Adgf-A , BM-40-SPARC , bnl , Pvf2 , Pvf1 , Idgf2

calcium-dependent phospholipid binding

GOID	GOTerm	NodeSize	Exp.Count	Count	Pvalue	GeneSymb
GO:0005544	calcium-dependent phospholipid binding	4	0.4	3	0.0037	CG7632 , AnnX , AnnIX

serine-type endopeptidase inhibitor activity

GOID	GOTerm	NodeSize	Exp.Count	Count	Pvalue	GeneSymb
GO:0004867	serine-type endopeptidase inhibitor activity	27	2.7	8	0.0039	Spn4 , CG7722 , CG9460 , TepII , CG2816 , nec , Spn43Aa , CG31777

chitin binding

GOID	GOTerm	NodeSize	Exp.Count	Count	Pvalue	GeneSymb
GO:0008061	chitin binding	17	1.7	6	0.0047	Tequila , CG11142 , CG32209 , CG12009 , CG32499 , CG1869

monooxygenase activity

GOID	GOTerm	NodeSize	Exp.Count	Count	Pvalue	GeneSymb
GO:0004497	monooxygenase activity	34	3.4	9	0.0051	sad , Cyp4e2 , Cyp6a17 , Cyp6d4 , Cyp6a20 , Cyp301a1 , Cyp6d2 , Cyp4d2 , Hn

cytokine activity

GOID	GOTerm	NodeSize	Exp.Count	Count	Pvalue	GeneSymb
GO:0005125	cytokine activity	12	0.91	4	0.0084	bnl , Pvf2 , Pvf1 , os , egr , spz

GO:0005172	vascular endothelial growth factor receptor binding	3	0.3	2	0.028	Pvf2 , Pvf1

signal transducer activity

GOID	GOTerm	NodeSize	Exp.Count	Count	Pvalue	GeneSymb
GO:0016015	morphogen activity	5	0.5	3	0.0086	dl , os , spz
GO:0004871	signal transducer activity	410	41	54	0.014	dl , Appl , Adgf-A , w , Tequila , NPC2 , CCKLR-17D3 , BM-40-SPARC , CG11835 , stan , bt , CG15556 , bnl , loco , Nplp1 , Nf1 , PGRP-SA , DAAM , Gr94a , cdi , scb , grp , alphaPS5 , Pvf2 , Pkc98E , Pvf1 , CG6749 , Ect4 , CG4322 , Rya-r44F , l(1)G0232 , os , arr , RacGAP50C , CG5888 , Stam , CG31839 , Tsp42El , dlg1 , mei-41 , mthl2 , CG32843 , Ptr , CG11168 , mthl3 , CG31640 , Grip , mas , ETH , lbm , CG9593 , CG5096 , CG3603 , egr , Idgf2 , spz , CG6739

vitamin E binding

GOID	GOTerm	NodeSize	Exp.Count	Count	Pvalue	GeneSymb
GO:0008431	vitamin E binding	5	0.5	3	0.0086	CG13848 , CG10026 , CG3091

aldehyde reductase activity

GOID	GOTerm	NodeSize	Exp.Count	Count	Pvalue	GeneSymb
GO:0004032	aldehyde reductase activity	5	0.5	3	0.0086	CG10638 , CG9436 , CG10863

protease inhibitor activity

GOID	GOTerm	NodeSize	Exp.Count	Count	Pvalue	GeneSymb
GO:0030414	protease inhibitor activity	37	3.7	9	0.0093	Spn4 , CG7722 , CG9460 , CG8066 , TepII , CG2816 , nec , Spn43Aa , CG31777

heparin binding

GOID	GOTerm	NodeSize	Exp.Count	Count	Pvalue	GeneSymb
GO:0008201	heparin binding	2	0.2	2	0.01	Pvf1 , CG1869

trypsin activity

GOID	GOTerm	NodeSize	Exp.Count	Count	Pvalue	GeneSymb
GO:0004295	trypsin activity	59	5.9	12	0.013	CG10041 , Tequila , CG8170 , CG1102 , CG13744 , CG6041 , CG9372 , CG31412 , CG31728 , psh , CG4386 , mas

chymotrypsin activity

GOID	GOTerm	NodeSize	Exp.Count	Count	Pvalue	GeneSymb
GO:0004263	chymotrypsin activity	54	5.4	11	0.017	CG10041 , Tequila , CG8170 , CG1102 , CG13744 , CG6041 , CG9372 , CG31728 , psh , CG4386 , mas

lipid binding

GOID	GOTerm	NodeSize	Exp.Count	Count	Pvalue	GeneSymb
GO:0008289	lipid binding	35	3.5	8	0.02	CG7632 , AnnX , CG7054 , NLaz , Pkc98E , CG10737 , RacGAP50C , AnnIX

amino acid-polyamine transporter activity

GOID	GOTerm	NodeSize	Exp.Count	Count	Pvalue	GeneSymb
GO:0005279	amino acid-polyamine transporter activity	12	1.2	4	0.026	CG5535 , CG31547 , CG1139 , path

ligand-gated ion channel activity

GOID	GOTerm	NodeSize	Exp.Count	Count	Pvalue	GeneSymb
GO:0015276	ligand-gated ion channel activity	7	0.7	3	0.026	rpk , CG2657 , Rya-r44F

triacylglycerol lipase activity

GOID	GOTerm	NodeSize	Exp.Count	Count	Pvalue	GeneSymb
GO:0004806	triacylglycerol lipase activity	7	0.7	3	0.026	CG6847 , CG7367 , CG6753

glutathione transferase activity

GOID	GOTerm	NodeSize	Exp.Count	Count	Pvalue	GeneSymb
GO:0004364	glutathione transferase activity	24	2.4	6	0.028	GstE1 , GstE5 , GstE6 , GstE9 , GstD2 , GstD3

organic cation transporter activity

GOID	GOTerm	NodeSize	Exp.Count	Count	Pvalue	GeneSymb
GO:0015101	organic cation transporter activity	8	0.8	3	0.038	Orct , CG8654 , CG6126

serine-type peptidase activity

GOID	GOTerm	NodeSize	Exp.Count	Count	Pvalue	GeneSymb
GO:0008236	serine-type peptidase activity	92	9.3	15	0.039	CG10041 , Tequila , CG8170 , CG1102 , CG13744 , cutlet , CG6041 , CG9372 , CG2145 , CG31412 , CG31728 , psh , CG11883 , CG4386 , mas

carboxylic ester hydrolase activity

GOID	GOTerm	NodeSize	Exp.Count	Count	Pvalue	GeneSymb
GO:0016789	carboxylic ester hydrolase activity	40	4	8	0.042	CG6847 , CG18641 , CG10175 , CG4267 , CG6428 , CG4757 , CG7367 , CG6753
